# The transgenerational effects of oocyte mitochondrial supplementation

**DOI:** 10.1038/s41598-019-43135-4

**Published:** 2019-04-30

**Authors:** Justin C. St. John, Yogeshwar Makanji, Jacqueline L. Johnson, Te-Sha Tsai, Simone Lagondar, Fleur Rodda, Xin Sun, Mulyoto Pangestu, Penny Chen, Peter Temple-Smith

**Affiliations:** 1 Hudson Institute of Medical Research, 27-31 Wright Street, Clayton, Vic 3168 Australia; 2 Department of Molecular and Translational Science, Monash University, 27-31 Wright Street, Clayton, Vic 3168 Australia; 3 Education Program in Reproduction and Development, Department of Obstetrics and Gynaecology, Department of Obstetrics & Gynaecology, School of Clinical Sciences, Monash University, Clayton, VIC 3168 Australia; 4 Monash Animal Research Platform, Monash University, Clayton, VIC 3168 Australia

**Keywords:** Embryology, Development

## Abstract

Many women suffer from either failed fertilisation or their embryos arrest early during development. Autologous mitochondrial supplementation has been proposed as an assisted reproductive technology to overcome these problems. However, its safety remains to be tested in an animal model to determine if there are transgenerational effects. We have supplemented oocytes with autologous populations of mitochondria to generate founders. We mated the female founders and their offspring to produce three generations. We assessed litter size, the ovarian reserve, and weight gain and conducted a full histopathological analysis from each of the three generations. Across the generations, we observed significant increases in litter size and in the number of primordial follicles in the ovary matched by changes in global gene expression patterns for these early-stage oocytes. However, full histopathological analysis revealed that cardiac structure was compromised in first and second generation offspring, which could seriously affect the health of the offspring. Furthermore, the offspring were prone to increased weight gain during early life. Mitochondrial supplementation appears to perturb the regulation of the chromosomal genome resulting in transgenerational phenotypic gains and losses. These data highlight the need for caution when using autologous mitochondrial supplementation to treat female factor infertility.

## Introduction

An increasing number of couples are seeking assisted reproductive treatment as they fail to achieve a pregnancy of which approximately 61% of the female partners are over the age of 35^1,2^. Many suffer from either failed fertilisation or their embryos arrest during preimplantation development, which can persist through several rounds of treatment^1^. Although a number of causes have been cited including aneuploidy^3^, it has become increasingly evident that this failure also arises from the oocytes of these patients having too few copies of the maternally-only inherited mitochondrial genome (mtDNA)^4,5^.

mtDNA is located in each of the cell’s mitochondria and encodes 13 subunits of the electron transfer chain, 22 tRNAs and 2 rRNAs^6^. It has one major non-coding region, the D-Loop that interacts with nuclear-encoded factors to transcribe and replicate mtDNA. MtDNA replication is strictly regulated during development. During oogenesis, the primordial germ cells differentiate into mature, metaphase II oocytes. Each primordial germ cell possesses ~200 copies of mtDNA^7^, which are clonally expanded at later stages to provide mature metaphase II oocytes with >150,000 copies of mtDNA^7,8^ to surpass the threshold required for successful fertilisation outcome. Studies in pigs^8,9^, cattle^10^ and humans^4,5,11,12^ highlight how sufficient numbers of mtDNA copy promote fertilisation outcome and development. Indeed, human oocytes with fewer than 100,000 copies of mtDNA have significantly lower rates of fertilisation than oocytes with greater than 150,000 copies^4,5,11^.

Autologous mitochondrial transfer has been proposed as an assisted reproductive technology to ensure sufficient copies of mtDNA are present to support post-fertilisation developmental events in embryos until post-gastrulation when mtDNA replication is initiated^13,14^. Autologous transfer would not compromise the genetic identity of the offspring as is the case when third party or ‘donor’ mitochondria are used in an approach commonly referred to as ‘3-parent IVF’^15^. Furthermore, the use of ‘3-parent IVF’ in both mouse models and humans has been associated with a number of disorders^16,17^ that have resulted in this approach being banned by regulators in many countries.

In a pig model of mtDNA deficiency, it has been shown that the introduction of autologous mtDNA (~790 copies) increased mtDNA copy number by four-fold at the 2-cell stage and there was reduced gene expression associated with metabolic disorders, such as obesity and diabetes, by the blastocyst stage^18,19^. Likewise, in a bovine model of somatic cell nuclear transfer, it has been shown that oocytes with sufficient mtDNA also benefited from mtDNA supplementation (~560 copies) with there being increased levels of expression of genes involved in glycolysis and decreased levels of expression of genes involved in embryonic death^20^, which promote cell proliferation and embryonic development. In addition, the blastocyst-stage embryos possessed significantly higher numbers of mtDNA copy when compared with non-supplemented counterparts^20^.

As oocytes for assisted reproduction are normally collected following hormonal stimulation or less frequently through *in vitro* maturation and the mtDNA content of an oocyte cannot be directly assessed prior to treatment, we have supplemented superovulated mouse oocytes at the time of fertilisation with autologous populations of mitochondria to generate founders. This enabled us to determine if the transfer of additional mtDNA is safe practice and if subsequent generations are compromised, irrespective of whether the oocyte has sufficient mtDNA or not. We mated the female founders and their female offspring to produce three generations of offspring and analysed litter size, the ovarian reserve, and weight gain during neonatal development, and conducted a full histopathological analysis of the three generations of offspring. We found that autologous supplementation enhanced fertility but led to structural defects in cardiac tissue suggesting that the regulation of chromosomal gene expression that is established during oogenesis is perturbed by this process.

## Results

To generate founders, superovulated oocytes from F1: C57BL6/J/MARP × CBA/MARP females were supplemented with an isolate of mitochondria from the same strain at the same time as a spermatozoon obtained from the same strain of proven stud colony males was introduced into the oocyte by intracytoplasmic sperm injection (ICSI). The mitochondria were isolated from egg precursor cells (EPCs), which are putative naïve germ cells that are present in the ovarian cortex and can give rise to oocytes^21^. Each mitochondrial isolate was 3 pl in volume and contained 530 ± 28.8 (mean ± SEM) copies of mtDNA, which is the largest volume that can be introduced into an oocyte without inducing harmful effects. Given that the mature, metaphase II oocyte from the F1: C57BL6/J/MARP x CBA/MARP strain contains 154,251 ± 9021 (mean ± SEM) copies of mtDNA, the mtDNA introduced into the oocyte represents 0.34% of the oocyte’s total mtDNA content at the time of fertilisation.

**Figure 1:**
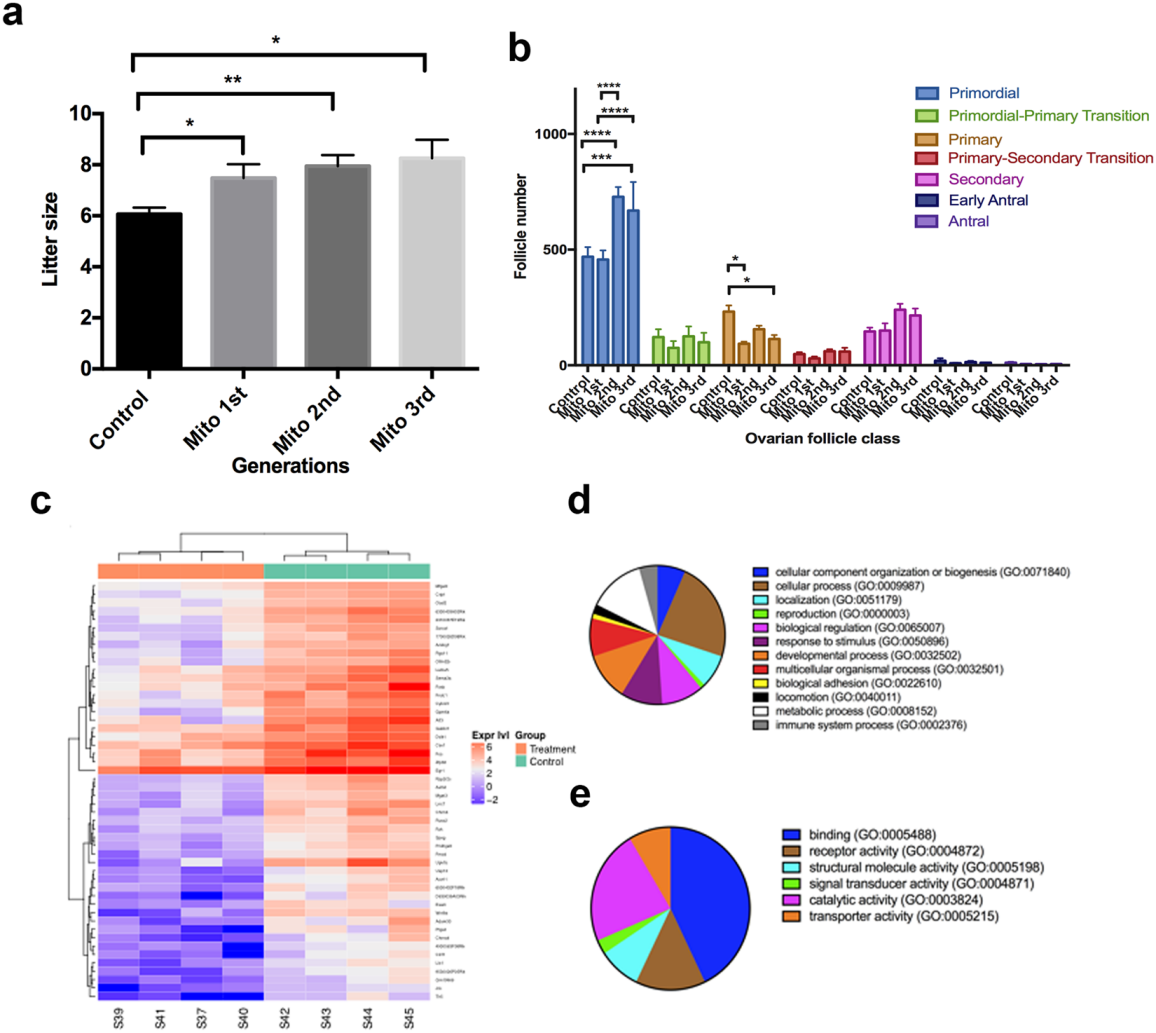
The effects of mitochondrial supplementation on litter size and the ovarian reserve. (**a**) Assessment of litter size (mean ± SEM) over three generations (1^st^, 2^nd^ and 3^rd^) of the offspring derived from founders generated through mitochondrial supplementation (Mito 1^st^ – n = 21 litters; Mito 2^nd^ – n = 19 litters; and Mito 3^rd^ – n = 8 litters). Litter sizes were compared to those from colony control mice (n = 93 litters). (**b**) Assessment of follicle number (mean ± SEM) from three generations of ten week old mice (Mito 1^st^; Mito 2^nd^; and Mito 3^rd^) generated from founders derived through mitochondrial supplementation and compared to colony controls (control). Primordial, Primordial-Primary Transition, Primary, Primary–Secondary Transition, Secondary, Early Antral, and Antral stages were assessed. (**c**) Heat map of RNA-Seq profiles. Total identified differentially expressed genes (FDR ≤ 0.05, absolute logFC ≥ 1) for third generation MITO supplemented (treatment; n = 4) and control mice (n = 4) were plotted based on the fold changes (log2) of read counts. ‘Expr lvl’ = expression levels. (**d**) PANTHER Gene Ontology Classification analysis for biological processes. (**e**) PANTHER Gene Ontology Classification analysis for molecular functions. *P < 0.05, **P < 0.01, ***P < 0.001, ****P < 0.0001.

Zygotes that progressed to the 2-cell stage of preimplantation development were transferred into pseudopregnant surrogate females. From three transfers, seven founder females and one male were generated. Once the founder females had reached sexual maturity, they were mated with proven stud colony males to determine their fertility using litter size as a readout, which is an important indicator of fertility in multiparous species such as mice^22^. The first litter from each of the seven founders produced litter sizes of 6.57 ± 0.48 (mean ± SEM; Supplementary Fig. 1a). We again mated the founders with colony males to produce a further four parities from three of the founders. There were no significant differences between each of the parities (Supplementary Fig. 1a). Collectively, the parities from the first generation produced a mean litter size of 7.48 ± 0.55 (Fig. 1a), which was significantly larger than colony controls conceived through natural matings (6.07 ± 0.26; p < 0.05; Fig. 1a). Female offspring from the first generation were mated with colony males to produce a second generation of offspring comprising three parities. There were no significant differences in litter size amongst the parities (Supplementary Fig. 1b). However, litter size for the second generation was also significantly larger than colony controls (7.95 ± 0.44; p < 0.01; Fig. 1a) but not to the first generation. Finally, we mated the females from the second generation to produce a third generation comprising one parity. The mean litter size for the third generation (8.25 ± 0.73; Fig. 1a) was also significantly higher than the colony controls (p < 0.05) but not higher than the first and second generations (p > 0.05). We conclude that the introduction of additional mtDNA into superovulated oocytes results in a transgenerational enhancement of fertility, as demonstrated through increased litter size.

To determine if the increased litter size resulted from changes to the size of the ovarian reserve, we assessed the number of follicles present at the primordial, primordial-primary transition, primary, primary to secondary transition, secondary, early antral and antral stages in ten-week-old mice. For the second and third generations, we observed significant increases in the primordial follicle stages against both the control (range p < 0.001 to p < 0.0001) and first (p < 0.0001) generation mice (Fig. 1b; Supplementary Fig. 2). Furthermore, we observed reductions in primary follicles compared with controls for each of the generations, which were significant in the first (p < 0.05) and third (p < 0.05) generations.

As the third generation produced the largest litter sizes and significantly higher primordial follicle counts, we performed RNA-Seq on primordial follicles from day 3 ovaries from this generation. In all, we identified 53 differentially expressed genes between the primordial follicles of third generation mice and controls based on >absolute 2-fold differences and an adjusted p value < 0.05, which are presented in a heat map (Fig. 1c). Using the Panther Ontology Classification System, cellular, metabolic and developmental processes were the most modified biological processes (Fig. 1d), whilst binding, catalytic activity and receptor activity were the molecular functions most modified (Fig. 1e). Collectively, these data indicate that mitochondrial supplementation at the time of fertilisation can act on the germline to modify chromosomal gene expression profiles that can influence primordial follicle numbers, which, in turn, results in a transgenerational increase in litter size and to phenotypically promote fertility.

**Figure 2:**
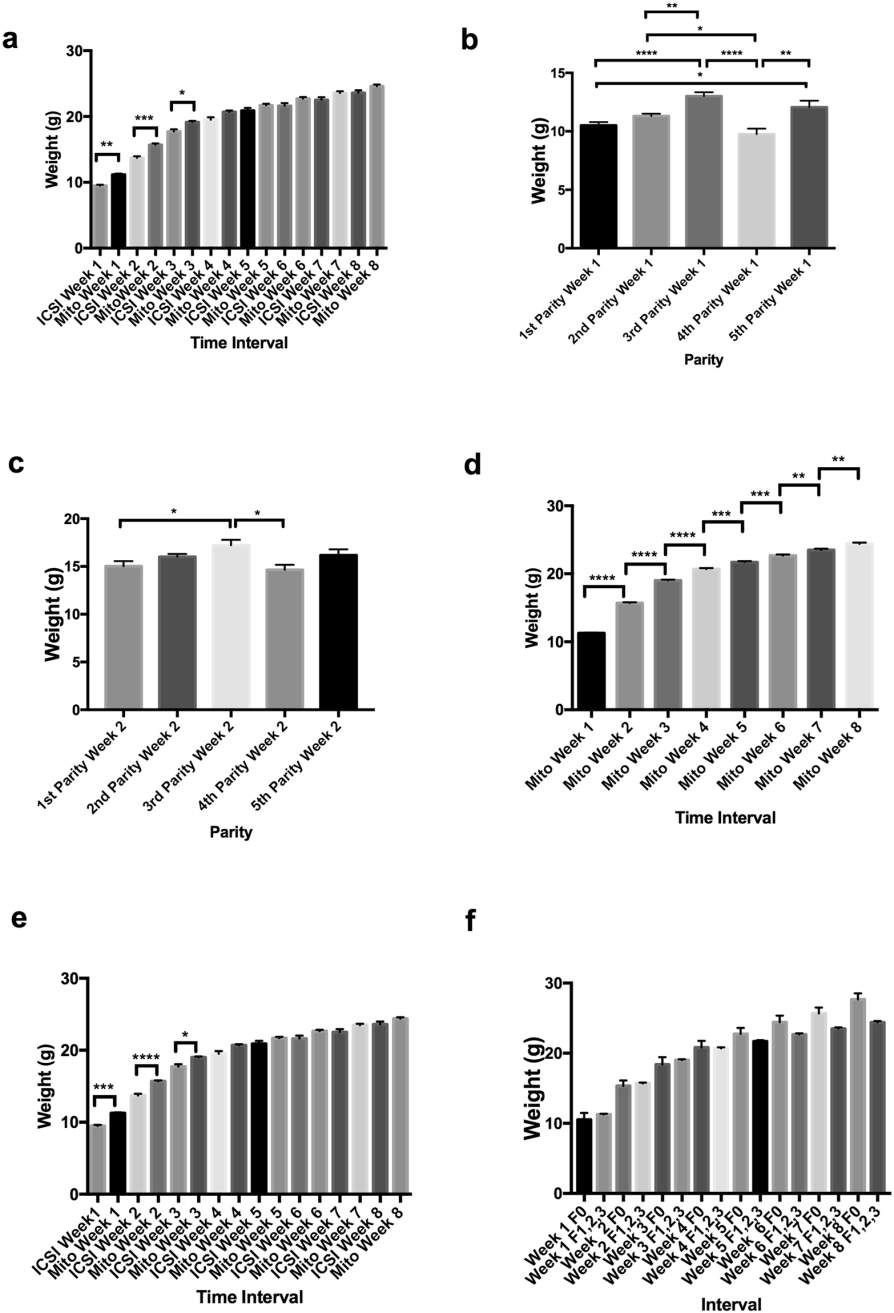
Assessment of weight gain in mitochondrial supplemented mice. Mice were assessed over eight weeks (weeks 1 to 8) after birth. (**a**) Weight gain in first generation mitochondrial supplemented mice (all parities) compared with ICSI-derived mice. (**b**) Weights at week 1 over five parities from first generation mitochondrial supplemented mice. (**c**) Weights at week 2 over five parities from first generation mitochondrial supplemented mice. (**d**) Total values for first, second and third generation mitochondrial supplemented mice. (**e**) Total values for first, second and third generation mitochondrial supplemented mice compared with ICSI mice. (**f**) Mitochondrial supplemented founders (F0) compared with 1^st^ (F1), 2^nd^ (F2), and 3^rd^ (F3) generation mice. *P < 0.05, **P < 0.01, ***P < 0.001, ****P < 0.0001. All values are mean ± SEM.

To determine if the unexpected enhancement of one phenotypic trait would impact on other phenotypic traits, we conducted physiological and anatomical assessments of females from each of the three generations. Firstly, we weighed offspring after the first week of birth over eight consecutive weeks. For the first generation of offspring derived from mitochondrial supplementation (inclusive of parities 1 to 5), we observed significant incremental increases in weight from weeks 1 to 2 (p < 0.0001), 2 to 3 (p < 0.0001), 3 to 4 (p < 0.001), and 5 to 6 (p < 0.05) (Supplementary Fig. 3a). Similar incremental increases were observed in mice derived through ICSI, although weeks 5 to 6 were not significant (Supplementary Fig. 3b). A comparison between mitochondrial supplemented- and ICSI-derived mice showed that the mice derived from mitochondrial-supplemented founders were significantly heavier in weeks 1 (p < 0.01), 2 (p < 0.001) and 3 (p < 0.05) (Fig. 2a). However, there were no differences from week 4 onwards, although the trend was for the mitochondrial-supplemented mice to be heavier. For each of the five parities from the first generation of mitochondrial-supplemented mice, we observed significant differences in weeks 1 (Figs. 2b), 2 (Fig. 2c) and 4 (Supplementary Fig. 3c) with the third parity being predominately heavier. The most significantly affected time period during development was week 1 (*cf* Fig. 2b,c, Supplementary Fig. 3c). Interestingly, the trends set in the first generation were maintained across the second and third generations as there were no significant differences in weight following each weekly recording (Supplementary Fig. 3d), although there were incremental increases from week to week when the values from each generation were combined (Fig. 2d). When the combined values for the three generations were compared with ICSI-derived offspring, there were once more significant differences at weeks 1, 2 and 3 with mitochondrial supplemented mice being heavier (Fig. 2e). Differences in patterns of neonatal weight gain were not observed between the mitochondrial-supplemented founders and their three generations of offspring. We found a significant difference in weight for founders between weeks 1 and 2 (p < 0.05; Supplementary Fig. 4a) but did not observe differences between ICSI and mitochondrial-supplemented founders (Supplementary Fig. 4b) and between mitochondrial-supplemented founders and the first generation of their offspring (Supplementary Fig. 4c) and all three generations (Fig. 2f). This suggests that the inherited weight gains observed in mitochondrial-supplemented offspring are present in the founders and transmitted through to subsequent generations.

**Figure 3:**
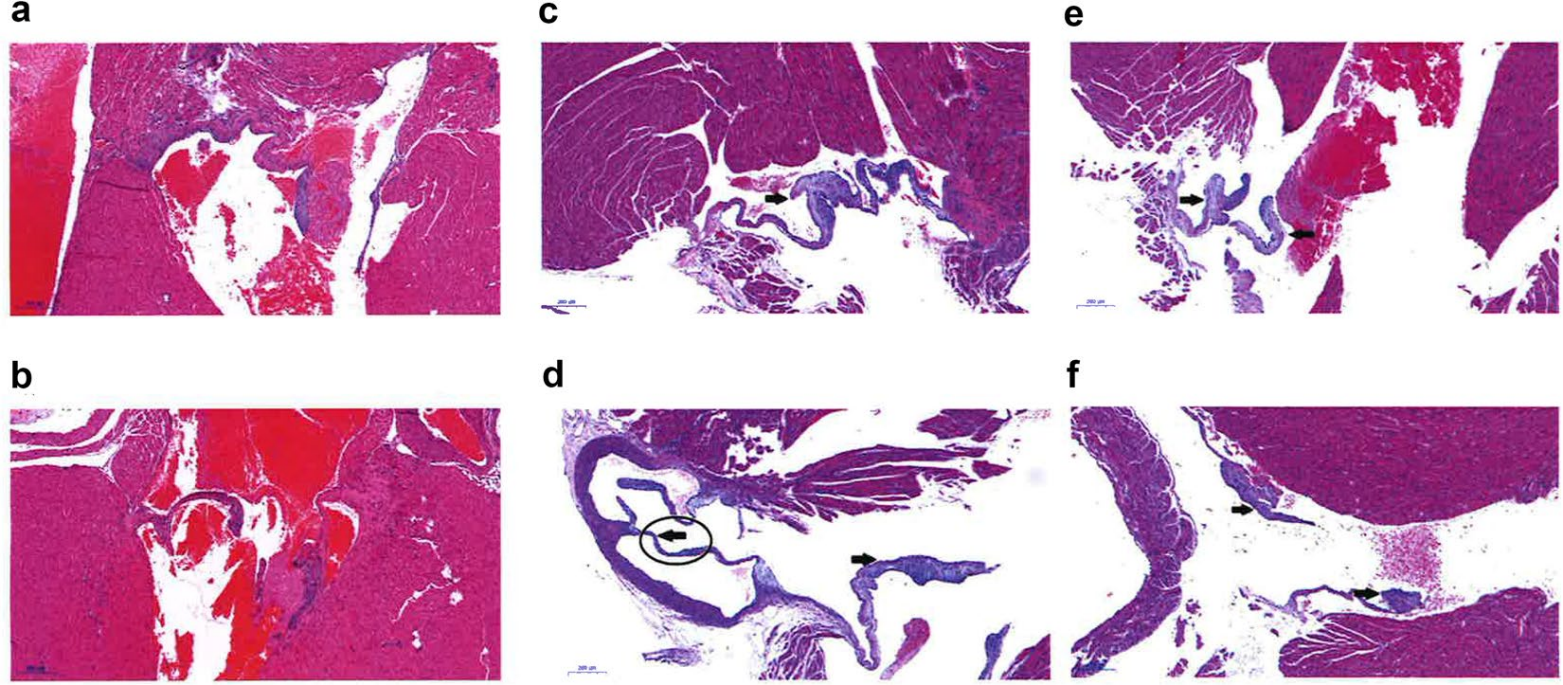
Heart valve defects. (**a**,**b**) Haematoxylin and Eosin staining of normal heart valves in control mice. (**c**–**f**) Thickened heart valves with myxomatous stroma, as indicated by arrows in four first generation mice derived from mitochondrial supplemented founders. Scale bar = 200 μm.

As we observed differences in weights resulting from mitochondrial supplementation, we subjected three adult female mice from each of the three generations and three age matched control mice to histopathological analysis. Macroscopic observation revealed that the three generations of mice arising from the founders derived from mitochondrial supplementation and controls all appeared to be well nourished, active and healthy with no evidence of illness or lesions (Supplementary File 1). Furthermore, all organs were normal in size and appearance when compared to controls. In general, the organs examined in all mitochondrial-supplemented mice appeared pathologically normal when compared to, both, control mice and the recorded literature. Some minor anomalies were identified such as a thymic cyst in one animal; mild hyperplasia and inflammation in the stomach of another; and evidence of inflammation in the kidney of a third animal. Animals from all three generations also exhibited some mild extramedullary haematopoiesis in the spleen, which is a common finding in mice^23^. However, a major abnormality was observed in all three of the first generation mice and one from the second generation. They typically displayed lesions of, and thickening to, the connective tissue of the atria-ventricular valves (Fig. 3). These changes in valvular pathology suggest that with age, complications may arise. To confirm this finding, a further four hearts from the cohort of first generation mice derived from mitochondrial supplementation were micro-pathologically assessed. Whilst the cardiac muscle, including muscle fibres, chambers and vessels of these mice showed typical micromorphology, as seen in the three first generation and one second generation mice examined previously, all four hearts displayed varying degrees of severity of mucoid degeneration of the valves (Supplementary File 2; *cf* Fig. 3a–f). Although this defect has been reported in mice^24^, it was limited to the offspring derived from the mitochondrial supplemented founders in our study. This defect could lead to severe heart abnormalities later in life that, frequently, in humans require surgery to correct. In all, these histopathological assessments indicate several minor defects and the generational transmission of a potentially severe heart defect as a result of mitochondrial supplementation, although the heart defect did not appear to be present in the third generation.

To ascertain that the phenotypic changes did not arise from a third party, genetically diverse source of mtDNA, we performed Next Generation mtDNA Sequencing on day 3 primordial follicles from third generation offspring and isolates of mitochondria extracted from EPCs used to supplement the oocytes which generated the founders. Primordial follicles comprise the early stage oocytes that have the potential to mature into fertilisable oocytes and, thus carry the mtDNA that is transmitted through the maternal lineage to subsequent generations. After mapping the reads against a reference genome, and then deriving a consensus sequence for each sample, which is indicative of the most frequently called base at each site, we observed differences in mtDNA sequence for each set of primordial follicles and EPC mitochondrial isolates. These were at nt9348 (G → A), nt9461 (T → C), nt9829 (InsA) and nt9830 (InsA) (Supplementary File 3). However, analysis of all reads showed these were sites exhibiting varying degrees of heteroplasmy. Of these variants, nt9348 (G → A) was predicted to be damaging following analysis using Panther’s Evolutionary Analysis of Coding SNPs (Supplementary Table 1). Moreover, we identified seven other sites with lower levels of heteroplasmy, which had frequencies for the minor base called at 3.1 to 23.6% (Supplementary Table 2). Of these variants, 12581(C → A) was predicted to be damaging although its level of heteroplasmy was only 9.8 ± 1.75 (Supplementary Table 1). This outcome indicates that the mtDNA populations were from the same maternal lineage. However, it is evident from the loss and gain of mtDNA variants between the EPC and the primordial follicles, both fixed and at low levels, that there is preferential selection for and against certain variants, which could account for the heart defect or minor anomalies identified through pathology. This is certainly the case for the Insertion/deletion at nt9821 (P < 0.001) and the single nucleotide variant at nt12581 (P < 0.0001).

## Discussion

The number of mtDNA copies present in the oocyte at fertilisation is regarded as an essential genetic investment in embryonic development^14^. This is exemplified by events during pig preimplantation development where there are very significant reductions in mtDNA copy number^8^, with each newly divided embryonic cell having fewer copies of mtDNA^9^. Studies in human embryos highlight this process whereby there appears to be an active process of shedding mtDNA, which promotes development to the blastocyst stage, the final stage of preimplantation development, and implantation^25^. The inner cell mass cells, which form the embryo proper and the foetus, continue to reduce mtDNA copy number^7^ as they remain in an uncommitted state through to gastrulation, which is when differentiation first starts, and establish the ‘mtDNA set point’^26,27^. The mtDNA set point comprises a small founder population of mtDNA that segregates to all cells of the body. This population of mtDNA is maintained at low levels as undifferentiated cells progress to organogenesis, when tissues and organs first form, and is then replicated in a cell specific manner. As a result, each mature cell type acquires a distinct number of mtDNA copies^28^, which enables them to support their specialised functions with appropriate levels of ATP^14^.

mtDNA supplementation at the time of fertilisation can increase the amount of mtDNA available to support development to a post-gastrulation stage when mtDNA replication is first initiated in the newly formed embryo. This enhancement would, for example, provide mtDNA-deficient oocytes with sufficient mtDNA to establish the ‘mtDNA set point’^27,28^ that, in turn, ensures sufficient template is available to initiate mtDNA replication, which takes place in the mouse at E6.5^29,30^. Indeed, initiation of mtDNA replication at this stage of development appears to be critical to development as mice homozygous for the mtDNA-specific replication factor (Polg^−/−^) die *in utero* due to insufficient template being available^29^. In a pig model of oocyte mtDNA deficiency, it is evident that supplementation with similar amounts of autologous populations of oocyte mtDNA can enhance embryo development rates^18^. This is mediated by the additional mtDNA inducing an mtDNA replication event between fertilisation and the 2-cell stage that increases mtDNA copy number by 4.4-fold^18^ accompanied by changes to the DNA methylation status of POLG^19^, the mtDNA-specific replication factor. Indeed, it has been shown that POLG is DNA methylated in a cell-specific manner in human^31,32^ and mouse cells^28^. Furthermore, mtDNA supplementation improves chromosomal gene expression patterns of the resultant blastocyst stage embryos whereby gene networks and pathways associated with metabolic disease and the mitochondrial regulation of epigenetic control are more or less fully restored when compared with blastocysts derived from oocytes with sufficient mtDNA copy number^18,19^. This suggests that mitochondrial supplementation influences chromosomal gene expression profiles during early development to promote improved embryo quality.

However, our data show in a mouse model that has normal reproductive function and is not hindered by poor gamete quality that the supplementation of superovulated oocytes with EPC mitochondria can alter an offspring’s phenotype and this persists over several generations. Firstly, we see increased fecundity through increased litter size, which is also reflected in primordial follicles having increased germ cell numbers and differences in gene expression profiles. However, this appears to be at the expense of increased weight gain during neonatal and juvenile development, and structural defects of the heart, which appear in at least one or more subsequent generations. Consequently, the simple addition of extra copies of mtDNA to a mature oocyte appears to influence the chromosomal genome perhaps by altering its epigenetic regulation through metabolites secreted from the mitochondria that, for example, can modulate DNA methylation. To this extent, α-ketoglutarate can mediate the transition of a DNA methylated to a DNA demethylated state by acting as a co-factor with the TET family of proteins that are largely involved in this process^33^. Indeed, in our porcine model, BCAT2, a regulator of amino acid metabolism and α-ketoglutarate^34^, was modulated as a result of mitochondrial supplementation^19^. As such, there appears to be a relationship between the differentiated state of the chromosomal genome and mtDNA copy number, which is likely established during oogenesis. During oogenesis, mtDNA copy number increases in a synchronous manner with the differentiation of early germ cells into mature metaphase II oocytes^35^. Indeed, a similar process takes place as embryonic stem cells differentiate from their naïve state into mature, functional adult cells^30^. Likewise, mtDNA depletion experiments have shown that the reduction in mtDNA copy number can diminish the differential potential of tumour-initiating cells to more of a semi-pluripotent state and they thus resume a more naïve status with reduced or delayed tumorigenic capacity^36^. Indeed, these tumours require the restoration of mtDNA copy number in order for tumorigenesis to proceed^36–38^.

Phenotypic trade-off is not unusual in breeding programs. For example, in livestock breeding, the attraction of increasing frame size and muscle mass is tempered by frequently observed declines in fertility of these enhanced animals^39,40^. In other species, such as birds, it is evident that there is a ‘trade-off’ between enhanced fertility at the expense of metabolic efficiencies^41^. Furthermore, differences between mtDNA haplotypes, which determine the common maternal origins of an individual, have been used to explain why pigs with better muscle depth and fatness to leanness ratios often have reduced fertility as indicated by poorer oocyte quality and smaller litter sizes, and vice versa^42^. In cattle, oocyte quality through decreased mtDNA copy number can also be accounted for by certain mtDNA haplotypes^10^. Similarly, cattle mtDNA can affect the phenotype of bison whereby bison carrying cattle mtDNA are smaller irrespective of whether the environment is nutritionally poor or rich^43^.

However, our studies show that simply introducing ~530 copies of mtDNA, less than 1% of an oocyte’s total mtDNA content, can induce a phenotypic trade-off, which appears to be at the expense of the structure of a key organ, namely the heart. If affected, this is likely to lead to serious health complications later in life, and, as it is a transmissible defect, it would affect subsequent generations^44^. The heart is most likely affected as it is a high ATP requiring organ that is highly dependent on oxidative phosphorylation for the production of energy through the electron transfer chain and, thus, possesses high mtDNA copy number^28^. Consequently, the potential dysregulation of mtDNA segregation or mtDNA replication during development following mitochondrial supplementation could manifest in a high-ATP requiring tissue, as is the case with many of the mtDNA disorders^45^. As a result, the strict regulation of mtDNA copy number during oogenesis^35,46^ is perturbed by mitochondrial supplementation and questions whether autologous supplementation could lead to health problems when used to improve fertility outcomes in humans and livestock species.

Whilst there has been much concern about the health and well-being of humans and mice generated from third party mitochondrial supplementation (reviewed in^14^), it appears that there is also cause for concern regarding autologous mitochondrial supplementation. In this instance, there appears to be preferential selection for four variants, which were identified in the female germline, as evidenced by their presence in the primordial follicles. One of these variants was deemed to be deleterious and, thus, harmful. However, another variant was also deemed to deleterious but it was present at a level sufficiently low enough that is unlikely to affect a phenotype^47^. Therefore, a degree of caution is required by regulators before granting licences or permission to exploit this technology clinically. This outcome is similar to third party human mitochondrial supplementation, which has been banned by regulators in a number of countries. Our data also provide a potential reason as to why many cloned offspring, which received a similar amount of mtDNA present in the donor cell as the nucleus is transferred into a recipient oocyte, exhibited poor pathologies and often died shortly after birth^48^. Furthermore, our data offer a cautionary note to proponents of nuclear transfer to treat mitochondrial disease and argue that the transfer of a small amount mitochondria with the metaphase II spindle or pronuclei, as proposed^49–51^, may have a profound effect on the offspring.

In all, we show that autologous mitochondrial supplementation can enhance reproductive function in a small cohort of founder mice by increasing litter size and the ovarian reserve in subsequent generations. However, this is at the expense of weight gain and the structure of heart tissue. Consequently, it appears that mitochondrial supplementation can induce a phenotypic trade off by modulating chromosomal gene expression profiles and changes in morphology likely by altering epigenetic patterns but also through the preferential selection of mtDNA, both of which are established during early oogenesis. However, it remains to be determined whether this effect is species-dependent and would always be specific to heart tissue or if other high-ATP requiring tissues and organs would also be affected in a random or non-random manner. Consequently, testing in a larger animal model with physiological and anatomical characteristics more similar to the human is also required.

## Methods

### Ethics approval and consent

All animal procedures and experimental protocols were approved by Monash University Animal Ethics Licensing Committee A under licence numbers MMCA 2014/43 and 2017/080 BC. All animal procedures and experimental protocols were conducted in accordance with the Australian Code for the Care and Use of Animals for Scientific Purposes, 8th Edition 2013. Mice were kept in the animal house under 12 hr light (8 am–8 pm) and 12 hr dark (8 pm–8 am).

### Superovulation and oocyte collection

Superovulation was conducted by injecting 4 to 6 week female mice (F1: C57BL6/J/MARP x CBA/MARP) with 5 IU PMSG (Folligon, MSD, Bendigo, Australia) intraperitoneally at 6 pm followed by hCG (Chorulon, MSD) intraperiotenally 48 h later. Mice were killed by cervical dislocation 15 h after hCG injection. Ampulla were isolated and kept in warm KSOM-Hepes^52^ and cumulus oocyte complexes were collected from the ampulla under a dissection microscope. Oocytes were denuded of the surrounding cumulus cells by incubating the cumulus oocyte complexes in a 100 µL drop of 100IU/mL Hyaluronidase (Sigma Aldrich, Sydney, Australia) in KSOM-Hepes for 1 min followed by pipetting using a pulled glass pipette. Only mature oocytes showing extrusion of the first polar body were used for injection. A group of 10 to 20 oocytes were kept in a drop of KSOM-culture media under oil in an incubator at 37 °C, 5% CO_2_ with humidified air until injections were performed.

### Mitochondrial preparation

EPCs were isolated from ovarian tissue of F1: C57BL6/J/MARP x CBA/MARP females using an antibody cell sorting protocol, as described in^21,53^. Mitochondria were isolated from EPCs by differential centrifugation, resuspended in buffer and condensed into a small volume by an additional centrifugation step, as described in^18^. The mitochondrial isolates were stored in aliquots in liquid nitrogen (LN_2_) until use. Mitochondrial isolates were thawed and kept at 37 °C on a heated stage in a microdroplet under mineral oil on an injection plate for injection.

### Sperm collection and preparation

A 10 week old male (F1: C57BL6/J/MARP × CBA/MARP) of proven fertility, which had produced at least one litter of 5 or more pups following its first mating, was killed by cervical dislocation and the cauda epididymis was isolated by dissection. Sperm were extracted by making a small incision in the cauda epididymis, which was then placed in 1 mL warmed KSOM-Hepes in a 5 mL tube. Sperm swam out from the epididymis into the media. The sperm suspension was transferred to a new tube. A sperm droplet for oocyte injection was made by mixing the sperm suspension with 10%PVP (1:1). A drop of 10 uL was placed in the microinjection dish (BD, Melbourne, Australia).

### ICSI and mitochondrial supplementation

A droplet of 5 uL of the mitochondrial suspension was placed in a microinjection dish next to the sperm, wash and oocyte droplets. All droplets were covered by light mineral oil (Sigma, Sydney, Australia). ICSI and mitochondrial supplementation were performed under an inverted microscope (Olympus IX71, Tokyo, Japan) equipped with an attached NK2 micromanipulator (Eppendorf AG, Eppendorf, Germany). ICSI was performed by catching a single sperm cell using a microinjection pipette (The Pipette Company, Origio, Adelaide, Australia), then injected into an oocyte that was held by a holding pipette (The Pipette Company), whilst mitochondrial supplementation was performed by catching a single sperm and then expelling it into the drop containing the mitochondria. The sperm cell was recaptured again and aspirated into the injection pipette along with ~3pl mitochondrial isolate. The sperm cell and mitochondria were then injected into the oocyte. Only surviving and non-lysed oocytes progressed to *in vitro* embryo culture.

### Embryo culture

Injected oocytes that survived were cultured in Cook-Research Embryo culture media (Cook, Brisbane, Australia) in an incubator (Heracell, Heraeus, Germany) at 37 °C, 5% CO2 in humidified air. Embryos were cultured until the 2-cell stage and then vitrified and stored for embryo transfer.

### Embryo cryopreservation and warming

2-cell stage embryos were vitrified in vitrification media containing 15% DMSO, 15% Ethylene Glycol and 0.6 M Sucrose made in KSOM-Hepes and using Fibreplugs (Cryologic, Blackburn, Australia) as the vitrification device. Vitrified embryos were kept in (LN_2_) until use. Embryos were warmed prior to embryo transfer by immersion directly in warming solution (0.5 M sucrose in KSOM-Hepes for 5 min; 0.25 M sucrose in KSOM-Hepes for 5 min) followed by transfer to embryo transfer handling media (KSOM-Hepes).

### Embryo transfer

Pseudopregnant recipients (C57BL6/J/MARP × CBA/MARP) were selected to be mated with vascetomised males based on visual appearance of being in oestrus, and only those that had a plug were used for transfer. Transfers were performed on 0.5 day post-coitus females.

Prior to surgery, 0.1 ml/10grams bodyweight of 1% of 50 mg/ml Carprofen (Norbrook Labs Australia Pty Ltd, Tullamarine, VIC, Australia) was administered. The mouse was anaesthetized with an induction volume of 5% of 1 ml/ml Isoflurane (Pharmachem, Eagle Farm, QLD, Australia) in oxygen (1–4 litres a minute) through inhalation using an AAS Stinger Anaesthetic Machine (AAS Medical, Gladesville NSW 2111, Australia) until unconscious. The vaporiser was then turned to a setting of 2.5% with an oxygen flow rate of 0.4 litre/minute (Tec 4 or 5 Vaporise). The mouse was shaved over the flanks to remove hair and the skin was swabbed with 80% Ethanol to remove any excess hair. A ~10 mm incision was made in the skin with fine dissection scissors along the dorsal midline at the level below the last rib, perpendicular to the vertebral column allowing access to both oviducts. Once the incision had been made, the connective tissue from a small area of the skin was carefully separated away from the muscle layer using scissors. A small incision (~5 mm) just over the ovary was made with fine dissection scissors and the ovary was drawn carefully through the small incision. A Serrafine clip (Fine Science Tools, North Vancouver, BC, Canada) was attached onto the fat pad and laid down over the middle of the back. The oviductal infundibulum was exposed and a hand pulled capillary containing the embryos was placed into the infundibulum until the first bend was reached. 15 to 18 embryos were transferred into the ampulla. The ovary was then placed back into the abdomen. The muscle incision was sutured using simple interrupted stitches and the skin wound was then stapled with an Autoclip Applier (Becton Dickinson, Sparks, MD, USA). 1 to 2 drops of 100 mg/20 ml Bupivicaine (Pfizer Australia Pty Ltd, West Ryde, NSW, Australia) were administered to the site. The mouse was placed on some tissues in a clean cage on a warming pad. The mouse was initially monitored every 15 min in the first hour until it was ambulatory. It was then monitored over the following 5 days, with particular attention paid to the surgical site. Post-operational analgesia, Meloxicam (2.5 ml of 1.5 mg/ml added to 280 ml of H_2_O; Boehringer Ingelheim Pty Limited Animal Health Division, West Ryde, NSW, Australia), was administered daily and orally for 3 days post-operation and the clips were removed 10 days post-operation. Once the mouse had recovered, it was returned to a quiet area in the designated transfer or quarantine room and monitored for pregnancy. Pregnant mice were allowed to give birth naturally and the offspring were maintained with the mother until weaned (3 weeks).

### Matings of controls, founders and first and second generation females

Once founder female mice and females from the first and second generations had reached sexual maturity, they were mated overnight with a proven stud (10–15 weeks old), which had, prior to use in this work, produced at least one litter of 5 or more pups following its first mating. Likewise, control female mice were mated with a proven stud that had met the same criteria. The evidence of a plug indicated that the mouse had been successfully mated. Pregnant mice gave birth naturally and the offspring were kept with the mother until weaned (3 weeks).

### Determination of mtDNA copy number per cell

DNA was isolated from oocytes and EPCs using The ARCTURUS® PicoPure® DNA Extraction Kit (Thermo Fisher Scientific, Carlsbad, CA, USA). mtDNA copy number per cell was determined, as previously described^28^. In short, targets in the nuclear genome (*ActB*) and mtDNA (n = 3) were quantified using a Rotor-Gene 3000 (Corbett Research, Cambridge, UK) under primer specific settings for *ActB* (Forward: CCCTACAGTGCTGTGGGTTT; Reverse: GAGACATGCAAGGAGTGCAA; annealing temperature 58 °C) and mtDNA (Forward: CAGTCTAATGCTTACTCAGC; Reverse: GGGCAGTTACGATAACATTG; annealing temperature 58 °C). For DNA purified from EPCs, mtDNA copy number was calculated using the formula: mtDNA copy number per cell = 2 × N_mtDNA_/N_ActB_, where N_mtDNA_ and N_ActB_ were determined by the formula: N = (qPCR product concentration × 6.023 × 10^14^)/(qPCR product size in bp × 660). The concentrations of the qPCR products were determined using the standard curves method. For DNA purified from a single oocyte, mtDNA copy number was calculated using the formula: mtDNA copy number per cell = N_mtDNA_ × Volume factor, where Volume factor was determined by the ratio of total elution volume of the DNA samples to the volume used in the qPCR reaction.

### Assessment of weights

Mice were weighed on a weekly basis from 9am to 10am on a Tuesday using the same rotation on each occasion.

### Follicle counting

The left ovary from control and mitochondrial supplemented 10 week old mice was fixed in 4% paraformaldehyde and processed at the Monash Health Translation Precinct (MHTP) Histology facility. Five micron sections were cut and five sections were mounted per slide. Light microscopy images were taken on a Olympus BX61 microscope (Olympus, Tokyo, Japan) and every fifth section was counted using CellSens Software (Olympus, Tokyo, Japan). Primordial, primary and secondary follicles were counted regardless of nuclear presence. However, antral follicles were only counted when the nucleus was visible on images. Follicles were categorized as either primordial, primordial-primary transitional, primary, primary-secondary transitional, secondary, early antral or antral.

### Isolation of primordial follicles

Day 3 ovaries were harvested and dissected into 18 to 20 pieces using 27-gauge needles. The ovary pieces were digested using Liberase DH enzyme (Roche Diagnostics Australia Pty Limited, North Ryde, NSW, Australia) at 50 ug/ml for 75 min at 37 °C with gentle agitation. The enzyme reaction was stopped using foetal calf serum (final concentration 5%). Primordial follicles were isolated using stripper tips and transferred to vials and snap frozen in liquid nitrogen and stored at −80 °C prior to RNA extraction.

### RNA extraction from primordial follicles

Total RNA was extracted from 50–100 mouse primordial follicles per ovary using The ARCTURUS® PicoPure® RNA Isolation Kit (Arcturus), according to manufacturer’s instructions. Briefly, follicles were lysed and added to pre-conditioned columns. Membrane bound RNA samples were washed, followed by an in-column DNase I (Qiagen, Hilden, Germany) treatment step to remove genomic DNA. After a series of wash steps, purified RNA samples were collected using the elution buffer and stored at −80 °C. RNA samples with high quality, as determined by the 2100 Bioanalyzer system (Agilent Technologies Inc., Santa Clara, CA, USA), were used for library preparation and sequencing, which were performed by the MHTP Medical Genomics Sequencing Facility (Clayton, VIC, Australia).

### RNA library preparation, sequencing and bioinformatics analysis

Sequencing libraries were prepared using the Trio RNA-Seq kit (NuGEN Technologies, Inc., San Carlos, CA, USA), according to the manufacturer’s protocol (M01440v1; 2017). Briefly, mitochondrial-supplemented (n = 4) and control (n = 5) RNA samples were converted to cDNA and amplified using the single-primer-isothermal-amplification (SPIA) technology (NuGEN Technologies). cDNA samples were then enzymatically fragmented to produce libraries ~320 bp in size, verified by the 2100 Bioanalyzer system (Agilent Technologies). rRNA was subsequently removed from the final libraries. An equimolar pool of each library was prepared for sequencing on the Illumina HiSeq 3000 instrument (Illumina Inc., San Diego, CA, USA) using 75 bp single-read chemistry. A total of 267 million reads passed the quality filter with a mean of 29.7 million reads amongst the samples. 96% of the reads were above the Q score of 30 with the expected PhiX spike-in error rate of 0.19%.

Raw sequence reads were processed using the ‘Trim_Galore’ package (v0.4.4; www.bioinformatics.babraham.ac.uk/projects; last accessed on 3^rd^ June, 2018) and mapped to the mouse reference genome (GRChm38/mm10) using the ‘STAR’ software package (v2.5.3a), as described in^54^. High alignment rates, determined by ‘STAR’, and low rRNA quantities, determined by the ‘SortMeRNA’ software package, as described in^55^, were observed in the samples. After initial quality checks, the ArrayQualityMetrics function from the ‘DESeq2’ software package (10.18129/B9.bioc.DESeq2; last accessed on 3^rd^ June, 2018), as described in^56^, identified one outlier from the control group, which was excluded from subsequent analyses. The ‘featureCounts’ software package, as described in^57^, was then used to summarise reads mapped to annotated exons on the reference genome. Lowly expressed genes were filtered out prior to differential testing. Differentially expressed genes were determined using ‘edgeR’ (v3.20.9) (10.18129/B9.bioc.edgeR; last accessed on 3^rd^ June, 2018). The likelihood ratio test was performed to identify differentially expressed genes between the mitochondrial supplemented (n = 4) and control (n = 4) groups. Statistical significance is defined by an adjusted p-value of <0.05 using the Benjamini-Hochberg multiple testing correction. RNA sequencing data are available at NCBI Gene Expression Omnibus under project accession number GSE114985. Gene ontology was determined with the use of the PANTHER classification system (http://www.pantherdb.org/ last accessed on 3^rd^ June, 2018).

### Next Generation Sequencing of mitochondrial genomes

Total DNA was extracted from six primordial follicle samples from third generation mice and three separate aliquots of EPC mitochondrial isolates using The ARCTURUS® PicoPure® DNA Extraction Kit (Arcturus). Whole mitochondrial genome products for sequencing were generated by amplifying two overlapping fragments (A and B) of the whole mouse mitochondrial genome using long PCR. Each reaction comprised 50 ng total DNA, 1x High Fidelity PCR buffer, 100 mM MgSO4, 1 mM dNTPs (Bioline, London, UK), 1U of Platinum Taq High Fidelity (Invitrogen, Carlsbad, CA, USA) and 10 μM each forward and reverse primer (Amplification A: Forward-CCGTGCTACCTAAACACCTTATC and Reverse-CGTCCGTACCATCATCCAATTA; Amplification B: Forward-CCCTTCATCCTTCTCTCCCTAT and Reverse GTGGGATCCCTTGAGTTACTTC). Reaction conditions were initial denaturation at 94 °C for 2 min; 34 cycles of 94 °C for 15 sec, 57 °C for 30 sec, 68 °C for 10 min; 68 °C for 10 min. PCR products were purified using the QIAquick PCR Purification Kit (Qiagen), according to the manufacturer’s protocol.

Sequencing preparation and runs were performed by the MHTP Medical Genomics Sequencing Facility. Briefly, DNA concentrations for the purified amplicons were determined using the Qubit® dsDNA HS Assay kit (Invitrogen, Eugene, OR, USA). The purified amplicon pairs (A and B) from each sample were combined at equal concentrations in order to generate libraries. Firstly, the fragments were sonicated using the S220 Focused-ultrasonicator (Covaris, Woburn, MA, USA). Sequencing libraries were prepared using the Ovation Ultralow system V2 (protocol M01380v1; NuGEN) to add adaptors. Sequencing was performed using 250 bp paired-end chemistry on the Illumina MiSeq v2 (Illumina). A PhiX spike-in (Illumina) was used to provide a technical control.

Sequences were determined as described in^19^. In short, the two FASTQ files for each sample were imported into CLC Genomics Workbench v10.0.1 (CLC bio, Aarhus, Denmark). Base pairs associated with the adaptors were removed using the ‘trimming’ tool. The reads were mapped to the mouse mitochondrial reference genome (NCBI accession: AJ489607). Reads were mapped without masking, with the built-in criteria of an insertion and deletion cost of 3 and at least 80% identity, after which duplicate reads were removed. From the mapped reads, a reference genome was generated for each sample (consensus sequence) based on the majority of reads at each base. The reads were again mapped against the consensus sequence and the low frequency variant detection tool was used to identify variants, insertions and microdeletions. A 3% minimum threshold was employed for variant calling with the variant being present on both forward and reverse reads. A minimum coverage of 2800 was also required. The ‘alignment’ tool was used to compare the consensus sequences amongst the EPC and primordial follicle samples. mtDNA sequencing data can be found at NCBI Sequence Read Archive under project accession number PRJNA474546.

### Assessment of mtDNA variants and Indels

Further analysis of the mtDNA variants and Indels, including annotation, Position-specific evolutionary preservation (PSEP), and their potential effects on function, was performed using Panther’s Evolutionary Analysis of Coding SNPs and http://mamit-tRNA.u-strasbg.fr, as described in Supplementary Table 1.

### Histopathological Assessment of Mice

Histopathological assessments of three 12-week-old female mice from each of the three mitochondrial supplemented generations and three age and sex matched control mice were carried out by the Australian Phenomics Network Histopathology Service (APNHS, Melbourne, VIC, Australia). The following organs were removed, fixed with neutral buffered formalin (NBF), and haematoxylin and eosin (H&E) stained sections were prepared: Adrenal glands, Bladder, Bone marrow, Brain, Cecum, Cervix, Clitoral gland, Colon, Duodenum, Eyes, Gall bladder, Harderian glands, Head, Heart, Hind leg (Long bone, Bone marrow, Synovial joint, Skeletal muscle), Ileum, Jejunum, Kidney, Liver, Lungs, Mammary tissue, Mesenteric lymph node, Ovaries, Oviducts, Pancreas, Salivary glands and Regional lymph nodes, Skin, Spinal cord, Spleen, Stomach, Tail, Thymus, Thyroids, Trachea, Uterus, Vagina. The mice, all organs and H&E sections of organs were examined by APNHS pathologists for macro- and micro-morphological anomalies. In addition, the hearts from four 12-month-old female 1st generation mitochondrial supplemented-derived mice were fixed, sectioned and assessed by the APNHS pathologists, as described above. Full details of analyses undertaken and the reports for each mouse are available in Supplementary File 1 with additional analysis of hearts detailed in Supplementary File 2.

## Supplementary information

Supplementary Information

## Data Availability

The RNA sequencing data are available at NCBI Gene Expression Omnibus under project accession number GSE114985. The mtDNA sequencing data can be found at NCBI Sequence Read Archive under project accession number PRJNA474546.
