# Supplementary material for: The transgenerational effects of oocyte mitochondrial supplementation
Source: Sci Rep. 2019 Apr 30;9:6694. doi: 10.1038/s41598-019-43135-4 (PMC6491721; doi:10.1038/s41598-019-43135-4)
Supplement: Supplementary file 1 — Supplementary Information [file 41598_2019_43135_MOESM1_ESM.pdf]

Supplementary Information

The transgenerational effects of oocyte mitochondrial supplementation

Authors: Justin C. St. John[1,2,*], Yogeshwar Makanji[1,2,3], Jacqueline L. Johnson[1,2], Te-Sha Tsai[1,2], Simone Lagondar[3], Fleur Rodda[4], Xin Sun[1,2], Mulyoto Pangestu[3], Penny Chen[3], Peter Temple-Smith[3]

[1] Hudson Institute of Medical Research, 27-31 Wright Street, Clayton, Vic 3168, AUSTRALIA.

[2] Department of Molecular and Translational Science, Monash University, 27-31 Wright Street, Clayton, Vic 3168, AUSTRALIA.

[3] Education Program in Reproduction and Development, Department of Obstetrics and Gynaecology, Department of Obstetrics & Gynaecology, School of Clinical Sciences, Monash University, Clayton, VIC 3168, AUSTRALIA.

[4] Monash Animal Research Platform, Monash University, Clayton, VIC 3168, AUSTRALIA.

* Corresponding author: Justin St. John, The Robinson Research Institute, The University of Adelaide, Adelaide, SA 5006, AUSTRALIA, +61 8 8313 8163, jus.stjohn@adelaide.edu.au

Supplementary Table 1: Annotation and effect of mtDNA variants and Indels identified in third generation primordial follicles and egg precursor cells.

Variant	Annotation	Substitution	PSEP	Potential effect
nt9348 (G→A)	CO3	V247I	2800	Probably damaging
nt9461 (T→C)	ND3	M1T	176	Probably benign
nt9829(InsA)	tRNA-Arg	D-loop region; Non-anticodon region		
nt9830(InsA)	tRNA-Arg	D-loop region; Non-anticodon region		
4052 (DelA)	ND2	Premature translation termination		
5172(InsA)	O_L	Regulatory region		
5172(DelA)	O_L	Regulatory region		
5172(DelAA)	O_L	Regulatory region		
9821(InsA)	tRNA-Arg	D-loop region; non-anticodon region		
9821(DelA)	tRNA-Arg	Non anticodon region		
12581(C→A)	ND5	L280I	910	Probably damaging

Annotation is based on *Mus musculus* genome assembly 10 (mm10). Protein coding substitutions were determined using Panther's Evolutionary Analysis of Coding SNPs. Position-specific evolutionary preservation (PSEP) measures the length of time (millions of years; my) that the position of a current protein has been preserved by back tracking to its direct ancestors. The greater the length of time that a position has been preserved, the greater the likelihood that it would be deleterious. The thresholds chosen were: "probably damaging" (time > 450my, which corresponds to a false positive rate of ~0.2 as tested on HumVar); "possibly damaging" (450my > time > 200my, which corresponds to a false positive rate of ~0.4); and "probably benign" (time < 200my). tRNA variants and Indels were determined based on the potential of variants to affect structure using http://mamit-tRNA.u-strasbg.fr

Supplementary Table 2: Low level variants and Indels identified in mtDNA from third generation primordial follicles (PF 1 to 6) and egg precursor cells (EPC 1 to 3).

Reference Position	Type	Reference	Allele	PF1	PF2	PF3	PF4	PF5	PF6	EPC1	EPC2	EPC3	Coding region	Mean All (± SEM)	Mean PF (± SEM)	Mean EPC (±SEM)	Significance
4052	Deletion	A	-	5.1	4.5	5.9	5	4.2	4.8	4.9	4.9	4.6	ND2	4.9 ± 1.6	4.2 ± 0.2	4.8 ± 0.1	
5172	Insertion	-	A	8.1	8.3	8.4	8.8	7.9	8.7	7.2	7.2	7.5	OL	8.0 ± 0.2	8.4 ± 0.1	7.3 ± 0.1	
5172	Deletion	A	-	22.7	22.8	21.2	22	23.6	21.1	21	21	21.2	OL	21.2 ± 0.33	22.2 ± 0.4	21.04 ± 0.1	
5172	Deletion	AA	-	4.1	3.7	3.2	3.8	3.9	3.1	4.1			OL	3.7 ± 0.15	3.6 ± 0.2	4.11 ± 0	
9821	Insertion	-	A				13.2	6.3	12.7		4.1	3.9	tRNA-Arg	8.05± 2.1	10.75 ± 2.2	4.01 ± 0.1	P<0.001
9821	Deletion	A	-							14	14	13.7	tRNA-Arg	13.9 ± 0.12		13.9 ± 0.1	P<0.0001
12581	SNV	C	A	13.9	12.5	8.6	3.8	10					ND5	9.8 ± 1.75	9.8 ± 1.75		P<0.0001

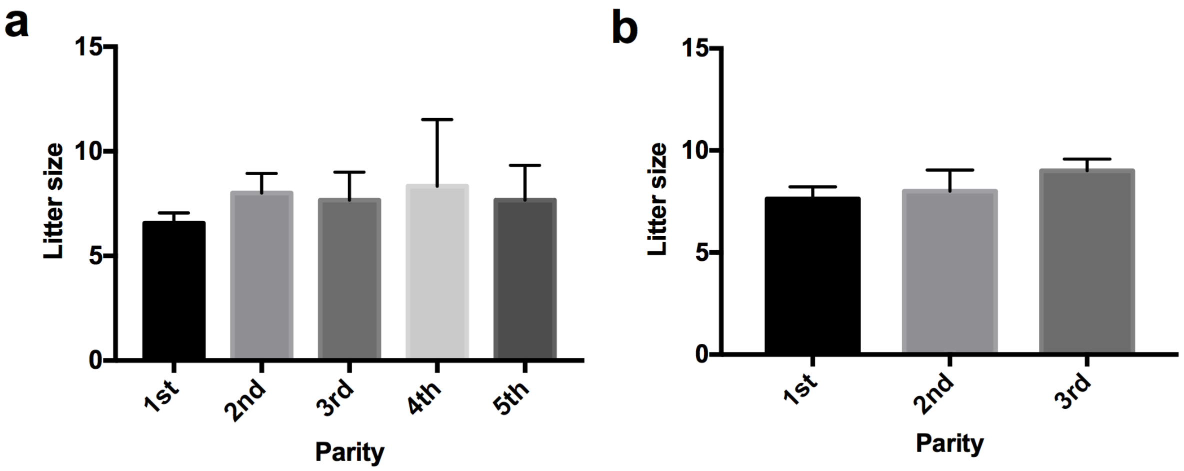

Supplementary Figure 1 The effects of mitochondrial supplementation on litter size relative to parity. (**a**) Assessment of litter size over five parities from the first generation of mitochondrial supplemented mice (n = 21). (**b**) Assessment of litter size (mean ± SEM) over three parities from the second generation of mitochondrial supplemented mice (n = 19). All values are mean ± SEM.

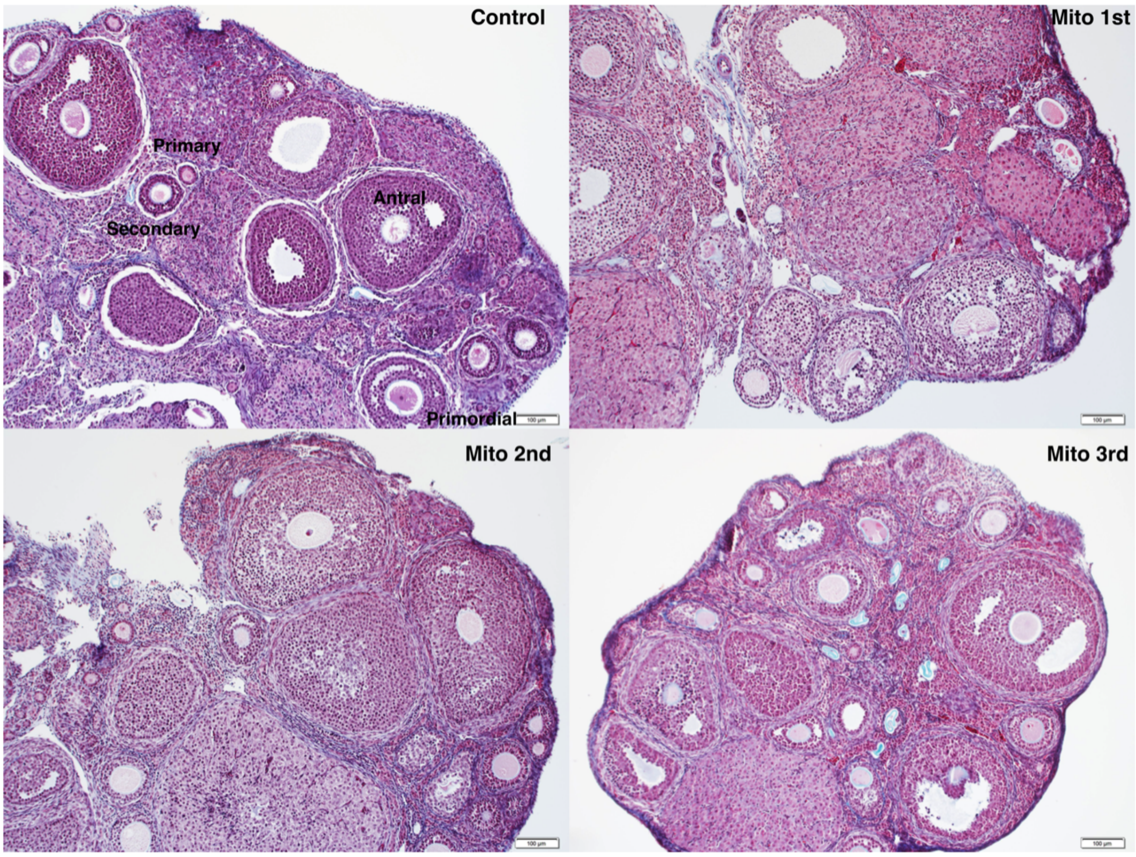

Supplementary Figure 2 Representative histological images of ovaries from ten week old control, and mitochondrial supplemented first (Mito 1st), second (Mito 2nd) and third (Mito 3rd) generation mice. Sections were stained with Masson's trichrome. Examples of primordial, primary, secondary and antral follicles are indicated on the control image. Scale bar = 100μm.

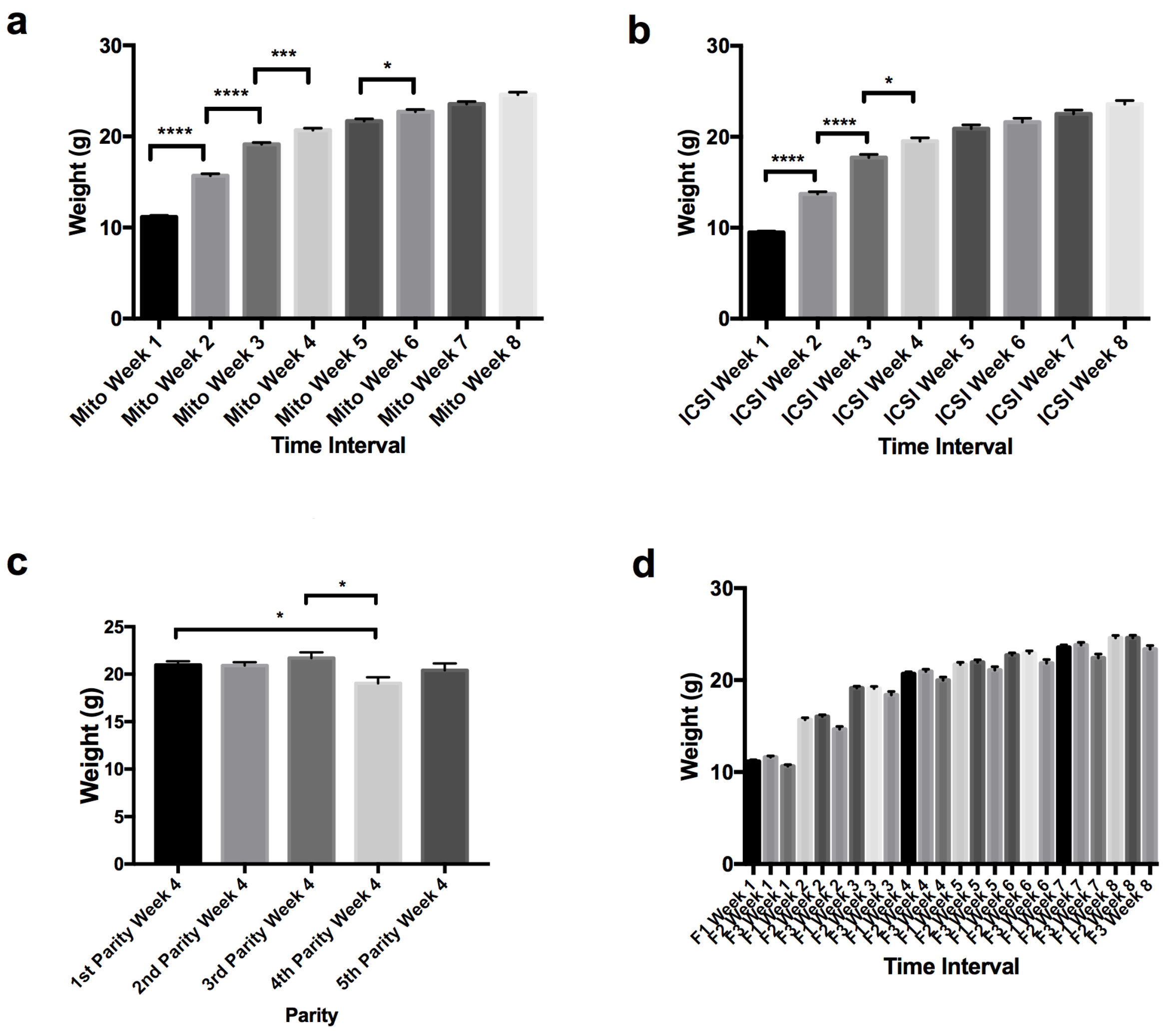

Supplementary Figure 3 Assessment of weight gain in mitochondrial supplemented mice. Mice were assessed over eight weeks (weeks 1 to 8) after birth. (**a**) Weight gain in first generation (all parities) mitochondrial supplemented mice. (**b**) Weight gain in ICSI-derived mice. (**c**) Weights at week 4 over five parities from first generation mitochondrial supplemented mice. (**d**) Total values for first (F1), second (F2) and third (F3) generation mitochondrial supplemented mice. * = P<0.05, *** = P<0.001, **** = P<0.0001. All values are mean ± SEM.

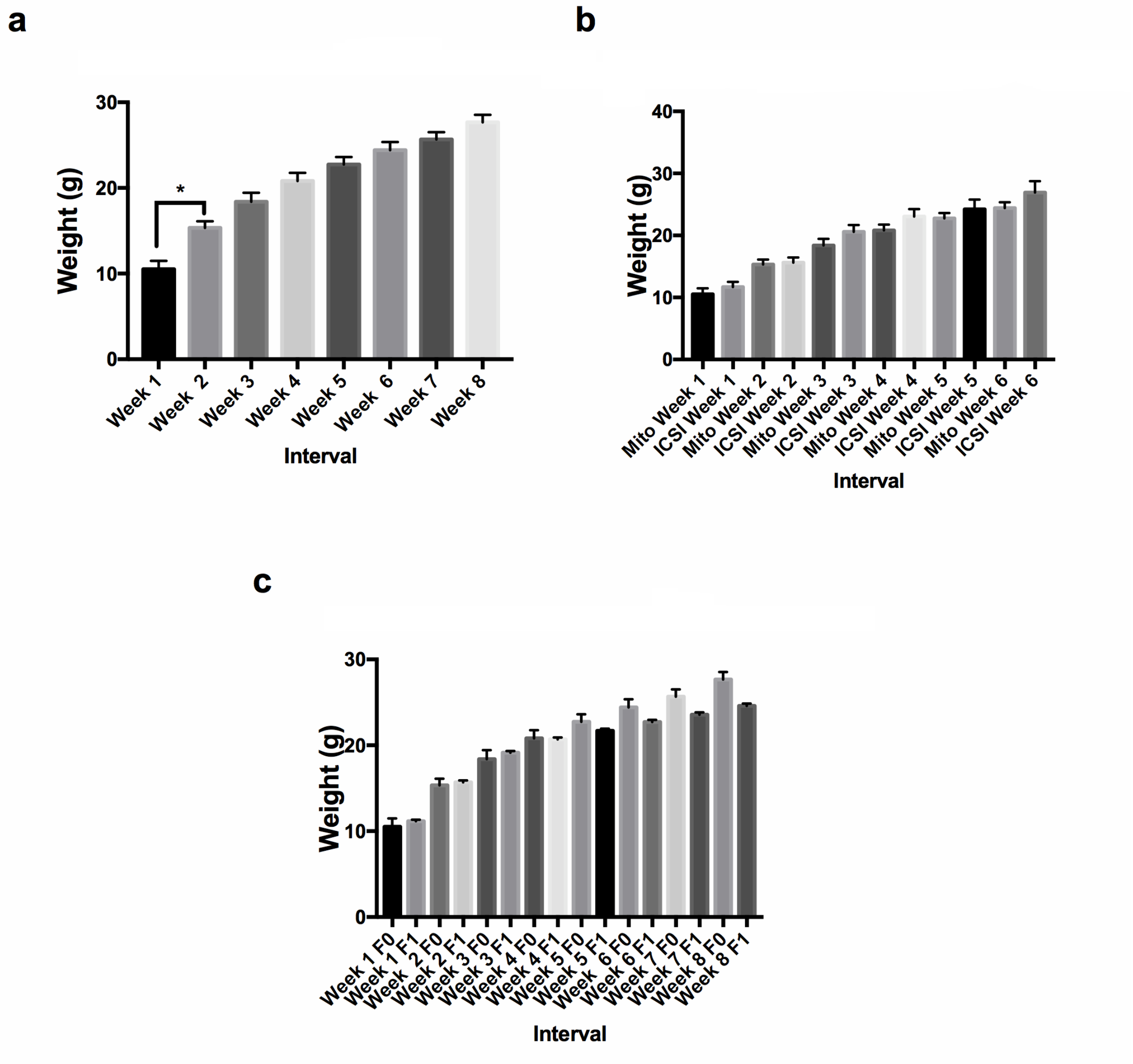

Supplementary Figure 4 Weight comparisons for founders and offspring. (**a**) Weight gain over weeks 1 to 8 for founders. (**b**) Weight gain comparisons for mitochondrial supplemented founders and ICSI-derived founders over weeks 1 to 6. (**c**) Mitochondrial supplemented founders (F0) compared with 1st (F1) mitochondrial supplemented mice. * = P<0.05. All values are mean ± SEM.

Supplementary File 1 Histopathology Report

Contacts:
Tina Cardamone, Manager APN (Melbourne)
Louise Pontell, Research Histologist
Professor Janet Keast, Head APN (Melbourne)

Australian Phenomics Network
Histopathology and Organ Pathology
The University of Melbourne
Department of Anatomy and Neuroscience
Grattan Street, PARKVILLE, VIC 3010
T: +61 3 83448044 F: +61 3 93479619
apn-info@unimelb.edu.au

9.1 Histopathology Report

Case Number APN16/016 Hudson Institute of Medical Research (Jacqui Johnson)

Registration Date Wed 23/03/2016

Animal Details

Control-1
DOB: 31/01/16, 9 weeks, Female, 21.9g, Agouti
Strain: F1 C57BL6/J x CBA

Control-2
DOB: 31/01/16, 9 weeks, Female, 20.3g, Agouti
Strain: F1 C57BL6/J x CBA

Control-3
DOB: 31/01/16, 9 weeks, Female, 17.0g, Agouti
Strain: F1 C57BL6/J x CBA

A-1 (#A137),
DOB: 12/01/16, 13 weeks, Female, 21.3g, Agouti
Strain: F1 C57BL6/J x CBA (MICSI)

A-2 (#A140),
DOB: 12/01/16, 13 weeks, Female, 20.6g, Agouti
Strain: F1 C57BL6/J x CBA (MICSI)

A-3 (#A138),
DOB: 12/01/16, 13 weeks, Female, 18.4g, Agouti
Strain: F1 C57BL6/J x CBA (MICSI)

B-1 (#B126)
DOB: 12/01/16, 13 weeks, Female, 23.7g, Agouti
Strain: F1 C57BL6/J x CBA (MICSI)

B-2 (#B133)
DOB: 12/01/16, 13 weeks, Female, 20.5g, Agouti
Strain: F1 C57BL6/J x CBA (MICSI)

B-3 (#B124)
DOB: 12/01/16, 13 weeks, Female, 24.0g, Agouti
Strain: F1 C57BL6/J x CBA (MICSI)

C-1 (#C27)
DOB: 12/01/16, 13 weeks, Female, 21.2g, Agouti
Strain: F1 C57BL6/J x CBA (MICSI)

C-2 (#C20)
DOB: 12/01/16, 13 weeks, Female, 19.9g, Agouti
Strain: F1 C57BL6/J x CBA (MICSI)

C-3 (#C29)
DOB: 12/01/16, 13 weeks, Female, 21.9g, Agouti
Strain: F1 C57BL6/J x CBA (MICSI)

**DoD / Necropsy**	Tue 05/04/2016
**Death**	CO2
**Origin**	Hudson Institute of Medical Research
**Treatment**	Treatment<br>3 x groups of adult female mice, groups designated A, B and C with 3 animals per group and 3 x matched control mice.
**Species / Breed / Strain**	F1 C57BL6/J x CBA
**Animal Health Facility**	Monash Medical Centre Animal Facility<br>POSITIVE for Pasteurella pneumotropica<br>POSITIVE for Tritrichomonas muris<br>POSITIVE for Mouse Histology<br>POSITIVE for Mouse Hepatitis Virus<br>POSITIVE for Proteus spp<br>POSITIVE for Pasteurellaceae spp (not including pneumotropica)<br>POSITIVE for Mouse Norovirus
**Organs Examined**	Adrenal glands, Bladder, Bone marrow, Brain, Cecum, Cervix, Clitoral gland, Colon, Duodenum, Eyes, Gall bladder, Harderian glands, Head, Heart, Hind leg (Long bone, Bone marrow, Synovial joint, Skeletal muscle), Ileum, Jejunum, Kidney, Liver, Lungs, Mammary tissue, Mesenteric lymph node, Other tissue, Ovaries, Oviducts, Pancreas, Salivary glands and Regional lymph nodes, Skin, Spinal cord, Spleen, Stomach, Tail, Thymus, Thyroids, Trachea, Uterus, Vagina
**Macroscopic Observations**	Animals are in good general health (J. Johnson).<br><br>Delivered to the APN Tuesday AM 5th April by Jetpets.<br><br>At the time of necropsy, the animals appeared well nourished, well groomed, active/curious and healthy with normal movement and gait. There were no observable dermal lesions and no nasal/ocular discharges. The gastrointestinal tract contained ample ingesta and the thoracic and abdominal viscera showed no macroscopic abnormalities.

Microscopic Observations

Most readings are within the normal mouse reference intervals. The Hematocrit was mildly elevated in some samples. This is likely to be a result of mild dehydration as the RBC count was normal. The platelet count for all samples was low, worth noting as some MPV values were elevated indicating large, immature platelets. We will pay close attention to the morphology of the megakaryocytes.
Please note that some low platelet counts may be due to blood collection difficulties rather than a true decrease in platelet counts. Mouse platelets readily aggregate and instrument generated platelet counts will underestimate true platelet counts in the presence of platelet clumping. In addition, mouse platelet clumps are counted as eosinophils by some automated hematology analyser. Note that the eosinophil count for all samples was elevated.

Note:
(i)Reactive lymph nodes are defined as mild follicular hyperplasia, germinal centre formation and occasional sinus histiocytosis- a common finding in mice
(ii) Mild extramedullary hematopoiesis (EMH) identified in the red pulp of all the spleens, a common finding in the mouse. EMH consists of erythroid precursors, myeloid precursors, megakaryocytes or all three. While some degree of extramedullary hematopoiesis is present in normal rodents, especially in mice, increased extramedullary hematopoiesis can result from hematotoxic insult, systemic anemia, and infections elsewhere in the body.

A.W Suttie, 2006

Summary:

A-3(#A138)-Kidney-Focal lymphocytic perivascular inflammatory aggregate identified in one kidney (54109)
B-2(#B133)-Stomach:Mild enterocyte hyperplasia, mild inflammation (neutrophils) in the lower gastric glands and submucosa (54161)
Salivary glands: Mild, multifocal, perivascular lymphocytic infiltrates(54153)
B-3(#B124)-Thymus: small cyst (54176), worth noting but not significant
C-3(#C29)- Skin: Mild, focal peri-follicular inflammation. Mild, multifocal epidermal hyperplasia with a papillated appearance (54239)

Please note:
Large, prominent and reactive lymph nodes and Peyer's patches are also a common feature in the control samples.

Request pathology comment on all kidney samples

Control-1 (control)

Macro Observations

Spleen: 10x4x2mm
Kidneys: 8x6x5mm, symmetrical
Thymus: 7x6x2mm
Heart: 8x6x5mm
Brain: 15x10x5mm, symmetrical
Pituitary gland identified, macroscopically normal
Tail 90mm (straight)
Teeth, tongue and oral mucosa unremarkable - Head harvested for evaluation of auditory and vestibular structures
Tail suspension test for neurological defects-negative
Left hind leg-Bone marrow smear

Micro Observations

Animal Control-1 was used as a histological control

Peripheral blood smear:
Examination of the smear showed red blood cells (majority of cells shown), occasional white blood cells including segmented neutrophils. No discernible morphological changes or detectable parasites (54321)

Marrow smear:
Cells observed were readily identified as those from the erythroid and myeloid series. Conspicuous cells from the lymphoid series. Numerous and unremarkable megakaryoblasts (54320).

No lesions of significance

Control-2 (control)

Macro Observations

Spleen: 14x4x2mm
Kidneys: 10x5x5mm, symmetrical
Thymus: 10x7x2mm
Heart: 8x5x5mm
Brain: 14x10x6mm, symmetrical
Pituitary gland identified, macroscopically normal
Tail 86mm (straight)

Teeth, tongue and oral mucosa unremarkable - Head harvested for evaluation of auditory and vestibular structures
Tail suspension test for neurological defects-negative
Left hind leg-Bone marrow smear

Micro Observations

Control-2 was used as a histological control

Peripheral blood smear:
Examination of the smear showed red blood cells (majority of cells shown), occasional white blood cells including segmented neutrophils and platelets (clumps). No discernible morphological changes or detectable parasites (54322)

Marrow smear:
Cells observed were readily identified as those from the erythroid and myeloid series. Conspicuous cells from the lymphoid series. Discernible and unremarkable megakaryoblasts (54323).

No lesions of significance

Control-3 (control)

Macro Observations

Spleen: 10x4x2mm
Kidneys: 8x6x5mm, symmetrical
Thymus: 7x6x2mm
Heart: 8x6x5mm
Brain: 15x10x5mm, symmetrical
Pituitary gland identified, macroscopically normal
Tail 90mm (straight)
Teeth, tongue and oral mucosa unremarkable - Head harvested for evaluation of auditory and vestibular structures
Tail suspension test for neurological defects-negative
Left hind leg-Bone marrow smear

Micro Observations

Control-3 was used as a histological control

Peripheral blood smear:
Examination of the smear showed red blood cells (majority of cells shown), occasional white blood cells including segmented neutrophils and platelets (clumps). No discernible morphological changes or detectable parasites (54324)

Marrow smear:
Cells observed were readily identified as those from the erythroid and myeloid series. Conspicuous cells from the lymphoid series. Discernible and unremarkable megakaryoblasts (54325).

No lesions of significance

A-1 (#A137)

Macro Observations

Spleen: 14x3x2mm
Kidneys: 11x6x4mm, symmetrical
Thymus: 9x5x2mm
Heart: 8x5x5mm

Brain: 14x11x7mm, symmetrical
Pituitary gland identified, macroscopically normal
Tail 82mm (straight)
Teeth, tongue and oral mucosa unremarkable - Head harvested for evaluation of auditory and vestibular structures
Tail suspension test for neurological defects-negative
Left hind leg-Bone marrow smear
Additional notes:
Excessive abdominal fat
Lungs inflated but marked congestion; judged to be terminal
Terminal blood was difficult to take

Micro Observations

Peripheral blood smear:
Examination of the smear showed red blood cells (majority of cells shown), occasional white blood cells including segmented neutrophils and platelets (clumps). No discernible morphological changes or detectable parasites (54302)

Marrow smear:
Cells observed were readily identified as those from the erythroid and myeloid series. Conspicuous cells from the lymphoid series. Discernible and unremarkable megakaryoblasts (54303).

Mammary glands

Section shows typical mammary fat pad with developing lactiferous ducts, blood vessels, nerve bundles and a large lymph node with mild follicular hyperplasia. Some scattered mast cells identified; a common finding in mouse mammary tissue.
No lesions of significance
(54065)

Ovaries/Oviducts

Section shows unremarkable ovaries containing follicles at various stages of development and several corpora lutea.
Unremarkable oviduct micromorphology with typical columnar epithelium and mucosal folds.
No lesions of significance
(54064)

Uterus/Cervix/Vagina/Clitoral gland

Unremarkable architecture of the endometrium/ endometrial glands, myometrium and adventitia. The endometrial stroma appeared distended judged to be estrus related.Some neutrophils and low numbers of lymphocytes identified in the endometrial glands; a common finding within a non-inflamed endometrium during the phases of estrus.
The micromorphology of the uterus and vagina places the animal at metestrus
No lesions of significance
(54064)

Urinary Bladder

Section does not include bladder

Liver/Gall bladder

Section shows typical liver parenchyma including hepatocytes, Kupffer cells, portal triads and central veins.
The cytoplasm of the hepatocytes appeared vacuolated indicating a non-fasted nutritional status of the animal.
Unremarkable Gall bladder

No lesions of significance
(54062)

Stomach

Section shows unremarkable fore and glandular portions of the stomach with limiting ridge.
No lesions of significance
(54081)

Small Intestine (Duodenum, Jejunum & Ileum)/GALT

Section shows typical mucosal villi and submucosal layers.
Peyer’s patches displayed typical reactive nodal histology with discernible follicles and an expanded paracortex.
No lesions of significance.
(54072, 54076)

Cecum/Colon/GALT

Typical mucosal folds and submucosal layers and occasional reactive lymphoid cluster (Peyer's patches)
No lesions of significance
(54070, 54073)

Mesenteric lymph node

Section shows mesenteric lymph node with typical reactive micromorphology including mild follicular hyperplasia, germinal centre formation, an expanded paracortex and mild sinus histiocytosis.
No lesions of significance
(54067)

Spleen

Unremarkable follicular pattern identified with typical red and white pulp micromorphology.
Mild extramedullary hematopoiesis identified in the red pulp of the spleen, a common finding in the mouse.
(54069)

Pancreas

Section shows representative exocrine tissue (serous acini) and endocrine tissue (islets of Langerhans).
No lesions of significance
(54069)

Kidney

Section shows a cortex, medulla, and papilla. There is a uniform distribution of Glomeruli and accompanying nephron components and the micromorphology of the convoluted and straight segment tubules was unremarkable.
(54063)

Comments:

Pathology to comment (medulla)

Adrenal glands

Section shows adrenal glands with typical cortex/medulla micromorphology.
No lesions of significance
(54063)

Salivary glands and Regional lymph nodes

Section shows submandibular, sublingual and parotid glands.
The lymphoid cells of the regional lymph nodes displayed mild follicular hyperplasia with germinal centre formation, indicating a reactive state.
No lesions of significance
(54067)

Thyroids

Normal lateral lobes of the thyroid gland with typical colloid secreting follicles lined by cuboidal epithelium.
No lesions of significance
(54079)

Trachea/Lungs

Section shows typical lung micromorphology demonstrating parenchyma/alveoli, bronchioles and blood vessels.
Mild degree of parenchymal congestion, judged to be artefactual.
Oesophagus with typical features including stratified squamous epithelium
No lesions of significance
(54079)

Thymus

Section shows typical medulla/cortex distribution and micromorphology.
No lesions of significance
(54078)

Heart/chambers/vessels/valves

Typical micromorphological observed in cardiac muscle, chambers, valves and vessels of the heart.
The cardiac muscle fibres demonstrated typical features including central nuclei, branching fibres and striations.
No lesions of significance
(54074, 54079)

Comments:

Pathology to comment

Skin

Typical dermal appendages and distribution. Numerous scattered mast cells identified in the subcutis and the reticular dermis; a common finding in mouse.
No lesions of significance
(54066)

Tail

Section shows typical tail components including keratinized squamous epithelium, dense regular connective tissue, tendons, bone, bone marrow, skeletal muscle, nerves and blood vessels.
No lesions of significance
(54080)

Eyes/Harderian glands

Section shows eyes with unremarkable retina, cornea, iris, ciliary body, lens, sclera and choroid.
Typical branched tubuloalveolar formation of the Harderian gland.
No lesions of significance
(54075)

Brain

Sections were prepared from the standard levels of the brain:

Level I forebrain: including cortex (Bregma 2.34mm)

Level II midbrain: including lateral and third ventricle,anterior commissure and caudate putamen (Bregma 0.38mm)

Level III hindbrain: includes the cerebellum, pons and fourth ventricle (Bregma 5.68mm)

Sections of brain stained with Haematoxylin and Eosin, Luxol Fast Blue appear symmetrical with no ventricular dilation observed, unremarkable meninges and typical lamination.
The cerebellum appears symmetrical with typical architecture and Purkinje cells.
There was no evidence of neuronal loss and the myelination appeared normal.
No lesions of significance
(54071, 54326)

Comments:

Neuropathology to comment

Spinal cord

Representative thoracic and lumbar region of spinal cord, vertebral bone, striated muscle, peripheral nerves and bone marrow.
No lesions of significance
(54068)

Comments:

Neuropathology to comment

(Hind leg) Long bone/Bone marrow/Synovial joint/Skeletal muscle

Section shows unremarkable long bone, striated muscle, synovial joint and bone marrow including conspicuous megakaryoblasts. The skeletal muscle shows consistent fiber size with peripheral nuclei.
No lesions of significance
(54077)

Head

Multiple levels through the head demonstrate dermal appendages, nasal cavity,oral cavity, teeth and tongue including muscle bundles. Sections also show unremarkable pituitary gland including pars intermedia, pars distalis and pars nervosa
as well and the trigeminal nerve/ganglia (54338).The outer and middle regions of the ear are discernible. The tympanic membrane is intact and the ossicles are unremarkable and include the stapedial annular ligaments (54340, 54342).
Typical components of the inner ear including bony labyrinth, organ of corti, stria vascularis and scala cavities are discernible. Based on multiple levels, the organ of corti is unremarkable with no discernible loss of inner/outer hair cells and typical tectorial membrane (54340, 54341, 54342).
The cochlear nerve and spiral ganglion is also demonstrated and based on several levels, there is no reduction in the density of the spiral ganglion cells. Examples of otolith organs can be seen with typical features such as the hair cells and mineral otoliths. The ampulla including the crista ridge with hair cells is discernible (54342)

No lesions of significance
(54338-54341, 55934)

A-2 (#A140)

Macro Observations

Spleen: 12x4x2mm
Kidneys: 10x6x5mm, symmetrical
Thymus: 6x5x2mm
Heart: 7x5x5mm
Brain: 15x10x5mm, symmetrical
Pituitary gland identified, macroscopically normal
Tail 80mm (straight)
Teeth, tongue and oral mucosa unremarkable - Head harvested for evaluation of auditory and vestibular structures
Tail suspension test for neurological defects-negative
Left hind leg-Bone marrow smear
Additional notes:
Terminal blood was difficult to take

Micro Observations

Peripheral blood smear:
Examination of the smear showed red blood cells (majority of cells shown), occasional white blood cells including segmented neutrophils. No obvious platelets (clumps). No discernible morphological changes or detectable parasites (54304)

Marrow smear:
Cells observed were readily identified as those from the erythroid and myeloid series. Conspicuous cells from the lymphoid series. Discernible and unremarkable megakaryoblasts (54305).

Mammary glands

Section shows typical mammary fat pad with developing lactiferous ducts, blood vessels, nerve bundles and a large lymph node with mild follicular hyperplasia. Some scattered mast cells identified; a common finding in mouse mammary tissue.
No lesions of significance
(54100)

Ovaries/Oviducts

Section shows unremarkable ovaries containing follicles at various stages of development and several corpora lutea.
Unremarkable oviduct micromorphology with typical columnar epithelium and mucosal folds.
No lesions of significance
(54101)

Uterus/Cervix/Vagina/Clitoral gland

Unremarkable architecture of the endometrium/ endometrial glands, myometrium and adventitia. Some neutrophils and low numbers of lymphocytes identified in the endometrial glands; a common finding within a non-inflamed endometrium during the phases of estrus.
No lesions of significance
(54101)

Urinary Bladder

Unremarkable collapsed bladder with typical urothelium and detrusor muscle
No lesions of significance
(54101)

Liver/Gall bladder

Section shows typical liver parenchyma including hepatocytes, Kupffer cells, portal triads and central veins.
The cytoplasm of the hepatocytes appeared vacuolated indicating a non-fasted nutritional status of the animal.
Unremarkable Gall bladder
No lesions of significance
(54099)

Stomach

Section shows unremarkable glandular portions of the stomach with limiting ridge and a portion of the non-glandular region.
No lesions of significance
(54091)

Small Intestine (Duodenum, Jejunum & Ileum)/GALT

Section shows typical mucosal villi and submucosal layers.
The Peyer's patches are prominent and displayed mild follicular hyperplasia with germinal centre formation, indicating a reactive state.
No lesions of significance
(54082, 54097)

Cecum/Colon/GALT

Typical mucosal folds and submucosal layers and occasional prominent and reactive lymphoid cluster (Peyer's patch)
No lesions of significance
(54083, 54084)

Mesenteric lymph node

Section shows small portion of mesenteric lymph node with typical nodal histology
No lesions of significance
(54086, 54082)

Spleen

Unremarkable follicular pattern identified with typical red and white pulp micromorphology.
Mild extramedullary hematopoiesis identified in the red pulp of the spleen, a common finding in the mouse.
No lesions of significance
(54088)

Pancreas

Section shows representative exocrine tissue (serous acini) and endocrine tissue (islets of Langerhans).
No lesions of significance
(54088)

Kidney

Section shows a cortex, medulla, and papilla. There is a uniform distribution of Glomeruli and accompanying nephron components and the micromorphology of the convoluted and straight segment tubules was unremarkable.
Section also includes renal lymph nodes with typical reactive nodal histology and mild sinus histiocytosis.

No lesions of significance
(54098)

Adrenal glands

Section shows adrenal glands with typical cortex/medulla micromorphology.
No lesions of significance
(54098)

Salivary glands and Regional lymph nodes

The regional lymph nodes displayed mild follicular hyperplasia with germinal centre formation and mild sinus histiocytosis, indicating a reactive state.
No lesions of significance
(54086)

Thyroids

Normal lateral lobes of the thyroid gland with typical colloid secreting follicles lined by cuboidal epithelium.
No lesions of significance
(54093)

Trachea/Lungs

Section shows typical lung micromorphology demonstrating parenchyma/alveoli, bronchioles, blood vessels and parabronchial lymph node.
Mild degree of parenchymal and vascular congestion, judged to be artefactual.
Oesophagus with typical features including stratified squamous epithelium
No lesions of significance
(54093)

Thymus

Section shows typical medulla/cortex distribution and micromorphology.
No lesions of significance
(54092)

Heart/chambers/vessels/valves

Typical micromorphological observed in cardiac muscle, chambers, valves and vessels of the heart.
The cardiac muscle fibres demonstrated typical features including central nuclei, branching fibres and striations.
No lesions of significance
(54095)

Comments:

Pathology to comment

Skin

Typical dermal appendages and distribution
No lesions of significance
(54087)

Tail

Section shows typical tail components including keratinized squamous epithelium, dense regular connective tissue, tendons, bone, bone marrow, skeletal muscle, nerves and blood vessels.
No lesions of significance
(54090)

Eyes/Harderian glands

Section shows eyes with unremarkable retina, cornea, iris, ciliary body, lens, sclera and choroid. Typical branched tubuloalveolar formation of the Harderian gland.
No lesions of significance
(54094)

Brain

Sections were prepared from the standard levels of the brain:

Level I forebrain: corpus callosum, the lateral ventricles, caudate putamen and anterior commissure (Bregma 1.18mm)

Level II midbrain: includes the hippocampus, thalamus,hypothalamus, median eminence and lateral and third ventricles (Bregma -2.18mm)

Level III hindbrain: includes the cerebellum, pons and fourth ventricle (Bregma -5.68mm)

Sections of brain stained with Haematoxylin and Eosin, Luxol Fast Blue appear symmetrical with no ventricular dilation observed, unremarkable meninges and typical lamination. The cerebellum appears symmetrical with typical architecture and Purkinje cells.
(54085, 54327)

Comments:

Neuropathology to comment

Spinal cord

Representative thoracic and lumbar region of spinal cord, brown fat (dorsal and intermuscular), vertebral bone, striated muscle, peripheral nerves and bone marrow.
(54089)

Comments:

Pathology & Neuropathology to comment

(Hind leg) Long bone/Bone marrow/Synovial joint/Skeletal muscle

Section shows unremarkable long bone, striated muscle, synovial joint and bone marrow including conspicuous megakaryoblasts. The skeletal muscle shows consistent fiber size with peripheral nuclei.
No lesions of significance
(54096)

Head

Multiple levels through the head demonstrate dermal appendages, nasal cavity,oral cavity, teeth and tongue including muscle bundles. Sections also show unremarkable pituitary gland including pars intermedia, pars distalis and pars nervosa
as well and the trigeminal nerve/ganglia (54344).The outer and middle regions of the ear are discernible. The tympanic membrane is intact and the ossicles are unremarkable and include the stapedial annular ligaments (54345, 54346, 55824).
Typical components of the inner ear including bony labyrinth, organ of corti, stria vascularis and scala cavities are discernible. Based on multiple levels, the organ of corti is unremarkable with no discernible loss of inner/outer hair cells and typical tectorial membrane (54346, 54347).
The cochlear nerve and spiral ganglion is also demonstrated and based on several levels, there is no reduction in the density of the spiral ganglion cells. Examples of otolith organs can be seen with typical features such as the hair cells and mineral otoliths (54347, 55824)

No lesions of significance
(54343-54347, 55824)

A-3 (#A138)

Macro Observations

Spleen: 9x3x3mm
Kidneys: 11x6x4mm, symmetrical
Thymus: 10x7x2mm
Heart: 8x5x5mm
Brain: 14x10x6mm, symmetrical
Pituitary gland identified, macroscopically normal
Tail 84mm (straight)
Teeth, tongue and oral mucosa unremarkable - Head harvested for evaluation of auditory and vestibular structures
Tail suspension test for neurological defects-negative
Left hind leg-Bone marrow smear
Additional notes:
Excessive abdominal fat
Lungs show marked congestion; judged to be terminal
Terminal blood was difficult to take

Micro Observations

Peripheral blood smear:
Examination of the smear showed red blood cells (majority of cells shown), occasional white blood cells including segmented neutrophils and platelets (clumps). No discernible morphological changes or detectable parasites (54306)

Marrow smear:
Cells observed were readily identified as those from the erythroid and myeloid series. Conspicuous cells from the lymphoid series. Discernible and unremarkable megakaryoblasts (54307).

Mammary glands

Section shows small portion of typical mammary fat pad with developing lactiferous ducts, blood vessels and nerve bundles.
No lesions of significance
(54107)

Ovaries/Oviducts

Section shows unremarkable ovaries containing follicles at various stages of development and several corpora lutea.
Unremarkable oviduct micromorphology with typical columnar epithelium and mucosal folds.
No lesions of significance
(54106, 55413)

Uterus/Cervix/Vagina/Clitoral gland

Unremarkable architecture of the endometrium/ endometrial glands, myometrium and adventitia. Some neutrophils and low numbers of lymphocytes identified in the endometrial glands; a common finding within a non-inflamed endometrium during the phases of estrus. The micromorphology of the uterus and vagina places the animal at metestrus
No lesions of significance
(54106, 55413)

Urinary Bladder

Unremarkable collapsed bladder with typical urothelium and detrusor muscle
No lesions of significance
(55413)

Liver/Gall bladder

Section shows typical liver parenchyma including hepatocytes, Kupffer cells, portal triads and central veins.
The cytoplasm of the hepatocytes appeared vacuolated indicating a non-fasted nutritional status of the animal.
Unremarkable Gall bladder
No lesions of significance
(54108)

Stomach

Section shows unremarkable glandular portions of the stomach with limiting ridge and a portion of the non-glandular region.
No lesions of significance
(54104)

Small Intestine (Duodenum, Jejunum & Ileum)/GALT

Section shows typical mucosal villi and submucosal layers.
The Peyer’s patches are prominent and displayed mild follicular hyperplasia with germinal centre formation, indicating a reactive state.
No lesions of significance
(54115, 54119)

Cecum/Colon/GALT

Typical mucosal folds and submucosal layers and occasional, prominent and reactive lymphoid clusters (Peyer's patch)
No lesions of significance
(54114, 54117)

Mesenteric lymph node

Section shows mesenteric lymph node with typical reactive micromorphology including mild follicular hyperplasia, germinal centre formation and mild sinus histiocytosis.
No lesions of significance
(54113)

Spleen

Unremarkable follicular pattern identified with typical red and white pulp micromorphology.
Mild extramedullary hematopoiesis identified in the red pulp of the spleen, a common finding in the mouse.
No lesions of significance
(54111)

Pancreas

Section shows representative exocrine tissue (serous acini) and endocrine tissue (islets of Langerhans).
No lesions of significance
(54111)

Kidney

Section shows a cortex, medulla, and papilla. There is a uniform distribution of Glomeruli and accompanying nephron components and the micromorphology of the convoluted and straight segment tubules was unremarkable.
Focal lymphocytic perivascular inflammatory aggregate identified in one kidney.
(54109)

Comments:

Pathology to comment

Adrenal glands

Section shows adrenal glands with typical cortex/medulla micromorphology.
No lesions of significance
(54109)

Salivary glands and Regional lymph nodes

Section shows submandibular, sublingual and parotid glands.
The regional lymph nodes with typical reactive nodal histology.
No lesions of significance
(54113)

Thyroids

Normal lateral lobes of the thyroid gland with typical colloid secreting follicles lined by cuboidal epithelium.
No lesions of significance
(54102)

Trachea/Lungs

Section shows typical lung micromorphology demonstrating parenchyma/alveoli, bronchioles, blood vessels and parabronchial lymph node.
Mild degree of parenchymal and vascular congestion, judged to be artefactual.
Trachea with unremarkable mucosal epithelial lining and hyaline cartilage.
Oesophagus with typical features including stratified squamous epithelium
No lesions of significance
(54093)

Thymus

Section shows typical medulla/cortex distribution and micromorphology.
No lesions of significance
(54103)

Heart/chambers/vessels/valves

Typical micromorphological observed in cardiac muscle, chambers, valves and vessels of the heart.
The cardiac muscle fibres demonstrated typical features including central nuclei, branching fibres and striations.
No lesions of significance
(54121)

Comments:

Pathology to comment

Skin

Typical dermal appendages and distribution
No lesions of significance
(54112)

Tail

Section shows typical tail components including keratinized squamous epithelium, dense regular connective tissue, tendons, bone, bone marrow, skeletal muscle, nerves and blood vessels.
No lesions of significance
(54105)

Eyes/Harderian glands

Section shows eyes with unremarkable retina, cornea, iris, ciliary body, lens, sclera and choroid.
Typical branched tubuloalveolar formation of the Harderian gland.
No lesions of significance
(54120)

Brain

Sections were prepared from the standard levels of the brain:

Level I forebrain: corpus callosum, the third and lateral ventricles and caudate putamen (Bregma 0.14mm)

Level II midbrain: includes the hippocampus, thalamus,hypothalamus, median eminence and lateral and third ventricles (Bregma -1.94mm)

Level III hindbrain: includes the cerebellum, pons and fourth ventricle (Bregma -5.68mm)

Sections of brain stained with Haematoxylin and Eosin, Luxol Fast Blue appear symmetrical with no ventricular dilation observed, unremarkable meninges and typical lamination. The cerebellum appears symmetrical with typical architecture and Purkinje cells.
(54116,54328)

Comments:

Neuropathology to comment

Spinal cord

Representative thoracic and lumbar region of spinal cord, vertebral bone, striated muscle, peripheral nerves and bone marrow.
No lesions of significance
(54110)

Comments:

Neuropathology to comment

(Hind leg) Long bone/Bone marrow/Synovial joint/Skeletal muscle

Section shows unremarkable long bone, striated muscle, synovial joint, ligaments and bone marrow
including conspicuous megakaryoblasts. The skeletal muscle shows consistent fiber size with peripheral nuclei
No lesions of significance
(54118)

Head

Multiple levels through the head demonstrate dermal appendages, nasal cavity,oral cavity, teeth and tongue including muscle bundles. Sections also show unremarkable pituitary gland including pars intermedia, pars distalis and pars nervosa
as well and the trigeminal nerve/ganglia (54349). Levels also include small portion of thyroid glands (54350).
The outer and middle regions of the ear are discernible. The tympanic membrane is intact and the ossicles are unremarkable and include the stapedial annular ligaments (54352, 55828, 55830).
Typical components of the inner ear including bony labyrinth, organ of corti, stria vascularis and scala cavities are discernible. Based on multiple levels, the organ of corti is unremarkable with no discernible loss of inner/outer hair cells and typical tectorial membrane (54352, 55828).
The cochlear nerve and spiral ganglion is also demonstrated and based on several levels, there is no reduction in the density of the spiral ganglion cells. Examples of otolith organs can be seen with typical features such as the hair cells and mineral otoliths. The ampulla including the crista ridge with hair cells is discernible (55830)

No lesions of significance
(54348-54352, 55828,55830)

B-1 (#B126)

Macro Observations

Spleen: 12x4x2mm
Kidneys: 10x6x5mm, symmetrical
Thymus: 6x5x2mm
Heart: 8x6x5mm
Brain: 14x10x5mm, symmetrical
Pituitary gland identified, macroscopically normal
Tail 90mm (straight)
Teeth, tongue and oral mucosa unremarkable - Head harvested for evaluation of auditory and vestibular structures
Tail suspension test for neurological defects-negative
Left hind leg-Bone marrow smear
Additional notes:
Excessive abdominal fat and subcutaneous dorsal fat
Lungs show marked congestion; judged to be terminal
Terminal blood was difficult to take

Micro Observations

Peripheral blood smear:
Examination of the smear showed red blood cells (majority of cells shown), occasional white blood cells including segmented neutrophils and platelets (clumps). No discernible morphological changes or detectable parasites (54768)

Marrow smear:
Cells observed were readily identified as those from the erythroid and myeloid series. Conspicuous cells from the lymphoid series. Discernible and unremarkable megakaryoblasts (54309).

Mammary glands

Section shows typical mammary fat pad with developing lactiferous ducts, blood vessels, dermal appendages and nerve bundles.
No lesions of significance
(54127)

Ovaries/Oviducts

Section shows unremarkable ovaries containing numerous follicles at various stages of development and several corpora lutea.
No lesions of significance
(54129)

Uterus/Cervix/Vagina/Clitoral gland

Unremarkable architecture of the endometrium/ endometrial glands, myometrium and adventitia. Some neutrophils and low numbers of lymphocytes identified in the endometrial glands; a common finding within a non-inflamed endometrium during the phases of estrus. The micromorphology of the uterus and vagina places the animal at Diestrus.
No lesions of significance
(54129)

Urinary Bladder

Unremarkable collapsed bladder with typical urothelium and detrusor muscle.
No lesions of significance
(54129)

Liver/Gall bladder

Section shows typical liver parenchyma including hepatocytes, Kupffer cells, portal triads and central veins. The cytoplasm of the hepatocytes appeared vacuolated indicating a non-fasted nutritional status of the animal. Section also shows unremarkable regional lymph node.
Unremarkable Gall bladder
No lesions of significance
(54133)

Stomach

Section shows unremarkable glandular portions of the stomach with limiting ridge and a portion of the non-glandular region.
No lesions of significance
(54140)

Small Intestine (Duodenum, Jejunum & Ileum)/GALT

Section shows typical mucosal villi and submucosal layers.
The Peyer's patches are prominent and displayed mild follicular hyperplasia with germinal centre formation, indicating a reactive state.
No lesions of significance
(54123, 54137)

Cecum/Colon/GALT

Typical mucosal folds and submucosal layers and occasional, prominent and reactive lymphoid clusters (Peyer's patch)
No lesions of significance
(54122, 54125)

Mesenteric lymph node

Section shows mesenteric lymph node with typical reactive micromorphology including mild follicular hyperplasia, germinal centre formation and mild sinus histiocytosis.
No lesions of significance
(54130)

Spleen

Unremarkable follicular pattern identified with typical red and white pulp micromorphology.
Mild extramedullary hematopoiesis identified in the red pulp of the spleen, a common finding in the mouse.
No lesions of significance
(54126)

Pancreas

Section shows representative exocrine tissue (serous acini) and endocrine tissue (islets of Langerhans).
No lesions of significance
(54126)

Kidney

Section shows a cortex, medulla, and papilla. There is a uniform distribution of Glomeruli and accompanying nephron components and the micromorphology of the convoluted and straight segment tubules was unremarkable.
(54131)

Comments:

Pathology to comment (medulla)

Adrenal glands

Section shows one adrenal gland with typical cortex/medulla micromorphology.
No lesions of significance
(54131)

Salivary glands and Regional lymph nodes

Section shows submandibular, sublingual and parotid glands.
The regional lymph nodes displayed mild follicular hyperplasia with germinal centre formation, indicating a reactive state.
No lesions of significance
(54130)

Thyroids

Normal lateral lobes of the thyroid gland with typical colloid secreting follicles lined by cuboidal epithelium.
No lesions of significance
(54136)

Trachea/Lungs

Section shows typical lung micromorphology demonstrating parenchyma/alveoli, bronchioles and blood vessels.
Mild degree of parenchymal and vascular congestion, judged to be artefactual.
Trachea with unremarkable mucosal epithelial lining and hyaline cartilage
Oesophagus with typical features including stratified squamous epithelium
No lesions of significance
(54136)

Thymus

Section shows typical medulla/cortex distribution and micromorphology.
No lesions of significance
(54134)

Heart/chambers/vessels/valves

Typical micromorphological observed in cardiac muscle, chambers, valves and vessels of the heart.
The cardiac muscle fibres demonstrated typical features including central nuclei, branching fibres and striations.
No lesions of significance
(54141)

Skin

Typical dermal appendages and distribution. Unremarkable thin layer of striated muscle (panniculus carnosus)
No lesions of significance
(54132)

Tail

Section shows typical tail components including keratinized squamous epithelium, dense regular connective tissue, tendons, bone, bone marrow, skeletal muscle, nerves and blood vessels.
No lesions of significance
(54138)

Eyes/Harderian glands

Section shows eyes with unremarkable retina, cornea, iris, ciliary body, lens, sclera and choroid.
Typical branched tubuloalveolar formation of the Harderian gland.
No lesions of significance
(54139)

Brain

Sections were prepared from the standard levels of the brain:

Level I forebrain: corpus callosum, lateral ventricles and caudate putamen (Bregma 0.38mm)

Level II midbrain: includes the hippocampus, thalamus,hypothalamus, median eminence, lateral and third ventricles (Bregma -1.82mm)

Level III hindbrain: includes the cerebellum, pons and fourth ventricle (Bregma -6.12mm)

Sections of brain stained with Haematoxylin and Eosin, Luxol Fast Blue appear symmetrical with no ventricular dilation observed, unremarkable meninges and typical lamination. The cerebellum appears symmetrical with typical architecture and Purkinje cells.
(54124, 54329)

Comments:

Neuropathology to comment

Spinal cord

Representative thoracic and lumbar region of spinal cord, vertebral bone, striated muscle, peripheral nerves and bone marrow.
No lesions of significance
(54128)

Comments:

Neuropathology to comment

(Hind leg) Long bone/Bone marrow/Synovial joint/Skeletal muscle

Section shows unremarkable long bone, striated muscle, synovial joint, ligaments and bone marrow
including conspicuous megakaryoblasts. The skeletal muscle shows consistent fiber size with peripheral nuclei
No lesions of significance
(54135)

Head

Multiple levels through the head demonstrate dermal appendages, nasal cavity,oral cavity, teeth and tongue including muscle bundles. Sections also show unremarkable pituitary gland including pars intermedia, pars distalis and pars nervosa
as well and the trigeminal nerve/ganglia (54353, 54354).The outer and middle regions of the ear are discernible. The tympanic membrane is intact and the ossicles are unremarkable and include the stapedial annular ligaments (54355, 54357, 55829).
Typical components of the inner ear including bony labyrinth, organ of corti, stria vascularis and scala cavities are discernible. Based on multiple levels, the organ of corti is unremarkable with no discernible loss of inner/outer hair cells and typical tectorial membrane (54355, 54357).
The cochlear nerve and spiral ganglion is also demonstrated and based on several levels, there is no reduction in the density of the spiral ganglion cells. Examples of otolith organs can be seen with typical features such as the hair cells and mineral otoliths. The ampulla including the crista ridge with hair cells is discernible (54357, 55829)

No lesions of significance
(54353-54357, 55829)

B-2 (#B133)

Macro Observations

Spleen: 14x4x3mm
Kidneys: 11x5x4mm, symmetrical
Thymus: 12x6x2mm
Heart: 8x6x5mm
Brain: 15x10x6mm, symmetrical
Pituitary gland identified, macroscopically normal
Tail 82mm (straight)
Teeth, tongue and oral mucosa unremarkable - Head harvested for evaluation of auditory and vestibular structures
Tail suspension test for neurological defects-negative
Left hind leg-Bone marrow smear
Additional notes:
Excessive abdominal fat
Excessive fat surrounding salivary glands and ovaries
Lungs show marked congestion; judged to be terminal
Terminal blood was difficult to take

Micro Observations

Peripheral blood smear:
Examination of the smear showed red blood cells (majority of cells shown), occasional white blood cells including segmented neutrophils and platelets (clumps). No discernible morphological changes or detectable parasites (54767)

Marrow smear:
Cells observed were readily identified as those from the erythroid and myeloid series.

Conspicuous cells from the lymphoid series. Discernible and unremarkable megakaryoblasts (54311).

Mammary glands

Section shows typical mammary fat pad with developing lactiferous ducts, blood vessels, nerve bundles and a large lymph node with mild follicular hyperplasia.
No lesions of significance
(54155)

Ovaries/Oviducts

Section shows unremarkable ovaries containing follicles at various stages of development and several corpora lutea.
Unremarkable oviduct micromorphology with typical columnar epithelium and mucosal folds.
No lesions of significance
(54154)

Uterus/Cervix/Vagina/Clitoral gland

Unremarkable architecture of the endometrium/ endometrial glands, myometrium and adventitia.Some neutrophils and low numbers of lymphocytes identified in the endometrial glands; a common finding within a non-inflamed endometrium during the phases of estrus.
The micromorphology of the uterus and vagina places the animal at diestrus.
No lesions of significance

Urinary Bladder

Unremarkable collapsed bladder with typical urothelium and detrusor muscle
No lesions of significance
(54154)

Liver/Gall bladder

Section shows typical liver parenchyma including hepatocytes, Kupffer cells, portal triads and central veins. The cytoplasm of the hepatocytes appeared vacuolated indicating a non-fasted nutritional status of the animal.
Unremarkable Gall bladder
No lesions of significance
(54156)

Stomach

Section shows unremarkable glandular portions of the stomach with limiting ridge and a portion of the non-glandular region.
In the glandular portion, mild enterocyte hyperplasia, mild inflammation (neutrophils) in the lower gastric glands and submucosa.
(54161)

Comments:

Pathology to comment

Small Intestine (Duodenum, Jejunum & Ileum)/GALT

Section shows typical mucosal villi and submucosal layers.
The Peyer’s patches are large, prominent and displayed mild follicular hyperplasia with germinal centre formation, indicating a reactive state.
No lesions of significance
(54142, 54146)

Cecum/Colon/GALT

Typical mucosal folds and submucosal layers and occasional, prominent and reactive lymphoid clusters (Peyer's patch)
No lesions of significance
(54147, 54148)

Mesenteric lymph node

Section shows mesenteric lymph node with typical reactive micromorphology including mild follicular hyperplasia, germinal centre formation and mild sinus histiocytosis.
No lesions of significance
(54153)

Spleen

Unremarkable follicular pattern identified with typical red and white pulp micromorphology.
Mild extramedullary hematopoiesis identified in the red pulp of the spleen, a common finding in the mouse.
No lesions of significance
(54151)

Pancreas

Section shows representative exocrine tissue (serous acini) and endocrine tissue (islets of Langerhans).
No lesions of significance
(54151)

Kidney

Section shows a cortex, medulla, and papilla. There is a uniform distribution of Glomeruli and accompanying nephron components and the micromorphology of the convoluted and straight segment tubules was unremarkable.
No lesions of significance
(54157)

Adrenal glands

Section shows one adrenal gland with typical cortex/medulla micromorphology.
No lesions of significance
(54157)

Salivary glands and Regional lymph nodes

Section shows submandibular, sublingual and parotid glands. Mild, multifocal, perivascular lymphocytic infiltrates in the submandibular glands, a common background lesion in the mouse
The regional lymph nodes displayed mild follicular hyperplasia with germinal centre formation, indicating a reactive state.
(54153)

Comments:

Note: Perivascular lymphocytic infiltrates in the submandibular and parotid glands increase in incidence and severity with age (M.R. Anver and D.C Haines, The Laboratory Mouse)
Pathology to comment

Thyroids

Unremarkable colloid secreting follicles identified.
No lesions of significance
(54159)

Trachea/Lungs

Section shows typical lung micromorphology demonstrating parenchyma/alveoli, bronchioles and blood vessels.
Mild degree of parenchymal and vascular congestion, judged to be artefactual.
Oesophagus with typical features including stratified squamous epithelium
No lesions of significance
(54159)

Thymus

Section shows typical medulla/cortex distribution and micromorphology.
No lesions of significance
(54158)

Heart/chambers/vessels/valves

Typical micromorphological observed in cardiac muscle, chambers, valves and vessels of the heart.
The cardiac muscle fibres demonstrated typical features including central nuclei, branching fibres and striations.
No lesions of significance
(54144)

Skin

Typical dermal appendages and distribution. Unremarkable thin layer of striated muscle (panniculus carnosus).
No lesions of significance
(54152)

Tail

Section shows typical tail components including keratinized squamous epithelium, dense regular connective tissue, tendons, bone, bone marrow, skeletal muscle, nerves and blood vessels.
No lesions of significance
(54160)

Eyes/Harderian glands

Section shows eyes with unremarkable retina, cornea, iris, ciliary body, lens, sclera and choroid.
Typical branched tubuloalveolar formation of the Harderian gland.
No lesions of significance
(54145)

Brain

Sections were prepared from the standard levels of the brain:

Level I forebrain: corpus callosum, lateral ventricles and caudate putamen (Bregma 0.62mm)

Level II midbrain: includes the hippocampus, thalamus,hypothalamus, lateral and third ventricles (Bregma -1.58mm)

Level III hindbrain: includes the cerebellum, pons and fourth ventricle (Bregma -5.68mm)

Sections of brain stained with Haematoxylin and Eosin, Luxol Fast Blue appear symmetrical with

no ventricular dilation observed, unremarkable meninges and typical lamination. The cerebellum appears symmetrical with typical architecture and Purkinje cells.
(54149, 54330)

Comments:

Neuropathology to comment

Spinal cord

Representative thoracic and lumbar region of spinal cord, vertebral bone, striated muscle, peripheral nerves and bone marrow.
No lesions of significance
(54150)

Comments:

Neuropathology to comment

(Hind leg) Long bone/Bone marrow/Synovial joint/Skeletal muscle

Section shows unremarkable long bone, striated muscle, synovial joint, ligaments and bone marrow
including conspicuous megakaryoblasts. The skeletal muscle shows consistent fiber size with peripheral nuclei
No lesions of significance
(54143)

Head

Multiple levels through the head demonstrate dermal appendages, nasal cavity,oral cavity, teeth and tongue including muscle bundles. Sections also show unremarkable pituitary gland including pars intermedia, pars distalis and pars nervosa
as well and the trigeminal nerve/ganglia (54359).The outer and middle regions of the ear are discernible. The tympanic membrane is intact and the ossicles are unremarkable and include the stapedial annular ligaments (55826, 54360, 54361, 54362, 55826).
Typical components of the inner ear including bony labyrinth, organ of corti, stria vascularis and scala cavities are discernible. Based on multiple levels, the organ of corti is unremarkable with no discernible loss of inner/outer hair cells and typical tectorial membrane (54361, 55827).
The cochlear nerve and spiral ganglion is also demonstrated and based on several levels, there is no reduction in the density of the spiral ganglion cells. Examples of otolith organs can be seen with typical features such as the hair cells and mineral otoliths. The ampulla including the crista ridge with hair cells is discernible (54361)

No lesions of significance
(54358-54362, 55826-55827)

B-3 (#B124)

Macro Observations

Spleen: 12x5x2mm
Kidneys: 10x6x5mm, symmetrical
Thymus: 6x6x2 mm
Heart: 8x6x5mm
Brain: 15x10x5mm, symmetrical
Pituitary gland identified, macroscopically normal
Tail 82mm (straight)
Teeth, tongue and oral mucosa unremarkable - Head harvested for evaluation of auditory and vestibular structures
Tail suspension test for neurological defects-negative
Left hind leg-Bone marrow smear

Additional notes:
Excessive abdominal fat
Excessive fat surrounding salivary glands and ovaries
Lungs show marked congestion; judged to be terminal
Terminal blood was difficult to take

Micro Observations

Peripheral blood smear:
Examination of the smear showed red blood cells (majority of cells shown), occasional white blood cells including segmented neutrophils and platelets (clumps). No discernible morphological changes or detectable parasites (54769)

Marrow smear:
Cells observed were readily identified as those from the erythroid and myeloid series. Conspicuous cells from the lymphoid series. Discernible and unremarkable megakaryoblasts (54313).

Mammary glands

Section shows typical mammary fat pad with developing lactiferous ducts, blood vessels, nerve bundles and a large lymph node with mild follicular hyperplasia.
No lesions of significance
(54168, 56045)

Ovaries/Oviducts

Section shows unremarkable ovaries containing follicles at various stages of development and several corpora lutea.
Unremarkable oviduct micromorphology with typical columnar epithelium and mucosal folds.
No lesions of significance
(54169)

Uterus/Cervix/Vagina/Clitoral gland

Unremarkable architecture of the endometrium/ endometrial glands, myometrium and adventitia.Some neutrophils and low numbers of lymphocytes identified in the endometrial glands; a common finding within a non-inflamed endometrium during the phases of estrus.
The micromorphology of the uterus and vagina places the animal at diestrus.
No lesions of significance
(54169)

Urinary Bladder

Unremarkable collapsed bladder with typical urothelium and detrusor muscle
No lesions of significance
(54169)

Liver/Gall bladder

Section shows typical liver parenchyma including hepatocytes, Kupffer cells, portal triads and central veins. The cytoplasm of the hepatocytes appeared vacuolated indicating a non-fasted nutritional status of the animal.
Unremarkable Gall bladder
No lesions of significance
(54167)

Stomach

Section shows unremarkable fore and glandular portions of the stomach with limiting ridge.
No lesions of significance
(54175)

Small Intestine (Duodenum, Jejunum & Ileum)/GALT

Section shows typical mucosal villi and submucosal layers.
The Peyer’s patches are prominent and displayed mild follicular hyperplasia with germinal centre formation, indicating a reactive state.
No lesions of significance
(54164, 54173)

Cecum/Colon/GALT

Typical mucosal folds and submucosal layers and occasional, prominent and reactive lymphoid clusters (Peyer's patch)
No lesions of significance
(54162, 54165)

Mesenteric lymph node

Section shows mesenteric lymph node with typical reactive micromorphology including mild follicular hyperplasia, germinal centre formation and mild sinus histiocytosis.
No lesions of significance
(54178, 54165)

Spleen

Unremarkable follicular pattern identified with typical red and white pulp micromorphology.
Mild extramedullary hematopoiesis identified in the red pulp of the spleen, a common finding in the mouse.
No lesions of significance
(54180)

Pancreas

Section shows representative exocrine tissue (serous acini) and endocrine tissue (islets of Langerhans).
No lesions of significance
(54180)

Kidney

Section shows a cortex, medulla, and papilla. There is a uniform distribution of Glomeruli and accompanying nephron components and the micromorphology of the convoluted and straight segment tubules was unremarkable.
(54166)

Comments:

Pathology to comment (medulla)

Adrenal glands

Section shows adrenal glands with typical cortex/medulla micromorphology.
No lesions of significance
(54166)

Salivary glands and Regional lymph nodes

Section shows submandibular, sublingual and parotid glands.
The regional lymph nodes displayed mild follicular hyperplasia with germinal centre formation, indicating a reactive state.
No lesions of significance

(54178)

Thyroids

Unremarkable colloid secreting follicles identified. Section also shows a small sheet-like mass of polygonal cells, characteristic of the parathyroid gland.
No lesions of significance
(54177)

Trachea/Lungs

Section shows typical lung micromorphology demonstrating parenchyma/alveoli, bronchioles and blood vessels.
Mild degree of parenchymal and vascular congestion, judged to be artefactual.
Oesophagus with typical features including stratified squamous epithelium.
No lesions of significance
(54177)

Thymus

Section shows typical medulla/cortex distribution and micromorphology.
One thymic cyst identified; worth noting but not significant
(54176)

Comments:

Thymic cysts represent either a dilation of thymic tubular structures or remnants of the thymopharyngeal ducts. Cysts are a common finding in aged mice or they may be associated with epithelial glandular hyperplasia.
Pathology of the Mouse, R.R Maronpot
Pathology to comment

Heart/chambers/vessels/valves

Typical micromorphological observed in cardiac muscle, chambers, valves and vessels of the heart.
The cardiac muscle fibres demonstrated typical features including central nuclei, branching fibres and striations.
No lesions of significance
(54171)

Skin

Typical dermal appendages and distribution. Unremarkable thin layer of striated muscle (panniculus carnosus)
No lesions of significance
(54179)

Tail

Section shows typical tail components including keratinized squamous epithelium, dense regular connective tissue, tendons, bone, bone marrow, skeletal muscle, nerves and blood vessels.
No lesions of significance
(54174)

Eyes/Harderian glands

Section shows eyes with unremarkable retina, cornea, iris, ciliary body, lens, sclera and choroid. Section also includes portion of optic nerve. Typical branched tubuloalveolar formation of the Harderian gland.
No lesions of significance
(54170)

Brain

Sections were prepared from the standard levels of the brain:

Level I forebrain: corpus callosum, lateral ventricles and caudate putamen (Bregma 1.10mm)

Level II midbrain: includes the hippocampus, thalamus,hypothalamus, lateral and third ventricles (Bregma -1.70mm)

Level III hindbrain: includes the cerebellum, pons and fourth ventricle (Bregma -5.52mm)

Sections of brain stained with Haematoxylin and Eosin, Luxol Fast Blue appear symmetrical with no ventricular dilation observed, unremarkable meninges and typical lamination. The cerebellum appears symmetrical with typical architecture and Purkinje cells.
(54163, 54331)

Comments:

Neuropathology to comment

Spinal cord

Representative thoracic and lumbar region of spinal cord, vertebral bone, striated muscle, peripheral nerves and bone marrow.
No lesions of significance
(54181)

Comments:

Neuropathology to comment

(Hind leg) Long bone/Bone marrow/Synovial joint/Skeletal muscle

Section shows unremarkable long bone, striated muscle, synovial joint, ligaments and bone marrow
including conspicuous megakaryoblasts. The skeletal muscle shows consistent fiber size with peripheral nuclei
No lesions of significance
(54172)

Head

Multiple levels through the head demonstrate dermal appendages, nasal cavity,oral cavity, teeth and tongue including muscle bundles. Sections also show unremarkable pituitary gland including pars intermedia, pars distalis and pars nervosa
as well and the trigeminal nerve/ganglia (54364).The outer and middle regions of the ear are discernible. The tympanic membrane is intact and the ossicles are unremarkable and include the stapedial annular ligaments (54365,54366).
Typical components of the inner ear including bony labyrinth, organ of corti, stria vascularis and scala cavities are discernible. The organ of corti is discernible however there is marked mechanical artefact (54366).
Examples of otolith organs can be seen with typical features such as the hair cells and mineral otoliths. The ampulla including the crista ridge with hair cells is discernible (54366)

No lesions of significance
(54363-54367)

C-1 (#C27)

Macro Observations

Spleen: 13x4x3mm
Kidneys: 10x5x4mm, symmetrical
Thymus: 9x7x3mm
Heart: 8x5x5mm
Brain: 15x10x5mm, symmetrical
Pituitary gland identified, macroscopically normal
Tail 83mm (straight)
Teeth, tongue and oral mucosa unremarkable - Head harvested for evaluation of auditory and vestibular structures
Tail suspension test for neurological defects-negative
Left hind leg-Bone marrow smear
Additional notes:
Terminal blood was difficult to take

Micro Observations

Peripheral blood smear:
Examination of the smear showed red blood cells (majority of cells shown), occasional white blood cells including segmented neutrophils and platelets (clumps). No discernible morphological changes or detectable parasites (54770)

Marrow smear:
Cells observed were readily identified as those from the erythroid and myeloid series. Conspicuous cells from the lymphoid series. Discernible and unremarkable megakaryoblasts (54315).

Mammary glands

Section shows typical mammary fat pad with developing lactiferous ducts, blood vessels and nerve bundles. Section also includes unremarkable dermal appendages
No lesions of significance
(54189)

Ovaries/Oviducts

Section shows unremarkable ovaries containing follicles at various stages of development and several corpora lutea.
Unremarkable oviduct micromorphology with typical columnar epithelium and mucosal folds.
No lesions of significance
(54188)

Uterus/Cervix/Vagina/Clitoral gland

Unremarkable architecture of the endometrium/ endometrial glands, myometrium and adventitia.Some neutrophils and low numbers of lymphocytes identified in the endometrial glands; a common finding within a non-inflamed endometrium during the phases of estrus. The micromorphology of the uterus and vagina places the animal at metestrus.
No lesions of significance
(54188, 56046)

Urinary Bladder

Unremarkable collapsed bladder with typical urothelium and detrusor muscle
No lesions of significance
(56046)

Liver/Gall bladder

Section shows typical liver parenchyma including hepatocytes, Kupffer cells, portal triads and central veins. The cytoplasm of the hepatocytes appeared vacuolated indicating a non-fasted nutritional status of the animal.
Unremarkable Gall bladder
No lesions of significance

(54186)

Stomach

Section shows unremarkable glandular portions of the stomach with limiting ridge and a portion of the non-glandular region.
No lesions of significance
(54190)

Small Intestine (Duodenum, Jejunum & Ileum)/GALT

Section shows typical mucosal villi and submucosal layers.
The Peyer's patches are large, prominent and displayed mild follicular hyperplasia with germinal centre formation, indicating a reactive state.
No lesions of significance
(54195, 54199)

Cecum/Colon/GALT

Typical mucosal folds and submucosal layers and occasional, prominent and reactive lymphoid clusters (Peyer's patch)
No lesions of significance
(54194, 54197)

Mesenteric lymph node

Section shows mesenteric lymph node with typical reactive micromorphology including mild follicular hyperplasia, germinal centre formation and mild sinus histiocytosis.
No lesions of significance
(54183)

Spleen

Unremarkable follicular pattern identified with typical red and white pulp micromorphology.
Mild extramedullary hematopoiesis identified in the red pulp of the spleen, a common finding in the mouse.
No lesions of significance
(54185)

Pancreas

Section shows representative exocrine tissue (serous acini) and endocrine tissue (islets of Langerhans).
No lesions of significance
(54185)

Kidney

Section shows a cortex, medulla, and papilla. There is a uniform distribution of Glomeruli and accompanying nephron components and the micromorphology of the convoluted and straight segment tubules was unremarkable.
No lesions of significance
(54187)

Comments:

Pathology to comment (medulla)

Adrenal glands

Section shows adrenal glands with typical cortex/medulla micromorphology.
No lesions of significance
(54187)

Salivary glands and Regional lymph nodes

Section shows submandibular, sublingual and parotid glands.
The regional lymph nodes displayed mild follicular hyperplasia with germinal centre formation, indicating a reactive state.
No lesions of significance
(54183)

Thyroids

Normal lateral lobes of the thyroid gland with typical colloid secreting follicles lined by cuboidal epithelium. Section also shows a small sheet-like mass of polygonal cells, characteristic of the parathyroid gland
No lesions of significance
(54369, 54192)

Trachea/Lungs

Section shows typical lung micromorphology demonstrating parenchyma/alveoli, bronchioles and blood vessels.
Mild degree of parenchymal and vascular congestion, judged to be artefactual.
Oesophagus with typical features including stratified squamous epithelium.
Section also shows portion of unremarkable atria and valves.
No lesions of significance
(54192)

Thymus

Section shows typical medulla/cortex distribution and micromorphology.
No lesions of significance
(54193)

Heart/chambers/vessels/valves

Typical micromorphological observed in cardiac muscle, chambers, valves and vessels of the heart.
The cardiac muscle fibres demonstrated typical features including central nuclei, branching fibres and striations.
No lesions of significance
(54201, 54192)

Skin

Typical dermal appendages and distribution. Unremarkable thin layer of striated muscle (panniculus carnosus)
No lesions of significance
(54182)

Tail

Section shows typical tail components including keratinized squamous epithelium, dense regular connective tissue, tendons, bone, bone marrow, skeletal muscle, nerves and blood vessels.
No lesions of significance
(54191)

Eyes/Harderian glands

Section shows eyes with unremarkable retina, cornea, iris, ciliary body, lens, sclera and choroid. Section also includes portion of unremarkable optic nerve and extraocular muscles. Typical branched tubuloalveolar formation of the Harderian gland.
No lesions of significance
(54200)

Brain

Sections were prepared from the standard levels of the brain:

Level I forebrain: corpus callosum, lateral ventricles and caudate putamen (Bregma 3.08mm to 1.42mm)

Level II midbrain: includes the hippocampus, thalamus,hypothalamus, lateral and third ventricles (Bregma -2.18mm to -2.80mm)

Level III hindbrain: includes the cerebellum, pons and fourth ventricle (Bregma -5.34mm to -6.64mm)

Sections of brain stained with Haematoxylin and Eosin, Luxol Fast Blue appear symmetrical with no ventricular dilation observed, unremarkable meninges and typical lamination. The cerebellum appears symmetrical with typical architecture and Purkinje cells.
(554196, 54332, 56044)

Comments:

Neuropathology to comment

Spinal cord

Representative thoracic and lumbar region of spinal cord, vertebral bone, striated muscle, peripheral nerves and bone marrow.
Sections also show white and brown fat and portion of mammary fat pad with lactiferous ducts.
No lesions of significance
(54184)

Comments:

Neuropathology to comment

(Hind leg) Long bone/Bone marrow/Synovial joint/Skeletal muscle

Section shows unremarkable long bone, striated muscle, synovial joint, ligaments and bone marrow
including conspicuous megakaryoblasts. The skeletal muscle shows consistent fiber size with peripheral nuclei
No lesions of significance
(54198)

Head

Multiple levels through the head demonstrate dermal appendages, nasal cavity,oral cavity, teeth and tongue including muscle bundles. Sections also show unremarkable pituitary gland including pars intermedia, pars distalis and pars nervosa
as well and the trigeminal nerve/ganglia (54369).The outer and middle regions of the ear are discernible. The tympanic membrane is intact and the ossicles are unremarkable and include the stapedial annular ligaments (54370, 54371).
Typical components of the inner ear including bony labyrinth, organ of corti, stria vascularis and scala cavities are discernible. Based on multiple levels, the organ of corti is unremarkable with no discernible loss of inner/outer hair cells and typical tectorial membrane (54371).
The cochlear nerve and spiral ganglion is also demonstrated and based on several levels, there is no reduction in the density of the spiral ganglion cells. Examples of otolith organs can be seen with typical features such as the hair cells and mineral otoliths. The ampulla including the crista ridge with hair cells is discernible (54371)

No lesions of significance
(54368-54371)

C-2 (#C20)

Macro Observations

Spleen: 12x4x2mm
Kidneys: 10x6x5mm, symmetrical
Thymus: 6x6x2mm
Heart: 8x6x5mm
Brain: 15x10x5mm, symmetrical
Pituitary gland identified, macroscopically normal
Tail 85mm (straight)
Teeth, tongue and oral mucosa unremarkable - Head harvested for evaluation of auditory and vestibular structures
Tail suspension test for neurological defects-negative
Left hind leg-Bone marrow smear
Additional notes:
Excessive abdominal fat
Excessive fat surrounding ovaries
Lungs show marked congestion; judged to be terminal
Terminal blood was difficult to take

Micro Observations

Peripheral blood smear:
Examination of the smear showed red blood cells (majority of cells shown), occasional white blood cells including segmented neutrophils and platelets (clumps). No discernible morphological changes or detectable parasites (54316)

Marrow smear:
Cells observed were readily identified as those from the erythroid and myeloid series. Conspicuous cells from the lymphoid series. Discernible and unremarkable megakaryoblasts (54317).

Mammary glands

Section shows typical mammary fat pad with developing lactiferous ducts, blood vessels, nerve bundles and a large lymph node with mild follicular hyperplasia. Also, unremarkable dermal appendages.
No lesions of significance
(54211)

Ovaries/Oviducts

Section shows unremarkable ovaries containing follicles at various stages of development and several corpora lutea.
Unremarkable oviduct micromorphology with typical columnar epithelium and mucosal folds.
No lesions of significance
(54210)

Uterus/Cervix/Vagina/Clitoral gland

Unremarkable architecture of the endometrium/ endometrial glands, myometrium and adventitia. The endometrial stroma appeared distended judged to be estrus related. Some neutrophils and low numbers of lymphocytes identified in the endometrial glands; a common finding within a non-inflamed endometrium during the phases of estrus.
The micromorphology of the uterus and vagina places the animal at proestrus.
No lesions of significance
(54210)

Urinary Bladder

Unremarkable collapsed bladder with typical urothelium and detrusor muscle
No lesions of significance
(54210)

Liver/Gall bladder

Section shows typical liver parenchyma including hepatocytes, Kupffer cells, portal triads and central veins. The cytoplasm of the hepatocytes appeared vacuolated indicating a non-fasted nutritional status of the animal.
Unremarkable Gall bladder
No lesions of significance
(54212)

Stomach

Section shows unremarkable glandular portions of the stomach and a portion of the non-glandular region. marked artefactual tearing of section.
(54221)

Small Intestine (Duodenum, Jejunum & Ileum)/GALT

Section shows typical mucosal villi and submucosal layers.
The Peyer's patches are large, prominent and displayed mild follicular hyperplasia with germinal centre formation, indicating a reactive state.
No lesions of significance
(54202, 54206)

Cecum/Colon/GALT

Typical mucosal folds and submucosal layers and occasional, prominent and reactive lymphoid clusters (Peyer's patch)
No lesions of significance
(54203, 54204)

Mesenteric lymph node

Section shows mesenteric lymph node with typical reactive micromorphology including mild follicular hyperplasia, germinal centre formation and mild sinus histiocytosis.
No lesions of significance
(54216)

Spleen

Unremarkable follicular pattern identified with typical red and white pulp micromorphology.
Mild extramedullary hematopoiesis identified in the red pulp of the spleen, a common finding in the mouse.
No lesions of significance
(54214)

Pancreas

Section shows representative exocrine tissue (serous acini) and endocrine tissue (islets of Langerhans).
No lesions of significance
(54214)

Kidney

Section shows a cortex, medulla, and papilla. There is a uniform distribution of Glomeruli and accompanying nephron components and the micromorphology of the convoluted and straight segment tubules was unremarkable.
(54213)

Comments:

Pathology to comment (medulla)

Adrenal glands

Section shows adrenal glands with typical cortex/medulla micromorphology.
No lesions of significance
(54213)

Salivary glands and Regional lymph nodes

Section shows submandibular, sublingual and parotid glands.
The regional lymph nodes displayed mild follicular hyperplasia with germinal centre formation, indicating a reactive state.
No lesions of significance
(54216)

Thyroids

Sections do not include thyroid glands

Trachea/Lungs

Section shows typical lung micromorphology demonstrating parenchyma/alveoli, bronchioles and blood vessels.
Mild degree of parenchymal and vascular congestion, judged to be artefactual.
Oesophagus with typical features including stratified squamous epithelium.
Trachea with unremarkable mucosal epithelial lining and hyaline cartilage.
Section also shows portion of unremarkable atria and valves.
No lesions of significance
(54219)

Thymus

Section shows typical medulla/cortex distribution and micromorphology.
No lesions of significance
(54218)

Heart/chambers/vessels/valves

Typical micromorphological observed in cardiac muscle, chambers, valves and vessels of the heart.
The cardiac muscle fibres demonstrated typical features including central nuclei, branching fibres and striations.
No lesions of significance
(54208)

Skin

Typical dermal appendages and distribution. Unremarkable thin layer of striated muscle (panniculus carnosus)
No lesions of significance
(54217)

Tail

Section shows typical tail components including keratinized squamous epithelium, dense regular connective tissue, tendons, bone, bone marrow, skeletal muscle, nerves and blood vessels.
No lesions of significance
(54220)

Eyes/Harderian glands

Section shows eyes with unremarkable retina, cornea, iris, ciliary body, lens, sclera and choroid. Section also includes portion of unremarkable optic nerve. Typical branched tubuloalveolar formation of the Harderian gland.
No lesions of significance
(54209)

Brain

Sections were prepared from the standard levels of the brain:

Level I forebrain: corpus callosum, lateral ventricles and caudate putamen (Bregma 0.26 to -0.22mm)

Level II midbrain: includes the hippocampus, thalamus,hypothalamus, lateral and third ventricles (Bregma -2.18mm to -2.92mm)

Level III hindbrain: includes the cerebellum, pons and fourth ventricle (Bregma -5.68mm)

Sections of brain stained with Haematoxylin and Eosin, Luxol Fast Blue appear symmetrical with no ventricular dilation observed, unremarkable meninges and typical lamination. The cerebellum appears symmetrical with typical architecture and Purkinje cells.
(54205, 54333)

Comments:

Neuropathology to comment

Spinal cord

Representative thoracic and lumbar region of spinal cord, vertebral bone, intervertebral disc, striated muscle, peripheral nerves and bone marrow.

No lesions of significance
(54215)

Comments:

Neuropathology to comment

(Hind leg) Long bone/Bone marrow/Synovial joint/Skeletal muscle

Section shows unremarkable long bone, striated muscle, synovial joint, ligaments and bone marrow
including conspicuous megakaryoblasts. The skeletal muscle shows consistent fiber size with peripheral nuclei
No lesions of significance
(54207)

Head

Multiple levels through the head demonstrate dermal appendages, nasal cavity,oral cavity, teeth and tongue including muscle bundles. Sections also show unremarkable pituitary gland including pars intermedia, pars distalis and pars nervosa
as well and the trigeminal nerve/ganglia (54375).The outer and middle regions of the ear are discernible. The tympanic membrane is intact and the ossicles are unremarkable and include the stapedial annular ligaments (54376, 54377, 55926).
Typical components of the inner ear including bony labyrinth, organ of corti, stria vascularis and scala cavities are discernible. Based on multiple levels, the organ of corti is unremarkable with no discernible loss of inner/outer hair cells and typical tectorial membrane (54377, 55925).
The cochlear nerve and spiral ganglion is also demonstrated and based on several levels, there is no reduction in the density of the spiral ganglion cells. Examples of otolith organs can be seen with typical features such as the hair cells and mineral otoliths. The ampulla including the crista

ridge with hair cells is discernible (54377, 55926)

No lesions of significance
(54373-54377,55925-55926)

C-3 (#C29)

Macro Observations

Spleen: 15x4x3mm
Kidneys: 10x6x4mm, symmetrical
Thymus: 8x6x2mm
Heart: 8x6x5mm
Brain: 15x11x6mm, symmetrical
Pituitary gland identified, macroscopically normal
Tail 84mm (straight)
Teeth, tongue and oral mucosa unremarkable - Head harvested for evaluation of auditory and vestibular structures
Tail suspension test for neurological defects-negative
Left hind leg-Bone marrow smear

Micro Observations

Peripheral blood smear:
Examination of the smear showed red blood cells (majority of cells shown), occasional white blood cells including segmented neutrophils and platelets (clumps). No discernible morphological changes or detectable parasites (54319)

Marrow smear:
Cells observed were readily identified as those from the erythroid and myeloid series. Conspicuous cells from the lymphoid series. Discernible and unremarkable megakaryoblasts (54318).

Mammary glands

Section shows typical mammary fat pad with developing lactiferous ducts, blood vessels, nerve bundles and a large lymph node with mild follicular hyperplasia.
No lesions of significance
(54236)

Ovaries/Oviducts

Section shows unremarkable ovaries containing follicles at various stages of development and several corpora lutea.
Unremarkable oviduct micromorphology with typical columnar epithelium and mucosal folds.
No lesions of significance
(54237)

Uterus/Cervix/Vagina/Clitoral gland

Unremarkable architecture of the endometrium/ endometrial glands, myometrium and adventitia.Some neutrophils and low numbers of lymphocytes identified in the endometrial glands; a common finding within a non-inflamed endometrium during the phases of estrus. The micromorphology of the uterus and vagina places the animal at proestrus.
No lesions of significance
(54237)

Urinary Bladder

Unremarkable collapsed bladder with typical urothelium and detrusor muscle
No lesions of significance
(54237, 56047)

Liver/Gall bladder

Section shows typical liver parenchyma including hepatocytes, Kupffer cells, portal triads and central veins. The cytoplasm of the hepatocytes appeared vacuolated indicating a non-fasted nutritional status of the animal.
Section does not include Gall bladder
No lesions of significance
(54235)

Stomach

Section shows unremarkable glandular portions of the stomach with limiting ridge and a portion of the non-glandular region.
No lesions of significance
(54224)

Small Intestine (Duodenum, Jejunum & Ileum)/GALT

Section shows typical mucosal villi and submucosal layers.
The Peyer's patches are prominent and displayed mild follicular hyperplasia with germinal centre formation, indicating a reactive state.
No lesions of significance
(54229, 54233)

Cecum/Colon/GALT

Typical mucosal folds and submucosal layers and occasional, prominent and reactive lymphoid clusters (Peyer's patch)
No lesions of significance
(54227, 54228)

Mesenteric lymph node

Section shows mesenteric lymph node with typical reactive micromorphology including mild follicular hyperplasia, germinal centre formation and mild sinus histiocytosis.
No lesions of significance
(54238)

Spleen

Unremarkable follicular pattern identified with typical red and white pulp micromorphology.
Mild extramedullary hematopoiesis identified in the red pulp of the spleen, a common finding in the mouse.
No lesions of significance
(54240)

Pancreas

Section shows representative exocrine tissue (serous acini) and endocrine tissue (islets of Langerhans).
No lesions of significance
(54240)

Kidney

Section shows a cortex, medulla, and papilla. There is a uniform distribution of Glomeruli and accompanying nephron components and the micromorphology of the convoluted and straight segment tubules was unremarkable.
(54234)

Comments:

Pathology to comment

Adrenal glands

Section shows adrenal glands with typical cortex/medulla micromorphology.
No lesions of significance
(54234)

Salivary glands and Regional lymph nodes

Section shows submandibular, sublingual and parotid glands.
The regional lymph nodes displayed mild follicular hyperplasia with germinal centre formation, indicating a reactive state.
No lesions of significance
(54238)

Thyroids

Section does not include thyroid glands

Trachea/Lungs

Section shows typical lung micromorphology demonstrating parenchyma/alveoli, bronchioles and blood vessels.
Mild degree of parenchymal and vascular congestion, judged to be artefactual.
Oesophagus with typical features including stratified squamous epithelium.
Section also includes portion of typical heart atria and aorta.
No lesions of significance
(54222)

Thymus

Section shows typical medulla/cortex distribution and micromorphology.
No lesions of significance
(54223)

Heart/chambers/vessels/valves

Typical micromorphological observed in cardiac muscle, chambers, valves and vessels of the heart.
The cardiac muscle fibres demonstrated typical features including central nuclei, branching fibres and striations.
No lesions of significance
(54231, 54222)

Skin

Typical dermal appendages and distribution. Unremarkable thin layer of striated muscle (panniculus carnosus).
Mild, focal peri-follicular inflammation.
Mild, multifocal epidermal hyperplasia with a papillated appearance in some areas.
(54239)

Comments:

Pathology to comment

Tail

Section shows typical tail components including keratinized squamous epithelium, dense regular connective tissue, tendons, bone, bone marrow, skeletal muscle, nerves and blood vessels.
No lesions of significance
(54225)

Eyes/Harderian glands

Section shows eyes with unremarkable retina, cornea, iris, ciliary body, lens, sclera and choroid. Typical branched tubuloalveolar formation of the Harderian gland.
No lesions of significance
(54230)

Brain

Sections were prepared from the standard levels of the brain:

Level I forebrain: corpus callosum, lateral ventricles and caudate putamen (Bregma 0.62mm)

Level II midbrain: includes the hippocampus, thalamus,hypothalamus, lateral and third ventricles (Bregma -1.70mm)

Level III hindbrain: includes the cerebellum, pons and fourth ventricle (Bregma -5.68mm)

Sections of brain stained with Haematoxylin and Eosin, Luxol Fast Blue appear symmetrical with no ventricular dilation observed, unremarkable meninges and typical lamination. The cerebellum appears symmetrical with typical architecture and Purkinje cells.
(54226, 54334)

Comments:

Neuropathology to comment

Spinal cord

Representative thoracic and lumbar region of spinal cord, vertebral bone, striated muscle, peripheral nerves and bone marrow.
No lesions of significance
(54241)

Comments:

Neuropathology to comment

(Hind leg) Long bone/Bone marrow/Synovial joint/Skeletal muscle

Section shows unremarkable long bone, striated muscle, synovial joint, ligaments and bone marrow
including conspicuous megakaryoblasts. The skeletal muscle shows consistent fiber size with peripheral nuclei
No lesions of significance
(54232)

Head

Multiple levels through the head demonstrate dermal appendages, nasal cavity,oral cavity, teeth and tongue including muscle bundles. Sections also show unremarkable pituitary gland including pars intermedia, pars distalis and pars nervosa
as well and the trigeminal nerve/ganglia (54379).The outer and middle regions of the ear are discernible. The tympanic membrane is intact and the ossicles are unremarkable and include the stapedial annular ligaments (54382, 55927,55928).
Typical components of the inner ear including bony labyrinth, organ of corti, stria vascularis and scala cavities are discernible. Based on multiple levels, the organ of corti is unremarkable with no discernible loss of inner/outer hair cells and typical tectorial membrane (55928, 55929).
The cochlear nerve and spiral ganglion is also demonstrated and based on several levels, there is no reduction in the density of the spiral ganglion cells. Examples of otolith organs can be seen with typical features such as the hair cells and mineral otoliths. The ampulla including the crista ridge with hair cells is discernible (55928-55930)
No lesions of significance
(54378-54382, 55927-55930)

Comment / Plan

Case APN16/016 will be referred to Professor Rolfe Howlett R&A Pathology Services NSW and Professor Catriona McLean, Anatomical Pathology, Alfred Hospital Melbourne for comment.

Tina Cardamone
21st June, 2016

Supplementary Pathology Report

Control-1 (control)

Control-2 (control)

Control-3 (control)

A-1 (#A137)

Peripheral Blood Smear

The majority of the cells are erythrocytes throughout which are scattered neutrophils, lymphocytes and clumps of platelets.
No lesions of significance.

Bone marrow smear

Cells from the erythroid, myeloid and lymphocytic series are readily recognized as are occasional megakaryocytes.

Ovary/Fallopian tubes

In both ovaries several follicles at different stages of development as well as Corpora Lutea had formed. The oviducts (Fallopian tubes) present have no features of abnormality.

Uterus/Vagina

The horns of the uterus lead into the cervix and vagina. Changes particularly on the vaginal surface place this mouse at metoestrus.
No lesions of significance.

Mammary glands

Sections display the mammary gland with lactiferous compound tubuloalveolar.
Glands scattered through the mammary fat pad. A small lymph node is present in the fat pad.
The microscopic pattern of the mammary fat pad is normal.
No lesions of significance.

Liver /Gall Bladder

Sections from liver and gallbladder display unremarkable microscopic features with no lesions of significance.

Oesophagus

A longitudinal section of the oesophagus in the mediastinum does not show any lesion of significance.

Stomach

The section reveals the glandular and non-glandular regions of the stomach separated by the limiting ridge.
No lesions of significance.

Small Intestine

Sections of the duodenum, jejunum and ilium display no lesions of significance.
The Peyer's patches in the ilium were prominent some displaying follicular hyperplasia.
An indication of immunogenic stimulation.
No lesions of significance.

Large Intestine /Caecum

Typical mucosal folds and the walls of the large bowel are unremarkable.
Lymphocytic nodules in the large bowel walls display follicular hyperplasia; induced immunogenicity.
No lesions of significance.

Mesenteric Lymph Node

Sections reveal follicular hyperplasia with germinal centers as well as sinus histiocytosis.
No lesions of significance.

Spleen

The spleen has a nodular pattern with lymphoid cells forming dense islands separated by the red pulp stroma containing many red blood cells. Mild extramedullary haematopoiesis was present.
No lesions of significance.

Pancreas

The dominant component of the pancreas is an exocrine gland (compound tubuloalveolar gland or a compound acinar gland).The endocrine gland consists of a number of islets of Langerhans.
The structure and the micromorphology is as expected in a normal gland.
No lesions of significance.

Kidneys

Both kidneys were sectioned and do not reveal any specific lesion.
The cortex merges into the medulla which flows into a renal papilla that fills and occupies much of the renal pelvis.
The tubular portions of the nephron that reside in the medulla and also within the single renal papilla are patent and do not contain any obstructive proteinaceous material in their lumens nor any cellular response within the interstitium in cortex, medulla and renal papilla. The ureter appears to have a few epithelial cells in the lumen. However, in my opinion these cells have dislodged in sectioning.
No lesions of significance

Comments:

If suspecting a lesion within the medulla (e.g. urinary concentration) and light microscopic appraisal does not reveal any micromorphological lesion suggest the research team should appraise the micro-morphology using electron microscopy. Alternatively urinalysis should be undertaken.

Adrenal glands

Both adrenals were present in the section and do not display any feature of abnormality.
No lesions of significance.

Thyroid glands

The thyroid straddles both sides of the trachea. The colloid containing acini are as expected in a normal mouse.
No lesions of significance.

Salivary glands and associated Lymph nodes

Glands of the parotid, submandibular and sublingual are present. No lesions of significance was present. The regional lymph nodes were reactive displaying follicular hyperplasia and sinus histiocytosis.
No lesions of significance.

Lungs/ Trachea /Bronchi

The right and left lungs were present as was the most proximal portion of the trachea as entering the oral cavity. The pulmonary parenchyma including airways, alveolae and vascular network are all unremarkable
No lesions of significance.

Thymus

Typical cortex and medulla with expected cell types.
No lesions of significance.

Heart / major vessels / valves

The sections mostly include the ventricular muscle. However in one section of the heart there is a small part of the atria-ventricular valve that has a small fibrotic nodule (about 100 microns in diameter) with spindle cells and accompanying myxomatous matrix. (section# 54074)
A cross section of the aorta was present in section #54079; the features were normal.

Skin

Epidermis, dermis and dermal appendages are unremarkable although a light scattering of mast cells is present in the dermis and upper hypodermis. The Panniculus carnosus has no lesions of significance.
Mast cells do occur in murine skin that appears microscopically normal.
No lesion of significance.

Eyes / Harderian glands

Microscopic sections for both eyes reveal normal microscopic detail in all critical layers of the retina, iris, cornea, lens ciliary body, sclera and choroid.
Additionally the Harderian glands are normal
No lesion of significance.

Skeletal Tissue (appendicular skeleton)/ synovial joint/ skeletal muscle

Sections included the femur, tibia, patella, patella ligament and anterior cruciate ligament. The femoral and tibial epiphyses as well as the marrow cavities are with active marrow with a light scattering of megakaryocytes.
No lesions of significance.

Head

Six levels were prepared from this site.
The nasal and oral cavities with teeth appear normal as did the skin covering the head.
The pituitary gland, with all its regions, is present and has no lesions of significance.
The outer and middle ear are normal as were the ossicles and tympanic membrane is intact.
The levels readily allowed identification of the features of the inner ear e.g. bony labyrinth, organ of Corti and Scala cavities, cochlear duct and the organ of Corti with tectorial membrane and the inner and outer hair cells.
Most importantly the spiral ganglion cells appear not to have reduction in density from level to level.
No lesions of significance were observed.

A-2 (#A140)

Peripheral Blood Smear

The majority of cells are erythrocytes. The common nucleated cells are neutrophils and lymphocytes accompanied by platelet clumps.
No abnormalities observed.

Bone marrow smear

Cells of erythroid and myeloid series as well as the lymphocytic series are readily observed.
Megakaryocytes are discernible and do not display any feature of abnormality.
Additionally in the sections cut from the appendicular skeleton, cells of erythroid and myeloid series fill the tibial and femoral cavities without lesions of significance.
Megakaryocytes are also obvious in the marrow.
No lesions of significance.

Ovary/ Fallopian tubes

Several follicles at different development stages as well as corpora Lutea occurred in both ovaries. The fallopian tubes together with the ovaries have no features or lesions of significance.

Uterus / Vagina

The features of uterine horns and the composite endometrium, endometrial glands and myometrium are normal throughout which are a scattering of neutrophils. The latter observation is consistent with the mouse, being commonly observed in various stages of oestrus. There are no lesions of significance. No vagina is present in the section.
No lesions of significance.

Mammary glands

Typical mammary fat pad with developing lactiferous glands.
A small lymph node (1.5mm in length) is present.
The skin covering the mammary gland is without lesions and a few normal mast cells are observed in the dermis.
No lesions of significance.

Urinary Bladder

The in-tact bladder wall is collapsed; the lining epithelium and the muscular wall are normal. One ureter entering the bladder is present.
There are no lesions of significance.

Liver /Gall Bladder

This major organ and its gall bladder display no features or lesions of significance.

Oesophagus

Longitudinal sections from the thoracic portion of the oesophagus display superficial erosions limited mainly to the keratinous surface. In the keratinous erosions are bacterial colonies but no inflammatory microscopic features has occurred in the epithelial or muscular coats of the oesophagus.

Stomach

The section displays the glandular and non-glandular portions of the stomach separated by the limiting ridge.
There are no lesions of significance.

Small Intestine

Normal with no lesions of significance.
Lymph nodes in the wall of the small intestine(Peyer’s patches) display nodular hyperplasia, often with a germinal center, indicative of immune reactivity.
No lesions of significance.

Large Intestine /Caecum

Typical mucosal folds and epithelial lining.
Minimal lymphocytic islands in the intestinal wall. There appeared to be little in the way of immune reactivity.
No lesions of significance.

Mesenteric Lymph Node

A small portion of the node.
No lesions of significance.

Spleen

A follicular pattern formed by islands of white pulp enclosed by red pulp. The latter is formed mainly by erythrocytes supported on the splenic stroma.
No lesions of significance.

Pancreas

The exocrine and endocrine (Islets of Langerhans) portions of this gland are easily recognized and there are no lesions of significance.

Kidneys

Sections of both kidneys reveal no lesions of significance in the cortex, medulla and renal papillae. The latter enter the normal renal pelves. The tubular structures that course from the medulla into the renal papilla do not display any feature of significance and their lumens do not contain any material e.g. proteinaceous or cellular material.
No lesions of significance.

Adrenal Glands

Both glands are present and show no lesions of significance.

Thyroid Glands

Normal colloid secreting lateral lobes of the thyroid gland.
The colloid follicles are lined by cuboidal epithelium.
No lesions of significance.

Salivary glands and associated Lymph nodes

Parotid, submandibular and sublingual glands are present and accompanied by regional lymph nodes. The latter displays mild immunogenic reactivity.
No significant lesions.

Lungs/ Trachea /Bronchi

Sections include normal pulmonary parenchyma that includes vascular tributaries and alveolar sacs as well as airways.
No lesions of significance.

Thymus

The cortex and medulla of the thymus are present.
No lesions of significance.

Heart / major vessels / valves

Left and right atria and corresponding ventricles are identified as normal. Only small portions of the large vessels at the base of the heart are available in the section.
However, at the terminal end of a valve (possibly the aortic valve) there is a myxomatous thickening of the connective tissue.

Comments:

Deeper levels will be required from this block should a more definitive identification and location be required. Additionally the valve may have to be re-embedded to ensure that valve is locally thickened for at the moment the terminal end appears to have rolled.
Furthermore, it is worth noting that Robert R. Maronpot editor of "Pathology of the Mouse" Page 362 reports that typically in mice there is a pale myxomatous appearance to the connective tissue of the aortic valve when stained with H & E.

Skin

Sections display the epidermis, dermis, dermal appendages as well as the hypodermis and the Panniculus carnosus. No lesions of significance were encountered.

Eyes / Harderian glands

Sections from both eyes display retina, lens, cornea, ciliary body, iris and sclera having normal microscopic features. The Harderian glands were also were without lesions or abnormalities.
No lesions of significance.

Spinal Cord /vertebrae

The sections from the spinal cord at different levels does not display any microscopic lesion of the vertebral bone or of the intervertebral disc.
The spinal cord is symmetrical and appears not to have any local lesion.

Skeletal Tissue (appendicular skeleton)/ synovial joint/ skeletal muscle

The appendicular skeleton including portions of the femur and tibia separated by a normal styfle joint including the patella and the patellar ligament. The striated skeletal muscle is composed of fibres of consistent size and peripheral nuclei.
No lesions of significance are present.

Head

Sections from 6 levels display the features as described in the preliminary report.
Sections reveal normal nasal and oral cavities with tongue and teeth.
The pituitary gland is displayed in slide 54344 showing the component parts viz; Pars intermedia,
pars distalis and pars nervosa and nearby trigeminal nerve.
Furthermore, sections display ;
The outer and middle ear with intact tympanic membrane and ossicles
The inner ear including the bony labyrinth, cochlear duct , organ of Corti, and Scala Cavities with tectorial membrane and associated inner and outer hair cells.
No reduction in the density of spiral ganglion cells over several levels.
And micro-organs with otoliths buried in a covering cupula.
No lesions or abnormal features of significance were observed.

A-3 (#A138)

Peripheral Blood Smear

The majority of the cells are erythrocytes throughout which are a light scattering of neutrophils and lymphocytes as well as platelet clumps. No lesions or features of abnormality.

Bone Marrow Smear

Microscopic anatomical sections reveal cells of erythroid and myeloid series which fill the tibial and femoral cavities without lesions of significance.
Megakaryocytes are obvious in these marrow sections.
In the marrow smear cells from the erythroid and myeloid series as well as lymphocytic series are readily identified and those few megakaryocytes encountered are discernible and have no abnormalities.
No lesions of significance.

Ovary/ Fallopian tubes

Several follicles and corpora Lutea at different stages in both ovaries. The fallopian tubes together with the ovaries have no features of significance.

Uterus / Vagina

The features of uterus and vagina are those observed in metoestrus. The low numbers of neutrophils and lymphocytes very lightly scattered through the myometrium particularly in the endometrial glands is consistent with the mouse being in one of the stages of oestrus.
There are no lesions of significance.

Mammary glands

A small portion of the mammary fat pad displayed developing lactiferous glands.
No lesions of significance.

Urinary Bladder

The bladder has collapsed.
There are no lesions in the urothelium or the Tunica muscularis
No lesions of significance.

Liver /Gall Bladder

This major organ and its gall bladder display no features or lesions of significance.
A small area of oesophagus lined by stratified squamous cell epithelium is buried in connective tissue adjoining the liver.

Oesophagus

A Longitudinal section from the thoracic portion of the oesophagus is displayed in the section taken from the lung.
No lesions of significance.

Stomach

The section displays the glandular and non-glandular portions of the stomach separated by the limiting ridge.
There are no lesions of significance.

Small Intestine

Sections from the duodenum, jejunum and ileum reveal unremarkable microscopic features.
Lymph nodes in the wall of the small intestine display nodular hyperplasia indicative of immune reactivity.
No lesions of significance.

Large Intestine /Caecum

Typical mucosal folds with a normal mucosa and Tunica muscularis.
A few lymphoid nodules in the intestinal wall; a few displayed a mild degree of immunogenic activity.
No lesions of significance.

Mesenteric Lymph Node

Mild sinus histiocytosis and follicular hyperplasia; indicative of a mild immunogenic response.
No lesions of significance.

Spleen

Follicular pattern formed with white pulp (nucleated cells mostly lymphocytes) and red pulp (mainly erythrocytes supported in the splenic stroma) cell types. Mild extramedullary haematopoiesis; a very common finding in mice.
No lesions of significance.

Pancreas

The exocrine (compound tubuloacinar gland) and endocrine portions (Islets of Langerhans) of this gland are easily recognized.
No lesions of significance.

Kidneys

Sections from both kidneys reveal a mild difference. One kidney appears not to have any lesion while the other has mild observable changes. The latter kidney has small aggregates of round cells (mainly lymphocytes) in the renal pelvis wall as well there is a perivascular nodule (0.05mm in diameter) of lymphocytes in the supporting stroma. Such pattern may suggest a previous ascending infection that has healed. However, in the main there are no lesions in the cortex, glomeruli and medulla. On close examination the tubular structures that course from the medulla into the renal papilla do not display any feature of significance and their lumens do not contain any material e.g. proteinaceous or cellular material and nothing in the renal pelvis.
The normal kidney had no lesions of significance in the cortex, medulla and renal papilla that enters the renal pelvis.

Adrenal glands

Both glands were present and show a typical cortex and medulla.
No lesions of significance.

Thyroid glands

Colloid secreting thyroid gland on both sides of the trachea.
No lesions of significance.

Salivary glands and associated Lymph nodes

Parotid, submandibular and sublingual glands are present with no apparent lesions.
The accompanying regional lymph nodes display a minor immune reactivity.
No significant lesions.

Lungs/ Trachea /Bronchi

Sections display left and right lungs with extra and intra pulmonary airways as well as pulmonary alveolar sacs. There is no evidence of abnormalities or pathology.
No significant lesions.

Thymus

The cortex and medulla of the thymus were unremarkable.
No lesions of significance.

Heart / major vessels / valves

One section displays ventricular muscle only, the other section displays ventricular muscle and a few tissue fronds possibly from heart valves rather than the wall of the atrium. There does appear to be a couple of cellular nodules with a myxomatous connective tissue.

Comments:

Suggest deeper sections(levels) to ascertain the presumptive changes and their location. Furthermore, It may be worth re-embedding the organ with attention to structures on the base of the heart and ensure to include the large vessels of the heart.

Skin

Sections display the epidermis, dermis, dermal appendages as well as the upper hypodermis and the Panniculus carnosus. No lesions of significance were encountered.

Eyes / Harderian glands

The eyes and all the known structures for each eye were identified and unremarkable; no lesions of significance.
Typical branched tubuloalveolar glands(Harderian glands) from both eyes are devoid of any significant lesions.
No lesions of significance.

Skeletal Tissue (appendicular skeleton)/ synovial joint/ skeletal muscle

Portions of the appendicular skeleton in the sections and the styfle joint have no significant lesions.
The growth zones were still functioning albeit minimally.
The striated skeletal muscle have consistent fibre size with peripheral nuclei.
No lesions of significance.

Head

Sections from 6 levels display the features as described in the preliminary report.
In details sections reveal normal nasal and oral cavities with tongue and teeth.
The pituitary gland is displayed in slide 54349 showing the component parts viz; Pars intermedia, pars distalis and pars nervosa.
Furthermore, sections display;
The outer and middle ear with intact tympanic membrane and ossicles.
The inner ear including the bony labyrinth, cochlear duct , organ of Corti, and Scala Cavities with tectorial membrane and associated inner and outer hair cells.
No reduction in the density of spiral ganglion cells over several levels.
And micro-organs with otoliths buried in a covering cupula.
No lesions or abnormal features of significance were observed.

B-1 (#B126)

Peripheral blood smear

Mainly red blood cells but scattered throughout are platelet clumps, lymphocytes and neutrophils. No abnormalities detected.

Bone marrow smear

Cells from the erythroid and myeloid series together with precursors of lymphocytes and megakaryocytes are readily detected.

Ovary/ Fallopian tubes

Both ovaries were sectioned and display numerous follicles at varying stages of maturation as well as at least two corpora lutea.
No lesions of significance.

Uterus / Vagina

The uterus and vagina have a normal appearance. The features of the vaginal lumenal surface would place this female mouse at the Dioestrus stage of the oestrus cycle.
There are no lesions of significance.

Mammary glands

The gland has lactiferous glands scattered through the background of adipose tissue.
No lesions of significance.

Urinary Bladder

The bladder shape, lining epithelium and the Tunica muscularis display no features of abnormal significance.

Liver /Gall Bladder

The liver and the accompanying gall bladder show no lesions of significance.

Oesophagus

The thoracic portion of the oesophagus is without lesions of significance.

Stomach

Sections include the glandular and non glandular regions, separated by the limiting ridge, of the stomach.
No lesions of significance .

Small Intestine

The features are normal. Additionally the aggregated Lymphatic nodules show nodular hyperplasia with germinal centers; such a pattern is representative of local/regional immunogenic activity.
No lesions of significance.

Large Intestine /Caecum

The features are normal. Additionally the aggregated Lymphatic nodules show nodular hyperplasia with germinal centers.
No lesions of significance.

Mesenteric Lymph Node

Prominent follicular hyperplasia and sinus histiocytosis of this lymph node.
No lesions of significance.

Spleen

A nodular pattern of nucleated cells scattered through the splenic parenchyma dominantly occupied by erythrocytes. A mild extramedullary haematopoiesis.
No lesions of significance.

Pancreas

This glandular pattern consists mainly of exocrine serous acini throughout which are islets of Langerhans (endocrine tissue).
No lesions of significance.

Kidneys

Both kidneys were sectioned and do not reveal any specific lesion.
The cortex merges into the medulla which flows into a renal papilla that fills and occupies much of the renal pelvis.
The tubular portions of the nephron that reside in the medulla and also within the single renal papilla are patent and do not contain any obstructive proteinaceous material in their lumens nor any cellular response within the interstitium in cortex, medulla and renal papilla.
No lesions of significance.

Comments:

If suspecting a lesion within the medulla (e.g. urinary concentration) and light microscopic appraisal does not reveal any micromorphological lesion suggest the research team should appraise the micro-morphology using electron microscopy. Alternatively urinalysis should be undertaken.

Adrenal glands

Micromorphology of the cortex and medulla of the one adrenal gland sectioned show no lesions of significance .

Thyroid glands

Colloid secreting portions of the gland do not display any lesions of significance.

Salivary glands and associated Lymph nodes

Sections include the parotid, submandibular and sublingual salivary glands as well as the regional lymph nodes. The latter display nodular hyperplasia with germinal centers; features indicative of immunogenic reactivity.

Lungs/ Trachea /Bronchi

The lungs together with extra and intra pulmonary airways do not display any lesion of significance.
No lesions of significance.

Thymus

Typical cortex and medulla morphology.
No lesions of significance.

Heart / major vessels / valves

Sections display portions of the left and right ventricles and atria as well as large vessels at the base of the heart.
No lesions of significance.

Skin

The skin has regularly spaced dermal appendages. This layer overlies the upper hypodermis that in turn covered the thin muscle band (Panniculus carnosus).
No lesions of significance.

Eyes / Harderian glands

Both eyes have a normal micromorphology as do their Harderian glands.
No lesions of significance.

Skeletal Tissue (appendicular skeleton)/ synovial joint/ skeletal muscle

The bones from the appendicular skeleton appear normal. The proximal tibial and distal femur still have functional growth plates although the tibial growth plate shows peripheral margins. Displaying features signifying early closure. The marrow cavities and the epiphyses of tibia and femur are filled with active marrow. The skeletal muscle is striated and appeared normal.
No lesions of significance.

Head

Six slides were prepared from different levels.
The sections reveal the nasal cavity and the oral cavity with teeth; the head is covered by skin.
The pituitary gland with its subsections was located and is micromorphologically normal.
The outer and middle ear, with ossicles, are displayed as well as the inner ear. No lesions of note or significance.
The cochlear with components, are readily identifiable and do not display any lesions of significance. The scala cavities, vestibular membrane, tectorial membrane, the hair cells and

spiral ganglion of the cochlear nerve are readily identified. Finally the ganglion cells at different section levels do not show any decrease in cellular numbers/density.
No lesions of significance.

B-2 (#B133)

Peripheral Blood Smear

Mainly red blood cells with a scattering of neutrophils, small lymphocytes and platelet clumps.
No lesion of significance.

Bone marrow smear

Cells were readily identified from the erythroid and myeloid series as well as the lymphocytic series and multinucleated megakaryocytes.
No lesion of significance.

Ovary/ Fallopian tubes

Multiple follicular at varying stages of development as well as a number of corpora Lutea.
No lesion of significance.

Uterus / Vagina

The uterine architecture displayed in the section is consistent with normal uterine and vaginal micromorphology. This mouse is at the dioestrus stage in the oestrus cycle.
No lesion of significance.

Mammary glands

The section from this site displayed numerous lactiferous glands at early development.
No lesion of significance.

Urinary Bladder

The bladder wall lined by transitional epithelium has a normal muscular coat.
No lesion of significance.

Liver /Gall Bladder

The liver is populated by normal hepatocytes and structural support and vascular cells. There are no lesions of significance in the liver or the accompanying gall bladder.
No lesion of significance.

Oesophagus

A section of the thoracic oesophagus was included in the section of the lung.
The oesophagus displayed no lesion of significance.

Stomach

The section included both the glandular and non glandular portions of the gastric wall with the limiting ridge separating the two major divisions of the stomach.
Coursing from limiting ridge a band of neutrophils and lymphocytes have infiltrated into the mucosa and into the tunica muscularis. This is a mild acute gastritis. I have noticed this region not infrequently displays this mild inflammatory response in young mice. In older mice an increase in fibrosis occurs in this site. The lesion has to be judged as significant albeit mild.

Small Intestine

The small intestine has a normal profile for its wall including its mucosa. The lymphocytic aggregate nodules were prominent some with germinal centers indicative of immunogenic activity.
No lesion of significance.

Large Intestine /Caecum

The sections display no features of significance.

Mesenteric Lymph Node

The nodes display follicular formation with germinal centers as well as a mild sinus histiocytosis.
No lesion of significance.

Spleen

The follicular pattern created by islands of nucleated cells separated by the red marrow where erythrocytes are dominant in the splenic stroma, the pattern is as expected and cell types are normal.
Mild extramedullary haematopoiesis was observed; a change very frequently observed in mice.
No lesion of significance.

Pancreas

The exocrine and endocrine glandular components occur in the pancreatic tissue.
No lesion of significance.

Kidneys

The cortex, medulla and the renal papilla sited in the pelvises of both kidneys appear microscopically without lesions of significance. Circumferentially around one renal pelvis is a light scattering of lymphocytic foci, a feature of no significance.
Para-renal lymph node displays a mild sinus histiocytosis.
No lesion of significance.

Adrenal glands

Only one gland occurrs in the section. The micromorphology reveals an appropriate cortex and medulla.
No lesion of significance.

Thyroid glands

Unremarkable colloid secreting follicular gland with normal follicular epithelium.
No lesions of significance.

Salivary glands and associated Lymph nodes

Sections have sampled the parotid, sublingual and submandibular salivary glands.
Periductal lymphocytic accumulation is apparent in two lobes of the submandibular salivary gland; a patchy mild chronic periductal adenitis. Such findings are relatively frequent encountered in mice and is consistent of an ascending bacterial infection (the cellular pattern is consistent with the inflammation being healed / quiescent. The regional lymph nodes showed follicular hyperplasia and sinus histiocytosis; indicative of enhanced immunogenicity.
These changes are worthy of note and are of minor significance.

Lungs/ Trachea /Bronchi

The right and left lungs are displayed completely and have no lesions of significance.
No Lesions of significance.

Thymus

The gland has a well developed cortex and medulla.
No lesions of significance.

Heart / major vessels / valves

The section apparently has left ventricular muscle and a portion of the atria-ventricular valve with a myxomatous nodular attached to it. Such an abnormality in time will lead to a cardiac incompetence.

Skin

Skin with regularly formed dermal appendages covered the upper hypodermis and in turn the thin muscular layer (Panniculus carnosus).
No lesions of significance.

Eyes / Harderian glands

Both eyes and their Harderian glands were sectioned and revealed.
The component elements of these structures are normal and have no lesions of significance.

Skeletal Tissue (appendicular skeleton)/ synovial joint/ skeletal muscle

Sections display the femur, tibia, tarsus, metatarsus and the styfle joint with the appropriate structures. The striated muscle of the leg is normal and covered by normal skin. The marrow in the tibia and femur is active and throughout the marrow are scattered megakaryocytes.
No lesions of significance.

Head

Examination of 7 levels reveal the following:
Pituitary gland, nasal cavity, oral cavity with teeth, outer and middle ear with ossicles, inner ear with osseous labyrinth,
cochlear, organ of Corti, scala vestibular and Scala Tympani Tectorial membrane and associated cells, sensory epithelium with otoliths in cupula, stria Vascularis and no apparent reduction in spiral ganglion cells at several levels.
No lesions of significance.

B-3 (#B124)

Ovary/ Fallopian tubes

Several follicles at different stages of development as well as corpora lutea occured in both ovaries. The fallopian tubes together with the ovaries have no features of significance.

Uterus / Vagina

The features of uterus and vagina are those observed in dioestrus. There are no lesions of significance.

Mammary glands

Typical mammary fat pad with developing lactiferous glands. No lesions of significance.

Urinary Bladder

No lesions of significance.

Liver /Gall Bladder

This major organ and its gall bladder display no features of significance.

Oesophagus

Longitudinal sections from the thoracic portion of the oesophagus display no lesions of significance.

Stomach

The section displays the glandular and non-glandular portions of the stomach. There are no lesions of significance.

Small Intestine

Normal with no lesions of significance. Lymph nodes in the wall of the small intestine displays nodular hyperplasia indicative of immune reactivity. No lesions of significance.

Large Intestine /Caecum

No lesions of significance.

Mesenteric Lymph Node

Mild sinus histiocytosis. No lesions of significance.

Spleen

Follicular pattern formed with white and red pulp cell types.
No lesions of significance.

Pancreas

The exocrine and endocrine portions of this gland are easily recognized and there are no lesions of significance.

Kidneys

Sections of both kidneys reveal no lesions of significance in the cortex, medulla and renal papilla that enters the renal pelvis. The tubular structures that course from the medulla into the renal papilla do not display any feature of significance and their lumens do not contain any material e.g. proteinaceous or cellular material.

Adrenal glands

Both glands were present and show no lesions of significance.

Thyroid glands

Colloid secreting thyroid gland on both sides of the trachea and in one there was a portion of a normal parathyroid gland present.
No lesions of significance.

Salivary glands and associated Lymph nodes

Parotid, submandibular and sublingual were present the accompanying regional lymph nodes displayed a minor reactivity. No significant lesions.

Lungs/ Trachea /Bronchi

No lesions of significance.

Thymus

The cortex and medulla of the thymus were present. A small cyst was present; this cyst was of little significance.
Please see information/comment re thymic cysts in the preliminary report.

Heart / major vessels / valves

Left atrium and ventricle with a portion of the aorta were identified as was a small amount of the right atrium and ventricle. No significant lesions were observed.

Skin

Sections display the epidermis, dermis, dermal appendages as well as the hypodermis and the panniculus carnosus. No lesions of significance were encountered.

Eyes / Harderian glands

The eyes and the Harderian glands were devoid of significant lesions.

Skeletal Tissue (appendicular skeleton)/ synovial joint/ skeletal muscle

The appendicular skeleton and the styfle joint appeared without significant lesions. The striated skeletal muscle appeared without a significant lesion.

Bone marrow smear

Cells of erythroid and myeloid series filled the tibial and femoral cavities without lesions of significance. Megakaryocytes were obvious in the marrow.
No lesions of significance.

Tail

Longitudinal and cross sections display the components expected and no lesion of significance was observed.

Head

Sections from 5 levels display the features as described in the preliminary report. Please note that a portion of the inner ear was not present in the pertinent section. However, that displayed in the section seemed without significant lesions.

C-1 (#C27)

Peripheral Blood Smear

Mainly red blood cells with a scattering of neutrophils, small lymphocytes and platelet clumps.
No lesion of significance.

Bone marrow smear

Cells were readily identified from the erythroid and myeloid series as well as the lymphocytic series and multinucleated megakaryocytes were occasionally observed.
No lesion of significance.

Ovary/ Fallopian tubes

Sections from both ovaries display follicles and corpora lutea at different development stages.
Unremarkable fallopian tubes.
No lesions of significance.

Uterus / Vagina

The architecture of the uterus and the low numbers of neutrophils and lymphocytes in the myometrium and the endometrial glands is consistent with the migration of these cells during a stage of oestrus. The loss of much of the luminal vaginal keratinous layer by neutrophils is consistent with the mouse being in metoestrus.
No lesions of significance.

Mammary glands

Typical mammary fat pad with regular spaced scattering of lactiferous ducts covered by skin.
No lesions of significance.

Urinary Bladder

Collapsed bladder with an out-flowing urethra. The Tunica muscularis appears normal.
No lesions of significance.

Liver /Gall Bladder

The liver displays a few foci of neutrophils and lymphocytes scattered throughout the parenchyma. The gall bladder does not display any lesion of significance.
The few parenchymal foci of inflammatory cells are indicative of ascending bacterial occurrence; such a feature is common in murine livers and is not considered consistent with lesions of significance.
No lesions of significance.

Oesophagus

A cross section of the thoracic oesophagus was examined and does not display any lesion of significance.
No lesions of significance.

Stomach

Sections display glandular and non-glandular regions of the stomach separated by the limiting ridge.
No lesions of significance.

Small Intestine

Regions from the duodenum, jejunum and ileum did not display any lesion.
Lymph nodes in the wall of the small intestine displays nodular hyperplasia with germinal centers indicative of immune reactivity.
No lesions of significance.

Large Intestine /Caecum

The mucosal folds and the mucosa of these parts of the large intestine do not show any feature of abnormality.
No lesions of significance.

Mesenteric Lymph Node

Sections reveal mild nodular hyperplasia and sinus histiocytosis.
These features are indicative of increase immunogenicity; a constant occurrence in this node.
No lesions of significance.

Spleen

The splenic parenchyma displays a follicular pattern with lymphocytes forming the follicles while the red pulp that separates the follicles has a high density of red blood cells.
A mild extramedullary haematopoiesis is apparent; such occurs regularly in mice.
No lesions of significance.

Pancreas

The gland has an exocrine and endocrine section (islets of Langerhans).
No features of abnormality or lesions are microscopically apparent.
No lesions of significance.

Kidneys

The sections from both kidneys appear to be without any microscopic lesions.
Both kidneys were sectioned but the section of one kidney does not display the renal papilla or pelvis.
However most lesions in the pelvis and /or papilla result in a flow or filter alteration such is not the case in this kidney; hence possible lesions are unlikely. Regardless if you suspect a lesion in the medulla then levels should be cut to ensure that there are no microscopic lesions.
The other kidney displays a cortex that merges into the medulla which flows into a renal papilla that occupies a portion of the renal pelvis.
The tubular portions of each nephron that reside in the medulla and also within the renal papilla appear patent and do not contain any obstructive proteinaceous material in their lumens nor any cellular response within the sectioned interstitium of cortex, medulla and renal papilla.
No lesions of significance.

Comments:

If suspecting a lesion within the medulla (e.g. urinary concentration) light microscopic appraisal does not reveal any micromorphological lesion. Therefore the research team should appraise the morphology using electron microscopy. Alternatively Urinalysis should be undertaken.

Adrenal glands

Both glands were sectioned revealing cortex and medulla in one adrenal and cortex in the other no lesions were observed.
No lesions of significance.

Thyroid glands

Sections reveal a colloid secreting follicular parenchyma of a lateral margin of the thyroid gland.
The follicles are lined by cuboidal epithelium.
Furthermore, there is a small area of parathyroid gland embedded in the thyroid gland.
No lesions of significance.

Salivary glands and associated Lymph nodes

Sections display the parotid, submandibular and sublingual glands. No features of abnormality.
The regional node shows a mild sinus histiocytosis (part of an innate immunogenicity).
No lesions of significance.

Lungs/ Trachea /Bronchi

Sections reveal most of the right and left lungs not displaying any abnormality. A portion of the cardiac atria and at least, one atria-ventricular valve (see Heart / major vessels / valves).
No lesions of significance.

Thymus

Sections show a cortex and medulla with cellular features expected in these regions.
No lesions of significance.

Heart / major vessels / valves

A microscopic thickening of one cardiac valve on its free margin with the connective matrix staining blue (H &E stained) as for a myxomatous matrix. Similar changes were observed in one of the controls mice.
The myocardial ventricular muscle fibres from left and right ventricles display no lesions of significance.
No lesions of significance.

Skin

The epidermis, dermis and dermal appendages e.g. follicles, sebaceous glands have normal features. The upper hypodermis and Panniculus carnosus do not have and features of abnormality.
No lesions of significance.

Eyes / Harderian glands

Sections from each eye display normal retina, lens, cornea, sclera, ciliary body ,sclera, iris and choroid.
The Harderian glands displaying a tubuloalveolar pattern, were both sampled.
No lesions of significance.

Skeletal Tissue (appendicular skeleton)/ synovial joint/ skeletal muscle

Sections revealed the distal end of the femur and the proximal half of the tibia, separated by the synovial joint in which a patella and patella ligament are readily observed.
Active marrow occurred in the marrow cavity of femur and tibia. Megakaryocytes are observed.

Head

Four levels were taken from this structure revealing no lesions in the nasal and oral cavities.
Teeth in the appropriate sites were readily observed and the pituitary gland including Pars intermedia, pars distalis and pars nervosa.
The outer and middle ear with an intact tympanic membrane and the ossicles were present.
The inner ear including the bony labyrinth, organ of Corti, cochlear with tectorial membrane and the hair cells. Scala cavities identified and separated by the cochlear duct.
There does not appear to be a reduction in the spiral ganglion cells at different levels (slide No54371).
Sections passed through an ampulla displaying a crista and a macula with sensory epithelium.
These areas have sensory cells some of which have hairs which project into a gelatinous material cap (cupula) which also contain otoliths.
No lesions of significance.

C-2 (#C20)

Peripheral Blood Smear

Mainly red blood cells with a scattering of neutrophils, small lymphocytes and platelet clumps.
No lesions of significance.

Bone marrow smear

Cells are readily identified from the erythroid and myeloid series as well as the lymphocytic series. Multinucleated megakaryocytes are occasionally discernible.
No lesions of significance.

Ovary/ Fallopian tubes

Sections from both ovaries display follicles and corpora lutea at different development stages.
Unremarkable fallopian tubes.
No lesions of significance.

Uterus / Vagina

The architecture of the uterus with two horns and a body that merges into the vagina. Low numbers of neutrophils and lymphocytes occurred in the myometrium and occasionally in the endometrial glands. Such a pattern is consistent with the migration of a few cells during a stage of oestrus. A prominent layer of mucified cells above a line of discrete cornification lines the vaginal luminal surface such a pattern is consistent with the mouse being in proestrus.
No lesions of significance.

Mammary glands

Typical mammary fat pad with regular spaced scattering of lactiferous ducts covered by skin.
No lesions of significance.

Urinary Bladder

Collapsed bladder lined by urothelium. The Tunica muscularis appeared normal
No lesions of significance.

Liver /Gall Bladder

In several portal triads in the liver there isa mild accumulation of small lymphocytes, otherwise there are no lesions of significance in the liver. The gall bladder does not display any lesion of significance.
No lesions of significance.

Oesophagus

Two long longitudinal sections of the thoracic oesophagus, located in the mediastinum, are examined and do not display any lesions of significance.
No lesions of significance.

Stomach

Sections display portions of the glandular and non-glandular regions of the stomach.
No lesions of significance.

Small Intestine

Regions from the duodenum, jejunum and ileum do not display any lesions.
Lymph nodes in the wall of the small intestine display nodular hyperplasia with germinal centers indicative of immune reactivity; the latter feature more frequently occurrs in the Peyer's patches of the ileum.
No lesions of significance.

Large Intestine /Caecum

The mucosal folds and the mucosa of these parts of the large intestine do not show any feature of abnormality. Prominent and reactive immunogenic lymphoid nodules.
No lesions of significance.

Mesenteric Lymph Node

Sections reveal mild nodular hyperplasia and moderate sinus histiocytosis.
These features are indicative of increase immunogenicity; a constant occurrence in this node.
No lesions of significance.

Spleen

The splenic parenchyma displays a follicular pattern with lymphocytes forming the follicles while the red pulp that separates the follicles has a high density of red blood cells with a sprinkling of nucleated cells.
A mild extramedullary haematopoiesis is apparent; such occurs regularly in mice.
No lesions of significance.

Pancreas

The gland has an exocrine and endocrine section (islets of Langerhans).
No features of abnormality or lesions are microscopically apparent.
No lesions of significance.

Kidneys

The sections from both kidneys appear to be without any microscopic lesions.
Both kidneys are sectioned but the section in the main does not display the renal papilla or pelvis although the medulla was viewed centrally. There do not appear to be any lesions in the kidney nor in the medulla. However, to exclude any micromorphological lesions in the medulla, renal papilla and renal pelvis deeper sections need to be prepared if medullary lesions are suspected regardless of whether a microscopic lesion has been identified.
The tubular portions of each nephron that reside in the medulla should be inspected and also within the renal papilla ensuring that the lumens of the nephron tubules appear patent and do not contain any obstructive proteinaceous material in their lumens. Moreover, check for any cellular response within the sectioned interstitium of cortex, medulla and renal papilla.
No microscopic lesions of significance were observed in the sections presented in this case.

Comments:

If suspecting a lesion within the medulla (e.g. urinary concentration) and light microscopic appraisal does not reveal any micromorphological lesion, suggest the research team should appraise the morphology using electron microscopy. Alternatively urinalysis should be undertaken.

Adrenal glands

Both glands were sectioned revealing cortex and medulla in one adrenal and cortex in the other; no lesions were observed.
No lesions of significance.

Thyroid glands

A tiny cluster of colloid secreting follicles in slide 54219 adjacent to the trachea.
No lesions of significance.

Salivary glands and associated Lymph nodes

Section displays the parotid, submandibular and sublingual glands. No features of abnormality.
The regional lymph nodes show a mild nodular hyperplasia and sinus histiocytosis (part of an innate immunogenicity).
No lesions of significance.

Lungs/ Trachea /Bronchi

Section reveals most of the right and left lungs not displaying any abnormality.
The entire length of the trachea is included and near the proximal exit of the major airway is a large aggregation of mucin secreting glands.
A cross section of a rib and some striated muscle is encountered.
No lesions of significance.

Thymus

Sections show a cortex and medulla with cellular features in these regions as normal.
No lesions of significance.

Heart / major vessels / valves

The right and left atria and corresponding ventricles with atria-ventricle valve from the right side of the heart
No lesions of significance.

Skin

The epidermis, dermis and dermal appendages e.g. follicles, sebaceous glands have normal features. The upper hypodermis and Panniculus carnosus do not have and features of abnormality.
No lesions of significance.

Eyes / Harderian glands

Sections from each eye displays normal retina, lens, cornea, sclera, ciliary body ,sclera, iris and choroid.
The Harderian glands displaying a branched tubuloalveolar pattern were both sampled.
No lesions of significance.

Skeletal Tissue (appendicular skeleton)/ synovial joint/ skeletal muscle

Sections reveal the distal end of the femur and the entire tibia and tarsal bones. The synovial joint with relatively normal articular cartilage has a normal patella and patella ligament.
Active marrow occurred in the marrow cavity of femur and tibia. Megakaryocytes are observed.
The skeletal striated muscle has regular and consistent sized fibres and peripheral nuclei.
No lesions of significance.

Skin

Section reveal epidermis, dermis and upper hypodermis as well as Panniculus carnosus regions having a normal microanatomical structure. The dermal appendages are regularly spaced and the follicles and glands do not display any feature or lesion of significance.
No lesions of significance.

Head

Seven levels were taken from the head revealing no lesions of significance in the nasal and oral cavities. Teeth seem to be in the appropriate sites for the mandible and maxillary facial bones.
The pituitary gland including Pars intermedia, pars distalis and pars nervosa are present.
The outer and middle ear with intact tympanic membranes and residing ossicles are observed.
The inner ear including the bony labyrinth, cochlear with organ of Corti(spiral organ) the latter included tectorial membrane, structural rods and hair cells. Scala vestibuli and scala tympani cavities are separated by the cochlear duct.
There does not appear to be a reduction in the spiral ganglion cells at different levels (slide No55925).
Sections pass through an ampulla displaying a crista and a macula with sensory epithelium.
These areas have sensory cells some of which have hairs that project into a gelatinous cap (cupula) in which were otoliths.
No lesions of significance.

C-3 (#C29)

Peripheral Blood Smear

Red blood cells are the main cell in this smear together with a very light scattering of lymphocytes and fewer neutrophils. Additionally clumps and small aggregates of platelets occurr.
No lesions or abnormalities were encountered.

Bone marrow smear

Representative cells from the myeloid and erythroid series as well as lymphocytic series are readily discerned. Occasional unremarkable megakaryocytes are encountered.
No lesions or abnormalities were encountered.

Ovary/ Fallopian tubes

The ovaries appeared with out abnormality. Both ovaries display follicles at various development stages as well as several corpora lutea. The Fallopian tubes appear unremarkable.
No lesions or abnormalities were encountered.

Uterus / Vagina

The horns and body of the uterus as well as the vagina display cellular activity indicative of an active stage of oestrus. In light of the mucoid cellular layer over a keratinous layer formed on the luminal surface of the vagina the mouse is at stage of proestrus.

Mammary glands

Sections from the mammary fat pad display regularly spaced active developing lactiferous ducts.
No lesions or abnormalities were encountered.

Urinary Bladder

Sections nominated in the primary report display a collapsed bladder with normal urothelium and tunica muscularis.
No lesions or abnormalities were encountered.

Liver /Gall Bladder

The liver reveals a normal and unremarkable structured organ with a small portion of the gallbladder attached.
Adjoining part of the liver was a muscle enclosed tangential portion of the oesophagus.
No lesions or abnormalities were encountered.

Oesophagus

In the mediastinum (slide 54222) is a cross section of the oesophagus.
No lesions or abnormalities were encountered.

Stomach

The section displays mainly the glandular portion of the stomach as well as some of the non-glandular part of the gastric wall, separating these regions was the limiting ridge.
No lesions or abnormalities were encountered.

Small Intestine

The section includs the duodenum, jejunum and ileum with typically arranged villi for each of these regions of the small intestine. Additionally, there are nodular islands of lymphoid tissue scattered in the intestinal wall and in the ileum these nodular islands are nominated as Peyer’s patches. All the lymphoid islands show varying degrees of reactive immunogenicity; an expected phenomena.
No lesions or abnormalities were encountered.

Large Intestine /Caecum

Typical mucosal folds and micromorphology for the large bowel with an occasional nodular lymphoid island in the intestinal wall displaying mild features of immunogenicity.
No lesions or abnormalities were encountered.

Mesenteric Lymph Node

A mild follicular hyperplasia and sinus histiocytosis such features are consistent with a mild degree of immunogenicity.
No lesions or abnormalities were encountered.

Spleen

Sections display a follicular pattern composed of nodules composed mainly of lymphocytes, separated from other nearby nodular islands by red pulp where the dominant cells are erythrocytes. Additionally there is a mild extramedullary haematopoiesis.
No lesions or abnormalities were encountered.

Pancreas

Sections reveal exocrine glandular tissue throughout which are scattered islets of Langerhans (endocrine glands).
No lesions or abnormalities were encountered.

Kidneys

Sections from both kidneys reveal a cortex, medulla and a portion of the renal papilla within the external portion of the renal pelvis. There are no lesions of significance in either kidney and their microanatomy does not indicate any feature indicative of an acute or chronic pending lesion/lesions.
However, to exclude any micromorphological lesions in the cortex, medulla, renal papilla and renal pelvis sections at levels could be prepared if medullary lesions are suspected regardless of whether a microscopic lesion has been identified. The tubular portions of each nephron that reside in the medulla should be inspected and also within the renal papilla ensuring that the lumens of the nephron tubules appear patent and do not contain any obstructive proteinaceous material in their lumens. Moreover, check for any cellular response within the sectioned interstitium of cortex, medulla and renal papilla.
No microscopic lesions of significance were observed in the sections presented to me in this case.

Comments:

If suspecting a lesion within the medulla (e.g. urinary concentration) and light microscopic appraisal does not reveal any micromorphological lesion suggest the research team should appraise the micro-morphology using electron microscopy. Alternatively urinalysis should be undertaken.

Adrenal glands

Both adrenal glands have been sectioned and display unremarkable micromorphology for cortex and medulla of each gland.
No lesions of significance.

Thyroid glands

Sections from the head at level 6 display an active colloid secreting follicles lined by cuboidal epithelium on both sides of the larynx.

Salivary glands and associated Lymph nodes

Section displays parotid, submandibular and sublingual glands. Regional lymph nodes show a mild degree of immunogenicity featuring as mild sinus histiocytosis and follicular hyperplasia.
No lesions of significance.

Lungs/ Trachea /Bronchi

Section includes the left and right lungs with appropriate airways, blood vessels and alveolar parenchyma.
No lesions of significance.

Thymus

A small mucoid cyst occurs in the cortico-medullary junction. The cyst is isolated by an outer band of connective tissue and appears to be a remnant of the thymopharyngeal duct. The cyst measures less than 100 microns in diameter and would not be of significance.
No lesions of significance.

Heart / major vessels / valves

Sections (54231 and 54222) show components of the heart viz left and right atria and ventricles and large vessels arising from the heart base.
Cardiac muscle fibres of the myocardium have a centrally located nuclei.
No lesions of significance.

Skin

The macroscopic arrangement of the skin in location and components appear unremarkable. The epidermis as described in the preliminary report as "mild multifocal epidermal hyperplasia with a papillated appearance in some areas" does appear to be so. However, on examination of strips of skin overlying the mammary gland from this mouse the epidermis appeared to be as normal. Furthermore, examination of cutaneous sections from the control animals, supplied the epidermis has a scattered somewhat similarity to that observed in this experimental mouse #C3. Therefore leaving aside the possibility of a solitary fibropapilloma, the mild multifocal epidermal hyperplasia in my opinion is artefactual, possibly resulting from the fixation technique.
No lesions of significance.

Eyes / Harderian glands

The retina, choroid, cornea, iris ,lens, sclera are readily observed and these regions of the eye seem unremarkable.
The tubuloalveolar glandular pattern of the Harderian glands appears as normal.
No lesions of significance.

Skeletal Tissue (appendicular skeleton)/ synovial joint/ skeletal muscle

Section includes distal end of the femur and most of the tibia, these bones were separated by the synovial joint. The joint appears normal and section highlights the presence of the patella and the patella ligament. Some of the bones of the tarsus and metatarsus are included in the section. The femur and tibia contain active bone marrow. Growth plates of the tibia and femur display features consistent with pending closure.
The skeletal muscle of the limb appear normal and its muscle fibres are consistently sized with nuclei located peripherally.
No lesions of significance.

Head

Nine levels were prepared from the head and reveal the nasal and oral cavities with their teeth and tongue present. In slide 54379 the pituitary gland Pars intermedia, pars distalis and pars nervosa) and the trigeminal nerve are displayed. The outer and middle ear cavities are clearly shown as is the tympanic membrane and indwelling ossicles.

Components of the inner ear including the bony labyrinth, organ of Corti (spiral organ).
Scala tympani and Scala vestibuli separated by cochlear duct are readily observed. The organ of Corti (spiral organ) with Hair cells and rods covered by tectorial membrane appear without lesion.
Moreover, the cochlear nerve and the spiral ganglion cells are observed at several levels with no apparent reduction in the number of these cells at different levels.
Sensory epithelium of the macula and the ampulla including the crista are encountered and in these sensory regions the gelatinous cupula that covers the specialized cells frequently with otoliths are present.

No lesions of significance.

Summary

The histopathology from each organ was compared to the same murine tissues recorded in the literature as well as comparing to sampled tissue from the control mice (Control 1:Control 2:Control 3).
The animals presented in this case bear no lesions of significance.

Worth noting in animal B-2 (#B133), the atria-ventricular valve with a myxomatous nodule
Worth noting in Group A, possible myxomatous thickening of the connective tissue of the heart valves

Professor Rolfe Howlett
R&A Pathology Services, NSW

8th July, 2016

ADDENDUM
Supplementary report on three mice displaying possible cardiac lesions APN16/016 Group A. In all cases 5 further levels were cut and examined (6 levels in total).
Valvular lesions displayed suggest that with age complications may arise.

Mouse A-1(#A137):
Further levels reveal definitive lesion and its complication.
Levels 2 and 3
Right atrium, right atria-ventricular valve and a recently formed thrombus in the right ventricle extending through the valvular opening. One leaflet of the atria-ventricular valve shows a distortion nodularity of the last 450microns measured from the free margin.
On the free valvular margin is a nodular mesenchymal lesion of some 80 microns in diameter.
Levels 5 &6
Aortic valve exposed to display mild myxomatous matrix thickening one leaflet.
The left atria-ventricular valve shows a mild myxomatous thickening along the free margin.

Mouse A-3(#A138)
In level 4 one leaflet of the left atria-ventricular is displayed to allow observation of a small nodular thickening near to the free margin of a valve, appearing not to be on the free margin.
The nodular thickening extends from margin to margin with readily identified endothelial cells on either margin.
The small nodular thickening is a consequence of an increased amount of intercellular myxomatous matrix .

Mouse A2(#A140)
The six levels display normal right and left atria and right and left ventricle. Additionally the aorta and one aortic valvular leaflet appear normal.
A thrombotic like mass in the right ventricle such may occur after euthanasia.
The A/V valves from their free margins for about 20 microns the intercellular matrix is lightly basophilic indicative of a minor alteration in the chemistry of the matrices see "Pathology of the Mouse" edited by Robert R Maronpot, page362, which states that typically in mice there is a pale myxomatous appearance to the connective tissue of the aortic valve when stained with H & E.

Professor Rolfe Howlett
R&A Pathology Services, NSW
26th July, 2016

Supplementary Neuropathology Report

Control-1 (control)

H&E and LFB stained brain sections were examined.
Representative H&E stained sections of spinal cord were examined.
Slides were used as histological controls

Control-2 (control)

H&E and LFB stained brain sections were examined.
Representative H&E stained sections of spinal cord were examined.
Slides were used as histological controls

Control-3 (control)

H&E and LFB stained brain sections were examined.
Representative H&E stained sections of spinal cord were examined.
Slides were used as histological controls

A-1 (#A137)

H&E and LFB stained brain sections were examined.
Representative H&E stained sections of spinal cord were examined.
Sections of brain and spinal cord show no significant neuropathology.

A-2 (#A140)

H&E and LFB stained brain sections were examined.
Representative H&E stained sections of spinal cord were examined.
Sections of brain and spinal cord show no significant neuropathology.

A-3 (#A138)

H&E and LFB stained brain sections were examined.
Representative H&E stained sections of spinal cord were examined.
Sections of brain and spinal cord show no significant neuropathology.

B-1 (#B126)

H&E and LFB stained brain sections were examined.
Representative H&E stained sections of spinal cord were examined.
Sections of brain and spinal cord show no significant neuropathology.

B-2 (#B133)

H&E and LFB stained brain sections were examined.
Representative H&E stained sections of spinal cord were examined.
Sections of brain and spinal cord show no significant neuropathology.

B-3 (#B124)

H&E and LFB stained brain sections were examined.
Representative H&E stained sections of spinal cord were examined.
Sections of brain and spinal cord show no significant neuropathology.

C-1 (#C27)

H&E and LFB stained brain sections were examined.
Representative H&E stained sections of spinal cord were examined.
Sections of brain and spinal cord show no significant neuropathology.

C-2 (#C20)

H&E and LFB stained brain sections were examined.
Representative H&E stained sections of spinal cord were examined.
Sections of brain and spinal cord show no significant neuropathology.

C-3 (#C29)

H&E and LFB stained brain sections were examined.
Representative H&E stained sections of spinal cord were examined.
Sections of brain and spinal cord show no significant neuropathology.

Summary

Sections of brain and spinal cord show no significant neuropathology.

Professor Catriona A McLean
BSc, MBBS, FRCPA, MD, FFSc (RCPA)

Head Anatomical Pathology, Alfred Health
14th July, 2016

The Australian Phenomics Network advises all research groups that images or results obtained through the services offered by the APN are to be acknowledged in resultant publications. Example acknowledgement: "This study utilised the Australian Phenomics Network Histopathology and Organ Pathology Service, University of Melbourne."

Authorised by Tina Cardamone, APN Histopathology and Organ Pathology Node manager.

Supplementary File 2 Histopathology Report (additional hearts)

Contacts:
Tina Cardamone, Manager APN (Melbourne)
Professor Janet Keast, Head APN (Melbourne)

Australian Phenomics Network
Histopathology and Organ Pathology
The University of Melbourne
Department of Anatomy and Neuroscience
Grattan Street, PARKVILLE, VIC 3010
T: +61 3 83448044
apn-info@unimelb.edu.au

9.1 Histopathology Report

**Case Number**	APN17/006 Monash University-HIMR (Justin St John & Jacqueline Johnson)
**Registration Date**	Thu 16/02/2017
**Animal Details**	A148<br>DOB: null, Female, nullg<br><br>A150<br>DOB: null, Female, nullg<br><br>A151<br>DOB: null, Female, nullg<br><br>A152<br>DOB: null, Female, nullg
**DoD / Necropsy**	Necropsy not conducted at HOPS
**Death**	Unknown
**Origin**	Monash University-HIMR
**Treatment**	Treatment<br>Confirm heart valvular changes noted in B-2 (#B133) & Group A, case APN16/016
**Species / Breed / Strain**	C57BL6/JxCBA
**Animal Health Facility**	N/A<br>N/A
**Organs Examined**	Heart
**Macroscopic Observations**	Four fixed mouse hearts delivered to APN HOPS 16/2/2017

Microscopic Observations

A148

Macro Observations

Fixed mouse heart
9x5x4mm

Micro Observations

Typical micromorphological observed in cardiac muscle, chambers and vessels of the heart.
The cardiac muscle fibers demonstrated typical features including central nuclei, branching fibers and striations.

Heart valves - myxomatous stroma with varying amounts of collagen and melanin.

A150

Macro Observations

Fixed mouse heart
10x6x5mm

Micro Observations

Typical micromorphological observed in cardiac muscle, chambers and vessels of the heart.
The cardiac muscle fibers demonstrated typical features including central nuclei, branching fibers and striations.

Heart valves - myxomatous stroma with varying amounts of collagen and melanin.

A151

Macro Observations

Fixed mouse heart
10x6x5mm

Micro Observations

Typical micromorphological observed in cardiac muscle, chambers and vessels of the heart.
The cardiac muscle fibers demonstrated typical features including central nuclei, branching fibers and striations.
Possible mild valvular changes.
Pathology to confirm

A152

Macro Observations

Fixed mouse heart
8x6x5mm

Micro Observations

Typical micromorphological observed in cardiac muscle, chambers and vessels of the heart.
The cardiac muscle fibers demonstrated typical features including central nuclei, branching fibers and striations.
Possible mild valvular changes.
Pathology to confirm

Comment / Plan

Case APN17/006HIMR will be referred to Dr. John Finnie, SA Pathology for comment.

Tina Cardamone
3rd March, 2017

A148

A150

A151

A152

Summary

All heart samples had varying degrees of severity of mucoid degeneration of the valves.

Dr John Finnie
Senior Veterinary Pathologist
SA Pathology, Adelaide, S.A. 5000
Affiliate Associate Professor
School of Medicine, University of Adelaide
8th March, 2017

The Australian Phenomics Network advises all research groups that images or results obtained through the services offered by the APN are to be acknowledged in resultant publications. Example acknowledgement: "This study utilised the Australian Phenomics Network Histopathology and Organ Pathology Service, University of Melbourne."

Authorised by Tina Cardamone, APN Histopathology and Organ Pathology Node manager.

Supplementary File 3 Whole mitochondrial genome alignments for consensus sequences from six primordial follicles from third generation mitochondrial supplemented mice and three EPC aliquots used for mitochondrial supplementation. An asterisk indicates base changes on pages 67, 68 and 71.

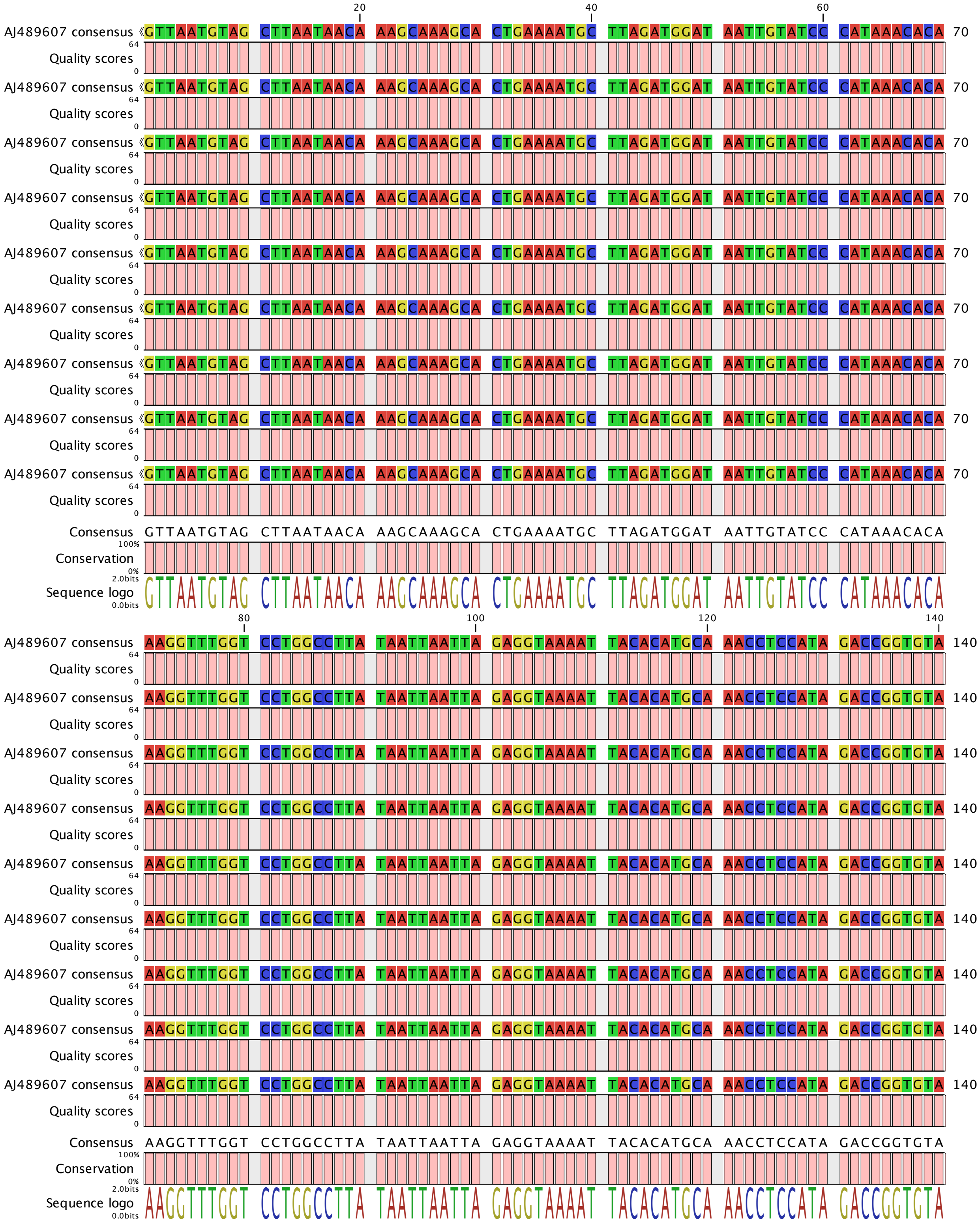
20
40
60
AJ489607 consensus GTTAATGTAG CTTAATAACA AAGCAAAGCA CTGAAAATGC TTAGATGGAT AATTGTATCC CATAAACACA 70
Quality scores
AJ489607 consensus GTTAATGTAG CTTAATAACA AAGCAAAGCA CTGAAAATGC TTAGATGGAT AATTGTATCC CATAAACACA 70
Quality scores
AJ489607 consensus GTTAATGTAG CTTAATAACA AAGCAAAGCA CTGAAAATGC TTAGATGGAT AATTGTATCC CATAAACACA 70
Quality scores
AJ489607 consensus GTTAATGTAG CTTAATAACA AAGCAAAGCA CTGAAAATGC TTAGATGGAT AATTGTATCC CATAAACACA 70
Quality scores
AJ489607 consensus GTTAATGTAG CTTAATAACA AAGCAAAGCA CTGAAAATGC TTAGATGGAT AATTGTATCC CATAAACACA 70
Quality scores
AJ489607 consensus GTTAATGTAG CTTAATAACA AAGCAAAGCA CTGAAAATGC TTAGATGGAT AATTGTATCC CATAAACACA 70
Quality scores
AJ489607 consensus GTTAATGTAG CTTAATAACA AAGCAAAGCA CTGAAAATGC TTAGATGGAT AATTGTATCC CATAAACACA 70
Quality scores
AJ489607 consensus GTTAATGTAG CTTAATAACA AAGCAAAGCA CTGAAAATGC TTAGATGGAT AATTGTATCC CATAAACACA 70
Quality scores
AJ489607 consensus GTTAATGTAG CTTAATAACA AAGCAAAGCA CTGAAAATGC TTAGATGGAT AATTGTATCC CATAAACACA 70
Quality scores
Consensus GTTAATGTAG CTTAATAACA AAGCAAAGCA CTGAAAATGC TTAGATGGAT AATTGTATCC CATAAACACA
Conservation
Sequence logo
80
100
120
140
AJ489607 consensus AAGGTTTGGT CCTGGCCTTA TAATTAATTA GAGGTAAAAT TACACATGCA AACCTCCATA GACCGGTGTA 140
Quality scores
AJ489607 consensus AAGGTTTGGT CCTGGCCTTA TAATTAATTA GAGGTAAAAT TACACATGCA AACCTCCATA GACCGGTGTA 140
Quality scores
AJ489607 consensus AAGGTTTGGT CCTGGCCTTA TAATTAATTA GAGGTAAAAT TACACATGCA AACCTCCATA GACCGGTGTA 140
Quality scores
AJ489607 consensus AAGGTTTGGT CCTGGCCTTA TAATTAATTA GAGGTAAAAT TACACATGCA AACCTCCATA GACCGGTGTA 140
Quality scores
AJ489607 consensus AAGGTTTGGT CCTGGCCTTA TAATTAATTA GAGGTAAAAT TACACATGCA AACCTCCATA GACCGGTGTA 140
Quality scores
AJ489607 consensus AAGGTTTGGT CCTGGCCTTA TAATTAATTA GAGGTAAAAT TACACATGCA AACCTCCATA GACCGGTGTA 140
Quality scores
AJ489607 consensus AAGGTTTGGT CCTGGCCTTA TAATTAATTA GAGGTAAAAT TACACATGCA AACCTCCATA GACCGGTGTA 140
Quality scores
AJ489607 consensus AAGGTTTGGT CCTGGCCTTA TAATTAATTA GAGGTAAAAT TACACATGCA AACCTCCATA GACCGGTGTA 140
Quality scores
AJ489607 consensus AAGGTTTGGT CCTGGCCTTA TAATTAATTA GAGGTAAAAT TACACATGCA AACCTCCATA GACCGGTGTA 140
Quality scores
Consensus AAGGTTTGGT CCTGGCCTTA TAATTAATTA GAGGTAAAAT TACACATGCA AACCTCCATA GACCGGTGTA
Conservation
Sequence logo

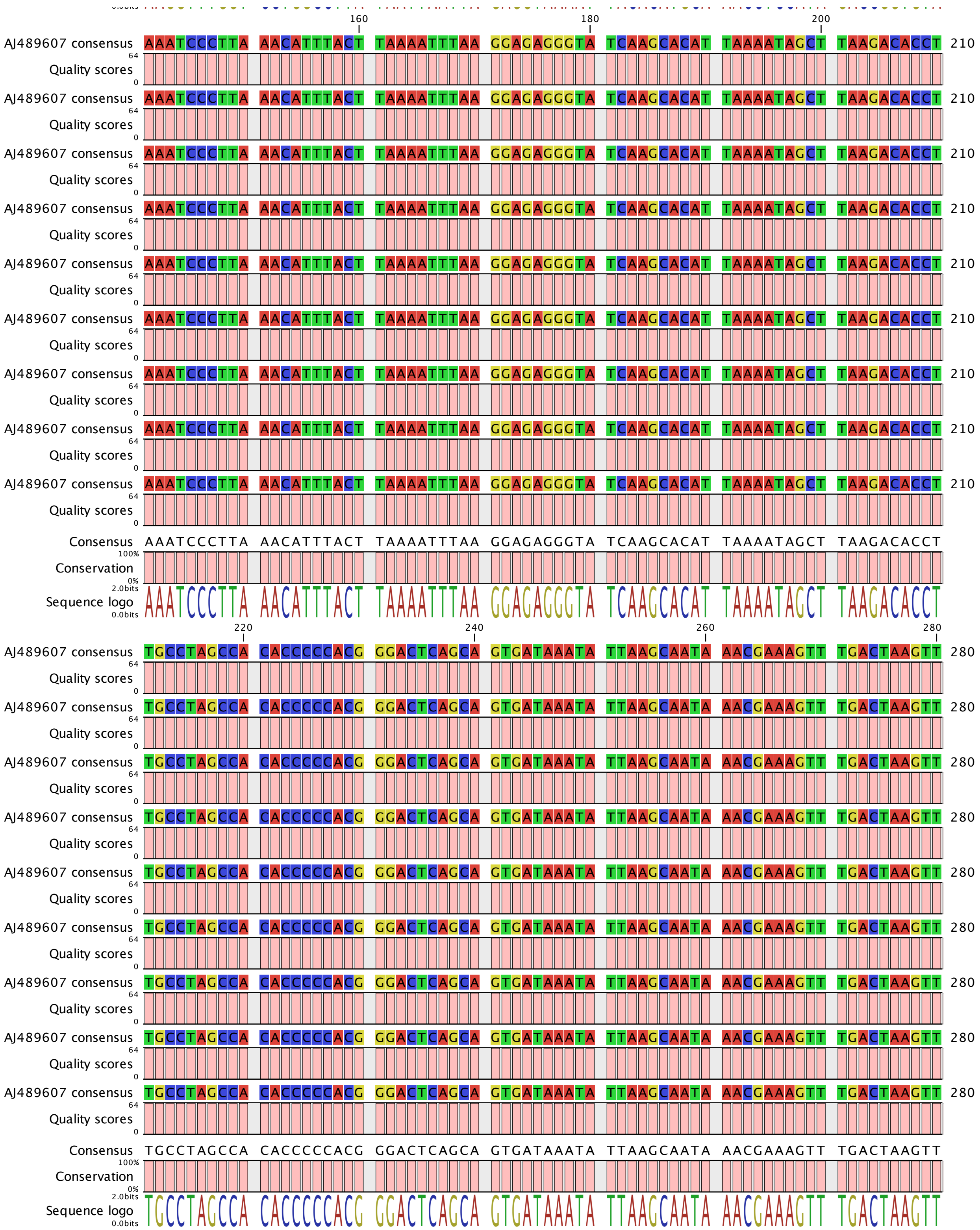
160
180
200
AJ489607 consensus AAATCCCTTA AACATTTACT TAAAATTTAA GGAGAGGGTA TCAAGCACAT TAAAATAGCT TAAGACACCT 210
Quality scores
Consensus AAATCCCTTA AACATTTACT TAAAATTTAA GGAGAGGGTA TCAAGCACAT TAAAATAGCT TAAGACACCT
Conservation
Sequence logo
220
240
260
280
AJ489607 consensus TGCCTAGCCA CACCCCCACG GGACTCAGCA GTGATAAATA TTAAGCAATA AACGAAAGTT TGACTAAGTT 280
Quality scores
Consensus TGCCTAGCCA CACCCCCACG GGACTCAGCA GTGATAAATA TTAAGCAATA AACGAAAGTT TGACTAAGTT
Conservation
Sequence logo

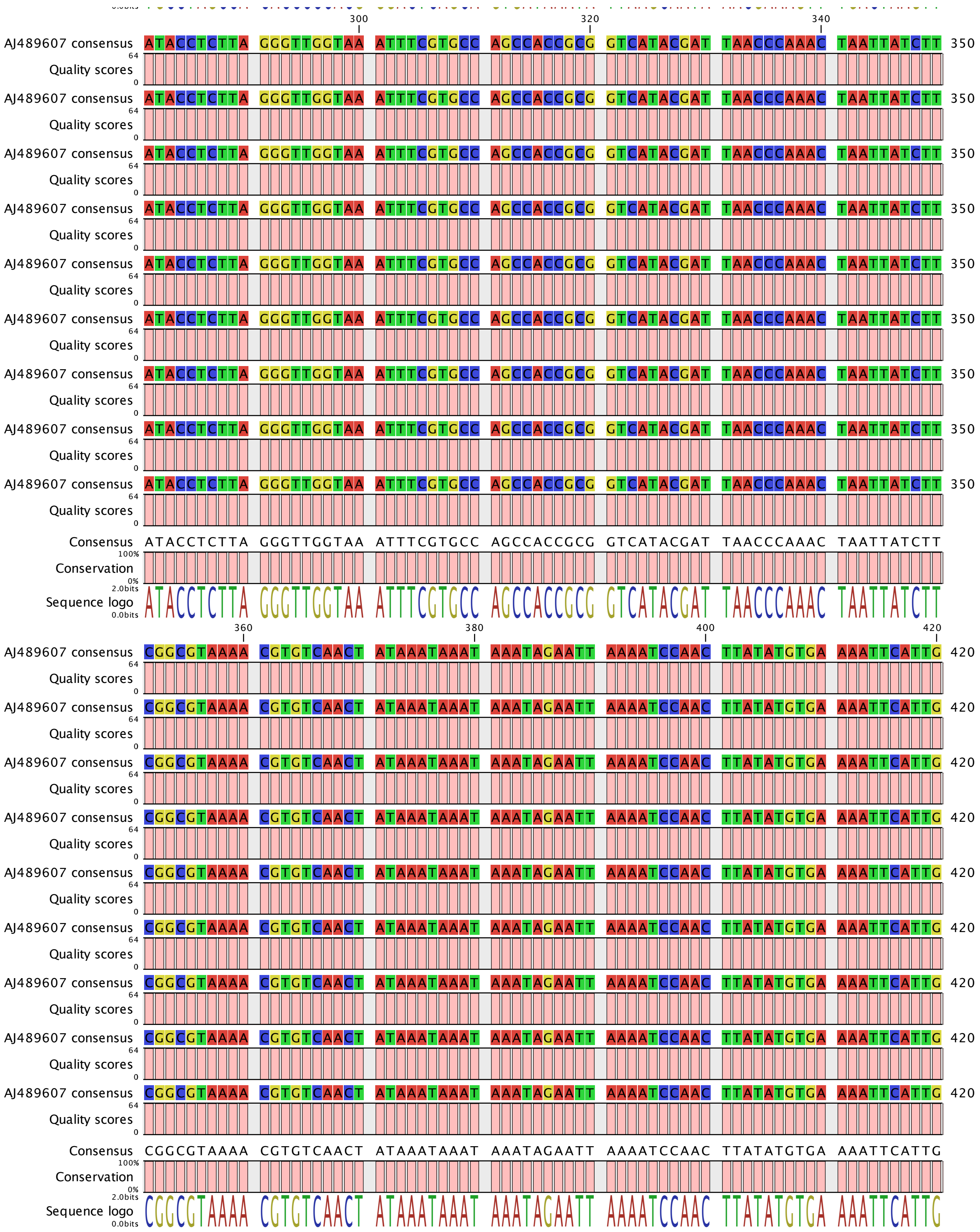
300
320
340
AJ489607 consensus ATACCTCTTA GGGTTGGTAA ATTTCGTGCC AGCCACCGCG GTCATACGAT TAACCCAAAC TAATTATCTT 350
Quality scores 64 0
AJ489607 consensus ATACCTCTTA GGGTTGGTAA ATTTCGTGCC AGCCACCGCG GTCATACGAT TAACCCAAAC TAATTATCTT 350
Quality scores 64 0
AJ489607 consensus ATACCTCTTA GGGTTGGTAA ATTTCGTGCC AGCCACCGCG GTCATACGAT TAACCCAAAC TAATTATCTT 350
Quality scores 64 0
AJ489607 consensus ATACCTCTTA GGGTTGGTAA ATTTCGTGCC AGCCACCGCG GTCATACGAT TAACCCAAAC TAATTATCTT 350
Quality scores 64 0
AJ489607 consensus ATACCTCTTA GGGTTGGTAA ATTTCGTGCC AGCCACCGCG GTCATACGAT TAACCCAAAC TAATTATCTT 350
Quality scores 64 0
AJ489607 consensus ATACCTCTTA GGGTTGGTAA ATTTCGTGCC AGCCACCGCG GTCATACGAT TAACCCAAAC TAATTATCTT 350
Quality scores 64 0
AJ489607 consensus ATACCTCTTA GGGTTGGTAA ATTTCGTGCC AGCCACCGCG GTCATACGAT TAACCCAAAC TAATTATCTT 350
Quality scores 64 0
AJ489607 consensus ATACCTCTTA GGGTTGGTAA ATTTCGTGCC AGCCACCGCG GTCATACGAT TAACCCAAAC TAATTATCTT 350
Quality scores 64 0
AJ489607 consensus ATACCTCTTA GGGTTGGTAA ATTTCGTGCC AGCCACCGCG GTCATACGAT TAACCCAAAC TAATTATCTT 350
Quality scores 64 0
Consensus ATACCTCTTA GGGTTGGTAA ATTTCGTGCC AGCCACCGCG GTCATACGAT TAACCCAAAC TAATTATCTT
Conservation 100% 0%
Sequence logo 2.0bits 0.0bits ATACCTCTTA GGGTTGGTAA ATTTCGTGCC AGCCACCGCG GTCATACGAT TAACCCAAAC TAATTATCTT
360
380
400
420
AJ489607 consensus CGGCGTAAAA CGTGTCAACT ATAAATAAAT AAATAGAATT AAAATCCAAC TTATATGTGA AAATTCATTG 420
Quality scores 64 0
AJ489607 consensus CGGCGTAAAA CGTGTCAACT ATAAATAAAT AAATAGAATT AAAATCCAAC TTATATGTGA AAATTCATTG 420
Quality scores 64 0
AJ489607 consensus CGGCGTAAAA CGTGTCAACT ATAAATAAAT AAATAGAATT AAAATCCAAC TTATATGTGA AAATTCATTG 420
Quality scores 64 0
AJ489607 consensus CGGCGTAAAA CGTGTCAACT ATAAATAAAT AAATAGAATT AAAATCCAAC TTATATGTGA AAATTCATTG 420
Quality scores 64 0
AJ489607 consensus CGGCGTAAAA CGTGTCAACT ATAAATAAAT AAATAGAATT AAAATCCAAC TTATATGTGA AAATTCATTG 420
Quality scores 64 0
AJ489607 consensus CGGCGTAAAA CGTGTCAACT ATAAATAAAT AAATAGAATT AAAATCCAAC TTATATGTGA AAATTCATTG 420
Quality scores 64 0
AJ489607 consensus CGGCGTAAAA CGTGTCAACT ATAAATAAAT AAATAGAATT AAAATCCAAC TTATATGTGA AAATTCATTG 420
Quality scores 64 0
AJ489607 consensus CGGCGTAAAA CGTGTCAACT ATAAATAAAT AAATAGAATT AAAATCCAAC TTATATGTGA AAATTCATTG 420
Quality scores 64 0
AJ489607 consensus CGGCGTAAAA CGTGTCAACT ATAAATAAAT AAATAGAATT AAAATCCAAC TTATATGTGA AAATTCATTG 420
Quality scores 64 0
Consensus CGGCGTAAAA CGTGTCAACT ATAAATAAAT AAATAGAATT AAAATCCAAC TTATATGTGA AAATTCATTG
Conservation 100% 0%
Sequence logo 2.0bits 0.0bits CGGCGTAAAA CGTGTCAACT ATAAATAAAT AAATAGAATT AAAATCCAAC TTATATGTGA AAATTCATTG

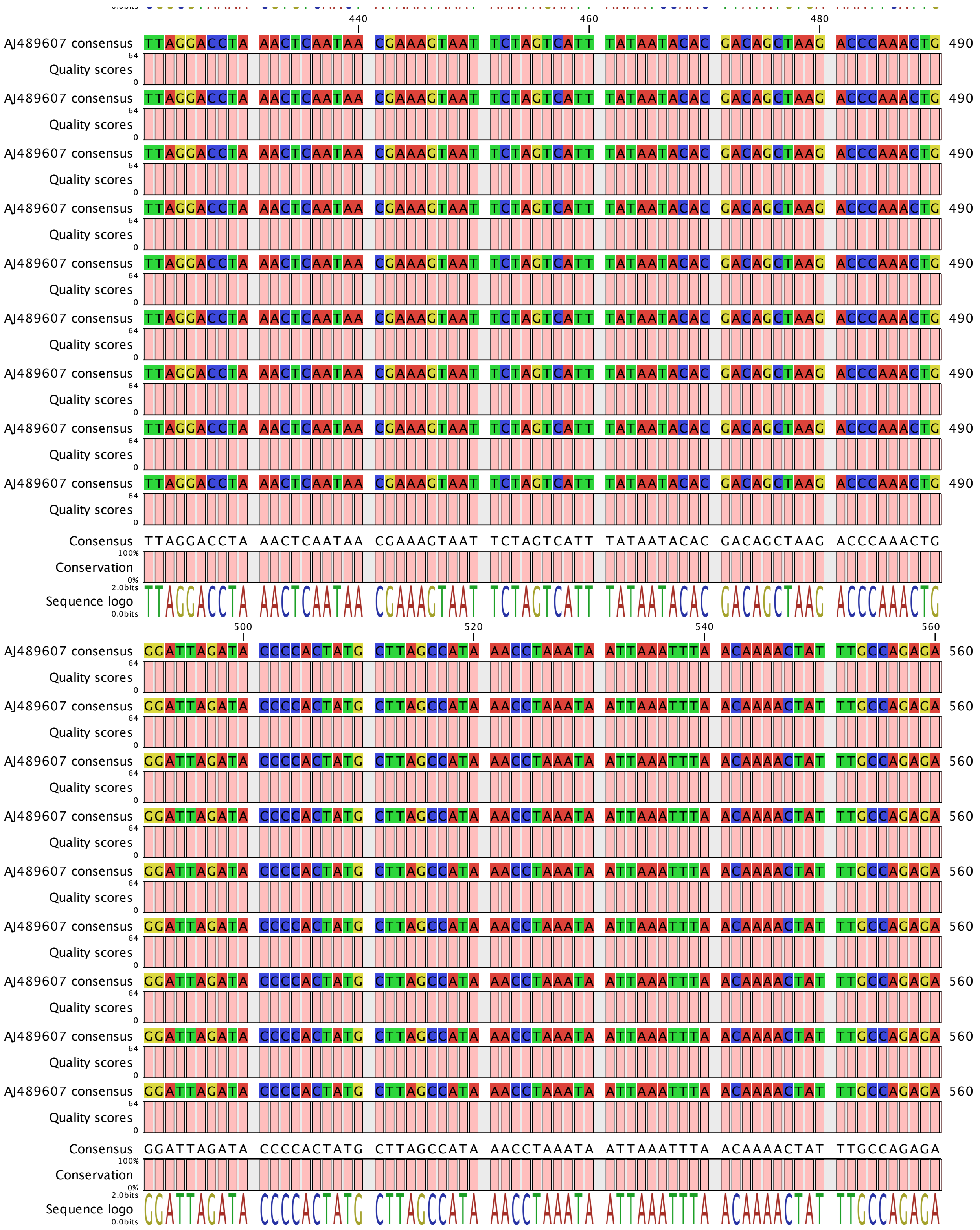

440 460 480
AJ489607 consensus TTAGGACCTA AACTCAATAA CGAAAGTAAT TCTAGTCATT TATAATACAC GACAGCTAAG ACCCAAACTG 490
64 Quality scores 0
AJ489607 consensus TTAGGACCTA AACTCAATAA CGAAAGTAAT TCTAGTCATT TATAATACAC GACAGCTAAG ACCCAAACTG 490
64 Quality scores 0
AJ489607 consensus TTAGGACCTA AACTCAATAA CGAAAGTAAT TCTAGTCATT TATAATACAC GACAGCTAAG ACCCAAACTG 490
64 Quality scores 0
AJ489607 consensus TTAGGACCTA AACTCAATAA CGAAAGTAAT TCTAGTCATT TATAATACAC GACAGCTAAG ACCCAAACTG 490
64 Quality scores 0
AJ489607 consensus TTAGGACCTA AACTCAATAA CGAAAGTAAT TCTAGTCATT TATAATACAC GACAGCTAAG ACCCAAACTG 490
64 Quality scores 0
AJ489607 consensus TTAGGACCTA AACTCAATAA CGAAAGTAAT TCTAGTCATT TATAATACAC GACAGCTAAG ACCCAAACTG 490
64 Quality scores 0
AJ489607 consensus TTAGGACCTA AACTCAATAA CGAAAGTAAT TCTAGTCATT TATAATACAC GACAGCTAAG ACCCAAACTG 490
64 Quality scores 0
AJ489607 consensus TTAGGACCTA AACTCAATAA CGAAAGTAAT TCTAGTCATT TATAATACAC GACAGCTAAG ACCCAAACTG 490
64 Quality scores 0
AJ489607 consensus TTAGGACCTA AACTCAATAA CGAAAGTAAT TCTAGTCATT TATAATACAC GACAGCTAAG ACCCAAACTG 490
64 Quality scores 0
Consensus TTAGGACCTA AACTCAATAA CGAAAGTAAT TCTAGTCATT TATAATACAC GACAGCTAAG ACCCAAACTG
100% Conservation 0%
2.0bits Sequence logo 0.0bits
TTAGGACCTA AACTCAATAA CGAAAGTAAT TCTAGTCATT TATAATACAC GACAGCTAAG ACCCAAACTG
500 520 540 560
AJ489607 consensus GGATTAGATA CCCCACTATG CTTAGCCATA AACCTAAATA ATTAAATTTA ACAAAACTAT TTGCCAGAGA 560
64 Quality scores 0
AJ489607 consensus GGATTAGATA CCCCACTATG CTTAGCCATA AACCTAAATA ATTAAATTTA ACAAAACTAT TTGCCAGAGA 560
64 Quality scores 0
AJ489607 consensus GGATTAGATA CCCCACTATG CTTAGCCATA AACCTAAATA ATTAAATTTA ACAAAACTAT TTGCCAGAGA 560
64 Quality scores 0
AJ489607 consensus GGATTAGATA CCCCACTATG CTTAGCCATA AACCTAAATA ATTAAATTTA ACAAAACTAT TTGCCAGAGA 560
64 Quality scores 0
AJ489607 consensus GGATTAGATA CCCCACTATG CTTAGCCATA AACCTAAATA ATTAAATTTA ACAAAACTAT TTGCCAGAGA 560
64 Quality scores 0
AJ489607 consensus GGATTAGATA CCCCACTATG CTTAGCCATA AACCTAAATA ATTAAATTTA ACAAAACTAT TTGCCAGAGA 560
64 Quality scores 0
AJ489607 consensus GGATTAGATA CCCCACTATG CTTAGCCATA AACCTAAATA ATTAAATTTA ACAAAACTAT TTGCCAGAGA 560
64 Quality scores 0
AJ489607 consensus GGATTAGATA CCCCACTATG CTTAGCCATA AACCTAAATA ATTAAATTTA ACAAAACTAT TTGCCAGAGA 560
64 Quality scores 0
AJ489607 consensus GGATTAGATA CCCCACTATG CTTAGCCATA AACCTAAATA ATTAAATTTA ACAAAACTAT TTGCCAGAGA 560
64 Quality scores 0
Consensus GGATTAGATA CCCCACTATG CTTAGCCATA AACCTAAATA ATTAAATTTA ACAAAACTAT TTGCCAGAGA
100% Conservation 0%
2.0bits Sequence logo 0.0bits
GGATTAGATA CCCCACTATG CTTAGCCATA AACCTAAATA ATTAAATTTA ACAAAACTAT TTGCCAGAGA

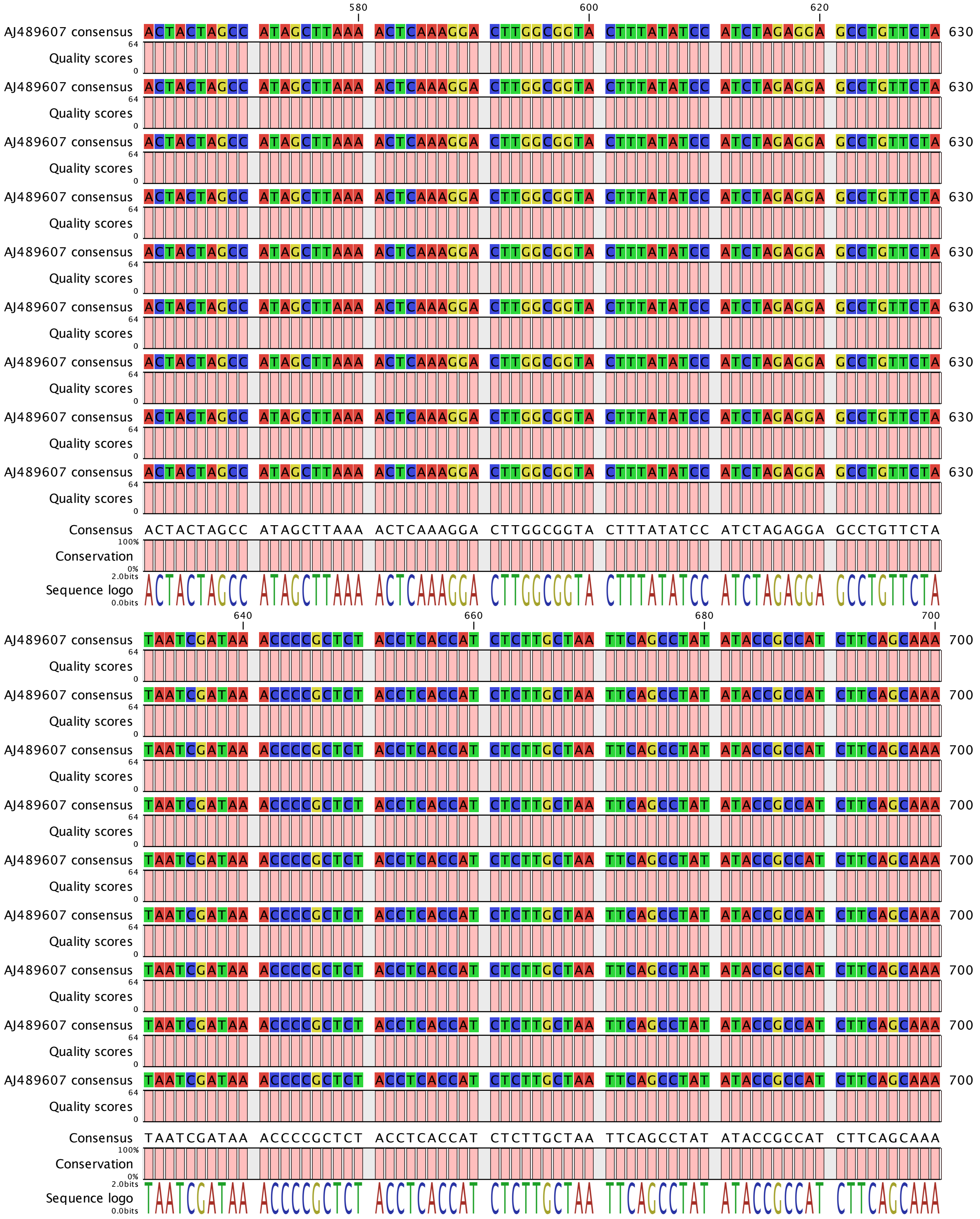
580 600 620
AJ489607 consensus ACTACTAGCC ATAGCTTAAA ACTCAAAGGA CTTGGCGGTA CTTTATATCC ATCTAGAGGA GCCTGTTCTA 630
Quality scores 64 0
AJ489607 consensus ACTACTAGCC ATAGCTTAAA ACTCAAAGGA CTTGGCGGTA CTTTATATCC ATCTAGAGGA GCCTGTTCTA 630
Quality scores 64 0
AJ489607 consensus ACTACTAGCC ATAGCTTAAA ACTCAAAGGA CTTGGCGGTA CTTTATATCC ATCTAGAGGA GCCTGTTCTA 630
Quality scores 64 0
AJ489607 consensus ACTACTAGCC ATAGCTTAAA ACTCAAAGGA CTTGGCGGTA CTTTATATCC ATCTAGAGGA GCCTGTTCTA 630
Quality scores 64 0
AJ489607 consensus ACTACTAGCC ATAGCTTAAA ACTCAAAGGA CTTGGCGGTA CTTTATATCC ATCTAGAGGA GCCTGTTCTA 630
Quality scores 64 0
AJ489607 consensus ACTACTAGCC ATAGCTTAAA ACTCAAAGGA CTTGGCGGTA CTTTATATCC ATCTAGAGGA GCCTGTTCTA 630
Quality scores 64 0
AJ489607 consensus ACTACTAGCC ATAGCTTAAA ACTCAAAGGA CTTGGCGGTA CTTTATATCC ATCTAGAGGA GCCTGTTCTA 630
Quality scores 64 0
AJ489607 consensus ACTACTAGCC ATAGCTTAAA ACTCAAAGGA CTTGGCGGTA CTTTATATCC ATCTAGAGGA GCCTGTTCTA 630
Quality scores 64 0
AJ489607 consensus ACTACTAGCC ATAGCTTAAA ACTCAAAGGA CTTGGCGGTA CTTTATATCC ATCTAGAGGA GCCTGTTCTA 630
Quality scores 64 0
Consensus ACTACTAGCC ATAGCTTAAA ACTCAAAGGA CTTGGCGGTA CTTTATATCC ATCTAGAGGA GCCTGTTCTA
Conservation 100% 0%
Sequence logo 2.0bits 0.0bits
640 660 680 700
AJ489607 consensus TAATCGATAA ACCCCGCTCT ACCTCACCAT CTCTTGCTAA TTCAGCCTAT ATACCGCCAT CTTCAGCAAA 700
Quality scores 64 0
AJ489607 consensus TAATCGATAA ACCCCGCTCT ACCTCACCAT CTCTTGCTAA TTCAGCCTAT ATACCGCCAT CTTCAGCAAA 700
Quality scores 64 0
AJ489607 consensus TAATCGATAA ACCCCGCTCT ACCTCACCAT CTCTTGCTAA TTCAGCCTAT ATACCGCCAT CTTCAGCAAA 700
Quality scores 64 0
AJ489607 consensus TAATCGATAA ACCCCGCTCT ACCTCACCAT CTCTTGCTAA TTCAGCCTAT ATACCGCCAT CTTCAGCAAA 700
Quality scores 64 0
AJ489607 consensus TAATCGATAA ACCCCGCTCT ACCTCACCAT CTCTTGCTAA TTCAGCCTAT ATACCGCCAT CTTCAGCAAA 700
Quality scores 64 0
AJ489607 consensus TAATCGATAA ACCCCGCTCT ACCTCACCAT CTCTTGCTAA TTCAGCCTAT ATACCGCCAT CTTCAGCAAA 700
Quality scores 64 0
AJ489607 consensus TAATCGATAA ACCCCGCTCT ACCTCACCAT CTCTTGCTAA TTCAGCCTAT ATACCGCCAT CTTCAGCAAA 700
Quality scores 64 0
AJ489607 consensus TAATCGATAA ACCCCGCTCT ACCTCACCAT CTCTTGCTAA TTCAGCCTAT ATACCGCCAT CTTCAGCAAA 700
Quality scores 64 0
AJ489607 consensus TAATCGATAA ACCCCGCTCT ACCTCACCAT CTCTTGCTAA TTCAGCCTAT ATACCGCCAT CTTCAGCAAA 700
Quality scores 64 0
Consensus TAATCGATAA ACCCCGCTCT ACCTCACCAT CTCTTGCTAA TTCAGCCTAT ATACCGCCAT CTTCAGCAAA
Conservation 100% 0%
Sequence logo 2.0bits 0.0bits

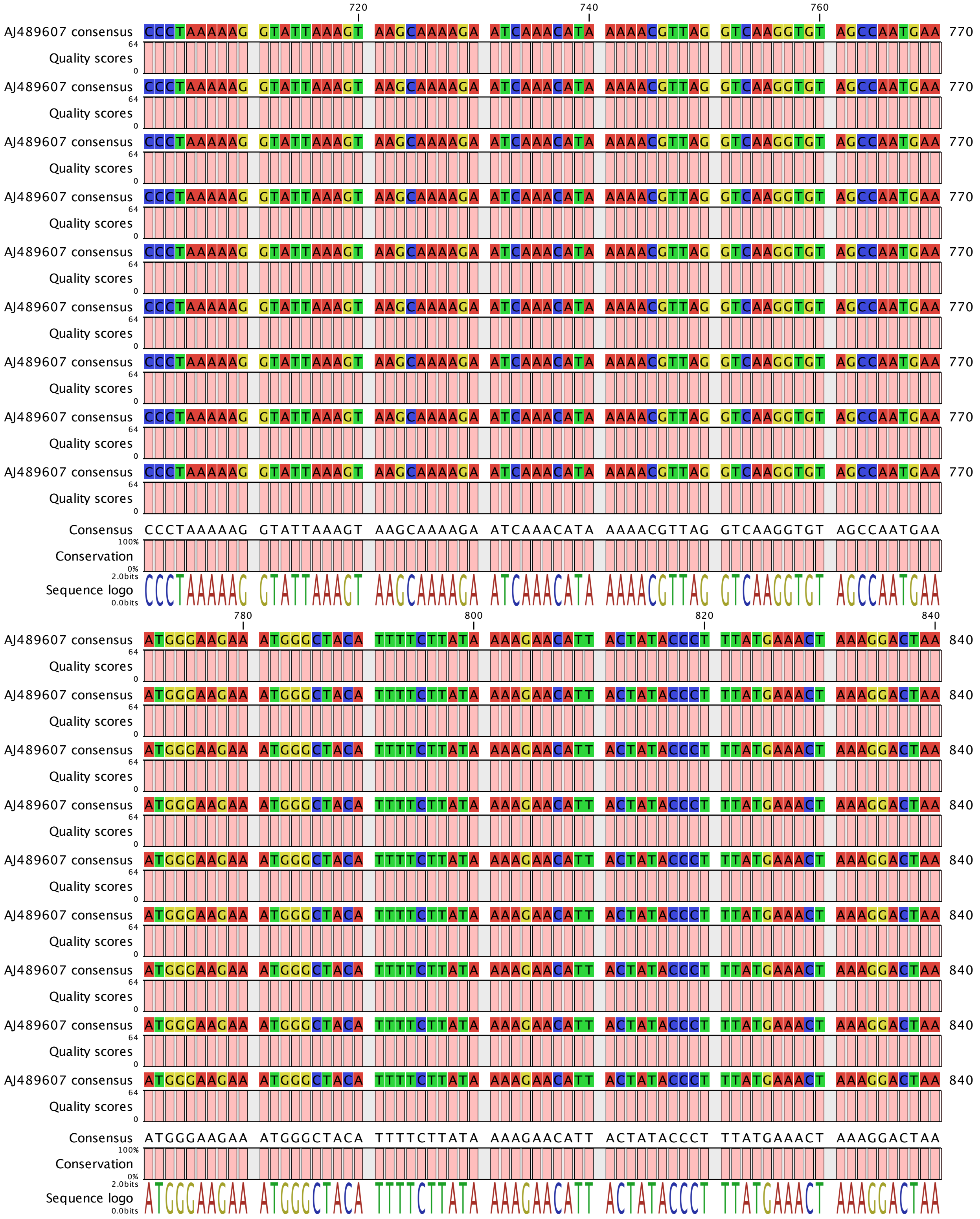

720
740
760
AJ489607 consensus CCCTAAAAAG GTATTAAAGT AAGCAAAAGA ATCAAACATA AAAACGTTAG GTCAAGGTGT AGCCAATGAA 770
Quality scores
AJ489607 consensus CCCTAAAAAG GTATTAAAGT AAGCAAAAGA ATCAAACATA AAAACGTTAG GTCAAGGTGT AGCCAATGAA 770
Quality scores
AJ489607 consensus CCCTAAAAAG GTATTAAAGT AAGCAAAAGA ATCAAACATA AAAACGTTAG GTCAAGGTGT AGCCAATGAA 770
Quality scores
AJ489607 consensus CCCTAAAAAG GTATTAAAGT AAGCAAAAGA ATCAAACATA AAAACGTTAG GTCAAGGTGT AGCCAATGAA 770
Quality scores
AJ489607 consensus CCCTAAAAAG GTATTAAAGT AAGCAAAAGA ATCAAACATA AAAACGTTAG GTCAAGGTGT AGCCAATGAA 770
Quality scores
AJ489607 consensus CCCTAAAAAG GTATTAAAGT AAGCAAAAGA ATCAAACATA AAAACGTTAG GTCAAGGTGT AGCCAATGAA 770
Quality scores
AJ489607 consensus CCCTAAAAAG GTATTAAAGT AAGCAAAAGA ATCAAACATA AAAACGTTAG GTCAAGGTGT AGCCAATGAA 770
Quality scores
AJ489607 consensus CCCTAAAAAG GTATTAAAGT AAGCAAAAGA ATCAAACATA AAAACGTTAG GTCAAGGTGT AGCCAATGAA 770
Quality scores
AJ489607 consensus CCCTAAAAAG GTATTAAAGT AAGCAAAAGA ATCAAACATA AAAACGTTAG GTCAAGGTGT AGCCAATGAA 770
Quality scores
Consensus CCCTAAAAAG GTATTAAAGT AAGCAAAAGA ATCAAACATA AAAACGTTAG GTCAAGGTGT AGCCAATGAA
Conservation
Sequence logo
780
800
820
840
AJ489607 consensus ATGGGAAGAA ATGGGCTACA TTTTCTTATA AAAGAACATT ACTATACCCT TTATGAAACT AAAGGACTAA 840
Quality scores
AJ489607 consensus ATGGGAAGAA ATGGGCTACA TTTTCTTATA AAAGAACATT ACTATACCCT TTATGAAACT AAAGGACTAA 840
Quality scores
AJ489607 consensus ATGGGAAGAA ATGGGCTACA TTTTCTTATA AAAGAACATT ACTATACCCT TTATGAAACT AAAGGACTAA 840
Quality scores
AJ489607 consensus ATGGGAAGAA ATGGGCTACA TTTTCTTATA AAAGAACATT ACTATACCCT TTATGAAACT AAAGGACTAA 840
Quality scores
AJ489607 consensus ATGGGAAGAA ATGGGCTACA TTTTCTTATA AAAGAACATT ACTATACCCT TTATGAAACT AAAGGACTAA 840
Quality scores
AJ489607 consensus ATGGGAAGAA ATGGGCTACA TTTTCTTATA AAAGAACATT ACTATACCCT TTATGAAACT AAAGGACTAA 840
Quality scores
AJ489607 consensus ATGGGAAGAA ATGGGCTACA TTTTCTTATA AAAGAACATT ACTATACCCT TTATGAAACT AAAGGACTAA 840
Quality scores
AJ489607 consensus ATGGGAAGAA ATGGGCTACA TTTTCTTATA AAAGAACATT ACTATACCCT TTATGAAACT AAAGGACTAA 840
Quality scores
AJ489607 consensus ATGGGAAGAA ATGGGCTACA TTTTCTTATA AAAGAACATT ACTATACCCT TTATGAAACT AAAGGACTAA 840
Quality scores
Consensus ATGGGAAGAA ATGGGCTACA TTTTCTTATA AAAGAACATT ACTATACCCT TTATGAAACT AAAGGACTAA
Conservation
Sequence logo

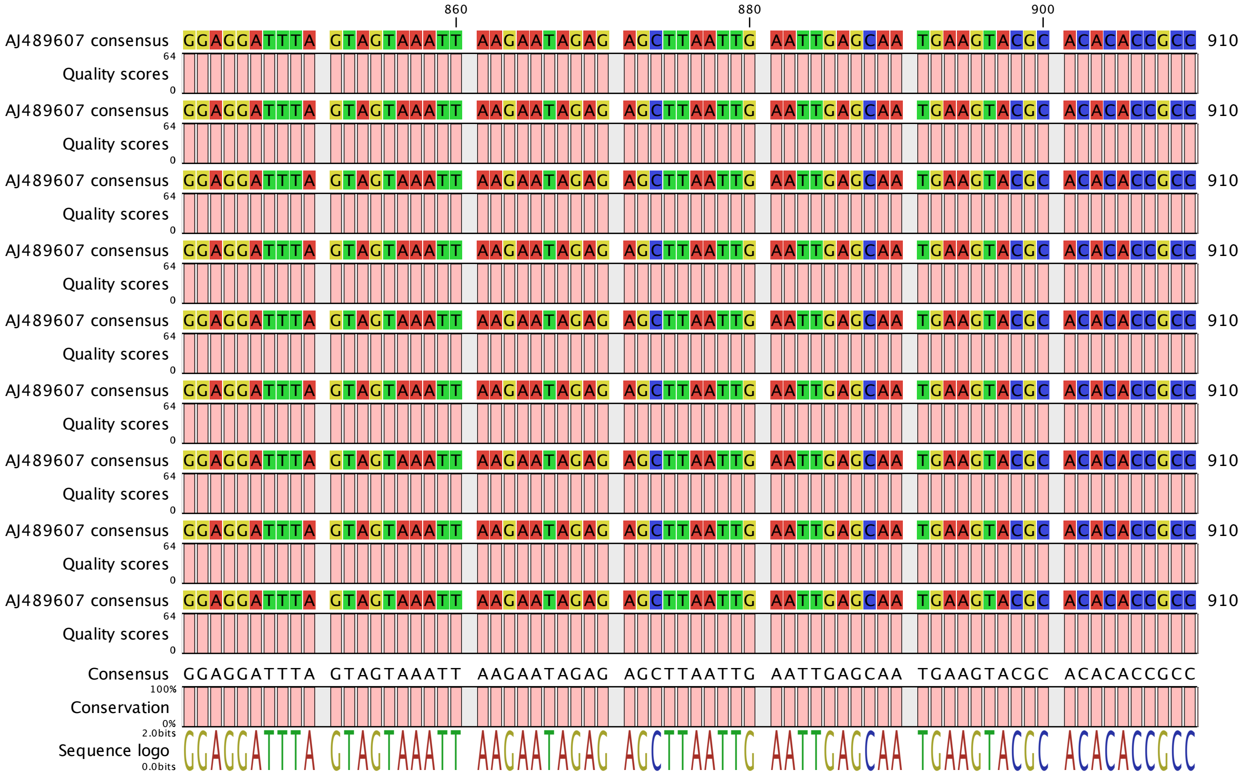
860
880
900
AJ489607 consensus GGAGGATTTA GTAGTAAATT AAGAATAGAG AGCTTAATTG AATTGAGCAA TGAAGTACGC ACACACCGCC 910
64
Quality scores
0
AJ489607 consensus GGAGGATTTA GTAGTAAATT AAGAATAGAG AGCTTAATTG AATTGAGCAA TGAAGTACGC ACACACCGCC 910
64
Quality scores
0
AJ489607 consensus GGAGGATTTA GTAGTAAATT AAGAATAGAG AGCTTAATTG AATTGAGCAA TGAAGTACGC ACACACCGCC 910
64
Quality scores
0
AJ489607 consensus GGAGGATTTA GTAGTAAATT AAGAATAGAG AGCTTAATTG AATTGAGCAA TGAAGTACGC ACACACCGCC 910
64
Quality scores
0
AJ489607 consensus GGAGGATTTA GTAGTAAATT AAGAATAGAG AGCTTAATTG AATTGAGCAA TGAAGTACGC ACACACCGCC 910
64
Quality scores
0
AJ489607 consensus GGAGGATTTA GTAGTAAATT AAGAATAGAG AGCTTAATTG AATTGAGCAA TGAAGTACGC ACACACCGCC 910
64
Quality scores
0
AJ489607 consensus GGAGGATTTA GTAGTAAATT AAGAATAGAG AGCTTAATTG AATTGAGCAA TGAAGTACGC ACACACCGCC 910
64
Quality scores
0
AJ489607 consensus GGAGGATTTA GTAGTAAATT AAGAATAGAG AGCTTAATTG AATTGAGCAA TGAAGTACGC ACACACCGCC 910
64
Quality scores
0
AJ489607 consensus GGAGGATTTA GTAGTAAATT AAGAATAGAG AGCTTAATTG AATTGAGCAA TGAAGTACGC ACACACCGCC 910
64
Quality scores
0
Consensus GGAGGATTTA GTAGTAAATT AAGAATAGAG AGCTTAATTG AATTGAGCAA TGAAGTACGC ACACACCGCC
100%
Conservation
0%
2.0bits
Sequence logo
0.0bits

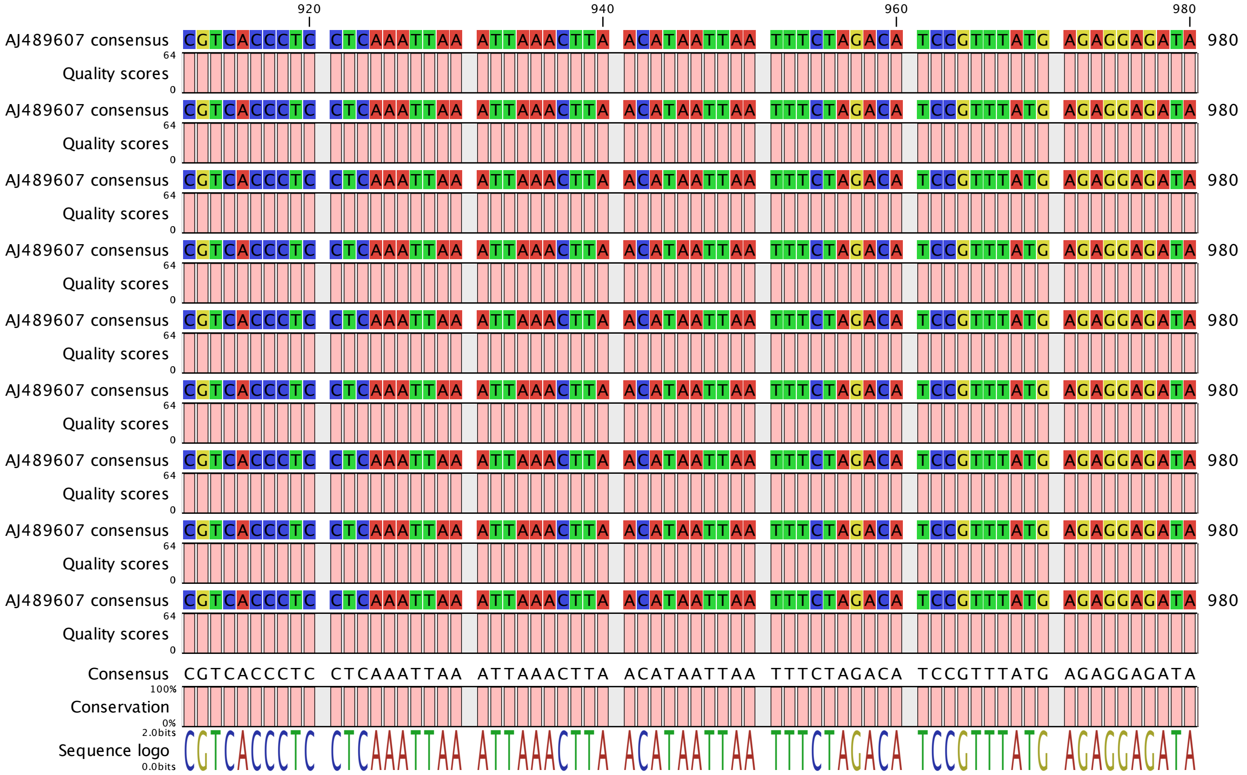
920
940
960
980
AJ489607 consensus CGTCACCCTC CTCAAATTAA ATTAAACTTA ACATAATTAA TTTCTAGACA TCCGTTTATG AGAGGAGATA 980
64
Quality scores
0
AJ489607 consensus CGTCACCCTC CTCAAATTAA ATTAAACTTA ACATAATTAA TTTCTAGACA TCCGTTTATG AGAGGAGATA 980
64
Quality scores
0
AJ489607 consensus CGTCACCCTC CTCAAATTAA ATTAAACTTA ACATAATTAA TTTCTAGACA TCCGTTTATG AGAGGAGATA 980
64
Quality scores
0
AJ489607 consensus CGTCACCCTC CTCAAATTAA ATTAAACTTA ACATAATTAA TTTCTAGACA TCCGTTTATG AGAGGAGATA 980
64
Quality scores
0
AJ489607 consensus CGTCACCCTC CTCAAATTAA ATTAAACTTA ACATAATTAA TTTCTAGACA TCCGTTTATG AGAGGAGATA 980
64
Quality scores
0
AJ489607 consensus CGTCACCCTC CTCAAATTAA ATTAAACTTA ACATAATTAA TTTCTAGACA TCCGTTTATG AGAGGAGATA 980
64
Quality scores
0
AJ489607 consensus CGTCACCCTC CTCAAATTAA ATTAAACTTA ACATAATTAA TTTCTAGACA TCCGTTTATG AGAGGAGATA 980
64
Quality scores
0
AJ489607 consensus CGTCACCCTC CTCAAATTAA ATTAAACTTA ACATAATTAA TTTCTAGACA TCCGTTTATG AGAGGAGATA 980
64
Quality scores
0
AJ489607 consensus CGTCACCCTC CTCAAATTAA ATTAAACTTA ACATAATTAA TTTCTAGACA TCCGTTTATG AGAGGAGATA 980
64
Quality scores
0
Consensus CGTCACCCTC CTCAAATTAA ATTAAACTTA ACATAATTAA TTTCTAGACA TCCGTTTATG AGAGGAGATA
100%
Conservation
0%
2.0bits
Sequence logo
0.0bits

1,000 1,020 1,040

AJ489607 consensus AGTCGTAACA AGGTAAGCAT ACTGGAAAGT GTGCTTGGAA TAATCATAGT GTAGCTTAAT ATTAAAGCAT 1050
Quality scores 64 0
AJ489607 consensus AGTCGTAACA AGGTAAGCAT ACTGGAAAGT GTGCTTGGAA TAATCATAGT GTAGCTTAAT ATTAAAGCAT 1050
Quality scores 64 0
AJ489607 consensus AGTCGTAACA AGGTAAGCAT ACTGGAAAGT GTGCTTGGAA TAATCATAGT GTAGCTTAAT ATTAAAGCAT 1050
Quality scores 64 0
AJ489607 consensus AGTCGTAACA AGGTAAGCAT ACTGGAAAGT GTGCTTGGAA TAATCATAGT GTAGCTTAAT ATTAAAGCAT 1050
Quality scores 64 0
AJ489607 consensus AGTCGTAACA AGGTAAGCAT ACTGGAAAGT GTGCTTGGAA TAATCATAGT GTAGCTTAAT ATTAAAGCAT 1050
Quality scores 64 0
AJ489607 consensus AGTCGTAACA AGGTAAGCAT ACTGGAAAGT GTGCTTGGAA TAATCATAGT GTAGCTTAAT ATTAAAGCAT 1050
Quality scores 64 0
AJ489607 consensus AGTCGTAACA AGGTAAGCAT ACTGGAAAGT GTGCTTGGAA TAATCATAGT GTAGCTTAAT ATTAAAGCAT 1050
Quality scores 64 0
AJ489607 consensus AGTCGTAACA AGGTAAGCAT ACTGGAAAGT GTGCTTGGAA TAATCATAGT GTAGCTTAAT ATTAAAGCAT 1050
Quality scores 64 0
AJ489607 consensus AGTCGTAACA AGGTAAGCAT ACTGGAAAGT GTGCTTGGAA TAATCATAGT GTAGCTTAAT ATTAAAGCAT 1050
Quality scores 64 0
Consensus AGTCGTAACA AGGTAAGCAT ACTGGAAAGT GTGCTTGGAA TAATCATAGT GTAGCTTAAT ATTAAAGCAT
Conservation 100% 0%
Sequence logo 2.0bits 0.0bits AGTCGTAACA AGGTAAGCAT ACTGGAAAGT GTGCTTGGAA TAATCATAGT GTAGCTTAAT ATTAAAGCAT

1,060 1,080 1,100 1,120

AJ489607 consensus CTGGCCTACA CCCAGAAGAT TTCATGACCA ATGAACACTC TGAACTAATC CTAGCCCTAG CCCTACACAA 1120
Quality scores 64 0
AJ489607 consensus CTGGCCTACA CCCAGAAGAT TTCATGACCA ATGAACACTC TGAACTAATC CTAGCCCTAG CCCTACACAA 1120
Quality scores 64 0
AJ489607 consensus CTGGCCTACA CCCAGAAGAT TTCATGACCA ATGAACACTC TGAACTAATC CTAGCCCTAG CCCTACACAA 1120
Quality scores 64 0
AJ489607 consensus CTGGCCTACA CCCAGAAGAT TTCATGACCA ATGAACACTC TGAACTAATC CTAGCCCTAG CCCTACACAA 1120
Quality scores 64 0
AJ489607 consensus CTGGCCTACA CCCAGAAGAT TTCATGACCA ATGAACACTC TGAACTAATC CTAGCCCTAG CCCTACACAA 1120
Quality scores 64 0
AJ489607 consensus CTGGCCTACA CCCAGAAGAT TTCATGACCA ATGAACACTC TGAACTAATC CTAGCCCTAG CCCTACACAA 1120
Quality scores 64 0
AJ489607 consensus CTGGCCTACA CCCAGAAGAT TTCATGACCA ATGAACACTC TGAACTAATC CTAGCCCTAG CCCTACACAA 1120
Quality scores 64 0
AJ489607 consensus CTGGCCTACA CCCAGAAGAT TTCATGACCA ATGAACACTC TGAACTAATC CTAGCCCTAG CCCTACACAA 1120
Quality scores 64 0
AJ489607 consensus CTGGCCTACA CCCAGAAGAT TTCATGACCA ATGAACACTC TGAACTAATC CTAGCCCTAG CCCTACACAA 1120
Quality scores 64 0
Consensus CTGGCCTACA CCCAGAAGAT TTCATGACCA ATGAACACTC TGAACTAATC CTAGCCCTAG CCCTACACAA
Conservation 100% 0%
Sequence logo 2.0bits 0.0bits CTGGCCTACA CCCAGAAGAT TTCATGACCA ATGAACACTC TGAACTAATC CTAGCCCTAG CCCTACACAA

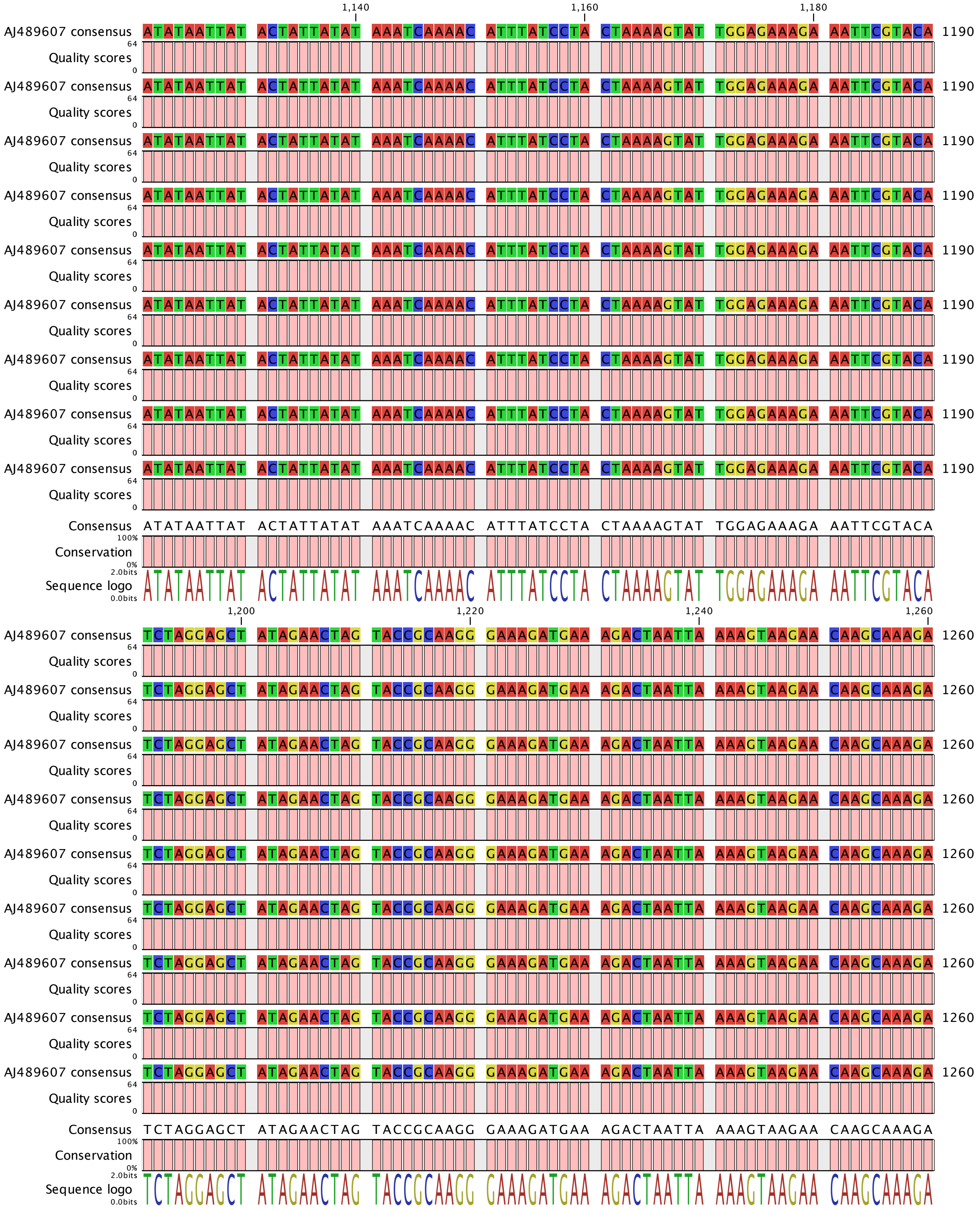
1,140 1,160 1,180
AJ489607 consensus ATATAATTAT ACTATTATAT AAATCAAAAC ATTTATCCTA CTAAAAGTAT TGGAGAAAGA AATTCGTACA 1190
Quality scores
AJ489607 consensus ATATAATTAT ACTATTATAT AAATCAAAAC ATTTATCCTA CTAAAAGTAT TGGAGAAAGA AATTCGTACA 1190
Quality scores
AJ489607 consensus ATATAATTAT ACTATTATAT AAATCAAAAC ATTTATCCTA CTAAAAGTAT TGGAGAAAGA AATTCGTACA 1190
Quality scores
AJ489607 consensus ATATAATTAT ACTATTATAT AAATCAAAAC ATTTATCCTA CTAAAAGTAT TGGAGAAAGA AATTCGTACA 1190
Quality scores
AJ489607 consensus ATATAATTAT ACTATTATAT AAATCAAAAC ATTTATCCTA CTAAAAGTAT TGGAGAAAGA AATTCGTACA 1190
Quality scores
AJ489607 consensus ATATAATTAT ACTATTATAT AAATCAAAAC ATTTATCCTA CTAAAAGTAT TGGAGAAAGA AATTCGTACA 1190
Quality scores
AJ489607 consensus ATATAATTAT ACTATTATAT AAATCAAAAC ATTTATCCTA CTAAAAGTAT TGGAGAAAGA AATTCGTACA 1190
Quality scores
AJ489607 consensus ATATAATTAT ACTATTATAT AAATCAAAAC ATTTATCCTA CTAAAAGTAT TGGAGAAAGA AATTCGTACA 1190
Quality scores
AJ489607 consensus ATATAATTAT ACTATTATAT AAATCAAAAC ATTTATCCTA CTAAAAGTAT TGGAGAAAGA AATTCGTACA 1190
Quality scores
Consensus ATATAATTAT ACTATTATAT AAATCAAAAC ATTTATCCTA CTAAAAGTAT TGGAGAAAGA AATTCGTACA
Conservation
Sequence logo
1,200 1,220 1,240 1,260
AJ489607 consensus TCTAGGAGCT ATAGAACTAG TACCGCAAGG GAAAGATGAA AGACTAATTA AAAGTAAGAA CAAGCAAAGA 1260
Quality scores
AJ489607 consensus TCTAGGAGCT ATAGAACTAG TACCGCAAGG GAAAGATGAA AGACTAATTA AAAGTAAGAA CAAGCAAAGA 1260
Quality scores
AJ489607 consensus TCTAGGAGCT ATAGAACTAG TACCGCAAGG GAAAGATGAA AGACTAATTA AAAGTAAGAA CAAGCAAAGA 1260
Quality scores
AJ489607 consensus TCTAGGAGCT ATAGAACTAG TACCGCAAGG GAAAGATGAA AGACTAATTA AAAGTAAGAA CAAGCAAAGA 1260
Quality scores
AJ489607 consensus TCTAGGAGCT ATAGAACTAG TACCGCAAGG GAAAGATGAA AGACTAATTA AAAGTAAGAA CAAGCAAAGA 1260
Quality scores
AJ489607 consensus TCTAGGAGCT ATAGAACTAG TACCGCAAGG GAAAGATGAA AGACTAATTA AAAGTAAGAA CAAGCAAAGA 1260
Quality scores
AJ489607 consensus TCTAGGAGCT ATAGAACTAG TACCGCAAGG GAAAGATGAA AGACTAATTA AAAGTAAGAA CAAGCAAAGA 1260
Quality scores
AJ489607 consensus TCTAGGAGCT ATAGAACTAG TACCGCAAGG GAAAGATGAA AGACTAATTA AAAGTAAGAA CAAGCAAAGA 1260
Quality scores
AJ489607 consensus TCTAGGAGCT ATAGAACTAG TACCGCAAGG GAAAGATGAA AGACTAATTA AAAGTAAGAA CAAGCAAAGA 1260
Quality scores
Consensus TCTAGGAGCT ATAGAACTAG TACCGCAAGG GAAAGATGAA AGACTAATTA AAAGTAAGAA CAAGCAAAGA
Conservation
Sequence logo

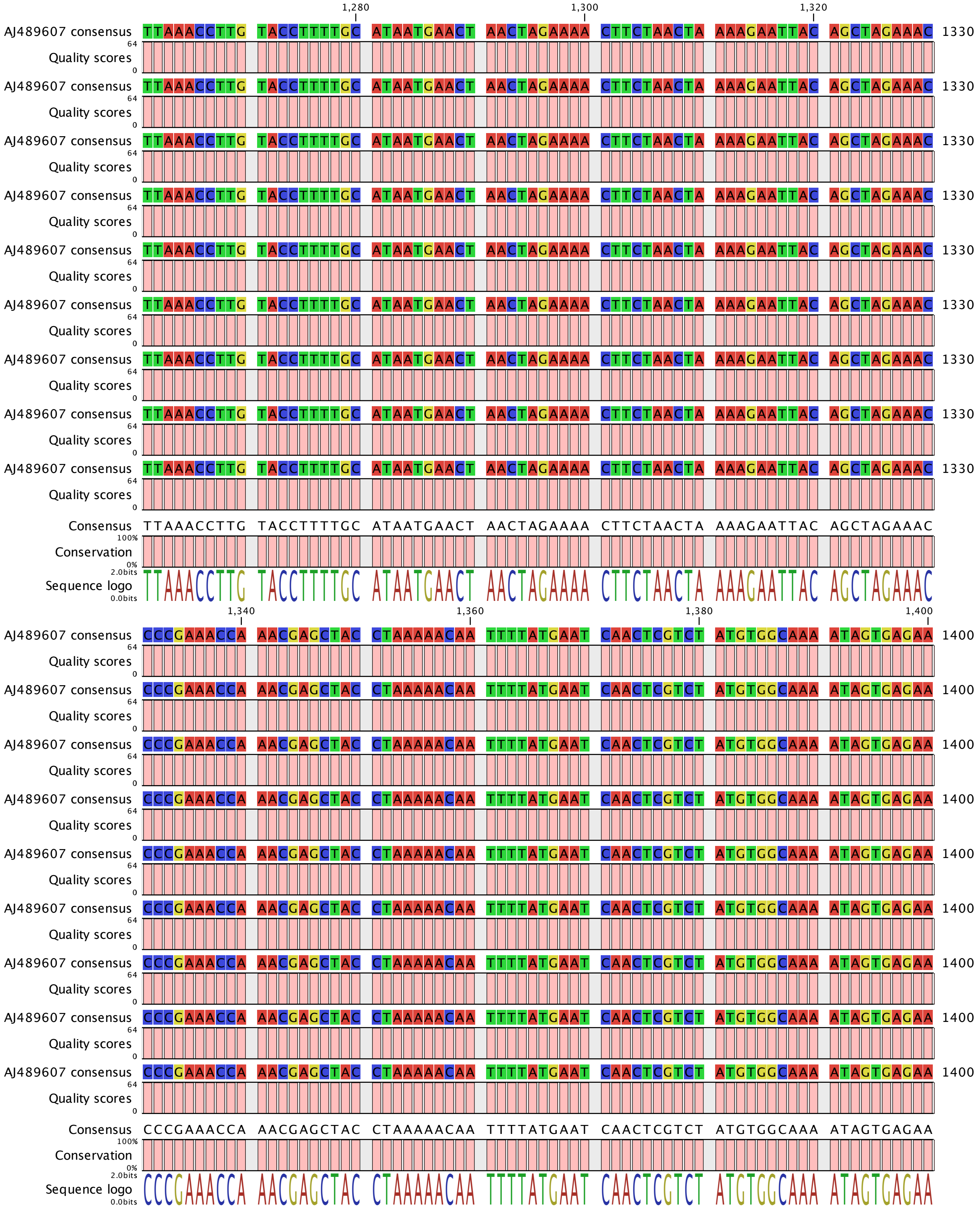

1,280
1,300
1,320
AJ489607 consensus TTAAACCTTG TACCTTTTGC ATAATGAACT AACTAGAAAA CTTCTAACTA AAAGAATTAC AGCTAGAAAC 1330
Quality scores
AJ489607 consensus TTAAACCTTG TACCTTTTGC ATAATGAACT AACTAGAAAA CTTCTAACTA AAAGAATTAC AGCTAGAAAC 1330
Quality scores
AJ489607 consensus TTAAACCTTG TACCTTTTGC ATAATGAACT AACTAGAAAA CTTCTAACTA AAAGAATTAC AGCTAGAAAC 1330
Quality scores
AJ489607 consensus TTAAACCTTG TACCTTTTGC ATAATGAACT AACTAGAAAA CTTCTAACTA AAAGAATTAC AGCTAGAAAC 1330
Quality scores
AJ489607 consensus TTAAACCTTG TACCTTTTGC ATAATGAACT AACTAGAAAA CTTCTAACTA AAAGAATTAC AGCTAGAAAC 1330
Quality scores
AJ489607 consensus TTAAACCTTG TACCTTTTGC ATAATGAACT AACTAGAAAA CTTCTAACTA AAAGAATTAC AGCTAGAAAC 1330
Quality scores
AJ489607 consensus TTAAACCTTG TACCTTTTGC ATAATGAACT AACTAGAAAA CTTCTAACTA AAAGAATTAC AGCTAGAAAC 1330
Quality scores
AJ489607 consensus TTAAACCTTG TACCTTTTGC ATAATGAACT AACTAGAAAA CTTCTAACTA AAAGAATTAC AGCTAGAAAC 1330
Quality scores
AJ489607 consensus TTAAACCTTG TACCTTTTGC ATAATGAACT AACTAGAAAA CTTCTAACTA AAAGAATTAC AGCTAGAAAC 1330
Quality scores
Consensus TTAAACCTTG TACCTTTTGC ATAATGAACT AACTAGAAAA CTTCTAACTA AAAGAATTAC AGCTAGAAAC
Conservation
Sequence logo
1,340
1,360
1,380
1,400
AJ489607 consensus CCCGAAACCA AACGAGCTAC CTAAAAACAA TTTTATGAAT CAACTCGTCT ATGTGGCAAA ATAGTGAGAA 1400
Quality scores
AJ489607 consensus CCCGAAACCA AACGAGCTAC CTAAAAACAA TTTTATGAAT CAACTCGTCT ATGTGGCAAA ATAGTGAGAA 1400
Quality scores
AJ489607 consensus CCCGAAACCA AACGAGCTAC CTAAAAACAA TTTTATGAAT CAACTCGTCT ATGTGGCAAA ATAGTGAGAA 1400
Quality scores
AJ489607 consensus CCCGAAACCA AACGAGCTAC CTAAAAACAA TTTTATGAAT CAACTCGTCT ATGTGGCAAA ATAGTGAGAA 1400
Quality scores
AJ489607 consensus CCCGAAACCA AACGAGCTAC CTAAAAACAA TTTTATGAAT CAACTCGTCT ATGTGGCAAA ATAGTGAGAA 1400
Quality scores
AJ489607 consensus CCCGAAACCA AACGAGCTAC CTAAAAACAA TTTTATGAAT CAACTCGTCT ATGTGGCAAA ATAGTGAGAA 1400
Quality scores
AJ489607 consensus CCCGAAACCA AACGAGCTAC CTAAAAACAA TTTTATGAAT CAACTCGTCT ATGTGGCAAA ATAGTGAGAA 1400
Quality scores
AJ489607 consensus CCCGAAACCA AACGAGCTAC CTAAAAACAA TTTTATGAAT CAACTCGTCT ATGTGGCAAA ATAGTGAGAA 1400
Quality scores
AJ489607 consensus CCCGAAACCA AACGAGCTAC CTAAAAACAA TTTTATGAAT CAACTCGTCT ATGTGGCAAA ATAGTGAGAA 1400
Quality scores
Consensus CCCGAAACCA AACGAGCTAC CTAAAAACAA TTTTATGAAT CAACTCGTCT ATGTGGCAAA ATAGTGAGAA
Conservation
Sequence logo

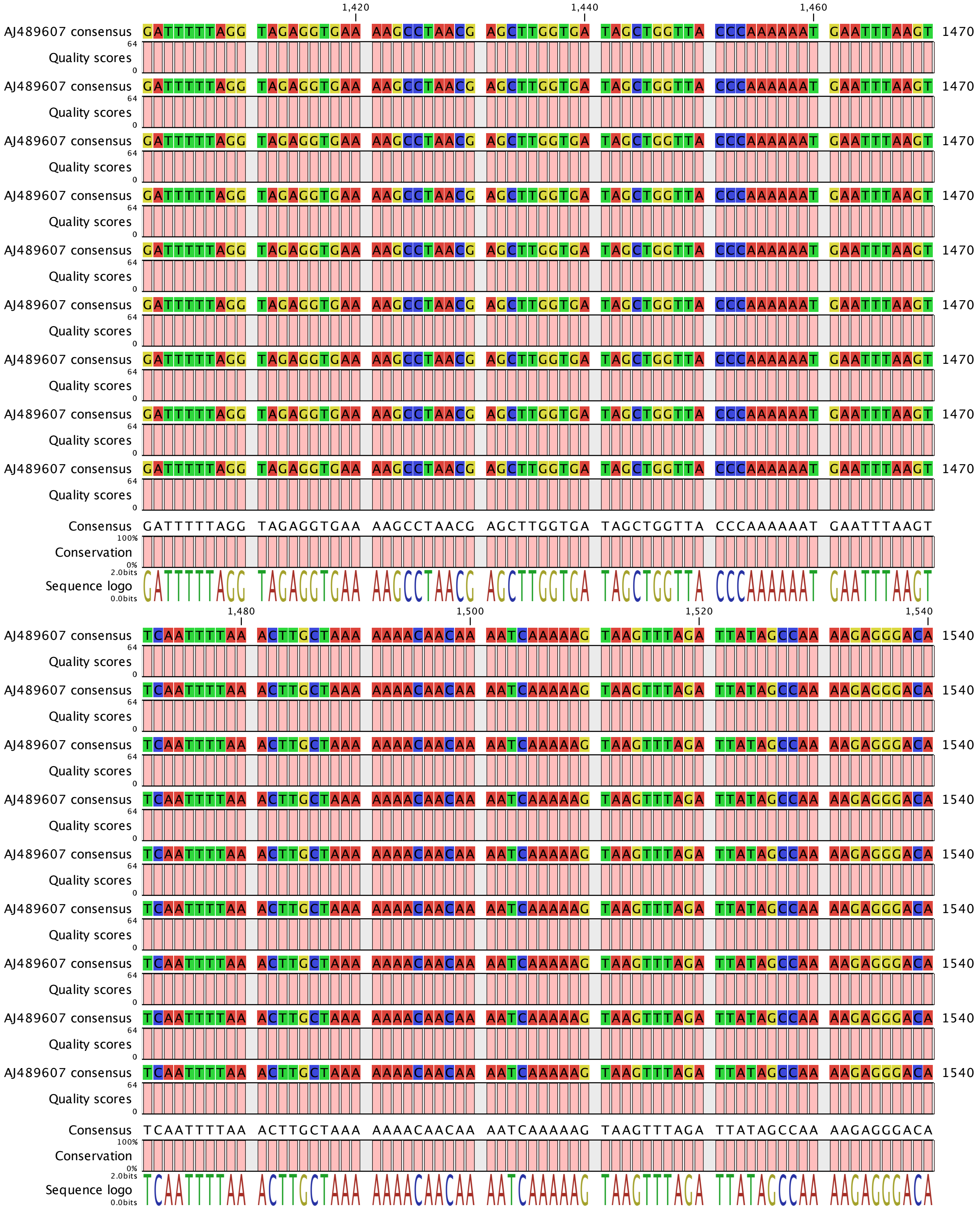
1,420 1,440 1,460
AJ489607 consensus GATTTTTAGG TAGAGGTGAA AAGCCTAACG AGCTTGGTGA TAGCTGGTTA CCCAAAAAAT GAATTTAAGT 1470
Quality scores
AJ489607 consensus GATTTTTAGG TAGAGGTGAA AAGCCTAACG AGCTTGGTGA TAGCTGGTTA CCCAAAAAAT GAATTTAAGT 1470
Quality scores
AJ489607 consensus GATTTTTAGG TAGAGGTGAA AAGCCTAACG AGCTTGGTGA TAGCTGGTTA CCCAAAAAAT GAATTTAAGT 1470
Quality scores
AJ489607 consensus GATTTTTAGG TAGAGGTGAA AAGCCTAACG AGCTTGGTGA TAGCTGGTTA CCCAAAAAAT GAATTTAAGT 1470
Quality scores
AJ489607 consensus GATTTTTAGG TAGAGGTGAA AAGCCTAACG AGCTTGGTGA TAGCTGGTTA CCCAAAAAAT GAATTTAAGT 1470
Quality scores
AJ489607 consensus GATTTTTAGG TAGAGGTGAA AAGCCTAACG AGCTTGGTGA TAGCTGGTTA CCCAAAAAAT GAATTTAAGT 1470
Quality scores
AJ489607 consensus GATTTTTAGG TAGAGGTGAA AAGCCTAACG AGCTTGGTGA TAGCTGGTTA CCCAAAAAAT GAATTTAAGT 1470
Quality scores
AJ489607 consensus GATTTTTAGG TAGAGGTGAA AAGCCTAACG AGCTTGGTGA TAGCTGGTTA CCCAAAAAAT GAATTTAAGT 1470
Quality scores
AJ489607 consensus GATTTTTAGG TAGAGGTGAA AAGCCTAACG AGCTTGGTGA TAGCTGGTTA CCCAAAAAAT GAATTTAAGT 1470
Quality scores
Consensus GATTTTTAGG TAGAGGTGAA AAGCCTAACG AGCTTGGTGA TAGCTGGTTA CCCAAAAAAT GAATTTAAGT
Conservation
Sequence logo
1,480 1,500 1,520 1,540
AJ489607 consensus TCAATTTTAA ACTTGCTAAA AAAACAACAA AATCAAAAAG TAAGTTTAGA TTATAGCCAA AAGAGGGACA 1540
Quality scores
AJ489607 consensus TCAATTTTAA ACTTGCTAAA AAAACAACAA AATCAAAAAG TAAGTTTAGA TTATAGCCAA AAGAGGGACA 1540
Quality scores
AJ489607 consensus TCAATTTTAA ACTTGCTAAA AAAACAACAA AATCAAAAAG TAAGTTTAGA TTATAGCCAA AAGAGGGACA 1540
Quality scores
AJ489607 consensus TCAATTTTAA ACTTGCTAAA AAAACAACAA AATCAAAAAG TAAGTTTAGA TTATAGCCAA AAGAGGGACA 1540
Quality scores
AJ489607 consensus TCAATTTTAA ACTTGCTAAA AAAACAACAA AATCAAAAAG TAAGTTTAGA TTATAGCCAA AAGAGGGACA 1540
Quality scores
AJ489607 consensus TCAATTTTAA ACTTGCTAAA AAAACAACAA AATCAAAAAG TAAGTTTAGA TTATAGCCAA AAGAGGGACA 1540
Quality scores
AJ489607 consensus TCAATTTTAA ACTTGCTAAA AAAACAACAA AATCAAAAAG TAAGTTTAGA TTATAGCCAA AAGAGGGACA 1540
Quality scores
AJ489607 consensus TCAATTTTAA ACTTGCTAAA AAAACAACAA AATCAAAAAG TAAGTTTAGA TTATAGCCAA AAGAGGGACA 1540
Quality scores
AJ489607 consensus TCAATTTTAA ACTTGCTAAA AAAACAACAA AATCAAAAAG TAAGTTTAGA TTATAGCCAA AAGAGGGACA 1540
Quality scores
Consensus TCAATTTTAA ACTTGCTAAA AAAACAACAA AATCAAAAAG TAAGTTTAGA TTATAGCCAA AAGAGGGACA
Conservation
Sequence logo

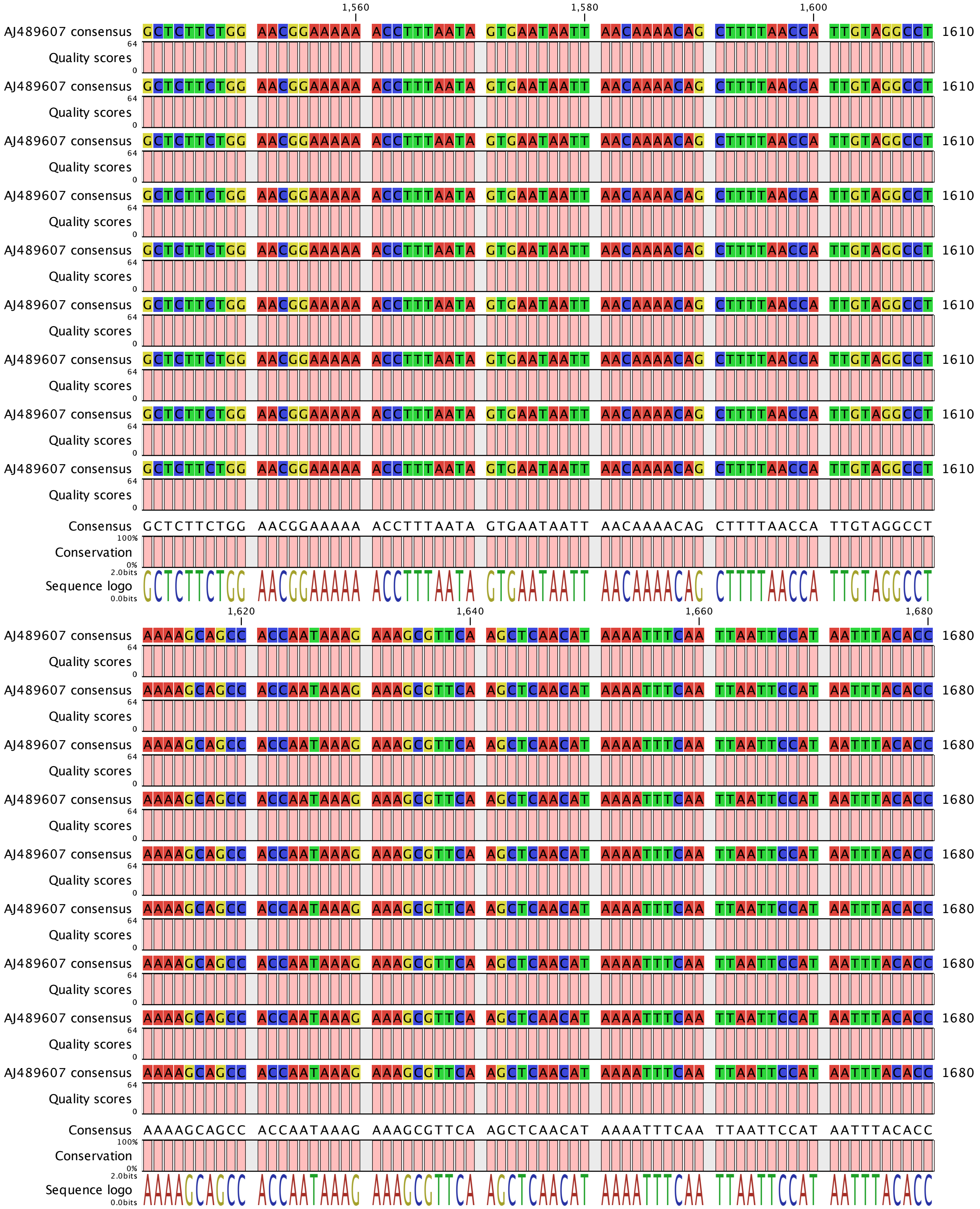
1,560 1,580 1,600
AJ489607 consensus GCTCTTCTGG AACGGAAAAA ACCTTTAATA GTGAATAATT AACAAAACAG CTTTTAACCA TTGTAGGCCT 1610
64
Quality scores
0
AJ489607 consensus GCTCTTCTGG AACGGAAAAA ACCTTTAATA GTGAATAATT AACAAAACAG CTTTTAACCA TTGTAGGCCT 1610
64
Quality scores
0
AJ489607 consensus GCTCTTCTGG AACGGAAAAA ACCTTTAATA GTGAATAATT AACAAAACAG CTTTTAACCA TTGTAGGCCT 1610
64
Quality scores
0
AJ489607 consensus GCTCTTCTGG AACGGAAAAA ACCTTTAATA GTGAATAATT AACAAAACAG CTTTTAACCA TTGTAGGCCT 1610
64
Quality scores
0
AJ489607 consensus GCTCTTCTGG AACGGAAAAA ACCTTTAATA GTGAATAATT AACAAAACAG CTTTTAACCA TTGTAGGCCT 1610
64
Quality scores
0
AJ489607 consensus GCTCTTCTGG AACGGAAAAA ACCTTTAATA GTGAATAATT AACAAAACAG CTTTTAACCA TTGTAGGCCT 1610
64
Quality scores
0
AJ489607 consensus GCTCTTCTGG AACGGAAAAA ACCTTTAATA GTGAATAATT AACAAAACAG CTTTTAACCA TTGTAGGCCT 1610
64
Quality scores
0
AJ489607 consensus GCTCTTCTGG AACGGAAAAA ACCTTTAATA GTGAATAATT AACAAAACAG CTTTTAACCA TTGTAGGCCT 1610
64
Quality scores
0
AJ489607 consensus GCTCTTCTGG AACGGAAAAA ACCTTTAATA GTGAATAATT AACAAAACAG CTTTTAACCA TTGTAGGCCT 1610
64
Quality scores
0
Consensus GCTCTTCTGG AACGGAAAAA ACCTTTAATA GTGAATAATT AACAAAACAG CTTTTAACCA TTGTAGGCCT
100%
Conservation
0%
2.0bits
Sequence logo
0.0bits
1,620 1,640 1,660 1,680
AJ489607 consensus AAAAGCAGCC ACCAATAAAG AAAGCGTTCA AGCTCAACAT AAAATTTCAA TTAATTCCAT AATTTACACC 1680
64
Quality scores
0
AJ489607 consensus AAAAGCAGCC ACCAATAAAG AAAGCGTTCA AGCTCAACAT AAAATTTCAA TTAATTCCAT AATTTACACC 1680
64
Quality scores
0
AJ489607 consensus AAAAGCAGCC ACCAATAAAG AAAGCGTTCA AGCTCAACAT AAAATTTCAA TTAATTCCAT AATTTACACC 1680
64
Quality scores
0
AJ489607 consensus AAAAGCAGCC ACCAATAAAG AAAGCGTTCA AGCTCAACAT AAAATTTCAA TTAATTCCAT AATTTACACC 1680
64
Quality scores
0
AJ489607 consensus AAAAGCAGCC ACCAATAAAG AAAGCGTTCA AGCTCAACAT AAAATTTCAA TTAATTCCAT AATTTACACC 1680
64
Quality scores
0
AJ489607 consensus AAAAGCAGCC ACCAATAAAG AAAGCGTTCA AGCTCAACAT AAAATTTCAA TTAATTCCAT AATTTACACC 1680
64
Quality scores
0
AJ489607 consensus AAAAGCAGCC ACCAATAAAG AAAGCGTTCA AGCTCAACAT AAAATTTCAA TTAATTCCAT AATTTACACC 1680
64
Quality scores
0
AJ489607 consensus AAAAGCAGCC ACCAATAAAG AAAGCGTTCA AGCTCAACAT AAAATTTCAA TTAATTCCAT AATTTACACC 1680
64
Quality scores
0
AJ489607 consensus AAAAGCAGCC ACCAATAAAG AAAGCGTTCA AGCTCAACAT AAAATTTCAA TTAATTCCAT AATTTACACC 1680
64
Quality scores
0
Consensus AAAAGCAGCC ACCAATAAAG AAAGCGTTCA AGCTCAACAT AAAATTTCAA TTAATTCCAT AATTTACACC
100%
Conservation
0%
2.0bits
Sequence logo
0.0bits

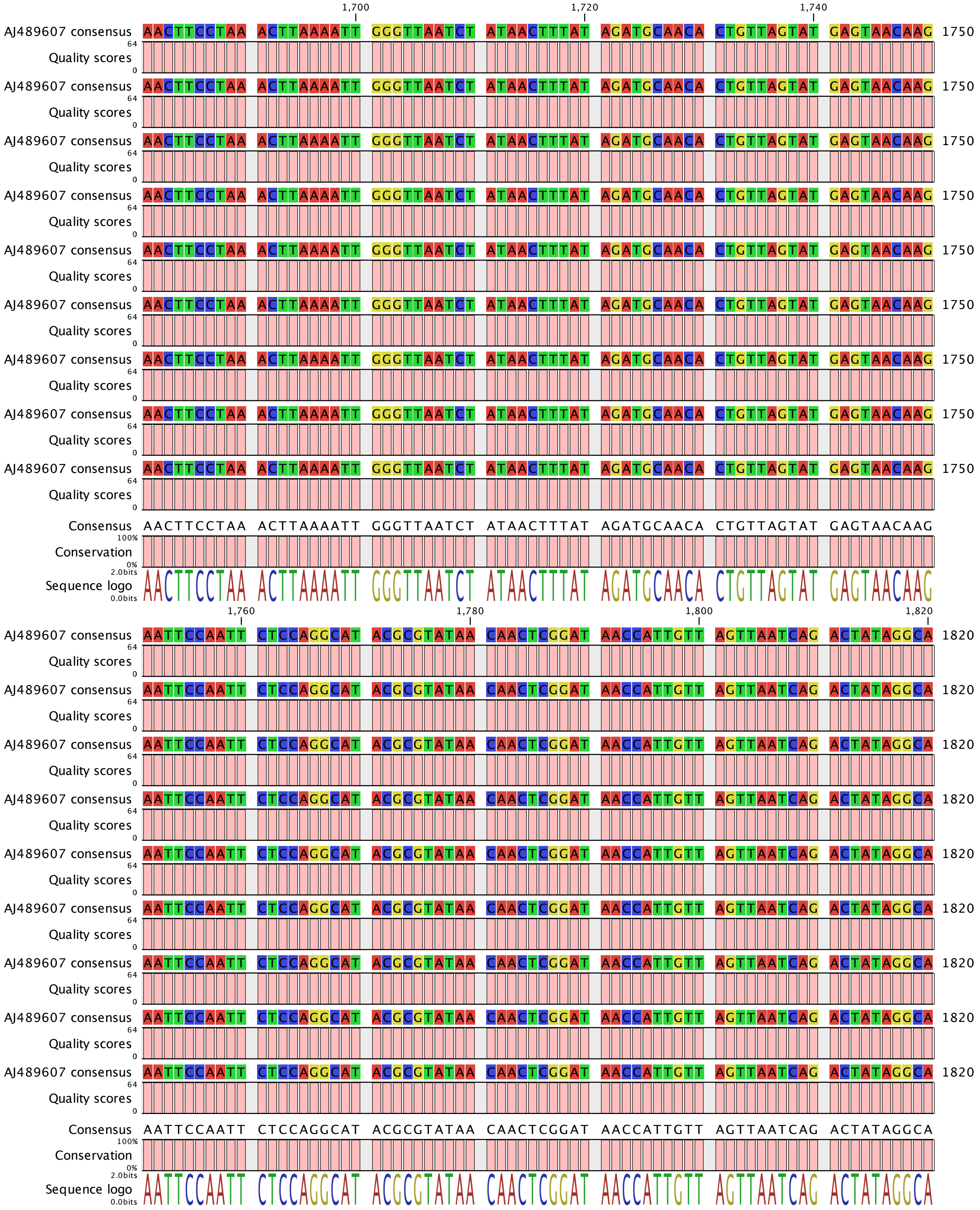
1,700
1,720
1,740
AJ489607 consensus
Quality scores
AACTTCCTAA ACTTAAAATT GGGTTAATCT ATAACTTTAT AGATGCAACA CTGTTAGTAT GAGTAACAAG 1750
Consensus AACTTCCTAA ACTTAAAATT GGGTTAATCT ATAACTTTAT AGATGCAACA CTGTTAGTAT GAGTAACAAG
Conservation
Sequence logo
1,760
1,780
1,800
1,820
AATTCCAATT CTCCAGGCAT ACGCGTATAA CAACTCGGAT AACCATTGTT AGTTAATCAG ACTATAGGCA 1820
Consensus AATTCCAATT CTCCAGGCAT ACGCGTATAA CAACTCGGAT AACCATTGTT AGTTAATCAG ACTATAGGCA

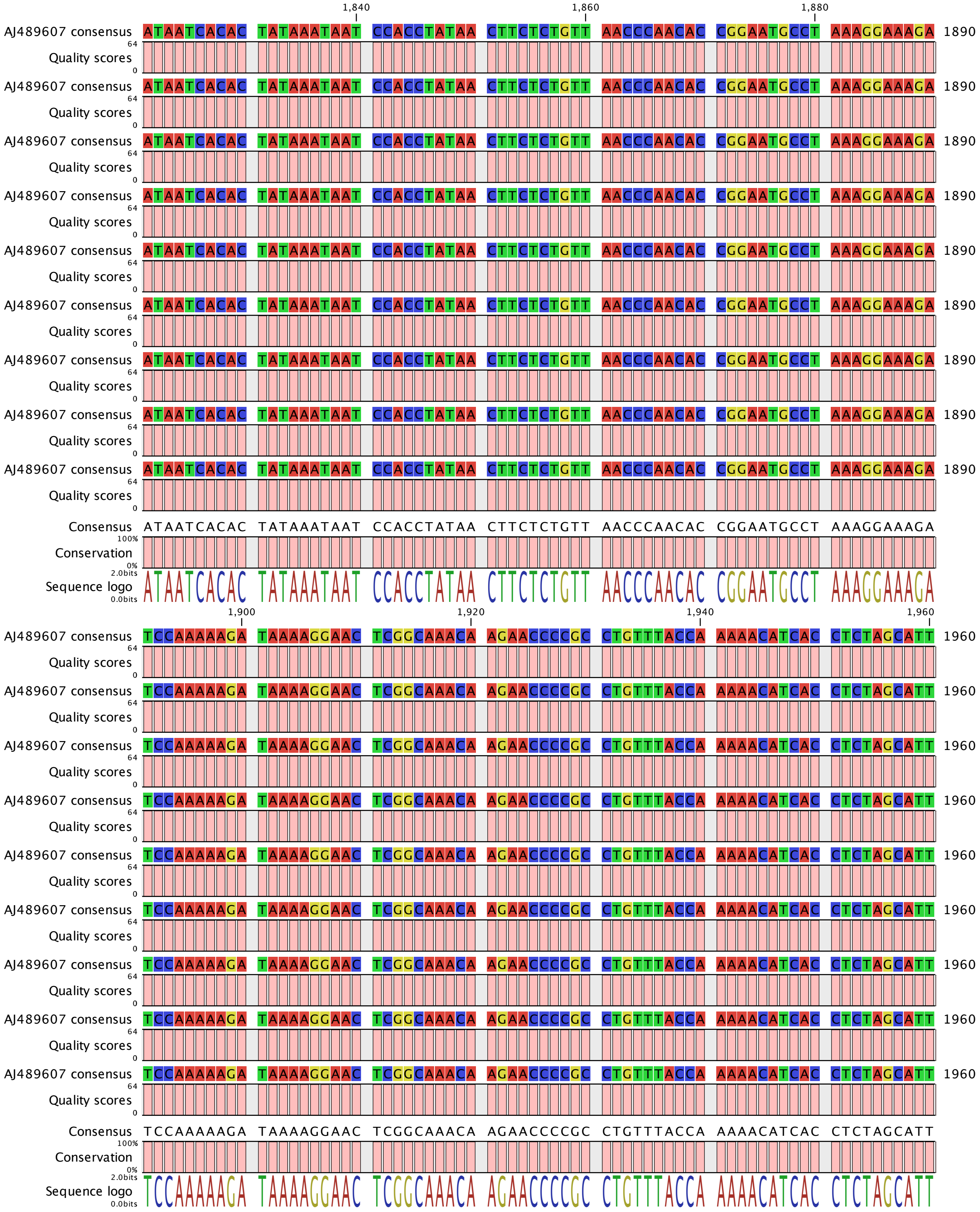
1,840
1,860
1,880
AJ489607 consensus ATAATCACAC TATAAATAAT CCACCTATAA CTTCTCTGTT AACCCAACAC CGGAATGCCT AAAGGAAAGA 1890
64 Quality scores 0
AJ489607 consensus ATAATCACAC TATAAATAAT CCACCTATAA CTTCTCTGTT AACCCAACAC CGGAATGCCT AAAGGAAAGA 1890
64 Quality scores 0
AJ489607 consensus ATAATCACAC TATAAATAAT CCACCTATAA CTTCTCTGTT AACCCAACAC CGGAATGCCT AAAGGAAAGA 1890
64 Quality scores 0
AJ489607 consensus ATAATCACAC TATAAATAAT CCACCTATAA CTTCTCTGTT AACCCAACAC CGGAATGCCT AAAGGAAAGA 1890
64 Quality scores 0
AJ489607 consensus ATAATCACAC TATAAATAAT CCACCTATAA CTTCTCTGTT AACCCAACAC CGGAATGCCT AAAGGAAAGA 1890
64 Quality scores 0
AJ489607 consensus ATAATCACAC TATAAATAAT CCACCTATAA CTTCTCTGTT AACCCAACAC CGGAATGCCT AAAGGAAAGA 1890
64 Quality scores 0
AJ489607 consensus ATAATCACAC TATAAATAAT CCACCTATAA CTTCTCTGTT AACCCAACAC CGGAATGCCT AAAGGAAAGA 1890
64 Quality scores 0
AJ489607 consensus ATAATCACAC TATAAATAAT CCACCTATAA CTTCTCTGTT AACCCAACAC CGGAATGCCT AAAGGAAAGA 1890
64 Quality scores 0
AJ489607 consensus ATAATCACAC TATAAATAAT CCACCTATAA CTTCTCTGTT AACCCAACAC CGGAATGCCT AAAGGAAAGA 1890
64 Quality scores 0
Consensus ATAATCACAC TATAAATAAT CCACCTATAA CTTCTCTGTT AACCCAACAC CGGAATGCCT AAAGGAAAGA
100% Conservation 0%
2.0bits Sequence logo 0.0bits
ATAATCACAC TATAAATAAT CCACCTATAA CTTCTCTGTT AACCCAACAC CGGAATGCCT AAAGGAAAGA
1,900
1,920
1,940
1,960
AJ489607 consensus TCCAAAAAGA TAAAAGGAAC TCGGCAAACA AGAACCCCGC CTGTTTACCA AAAACATCAC CTCTAGCATT 1960
64 Quality scores 0
AJ489607 consensus TCCAAAAAGA TAAAAGGAAC TCGGCAAACA AGAACCCCGC CTGTTTACCA AAAACATCAC CTCTAGCATT 1960
64 Quality scores 0
AJ489607 consensus TCCAAAAAGA TAAAAGGAAC TCGGCAAACA AGAACCCCGC CTGTTTACCA AAAACATCAC CTCTAGCATT 1960
64 Quality scores 0
AJ489607 consensus TCCAAAAAGA TAAAAGGAAC TCGGCAAACA AGAACCCCGC CTGTTTACCA AAAACATCAC CTCTAGCATT 1960
64 Quality scores 0
AJ489607 consensus TCCAAAAAGA TAAAAGGAAC TCGGCAAACA AGAACCCCGC CTGTTTACCA AAAACATCAC CTCTAGCATT 1960
64 Quality scores 0
AJ489607 consensus TCCAAAAAGA TAAAAGGAAC TCGGCAAACA AGAACCCCGC CTGTTTACCA AAAACATCAC CTCTAGCATT 1960
64 Quality scores 0
AJ489607 consensus TCCAAAAAGA TAAAAGGAAC TCGGCAAACA AGAACCCCGC CTGTTTACCA AAAACATCAC CTCTAGCATT 1960
64 Quality scores 0
AJ489607 consensus TCCAAAAAGA TAAAAGGAAC TCGGCAAACA AGAACCCCGC CTGTTTACCA AAAACATCAC CTCTAGCATT 1960
64 Quality scores 0
AJ489607 consensus TCCAAAAAGA TAAAAGGAAC TCGGCAAACA AGAACCCCGC CTGTTTACCA AAAACATCAC CTCTAGCATT 1960
64 Quality scores 0
Consensus TCCAAAAAGA TAAAAGGAAC TCGGCAAACA AGAACCCCGC CTGTTTACCA AAAACATCAC CTCTAGCATT
100% Conservation 0%
2.0bits Sequence logo 0.0bits
TCCAAAAAGA TAAAAGGAAC TCGGCAAACA AGAACCCCGC CTGTTTACCA AAAACATCAC CTCTAGCATT

1,980 2,000 2,020

AJ489607 consensus ACAAGTATTA GAGGCACTGC CTGCCCAGTG ACTAAAGTTT AACGGCCGCG GTATCCTGAC CGTGCAAAGG 2030
64 Quality scores 0
AJ489607 consensus ACAAGTATTA GAGGCACTGC CTGCCCAGTG ACTAAAGTTT AACGGCCGCG GTATCCTGAC CGTGCAAAGG 2030
64 Quality scores 0
AJ489607 consensus ACAAGTATTA GAGGCACTGC CTGCCCAGTG ACTAAAGTTT AACGGCCGCG GTATCCTGAC CGTGCAAAGG 2030
64 Quality scores 0
AJ489607 consensus ACAAGTATTA GAGGCACTGC CTGCCCAGTG ACTAAAGTTT AACGGCCGCG GTATCCTGAC CGTGCAAAGG 2030
64 Quality scores 0
AJ489607 consensus ACAAGTATTA GAGGCACTGC CTGCCCAGTG ACTAAAGTTT AACGGCCGCG GTATCCTGAC CGTGCAAAGG 2030
64 Quality scores 0
AJ489607 consensus ACAAGTATTA GAGGCACTGC CTGCCCAGTG ACTAAAGTTT AACGGCCGCG GTATCCTGAC CGTGCAAAGG 2030
64 Quality scores 0
AJ489607 consensus ACAAGTATTA GAGGCACTGC CTGCCCAGTG ACTAAAGTTT AACGGCCGCG GTATCCTGAC CGTGCAAAGG 2030
64 Quality scores 0
AJ489607 consensus ACAAGTATTA GAGGCACTGC CTGCCCAGTG ACTAAAGTTT AACGGCCGCG GTATCCTGAC CGTGCAAAGG 2030
64 Quality scores 0
AJ489607 consensus ACAAGTATTA GAGGCACTGC CTGCCCAGTG ACTAAAGTTT AACGGCCGCG GTATCCTGAC CGTGCAAAGG 2030
64 Quality scores 0
Consensus ACAAGTATTA GAGGCACTGC CTGCCCAGTG ACTAAAGTTT AACGGCCGCG GTATCCTGAC CGTGCAAAGG
100% Conservation 0%
2.0bits Sequence logo 0.0bits ACAAGTATTA GAGGCACTGC CTGCCCAGTG ACTAAAGTTT AACGGCCGCG GTATCCTGAC CGTGCAAAGG

2,040 2,060 2,080 2,100

AJ489607 consensus TAGCATAATC ACTTGTTCCT TAATTAGGGA CTAGCATGAA CGGCTAAACG AGGGTCCAAC TGTCTCTTAT 2100
64 Quality scores 0
AJ489607 consensus TAGCATAATC ACTTGTTCCT TAATTAGGGA CTAGCATGAA CGGCTAAACG AGGGTCCAAC TGTCTCTTAT 2100
64 Quality scores 0
AJ489607 consensus TAGCATAATC ACTTGTTCCT TAATTAGGGA CTAGCATGAA CGGCTAAACG AGGGTCCAAC TGTCTCTTAT 2100
64 Quality scores 0
AJ489607 consensus TAGCATAATC ACTTGTTCCT TAATTAGGGA CTAGCATGAA CGGCTAAACG AGGGTCCAAC TGTCTCTTAT 2100
64 Quality scores 0
AJ489607 consensus TAGCATAATC ACTTGTTCCT TAATTAGGGA CTAGCATGAA CGGCTAAACG AGGGTCCAAC TGTCTCTTAT 2100
64 Quality scores 0
AJ489607 consensus TAGCATAATC ACTTGTTCCT TAATTAGGGA CTAGCATGAA CGGCTAAACG AGGGTCCAAC TGTCTCTTAT 2100
64 Quality scores 0
AJ489607 consensus TAGCATAATC ACTTGTTCCT TAATTAGGGA CTAGCATGAA CGGCTAAACG AGGGTCCAAC TGTCTCTTAT 2100
64 Quality scores 0
AJ489607 consensus TAGCATAATC ACTTGTTCCT TAATTAGGGA CTAGCATGAA CGGCTAAACG AGGGTCCAAC TGTCTCTTAT 2100
64 Quality scores 0
AJ489607 consensus TAGCATAATC ACTTGTTCCT TAATTAGGGA CTAGCATGAA CGGCTAAACG AGGGTCCAAC TGTCTCTTAT 2100
64 Quality scores 0
Consensus TAGCATAATC ACTTGTTCCT TAATTAGGGA CTAGCATGAA CGGCTAAACG AGGGTCCAAC TGTCTCTTAT
100% Conservation 0%
2.0bits Sequence logo 0.0bits TAGCATAATC ACTTGTTCCT TAATTAGGGA CTAGCATGAA CGGCTAAACG AGGGTCCAAC TGTCTCTTAT

2,120 2,140 2,160

AJ489607 consensus CTTTAATCAG TGAAATTGAC CTTTCAGTGA AGAGGCTGAA ATATAATAAT AAGACGAGAA GACCCTATGG 2170
64 Quality scores 0
AJ489607 consensus CTTTAATCAG TGAAATTGAC CTTTCAGTGA AGAGGCTGAA ATATAATAAT AAGACGAGAA GACCCTATGG 2170
64 Quality scores 0
AJ489607 consensus CTTTAATCAG TGAAATTGAC CTTTCAGTGA AGAGGCTGAA ATATAATAAT AAGACGAGAA GACCCTATGG 2170
64 Quality scores 0
AJ489607 consensus CTTTAATCAG TGAAATTGAC CTTTCAGTGA AGAGGCTGAA ATATAATAAT AAGACGAGAA GACCCTATGG 2170
64 Quality scores 0
AJ489607 consensus CTTTAATCAG TGAAATTGAC CTTTCAGTGA AGAGGCTGAA ATATAATAAT AAGACGAGAA GACCCTATGG 2170
64 Quality scores 0
AJ489607 consensus CTTTAATCAG TGAAATTGAC CTTTCAGTGA AGAGGCTGAA ATATAATAAT AAGACGAGAA GACCCTATGG 2170
64 Quality scores 0
AJ489607 consensus CTTTAATCAG TGAAATTGAC CTTTCAGTGA AGAGGCTGAA ATATAATAAT AAGACGAGAA GACCCTATGG 2170
64 Quality scores 0
AJ489607 consensus CTTTAATCAG TGAAATTGAC CTTTCAGTGA AGAGGCTGAA ATATAATAAT AAGACGAGAA GACCCTATGG 2170
64 Quality scores 0
AJ489607 consensus CTTTAATCAG TGAAATTGAC CTTTCAGTGA AGAGGCTGAA ATATAATAAT AAGACGAGAA GACCCTATGG 2170
64 Quality scores 0
Consensus CTTTAATCAG TGAAATTGAC CTTTCAGTGA AGAGGCTGAA ATATAATAAT AAGACGAGAA GACCCTATGG
100% Conservation 0%
2.0bits Sequence logo 0.0bits CTTTAATCAG TGAAATTGAC CTTTCAGTGA AGAGGCTGAA ATATAATAAT AAGACGAGAA GACCCTATGG

2,180 2,200 2,220 2,240

AJ489607 consensus AGCTTAAATT ATATAACTTA TCTATTTAAT TTATTAAACC TAATGGCCCA AAAACTATAG TATAAGTTTG 2240
64 Quality scores 0
AJ489607 consensus AGCTTAAATT ATATAACTTA TCTATTTAAT TTATTAAACC TAATGGCCCA AAAACTATAG TATAAGTTTG 2240
64 Quality scores 0
AJ489607 consensus AGCTTAAATT ATATAACTTA TCTATTTAAT TTATTAAACC TAATGGCCCA AAAACTATAG TATAAGTTTG 2240
64 Quality scores 0
AJ489607 consensus AGCTTAAATT ATATAACTTA TCTATTTAAT TTATTAAACC TAATGGCCCA AAAACTATAG TATAAGTTTG 2240
64 Quality scores 0
AJ489607 consensus AGCTTAAATT ATATAACTTA TCTATTTAAT TTATTAAACC TAATGGCCCA AAAACTATAG TATAAGTTTG 2240
64 Quality scores 0
AJ489607 consensus AGCTTAAATT ATATAACTTA TCTATTTAAT TTATTAAACC TAATGGCCCA AAAACTATAG TATAAGTTTG 2240
64 Quality scores 0
AJ489607 consensus AGCTTAAATT ATATAACTTA TCTATTTAAT TTATTAAACC TAATGGCCCA AAAACTATAG TATAAGTTTG 2240
64 Quality scores 0
AJ489607 consensus AGCTTAAATT ATATAACTTA TCTATTTAAT TTATTAAACC TAATGGCCCA AAAACTATAG TATAAGTTTG 2240
64 Quality scores 0
AJ489607 consensus AGCTTAAATT ATATAACTTA TCTATTTAAT TTATTAAACC TAATGGCCCA AAAACTATAG TATAAGTTTG 2240
64 Quality scores 0
Consensus AGCTTAAATT ATATAACTTA TCTATTTAAT TTATTAAACC TAATGGCCCA AAAACTATAG TATAAGTTTG
100% Conservation 0%
2.0bits Sequence logo 0.0bits AGCTTAAATT ATATAACTTA TCTATTTAAT TTATTAAACC TAATGGCCCA AAAACTATAG TATAAGTTTG

2,260 2,280 2,300

AJ489607 consensus AAATTTCGGT TGGGGTGACC TCGGAGAATA AAAAATCCTC CGAATGATTA TAACCTAGAC TTACAAGTCA 2310
64 Quality scores 0
AJ489607 consensus AAATTTCGGT TGGGGTGACC TCGGAGAATA AAAAATCCTC CGAATGATTA TAACCTAGAC TTACAAGTCA 2310
64 Quality scores 0
AJ489607 consensus AAATTTCGGT TGGGGTGACC TCGGAGAATA AAAAATCCTC CGAATGATTA TAACCTAGAC TTACAAGTCA 2310
64 Quality scores 0
AJ489607 consensus AAATTTCGGT TGGGGTGACC TCGGAGAATA AAAAATCCTC CGAATGATTA TAACCTAGAC TTACAAGTCA 2310
64 Quality scores 0
AJ489607 consensus AAATTTCGGT TGGGGTGACC TCGGAGAATA AAAAATCCTC CGAATGATTA TAACCTAGAC TTACAAGTCA 2310
64 Quality scores 0
AJ489607 consensus AAATTTCGGT TGGGGTGACC TCGGAGAATA AAAAATCCTC CGAATGATTA TAACCTAGAC TTACAAGTCA 2310
64 Quality scores 0
AJ489607 consensus AAATTTCGGT TGGGGTGACC TCGGAGAATA AAAAATCCTC CGAATGATTA TAACCTAGAC TTACAAGTCA 2310
64 Quality scores 0
AJ489607 consensus AAATTTCGGT TGGGGTGACC TCGGAGAATA AAAAATCCTC CGAATGATTA TAACCTAGAC TTACAAGTCA 2310
64 Quality scores 0
AJ489607 consensus AAATTTCGGT TGGGGTGACC TCGGAGAATA AAAAATCCTC CGAATGATTA TAACCTAGAC TTACAAGTCA 2310
64 Quality scores 0
Consensus AAATTTCGGT TGGGGTGACC TCGGAGAATA AAAAATCCTC CGAATGATTA TAACCTAGAC TTACAAGTCA
100% Conservation 0%
2.0bits Sequence logo 0.0bits AAATTTCGGT TGGGGTGACC TCGGAGAATA AAAAATCCTC CGAATGATTA TAACCTAGAC TTACAAGTCA

2,320 2,340 2,360 2,380

AJ489607 consensus AAGTAAAATC AACATATCTT ATTGACCCAG ATATATTTTG ATCAACGGAC CAAGTTACCC TAGGGATAAC 2380
64 Quality scores 0
AJ489607 consensus AAGTAAAATC AACATATCTT ATTGACCCAG ATATATTTTG ATCAACGGAC CAAGTTACCC TAGGGATAAC 2380
64 Quality scores 0
AJ489607 consensus AAGTAAAATC AACATATCTT ATTGACCCAG ATATATTTTG ATCAACGGAC CAAGTTACCC TAGGGATAAC 2380
64 Quality scores 0
AJ489607 consensus AAGTAAAATC AACATATCTT ATTGACCCAG ATATATTTTG ATCAACGGAC CAAGTTACCC TAGGGATAAC 2380
64 Quality scores 0
AJ489607 consensus AAGTAAAATC AACATATCTT ATTGACCCAG ATATATTTTG ATCAACGGAC CAAGTTACCC TAGGGATAAC 2380
64 Quality scores 0
AJ489607 consensus AAGTAAAATC AACATATCTT ATTGACCCAG ATATATTTTG ATCAACGGAC CAAGTTACCC TAGGGATAAC 2380
64 Quality scores 0
AJ489607 consensus AAGTAAAATC AACATATCTT ATTGACCCAG ATATATTTTG ATCAACGGAC CAAGTTACCC TAGGGATAAC 2380
64 Quality scores 0
AJ489607 consensus AAGTAAAATC AACATATCTT ATTGACCCAG ATATATTTTG ATCAACGGAC CAAGTTACCC TAGGGATAAC 2380
64 Quality scores 0
AJ489607 consensus AAGTAAAATC AACATATCTT ATTGACCCAG ATATATTTTG ATCAACGGAC CAAGTTACCC TAGGGATAAC 2380
64 Quality scores 0
Consensus AAGTAAAATC AACATATCTT ATTGACCCAG ATATATTTTG ATCAACGGAC CAAGTTACCC TAGGGATAAC
100% Conservation 0%
2.0bits Sequence logo 0.0bits AAGTAAAATC AACATATCTT ATTGACCCAG ATATATTTTG ATCAACGGAC CAAGTTACCC TAGGGATAAC

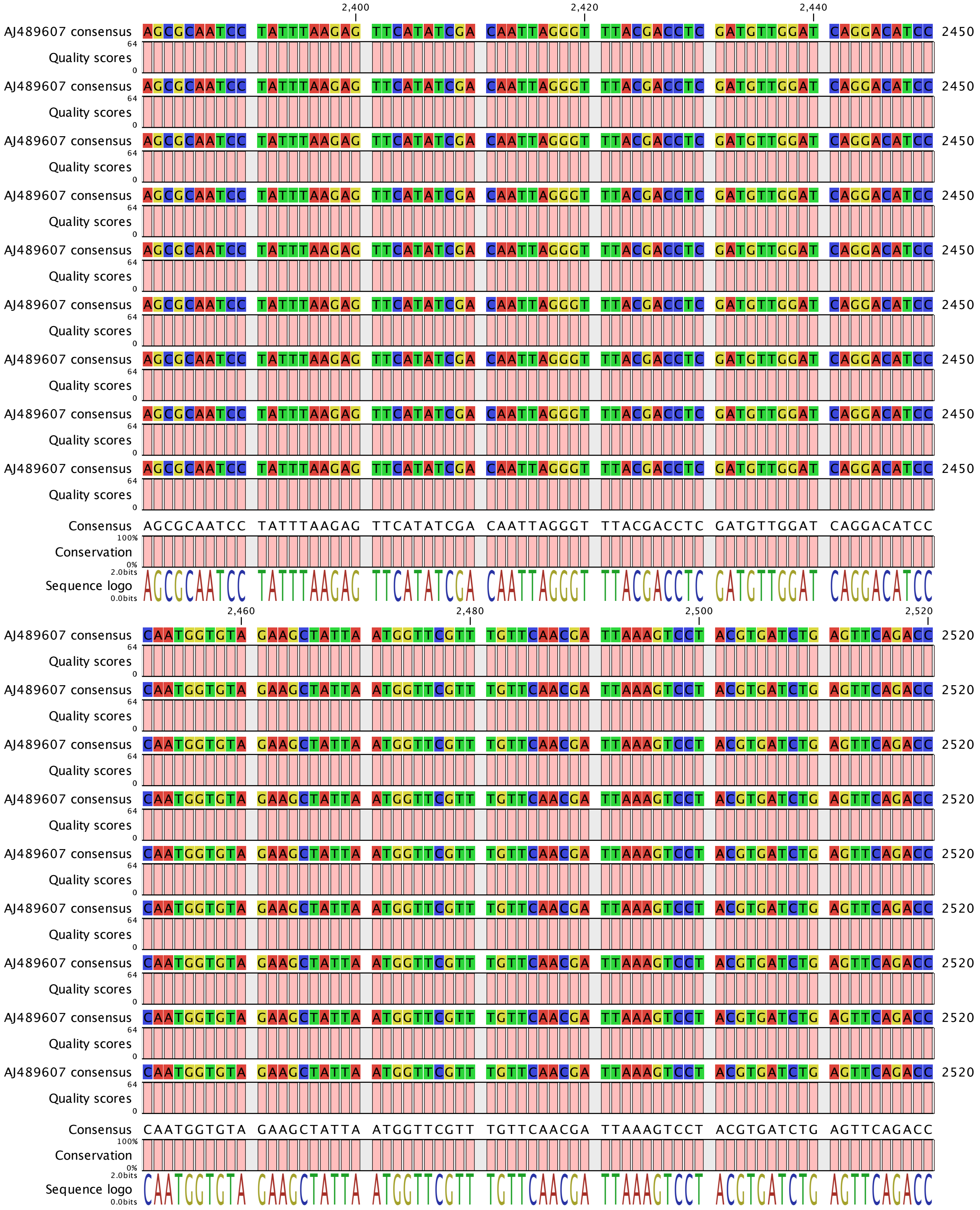
2,400 2,420 2,440
AJ489607 consensus AGCGCAATCC TATTTAAGAG TTCATATCGA CAATTAGGGT TTACGACCTC GATGTTGGAT CAGGACATCC 2450
Quality scores
AJ489607 consensus AGCGCAATCC TATTTAAGAG TTCATATCGA CAATTAGGGT TTACGACCTC GATGTTGGAT CAGGACATCC 2450
Quality scores
AJ489607 consensus AGCGCAATCC TATTTAAGAG TTCATATCGA CAATTAGGGT TTACGACCTC GATGTTGGAT CAGGACATCC 2450
Quality scores
AJ489607 consensus AGCGCAATCC TATTTAAGAG TTCATATCGA CAATTAGGGT TTACGACCTC GATGTTGGAT CAGGACATCC 2450
Quality scores
AJ489607 consensus AGCGCAATCC TATTTAAGAG TTCATATCGA CAATTAGGGT TTACGACCTC GATGTTGGAT CAGGACATCC 2450
Quality scores
AJ489607 consensus AGCGCAATCC TATTTAAGAG TTCATATCGA CAATTAGGGT TTACGACCTC GATGTTGGAT CAGGACATCC 2450
Quality scores
AJ489607 consensus AGCGCAATCC TATTTAAGAG TTCATATCGA CAATTAGGGT TTACGACCTC GATGTTGGAT CAGGACATCC 2450
Quality scores
AJ489607 consensus AGCGCAATCC TATTTAAGAG TTCATATCGA CAATTAGGGT TTACGACCTC GATGTTGGAT CAGGACATCC 2450
Quality scores
AJ489607 consensus AGCGCAATCC TATTTAAGAG TTCATATCGA CAATTAGGGT TTACGACCTC GATGTTGGAT CAGGACATCC 2450
Quality scores
Consensus AGCGCAATCC TATTTAAGAG TTCATATCGA CAATTAGGGT TTACGACCTC GATGTTGGAT CAGGACATCC
Conservation
Sequence logo
2,460 2,480 2,500 2,520
AJ489607 consensus CAATGGTGTA GAAGCTATTA ATGGTTCGTT TGTTCAACGA TTAAAGTCCT ACGTGATCTG AGTTCAGACC 2520
Quality scores
AJ489607 consensus CAATGGTGTA GAAGCTATTA ATGGTTCGTT TGTTCAACGA TTAAAGTCCT ACGTGATCTG AGTTCAGACC 2520
Quality scores
AJ489607 consensus CAATGGTGTA GAAGCTATTA ATGGTTCGTT TGTTCAACGA TTAAAGTCCT ACGTGATCTG AGTTCAGACC 2520
Quality scores
AJ489607 consensus CAATGGTGTA GAAGCTATTA ATGGTTCGTT TGTTCAACGA TTAAAGTCCT ACGTGATCTG AGTTCAGACC 2520
Quality scores
AJ489607 consensus CAATGGTGTA GAAGCTATTA ATGGTTCGTT TGTTCAACGA TTAAAGTCCT ACGTGATCTG AGTTCAGACC 2520
Quality scores
AJ489607 consensus CAATGGTGTA GAAGCTATTA ATGGTTCGTT TGTTCAACGA TTAAAGTCCT ACGTGATCTG AGTTCAGACC 2520
Quality scores
AJ489607 consensus CAATGGTGTA GAAGCTATTA ATGGTTCGTT TGTTCAACGA TTAAAGTCCT ACGTGATCTG AGTTCAGACC 2520
Quality scores
AJ489607 consensus CAATGGTGTA GAAGCTATTA ATGGTTCGTT TGTTCAACGA TTAAAGTCCT ACGTGATCTG AGTTCAGACC 2520
Quality scores
AJ489607 consensus CAATGGTGTA GAAGCTATTA ATGGTTCGTT TGTTCAACGA TTAAAGTCCT ACGTGATCTG AGTTCAGACC 2520
Quality scores
Consensus CAATGGTGTA GAAGCTATTA ATGGTTCGTT TGTTCAACGA TTAAAGTCCT ACGTGATCTG AGTTCAGACC
Conservation
Sequence logo

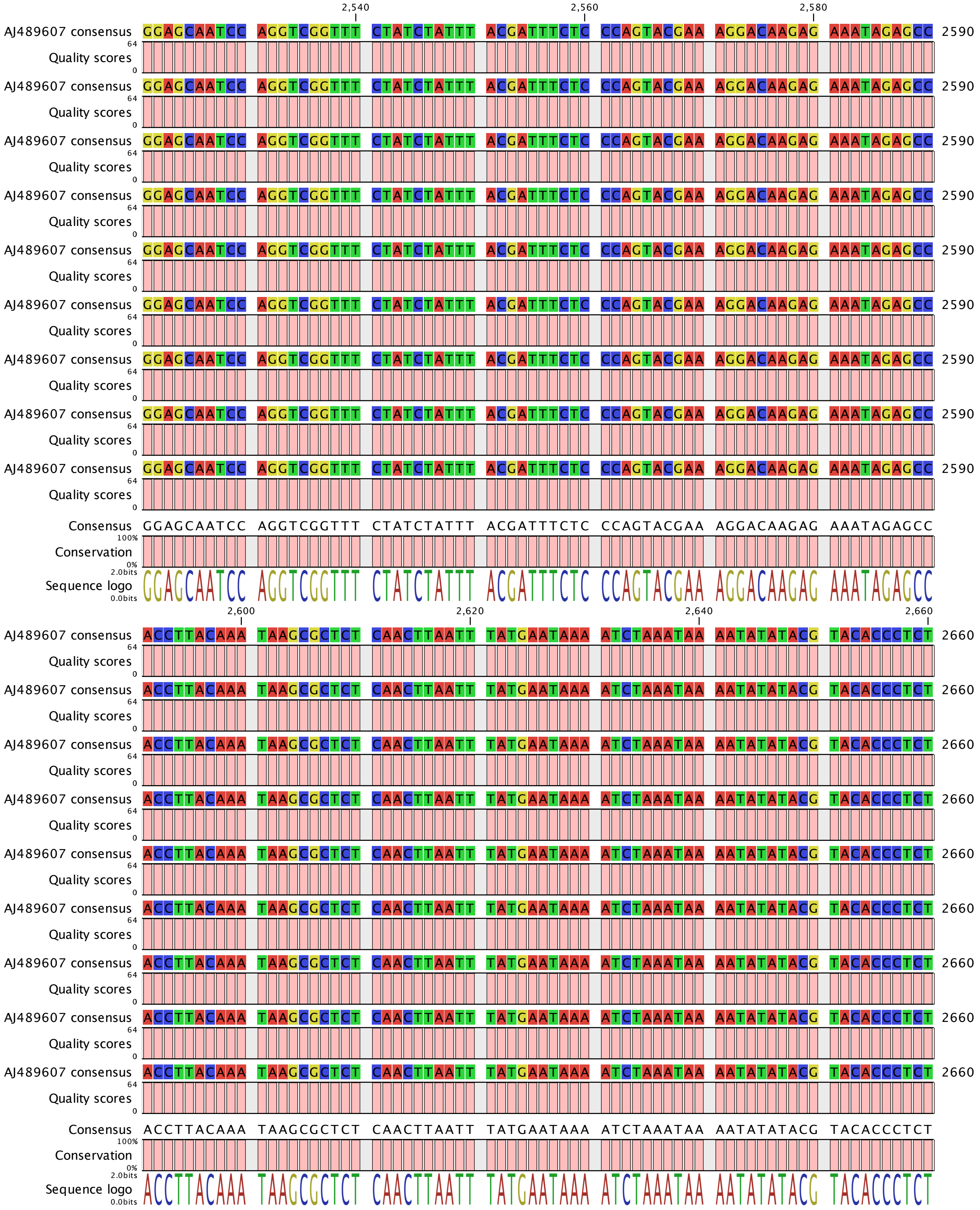
2,540
2,560
2,580
AJ489607 consensus GGAGCAATCC AGGTCGGTTT CTATCTATTT ACGATTTCTC CCAGTACGAA AGGACAAGAG AAATAGAGCC 2590
Quality scores
AJ489607 consensus GGAGCAATCC AGGTCGGTTT CTATCTATTT ACGATTTCTC CCAGTACGAA AGGACAAGAG AAATAGAGCC 2590
Quality scores
AJ489607 consensus GGAGCAATCC AGGTCGGTTT CTATCTATTT ACGATTTCTC CCAGTACGAA AGGACAAGAG AAATAGAGCC 2590
Quality scores
AJ489607 consensus GGAGCAATCC AGGTCGGTTT CTATCTATTT ACGATTTCTC CCAGTACGAA AGGACAAGAG AAATAGAGCC 2590
Quality scores
AJ489607 consensus GGAGCAATCC AGGTCGGTTT CTATCTATTT ACGATTTCTC CCAGTACGAA AGGACAAGAG AAATAGAGCC 2590
Quality scores
AJ489607 consensus GGAGCAATCC AGGTCGGTTT CTATCTATTT ACGATTTCTC CCAGTACGAA AGGACAAGAG AAATAGAGCC 2590
Quality scores
AJ489607 consensus GGAGCAATCC AGGTCGGTTT CTATCTATTT ACGATTTCTC CCAGTACGAA AGGACAAGAG AAATAGAGCC 2590
Quality scores
AJ489607 consensus GGAGCAATCC AGGTCGGTTT CTATCTATTT ACGATTTCTC CCAGTACGAA AGGACAAGAG AAATAGAGCC 2590
Quality scores
AJ489607 consensus GGAGCAATCC AGGTCGGTTT CTATCTATTT ACGATTTCTC CCAGTACGAA AGGACAAGAG AAATAGAGCC 2590
Quality scores
Consensus GGAGCAATCC AGGTCGGTTT CTATCTATTT ACGATTTCTC CCAGTACGAA AGGACAAGAG AAATAGAGCC
Conservation
Sequence logo
2,600
2,620
2,640
2,660
AJ489607 consensus ACCTTACAAA TAAGCGCTCT CAACTTAATT TATGAATAAA ATCTAAATAA AATATATACG TACACCCTCT 2660
Quality scores
AJ489607 consensus ACCTTACAAA TAAGCGCTCT CAACTTAATT TATGAATAAA ATCTAAATAA AATATATACG TACACCCTCT 2660
Quality scores
AJ489607 consensus ACCTTACAAA TAAGCGCTCT CAACTTAATT TATGAATAAA ATCTAAATAA AATATATACG TACACCCTCT 2660
Quality scores
AJ489607 consensus ACCTTACAAA TAAGCGCTCT CAACTTAATT TATGAATAAA ATCTAAATAA AATATATACG TACACCCTCT 2660
Quality scores
AJ489607 consensus ACCTTACAAA TAAGCGCTCT CAACTTAATT TATGAATAAA ATCTAAATAA AATATATACG TACACCCTCT 2660
Quality scores
AJ489607 consensus ACCTTACAAA TAAGCGCTCT CAACTTAATT TATGAATAAA ATCTAAATAA AATATATACG TACACCCTCT 2660
Quality scores
AJ489607 consensus ACCTTACAAA TAAGCGCTCT CAACTTAATT TATGAATAAA ATCTAAATAA AATATATACG TACACCCTCT 2660
Quality scores
AJ489607 consensus ACCTTACAAA TAAGCGCTCT CAACTTAATT TATGAATAAA ATCTAAATAA AATATATACG TACACCCTCT 2660
Quality scores
AJ489607 consensus ACCTTACAAA TAAGCGCTCT CAACTTAATT TATGAATAAA ATCTAAATAA AATATATACG TACACCCTCT 2660
Quality scores
Consensus ACCTTACAAA TAAGCGCTCT CAACTTAATT TATGAATAAA ATCTAAATAA AATATATACG TACACCCTCT
Conservation
Sequence logo

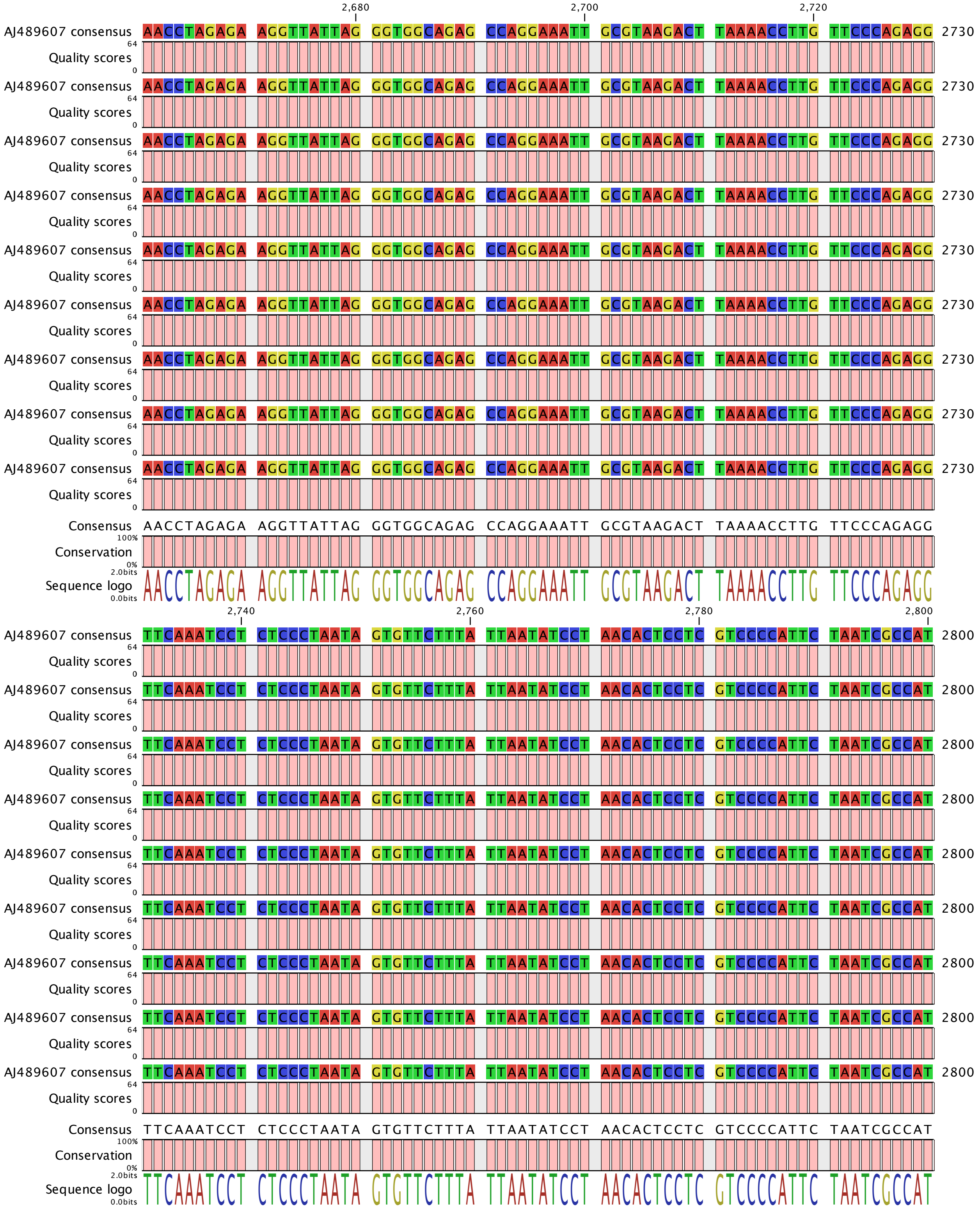

2,680
2,700
2,720
AJ489607 consensus AACCTAGAGA AGGTTATTAG GGTGGCAGAG CCAGGAAATT GCGTAAGACT TAAAACCTTG TTCCCAGAGG 2730
64
Quality scores
0
AJ489607 consensus AACCTAGAGA AGGTTATTAG GGTGGCAGAG CCAGGAAATT GCGTAAGACT TAAAACCTTG TTCCCAGAGG 2730
Quality scores
AJ489607 consensus AACCTAGAGA AGGTTATTAG GGTGGCAGAG CCAGGAAATT GCGTAAGACT TAAAACCTTG TTCCCAGAGG 2730
Quality scores
AJ489607 consensus AACCTAGAGA AGGTTATTAG GGTGGCAGAG CCAGGAAATT GCGTAAGACT TAAAACCTTG TTCCCAGAGG 2730
Quality scores
AJ489607 consensus AACCTAGAGA AGGTTATTAG GGTGGCAGAG CCAGGAAATT GCGTAAGACT TAAAACCTTG TTCCCAGAGG 2730
Quality scores
AJ489607 consensus AACCTAGAGA AGGTTATTAG GGTGGCAGAG CCAGGAAATT GCGTAAGACT TAAAACCTTG TTCCCAGAGG 2730
Quality scores
AJ489607 consensus AACCTAGAGA AGGTTATTAG GGTGGCAGAG CCAGGAAATT GCGTAAGACT TAAAACCTTG TTCCCAGAGG 2730
Quality scores
AJ489607 consensus AACCTAGAGA AGGTTATTAG GGTGGCAGAG CCAGGAAATT GCGTAAGACT TAAAACCTTG TTCCCAGAGG 2730
Quality scores
AJ489607 consensus AACCTAGAGA AGGTTATTAG GGTGGCAGAG CCAGGAAATT GCGTAAGACT TAAAACCTTG TTCCCAGAGG 2730
Quality scores
Consensus AACCTAGAGA AGGTTATTAG GGTGGCAGAG CCAGGAAATT GCGTAAGACT TAAAACCTTG TTCCCAGAGG
100%
Conservation
0%
2.0bits
Sequence logo
0.0bits
2,740
2,760
2,780
2,800
AJ489607 consensus TTCAAATCCT CTCCCTAATA GTGTTCTTTA TTAATATCCT AACACTCCTC GTCCCCATTC TAATCGCCAT 2800
Quality scores
AJ489607 consensus TTCAAATCCT CTCCCTAATA GTGTTCTTTA TTAATATCCT AACACTCCTC GTCCCCATTC TAATCGCCAT 2800
Quality scores
AJ489607 consensus TTCAAATCCT CTCCCTAATA GTGTTCTTTA TTAATATCCT AACACTCCTC GTCCCCATTC TAATCGCCAT 2800
Quality scores
AJ489607 consensus TTCAAATCCT CTCCCTAATA GTGTTCTTTA TTAATATCCT AACACTCCTC GTCCCCATTC TAATCGCCAT 2800
Quality scores
AJ489607 consensus TTCAAATCCT CTCCCTAATA GTGTTCTTTA TTAATATCCT AACACTCCTC GTCCCCATTC TAATCGCCAT 2800
Quality scores
AJ489607 consensus TTCAAATCCT CTCCCTAATA GTGTTCTTTA TTAATATCCT AACACTCCTC GTCCCCATTC TAATCGCCAT 2800
Quality scores
AJ489607 consensus TTCAAATCCT CTCCCTAATA GTGTTCTTTA TTAATATCCT AACACTCCTC GTCCCCATTC TAATCGCCAT 2800
Quality scores
AJ489607 consensus TTCAAATCCT CTCCCTAATA GTGTTCTTTA TTAATATCCT AACACTCCTC GTCCCCATTC TAATCGCCAT 2800
Quality scores
AJ489607 consensus TTCAAATCCT CTCCCTAATA GTGTTCTTTA TTAATATCCT AACACTCCTC GTCCCCATTC TAATCGCCAT 2800
Quality scores
Consensus TTCAAATCCT CTCCCTAATA GTGTTCTTTA TTAATATCCT AACACTCCTC GTCCCCATTC TAATCGCCAT
Conservation
Sequence logo

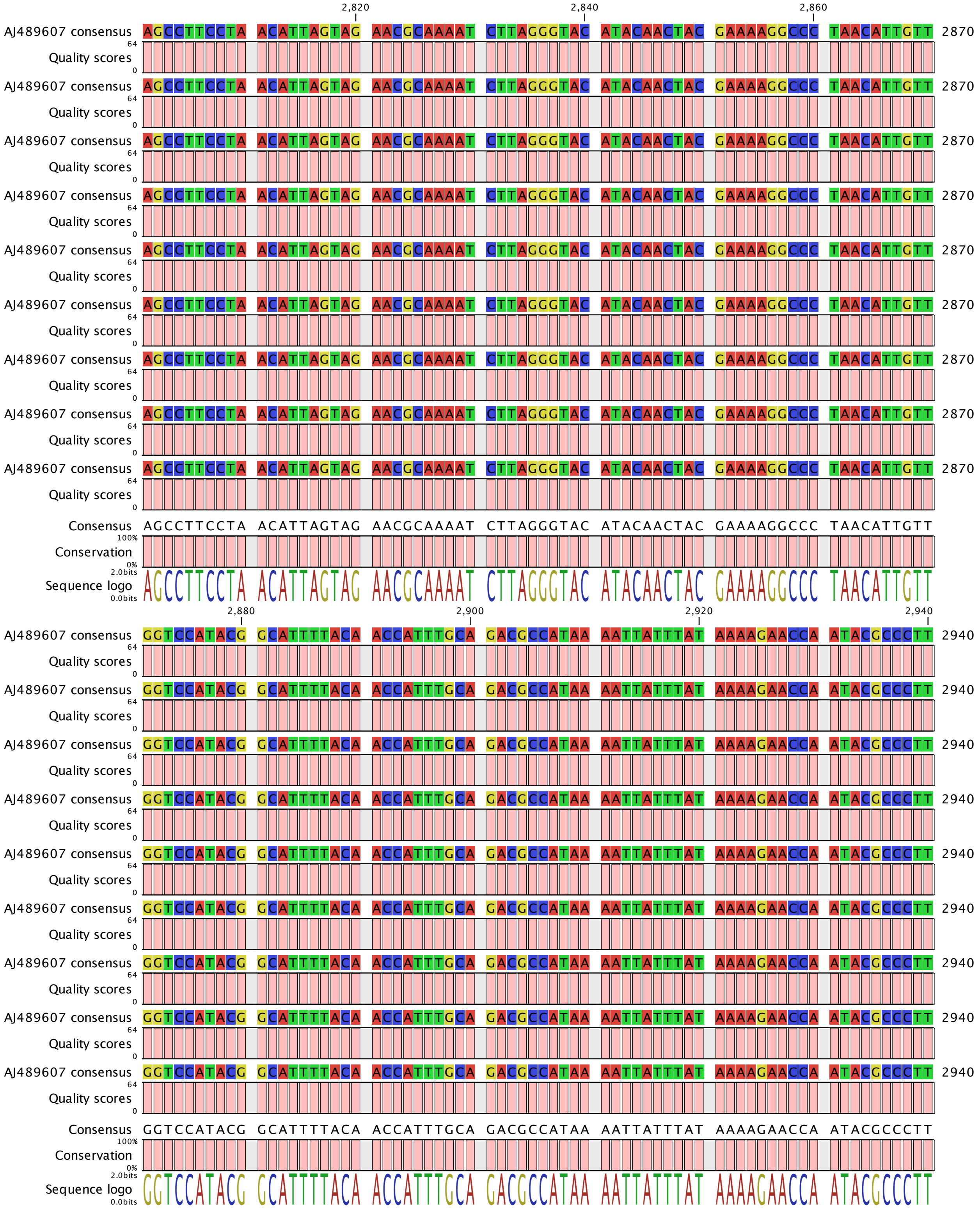
2,820 2,840 2,860
AJ489607 consensus AGCCTTCCTA ACATTAGTAG AACGCAAAAT CTTAGGGTAC ATACAACTAC GAAAAGGCCC TAACATTGTT 2870
Quality scores 64 0
AJ489607 consensus AGCCTTCCTA ACATTAGTAG AACGCAAAAT CTTAGGGTAC ATACAACTAC GAAAAGGCCC TAACATTGTT 2870
Quality scores 64 0
AJ489607 consensus AGCCTTCCTA ACATTAGTAG AACGCAAAAT CTTAGGGTAC ATACAACTAC GAAAAGGCCC TAACATTGTT 2870
Quality scores 64 0
AJ489607 consensus AGCCTTCCTA ACATTAGTAG AACGCAAAAT CTTAGGGTAC ATACAACTAC GAAAAGGCCC TAACATTGTT 2870
Quality scores 64 0
AJ489607 consensus AGCCTTCCTA ACATTAGTAG AACGCAAAAT CTTAGGGTAC ATACAACTAC GAAAAGGCCC TAACATTGTT 2870
Quality scores 64 0
AJ489607 consensus AGCCTTCCTA ACATTAGTAG AACGCAAAAT CTTAGGGTAC ATACAACTAC GAAAAGGCCC TAACATTGTT 2870
Quality scores 64 0
AJ489607 consensus AGCCTTCCTA ACATTAGTAG AACGCAAAAT CTTAGGGTAC ATACAACTAC GAAAAGGCCC TAACATTGTT 2870
Quality scores 64 0
AJ489607 consensus AGCCTTCCTA ACATTAGTAG AACGCAAAAT CTTAGGGTAC ATACAACTAC GAAAAGGCCC TAACATTGTT 2870
Quality scores 64 0
AJ489607 consensus AGCCTTCCTA ACATTAGTAG AACGCAAAAT CTTAGGGTAC ATACAACTAC GAAAAGGCCC TAACATTGTT 2870
Quality scores 64 0
Consensus AGCCTTCCTA ACATTAGTAG AACGCAAAAT CTTAGGGTAC ATACAACTAC GAAAAGGCCC TAACATTGTT
Conservation 100% 0%
Sequence logo 2.0bits 0.0bits
2,880 2,900 2,920 2,940
AJ489607 consensus GGTCCATACG GCATTTTACA ACCATTTGCA GACGCCATAA AATTATTTAT AAAAGAACCA ATACGCCCTT 2940
Quality scores 64 0
AJ489607 consensus GGTCCATACG GCATTTTACA ACCATTTGCA GACGCCATAA AATTATTTAT AAAAGAACCA ATACGCCCTT 2940
Quality scores 64 0
AJ489607 consensus GGTCCATACG GCATTTTACA ACCATTTGCA GACGCCATAA AATTATTTAT AAAAGAACCA ATACGCCCTT 2940
Quality scores 64 0
AJ489607 consensus GGTCCATACG GCATTTTACA ACCATTTGCA GACGCCATAA AATTATTTAT AAAAGAACCA ATACGCCCTT 2940
Quality scores 64 0
AJ489607 consensus GGTCCATACG GCATTTTACA ACCATTTGCA GACGCCATAA AATTATTTAT AAAAGAACCA ATACGCCCTT 2940
Quality scores 64 0
AJ489607 consensus GGTCCATACG GCATTTTACA ACCATTTGCA GACGCCATAA AATTATTTAT AAAAGAACCA ATACGCCCTT 2940
Quality scores 64 0
AJ489607 consensus GGTCCATACG GCATTTTACA ACCATTTGCA GACGCCATAA AATTATTTAT AAAAGAACCA ATACGCCCTT 2940
Quality scores 64 0
AJ489607 consensus GGTCCATACG GCATTTTACA ACCATTTGCA GACGCCATAA AATTATTTAT AAAAGAACCA ATACGCCCTT 2940
Quality scores 64 0
AJ489607 consensus GGTCCATACG GCATTTTACA ACCATTTGCA GACGCCATAA AATTATTTAT AAAAGAACCA ATACGCCCTT 2940
Quality scores 64 0
Consensus GGTCCATACG GCATTTTACA ACCATTTGCA GACGCCATAA AATTATTTAT AAAAGAACCA ATACGCCCTT
Conservation 100% 0%
Sequence logo 2.0bits 0.0bits

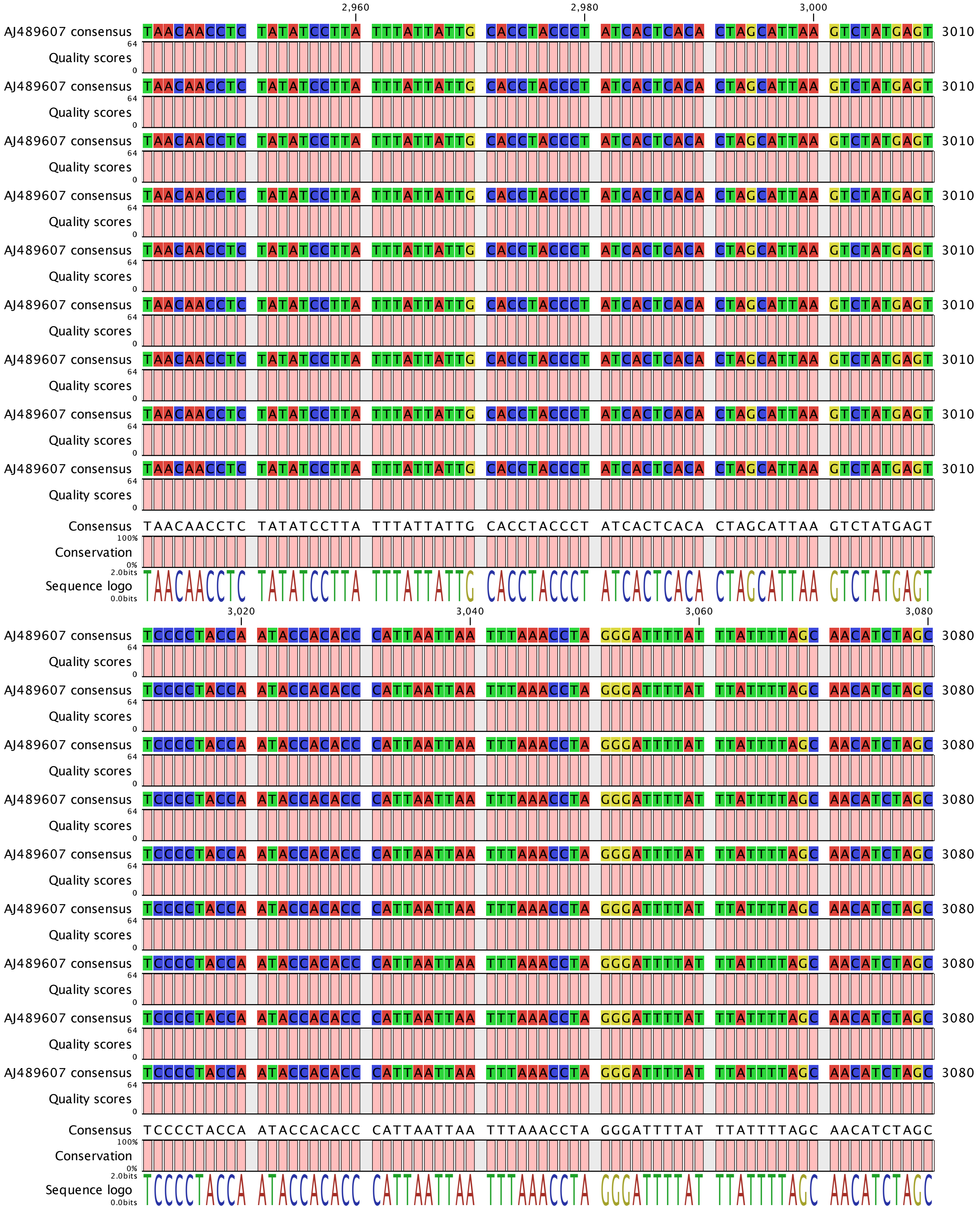
2,960
2,980
3,000
AJ489607 consensus TAACAACCTC TATATCCTTA TTTATTATTG CACCTACCCT ATCACTCACA CTAGCATTAA GTCTATGAGT 3010
Quality scores
AJ489607 consensus TAACAACCTC TATATCCTTA TTTATTATTG CACCTACCCT ATCACTCACA CTAGCATTAA GTCTATGAGT 3010
Quality scores
AJ489607 consensus TAACAACCTC TATATCCTTA TTTATTATTG CACCTACCCT ATCACTCACA CTAGCATTAA GTCTATGAGT 3010
Quality scores
AJ489607 consensus TAACAACCTC TATATCCTTA TTTATTATTG CACCTACCCT ATCACTCACA CTAGCATTAA GTCTATGAGT 3010
Quality scores
AJ489607 consensus TAACAACCTC TATATCCTTA TTTATTATTG CACCTACCCT ATCACTCACA CTAGCATTAA GTCTATGAGT 3010
Quality scores
AJ489607 consensus TAACAACCTC TATATCCTTA TTTATTATTG CACCTACCCT ATCACTCACA CTAGCATTAA GTCTATGAGT 3010
Quality scores
AJ489607 consensus TAACAACCTC TATATCCTTA TTTATTATTG CACCTACCCT ATCACTCACA CTAGCATTAA GTCTATGAGT 3010
Quality scores
AJ489607 consensus TAACAACCTC TATATCCTTA TTTATTATTG CACCTACCCT ATCACTCACA CTAGCATTAA GTCTATGAGT 3010
Quality scores
AJ489607 consensus TAACAACCTC TATATCCTTA TTTATTATTG CACCTACCCT ATCACTCACA CTAGCATTAA GTCTATGAGT 3010
Quality scores
Consensus TAACAACCTC TATATCCTTA TTTATTATTG CACCTACCCT ATCACTCACA CTAGCATTAA GTCTATGAGT
Conservation
Sequence logo
3,020
3,040
3,060
3,080
AJ489607 consensus TCCCCTACCA ATACCACACC CATTAATTAA TTTAAACCTA GGGATTTTAT TTATTTTAGC AACATCTAGC 3080
Quality scores
AJ489607 consensus TCCCCTACCA ATACCACACC CATTAATTAA TTTAAACCTA GGGATTTTAT TTATTTTAGC AACATCTAGC 3080
Quality scores
AJ489607 consensus TCCCCTACCA ATACCACACC CATTAATTAA TTTAAACCTA GGGATTTTAT TTATTTTAGC AACATCTAGC 3080
Quality scores
AJ489607 consensus TCCCCTACCA ATACCACACC CATTAATTAA TTTAAACCTA GGGATTTTAT TTATTTTAGC AACATCTAGC 3080
Quality scores
AJ489607 consensus TCCCCTACCA ATACCACACC CATTAATTAA TTTAAACCTA GGGATTTTAT TTATTTTAGC AACATCTAGC 3080
Quality scores
AJ489607 consensus TCCCCTACCA ATACCACACC CATTAATTAA TTTAAACCTA GGGATTTTAT TTATTTTAGC AACATCTAGC 3080
Quality scores
AJ489607 consensus TCCCCTACCA ATACCACACC CATTAATTAA TTTAAACCTA GGGATTTTAT TTATTTTAGC AACATCTAGC 3080
Quality scores
AJ489607 consensus TCCCCTACCA ATACCACACC CATTAATTAA TTTAAACCTA GGGATTTTAT TTATTTTAGC AACATCTAGC 3080
Quality scores
AJ489607 consensus TCCCCTACCA ATACCACACC CATTAATTAA TTTAAACCTA GGGATTTTAT TTATTTTAGC AACATCTAGC 3080
Quality scores
Consensus TCCCCTACCA ATACCACACC CATTAATTAA TTTAAACCTA GGGATTTTAT TTATTTTAGC AACATCTAGC
Conservation
Sequence logo

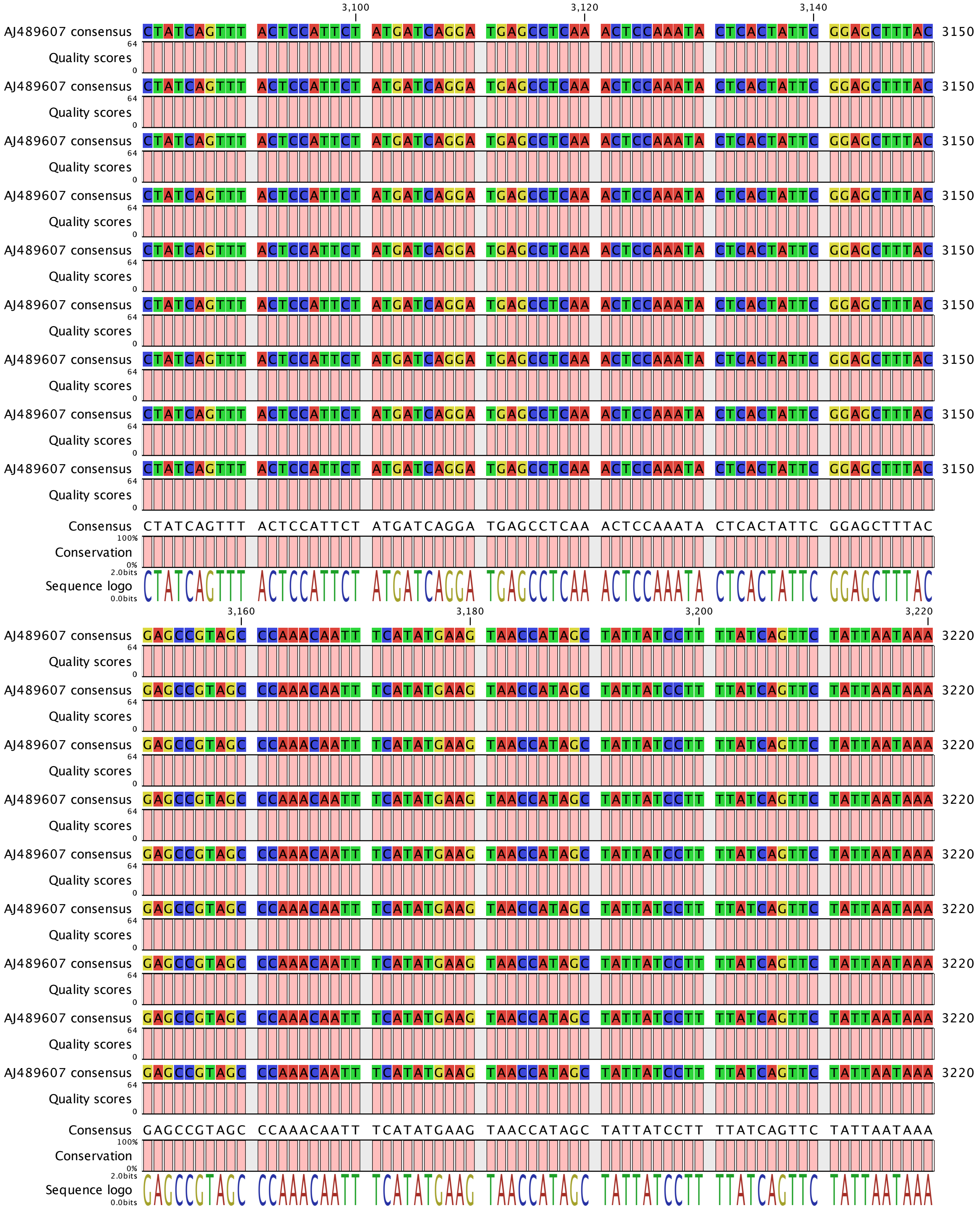
3,100
3,120
3,140
AJ489607 consensus CTATCAGTTT ACTCCATTCT ATGATCAGGA TGAGCCTCAA ACTCCAAATA CTCACTATTC GGAGCTTTAC 3150
Quality scores
AJ489607 consensus CTATCAGTTT ACTCCATTCT ATGATCAGGA TGAGCCTCAA ACTCCAAATA CTCACTATTC GGAGCTTTAC 3150
Quality scores
AJ489607 consensus CTATCAGTTT ACTCCATTCT ATGATCAGGA TGAGCCTCAA ACTCCAAATA CTCACTATTC GGAGCTTTAC 3150
Quality scores
AJ489607 consensus CTATCAGTTT ACTCCATTCT ATGATCAGGA TGAGCCTCAA ACTCCAAATA CTCACTATTC GGAGCTTTAC 3150
Quality scores
AJ489607 consensus CTATCAGTTT ACTCCATTCT ATGATCAGGA TGAGCCTCAA ACTCCAAATA CTCACTATTC GGAGCTTTAC 3150
Quality scores
AJ489607 consensus CTATCAGTTT ACTCCATTCT ATGATCAGGA TGAGCCTCAA ACTCCAAATA CTCACTATTC GGAGCTTTAC 3150
Quality scores
AJ489607 consensus CTATCAGTTT ACTCCATTCT ATGATCAGGA TGAGCCTCAA ACTCCAAATA CTCACTATTC GGAGCTTTAC 3150
Quality scores
AJ489607 consensus CTATCAGTTT ACTCCATTCT ATGATCAGGA TGAGCCTCAA ACTCCAAATA CTCACTATTC GGAGCTTTAC 3150
Quality scores
AJ489607 consensus CTATCAGTTT ACTCCATTCT ATGATCAGGA TGAGCCTCAA ACTCCAAATA CTCACTATTC GGAGCTTTAC 3150
Quality scores
Consensus CTATCAGTTT ACTCCATTCT ATGATCAGGA TGAGCCTCAA ACTCCAAATA CTCACTATTC GGAGCTTTAC
Conservation
Sequence logo
3,160
3,180
3,200
3,220
AJ489607 consensus GAGCCGTAGC CCAAACAATT TCATATGAAG TAACCATAGC TATTATCCTT TTATCAGTTC TATTAATAAA 3220
Quality scores
AJ489607 consensus GAGCCGTAGC CCAAACAATT TCATATGAAG TAACCATAGC TATTATCCTT TTATCAGTTC TATTAATAAA 3220
Quality scores
AJ489607 consensus GAGCCGTAGC CCAAACAATT TCATATGAAG TAACCATAGC TATTATCCTT TTATCAGTTC TATTAATAAA 3220
Quality scores
AJ489607 consensus GAGCCGTAGC CCAAACAATT TCATATGAAG TAACCATAGC TATTATCCTT TTATCAGTTC TATTAATAAA 3220
Quality scores
AJ489607 consensus GAGCCGTAGC CCAAACAATT TCATATGAAG TAACCATAGC TATTATCCTT TTATCAGTTC TATTAATAAA 3220
Quality scores
AJ489607 consensus GAGCCGTAGC CCAAACAATT TCATATGAAG TAACCATAGC TATTATCCTT TTATCAGTTC TATTAATAAA 3220
Quality scores
AJ489607 consensus GAGCCGTAGC CCAAACAATT TCATATGAAG TAACCATAGC TATTATCCTT TTATCAGTTC TATTAATAAA 3220
Quality scores
AJ489607 consensus GAGCCGTAGC CCAAACAATT TCATATGAAG TAACCATAGC TATTATCCTT TTATCAGTTC TATTAATAAA 3220
Quality scores
AJ489607 consensus GAGCCGTAGC CCAAACAATT TCATATGAAG TAACCATAGC TATTATCCTT TTATCAGTTC TATTAATAAA 3220
Quality scores
Consensus GAGCCGTAGC CCAAACAATT TCATATGAAG TAACCATAGC TATTATCCTT TTATCAGTTC TATTAATAAA
Conservation
Sequence logo

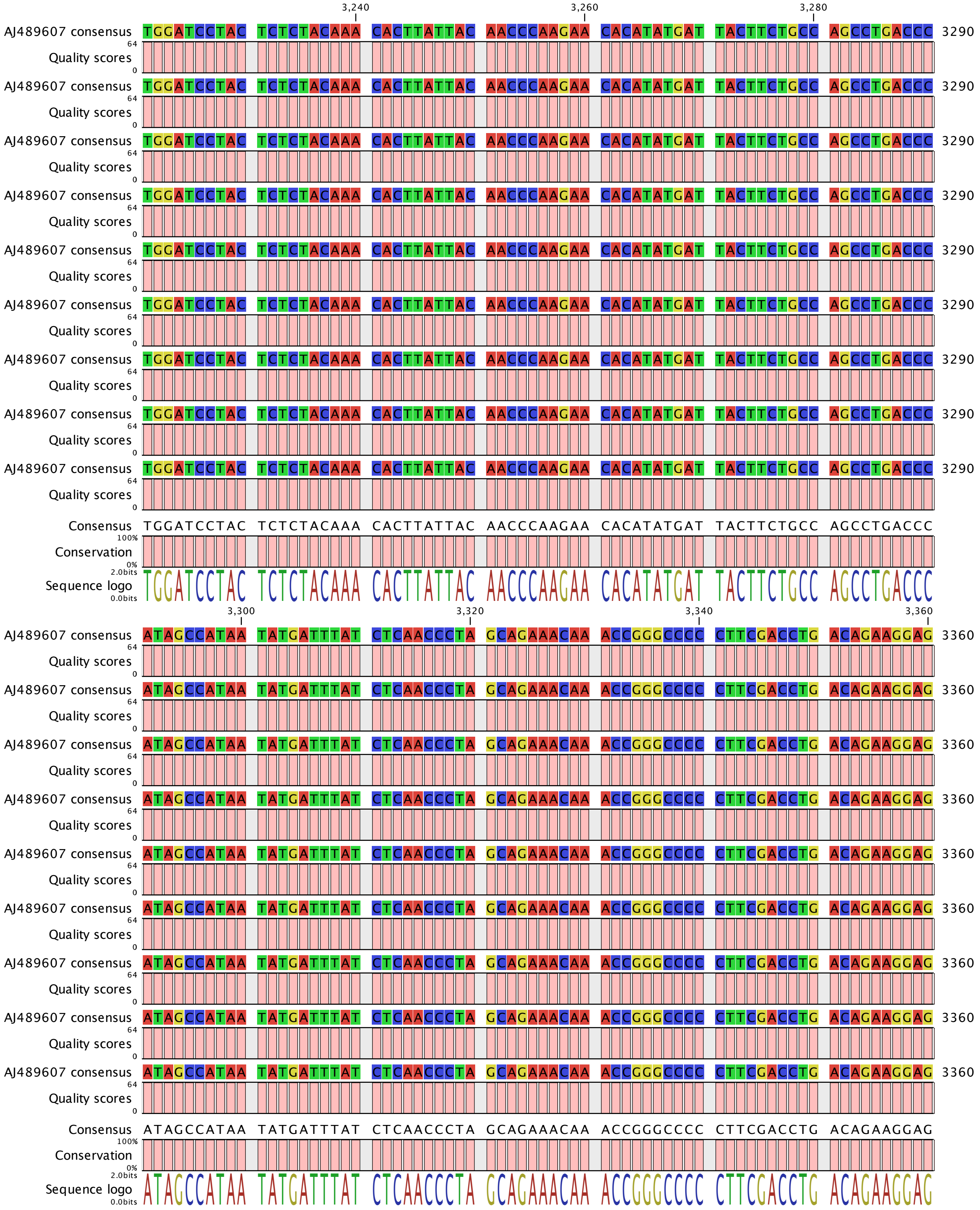
3,240
3,260
3,280
AJ489607 consensus TGGATCCTAC TCTCTACAAA CACTTATTAC AACCCAAGAA CACATATGAT TACTTCTGCC AGCCTGACCC 3290
Quality scores
AJ489607 consensus TGGATCCTAC TCTCTACAAA CACTTATTAC AACCCAAGAA CACATATGAT TACTTCTGCC AGCCTGACCC 3290
Quality scores
AJ489607 consensus TGGATCCTAC TCTCTACAAA CACTTATTAC AACCCAAGAA CACATATGAT TACTTCTGCC AGCCTGACCC 3290
Quality scores
AJ489607 consensus TGGATCCTAC TCTCTACAAA CACTTATTAC AACCCAAGAA CACATATGAT TACTTCTGCC AGCCTGACCC 3290
Quality scores
AJ489607 consensus TGGATCCTAC TCTCTACAAA CACTTATTAC AACCCAAGAA CACATATGAT TACTTCTGCC AGCCTGACCC 3290
Quality scores
AJ489607 consensus TGGATCCTAC TCTCTACAAA CACTTATTAC AACCCAAGAA CACATATGAT TACTTCTGCC AGCCTGACCC 3290
Quality scores
AJ489607 consensus TGGATCCTAC TCTCTACAAA CACTTATTAC AACCCAAGAA CACATATGAT TACTTCTGCC AGCCTGACCC 3290
Quality scores
AJ489607 consensus TGGATCCTAC TCTCTACAAA CACTTATTAC AACCCAAGAA CACATATGAT TACTTCTGCC AGCCTGACCC 3290
Quality scores
AJ489607 consensus TGGATCCTAC TCTCTACAAA CACTTATTAC AACCCAAGAA CACATATGAT TACTTCTGCC AGCCTGACCC 3290
Quality scores
Consensus TGGATCCTAC TCTCTACAAA CACTTATTAC AACCCAAGAA CACATATGAT TACTTCTGCC AGCCTGACCC
Conservation
Sequence logo
3,300
3,320
3,340
3,360
AJ489607 consensus ATAGCCATAA TATGATTTAT CTCAACCCTA GCAGAAACAA ACCGGGCCCC CTTCGACCTG ACAGAAGGAG 3360
Quality scores
AJ489607 consensus ATAGCCATAA TATGATTTAT CTCAACCCTA GCAGAAACAA ACCGGGCCCC CTTCGACCTG ACAGAAGGAG 3360
Quality scores
AJ489607 consensus ATAGCCATAA TATGATTTAT CTCAACCCTA GCAGAAACAA ACCGGGCCCC CTTCGACCTG ACAGAAGGAG 3360
Quality scores
AJ489607 consensus ATAGCCATAA TATGATTTAT CTCAACCCTA GCAGAAACAA ACCGGGCCCC CTTCGACCTG ACAGAAGGAG 3360
Quality scores
AJ489607 consensus ATAGCCATAA TATGATTTAT CTCAACCCTA GCAGAAACAA ACCGGGCCCC CTTCGACCTG ACAGAAGGAG 3360
Quality scores
AJ489607 consensus ATAGCCATAA TATGATTTAT CTCAACCCTA GCAGAAACAA ACCGGGCCCC CTTCGACCTG ACAGAAGGAG 3360
Quality scores
AJ489607 consensus ATAGCCATAA TATGATTTAT CTCAACCCTA GCAGAAACAA ACCGGGCCCC CTTCGACCTG ACAGAAGGAG 3360
Quality scores
AJ489607 consensus ATAGCCATAA TATGATTTAT CTCAACCCTA GCAGAAACAA ACCGGGCCCC CTTCGACCTG ACAGAAGGAG 3360
Quality scores
AJ489607 consensus ATAGCCATAA TATGATTTAT CTCAACCCTA GCAGAAACAA ACCGGGCCCC CTTCGACCTG ACAGAAGGAG 3360
Quality scores
Consensus ATAGCCATAA TATGATTTAT CTCAACCCTA GCAGAAACAA ACCGGGCCCC CTTCGACCTG ACAGAAGGAG
Conservation
Sequence logo

3,380 3,400 3,420

AJ489607 consensus AATCAGAATT AGTATCAGGG TTTAACGTAG AATACGCAGC CGGCCCATTC GCGTTATTCT TTATAGCAGA 3430
64 Quality scores 0
AJ489607 consensus AATCAGAATT AGTATCAGGG TTTAACGTAG AATACGCAGC CGGCCCATTC GCGTTATTCT TTATAGCAGA 3430
64 Quality scores 0
AJ489607 consensus AATCAGAATT AGTATCAGGG TTTAACGTAG AATACGCAGC CGGCCCATTC GCGTTATTCT TTATAGCAGA 3430
64 Quality scores 0
AJ489607 consensus AATCAGAATT AGTATCAGGG TTTAACGTAG AATACGCAGC CGGCCCATTC GCGTTATTCT TTATAGCAGA 3430
64 Quality scores 0
AJ489607 consensus AATCAGAATT AGTATCAGGG TTTAACGTAG AATACGCAGC CGGCCCATTC GCGTTATTCT TTATAGCAGA 3430
64 Quality scores 0
AJ489607 consensus AATCAGAATT AGTATCAGGG TTTAACGTAG AATACGCAGC CGGCCCATTC GCGTTATTCT TTATAGCAGA 3430
64 Quality scores 0
AJ489607 consensus AATCAGAATT AGTATCAGGG TTTAACGTAG AATACGCAGC CGGCCCATTC GCGTTATTCT TTATAGCAGA 3430
64 Quality scores 0
AJ489607 consensus AATCAGAATT AGTATCAGGG TTTAACGTAG AATACGCAGC CGGCCCATTC GCGTTATTCT TTATAGCAGA 3430
64 Quality scores 0
AJ489607 consensus AATCAGAATT AGTATCAGGG TTTAACGTAG AATACGCAGC CGGCCCATTC GCGTTATTCT TTATAGCAGA 3430
64 Quality scores 0
Consensus AATCAGAATT AGTATCAGGG TTTAACGTAG AATACGCAGC CGGCCCATTC GCGTTATTCT TTATAGCAGA
100% Conservation 0%
2.0bits Sequence logo 0.0bits AATCAGAATT AGTATCAGGG TTTAACGTAG AATACGCAGC CGGCCCATTC GCGTTATTCT TTATAGCAGA

3,440 3,460 3,480 3,500

AJ489607 consensus GTACACTAAC ATTATTCTAA TAAACGCCCT AACAACTATT ATCTTCCTAG GACCCCTATA CTATATCAAT 3500
64 Quality scores 0
AJ489607 consensus GTACACTAAC ATTATTCTAA TAAACGCCCT AACAACTATT ATCTTCCTAG GACCCCTATA CTATATCAAT 3500
64 Quality scores 0
AJ489607 consensus GTACACTAAC ATTATTCTAA TAAACGCCCT AACAACTATT ATCTTCCTAG GACCCCTATA CTATATCAAT 3500
64 Quality scores 0
AJ489607 consensus GTACACTAAC ATTATTCTAA TAAACGCCCT AACAACTATT ATCTTCCTAG GACCCCTATA CTATATCAAT 3500
64 Quality scores 0
AJ489607 consensus GTACACTAAC ATTATTCTAA TAAACGCCCT AACAACTATT ATCTTCCTAG GACCCCTATA CTATATCAAT 3500
64 Quality scores 0
AJ489607 consensus GTACACTAAC ATTATTCTAA TAAACGCCCT AACAACTATT ATCTTCCTAG GACCCCTATA CTATATCAAT 3500
64 Quality scores 0
AJ489607 consensus GTACACTAAC ATTATTCTAA TAAACGCCCT AACAACTATT ATCTTCCTAG GACCCCTATA CTATATCAAT 3500
64 Quality scores 0
AJ489607 consensus GTACACTAAC ATTATTCTAA TAAACGCCCT AACAACTATT ATCTTCCTAG GACCCCTATA CTATATCAAT 3500
64 Quality scores 0
AJ489607 consensus GTACACTAAC ATTATTCTAA TAAACGCCCT AACAACTATT ATCTTCCTAG GACCCCTATA CTATATCAAT 3500
64 Quality scores 0
Consensus GTACACTAAC ATTATTCTAA TAAACGCCCT AACAACTATT ATCTTCCTAG GACCCCTATA CTATATCAAT
100% Conservation 0%
2.0bits Sequence logo 0.0bits GTACACTAAC ATTATTCTAA TAAACGCCCT AACAACTATT ATCTTCCTAG GACCCCTATA CTATATCAAT

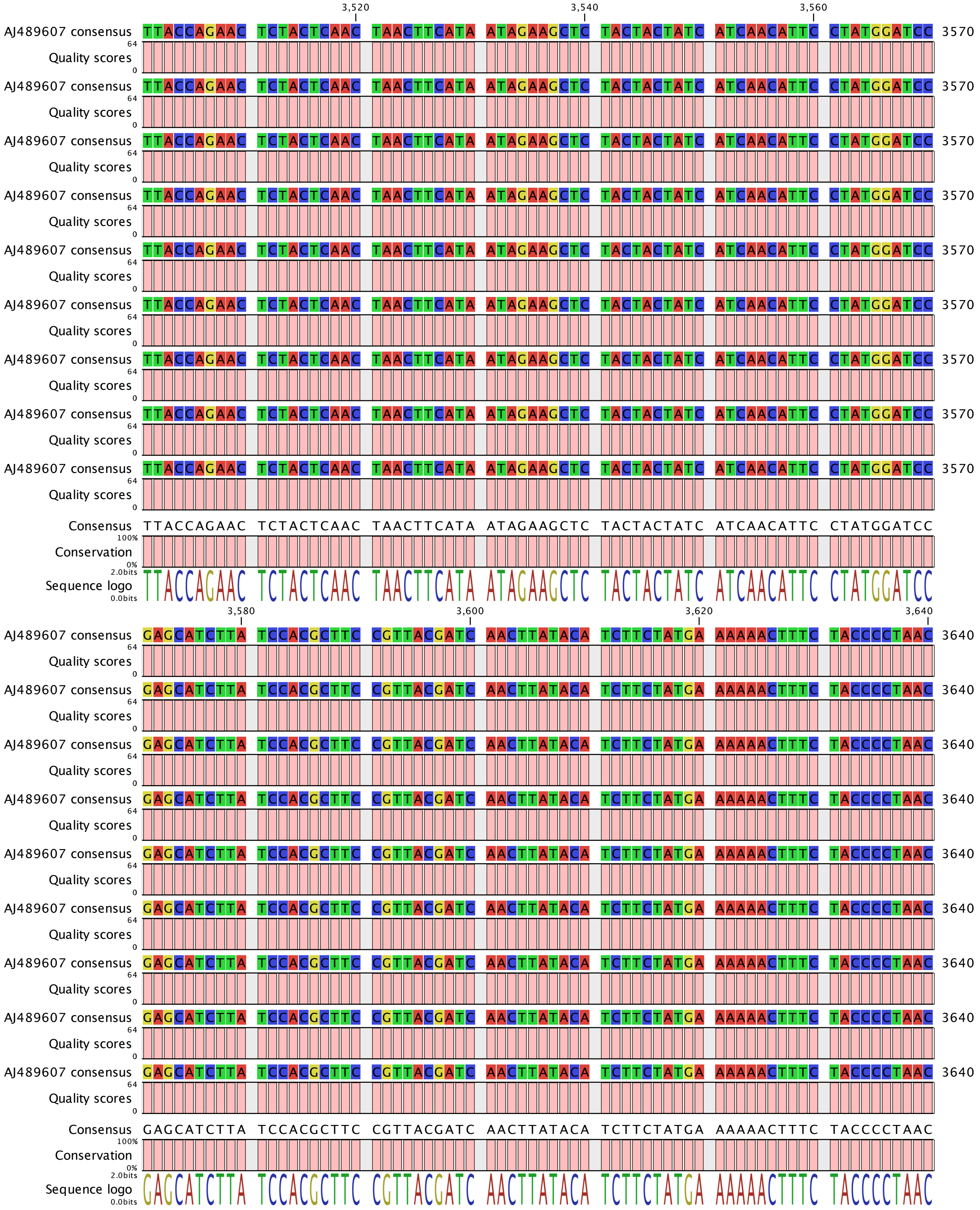
3,520
3,540
3,560
AJ489607 consensus TTACCAGAAC TCTACTCAAC TAACTTCATA ATAGAAGCTC TACTACTATC ATCAACATTC CTATGGATCC 3570
64
Quality scores
0
AJ489607 consensus TTACCAGAAC TCTACTCAAC TAACTTCATA ATAGAAGCTC TACTACTATC ATCAACATTC CTATGGATCC 3570
64
Quality scores
0
AJ489607 consensus TTACCAGAAC TCTACTCAAC TAACTTCATA ATAGAAGCTC TACTACTATC ATCAACATTC CTATGGATCC 3570
64
Quality scores
0
AJ489607 consensus TTACCAGAAC TCTACTCAAC TAACTTCATA ATAGAAGCTC TACTACTATC ATCAACATTC CTATGGATCC 3570
64
Quality scores
0
AJ489607 consensus TTACCAGAAC TCTACTCAAC TAACTTCATA ATAGAAGCTC TACTACTATC ATCAACATTC CTATGGATCC 3570
64
Quality scores
0
AJ489607 consensus TTACCAGAAC TCTACTCAAC TAACTTCATA ATAGAAGCTC TACTACTATC ATCAACATTC CTATGGATCC 3570
64
Quality scores
0
AJ489607 consensus TTACCAGAAC TCTACTCAAC TAACTTCATA ATAGAAGCTC TACTACTATC ATCAACATTC CTATGGATCC 3570
64
Quality scores
0
AJ489607 consensus TTACCAGAAC TCTACTCAAC TAACTTCATA ATAGAAGCTC TACTACTATC ATCAACATTC CTATGGATCC 3570
64
Quality scores
0
AJ489607 consensus TTACCAGAAC TCTACTCAAC TAACTTCATA ATAGAAGCTC TACTACTATC ATCAACATTC CTATGGATCC 3570
64
Quality scores
0
Consensus TTACCAGAAC TCTACTCAAC TAACTTCATA ATAGAAGCTC TACTACTATC ATCAACATTC CTATGGATCC
100%
Conservation
0%
2.0bits
Sequence logo
0.0bits
TTACCAGAAC TCTACTCAAC TAACTTCATA ATAGAAGCTC TACTACTATC ATCAACATTC CTATGGATCC
3,580
3,600
3,620
3,640
AJ489607 consensus GAGCATCTTA TCCACGCTTC CGTTACGATC AACTTATACA TCTTCTATGA AAAAACTTTC TACCCCTAAC 3640
64
Quality scores
0
AJ489607 consensus GAGCATCTTA TCCACGCTTC CGTTACGATC AACTTATACA TCTTCTATGA AAAAACTTTC TACCCCTAAC 3640
64
Quality scores
0
AJ489607 consensus GAGCATCTTA TCCACGCTTC CGTTACGATC AACTTATACA TCTTCTATGA AAAAACTTTC TACCCCTAAC 3640
64
Quality scores
0
AJ489607 consensus GAGCATCTTA TCCACGCTTC CGTTACGATC AACTTATACA TCTTCTATGA AAAAACTTTC TACCCCTAAC 3640
64
Quality scores
0
AJ489607 consensus GAGCATCTTA TCCACGCTTC CGTTACGATC AACTTATACA TCTTCTATGA AAAAACTTTC TACCCCTAAC 3640
64
Quality scores
0
AJ489607 consensus GAGCATCTTA TCCACGCTTC CGTTACGATC AACTTATACA TCTTCTATGA AAAAACTTTC TACCCCTAAC 3640
64
Quality scores
0
AJ489607 consensus GAGCATCTTA TCCACGCTTC CGTTACGATC AACTTATACA TCTTCTATGA AAAAACTTTC TACCCCTAAC 3640
64
Quality scores
0
AJ489607 consensus GAGCATCTTA TCCACGCTTC CGTTACGATC AACTTATACA TCTTCTATGA AAAAACTTTC TACCCCTAAC 3640
64
Quality scores
0
AJ489607 consensus GAGCATCTTA TCCACGCTTC CGTTACGATC AACTTATACA TCTTCTATGA AAAAACTTTC TACCCCTAAC 3640
64
Quality scores
0
Consensus GAGCATCTTA TCCACGCTTC CGTTACGATC AACTTATACA TCTTCTATGA AAAAACTTTC TACCCCTAAC
100%
Conservation
0%
2.0bits
Sequence logo
0.0bits
GAGCATCTTA TCCACGCTTC CGTTACGATC AACTTATACA TCTTCTATGA AAAAACTTTC TACCCCTAAC

3,660 3,680 3,700

AJ489607 consensus ACTAGCATTA TGTATGTGAC ATATTTCTTT ACCAATTTTT ACAGCGGGAG TACCACCATA CATATAGAAA 3710
64 Quality scores 0
AJ489607 consensus ACTAGCATTA TGTATGTGAC ATATTTCTTT ACCAATTTTT ACAGCGGGAG TACCACCATA CATATAGAAA 3710
64 Quality scores 0
AJ489607 consensus ACTAGCATTA TGTATGTGAC ATATTTCTTT ACCAATTTTT ACAGCGGGAG TACCACCATA CATATAGAAA 3710
64 Quality scores 0
AJ489607 consensus ACTAGCATTA TGTATGTGAC ATATTTCTTT ACCAATTTTT ACAGCGGGAG TACCACCATA CATATAGAAA 3710
64 Quality scores 0
AJ489607 consensus ACTAGCATTA TGTATGTGAC ATATTTCTTT ACCAATTTTT ACAGCGGGAG TACCACCATA CATATAGAAA 3710
64 Quality scores 0
AJ489607 consensus ACTAGCATTA TGTATGTGAC ATATTTCTTT ACCAATTTTT ACAGCGGGAG TACCACCATA CATATAGAAA 3710
64 Quality scores 0
AJ489607 consensus ACTAGCATTA TGTATGTGAC ATATTTCTTT ACCAATTTTT ACAGCGGGAG TACCACCATA CATATAGAAA 3710
64 Quality scores 0
AJ489607 consensus ACTAGCATTA TGTATGTGAC ATATTTCTTT ACCAATTTTT ACAGCGGGAG TACCACCATA CATATAGAAA 3710
64 Quality scores 0
AJ489607 consensus ACTAGCATTA TGTATGTGAC ATATTTCTTT ACCAATTTTT ACAGCGGGAG TACCACCATA CATATAGAAA 3710
64 Quality scores 0
Consensus ACTAGCATTA TGTATGTGAC ATATTTCTTT ACCAATTTTT ACAGCGGGAG TACCACCATA CATATAGAAA
100% Conservation 0%
2.0bits Sequence logo 0.0bits ACTAGCATTA TGTATGTGAC ATATTTCTTT ACCAATTTTT ACAGCGGGAG TACCACCATA CATATAGAAA

3,720 3,740 3,760 3,780

AJ489607 consensus TATGTCTGAT AAAAGAATTA CTTTGATAGA GTAAATTATA GAGGTTCAAG CCCTCTTATT TCTAGGACAA 3780
64 Quality scores 0
AJ489607 consensus TATGTCTGAT AAAAGAATTA CTTTGATAGA GTAAATTATA GAGGTTCAAG CCCTCTTATT TCTAGGACAA 3780
64 Quality scores 0
AJ489607 consensus TATGTCTGAT AAAAGAATTA CTTTGATAGA GTAAATTATA GAGGTTCAAG CCCTCTTATT TCTAGGACAA 3780
64 Quality scores 0
AJ489607 consensus TATGTCTGAT AAAAGAATTA CTTTGATAGA GTAAATTATA GAGGTTCAAG CCCTCTTATT TCTAGGACAA 3780
64 Quality scores 0
AJ489607 consensus TATGTCTGAT AAAAGAATTA CTTTGATAGA GTAAATTATA GAGGTTCAAG CCCTCTTATT TCTAGGACAA 3780
64 Quality scores 0
AJ489607 consensus TATGTCTGAT AAAAGAATTA CTTTGATAGA GTAAATTATA GAGGTTCAAG CCCTCTTATT TCTAGGACAA 3780
64 Quality scores 0
AJ489607 consensus TATGTCTGAT AAAAGAATTA CTTTGATAGA GTAAATTATA GAGGTTCAAG CCCTCTTATT TCTAGGACAA 3780
64 Quality scores 0
AJ489607 consensus TATGTCTGAT AAAAGAATTA CTTTGATAGA GTAAATTATA GAGGTTCAAG CCCTCTTATT TCTAGGACAA 3780
64 Quality scores 0
AJ489607 consensus TATGTCTGAT AAAAGAATTA CTTTGATAGA GTAAATTATA GAGGTTCAAG CCCTCTTATT TCTAGGACAA 3780
64 Quality scores 0
Consensus TATGTCTGAT AAAAGAATTA CTTTGATAGA GTAAATTATA GAGGTTCAAG CCCTCTTATT TCTAGGACAA
100% Conservation 0%
2.0bits Sequence logo 0.0bits TATGTCTGAT AAAAGAATTA CTTTGATAGA GTAAATTATA GAGGTTCAAG CCCTCTTATT TCTAGGACAA

3,800 3,820 3,840

AJ489607 consensus TAGGAATTGA ACCTACACTT AAGAATTCAA AATTCTCCGT GCTACCTAAA CACCTTATCC TAATAGTAAG 3850
64
Quality scores
0
AJ489607 consensus TAGGAATTGA ACCTACACTT AAGAATTCAA AATTCTCCGT GCTACCTAAA CACCTTATCC TAATAGTAAG 3850
64
Quality scores
0
AJ489607 consensus TAGGAATTGA ACCTACACTT AAGAATTCAA AATTCTCCGT GCTACCTAAA CACCTTATCC TAATAGTAAG 3850
64
Quality scores
0
AJ489607 consensus TAGGAATTGA ACCTACACTT AAGAATTCAA AATTCTCCGT GCTACCTAAA CACCTTATCC TAATAGTAAG 3850
64
Quality scores
0
AJ489607 consensus TAGGAATTGA ACCTACACTT AAGAATTCAA AATTCTCCGT GCTACCTAAA CACCTTATCC TAATAGTAAG 3850
64
Quality scores
0
AJ489607 consensus TAGGAATTGA ACCTACACTT AAGAATTCAA AATTCTCCGT GCTACCTAAA CACCTTATCC TAATAGTAAG 3850
64
Quality scores
0
AJ489607 consensus TAGGAATTGA ACCTACACTT AAGAATTCAA AATTCTCCGT GCTACCTAAA CACCTTATCC TAATAGTAAG 3850
64
Quality scores
0
AJ489607 consensus TAGGAATTGA ACCTACACTT AAGAATTCAA AATTCTCCGT GCTACCTAAA CACCTTATCC TAATAGTAAG 3850
64
Quality scores
0
AJ489607 consensus TAGGAATTGA ACCTACACTT AAGAATTCAA AATTCTCCGT GCTACCTAAA CACCTTATCC TAATAGTAAG 3850
64
Quality scores
0
Consensus TAGGAATTGA ACCTACACTT AAGAATTCAA AATTCTCCGT GCTACCTAAA CACCTTATCC TAATAGTAAG
100%
Conservation
0%
2.0bits
Sequence logo TAGGAATTGA ACCTACACTT AAGAATTCAA AATTCTCCGT GCTACCTAAA CACCTTATCC TAATAGTAAG
0.0bits

3,860 3,880 3,900 3,920

AJ489607 consensus GTCAGCTAAT TAAGCTATCG GGCCCATACC CCGAAAACGT TGGTTTAAAT CCTTCCCGTA CTAATAAATC 3920
64
Quality scores
0
AJ489607 consensus GTCAGCTAAT TAAGCTATCG GGCCCATACC CCGAAAACGT TGGTTTAAAT CCTTCCCGTA CTAATAAATC 3920
64
Quality scores
0
AJ489607 consensus GTCAGCTAAT TAAGCTATCG GGCCCATACC CCGAAAACGT TGGTTTAAAT CCTTCCCGTA CTAATAAATC 3920
64
Quality scores
0
AJ489607 consensus GTCAGCTAAT TAAGCTATCG GGCCCATACC CCGAAAACGT TGGTTTAAAT CCTTCCCGTA CTAATAAATC 3920
64
Quality scores
0
AJ489607 consensus GTCAGCTAAT TAAGCTATCG GGCCCATACC CCGAAAACGT TGGTTTAAAT CCTTCCCGTA CTAATAAATC 3920
64
Quality scores
0
AJ489607 consensus GTCAGCTAAT TAAGCTATCG GGCCCATACC CCGAAAACGT TGGTTTAAAT CCTTCCCGTA CTAATAAATC 3920
64
Quality scores
0
AJ489607 consensus GTCAGCTAAT TAAGCTATCG GGCCCATACC CCGAAAACGT TGGTTTAAAT CCTTCCCGTA CTAATAAATC 3920
64
Quality scores
0
AJ489607 consensus GTCAGCTAAT TAAGCTATCG GGCCCATACC CCGAAAACGT TGGTTTAAAT CCTTCCCGTA CTAATAAATC 3920
64
Quality scores
0
AJ489607 consensus GTCAGCTAAT TAAGCTATCG GGCCCATACC CCGAAAACGT TGGTTTAAAT CCTTCCCGTA CTAATAAATC 3920
64
Quality scores
0
Consensus GTCAGCTAAT TAAGCTATCG GGCCCATACC CCGAAAACGT TGGTTTAAAT CCTTCCCGTA CTAATAAATC
100%
Conservation
0%
2.0bits
Sequence logo GTCAGCTAAT TAAGCTATCG GGCCCATACC CCGAAAACGT TGGTTTAAAT CCTTCCCGTA CTAATAAATC
0.0bits

3,940 3,960 3,980

AJ489607 consensus CTATCACCCT TGCCATCATC TACTTCACAA TCTTCTTAGG TCCTGTAATC ACAATATCCA GCACCAACCT 3990
64 Quality scores 0
AJ489607 consensus CTATCACCCT TGCCATCATC TACTTCACAA TCTTCTTAGG TCCTGTAATC ACAATATCCA GCACCAACCT 3990
64 Quality scores 0
AJ489607 consensus CTATCACCCT TGCCATCATC TACTTCACAA TCTTCTTAGG TCCTGTAATC ACAATATCCA GCACCAACCT 3990
64 Quality scores 0
AJ489607 consensus CTATCACCCT TGCCATCATC TACTTCACAA TCTTCTTAGG TCCTGTAATC ACAATATCCA GCACCAACCT 3990
64 Quality scores 0
AJ489607 consensus CTATCACCCT TGCCATCATC TACTTCACAA TCTTCTTAGG TCCTGTAATC ACAATATCCA GCACCAACCT 3990
64 Quality scores 0
AJ489607 consensus CTATCACCCT TGCCATCATC TACTTCACAA TCTTCTTAGG TCCTGTAATC ACAATATCCA GCACCAACCT 3990
64 Quality scores 0
AJ489607 consensus CTATCACCCT TGCCATCATC TACTTCACAA TCTTCTTAGG TCCTGTAATC ACAATATCCA GCACCAACCT 3990
64 Quality scores 0
AJ489607 consensus CTATCACCCT TGCCATCATC TACTTCACAA TCTTCTTAGG TCCTGTAATC ACAATATCCA GCACCAACCT 3990
64 Quality scores 0
AJ489607 consensus CTATCACCCT TGCCATCATC TACTTCACAA TCTTCTTAGG TCCTGTAATC ACAATATCCA GCACCAACCT 3990
64 Quality scores 0
Consensus CTATCACCCT TGCCATCATC TACTTCACAA TCTTCTTAGG TCCTGTAATC ACAATATCCA GCACCAACCT
100% Conservation 0%
2.0bits Sequence logo 0.0bits CTATCACCCT TGCCATCATC TACTTCACAA TCTTCTTAGG TCCTGTAATC ACAATATCCA GCACCAACCT

4,000 4,020 4,040 4,060

AJ489607 consensus AATACTAATA TGAGTAGGCC TGGAATTCAG CCTACTAGCA ATTATCCCCA TACTAATCAA CAAAAAAAAC 4060
64 Quality scores 0
AJ489607 consensus AATACTAATA TGAGTAGGCC TGGAATTCAG CCTACTAGCA ATTATCCCCA TACTAATCAA CAAAAAAAAC 4060
64 Quality scores 0
AJ489607 consensus AATACTAATA TGAGTAGGCC TGGAATTCAG CCTACTAGCA ATTATCCCCA TACTAATCAA CAAAAAAAAC 4060
64 Quality scores 0
AJ489607 consensus AATACTAATA TGAGTAGGCC TGGAATTCAG CCTACTAGCA ATTATCCCCA TACTAATCAA CAAAAAAAAC 4060
64 Quality scores 0
AJ489607 consensus AATACTAATA TGAGTAGGCC TGGAATTCAG CCTACTAGCA ATTATCCCCA TACTAATCAA CAAAAAAAAC 4060
64 Quality scores 0
AJ489607 consensus AATACTAATA TGAGTAGGCC TGGAATTCAG CCTACTAGCA ATTATCCCCA TACTAATCAA CAAAAAAAAC 4060
64 Quality scores 0
AJ489607 consensus AATACTAATA TGAGTAGGCC TGGAATTCAG CCTACTAGCA ATTATCCCCA TACTAATCAA CAAAAAAAAC 4060
64 Quality scores 0
AJ489607 consensus AATACTAATA TGAGTAGGCC TGGAATTCAG CCTACTAGCA ATTATCCCCA TACTAATCAA CAAAAAAAAC 4060
64 Quality scores 0
AJ489607 consensus AATACTAATA TGAGTAGGCC TGGAATTCAG CCTACTAGCA ATTATCCCCA TACTAATCAA CAAAAAAAAC 4060
64 Quality scores 0
Consensus AATACTAATA TGAGTAGGCC TGGAATTCAG CCTACTAGCA ATTATCCCCA TACTAATCAA CAAAAAAAAC
100% Conservation 0%
2.0bits Sequence logo 0.0bits AATACTAATA TGAGTAGGCC TGGAATTCAG CCTACTAGCA ATTATCCCCA TACTAATCAA CAAAAAAAAC

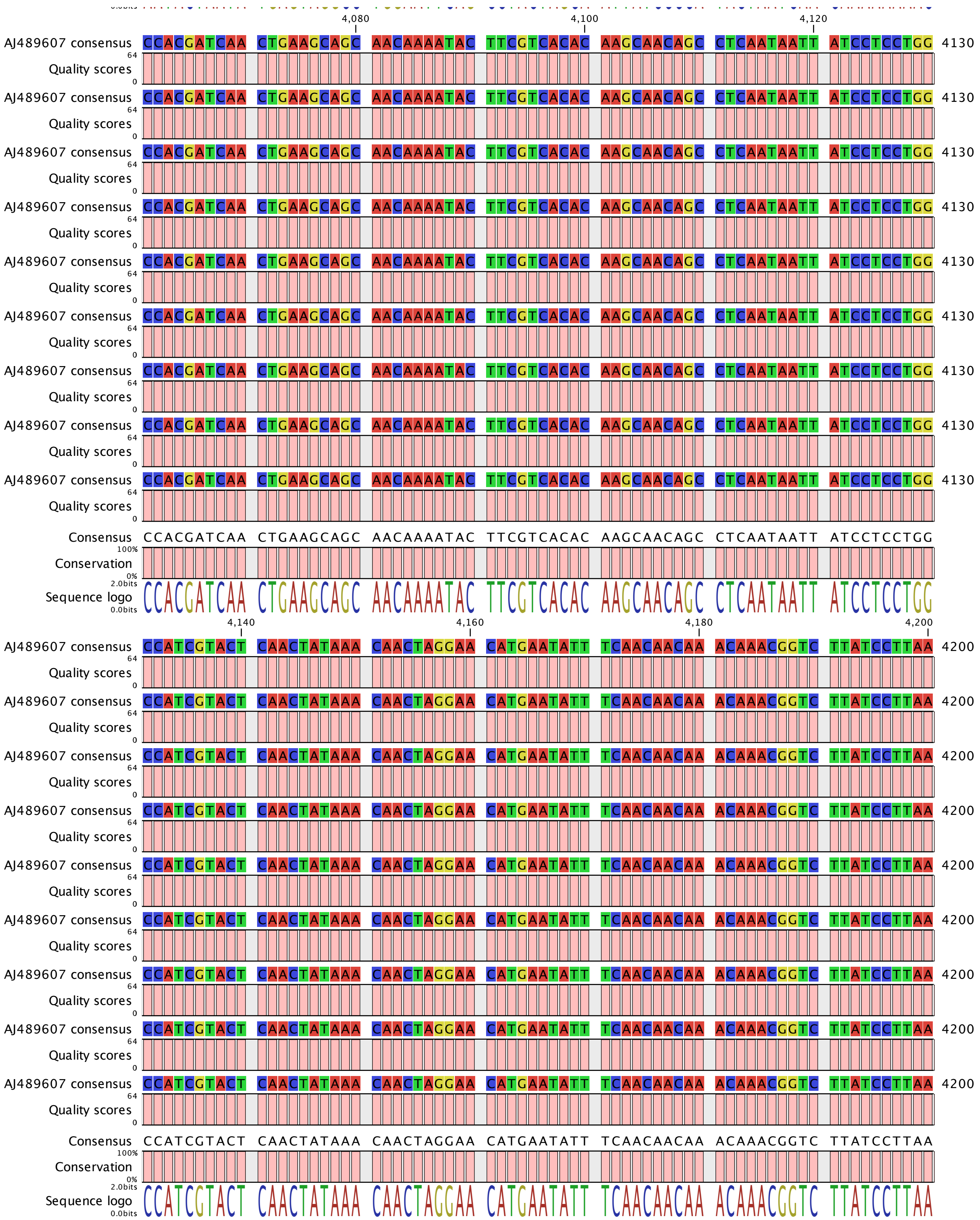
4,080
4,100
4,120
AJ489607 consensus CCACGATCAA CTGAAGCAGC AACAAAATAC TTCGTCACAC AAGCAACAGC CTCAATAATT ATCCTCCTGG 4130
64
Quality scores
0
AJ489607 consensus CCACGATCAA CTGAAGCAGC AACAAAATAC TTCGTCACAC AAGCAACAGC CTCAATAATT ATCCTCCTGG 4130
64
Quality scores
0
AJ489607 consensus CCACGATCAA CTGAAGCAGC AACAAAATAC TTCGTCACAC AAGCAACAGC CTCAATAATT ATCCTCCTGG 4130
64
Quality scores
0
AJ489607 consensus CCACGATCAA CTGAAGCAGC AACAAAATAC TTCGTCACAC AAGCAACAGC CTCAATAATT ATCCTCCTGG 4130
64
Quality scores
0
AJ489607 consensus CCACGATCAA CTGAAGCAGC AACAAAATAC TTCGTCACAC AAGCAACAGC CTCAATAATT ATCCTCCTGG 4130
64
Quality scores
0
AJ489607 consensus CCACGATCAA CTGAAGCAGC AACAAAATAC TTCGTCACAC AAGCAACAGC CTCAATAATT ATCCTCCTGG 4130
64
Quality scores
0
AJ489607 consensus CCACGATCAA CTGAAGCAGC AACAAAATAC TTCGTCACAC AAGCAACAGC CTCAATAATT ATCCTCCTGG 4130
64
Quality scores
0
AJ489607 consensus CCACGATCAA CTGAAGCAGC AACAAAATAC TTCGTCACAC AAGCAACAGC CTCAATAATT ATCCTCCTGG 4130
64
Quality scores
0
AJ489607 consensus CCACGATCAA CTGAAGCAGC AACAAAATAC TTCGTCACAC AAGCAACAGC CTCAATAATT ATCCTCCTGG 4130
64
Quality scores
0
Consensus CCACGATCAA CTGAAGCAGC AACAAAATAC TTCGTCACAC AAGCAACAGC CTCAATAATT ATCCTCCTGG
100%
Conservation
0%
2.0bits
Sequence logo CCACGATCAA CTGAAGCAGC AACAAAATAC TTCGTCACAC AAGCAACAGC CTCAATAATT ATCCTCCTGG
0.0bits
4,140
4,160
4,180
4,200
AJ489607 consensus CCATCGTACT CAACTATAAA CAACTAGGAA CATGAATATT TCAACAACAA ACAAACGGTC TTATCCTTAA 4200
64
Quality scores
0
AJ489607 consensus CCATCGTACT CAACTATAAA CAACTAGGAA CATGAATATT TCAACAACAA ACAAACGGTC TTATCCTTAA 4200
64
Quality scores
0
AJ489607 consensus CCATCGTACT CAACTATAAA CAACTAGGAA CATGAATATT TCAACAACAA ACAAACGGTC TTATCCTTAA 4200
64
Quality scores
0
AJ489607 consensus CCATCGTACT CAACTATAAA CAACTAGGAA CATGAATATT TCAACAACAA ACAAACGGTC TTATCCTTAA 4200
64
Quality scores
0
AJ489607 consensus CCATCGTACT CAACTATAAA CAACTAGGAA CATGAATATT TCAACAACAA ACAAACGGTC TTATCCTTAA 4200
64
Quality scores
0
AJ489607 consensus CCATCGTACT CAACTATAAA CAACTAGGAA CATGAATATT TCAACAACAA ACAAACGGTC TTATCCTTAA 4200
64
Quality scores
0
AJ489607 consensus CCATCGTACT CAACTATAAA CAACTAGGAA CATGAATATT TCAACAACAA ACAAACGGTC TTATCCTTAA 4200
64
Quality scores
0
AJ489607 consensus CCATCGTACT CAACTATAAA CAACTAGGAA CATGAATATT TCAACAACAA ACAAACGGTC TTATCCTTAA 4200
64
Quality scores
0
AJ489607 consensus CCATCGTACT CAACTATAAA CAACTAGGAA CATGAATATT TCAACAACAA ACAAACGGTC TTATCCTTAA 4200
64
Quality scores
0
Consensus CCATCGTACT CAACTATAAA CAACTAGGAA CATGAATATT TCAACAACAA ACAAACGGTC TTATCCTTAA
100%
Conservation
0%
2.0bits
Sequence logo CCATCGTACT CAACTATAAA CAACTAGGAA CATGAATATT TCAACAACAA ACAAACGGTC TTATCCTTAA
0.0bits

4,220 4,240 4,260

AJ489607 consensus CATAACATTA ATAGCCCTAT CCATAAAACT AGGCCTCGCC CCATTCCACT TCTGATTACC AGAAGTAACT 4270
Quality scores
AJ489607 consensus CATAACATTA ATAGCCCTAT CCATAAAACT AGGCCTCGCC CCATTCCACT TCTGATTACC AGAAGTAACT 4270
Quality scores
AJ489607 consensus CATAACATTA ATAGCCCTAT CCATAAAACT AGGCCTCGCC CCATTCCACT TCTGATTACC AGAAGTAACT 4270
Quality scores
AJ489607 consensus CATAACATTA ATAGCCCTAT CCATAAAACT AGGCCTCGCC CCATTCCACT TCTGATTACC AGAAGTAACT 4270
Quality scores
AJ489607 consensus CATAACATTA ATAGCCCTAT CCATAAAACT AGGCCTCGCC CCATTCCACT TCTGATTACC AGAAGTAACT 4270
Quality scores
AJ489607 consensus CATAACATTA ATAGCCCTAT CCATAAAACT AGGCCTCGCC CCATTCCACT TCTGATTACC AGAAGTAACT 4270
Quality scores
AJ489607 consensus CATAACATTA ATAGCCCTAT CCATAAAACT AGGCCTCGCC CCATTCCACT TCTGATTACC AGAAGTAACT 4270
Quality scores
AJ489607 consensus CATAACATTA ATAGCCCTAT CCATAAAACT AGGCCTCGCC CCATTCCACT TCTGATTACC AGAAGTAACT 4270
Quality scores
AJ489607 consensus CATAACATTA ATAGCCCTAT CCATAAAACT AGGCCTCGCC CCATTCCACT TCTGATTACC AGAAGTAACT 4270
Quality scores
Consensus CATAACATTA ATAGCCCTAT CCATAAAACT AGGCCTCGCC CCATTCCACT TCTGATTACC AGAAGTAACT
Conservation
Sequence logo CATAACATTA ATAGCCCTAT CCATAAAACT AGGCCTCGCC CCATTCCACT TCTGATTACC AGAAGTAACT

4,280 4,300 4,320 4,340

AJ489607 consensus CAAGGGATCC CACTGCACAT AGGACTTATT CTTCTTACAT GACAAAAAAT TGCTCCCCTA TCAATTTTAA 4340
Quality scores
AJ489607 consensus CAAGGGATCC CACTGCACAT AGGACTTATT CTTCTTACAT GACAAAAAAT TGCTCCCCTA TCAATTTTAA 4340
Quality scores
AJ489607 consensus CAAGGGATCC CACTGCACAT AGGACTTATT CTTCTTACAT GACAAAAAAT TGCTCCCCTA TCAATTTTAA 4340
Quality scores
AJ489607 consensus CAAGGGATCC CACTGCACAT AGGACTTATT CTTCTTACAT GACAAAAAAT TGCTCCCCTA TCAATTTTAA 4340
Quality scores
AJ489607 consensus CAAGGGATCC CACTGCACAT AGGACTTATT CTTCTTACAT GACAAAAAAT TGCTCCCCTA TCAATTTTAA 4340
Quality scores
AJ489607 consensus CAAGGGATCC CACTGCACAT AGGACTTATT CTTCTTACAT GACAAAAAAT TGCTCCCCTA TCAATTTTAA 4340
Quality scores
AJ489607 consensus CAAGGGATCC CACTGCACAT AGGACTTATT CTTCTTACAT GACAAAAAAT TGCTCCCCTA TCAATTTTAA 4340
Quality scores
AJ489607 consensus CAAGGGATCC CACTGCACAT AGGACTTATT CTTCTTACAT GACAAAAAAT TGCTCCCCTA TCAATTTTAA 4340
Quality scores
AJ489607 consensus CAAGGGATCC CACTGCACAT AGGACTTATT CTTCTTACAT GACAAAAAAT TGCTCCCCTA TCAATTTTAA 4340
Quality scores
Consensus CAAGGGATCC CACTGCACAT AGGACTTATT CTTCTTACAT GACAAAAAAT TGCTCCCCTA TCAATTTTAA
Conservation
Sequence logo CAAGGGATCC CACTGCACAT AGGACTTATT CTTCTTACAT GACAAAAAAT TGCTCCCCTA TCAATTTTAA

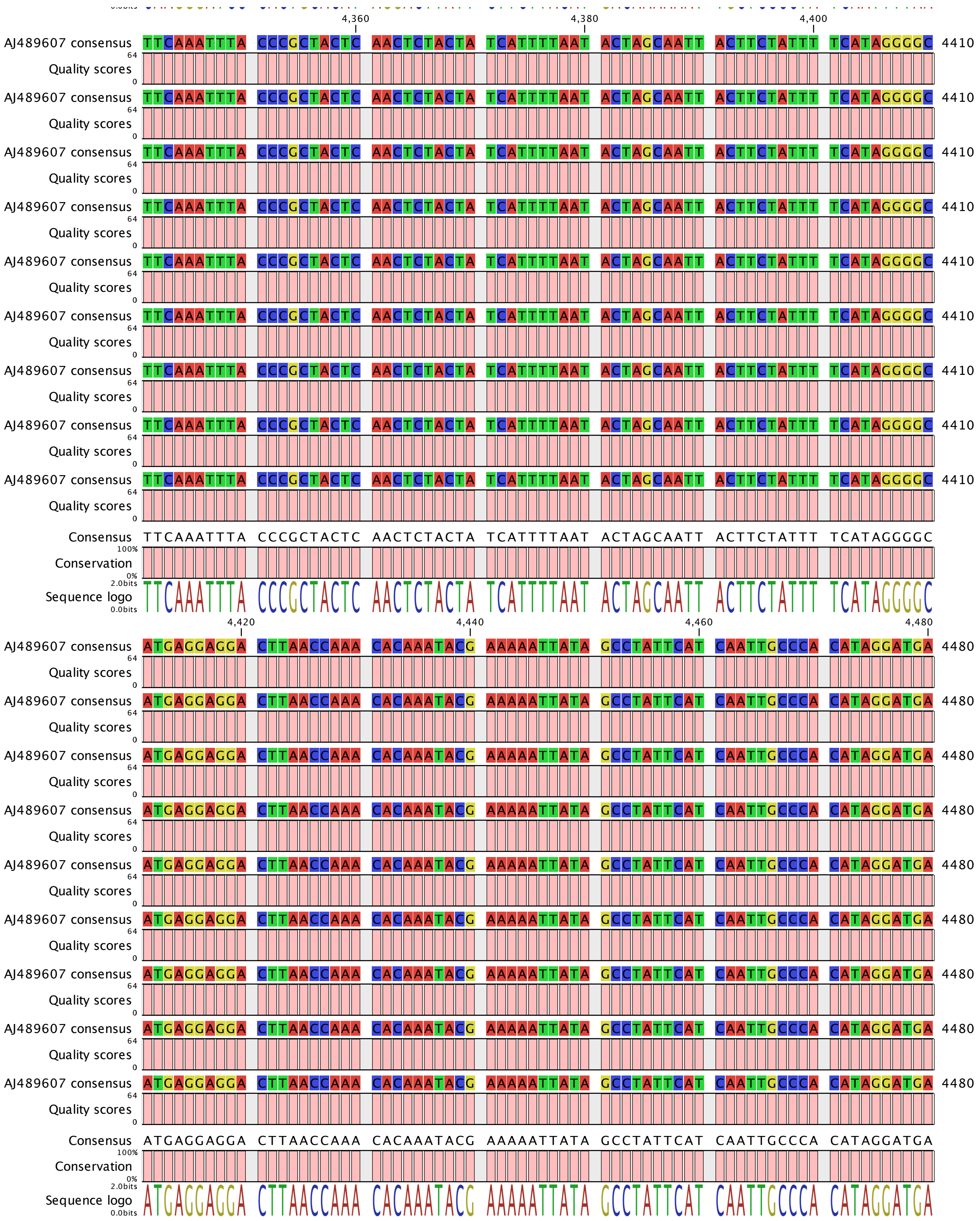
4,360 4,380 4,400
AJ489607 consensus TTCAAATTTA CCCGCTACTC AACTCTACTA TCATTTTAAT ACTAGCAATT ACTTCTATTT TCATAGGGGC 4410
Quality scores
AJ489607 consensus TTCAAATTTA CCCGCTACTC AACTCTACTA TCATTTTAAT ACTAGCAATT ACTTCTATTT TCATAGGGGC 4410
Quality scores
AJ489607 consensus TTCAAATTTA CCCGCTACTC AACTCTACTA TCATTTTAAT ACTAGCAATT ACTTCTATTT TCATAGGGGC 4410
Quality scores
AJ489607 consensus TTCAAATTTA CCCGCTACTC AACTCTACTA TCATTTTAAT ACTAGCAATT ACTTCTATTT TCATAGGGGC 4410
Quality scores
AJ489607 consensus TTCAAATTTA CCCGCTACTC AACTCTACTA TCATTTTAAT ACTAGCAATT ACTTCTATTT TCATAGGGGC 4410
Quality scores
AJ489607 consensus TTCAAATTTA CCCGCTACTC AACTCTACTA TCATTTTAAT ACTAGCAATT ACTTCTATTT TCATAGGGGC 4410
Quality scores
AJ489607 consensus TTCAAATTTA CCCGCTACTC AACTCTACTA TCATTTTAAT ACTAGCAATT ACTTCTATTT TCATAGGGGC 4410
Quality scores
AJ489607 consensus TTCAAATTTA CCCGCTACTC AACTCTACTA TCATTTTAAT ACTAGCAATT ACTTCTATTT TCATAGGGGC 4410
Quality scores
AJ489607 consensus TTCAAATTTA CCCGCTACTC AACTCTACTA TCATTTTAAT ACTAGCAATT ACTTCTATTT TCATAGGGGC 4410
Quality scores
Consensus TTCAAATTTA CCCGCTACTC AACTCTACTA TCATTTTAAT ACTAGCAATT ACTTCTATTT TCATAGGGGC
100%
Conservation
0%
2.0bits
Sequence logo
0.0bits
4,420 4,440 4,460 4,480
AJ489607 consensus ATGAGGAGGA CTTAACCAAA CACAAATACG AAAAATTATA GCCTATTCAT CAATTGCCCA CATAGGATGA 4480
Quality scores
AJ489607 consensus ATGAGGAGGA CTTAACCAAA CACAAATACG AAAAATTATA GCCTATTCAT CAATTGCCCA CATAGGATGA 4480
Quality scores
AJ489607 consensus ATGAGGAGGA CTTAACCAAA CACAAATACG AAAAATTATA GCCTATTCAT CAATTGCCCA CATAGGATGA 4480
Quality scores
AJ489607 consensus ATGAGGAGGA CTTAACCAAA CACAAATACG AAAAATTATA GCCTATTCAT CAATTGCCCA CATAGGATGA 4480
Quality scores
AJ489607 consensus ATGAGGAGGA CTTAACCAAA CACAAATACG AAAAATTATA GCCTATTCAT CAATTGCCCA CATAGGATGA 4480
Quality scores
AJ489607 consensus ATGAGGAGGA CTTAACCAAA CACAAATACG AAAAATTATA GCCTATTCAT CAATTGCCCA CATAGGATGA 4480
Quality scores
AJ489607 consensus ATGAGGAGGA CTTAACCAAA CACAAATACG AAAAATTATA GCCTATTCAT CAATTGCCCA CATAGGATGA 4480
Quality scores
AJ489607 consensus ATGAGGAGGA CTTAACCAAA CACAAATACG AAAAATTATA GCCTATTCAT CAATTGCCCA CATAGGATGA 4480
Quality scores
AJ489607 consensus ATGAGGAGGA CTTAACCAAA CACAAATACG AAAAATTATA GCCTATTCAT CAATTGCCCA CATAGGATGA 4480
Quality scores
Consensus ATGAGGAGGA CTTAACCAAA CACAAATACG AAAAATTATA GCCTATTCAT CAATTGCCCA CATAGGATGA
100%
Conservation
0%
2.0bits
Sequence logo
0.0bits

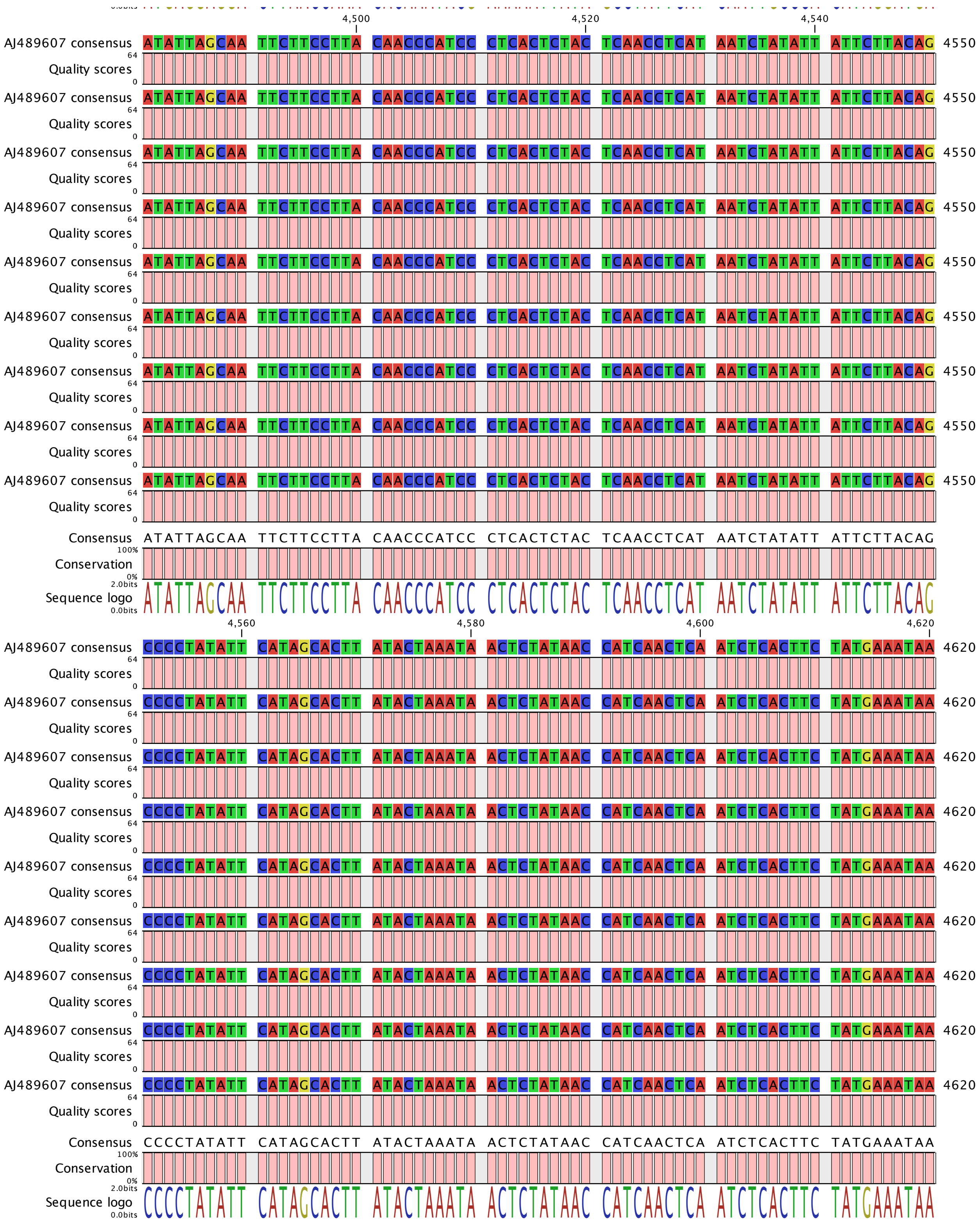
4,500
4,520
4,540
AJ489607 consensus ATATTAGCAA TTCTTCCTTA CAACCCATCC CTCACTCTAC TCAACCTCAT AATCTATATT ATTCTTACAG 4550
Quality scores
AJ489607 consensus ATATTAGCAA TTCTTCCTTA CAACCCATCC CTCACTCTAC TCAACCTCAT AATCTATATT ATTCTTACAG 4550
Quality scores
AJ489607 consensus ATATTAGCAA TTCTTCCTTA CAACCCATCC CTCACTCTAC TCAACCTCAT AATCTATATT ATTCTTACAG 4550
Quality scores
AJ489607 consensus ATATTAGCAA TTCTTCCTTA CAACCCATCC CTCACTCTAC TCAACCTCAT AATCTATATT ATTCTTACAG 4550
Quality scores
AJ489607 consensus ATATTAGCAA TTCTTCCTTA CAACCCATCC CTCACTCTAC TCAACCTCAT AATCTATATT ATTCTTACAG 4550
Quality scores
AJ489607 consensus ATATTAGCAA TTCTTCCTTA CAACCCATCC CTCACTCTAC TCAACCTCAT AATCTATATT ATTCTTACAG 4550
Quality scores
AJ489607 consensus ATATTAGCAA TTCTTCCTTA CAACCCATCC CTCACTCTAC TCAACCTCAT AATCTATATT ATTCTTACAG 4550
Quality scores
AJ489607 consensus ATATTAGCAA TTCTTCCTTA CAACCCATCC CTCACTCTAC TCAACCTCAT AATCTATATT ATTCTTACAG 4550
Quality scores
AJ489607 consensus ATATTAGCAA TTCTTCCTTA CAACCCATCC CTCACTCTAC TCAACCTCAT AATCTATATT ATTCTTACAG 4550
Quality scores
Consensus ATATTAGCAA TTCTTCCTTA CAACCCATCC CTCACTCTAC TCAACCTCAT AATCTATATT ATTCTTACAG
Conservation
Sequence logo
4,560
4,580
4,600
4,620
AJ489607 consensus CCCCTATATT CATAGCACTT ATACTAAATA ACTCTATAAC CATCAACTCA ATCTCACTTC TATGAAATAA 4620
Quality scores
AJ489607 consensus CCCCTATATT CATAGCACTT ATACTAAATA ACTCTATAAC CATCAACTCA ATCTCACTTC TATGAAATAA 4620
Quality scores
AJ489607 consensus CCCCTATATT CATAGCACTT ATACTAAATA ACTCTATAAC CATCAACTCA ATCTCACTTC TATGAAATAA 4620
Quality scores
AJ489607 consensus CCCCTATATT CATAGCACTT ATACTAAATA ACTCTATAAC CATCAACTCA ATCTCACTTC TATGAAATAA 4620
Quality scores
AJ489607 consensus CCCCTATATT CATAGCACTT ATACTAAATA ACTCTATAAC CATCAACTCA ATCTCACTTC TATGAAATAA 4620
Quality scores
AJ489607 consensus CCCCTATATT CATAGCACTT ATACTAAATA ACTCTATAAC CATCAACTCA ATCTCACTTC TATGAAATAA 4620
Quality scores
AJ489607 consensus CCCCTATATT CATAGCACTT ATACTAAATA ACTCTATAAC CATCAACTCA ATCTCACTTC TATGAAATAA 4620
Quality scores
AJ489607 consensus CCCCTATATT CATAGCACTT ATACTAAATA ACTCTATAAC CATCAACTCA ATCTCACTTC TATGAAATAA 4620
Quality scores
AJ489607 consensus CCCCTATATT CATAGCACTT ATACTAAATA ACTCTATAAC CATCAACTCA ATCTCACTTC TATGAAATAA 4620
Quality scores
Consensus CCCCTATATT CATAGCACTT ATACTAAATA ACTCTATAAC CATCAACTCA ATCTCACTTC TATGAAATAA
Conservation
Sequence logo

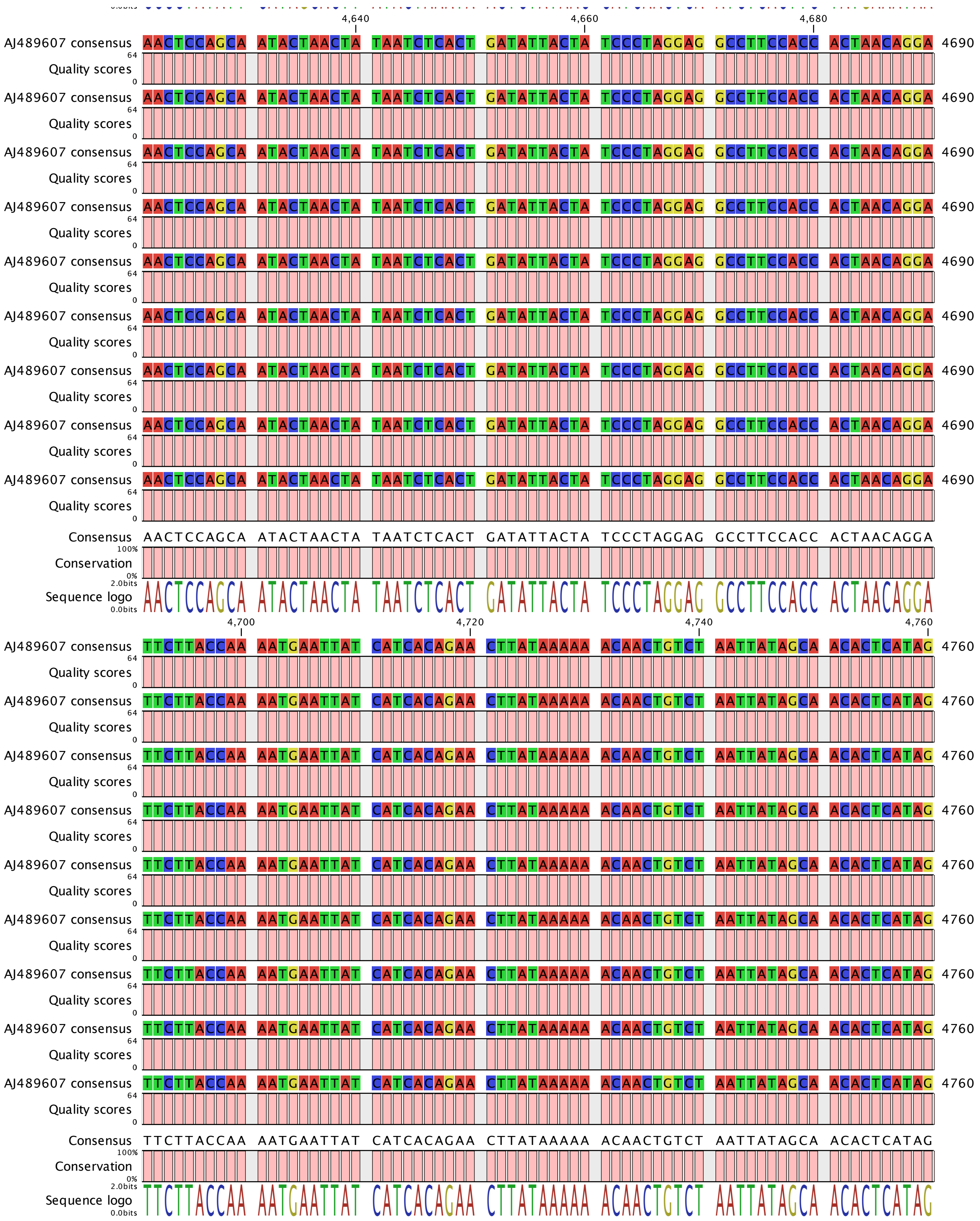
4,640
4,660
4,680
AJ489607 consensus AACTCCAGCA ATACTAACTA TAATCTCACT GATATTACTA TCCCTAGGAG GCCTTCCACC ACTAACAGGA 4690
Quality scores
AJ489607 consensus AACTCCAGCA ATACTAACTA TAATCTCACT GATATTACTA TCCCTAGGAG GCCTTCCACC ACTAACAGGA 4690
Quality scores
AJ489607 consensus AACTCCAGCA ATACTAACTA TAATCTCACT GATATTACTA TCCCTAGGAG GCCTTCCACC ACTAACAGGA 4690
Quality scores
AJ489607 consensus AACTCCAGCA ATACTAACTA TAATCTCACT GATATTACTA TCCCTAGGAG GCCTTCCACC ACTAACAGGA 4690
Quality scores
AJ489607 consensus AACTCCAGCA ATACTAACTA TAATCTCACT GATATTACTA TCCCTAGGAG GCCTTCCACC ACTAACAGGA 4690
Quality scores
AJ489607 consensus AACTCCAGCA ATACTAACTA TAATCTCACT GATATTACTA TCCCTAGGAG GCCTTCCACC ACTAACAGGA 4690
Quality scores
AJ489607 consensus AACTCCAGCA ATACTAACTA TAATCTCACT GATATTACTA TCCCTAGGAG GCCTTCCACC ACTAACAGGA 4690
Quality scores
AJ489607 consensus AACTCCAGCA ATACTAACTA TAATCTCACT GATATTACTA TCCCTAGGAG GCCTTCCACC ACTAACAGGA 4690
Quality scores
AJ489607 consensus AACTCCAGCA ATACTAACTA TAATCTCACT GATATTACTA TCCCTAGGAG GCCTTCCACC ACTAACAGGA 4690
Quality scores
Consensus AACTCCAGCA ATACTAACTA TAATCTCACT GATATTACTA TCCCTAGGAG GCCTTCCACC ACTAACAGGA
Conservation
Sequence logo
4,700
4,720
4,740
4,760
AJ489607 consensus TTCTTACCAA AATGAATTAT CATCACAGAA CTTATAAAAA ACAACTGTCT AATTATAGCA ACACTCATAG 4760
Quality scores
AJ489607 consensus TTCTTACCAA AATGAATTAT CATCACAGAA CTTATAAAAA ACAACTGTCT AATTATAGCA ACACTCATAG 4760
Quality scores
AJ489607 consensus TTCTTACCAA AATGAATTAT CATCACAGAA CTTATAAAAA ACAACTGTCT AATTATAGCA ACACTCATAG 4760
Quality scores
AJ489607 consensus TTCTTACCAA AATGAATTAT CATCACAGAA CTTATAAAAA ACAACTGTCT AATTATAGCA ACACTCATAG 4760
Quality scores
AJ489607 consensus TTCTTACCAA AATGAATTAT CATCACAGAA CTTATAAAAA ACAACTGTCT AATTATAGCA ACACTCATAG 4760
Quality scores
AJ489607 consensus TTCTTACCAA AATGAATTAT CATCACAGAA CTTATAAAAA ACAACTGTCT AATTATAGCA ACACTCATAG 4760
Quality scores
AJ489607 consensus TTCTTACCAA AATGAATTAT CATCACAGAA CTTATAAAAA ACAACTGTCT AATTATAGCA ACACTCATAG 4760
Quality scores
AJ489607 consensus TTCTTACCAA AATGAATTAT CATCACAGAA CTTATAAAAA ACAACTGTCT AATTATAGCA ACACTCATAG 4760
Quality scores
AJ489607 consensus TTCTTACCAA AATGAATTAT CATCACAGAA CTTATAAAAA ACAACTGTCT AATTATAGCA ACACTCATAG 4760
Quality scores
Consensus TTCTTACCAA AATGAATTAT CATCACAGAA CTTATAAAAA ACAACTGTCT AATTATAGCA ACACTCATAG
Conservation
Sequence logo

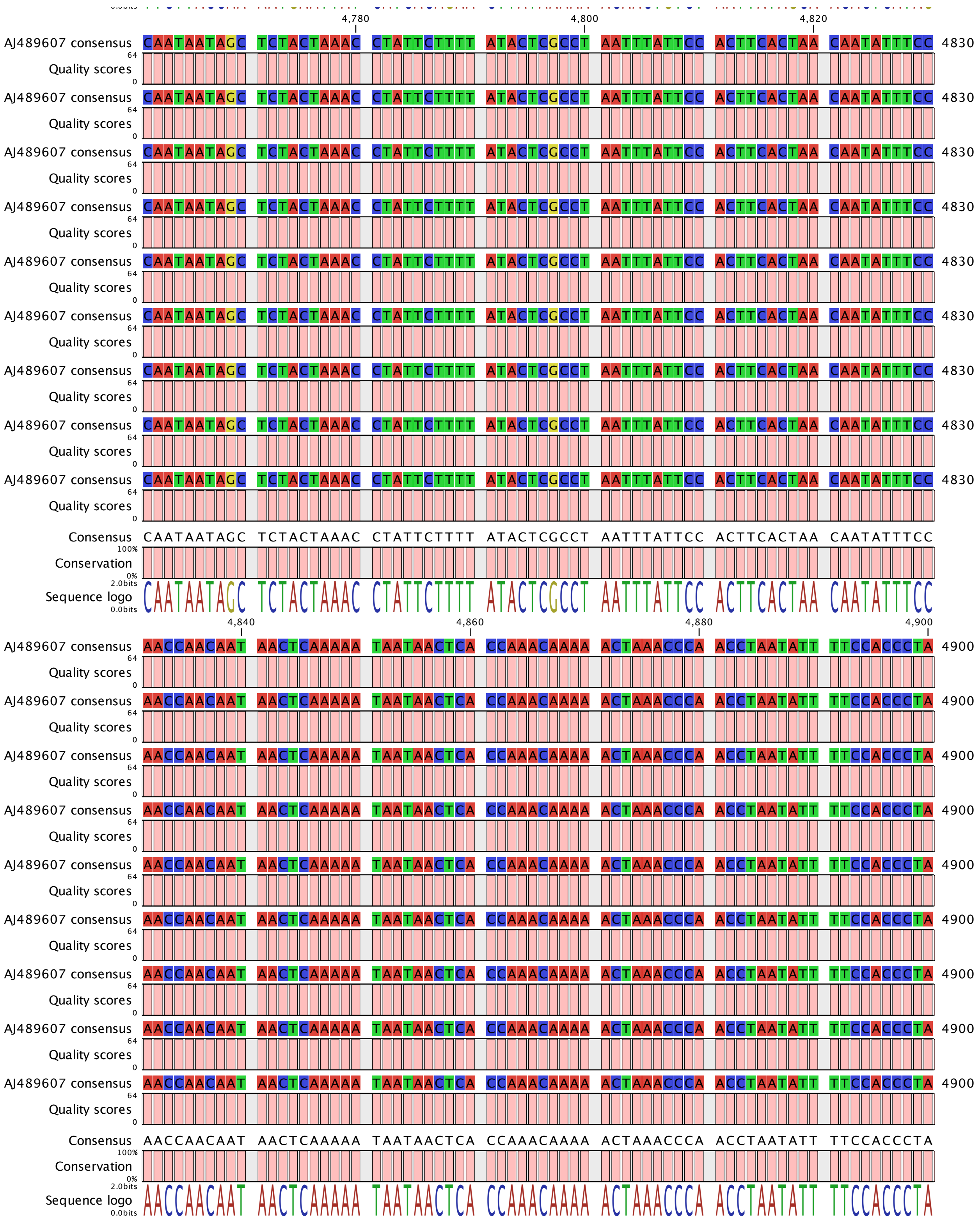

4,780
4,800
4,820
AJ489607 consensus CAATAATAGC TCTACTAAAC CTATTCTTTT ATACTCGCCT AATTTATTCC ACTTCACTAA CAATATTTCC 4830
Quality scores
AJ489607 consensus CAATAATAGC TCTACTAAAC CTATTCTTTT ATACTCGCCT AATTTATTCC ACTTCACTAA CAATATTTCC 4830
Quality scores
AJ489607 consensus CAATAATAGC TCTACTAAAC CTATTCTTTT ATACTCGCCT AATTTATTCC ACTTCACTAA CAATATTTCC 4830
Quality scores
AJ489607 consensus CAATAATAGC TCTACTAAAC CTATTCTTTT ATACTCGCCT AATTTATTCC ACTTCACTAA CAATATTTCC 4830
Quality scores
AJ489607 consensus CAATAATAGC TCTACTAAAC CTATTCTTTT ATACTCGCCT AATTTATTCC ACTTCACTAA CAATATTTCC 4830
Quality scores
AJ489607 consensus CAATAATAGC TCTACTAAAC CTATTCTTTT ATACTCGCCT AATTTATTCC ACTTCACTAA CAATATTTCC 4830
Quality scores
AJ489607 consensus CAATAATAGC TCTACTAAAC CTATTCTTTT ATACTCGCCT AATTTATTCC ACTTCACTAA CAATATTTCC 4830
Quality scores
AJ489607 consensus CAATAATAGC TCTACTAAAC CTATTCTTTT ATACTCGCCT AATTTATTCC ACTTCACTAA CAATATTTCC 4830
Quality scores
AJ489607 consensus CAATAATAGC TCTACTAAAC CTATTCTTTT ATACTCGCCT AATTTATTCC ACTTCACTAA CAATATTTCC 4830
Quality scores
Consensus CAATAATAGC TCTACTAAAC CTATTCTTTT ATACTCGCCT AATTTATTCC ACTTCACTAA CAATATTTCC
Conservation
Sequence logo CAATAATAGC TCTACTAAAC CTATTCTTTT ATACTCGCCT AATTTATTCC ACTTCACTAA CAATATTTCC
4,840
4,860
4,880
4,900
AJ489607 consensus AACCAACAAT AACTCAAAAA TAATAACTCA CCAAACAAAA ACTAAACCCA ACCTAATATT TTCCACCCTA 4900
Quality scores
AJ489607 consensus AACCAACAAT AACTCAAAAA TAATAACTCA CCAAACAAAA ACTAAACCCA ACCTAATATT TTCCACCCTA 4900
Quality scores
AJ489607 consensus AACCAACAAT AACTCAAAAA TAATAACTCA CCAAACAAAA ACTAAACCCA ACCTAATATT TTCCACCCTA 4900
Quality scores
AJ489607 consensus AACCAACAAT AACTCAAAAA TAATAACTCA CCAAACAAAA ACTAAACCCA ACCTAATATT TTCCACCCTA 4900
Quality scores
AJ489607 consensus AACCAACAAT AACTCAAAAA TAATAACTCA CCAAACAAAA ACTAAACCCA ACCTAATATT TTCCACCCTA 4900
Quality scores
AJ489607 consensus AACCAACAAT AACTCAAAAA TAATAACTCA CCAAACAAAA ACTAAACCCA ACCTAATATT TTCCACCCTA 4900
Quality scores
AJ489607 consensus AACCAACAAT AACTCAAAAA TAATAACTCA CCAAACAAAA ACTAAACCCA ACCTAATATT TTCCACCCTA 4900
Quality scores
AJ489607 consensus AACCAACAAT AACTCAAAAA TAATAACTCA CCAAACAAAA ACTAAACCCA ACCTAATATT TTCCACCCTA 4900
Quality scores
AJ489607 consensus AACCAACAAT AACTCAAAAA TAATAACTCA CCAAACAAAA ACTAAACCCA ACCTAATATT TTCCACCCTA 4900
Quality scores
Consensus AACCAACAAT AACTCAAAAA TAATAACTCA CCAAACAAAA ACTAAACCCA ACCTAATATT TTCCACCCTA
Conservation
Sequence logo AACCAACAAT AACTCAAAAA TAATAACTCA CCAAACAAAA ACTAAACCCA ACCTAATATT TTCCACCCTA

4,920 4,940 4,960

AJ489607 consensus GCTATCATAA GCACAATAAC CCTACCCCTA GCCCCCCAAC TAATTACCTA GAAGTTTAGG ATATACTAGT 4970
Quality scores (0–64)
AJ489607 consensus GCTATCATAA GCACAATAAC CCTACCCCTA GCCCCCCAAC TAATTACCTA GAAGTTTAGG ATATACTAGT 4970
Quality scores (0–64)
AJ489607 consensus GCTATCATAA GCACAATAAC CCTACCCCTA GCCCCCCAAC TAATTACCTA GAAGTTTAGG ATATACTAGT 4970
Quality scores (0–64)
AJ489607 consensus GCTATCATAA GCACAATAAC CCTACCCCTA GCCCCCCAAC TAATTACCTA GAAGTTTAGG ATATACTAGT 4970
Quality scores (0–64)
AJ489607 consensus GCTATCATAA GCACAATAAC CCTACCCCTA GCCCCCCAAC TAATTACCTA GAAGTTTAGG ATATACTAGT 4970
Quality scores (0–64)
AJ489607 consensus GCTATCATAA GCACAATAAC CCTACCCCTA GCCCCCCAAC TAATTACCTA GAAGTTTAGG ATATACTAGT 4970
Quality scores (0–64)
AJ489607 consensus GCTATCATAA GCACAATAAC CCTACCCCTA GCCCCCCAAC TAATTACCTA GAAGTTTAGG ATATACTAGT 4970
Quality scores (0–64)
AJ489607 consensus GCTATCATAA GCACAATAAC CCTACCCCTA GCCCCCCAAC TAATTACCTA GAAGTTTAGG ATATACTAGT 4970
Quality scores (0–64)
AJ489607 consensus GCTATCATAA GCACAATAAC CCTACCCCTA GCCCCCCAAC TAATTACCTA GAAGTTTAGG ATATACTAGT 4970
Quality scores (0–64)
Consensus GCTATCATAA GCACAATAAC CCTACCCCTA GCCCCCCAAC TAATTACCTA GAAGTTTAGG ATATACTAGT
Conservation (0%–100%)
Sequence logo (0.0bits–2.0bits) GCTATCATAA GCACAATAAC CCTACCCCTA GCCCCCCAAC TAATTACCTA GAAGTTTAGG ATATACTAGT

4,980 5,000 5,020 5,040

AJ489607 consensus CCGCGAGCCT TCAAAGCCCT AAGAAAACAC ACAAGTTTAA CTTCTGATAA GGACTGTAAG ACTTCATCCT 5040
Quality scores (0–64)
AJ489607 consensus CCGCGAGCCT TCAAAGCCCT AAGAAAACAC ACAAGTTTAA CTTCTGATAA GGACTGTAAG ACTTCATCCT 5040
Quality scores (0–64)
AJ489607 consensus CCGCGAGCCT TCAAAGCCCT AAGAAAACAC ACAAGTTTAA CTTCTGATAA GGACTGTAAG ACTTCATCCT 5040
Quality scores (0–64)
AJ489607 consensus CCGCGAGCCT TCAAAGCCCT AAGAAAACAC ACAAGTTTAA CTTCTGATAA GGACTGTAAG ACTTCATCCT 5040
Quality scores (0–64)
AJ489607 consensus CCGCGAGCCT TCAAAGCCCT AAGAAAACAC ACAAGTTTAA CTTCTGATAA GGACTGTAAG ACTTCATCCT 5040
Quality scores (0–64)
AJ489607 consensus CCGCGAGCCT TCAAAGCCCT AAGAAAACAC ACAAGTTTAA CTTCTGATAA GGACTGTAAG ACTTCATCCT 5040
Quality scores (0–64)
AJ489607 consensus CCGCGAGCCT TCAAAGCCCT AAGAAAACAC ACAAGTTTAA CTTCTGATAA GGACTGTAAG ACTTCATCCT 5040
Quality scores (0–64)
AJ489607 consensus CCGCGAGCCT TCAAAGCCCT AAGAAAACAC ACAAGTTTAA CTTCTGATAA GGACTGTAAG ACTTCATCCT 5040
Quality scores (0–64)
AJ489607 consensus CCGCGAGCCT TCAAAGCCCT AAGAAAACAC ACAAGTTTAA CTTCTGATAA GGACTGTAAG ACTTCATCCT 5040
Quality scores (0–64)
Consensus CCGCGAGCCT TCAAAGCCCT AAGAAAACAC ACAAGTTTAA CTTCTGATAA GGACTGTAAG ACTTCATCCT
Conservation (0%–100%)
Sequence logo (0.0bits–2.0bits) CCGCGAGCCT TCAAAGCCCT AAGAAAACAC ACAAGTTTAA CTTCTGATAA GGACTGTAAG ACTTCATCCT

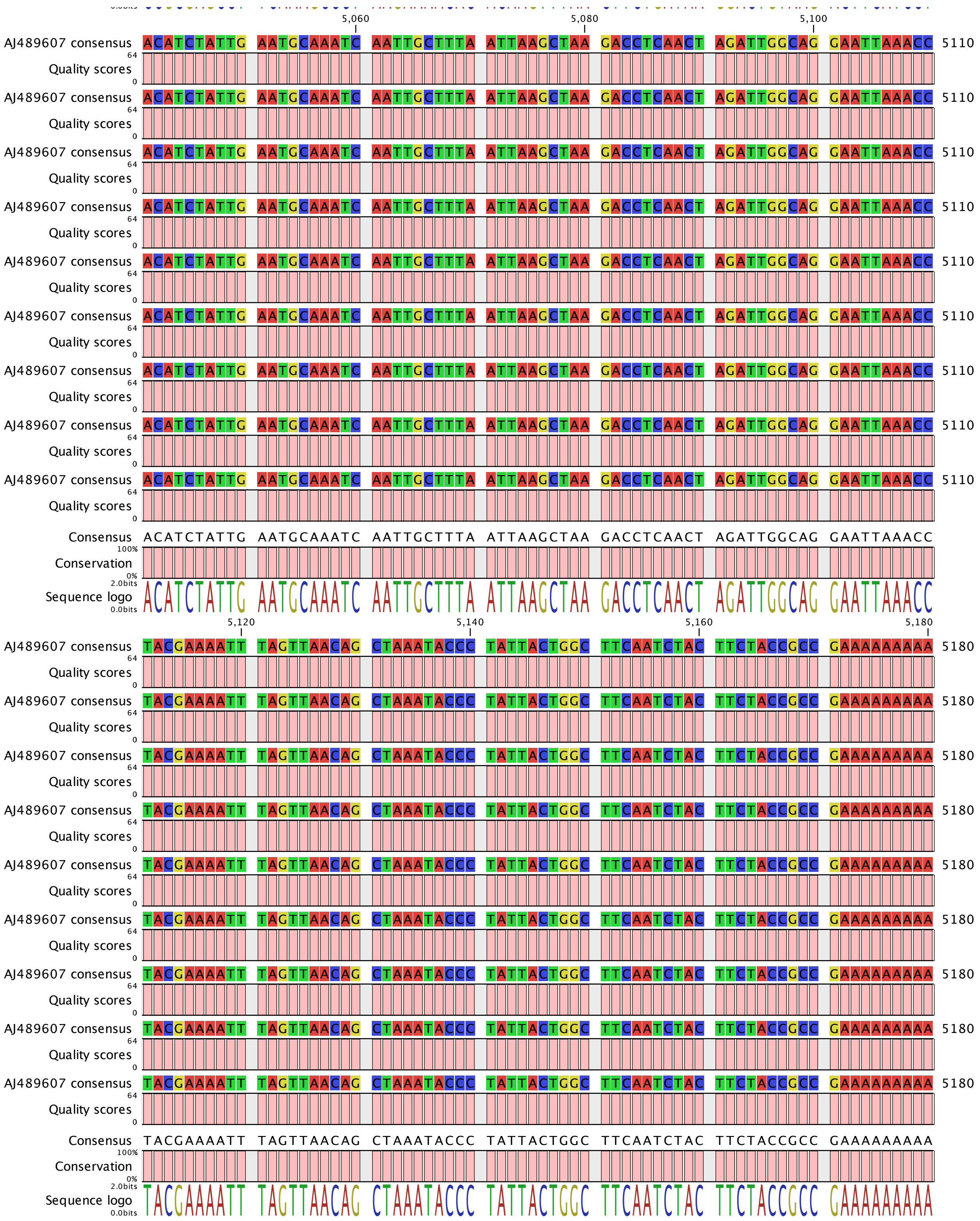
5,060
5,080
5,100
AJ489607 consensus ACATCTATTG AATGCAAATC AATTGCTTTA ATTAAGCTAA GACCTCAACT AGATTGGCAG GAATTAAACC 5110
64
Quality scores
0
AJ489607 consensus ACATCTATTG AATGCAAATC AATTGCTTTA ATTAAGCTAA GACCTCAACT AGATTGGCAG GAATTAAACC 5110
64
Quality scores
0
AJ489607 consensus ACATCTATTG AATGCAAATC AATTGCTTTA ATTAAGCTAA GACCTCAACT AGATTGGCAG GAATTAAACC 5110
64
Quality scores
0
AJ489607 consensus ACATCTATTG AATGCAAATC AATTGCTTTA ATTAAGCTAA GACCTCAACT AGATTGGCAG GAATTAAACC 5110
64
Quality scores
0
AJ489607 consensus ACATCTATTG AATGCAAATC AATTGCTTTA ATTAAGCTAA GACCTCAACT AGATTGGCAG GAATTAAACC 5110
64
Quality scores
0
AJ489607 consensus ACATCTATTG AATGCAAATC AATTGCTTTA ATTAAGCTAA GACCTCAACT AGATTGGCAG GAATTAAACC 5110
64
Quality scores
0
AJ489607 consensus ACATCTATTG AATGCAAATC AATTGCTTTA ATTAAGCTAA GACCTCAACT AGATTGGCAG GAATTAAACC 5110
64
Quality scores
0
AJ489607 consensus ACATCTATTG AATGCAAATC AATTGCTTTA ATTAAGCTAA GACCTCAACT AGATTGGCAG GAATTAAACC 5110
64
Quality scores
0
AJ489607 consensus ACATCTATTG AATGCAAATC AATTGCTTTA ATTAAGCTAA GACCTCAACT AGATTGGCAG GAATTAAACC 5110
64
Quality scores
0
Consensus ACATCTATTG AATGCAAATC AATTGCTTTA ATTAAGCTAA GACCTCAACT AGATTGGCAG GAATTAAACC
100%
Conservation
0%
2.0bits
Sequence logo
0.0bits
5,120
5,140
5,160
5,180
AJ489607 consensus TACGAAAATT TAGTTAACAG CTAAATACCC TATTACTGGC TTCAATCTAC TTCTACCGCC GAAAAAAAAA 5180
64
Quality scores
0
AJ489607 consensus TACGAAAATT TAGTTAACAG CTAAATACCC TATTACTGGC TTCAATCTAC TTCTACCGCC GAAAAAAAAA 5180
64
Quality scores
0
AJ489607 consensus TACGAAAATT TAGTTAACAG CTAAATACCC TATTACTGGC TTCAATCTAC TTCTACCGCC GAAAAAAAAA 5180
64
Quality scores
0
AJ489607 consensus TACGAAAATT TAGTTAACAG CTAAATACCC TATTACTGGC TTCAATCTAC TTCTACCGCC GAAAAAAAAA 5180
64
Quality scores
0
AJ489607 consensus TACGAAAATT TAGTTAACAG CTAAATACCC TATTACTGGC TTCAATCTAC TTCTACCGCC GAAAAAAAAA 5180
64
Quality scores
0
AJ489607 consensus TACGAAAATT TAGTTAACAG CTAAATACCC TATTACTGGC TTCAATCTAC TTCTACCGCC GAAAAAAAAA 5180
64
Quality scores
0
AJ489607 consensus TACGAAAATT TAGTTAACAG CTAAATACCC TATTACTGGC TTCAATCTAC TTCTACCGCC GAAAAAAAAA 5180
64
Quality scores
0
AJ489607 consensus TACGAAAATT TAGTTAACAG CTAAATACCC TATTACTGGC TTCAATCTAC TTCTACCGCC GAAAAAAAAA 5180
64
Quality scores
0
AJ489607 consensus TACGAAAATT TAGTTAACAG CTAAATACCC TATTACTGGC TTCAATCTAC TTCTACCGCC GAAAAAAAAA 5180
64
Quality scores
0
Consensus TACGAAAATT TAGTTAACAG CTAAATACCC TATTACTGGC TTCAATCTAC TTCTACCGCC GAAAAAAAAA
100%
Conservation
0%
2.0bits
Sequence logo
0.0bits

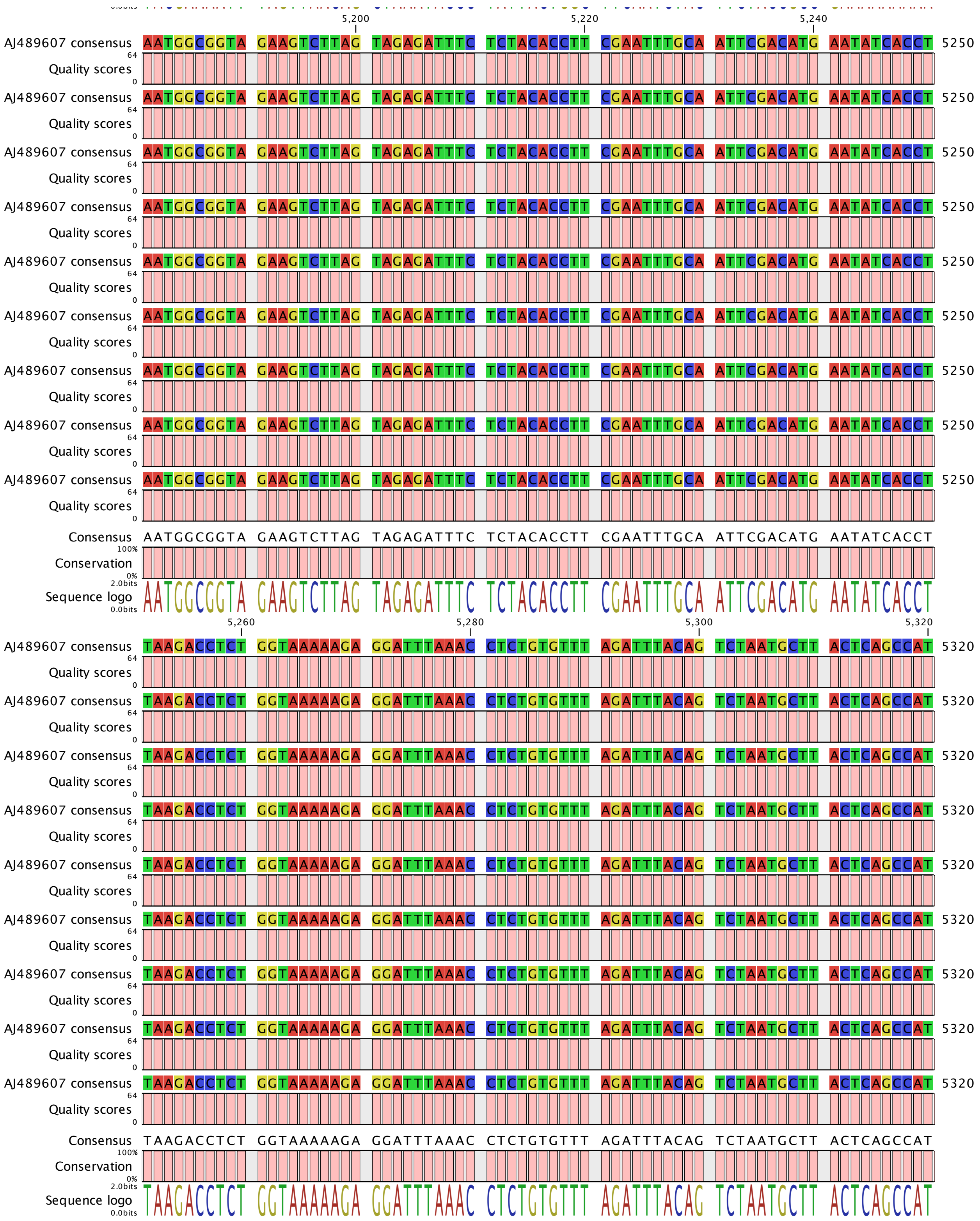
5,200
5,220
5,240
AJ489607 consensus AATGGCGGTA GAAGTCTTAG TAGAGATTTC TCTACACCTT CGAATTTGCA ATTCGACATG AATATCACCT 5250
64
Quality scores
0
AJ489607 consensus AATGGCGGTA GAAGTCTTAG TAGAGATTTC TCTACACCTT CGAATTTGCA ATTCGACATG AATATCACCT 5250
64
Quality scores
0
AJ489607 consensus AATGGCGGTA GAAGTCTTAG TAGAGATTTC TCTACACCTT CGAATTTGCA ATTCGACATG AATATCACCT 5250
64
Quality scores
0
AJ489607 consensus AATGGCGGTA GAAGTCTTAG TAGAGATTTC TCTACACCTT CGAATTTGCA ATTCGACATG AATATCACCT 5250
64
Quality scores
0
AJ489607 consensus AATGGCGGTA GAAGTCTTAG TAGAGATTTC TCTACACCTT CGAATTTGCA ATTCGACATG AATATCACCT 5250
64
Quality scores
0
AJ489607 consensus AATGGCGGTA GAAGTCTTAG TAGAGATTTC TCTACACCTT CGAATTTGCA ATTCGACATG AATATCACCT 5250
64
Quality scores
0
AJ489607 consensus AATGGCGGTA GAAGTCTTAG TAGAGATTTC TCTACACCTT CGAATTTGCA ATTCGACATG AATATCACCT 5250
64
Quality scores
0
AJ489607 consensus AATGGCGGTA GAAGTCTTAG TAGAGATTTC TCTACACCTT CGAATTTGCA ATTCGACATG AATATCACCT 5250
64
Quality scores
0
AJ489607 consensus AATGGCGGTA GAAGTCTTAG TAGAGATTTC TCTACACCTT CGAATTTGCA ATTCGACATG AATATCACCT 5250
64
Quality scores
0
Consensus AATGGCGGTA GAAGTCTTAG TAGAGATTTC TCTACACCTT CGAATTTGCA ATTCGACATG AATATCACCT
100%
Conservation
0%
2.0bits
Sequence logo
0.0bits
5,260
5,280
5,300
5,320
AJ489607 consensus TAAGACCTCT GGTAAAAAGA GGATTTAAAC CTCTGTGTTT AGATTTACAG TCTAATGCTT ACTCAGCCAT 5320
64
Quality scores
0
AJ489607 consensus TAAGACCTCT GGTAAAAAGA GGATTTAAAC CTCTGTGTTT AGATTTACAG TCTAATGCTT ACTCAGCCAT 5320
64
Quality scores
0
AJ489607 consensus TAAGACCTCT GGTAAAAAGA GGATTTAAAC CTCTGTGTTT AGATTTACAG TCTAATGCTT ACTCAGCCAT 5320
64
Quality scores
0
AJ489607 consensus TAAGACCTCT GGTAAAAAGA GGATTTAAAC CTCTGTGTTT AGATTTACAG TCTAATGCTT ACTCAGCCAT 5320
64
Quality scores
0
AJ489607 consensus TAAGACCTCT GGTAAAAAGA GGATTTAAAC CTCTGTGTTT AGATTTACAG TCTAATGCTT ACTCAGCCAT 5320
64
Quality scores
0
AJ489607 consensus TAAGACCTCT GGTAAAAAGA GGATTTAAAC CTCTGTGTTT AGATTTACAG TCTAATGCTT ACTCAGCCAT 5320
64
Quality scores
0
AJ489607 consensus TAAGACCTCT GGTAAAAAGA GGATTTAAAC CTCTGTGTTT AGATTTACAG TCTAATGCTT ACTCAGCCAT 5320
64
Quality scores
0
AJ489607 consensus TAAGACCTCT GGTAAAAAGA GGATTTAAAC CTCTGTGTTT AGATTTACAG TCTAATGCTT ACTCAGCCAT 5320
64
Quality scores
0
AJ489607 consensus TAAGACCTCT GGTAAAAAGA GGATTTAAAC CTCTGTGTTT AGATTTACAG TCTAATGCTT ACTCAGCCAT 5320
64
Quality scores
0
Consensus TAAGACCTCT GGTAAAAAGA GGATTTAAAC CTCTGTGTTT AGATTTACAG TCTAATGCTT ACTCAGCCAT
100%
Conservation
0%
2.0bits
Sequence logo
0.0bits

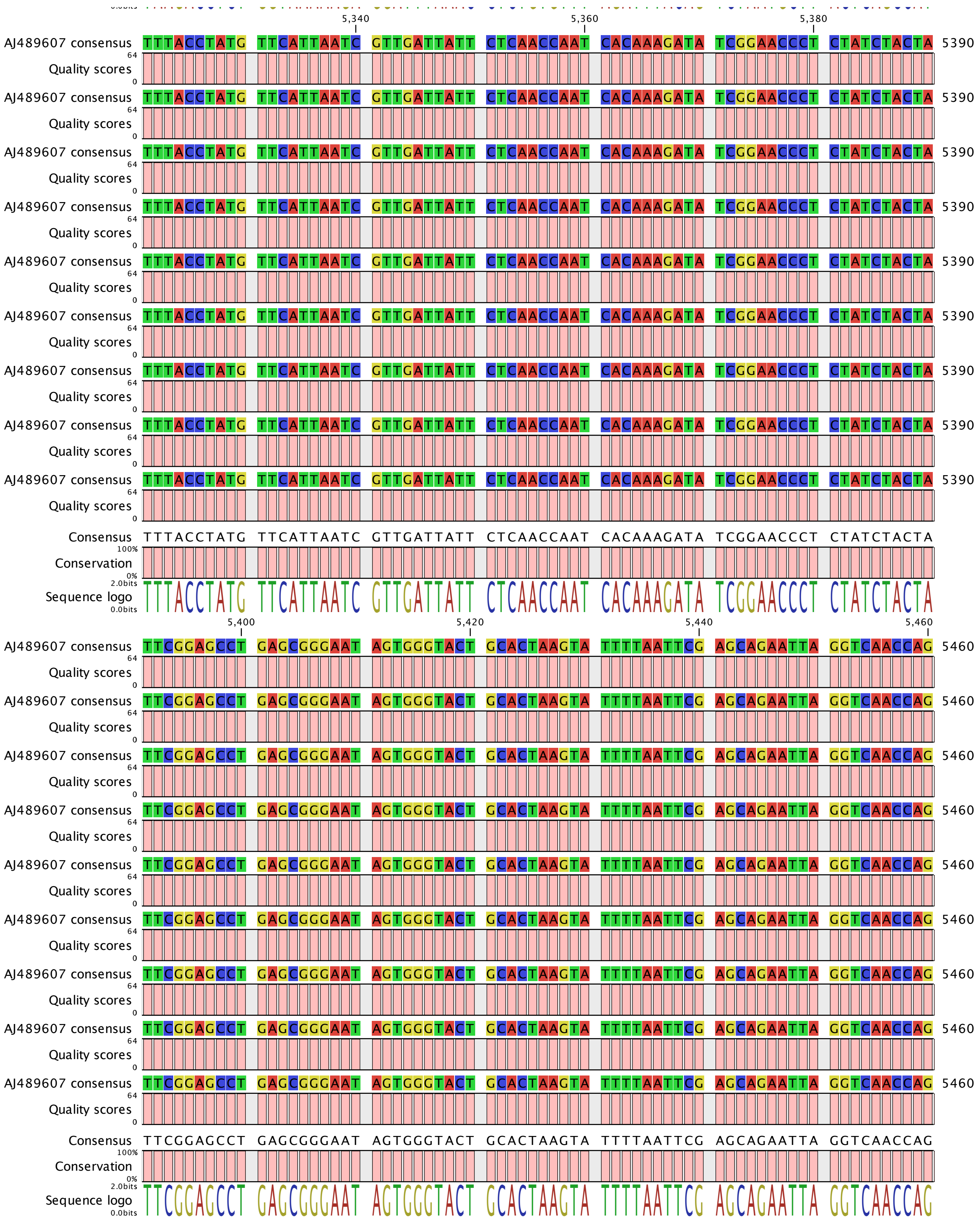
5,340
5,360
5,380
AJ489607 consensus TTTACCTATG TTCATTAATC GTTGATTATT CTCAACCAAT CACAAAGATA TCGGAACCCT CTATCTACTA 5390
Quality scores
AJ489607 consensus TTTACCTATG TTCATTAATC GTTGATTATT CTCAACCAAT CACAAAGATA TCGGAACCCT CTATCTACTA 5390
Quality scores
AJ489607 consensus TTTACCTATG TTCATTAATC GTTGATTATT CTCAACCAAT CACAAAGATA TCGGAACCCT CTATCTACTA 5390
Quality scores
AJ489607 consensus TTTACCTATG TTCATTAATC GTTGATTATT CTCAACCAAT CACAAAGATA TCGGAACCCT CTATCTACTA 5390
Quality scores
AJ489607 consensus TTTACCTATG TTCATTAATC GTTGATTATT CTCAACCAAT CACAAAGATA TCGGAACCCT CTATCTACTA 5390
Quality scores
AJ489607 consensus TTTACCTATG TTCATTAATC GTTGATTATT CTCAACCAAT CACAAAGATA TCGGAACCCT CTATCTACTA 5390
Quality scores
AJ489607 consensus TTTACCTATG TTCATTAATC GTTGATTATT CTCAACCAAT CACAAAGATA TCGGAACCCT CTATCTACTA 5390
Quality scores
AJ489607 consensus TTTACCTATG TTCATTAATC GTTGATTATT CTCAACCAAT CACAAAGATA TCGGAACCCT CTATCTACTA 5390
Quality scores
AJ489607 consensus TTTACCTATG TTCATTAATC GTTGATTATT CTCAACCAAT CACAAAGATA TCGGAACCCT CTATCTACTA 5390
Quality scores
Consensus TTTACCTATG TTCATTAATC GTTGATTATT CTCAACCAAT CACAAAGATA TCGGAACCCT CTATCTACTA
Conservation
Sequence logo
5,400
5,420
5,440
5,460
AJ489607 consensus TTCGGAGCCT GAGCGGGAAT AGTGGGTACT GCACTAAGTA TTTTAATTCG AGCAGAATTA GGTCAACCAG 5460
Quality scores
AJ489607 consensus TTCGGAGCCT GAGCGGGAAT AGTGGGTACT GCACTAAGTA TTTTAATTCG AGCAGAATTA GGTCAACCAG 5460
Quality scores
AJ489607 consensus TTCGGAGCCT GAGCGGGAAT AGTGGGTACT GCACTAAGTA TTTTAATTCG AGCAGAATTA GGTCAACCAG 5460
Quality scores
AJ489607 consensus TTCGGAGCCT GAGCGGGAAT AGTGGGTACT GCACTAAGTA TTTTAATTCG AGCAGAATTA GGTCAACCAG 5460
Quality scores
AJ489607 consensus TTCGGAGCCT GAGCGGGAAT AGTGGGTACT GCACTAAGTA TTTTAATTCG AGCAGAATTA GGTCAACCAG 5460
Quality scores
AJ489607 consensus TTCGGAGCCT GAGCGGGAAT AGTGGGTACT GCACTAAGTA TTTTAATTCG AGCAGAATTA GGTCAACCAG 5460
Quality scores
AJ489607 consensus TTCGGAGCCT GAGCGGGAAT AGTGGGTACT GCACTAAGTA TTTTAATTCG AGCAGAATTA GGTCAACCAG 5460
Quality scores
AJ489607 consensus TTCGGAGCCT GAGCGGGAAT AGTGGGTACT GCACTAAGTA TTTTAATTCG AGCAGAATTA GGTCAACCAG 5460
Quality scores
AJ489607 consensus TTCGGAGCCT GAGCGGGAAT AGTGGGTACT GCACTAAGTA TTTTAATTCG AGCAGAATTA GGTCAACCAG 5460
Quality scores
Consensus TTCGGAGCCT GAGCGGGAAT AGTGGGTACT GCACTAAGTA TTTTAATTCG AGCAGAATTA GGTCAACCAG
Conservation
Sequence logo

5,480 5,500 5,520

AJ489607 consensus GTGCACTTTT AGGAGATGAC CAAATTTACA ATGTTATCGT AACTGCCCAT GCTTTTGTTA TAATTTTCTT 5530
64
Quality scores
0
AJ489607 consensus GTGCACTTTT AGGAGATGAC CAAATTTACA ATGTTATCGT AACTGCCCAT GCTTTTGTTA TAATTTTCTT 5530
64
Quality scores
0
AJ489607 consensus GTGCACTTTT AGGAGATGAC CAAATTTACA ATGTTATCGT AACTGCCCAT GCTTTTGTTA TAATTTTCTT 5530
64
Quality scores
0
AJ489607 consensus GTGCACTTTT AGGAGATGAC CAAATTTACA ATGTTATCGT AACTGCCCAT GCTTTTGTTA TAATTTTCTT 5530
64
Quality scores
0
AJ489607 consensus GTGCACTTTT AGGAGATGAC CAAATTTACA ATGTTATCGT AACTGCCCAT GCTTTTGTTA TAATTTTCTT 5530
64
Quality scores
0
AJ489607 consensus GTGCACTTTT AGGAGATGAC CAAATTTACA ATGTTATCGT AACTGCCCAT GCTTTTGTTA TAATTTTCTT 5530
64
Quality scores
0
AJ489607 consensus GTGCACTTTT AGGAGATGAC CAAATTTACA ATGTTATCGT AACTGCCCAT GCTTTTGTTA TAATTTTCTT 5530
64
Quality scores
0
AJ489607 consensus GTGCACTTTT AGGAGATGAC CAAATTTACA ATGTTATCGT AACTGCCCAT GCTTTTGTTA TAATTTTCTT 5530
64
Quality scores
0
AJ489607 consensus GTGCACTTTT AGGAGATGAC CAAATTTACA ATGTTATCGT AACTGCCCAT GCTTTTGTTA TAATTTTCTT 5530
64
Quality scores
0
Consensus GTGCACTTTT AGGAGATGAC CAAATTTACA ATGTTATCGT AACTGCCCAT GCTTTTGTTA TAATTTTCTT
100%
Conservation
0%
2.0bits
Sequence logo GTGCACTTTT AGGAGATGAC CAAATTTACA ATGTTATCGT AACTGCCCAT GCTTTTGTTA TAATTTTCTT
0.0bits

5,540 5,560 5,580 5,600

AJ489607 consensus CATAGTAATA CCAATAATAA TTGGAGGCTT TGGAAACTGA CTTGTCCCAC TAATAATCGG AGCCCCAGAT 5600
64
Quality scores
0
AJ489607 consensus CATAGTAATA CCAATAATAA TTGGAGGCTT TGGAAACTGA CTTGTCCCAC TAATAATCGG AGCCCCAGAT 5600
64
Quality scores
0
AJ489607 consensus CATAGTAATA CCAATAATAA TTGGAGGCTT TGGAAACTGA CTTGTCCCAC TAATAATCGG AGCCCCAGAT 5600
64
Quality scores
0
AJ489607 consensus CATAGTAATA CCAATAATAA TTGGAGGCTT TGGAAACTGA CTTGTCCCAC TAATAATCGG AGCCCCAGAT 5600
64
Quality scores
0
AJ489607 consensus CATAGTAATA CCAATAATAA TTGGAGGCTT TGGAAACTGA CTTGTCCCAC TAATAATCGG AGCCCCAGAT 5600
64
Quality scores
0
AJ489607 consensus CATAGTAATA CCAATAATAA TTGGAGGCTT TGGAAACTGA CTTGTCCCAC TAATAATCGG AGCCCCAGAT 5600
64
Quality scores
0
AJ489607 consensus CATAGTAATA CCAATAATAA TTGGAGGCTT TGGAAACTGA CTTGTCCCAC TAATAATCGG AGCCCCAGAT 5600
64
Quality scores
0
AJ489607 consensus CATAGTAATA CCAATAATAA TTGGAGGCTT TGGAAACTGA CTTGTCCCAC TAATAATCGG AGCCCCAGAT 5600
64
Quality scores
0
AJ489607 consensus CATAGTAATA CCAATAATAA TTGGAGGCTT TGGAAACTGA CTTGTCCCAC TAATAATCGG AGCCCCAGAT 5600
64
Quality scores
0
Consensus CATAGTAATA CCAATAATAA TTGGAGGCTT TGGAAACTGA CTTGTCCCAC TAATAATCGG AGCCCCAGAT
100%
Conservation
0%
2.0bits
Sequence logo CATAGTAATA CCAATAATAA TTGGAGGCTT TGGAAACTGA CTTGTCCCAC TAATAATCGG AGCCCCAGAT
0.0bits

5,620 5,640 5,660

AJ489607 consensus ATAGCATTCC CACGAATAAA TAATATAAGT TTTTGACTCC TACCACCATC ATTTCTCCTT CTCCTAGCAT 5670
Quality scores 64 0
AJ489607 consensus ATAGCATTCC CACGAATAAA TAATATAAGT TTTTGACTCC TACCACCATC ATTTCTCCTT CTCCTAGCAT 5670
Quality scores 64 0
AJ489607 consensus ATAGCATTCC CACGAATAAA TAATATAAGT TTTTGACTCC TACCACCATC ATTTCTCCTT CTCCTAGCAT 5670
Quality scores 64 0
AJ489607 consensus ATAGCATTCC CACGAATAAA TAATATAAGT TTTTGACTCC TACCACCATC ATTTCTCCTT CTCCTAGCAT 5670
Quality scores 64 0
AJ489607 consensus ATAGCATTCC CACGAATAAA TAATATAAGT TTTTGACTCC TACCACCATC ATTTCTCCTT CTCCTAGCAT 5670
Quality scores 64 0
AJ489607 consensus ATAGCATTCC CACGAATAAA TAATATAAGT TTTTGACTCC TACCACCATC ATTTCTCCTT CTCCTAGCAT 5670
Quality scores 64 0
AJ489607 consensus ATAGCATTCC CACGAATAAA TAATATAAGT TTTTGACTCC TACCACCATC ATTTCTCCTT CTCCTAGCAT 5670
Quality scores 64 0
AJ489607 consensus ATAGCATTCC CACGAATAAA TAATATAAGT TTTTGACTCC TACCACCATC ATTTCTCCTT CTCCTAGCAT 5670
Quality scores 64 0
AJ489607 consensus ATAGCATTCC CACGAATAAA TAATATAAGT TTTTGACTCC TACCACCATC ATTTCTCCTT CTCCTAGCAT 5670
Quality scores 64 0
Consensus ATAGCATTCC CACGAATAAA TAATATAAGT TTTTGACTCC TACCACCATC ATTTCTCCTT CTCCTAGCAT
Conservation 100% 0%
Sequence logo 2.0bits 0.0bits ATAGCATTCC CACGAATAAA TAATATAAGT TTTTGACTCC TACCACCATC ATTTCTCCTT CTCCTAGCAT

5,680 5,700 5,720 5,740

AJ489607 consensus CATCAATAGT AGAAGCAGGA GCAGGAACAG GATGAACAGT CTACCCACCT CTAGCCGGAA ATCTAGCCCA 5740
Quality scores 64 0
AJ489607 consensus CATCAATAGT AGAAGCAGGA GCAGGAACAG GATGAACAGT CTACCCACCT CTAGCCGGAA ATCTAGCCCA 5740
Quality scores 64 0
AJ489607 consensus CATCAATAGT AGAAGCAGGA GCAGGAACAG GATGAACAGT CTACCCACCT CTAGCCGGAA ATCTAGCCCA 5740
Quality scores 64 0
AJ489607 consensus CATCAATAGT AGAAGCAGGA GCAGGAACAG GATGAACAGT CTACCCACCT CTAGCCGGAA ATCTAGCCCA 5740
Quality scores 64 0
AJ489607 consensus CATCAATAGT AGAAGCAGGA GCAGGAACAG GATGAACAGT CTACCCACCT CTAGCCGGAA ATCTAGCCCA 5740
Quality scores 64 0
AJ489607 consensus CATCAATAGT AGAAGCAGGA GCAGGAACAG GATGAACAGT CTACCCACCT CTAGCCGGAA ATCTAGCCCA 5740
Quality scores 64 0
AJ489607 consensus CATCAATAGT AGAAGCAGGA GCAGGAACAG GATGAACAGT CTACCCACCT CTAGCCGGAA ATCTAGCCCA 5740
Quality scores 64 0
AJ489607 consensus CATCAATAGT AGAAGCAGGA GCAGGAACAG GATGAACAGT CTACCCACCT CTAGCCGGAA ATCTAGCCCA 5740
Quality scores 64 0
AJ489607 consensus CATCAATAGT AGAAGCAGGA GCAGGAACAG GATGAACAGT CTACCCACCT CTAGCCGGAA ATCTAGCCCA 5740
Quality scores 64 0
Consensus CATCAATAGT AGAAGCAGGA GCAGGAACAG GATGAACAGT CTACCCACCT CTAGCCGGAA ATCTAGCCCA
Conservation 100% 0%
Sequence logo 2.0bits 0.0bits CATCAATAGT AGAAGCAGGA GCAGGAACAG GATGAACAGT CTACCCACCT CTAGCCGGAA ATCTAGCCCA

5,760 5,780 5,800

AJ489607 consensus TGCAGGAGCA TCAGTAGACC TAACAATTTT CTCCCTTCAT TTAGCTGGAG TGTCATCTAT TTTAGGTGCA 5810
Quality scores 64 0
AJ489607 consensus TGCAGGAGCA TCAGTAGACC TAACAATTTT CTCCCTTCAT TTAGCTGGAG TGTCATCTAT TTTAGGTGCA 5810
Quality scores 64 0
AJ489607 consensus TGCAGGAGCA TCAGTAGACC TAACAATTTT CTCCCTTCAT TTAGCTGGAG TGTCATCTAT TTTAGGTGCA 5810
Quality scores 64 0
AJ489607 consensus TGCAGGAGCA TCAGTAGACC TAACAATTTT CTCCCTTCAT TTAGCTGGAG TGTCATCTAT TTTAGGTGCA 5810
Quality scores 64 0
AJ489607 consensus TGCAGGAGCA TCAGTAGACC TAACAATTTT CTCCCTTCAT TTAGCTGGAG TGTCATCTAT TTTAGGTGCA 5810
Quality scores 64 0
AJ489607 consensus TGCAGGAGCA TCAGTAGACC TAACAATTTT CTCCCTTCAT TTAGCTGGAG TGTCATCTAT TTTAGGTGCA 5810
Quality scores 64 0
AJ489607 consensus TGCAGGAGCA TCAGTAGACC TAACAATTTT CTCCCTTCAT TTAGCTGGAG TGTCATCTAT TTTAGGTGCA 5810
Quality scores 64 0
AJ489607 consensus TGCAGGAGCA TCAGTAGACC TAACAATTTT CTCCCTTCAT TTAGCTGGAG TGTCATCTAT TTTAGGTGCA 5810
Quality scores 64 0
AJ489607 consensus TGCAGGAGCA TCAGTAGACC TAACAATTTT CTCCCTTCAT TTAGCTGGAG TGTCATCTAT TTTAGGTGCA 5810
Quality scores 64 0
Consensus TGCAGGAGCA TCAGTAGACC TAACAATTTT CTCCCTTCAT TTAGCTGGAG TGTCATCTAT TTTAGGTGCA
Conservation 100% 0%
Sequence logo 2.0bits 0.0bits TGCAGGAGCA TCAGTAGACC TAACAATTTT CTCCCTTCAT TTAGCTGGAG TGTCATCTAT TTTAGGTGCA

5,820 5,840 5,860 5,880

AJ489607 consensus ATTAATTTTA TTACCACTAT TATCAACATG AAACCCCCAG CCATAACACA GTATCAAACT CCACTATTTG 5880
Quality scores 64 0
AJ489607 consensus ATTAATTTTA TTACCACTAT TATCAACATG AAACCCCCAG CCATAACACA GTATCAAACT CCACTATTTG 5880
Quality scores 64 0
AJ489607 consensus ATTAATTTTA TTACCACTAT TATCAACATG AAACCCCCAG CCATAACACA GTATCAAACT CCACTATTTG 5880
Quality scores 64 0
AJ489607 consensus ATTAATTTTA TTACCACTAT TATCAACATG AAACCCCCAG CCATAACACA GTATCAAACT CCACTATTTG 5880
Quality scores 64 0
AJ489607 consensus ATTAATTTTA TTACCACTAT TATCAACATG AAACCCCCAG CCATAACACA GTATCAAACT CCACTATTTG 5880
Quality scores 64 0
AJ489607 consensus ATTAATTTTA TTACCACTAT TATCAACATG AAACCCCCAG CCATAACACA GTATCAAACT CCACTATTTG 5880
Quality scores 64 0
AJ489607 consensus ATTAATTTTA TTACCACTAT TATCAACATG AAACCCCCAG CCATAACACA GTATCAAACT CCACTATTTG 5880
Quality scores 64 0
AJ489607 consensus ATTAATTTTA TTACCACTAT TATCAACATG AAACCCCCAG CCATAACACA GTATCAAACT CCACTATTTG 5880
Quality scores 64 0
AJ489607 consensus ATTAATTTTA TTACCACTAT TATCAACATG AAACCCCCAG CCATAACACA GTATCAAACT CCACTATTTG 5880
Quality scores 64 0
Consensus ATTAATTTTA TTACCACTAT TATCAACATG AAACCCCCAG CCATAACACA GTATCAAACT CCACTATTTG
Conservation 100% 0%
Sequence logo 2.0bits 0.0bits ATTAATTTTA TTACCACTAT TATCAACATG AAACCCCCAG CCATAACACA GTATCAAACT CCACTATTTG

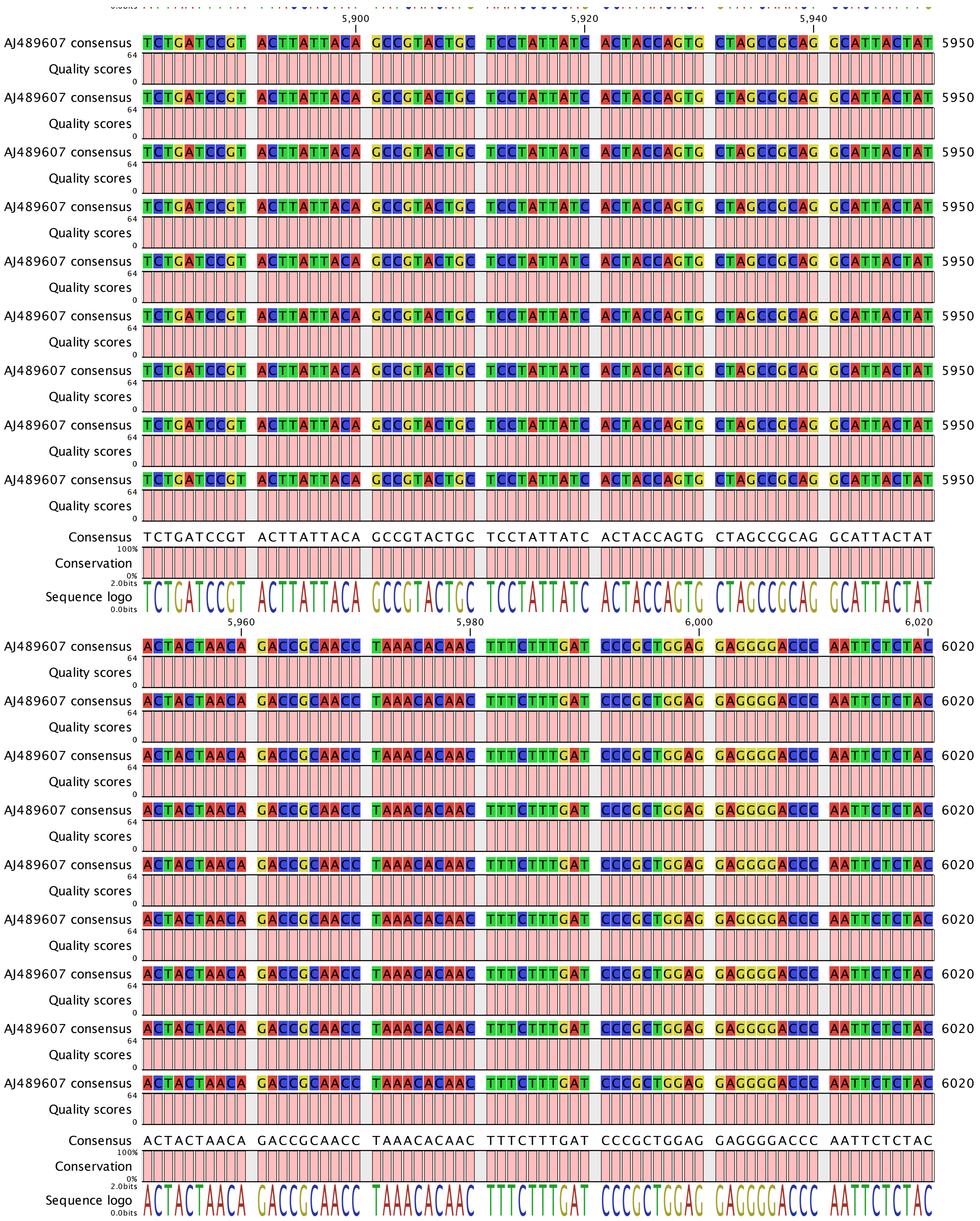
5,900 5,920 5,940
AJ489607 consensus TCTGATCCGT ACTTATTACA GCCGTACTGC TCCTATTATC ACTACCAGTG CTAGCCGCAG GCATTACTAT 5950
Quality scores 64 0
AJ489607 consensus TCTGATCCGT ACTTATTACA GCCGTACTGC TCCTATTATC ACTACCAGTG CTAGCCGCAG GCATTACTAT 5950
Quality scores 64 0
AJ489607 consensus TCTGATCCGT ACTTATTACA GCCGTACTGC TCCTATTATC ACTACCAGTG CTAGCCGCAG GCATTACTAT 5950
Quality scores 64 0
AJ489607 consensus TCTGATCCGT ACTTATTACA GCCGTACTGC TCCTATTATC ACTACCAGTG CTAGCCGCAG GCATTACTAT 5950
Quality scores 64 0
AJ489607 consensus TCTGATCCGT ACTTATTACA GCCGTACTGC TCCTATTATC ACTACCAGTG CTAGCCGCAG GCATTACTAT 5950
Quality scores 64 0
AJ489607 consensus TCTGATCCGT ACTTATTACA GCCGTACTGC TCCTATTATC ACTACCAGTG CTAGCCGCAG GCATTACTAT 5950
Quality scores 64 0
AJ489607 consensus TCTGATCCGT ACTTATTACA GCCGTACTGC TCCTATTATC ACTACCAGTG CTAGCCGCAG GCATTACTAT 5950
Quality scores 64 0
AJ489607 consensus TCTGATCCGT ACTTATTACA GCCGTACTGC TCCTATTATC ACTACCAGTG CTAGCCGCAG GCATTACTAT 5950
Quality scores 64 0
AJ489607 consensus TCTGATCCGT ACTTATTACA GCCGTACTGC TCCTATTATC ACTACCAGTG CTAGCCGCAG GCATTACTAT 5950
Quality scores 64 0
Consensus TCTGATCCGT ACTTATTACA GCCGTACTGC TCCTATTATC ACTACCAGTG CTAGCCGCAG GCATTACTAT
Conservation 100% 0%
Sequence logo 2.0bits 0.0bits
5,960 5,980 6,000 6,020
AJ489607 consensus ACTACTAACA GACCGCAACC TAAACACAAC TTTCTTTGAT CCCGCTGGAG GAGGGGACCC AATTCTCTAC 6020
Quality scores 64 0
AJ489607 consensus ACTACTAACA GACCGCAACC TAAACACAAC TTTCTTTGAT CCCGCTGGAG GAGGGGACCC AATTCTCTAC 6020
Quality scores 64 0
AJ489607 consensus ACTACTAACA GACCGCAACC TAAACACAAC TTTCTTTGAT CCCGCTGGAG GAGGGGACCC AATTCTCTAC 6020
Quality scores 64 0
AJ489607 consensus ACTACTAACA GACCGCAACC TAAACACAAC TTTCTTTGAT CCCGCTGGAG GAGGGGACCC AATTCTCTAC 6020
Quality scores 64 0
AJ489607 consensus ACTACTAACA GACCGCAACC TAAACACAAC TTTCTTTGAT CCCGCTGGAG GAGGGGACCC AATTCTCTAC 6020
Quality scores 64 0
AJ489607 consensus ACTACTAACA GACCGCAACC TAAACACAAC TTTCTTTGAT CCCGCTGGAG GAGGGGACCC AATTCTCTAC 6020
Quality scores 64 0
AJ489607 consensus ACTACTAACA GACCGCAACC TAAACACAAC TTTCTTTGAT CCCGCTGGAG GAGGGGACCC AATTCTCTAC 6020
Quality scores 64 0
AJ489607 consensus ACTACTAACA GACCGCAACC TAAACACAAC TTTCTTTGAT CCCGCTGGAG GAGGGGACCC AATTCTCTAC 6020
Quality scores 64 0
AJ489607 consensus ACTACTAACA GACCGCAACC TAAACACAAC TTTCTTTGAT CCCGCTGGAG GAGGGGACCC AATTCTCTAC 6020
Quality scores 64 0
Consensus ACTACTAACA GACCGCAACC TAAACACAAC TTTCTTTGAT CCCGCTGGAG GAGGGGACCC AATTCTCTAC
Conservation 100% 0%
Sequence logo 2.0bits 0.0bits

0.0bits

6,040 6,060 6,080

AJ489607 consensus CAGCATCTGT TCTGATTCTT TGGGCACCCA GAAGTTTATA TTCTTATCCT CCCAGGATTT GGAATTATTT 6090
64
Quality scores
0
AJ489607 consensus CAGCATCTGT TCTGATTCTT TGGGCACCCA GAAGTTTATA TTCTTATCCT CCCAGGATTT GGAATTATTT 6090
64
Quality scores
0
AJ489607 consensus CAGCATCTGT TCTGATTCTT TGGGCACCCA GAAGTTTATA TTCTTATCCT CCCAGGATTT GGAATTATTT 6090
64
Quality scores
0
AJ489607 consensus CAGCATCTGT TCTGATTCTT TGGGCACCCA GAAGTTTATA TTCTTATCCT CCCAGGATTT GGAATTATTT 6090
64
Quality scores
0
AJ489607 consensus CAGCATCTGT TCTGATTCTT TGGGCACCCA GAAGTTTATA TTCTTATCCT CCCAGGATTT GGAATTATTT 6090
64
Quality scores
0
AJ489607 consensus CAGCATCTGT TCTGATTCTT TGGGCACCCA GAAGTTTATA TTCTTATCCT CCCAGGATTT GGAATTATTT 6090
64
Quality scores
0
AJ489607 consensus CAGCATCTGT TCTGATTCTT TGGGCACCCA GAAGTTTATA TTCTTATCCT CCCAGGATTT GGAATTATTT 6090
64
Quality scores
0
AJ489607 consensus CAGCATCTGT TCTGATTCTT TGGGCACCCA GAAGTTTATA TTCTTATCCT CCCAGGATTT GGAATTATTT 6090
64
Quality scores
0
AJ489607 consensus CAGCATCTGT TCTGATTCTT TGGGCACCCA GAAGTTTATA TTCTTATCCT CCCAGGATTT GGAATTATTT 6090
64
Quality scores
0
Consensus CAGCATCTGT TCTGATTCTT TGGGCACCCA GAAGTTTATA TTCTTATCCT CCCAGGATTT GGAATTATTT
100%
Conservation
0%
2.0bits
Sequence logo CAGCATCTGT TCTGATTCTT TGGGCACCCA GAAGTTTATA TTCTTATCCT CCCAGGATTT GGAATTATTT
0.0bits

6,100 6,120 6,140 6,160

AJ489607 consensus CACATGTAGT TACTTACTAC TCCGGAAAAA AAGAACCTTT CGGCTATATA GGAATAGTAT GAGCAATAAT 6160
64
Quality scores
0
AJ489607 consensus CACATGTAGT TACTTACTAC TCCGGAAAAA AAGAACCTTT CGGCTATATA GGAATAGTAT GAGCAATAAT 6160
64
Quality scores
0
AJ489607 consensus CACATGTAGT TACTTACTAC TCCGGAAAAA AAGAACCTTT CGGCTATATA GGAATAGTAT GAGCAATAAT 6160
64
Quality scores
0
AJ489607 consensus CACATGTAGT TACTTACTAC TCCGGAAAAA AAGAACCTTT CGGCTATATA GGAATAGTAT GAGCAATAAT 6160
64
Quality scores
0
AJ489607 consensus CACATGTAGT TACTTACTAC TCCGGAAAAA AAGAACCTTT CGGCTATATA GGAATAGTAT GAGCAATAAT 6160
64
Quality scores
0
AJ489607 consensus CACATGTAGT TACTTACTAC TCCGGAAAAA AAGAACCTTT CGGCTATATA GGAATAGTAT GAGCAATAAT 6160
64
Quality scores
0
AJ489607 consensus CACATGTAGT TACTTACTAC TCCGGAAAAA AAGAACCTTT CGGCTATATA GGAATAGTAT GAGCAATAAT 6160
64
Quality scores
0
AJ489607 consensus CACATGTAGT TACTTACTAC TCCGGAAAAA AAGAACCTTT CGGCTATATA GGAATAGTAT GAGCAATAAT 6160
64
Quality scores
0
AJ489607 consensus CACATGTAGT TACTTACTAC TCCGGAAAAA AAGAACCTTT CGGCTATATA GGAATAGTAT GAGCAATAAT 6160
64
Quality scores
0
Consensus CACATGTAGT TACTTACTAC TCCGGAAAAA AAGAACCTTT CGGCTATATA GGAATAGTAT GAGCAATAAT
100%
Conservation
0%
2.0bits
Sequence logo CACATGTAGT TACTTACTAC TCCGGAAAAA AAGAACCTTT CGGCTATATA GGAATAGTAT GAGCAATAAT
0.0bits

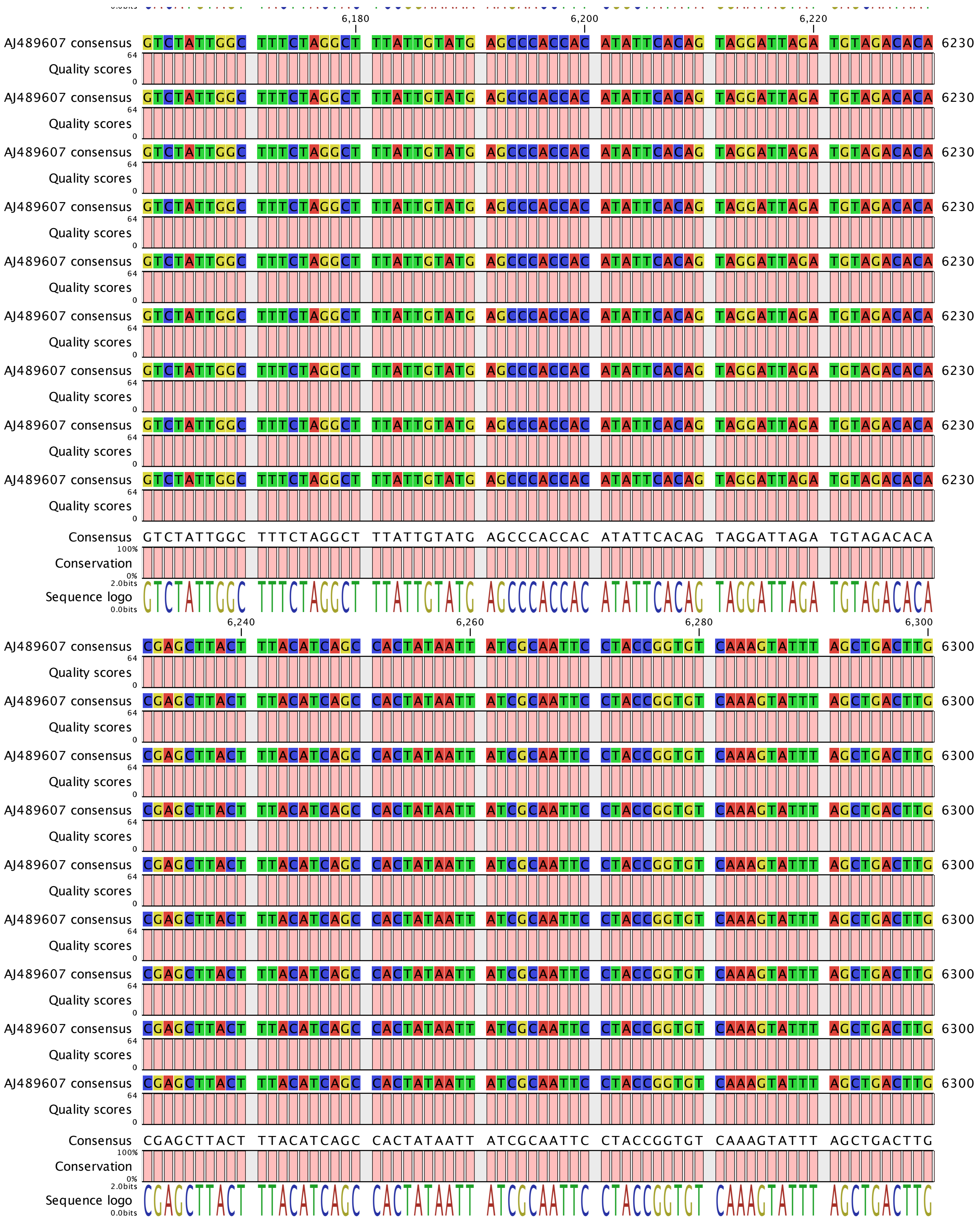
6,180
6,200
6,220
AJ489607 consensus GTCTATTGGC TTTCTAGGCT TTATTGTATG AGCCCACCAC ATATTCACAG TAGGATTAGA TGTAGACACA 6230
64
Quality scores
0
AJ489607 consensus GTCTATTGGC TTTCTAGGCT TTATTGTATG AGCCCACCAC ATATTCACAG TAGGATTAGA TGTAGACACA 6230
64
Quality scores
0
AJ489607 consensus GTCTATTGGC TTTCTAGGCT TTATTGTATG AGCCCACCAC ATATTCACAG TAGGATTAGA TGTAGACACA 6230
64
Quality scores
0
AJ489607 consensus GTCTATTGGC TTTCTAGGCT TTATTGTATG AGCCCACCAC ATATTCACAG TAGGATTAGA TGTAGACACA 6230
64
Quality scores
0
AJ489607 consensus GTCTATTGGC TTTCTAGGCT TTATTGTATG AGCCCACCAC ATATTCACAG TAGGATTAGA TGTAGACACA 6230
64
Quality scores
0
AJ489607 consensus GTCTATTGGC TTTCTAGGCT TTATTGTATG AGCCCACCAC ATATTCACAG TAGGATTAGA TGTAGACACA 6230
64
Quality scores
0
AJ489607 consensus GTCTATTGGC TTTCTAGGCT TTATTGTATG AGCCCACCAC ATATTCACAG TAGGATTAGA TGTAGACACA 6230
64
Quality scores
0
AJ489607 consensus GTCTATTGGC TTTCTAGGCT TTATTGTATG AGCCCACCAC ATATTCACAG TAGGATTAGA TGTAGACACA 6230
64
Quality scores
0
AJ489607 consensus GTCTATTGGC TTTCTAGGCT TTATTGTATG AGCCCACCAC ATATTCACAG TAGGATTAGA TGTAGACACA 6230
64
Quality scores
0
Consensus GTCTATTGGC TTTCTAGGCT TTATTGTATG AGCCCACCAC ATATTCACAG TAGGATTAGA TGTAGACACA
100%
Conservation
0%
2.0bits
Sequence logo GTCTATTGGC TTTCTAGGCT TTATTGTATG AGCCCACCAC ATATTCACAG TAGGATTAGA TGTAGACACA
0.0bits
6,240
6,260
6,280
6,300
AJ489607 consensus CGAGCTTACT TTACATCAGC CACTATAATT ATCGCAATTC CTACCGGTGT CAAAGTATTT AGCTGACTTG 6300
64
Quality scores
0
AJ489607 consensus CGAGCTTACT TTACATCAGC CACTATAATT ATCGCAATTC CTACCGGTGT CAAAGTATTT AGCTGACTTG 6300
64
Quality scores
0
AJ489607 consensus CGAGCTTACT TTACATCAGC CACTATAATT ATCGCAATTC CTACCGGTGT CAAAGTATTT AGCTGACTTG 6300
64
Quality scores
0
AJ489607 consensus CGAGCTTACT TTACATCAGC CACTATAATT ATCGCAATTC CTACCGGTGT CAAAGTATTT AGCTGACTTG 6300
64
Quality scores
0
AJ489607 consensus CGAGCTTACT TTACATCAGC CACTATAATT ATCGCAATTC CTACCGGTGT CAAAGTATTT AGCTGACTTG 6300
64
Quality scores
0
AJ489607 consensus CGAGCTTACT TTACATCAGC CACTATAATT ATCGCAATTC CTACCGGTGT CAAAGTATTT AGCTGACTTG 6300
64
Quality scores
0
AJ489607 consensus CGAGCTTACT TTACATCAGC CACTATAATT ATCGCAATTC CTACCGGTGT CAAAGTATTT AGCTGACTTG 6300
64
Quality scores
0
AJ489607 consensus CGAGCTTACT TTACATCAGC CACTATAATT ATCGCAATTC CTACCGGTGT CAAAGTATTT AGCTGACTTG 6300
64
Quality scores
0
AJ489607 consensus CGAGCTTACT TTACATCAGC CACTATAATT ATCGCAATTC CTACCGGTGT CAAAGTATTT AGCTGACTTG 6300
64
Quality scores
0
Consensus CGAGCTTACT TTACATCAGC CACTATAATT ATCGCAATTC CTACCGGTGT CAAAGTATTT AGCTGACTTG
100%
Conservation
0%
2.0bits
Sequence logo CGAGCTTACT TTACATCAGC CACTATAATT ATCGCAATTC CTACCGGTGT CAAAGTATTT AGCTGACTTG
0.0bits

0.0bits

6,320 6,340 6,360

AJ489607 consensus CAACCCTACA CGGAGGTAAT ATTAAATGAT CTCCAGCTAT ACTATGAGCC TTAGGCTTTA TTTTCTTATT 6370
Quality scores 64 0
AJ489607 consensus CAACCCTACA CGGAGGTAAT ATTAAATGAT CTCCAGCTAT ACTATGAGCC TTAGGCTTTA TTTTCTTATT 6370
Quality scores 64 0
AJ489607 consensus CAACCCTACA CGGAGGTAAT ATTAAATGAT CTCCAGCTAT ACTATGAGCC TTAGGCTTTA TTTTCTTATT 6370
Quality scores 64 0
AJ489607 consensus CAACCCTACA CGGAGGTAAT ATTAAATGAT CTCCAGCTAT ACTATGAGCC TTAGGCTTTA TTTTCTTATT 6370
Quality scores 64 0
AJ489607 consensus CAACCCTACA CGGAGGTAAT ATTAAATGAT CTCCAGCTAT ACTATGAGCC TTAGGCTTTA TTTTCTTATT 6370
Quality scores 64 0
AJ489607 consensus CAACCCTACA CGGAGGTAAT ATTAAATGAT CTCCAGCTAT ACTATGAGCC TTAGGCTTTA TTTTCTTATT 6370
Quality scores 64 0
AJ489607 consensus CAACCCTACA CGGAGGTAAT ATTAAATGAT CTCCAGCTAT ACTATGAGCC TTAGGCTTTA TTTTCTTATT 6370
Quality scores 64 0
AJ489607 consensus CAACCCTACA CGGAGGTAAT ATTAAATGAT CTCCAGCTAT ACTATGAGCC TTAGGCTTTA TTTTCTTATT 6370
Quality scores 64 0
AJ489607 consensus CAACCCTACA CGGAGGTAAT ATTAAATGAT CTCCAGCTAT ACTATGAGCC TTAGGCTTTA TTTTCTTATT 6370
Quality scores 64 0
Consensus CAACCCTACA CGGAGGTAAT ATTAAATGAT CTCCAGCTAT ACTATGAGCC TTAGGCTTTA TTTTCTTATT
Conservation 100% 0%
Sequence logo 2.0bits 0.0bits CAACCCTACA CGGAGGTAAT ATTAAATGAT CTCCAGCTAT ACTATGAGCC TTAGGCTTTA TTTTCTTATT

6,380 6,400 6,420 6,440

AJ489607 consensus TACAGTTGGT GGTCTAACCG GAATTGTTTT ATCCAACTCA TCCCTTGACA TCGTGCTTCA CGATACATAC 6440
Quality scores 64 0
AJ489607 consensus TACAGTTGGT GGTCTAACCG GAATTGTTTT ATCCAACTCA TCCCTTGACA TCGTGCTTCA CGATACATAC 6440
Quality scores 64 0
AJ489607 consensus TACAGTTGGT GGTCTAACCG GAATTGTTTT ATCCAACTCA TCCCTTGACA TCGTGCTTCA CGATACATAC 6440
Quality scores 64 0
AJ489607 consensus TACAGTTGGT GGTCTAACCG GAATTGTTTT ATCCAACTCA TCCCTTGACA TCGTGCTTCA CGATACATAC 6440
Quality scores 64 0
AJ489607 consensus TACAGTTGGT GGTCTAACCG GAATTGTTTT ATCCAACTCA TCCCTTGACA TCGTGCTTCA CGATACATAC 6440
Quality scores 64 0
AJ489607 consensus TACAGTTGGT GGTCTAACCG GAATTGTTTT ATCCAACTCA TCCCTTGACA TCGTGCTTCA CGATACATAC 6440
Quality scores 64 0
AJ489607 consensus TACAGTTGGT GGTCTAACCG GAATTGTTTT ATCCAACTCA TCCCTTGACA TCGTGCTTCA CGATACATAC 6440
Quality scores 64 0
AJ489607 consensus TACAGTTGGT GGTCTAACCG GAATTGTTTT ATCCAACTCA TCCCTTGACA TCGTGCTTCA CGATACATAC 6440
Quality scores 64 0
AJ489607 consensus TACAGTTGGT GGTCTAACCG GAATTGTTTT ATCCAACTCA TCCCTTGACA TCGTGCTTCA CGATACATAC 6440
Quality scores 64 0
Consensus TACAGTTGGT GGTCTAACCG GAATTGTTTT ATCCAACTCA TCCCTTGACA TCGTGCTTCA CGATACATAC
Conservation 100% 0%
Sequence logo 2.0bits 0.0bits TACAGTTGGT GGTCTAACCG GAATTGTTTT ATCCAACTCA TCCCTTGACA TCGTGCTTCA CGATACATAC

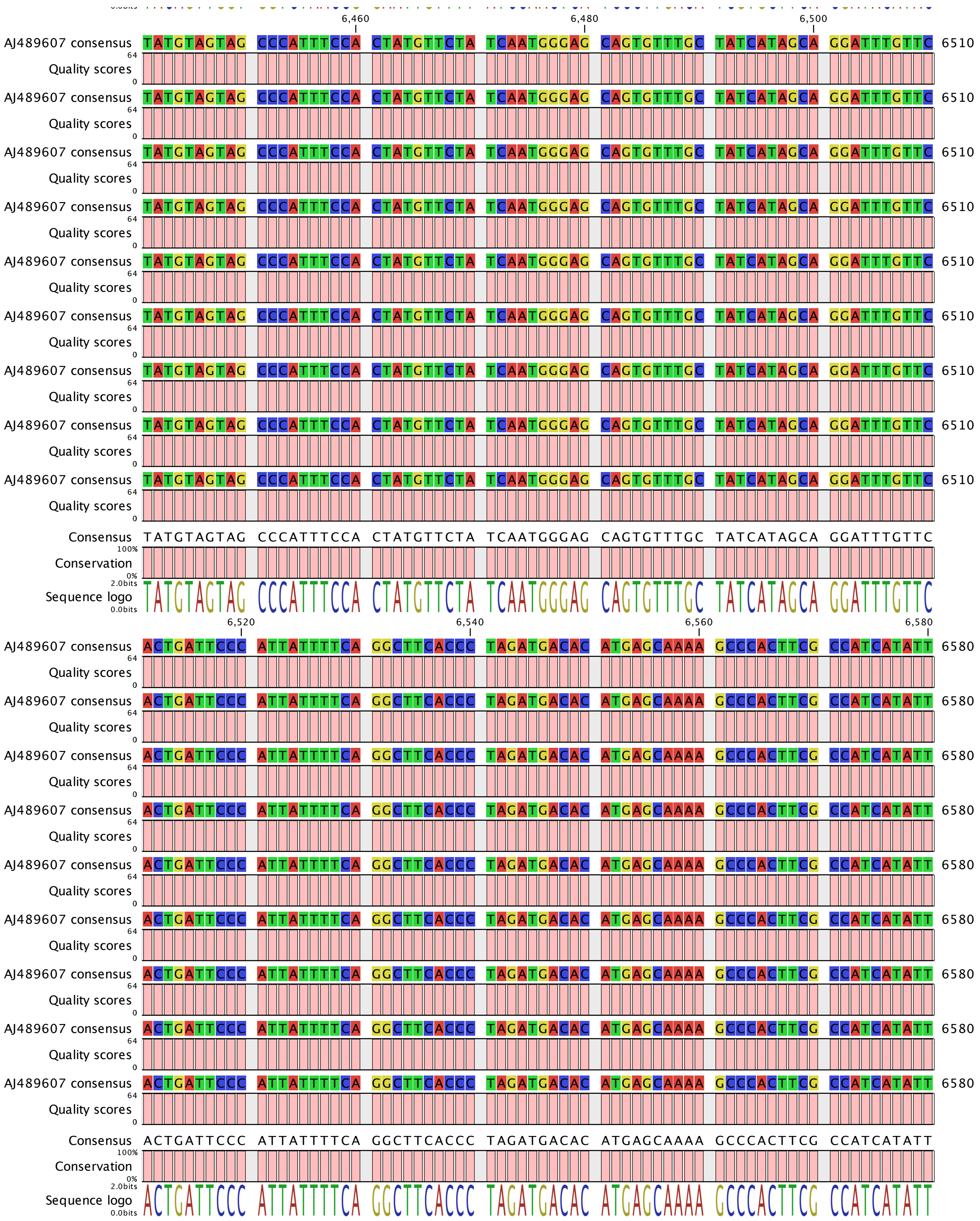
6,460 6,480 6,500
AJ489607 consensus TATGTAGTAG CCCATTTCCA CTATGTTCTA TCAATGGGAG CAGTGTTTGC TATCATAGCA GGATTTGTTC 6510
Quality scores
AJ489607 consensus TATGTAGTAG CCCATTTCCA CTATGTTCTA TCAATGGGAG CAGTGTTTGC TATCATAGCA GGATTTGTTC 6510
Quality scores
AJ489607 consensus TATGTAGTAG CCCATTTCCA CTATGTTCTA TCAATGGGAG CAGTGTTTGC TATCATAGCA GGATTTGTTC 6510
Quality scores
AJ489607 consensus TATGTAGTAG CCCATTTCCA CTATGTTCTA TCAATGGGAG CAGTGTTTGC TATCATAGCA GGATTTGTTC 6510
Quality scores
AJ489607 consensus TATGTAGTAG CCCATTTCCA CTATGTTCTA TCAATGGGAG CAGTGTTTGC TATCATAGCA GGATTTGTTC 6510
Quality scores
AJ489607 consensus TATGTAGTAG CCCATTTCCA CTATGTTCTA TCAATGGGAG CAGTGTTTGC TATCATAGCA GGATTTGTTC 6510
Quality scores
AJ489607 consensus TATGTAGTAG CCCATTTCCA CTATGTTCTA TCAATGGGAG CAGTGTTTGC TATCATAGCA GGATTTGTTC 6510
Quality scores
AJ489607 consensus TATGTAGTAG CCCATTTCCA CTATGTTCTA TCAATGGGAG CAGTGTTTGC TATCATAGCA GGATTTGTTC 6510
Quality scores
AJ489607 consensus TATGTAGTAG CCCATTTCCA CTATGTTCTA TCAATGGGAG CAGTGTTTGC TATCATAGCA GGATTTGTTC 6510
Quality scores
Consensus TATGTAGTAG CCCATTTCCA CTATGTTCTA TCAATGGGAG CAGTGTTTGC TATCATAGCA GGATTTGTTC
Conservation
Sequence logo
6,520 6,540 6,560 6,580
AJ489607 consensus ACTGATTCCC ATTATTTTCA GGCTTCACCC TAGATGACAC ATGAGCAAAA GCCCACTTCG CCATCATATT 6580
Quality scores
AJ489607 consensus ACTGATTCCC ATTATTTTCA GGCTTCACCC TAGATGACAC ATGAGCAAAA GCCCACTTCG CCATCATATT 6580
Quality scores
AJ489607 consensus ACTGATTCCC ATTATTTTCA GGCTTCACCC TAGATGACAC ATGAGCAAAA GCCCACTTCG CCATCATATT 6580
Quality scores
AJ489607 consensus ACTGATTCCC ATTATTTTCA GGCTTCACCC TAGATGACAC ATGAGCAAAA GCCCACTTCG CCATCATATT 6580
Quality scores
AJ489607 consensus ACTGATTCCC ATTATTTTCA GGCTTCACCC TAGATGACAC ATGAGCAAAA GCCCACTTCG CCATCATATT 6580
Quality scores
AJ489607 consensus ACTGATTCCC ATTATTTTCA GGCTTCACCC TAGATGACAC ATGAGCAAAA GCCCACTTCG CCATCATATT 6580
Quality scores
AJ489607 consensus ACTGATTCCC ATTATTTTCA GGCTTCACCC TAGATGACAC ATGAGCAAAA GCCCACTTCG CCATCATATT 6580
Quality scores
AJ489607 consensus ACTGATTCCC ATTATTTTCA GGCTTCACCC TAGATGACAC ATGAGCAAAA GCCCACTTCG CCATCATATT 6580
Quality scores
AJ489607 consensus ACTGATTCCC ATTATTTTCA GGCTTCACCC TAGATGACAC ATGAGCAAAA GCCCACTTCG CCATCATATT 6580
Quality scores
Consensus ACTGATTCCC ATTATTTTCA GGCTTCACCC TAGATGACAC ATGAGCAAAA GCCCACTTCG CCATCATATT
Conservation
Sequence logo

6,600 6,620 6,640

AJ489607 consensus CGTAGGAGTA AACATAACAT TCTTCCCTCA ACATTTCCTG GGCCTTTCAG GAATACCACG ACGCTACTCA 6650
64 Quality scores 0
AJ489607 consensus CGTAGGAGTA AACATAACAT TCTTCCCTCA ACATTTCCTG GGCCTTTCAG GAATACCACG ACGCTACTCA 6650
64 Quality scores 0
AJ489607 consensus CGTAGGAGTA AACATAACAT TCTTCCCTCA ACATTTCCTG GGCCTTTCAG GAATACCACG ACGCTACTCA 6650
64 Quality scores 0
AJ489607 consensus CGTAGGAGTA AACATAACAT TCTTCCCTCA ACATTTCCTG GGCCTTTCAG GAATACCACG ACGCTACTCA 6650
64 Quality scores 0
AJ489607 consensus CGTAGGAGTA AACATAACAT TCTTCCCTCA ACATTTCCTG GGCCTTTCAG GAATACCACG ACGCTACTCA 6650
64 Quality scores 0
AJ489607 consensus CGTAGGAGTA AACATAACAT TCTTCCCTCA ACATTTCCTG GGCCTTTCAG GAATACCACG ACGCTACTCA 6650
64 Quality scores 0
AJ489607 consensus CGTAGGAGTA AACATAACAT TCTTCCCTCA ACATTTCCTG GGCCTTTCAG GAATACCACG ACGCTACTCA 6650
64 Quality scores 0
AJ489607 consensus CGTAGGAGTA AACATAACAT TCTTCCCTCA ACATTTCCTG GGCCTTTCAG GAATACCACG ACGCTACTCA 6650
64 Quality scores 0
AJ489607 consensus CGTAGGAGTA AACATAACAT TCTTCCCTCA ACATTTCCTG GGCCTTTCAG GAATACCACG ACGCTACTCA 6650
64 Quality scores 0
Consensus CGTAGGAGTA AACATAACAT TCTTCCCTCA ACATTTCCTG GGCCTTTCAG GAATACCACG ACGCTACTCA
100% Conservation 0%
2.0bits Sequence logo 0.0bits CGTAGGAGTA AACATAACAT TCTTCCCTCA ACATTTCCTG GGCCTTTCAG GAATACCACG ACGCTACTCA

6,660 6,680 6,700 6,720

AJ489607 consensus GACTACCCAG ATGCTTACAC CACATGAAAC ACTGTCTCTT CTATAGGATC ATTTATTTCA CTAACAGCTG 6720
64 Quality scores 0
AJ489607 consensus GACTACCCAG ATGCTTACAC CACATGAAAC ACTGTCTCTT CTATAGGATC ATTTATTTCA CTAACAGCTG 6720
64 Quality scores 0
AJ489607 consensus GACTACCCAG ATGCTTACAC CACATGAAAC ACTGTCTCTT CTATAGGATC ATTTATTTCA CTAACAGCTG 6720
64 Quality scores 0
AJ489607 consensus GACTACCCAG ATGCTTACAC CACATGAAAC ACTGTCTCTT CTATAGGATC ATTTATTTCA CTAACAGCTG 6720
64 Quality scores 0
AJ489607 consensus GACTACCCAG ATGCTTACAC CACATGAAAC ACTGTCTCTT CTATAGGATC ATTTATTTCA CTAACAGCTG 6720
64 Quality scores 0
AJ489607 consensus GACTACCCAG ATGCTTACAC CACATGAAAC ACTGTCTCTT CTATAGGATC ATTTATTTCA CTAACAGCTG 6720
64 Quality scores 0
AJ489607 consensus GACTACCCAG ATGCTTACAC CACATGAAAC ACTGTCTCTT CTATAGGATC ATTTATTTCA CTAACAGCTG 6720
64 Quality scores 0
AJ489607 consensus GACTACCCAG ATGCTTACAC CACATGAAAC ACTGTCTCTT CTATAGGATC ATTTATTTCA CTAACAGCTG 6720
64 Quality scores 0
AJ489607 consensus GACTACCCAG ATGCTTACAC CACATGAAAC ACTGTCTCTT CTATAGGATC ATTTATTTCA CTAACAGCTG 6720
64 Quality scores 0
Consensus GACTACCCAG ATGCTTACAC CACATGAAAC ACTGTCTCTT CTATAGGATC ATTTATTTCA CTAACAGCTG
100% Conservation 0%
2.0bits Sequence logo 0.0bits GACTACCCAG ATGCTTACAC CACATGAAAC ACTGTCTCTT CTATAGGATC ATTTATTTCA CTAACAGCTG

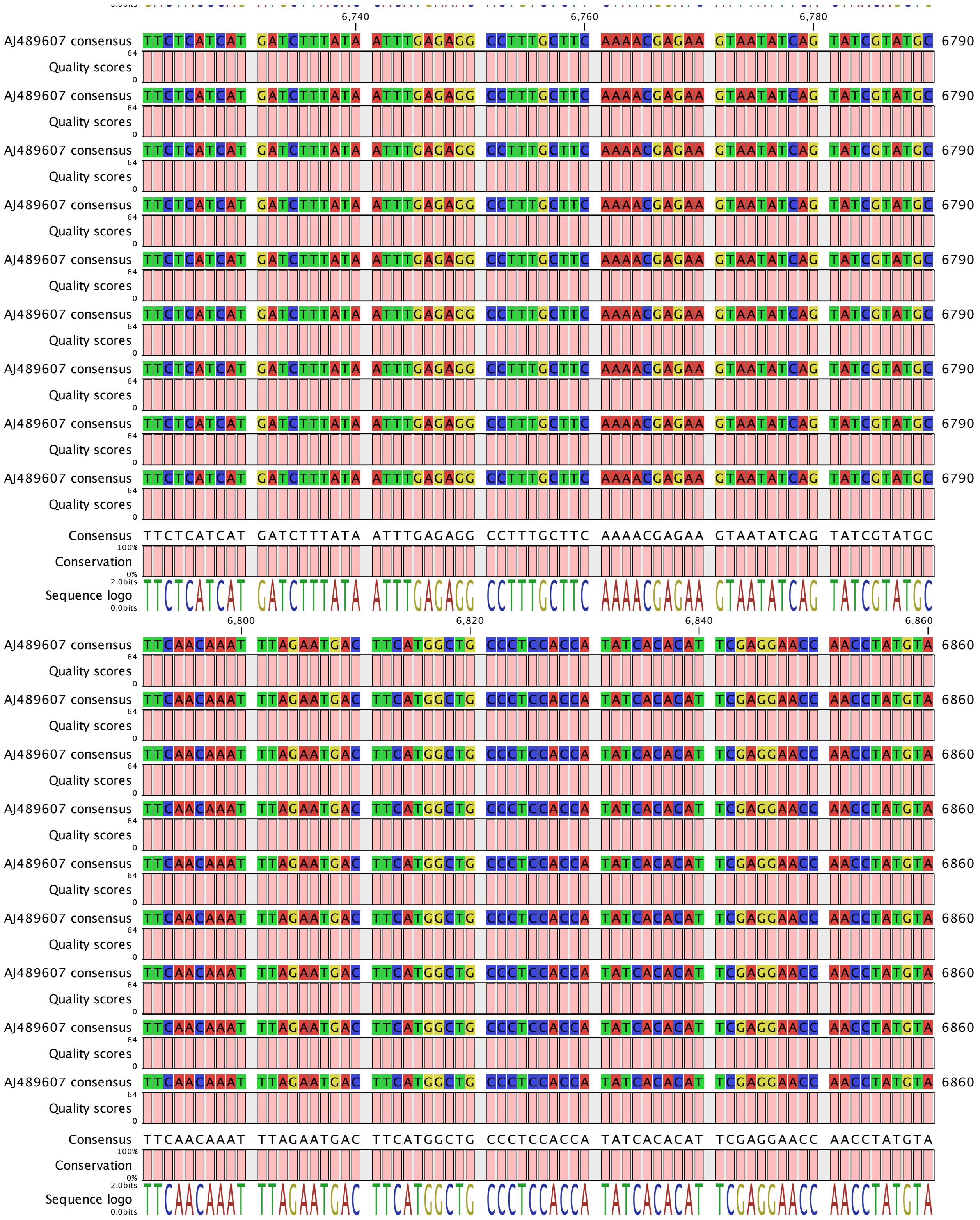
6,740
6,760
6,780
AJ489607 consensus TTCTCATCAT GATCTTTATA ATTTGAGAGG CCTTTGCTTC AAAACGAGAA GTAATATCAG TATCGTATGC 6790
Quality scores
Consensus TTCTCATCAT GATCTTTATA ATTTGAGAGG CCTTTGCTTC AAAACGAGAA GTAATATCAG TATCGTATGC
Conservation
Sequence logo
6,800
6,820
6,840
6,860
AJ489607 consensus TTCAACAAAT TTAGAATGAC TTCATGGCTG CCCTCCACCA TATCACACAT TCGAGGAACC AACCTATGTA 6860
Quality scores
Consensus TTCAACAAAT TTAGAATGAC TTCATGGCTG CCCTCCACCA TATCACACAT TCGAGGAACC AACCTATGTA
Conservation
Sequence logo

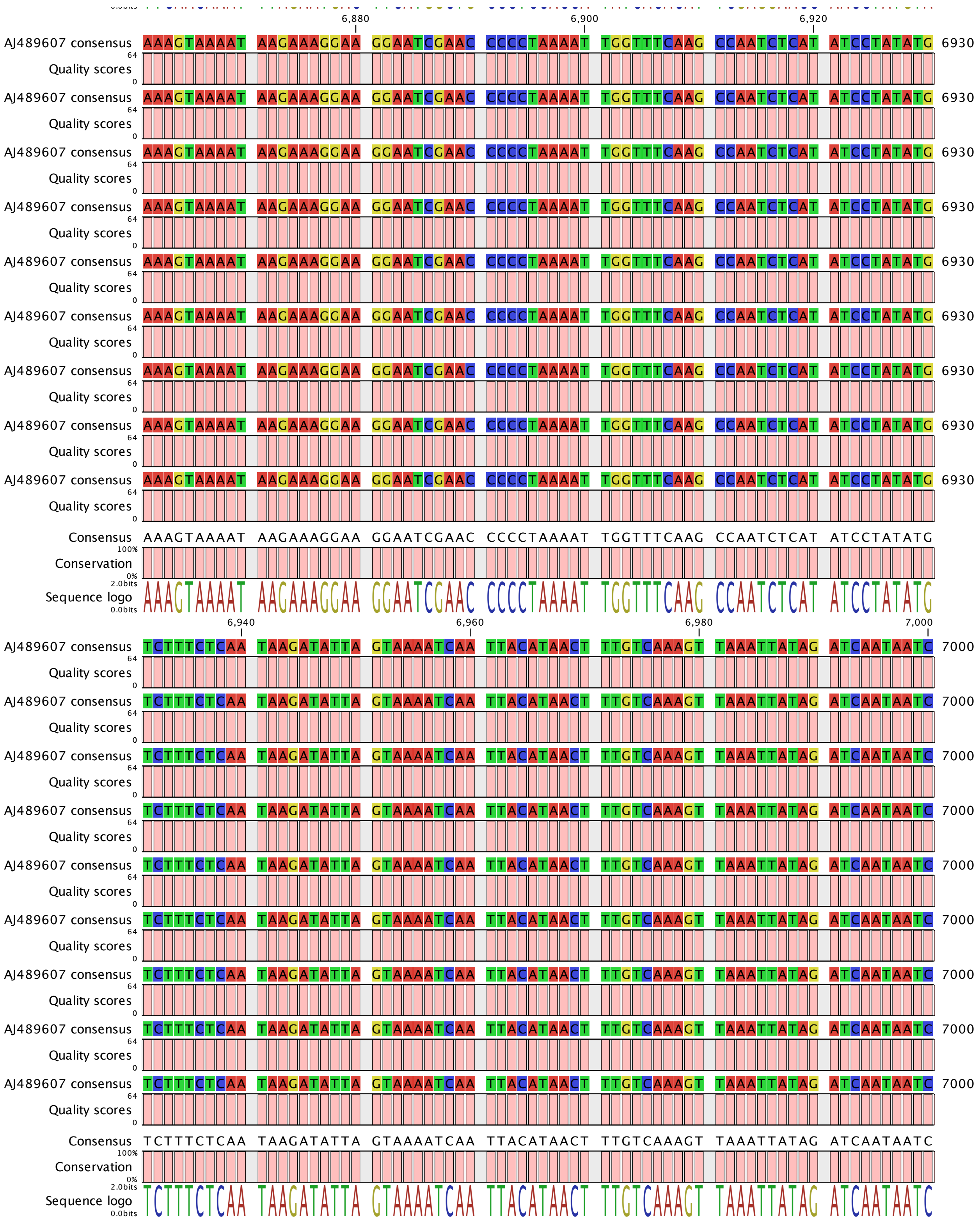
6,880 6,900 6,920
AJ489607 consensus AAAGTAAAAT AAGAAAGGAA GGAATCGAAC CCCCTAAAAT TGGTTTCAAG CCAATCTCAT ATCCTATATG 6930
Quality scores
AJ489607 consensus AAAGTAAAAT AAGAAAGGAA GGAATCGAAC CCCCTAAAAT TGGTTTCAAG CCAATCTCAT ATCCTATATG 6930
Quality scores
AJ489607 consensus AAAGTAAAAT AAGAAAGGAA GGAATCGAAC CCCCTAAAAT TGGTTTCAAG CCAATCTCAT ATCCTATATG 6930
Quality scores
AJ489607 consensus AAAGTAAAAT AAGAAAGGAA GGAATCGAAC CCCCTAAAAT TGGTTTCAAG CCAATCTCAT ATCCTATATG 6930
Quality scores
AJ489607 consensus AAAGTAAAAT AAGAAAGGAA GGAATCGAAC CCCCTAAAAT TGGTTTCAAG CCAATCTCAT ATCCTATATG 6930
Quality scores
AJ489607 consensus AAAGTAAAAT AAGAAAGGAA GGAATCGAAC CCCCTAAAAT TGGTTTCAAG CCAATCTCAT ATCCTATATG 6930
Quality scores
AJ489607 consensus AAAGTAAAAT AAGAAAGGAA GGAATCGAAC CCCCTAAAAT TGGTTTCAAG CCAATCTCAT ATCCTATATG 6930
Quality scores
AJ489607 consensus AAAGTAAAAT AAGAAAGGAA GGAATCGAAC CCCCTAAAAT TGGTTTCAAG CCAATCTCAT ATCCTATATG 6930
Quality scores
AJ489607 consensus AAAGTAAAAT AAGAAAGGAA GGAATCGAAC CCCCTAAAAT TGGTTTCAAG CCAATCTCAT ATCCTATATG 6930
Quality scores
Consensus AAAGTAAAAT AAGAAAGGAA GGAATCGAAC CCCCTAAAAT TGGTTTCAAG CCAATCTCAT ATCCTATATG
Conservation
Sequence logo AAAGTAAAAT AAGAAAGGAA GGAATCGAAC CCCCTAAAAT TGGTTTCAAG CCAATCTCAT ATCCTATATG
6,940 6,960 6,980 7,000
AJ489607 consensus TCTTTCTCAA TAAGATATTA GTAAAATCAA TTACATAACT TTGTCAAAGT TAAATTATAG ATCAATAATC 7000
Quality scores
AJ489607 consensus TCTTTCTCAA TAAGATATTA GTAAAATCAA TTACATAACT TTGTCAAAGT TAAATTATAG ATCAATAATC 7000
Quality scores
AJ489607 consensus TCTTTCTCAA TAAGATATTA GTAAAATCAA TTACATAACT TTGTCAAAGT TAAATTATAG ATCAATAATC 7000
Quality scores
AJ489607 consensus TCTTTCTCAA TAAGATATTA GTAAAATCAA TTACATAACT TTGTCAAAGT TAAATTATAG ATCAATAATC 7000
Quality scores
AJ489607 consensus TCTTTCTCAA TAAGATATTA GTAAAATCAA TTACATAACT TTGTCAAAGT TAAATTATAG ATCAATAATC 7000
Quality scores
AJ489607 consensus TCTTTCTCAA TAAGATATTA GTAAAATCAA TTACATAACT TTGTCAAAGT TAAATTATAG ATCAATAATC 7000
Quality scores
AJ489607 consensus TCTTTCTCAA TAAGATATTA GTAAAATCAA TTACATAACT TTGTCAAAGT TAAATTATAG ATCAATAATC 7000
Quality scores
AJ489607 consensus TCTTTCTCAA TAAGATATTA GTAAAATCAA TTACATAACT TTGTCAAAGT TAAATTATAG ATCAATAATC 7000
Quality scores
AJ489607 consensus TCTTTCTCAA TAAGATATTA GTAAAATCAA TTACATAACT TTGTCAAAGT TAAATTATAG ATCAATAATC 7000
Quality scores
Consensus TCTTTCTCAA TAAGATATTA GTAAAATCAA TTACATAACT TTGTCAAAGT TAAATTATAG ATCAATAATC
Conservation
Sequence logo TCTTTCTCAA TAAGATATTA GTAAAATCAA TTACATAACT TTGTCAAAGT TAAATTATAG ATCAATAATC

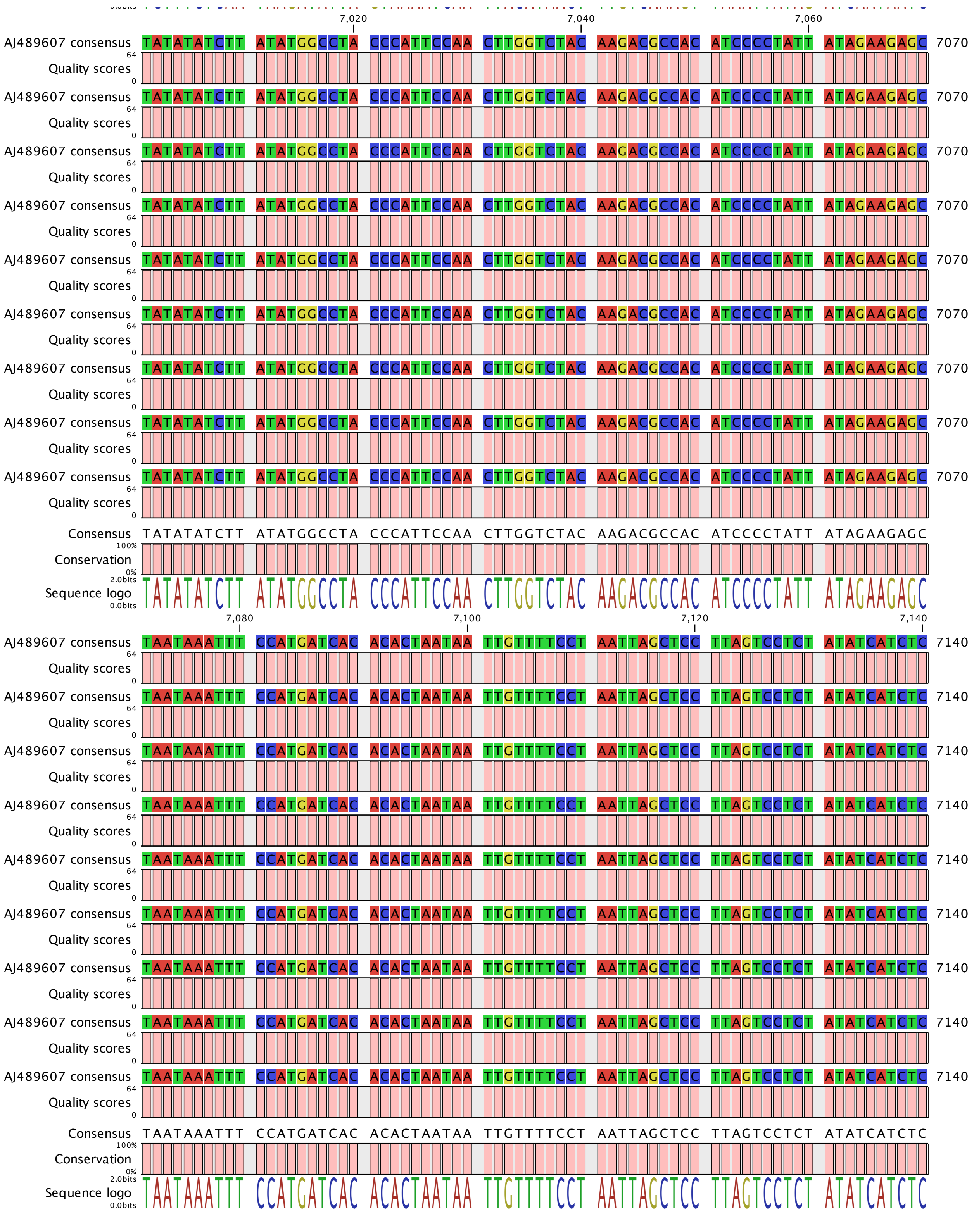

7,020 7,040 7,060
AJ489607 consensus TATATATCTT ATATGGCCTA CCCATTCCAA CTTGGTCTAC AAGACGCCAC ATCCCCTATT ATAGAAGAGC 7070
64 Quality scores 0
AJ489607 consensus TATATATCTT ATATGGCCTA CCCATTCCAA CTTGGTCTAC AAGACGCCAC ATCCCCTATT ATAGAAGAGC 7070
64 Quality scores 0
AJ489607 consensus TATATATCTT ATATGGCCTA CCCATTCCAA CTTGGTCTAC AAGACGCCAC ATCCCCTATT ATAGAAGAGC 7070
64 Quality scores 0
AJ489607 consensus TATATATCTT ATATGGCCTA CCCATTCCAA CTTGGTCTAC AAGACGCCAC ATCCCCTATT ATAGAAGAGC 7070
64 Quality scores 0
AJ489607 consensus TATATATCTT ATATGGCCTA CCCATTCCAA CTTGGTCTAC AAGACGCCAC ATCCCCTATT ATAGAAGAGC 7070
64 Quality scores 0
AJ489607 consensus TATATATCTT ATATGGCCTA CCCATTCCAA CTTGGTCTAC AAGACGCCAC ATCCCCTATT ATAGAAGAGC 7070
64 Quality scores 0
AJ489607 consensus TATATATCTT ATATGGCCTA CCCATTCCAA CTTGGTCTAC AAGACGCCAC ATCCCCTATT ATAGAAGAGC 7070
64 Quality scores 0
AJ489607 consensus TATATATCTT ATATGGCCTA CCCATTCCAA CTTGGTCTAC AAGACGCCAC ATCCCCTATT ATAGAAGAGC 7070
64 Quality scores 0
AJ489607 consensus TATATATCTT ATATGGCCTA CCCATTCCAA CTTGGTCTAC AAGACGCCAC ATCCCCTATT ATAGAAGAGC 7070
64 Quality scores 0
Consensus TATATATCTT ATATGGCCTA CCCATTCCAA CTTGGTCTAC AAGACGCCAC ATCCCCTATT ATAGAAGAGC
100% Conservation 0%
2.0bits Sequence logo 0.0bits
TATATATCTT ATATGGCCTA CCCATTCCAA CTTGGTCTAC AAGACGCCAC ATCCCCTATT ATAGAAGAGC
7,080 7,100 7,120 7,140
AJ489607 consensus TAATAAATTT CCATGATCAC ACACTAATAA TTGTTTTCCT AATTAGCTCC TTAGTCCTCT ATATCATCTC 7140
64 Quality scores 0
AJ489607 consensus TAATAAATTT CCATGATCAC ACACTAATAA TTGTTTTCCT AATTAGCTCC TTAGTCCTCT ATATCATCTC 7140
64 Quality scores 0
AJ489607 consensus TAATAAATTT CCATGATCAC ACACTAATAA TTGTTTTCCT AATTAGCTCC TTAGTCCTCT ATATCATCTC 7140
64 Quality scores 0
AJ489607 consensus TAATAAATTT CCATGATCAC ACACTAATAA TTGTTTTCCT AATTAGCTCC TTAGTCCTCT ATATCATCTC 7140
64 Quality scores 0
AJ489607 consensus TAATAAATTT CCATGATCAC ACACTAATAA TTGTTTTCCT AATTAGCTCC TTAGTCCTCT ATATCATCTC 7140
64 Quality scores 0
AJ489607 consensus TAATAAATTT CCATGATCAC ACACTAATAA TTGTTTTCCT AATTAGCTCC TTAGTCCTCT ATATCATCTC 7140
64 Quality scores 0
AJ489607 consensus TAATAAATTT CCATGATCAC ACACTAATAA TTGTTTTCCT AATTAGCTCC TTAGTCCTCT ATATCATCTC 7140
64 Quality scores 0
AJ489607 consensus TAATAAATTT CCATGATCAC ACACTAATAA TTGTTTTCCT AATTAGCTCC TTAGTCCTCT ATATCATCTC 7140
64 Quality scores 0
AJ489607 consensus TAATAAATTT CCATGATCAC ACACTAATAA TTGTTTTCCT AATTAGCTCC TTAGTCCTCT ATATCATCTC 7140
64 Quality scores 0
Consensus TAATAAATTT CCATGATCAC ACACTAATAA TTGTTTTCCT AATTAGCTCC TTAGTCCTCT ATATCATCTC
100% Conservation 0%
2.0bits Sequence logo 0.0bits
TAATAAATTT CCATGATCAC ACACTAATAA TTGTTTTCCT AATTAGCTCC TTAGTCCTCT ATATCATCTC

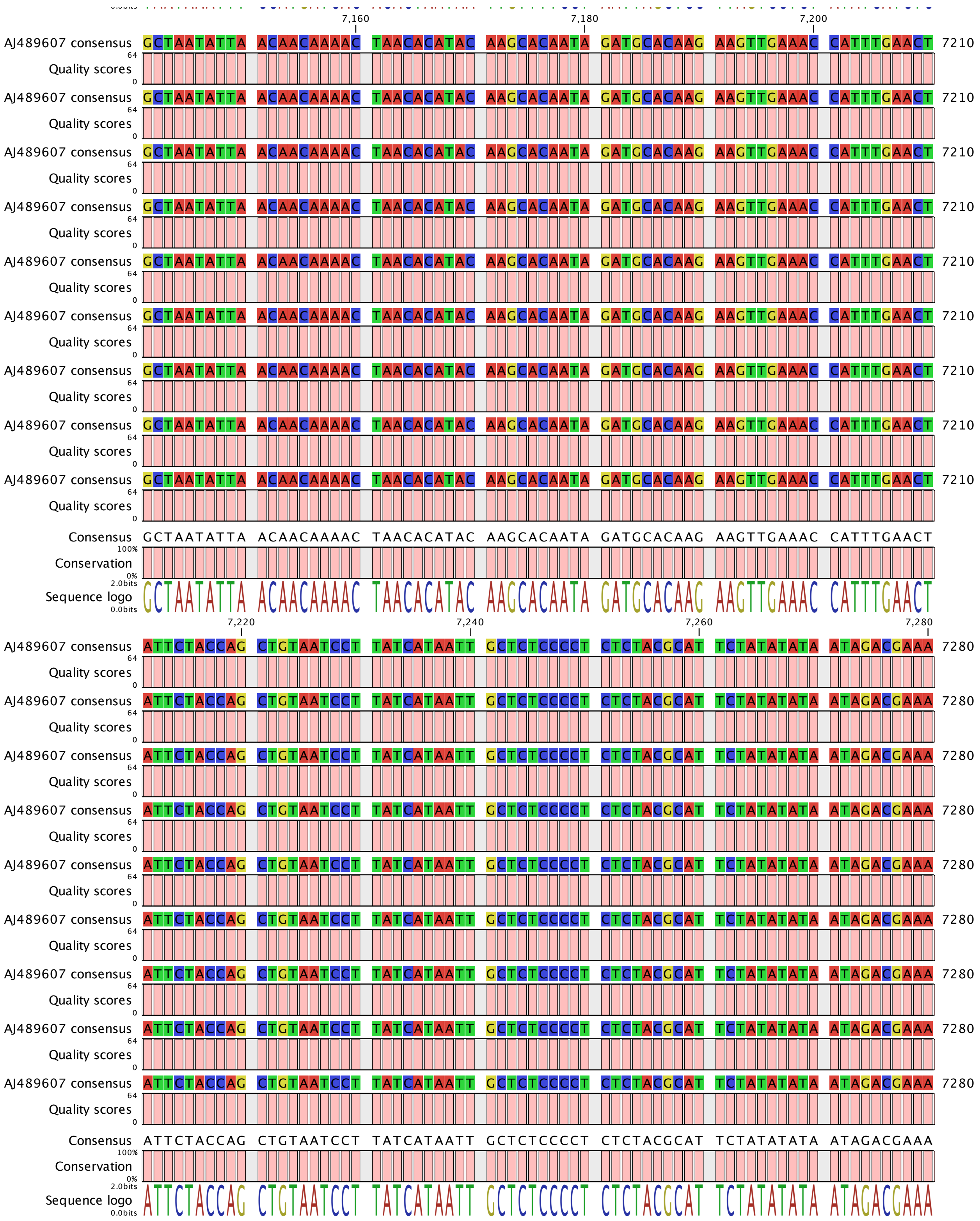
7,160
7,180
7,200
AJ489607 consensus GCTAATATTA ACAACAAAAC TAACACATAC AAGCACAATA GATGCACAAG AAGTTGAAAC CATTTGAACT 7210
Quality scores
AJ489607 consensus GCTAATATTA ACAACAAAAC TAACACATAC AAGCACAATA GATGCACAAG AAGTTGAAAC CATTTGAACT 7210
Quality scores
AJ489607 consensus GCTAATATTA ACAACAAAAC TAACACATAC AAGCACAATA GATGCACAAG AAGTTGAAAC CATTTGAACT 7210
Quality scores
AJ489607 consensus GCTAATATTA ACAACAAAAC TAACACATAC AAGCACAATA GATGCACAAG AAGTTGAAAC CATTTGAACT 7210
Quality scores
AJ489607 consensus GCTAATATTA ACAACAAAAC TAACACATAC AAGCACAATA GATGCACAAG AAGTTGAAAC CATTTGAACT 7210
Quality scores
AJ489607 consensus GCTAATATTA ACAACAAAAC TAACACATAC AAGCACAATA GATGCACAAG AAGTTGAAAC CATTTGAACT 7210
Quality scores
AJ489607 consensus GCTAATATTA ACAACAAAAC TAACACATAC AAGCACAATA GATGCACAAG AAGTTGAAAC CATTTGAACT 7210
Quality scores
AJ489607 consensus GCTAATATTA ACAACAAAAC TAACACATAC AAGCACAATA GATGCACAAG AAGTTGAAAC CATTTGAACT 7210
Quality scores
AJ489607 consensus GCTAATATTA ACAACAAAAC TAACACATAC AAGCACAATA GATGCACAAG AAGTTGAAAC CATTTGAACT 7210
Quality scores
Consensus GCTAATATTA ACAACAAAAC TAACACATAC AAGCACAATA GATGCACAAG AAGTTGAAAC CATTTGAACT
Conservation
Sequence logo
7,220
7,240
7,260
7,280
AJ489607 consensus ATTCTACCAG CTGTAATCCT TATCATAATT GCTCTCCCCT CTCTACGCAT TCTATATATA ATAGACGAAA 7280
Quality scores
AJ489607 consensus ATTCTACCAG CTGTAATCCT TATCATAATT GCTCTCCCCT CTCTACGCAT TCTATATATA ATAGACGAAA 7280
Quality scores
AJ489607 consensus ATTCTACCAG CTGTAATCCT TATCATAATT GCTCTCCCCT CTCTACGCAT TCTATATATA ATAGACGAAA 7280
Quality scores
AJ489607 consensus ATTCTACCAG CTGTAATCCT TATCATAATT GCTCTCCCCT CTCTACGCAT TCTATATATA ATAGACGAAA 7280
Quality scores
AJ489607 consensus ATTCTACCAG CTGTAATCCT TATCATAATT GCTCTCCCCT CTCTACGCAT TCTATATATA ATAGACGAAA 7280
Quality scores
AJ489607 consensus ATTCTACCAG CTGTAATCCT TATCATAATT GCTCTCCCCT CTCTACGCAT TCTATATATA ATAGACGAAA 7280
Quality scores
AJ489607 consensus ATTCTACCAG CTGTAATCCT TATCATAATT GCTCTCCCCT CTCTACGCAT TCTATATATA ATAGACGAAA 7280
Quality scores
AJ489607 consensus ATTCTACCAG CTGTAATCCT TATCATAATT GCTCTCCCCT CTCTACGCAT TCTATATATA ATAGACGAAA 7280
Quality scores
AJ489607 consensus ATTCTACCAG CTGTAATCCT TATCATAATT GCTCTCCCCT CTCTACGCAT TCTATATATA ATAGACGAAA 7280
Quality scores
Consensus ATTCTACCAG CTGTAATCCT TATCATAATT GCTCTCCCCT CTCTACGCAT TCTATATATA ATAGACGAAA
Conservation
Sequence logo

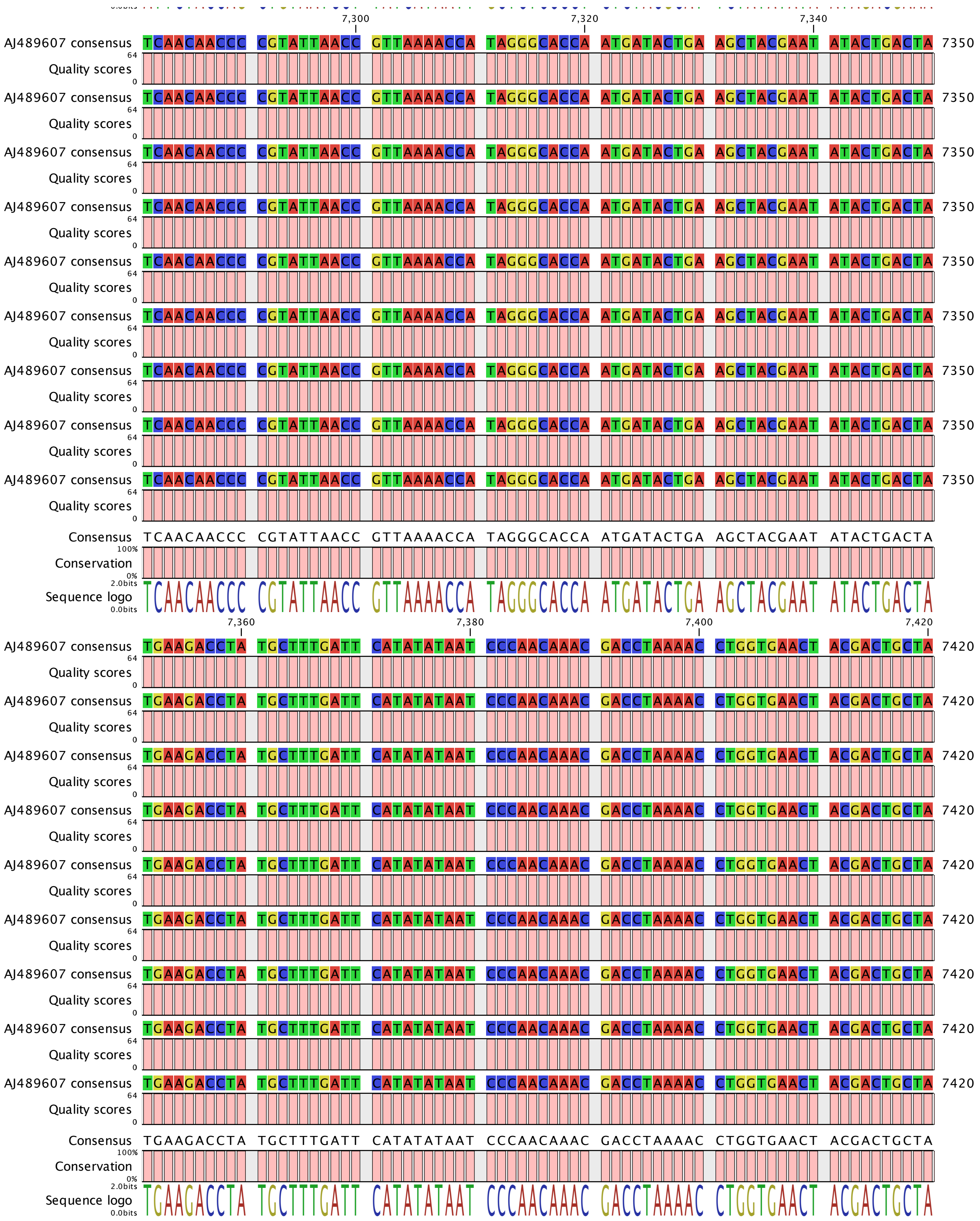
7,300
7,320
7,340
AJ489607 consensus TCAACAACCC CGTATTAACC GTTAAAACCA TAGGGCACCA ATGATACTGA AGCTACGAAT ATACTGACTA 7350
Quality scores
AJ489607 consensus TCAACAACCC CGTATTAACC GTTAAAACCA TAGGGCACCA ATGATACTGA AGCTACGAAT ATACTGACTA 7350
Quality scores
AJ489607 consensus TCAACAACCC CGTATTAACC GTTAAAACCA TAGGGCACCA ATGATACTGA AGCTACGAAT ATACTGACTA 7350
Quality scores
AJ489607 consensus TCAACAACCC CGTATTAACC GTTAAAACCA TAGGGCACCA ATGATACTGA AGCTACGAAT ATACTGACTA 7350
Quality scores
AJ489607 consensus TCAACAACCC CGTATTAACC GTTAAAACCA TAGGGCACCA ATGATACTGA AGCTACGAAT ATACTGACTA 7350
Quality scores
AJ489607 consensus TCAACAACCC CGTATTAACC GTTAAAACCA TAGGGCACCA ATGATACTGA AGCTACGAAT ATACTGACTA 7350
Quality scores
AJ489607 consensus TCAACAACCC CGTATTAACC GTTAAAACCA TAGGGCACCA ATGATACTGA AGCTACGAAT ATACTGACTA 7350
Quality scores
AJ489607 consensus TCAACAACCC CGTATTAACC GTTAAAACCA TAGGGCACCA ATGATACTGA AGCTACGAAT ATACTGACTA 7350
Quality scores
AJ489607 consensus TCAACAACCC CGTATTAACC GTTAAAACCA TAGGGCACCA ATGATACTGA AGCTACGAAT ATACTGACTA 7350
Quality scores
Consensus TCAACAACCC CGTATTAACC GTTAAAACCA TAGGGCACCA ATGATACTGA AGCTACGAAT ATACTGACTA
Conservation
Sequence logo
7,360
7,380
7,400
7,420
AJ489607 consensus TGAAGACCTA TGCTTTGATT CATATATAAT CCCAACAAAC GACCTAAAAC CTGGTGAACT ACGACTGCTA 7420
Quality scores
AJ489607 consensus TGAAGACCTA TGCTTTGATT CATATATAAT CCCAACAAAC GACCTAAAAC CTGGTGAACT ACGACTGCTA 7420
Quality scores
AJ489607 consensus TGAAGACCTA TGCTTTGATT CATATATAAT CCCAACAAAC GACCTAAAAC CTGGTGAACT ACGACTGCTA 7420
Quality scores
AJ489607 consensus TGAAGACCTA TGCTTTGATT CATATATAAT CCCAACAAAC GACCTAAAAC CTGGTGAACT ACGACTGCTA 7420
Quality scores
AJ489607 consensus TGAAGACCTA TGCTTTGATT CATATATAAT CCCAACAAAC GACCTAAAAC CTGGTGAACT ACGACTGCTA 7420
Quality scores
AJ489607 consensus TGAAGACCTA TGCTTTGATT CATATATAAT CCCAACAAAC GACCTAAAAC CTGGTGAACT ACGACTGCTA 7420
Quality scores
AJ489607 consensus TGAAGACCTA TGCTTTGATT CATATATAAT CCCAACAAAC GACCTAAAAC CTGGTGAACT ACGACTGCTA 7420
Quality scores
AJ489607 consensus TGAAGACCTA TGCTTTGATT CATATATAAT CCCAACAAAC GACCTAAAAC CTGGTGAACT ACGACTGCTA 7420
Quality scores
AJ489607 consensus TGAAGACCTA TGCTTTGATT CATATATAAT CCCAACAAAC GACCTAAAAC CTGGTGAACT ACGACTGCTA 7420
Quality scores
Consensus TGAAGACCTA TGCTTTGATT CATATATAAT CCCAACAAAC GACCTAAAAC CTGGTGAACT ACGACTGCTA
Conservation
Sequence logo

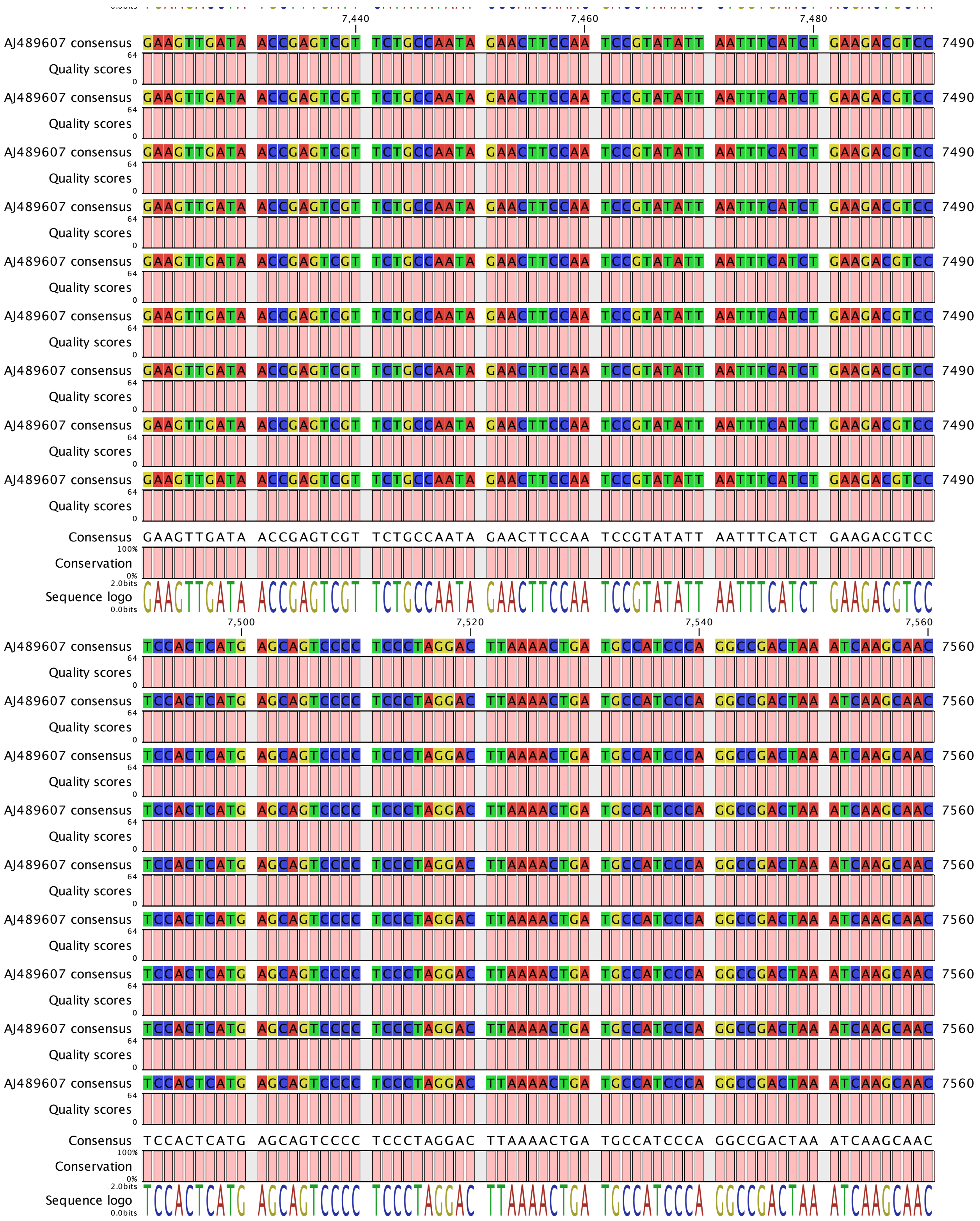

7,440
7,460
7,480
AJ489607 consensus GAAGTTGATA ACCGAGTCGT TCTGCCAATA GAACTTCCAA TCCGTATATT AATTTCATCT GAAGACGTCC 7490
Quality scores
AJ489607 consensus GAAGTTGATA ACCGAGTCGT TCTGCCAATA GAACTTCCAA TCCGTATATT AATTTCATCT GAAGACGTCC 7490
Quality scores
AJ489607 consensus GAAGTTGATA ACCGAGTCGT TCTGCCAATA GAACTTCCAA TCCGTATATT AATTTCATCT GAAGACGTCC 7490
Quality scores
AJ489607 consensus GAAGTTGATA ACCGAGTCGT TCTGCCAATA GAACTTCCAA TCCGTATATT AATTTCATCT GAAGACGTCC 7490
Quality scores
AJ489607 consensus GAAGTTGATA ACCGAGTCGT TCTGCCAATA GAACTTCCAA TCCGTATATT AATTTCATCT GAAGACGTCC 7490
Quality scores
AJ489607 consensus GAAGTTGATA ACCGAGTCGT TCTGCCAATA GAACTTCCAA TCCGTATATT AATTTCATCT GAAGACGTCC 7490
Quality scores
AJ489607 consensus GAAGTTGATA ACCGAGTCGT TCTGCCAATA GAACTTCCAA TCCGTATATT AATTTCATCT GAAGACGTCC 7490
Quality scores
AJ489607 consensus GAAGTTGATA ACCGAGTCGT TCTGCCAATA GAACTTCCAA TCCGTATATT AATTTCATCT GAAGACGTCC 7490
Quality scores
AJ489607 consensus GAAGTTGATA ACCGAGTCGT TCTGCCAATA GAACTTCCAA TCCGTATATT AATTTCATCT GAAGACGTCC 7490
Quality scores
Consensus GAAGTTGATA ACCGAGTCGT TCTGCCAATA GAACTTCCAA TCCGTATATT AATTTCATCT GAAGACGTCC
Conservation
Sequence logo
7,500
7,520
7,540
7,560
AJ489607 consensus TCCACTCATG AGCAGTCCCC TCCCTAGGAC TTAAAACTGA TGCCATCCCA GGCCGACTAA ATCAAGCAAC 7560
Quality scores
AJ489607 consensus TCCACTCATG AGCAGTCCCC TCCCTAGGAC TTAAAACTGA TGCCATCCCA GGCCGACTAA ATCAAGCAAC 7560
Quality scores
AJ489607 consensus TCCACTCATG AGCAGTCCCC TCCCTAGGAC TTAAAACTGA TGCCATCCCA GGCCGACTAA ATCAAGCAAC 7560
Quality scores
AJ489607 consensus TCCACTCATG AGCAGTCCCC TCCCTAGGAC TTAAAACTGA TGCCATCCCA GGCCGACTAA ATCAAGCAAC 7560
Quality scores
AJ489607 consensus TCCACTCATG AGCAGTCCCC TCCCTAGGAC TTAAAACTGA TGCCATCCCA GGCCGACTAA ATCAAGCAAC 7560
Quality scores
AJ489607 consensus TCCACTCATG AGCAGTCCCC TCCCTAGGAC TTAAAACTGA TGCCATCCCA GGCCGACTAA ATCAAGCAAC 7560
Quality scores
AJ489607 consensus TCCACTCATG AGCAGTCCCC TCCCTAGGAC TTAAAACTGA TGCCATCCCA GGCCGACTAA ATCAAGCAAC 7560
Quality scores
AJ489607 consensus TCCACTCATG AGCAGTCCCC TCCCTAGGAC TTAAAACTGA TGCCATCCCA GGCCGACTAA ATCAAGCAAC 7560
Quality scores
AJ489607 consensus TCCACTCATG AGCAGTCCCC TCCCTAGGAC TTAAAACTGA TGCCATCCCA GGCCGACTAA ATCAAGCAAC 7560
Quality scores
Consensus TCCACTCATG AGCAGTCCCC TCCCTAGGAC TTAAAACTGA TGCCATCCCA GGCCGACTAA ATCAAGCAAC
Conservation
Sequence logo

```
0.0bits
                              7,580                   7,600                   7,620
AJ489607 consensus  AGTAACATCA AACCGACCAG GGTTATTCTA TGGCCAATGC TCTGAAATTT GTGGATCTAA CCATAGCTTT  7630
64 Quality scores 0
AJ489607 consensus  AGTAACATCA AACCGACCAG GGTTATTCTA TGGCCAATGC TCTGAAATTT GTGGATCTAA CCATAGCTTT  7630
64 Quality scores 0
AJ489607 consensus  AGTAACATCA AACCGACCAG GGTTATTCTA TGGCCAATGC TCTGAAATTT GTGGATCTAA CCATAGCTTT  7630
64 Quality scores 0
AJ489607 consensus  AGTAACATCA AACCGACCAG GGTTATTCTA TGGCCAATGC TCTGAAATTT GTGGATCTAA CCATAGCTTT  7630
64 Quality scores 0
AJ489607 consensus  AGTAACATCA AACCGACCAG GGTTATTCTA TGGCCAATGC TCTGAAATTT GTGGATCTAA CCATAGCTTT  7630
64 Quality scores 0
AJ489607 consensus  AGTAACATCA AACCGACCAG GGTTATTCTA TGGCCAATGC TCTGAAATTT GTGGATCTAA CCATAGCTTT  7630
64 Quality scores 0
AJ489607 consensus  AGTAACATCA AACCGACCAG GGTTATTCTA TGGCCAATGC TCTGAAATTT GTGGATCTAA CCATAGCTTT  7630
64 Quality scores 0
AJ489607 consensus  AGTAACATCA AACCGACCAG GGTTATTCTA TGGCCAATGC TCTGAAATTT GTGGATCTAA CCATAGCTTT  7630
64 Quality scores 0
AJ489607 consensus  AGTAACATCA AACCGACCAG GGTTATTCTA TGGCCAATGC TCTGAAATTT GTGGATCTAA CCATAGCTTT  7630
64 Quality scores 0
Consensus           AGTAACATCA AACCGACCAG GGTTATTCTA TGGCCAATGC TCTGAAATTT GTGGATCTAA CCATAGCTTT
100% Conservation 0%
2.0bits Sequence logo 0.0bits  AGTAACATCA AACCGACCAG GGTTATTCTA TGGCCAATGC TCTGAAATTT GTGGATCTAA CCATAGCTTT

                 7,640                   7,660                   7,680                   7,700
AJ489607 consensus  ATGCCCATTG TCCTAGAAAT GGTTCCACTA AAATATTTCG AAAACTGATC TGCTTCAATA ATTTAATTTC  7700
64 Quality scores 0
AJ489607 consensus  ATGCCCATTG TCCTAGAAAT GGTTCCACTA AAATATTTCG AAAACTGATC TGCTTCAATA ATTTAATTTC  7700
64 Quality scores 0
AJ489607 consensus  ATGCCCATTG TCCTAGAAAT GGTTCCACTA AAATATTTCG AAAACTGATC TGCTTCAATA ATTTAATTTC  7700
64 Quality scores 0
AJ489607 consensus  ATGCCCATTG TCCTAGAAAT GGTTCCACTA AAATATTTCG AAAACTGATC TGCTTCAATA ATTTAATTTC  7700
64 Quality scores 0
AJ489607 consensus  ATGCCCATTG TCCTAGAAAT GGTTCCACTA AAATATTTCG AAAACTGATC TGCTTCAATA ATTTAATTTC  7700
64 Quality scores 0
AJ489607 consensus  ATGCCCATTG TCCTAGAAAT GGTTCCACTA AAATATTTCG AAAACTGATC TGCTTCAATA ATTTAATTTC  7700
64 Quality scores 0
AJ489607 consensus  ATGCCCATTG TCCTAGAAAT GGTTCCACTA AAATATTTCG AAAACTGATC TGCTTCAATA ATTTAATTTC  7700
64 Quality scores 0
AJ489607 consensus  ATGCCCATTG TCCTAGAAAT GGTTCCACTA AAATATTTCG AAAACTGATC TGCTTCAATA ATTTAATTTC  7700
64 Quality scores 0
AJ489607 consensus  ATGCCCATTG TCCTAGAAAT GGTTCCACTA AAATATTTCG AAAACTGATC TGCTTCAATA ATTTAATTTC  7700
64 Quality scores 0
Consensus           ATGCCCATTG TCCTAGAAAT GGTTCCACTA AAATATTTCG AAAACTGATC TGCTTCAATA ATTTAATTTC
100% Conservation 0%
2.0bits Sequence logo 0.0bits  ATGCCCATTG TCCTAGAAAT GGTTCCACTA AAATATTTCG AAAACTGATC TGCTTCAATA ATTTAATTTC
```

0.0bits

7,720 7,740 7,760

AJ489607 consensus ACTATGAAGC TAAGAGCGTT AACCTTTTAA GTTAAAGTTA GAGACCTTAA AATCTCCATA GTGATATGCC 7770
64 Quality scores 0
AJ489607 consensus ACTATGAAGC TAAGAGCGTT AACCTTTTAA GTTAAAGTTA GAGACCTTAA AATCTCCATA GTGATATGCC 7770
64 Quality scores 0
AJ489607 consensus ACTATGAAGC TAAGAGCGTT AACCTTTTAA GTTAAAGTTA GAGACCTTAA AATCTCCATA GTGATATGCC 7770
64 Quality scores 0
AJ489607 consensus ACTATGAAGC TAAGAGCGTT AACCTTTTAA GTTAAAGTTA GAGACCTTAA AATCTCCATA GTGATATGCC 7770
64 Quality scores 0
AJ489607 consensus ACTATGAAGC TAAGAGCGTT AACCTTTTAA GTTAAAGTTA GAGACCTTAA AATCTCCATA GTGATATGCC 7770
64 Quality scores 0
AJ489607 consensus ACTATGAAGC TAAGAGCGTT AACCTTTTAA GTTAAAGTTA GAGACCTTAA AATCTCCATA GTGATATGCC 7770
64 Quality scores 0
AJ489607 consensus ACTATGAAGC TAAGAGCGTT AACCTTTTAA GTTAAAGTTA GAGACCTTAA AATCTCCATA GTGATATGCC 7770
64 Quality scores 0
AJ489607 consensus ACTATGAAGC TAAGAGCGTT AACCTTTTAA GTTAAAGTTA GAGACCTTAA AATCTCCATA GTGATATGCC 7770
64 Quality scores 0
AJ489607 consensus ACTATGAAGC TAAGAGCGTT AACCTTTTAA GTTAAAGTTA GAGACCTTAA AATCTCCATA GTGATATGCC 7770
64 Quality scores 0

Consensus ACTATGAAGC TAAGAGCGTT AACCTTTTAA GTTAAAGTTA GAGACCTTAA AATCTCCATA GTGATATGCC
100% Conservation 0%
2.0bits Sequence logo 0.0bits ACTATGAAGC TAAGAGCGTT AACCTTTTAA GTTAAAGTTA GAGACCTTAA AATCTCCATA GTGATATGCC

7,780 7,800 7,820 7,840

AJ489607 consensus ACAACTAGAT ACATCAACAT GATTTATCAC AATTATCTCA TCAATAATTA CCCTATTTAT CTTATTTCAA 7840
64 Quality scores 0
AJ489607 consensus ACAACTAGAT ACATCAACAT GATTTATCAC AATTATCTCA TCAATAATTA CCCTATTTAT CTTATTTCAA 7840
64 Quality scores 0
AJ489607 consensus ACAACTAGAT ACATCAACAT GATTTATCAC AATTATCTCA TCAATAATTA CCCTATTTAT CTTATTTCAA 7840
64 Quality scores 0
AJ489607 consensus ACAACTAGAT ACATCAACAT GATTTATCAC AATTATCTCA TCAATAATTA CCCTATTTAT CTTATTTCAA 7840
64 Quality scores 0
AJ489607 consensus ACAACTAGAT ACATCAACAT GATTTATCAC AATTATCTCA TCAATAATTA CCCTATTTAT CTTATTTCAA 7840
64 Quality scores 0
AJ489607 consensus ACAACTAGAT ACATCAACAT GATTTATCAC AATTATCTCA TCAATAATTA CCCTATTTAT CTTATTTCAA 7840
64 Quality scores 0
AJ489607 consensus ACAACTAGAT ACATCAACAT GATTTATCAC AATTATCTCA TCAATAATTA CCCTATTTAT CTTATTTCAA 7840
64 Quality scores 0
AJ489607 consensus ACAACTAGAT ACATCAACAT GATTTATCAC AATTATCTCA TCAATAATTA CCCTATTTAT CTTATTTCAA 7840
64 Quality scores 0
AJ489607 consensus ACAACTAGAT ACATCAACAT GATTTATCAC AATTATCTCA TCAATAATTA CCCTATTTAT CTTATTTCAA 7840
64 Quality scores 0

Consensus ACAACTAGAT ACATCAACAT GATTTATCAC AATTATCTCA TCAATAATTA CCCTATTTAT CTTATTTCAA
100% Conservation 0%
2.0bits Sequence logo 0.0bits ACAACTAGAT ACATCAACAT GATTTATCAC AATTATCTCA TCAATAATTA CCCTATTTAT CTTATTTCAA

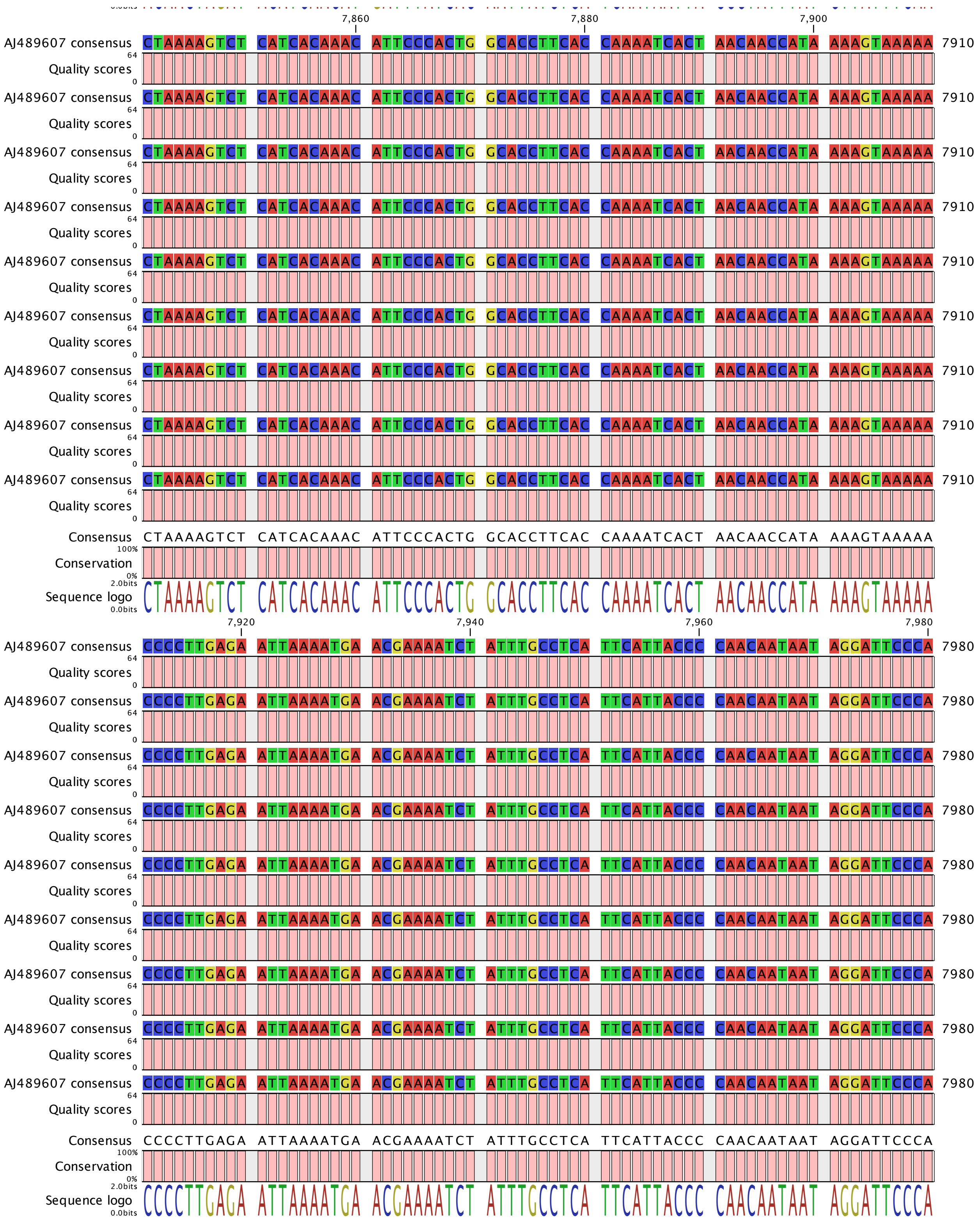
7,860 7,880 7,900
AJ489607 consensus CTAAAAGTCT CATCACAAAC ATTCCCACTG GCACCTTCAC CAAAATCACT AACAACCATA AAAGTAAAAA 7910
Quality scores
AJ489607 consensus CTAAAAGTCT CATCACAAAC ATTCCCACTG GCACCTTCAC CAAAATCACT AACAACCATA AAAGTAAAAA 7910
Quality scores
AJ489607 consensus CTAAAAGTCT CATCACAAAC ATTCCCACTG GCACCTTCAC CAAAATCACT AACAACCATA AAAGTAAAAA 7910
Quality scores
AJ489607 consensus CTAAAAGTCT CATCACAAAC ATTCCCACTG GCACCTTCAC CAAAATCACT AACAACCATA AAAGTAAAAA 7910
Quality scores
AJ489607 consensus CTAAAAGTCT CATCACAAAC ATTCCCACTG GCACCTTCAC CAAAATCACT AACAACCATA AAAGTAAAAA 7910
Quality scores
AJ489607 consensus CTAAAAGTCT CATCACAAAC ATTCCCACTG GCACCTTCAC CAAAATCACT AACAACCATA AAAGTAAAAA 7910
Quality scores
AJ489607 consensus CTAAAAGTCT CATCACAAAC ATTCCCACTG GCACCTTCAC CAAAATCACT AACAACCATA AAAGTAAAAA 7910
Quality scores
AJ489607 consensus CTAAAAGTCT CATCACAAAC ATTCCCACTG GCACCTTCAC CAAAATCACT AACAACCATA AAAGTAAAAA 7910
Quality scores
AJ489607 consensus CTAAAAGTCT CATCACAAAC ATTCCCACTG GCACCTTCAC CAAAATCACT AACAACCATA AAAGTAAAAA 7910
Quality scores
Consensus CTAAAAGTCT CATCACAAAC ATTCCCACTG GCACCTTCAC CAAAATCACT AACAACCATA AAAGTAAAAA
Conservation
Sequence logo
7,920 7,940 7,960 7,980
AJ489607 consensus CCCCTTGAGA ATTAAAATGA ACGAAAATCT ATTTGCCTCA TTCATTACCC CAACAATAAT AGGATTCCCA 7980
Quality scores
AJ489607 consensus CCCCTTGAGA ATTAAAATGA ACGAAAATCT ATTTGCCTCA TTCATTACCC CAACAATAAT AGGATTCCCA 7980
Quality scores
AJ489607 consensus CCCCTTGAGA ATTAAAATGA ACGAAAATCT ATTTGCCTCA TTCATTACCC CAACAATAAT AGGATTCCCA 7980
Quality scores
AJ489607 consensus CCCCTTGAGA ATTAAAATGA ACGAAAATCT ATTTGCCTCA TTCATTACCC CAACAATAAT AGGATTCCCA 7980
Quality scores
AJ489607 consensus CCCCTTGAGA ATTAAAATGA ACGAAAATCT ATTTGCCTCA TTCATTACCC CAACAATAAT AGGATTCCCA 7980
Quality scores
AJ489607 consensus CCCCTTGAGA ATTAAAATGA ACGAAAATCT ATTTGCCTCA TTCATTACCC CAACAATAAT AGGATTCCCA 7980
Quality scores
AJ489607 consensus CCCCTTGAGA ATTAAAATGA ACGAAAATCT ATTTGCCTCA TTCATTACCC CAACAATAAT AGGATTCCCA 7980
Quality scores
AJ489607 consensus CCCCTTGAGA ATTAAAATGA ACGAAAATCT ATTTGCCTCA TTCATTACCC CAACAATAAT AGGATTCCCA 7980
Quality scores
AJ489607 consensus CCCCTTGAGA ATTAAAATGA ACGAAAATCT ATTTGCCTCA TTCATTACCC CAACAATAAT AGGATTCCCA 7980
Quality scores
Consensus CCCCTTGAGA ATTAAAATGA ACGAAAATCT ATTTGCCTCA TTCATTACCC CAACAATAAT AGGATTCCCA
Conservation
Sequence logo

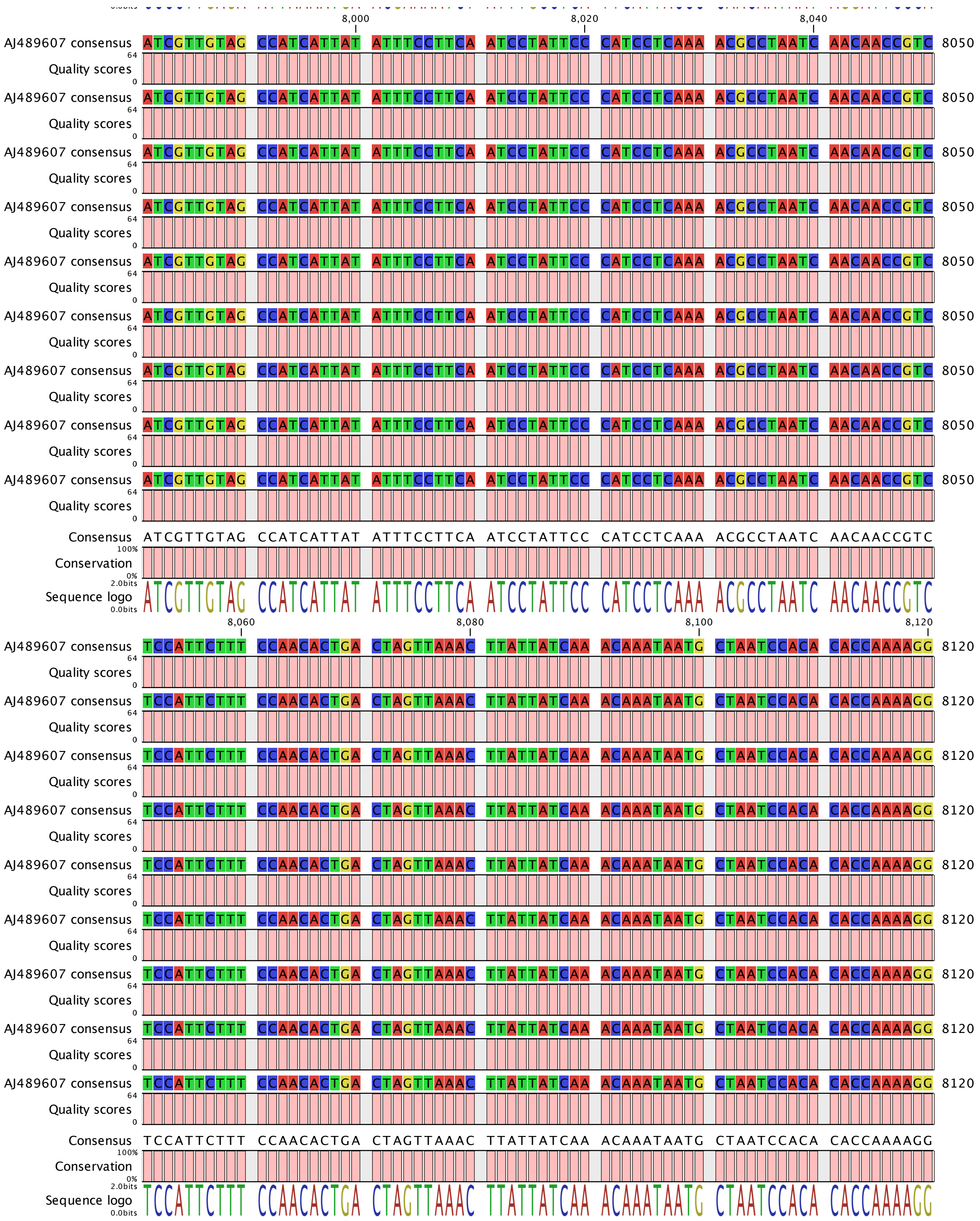
8,000
8,020
8,040
AJ489607 consensus ATCGTTGTAG CCATCATTAT ATTTCCTTCA ATCCTATTCC CATCCTCAAA ACGCCTAATC AACAACCGTC 8050
Quality scores
AJ489607 consensus ATCGTTGTAG CCATCATTAT ATTTCCTTCA ATCCTATTCC CATCCTCAAA ACGCCTAATC AACAACCGTC 8050
Quality scores
AJ489607 consensus ATCGTTGTAG CCATCATTAT ATTTCCTTCA ATCCTATTCC CATCCTCAAA ACGCCTAATC AACAACCGTC 8050
Quality scores
AJ489607 consensus ATCGTTGTAG CCATCATTAT ATTTCCTTCA ATCCTATTCC CATCCTCAAA ACGCCTAATC AACAACCGTC 8050
Quality scores
AJ489607 consensus ATCGTTGTAG CCATCATTAT ATTTCCTTCA ATCCTATTCC CATCCTCAAA ACGCCTAATC AACAACCGTC 8050
Quality scores
AJ489607 consensus ATCGTTGTAG CCATCATTAT ATTTCCTTCA ATCCTATTCC CATCCTCAAA ACGCCTAATC AACAACCGTC 8050
Quality scores
AJ489607 consensus ATCGTTGTAG CCATCATTAT ATTTCCTTCA ATCCTATTCC CATCCTCAAA ACGCCTAATC AACAACCGTC 8050
Quality scores
AJ489607 consensus ATCGTTGTAG CCATCATTAT ATTTCCTTCA ATCCTATTCC CATCCTCAAA ACGCCTAATC AACAACCGTC 8050
Quality scores
AJ489607 consensus ATCGTTGTAG CCATCATTAT ATTTCCTTCA ATCCTATTCC CATCCTCAAA ACGCCTAATC AACAACCGTC 8050
Quality scores
Consensus ATCGTTGTAG CCATCATTAT ATTTCCTTCA ATCCTATTCC CATCCTCAAA ACGCCTAATC AACAACCGTC
Conservation
Sequence logo
8,060
8,080
8,100
8,120
AJ489607 consensus TCCATTCTTT CCAACACTGA CTAGTTAAAC TTATTATCAA ACAAATAATG CTAATCCACA CACCAAAAGG 8120
Quality scores
AJ489607 consensus TCCATTCTTT CCAACACTGA CTAGTTAAAC TTATTATCAA ACAAATAATG CTAATCCACA CACCAAAAGG 8120
Quality scores
AJ489607 consensus TCCATTCTTT CCAACACTGA CTAGTTAAAC TTATTATCAA ACAAATAATG CTAATCCACA CACCAAAAGG 8120
Quality scores
AJ489607 consensus TCCATTCTTT CCAACACTGA CTAGTTAAAC TTATTATCAA ACAAATAATG CTAATCCACA CACCAAAAGG 8120
Quality scores
AJ489607 consensus TCCATTCTTT CCAACACTGA CTAGTTAAAC TTATTATCAA ACAAATAATG CTAATCCACA CACCAAAAGG 8120
Quality scores
AJ489607 consensus TCCATTCTTT CCAACACTGA CTAGTTAAAC TTATTATCAA ACAAATAATG CTAATCCACA CACCAAAAGG 8120
Quality scores
AJ489607 consensus TCCATTCTTT CCAACACTGA CTAGTTAAAC TTATTATCAA ACAAATAATG CTAATCCACA CACCAAAAGG 8120
Quality scores
AJ489607 consensus TCCATTCTTT CCAACACTGA CTAGTTAAAC TTATTATCAA ACAAATAATG CTAATCCACA CACCAAAAGG 8120
Quality scores
AJ489607 consensus TCCATTCTTT CCAACACTGA CTAGTTAAAC TTATTATCAA ACAAATAATG CTAATCCACA CACCAAAAGG 8120
Quality scores
Consensus TCCATTCTTT CCAACACTGA CTAGTTAAAC TTATTATCAA ACAAATAATG CTAATCCACA CACCAAAAGG
Conservation
Sequence logo

8,140 8,160 8,180

AJ489607 consensus ACGAACATGA ACCCTAATAA TTGTTTCCCT AATCATATTT ATTGGATCAA CAAATCTCCT AGGCCTTTTA 8190
64 Quality scores 0
AJ489607 consensus ACGAACATGA ACCCTAATAA TTGTTTCCCT AATCATATTT ATTGGATCAA CAAATCTCCT AGGCCTTTTA 8190
64 Quality scores 0
AJ489607 consensus ACGAACATGA ACCCTAATAA TTGTTTCCCT AATCATATTT ATTGGATCAA CAAATCTCCT AGGCCTTTTA 8190
64 Quality scores 0
AJ489607 consensus ACGAACATGA ACCCTAATAA TTGTTTCCCT AATCATATTT ATTGGATCAA CAAATCTCCT AGGCCTTTTA 8190
64 Quality scores 0
AJ489607 consensus ACGAACATGA ACCCTAATAA TTGTTTCCCT AATCATATTT ATTGGATCAA CAAATCTCCT AGGCCTTTTA 8190
64 Quality scores 0
AJ489607 consensus ACGAACATGA ACCCTAATAA TTGTTTCCCT AATCATATTT ATTGGATCAA CAAATCTCCT AGGCCTTTTA 8190
64 Quality scores 0
AJ489607 consensus ACGAACATGA ACCCTAATAA TTGTTTCCCT AATCATATTT ATTGGATCAA CAAATCTCCT AGGCCTTTTA 8190
64 Quality scores 0
AJ489607 consensus ACGAACATGA ACCCTAATAA TTGTTTCCCT AATCATATTT ATTGGATCAA CAAATCTCCT AGGCCTTTTA 8190
64 Quality scores 0
AJ489607 consensus ACGAACATGA ACCCTAATAA TTGTTTCCCT AATCATATTT ATTGGATCAA CAAATCTCCT AGGCCTTTTA 8190
64 Quality scores 0
Consensus ACGAACATGA ACCCTAATAA TTGTTTCCCT AATCATATTT ATTGGATCAA CAAATCTCCT AGGCCTTTTA
100% Conservation 0%
2.0bits Sequence logo 0.0bits ACGAACATGA ACCCTAATAA TTGTTTCCCT AATCATATTT ATTGGATCAA CAAATCTCCT AGGCCTTTTA

8,200 8,220 8,240 8,260

AJ489607 consensus CCACATACAT TTACACCTAC TACCCAACTA TCCATAAATC TAAGTATAGC CATTCCACTA TGAGCTGGAG 8260
64 Quality scores 0
AJ489607 consensus CCACATACAT TTACACCTAC TACCCAACTA TCCATAAATC TAAGTATAGC CATTCCACTA TGAGCTGGAG 8260
64 Quality scores 0
AJ489607 consensus CCACATACAT TTACACCTAC TACCCAACTA TCCATAAATC TAAGTATAGC CATTCCACTA TGAGCTGGAG 8260
64 Quality scores 0
AJ489607 consensus CCACATACAT TTACACCTAC TACCCAACTA TCCATAAATC TAAGTATAGC CATTCCACTA TGAGCTGGAG 8260
64 Quality scores 0
AJ489607 consensus CCACATACAT TTACACCTAC TACCCAACTA TCCATAAATC TAAGTATAGC CATTCCACTA TGAGCTGGAG 8260
64 Quality scores 0
AJ489607 consensus CCACATACAT TTACACCTAC TACCCAACTA TCCATAAATC TAAGTATAGC CATTCCACTA TGAGCTGGAG 8260
64 Quality scores 0
AJ489607 consensus CCACATACAT TTACACCTAC TACCCAACTA TCCATAAATC TAAGTATAGC CATTCCACTA TGAGCTGGAG 8260
64 Quality scores 0
AJ489607 consensus CCACATACAT TTACACCTAC TACCCAACTA TCCATAAATC TAAGTATAGC CATTCCACTA TGAGCTGGAG 8260
64 Quality scores 0
AJ489607 consensus CCACATACAT TTACACCTAC TACCCAACTA TCCATAAATC TAAGTATAGC CATTCCACTA TGAGCTGGAG 8260
64 Quality scores 0
Consensus CCACATACAT TTACACCTAC TACCCAACTA TCCATAAATC TAAGTATAGC CATTCCACTA TGAGCTGGAG
100% Conservation 0%
2.0bits Sequence logo 0.0bits CCACATACAT TTACACCTAC TACCCAACTA TCCATAAATC TAAGTATAGC CATTCCACTA TGAGCTGGAG

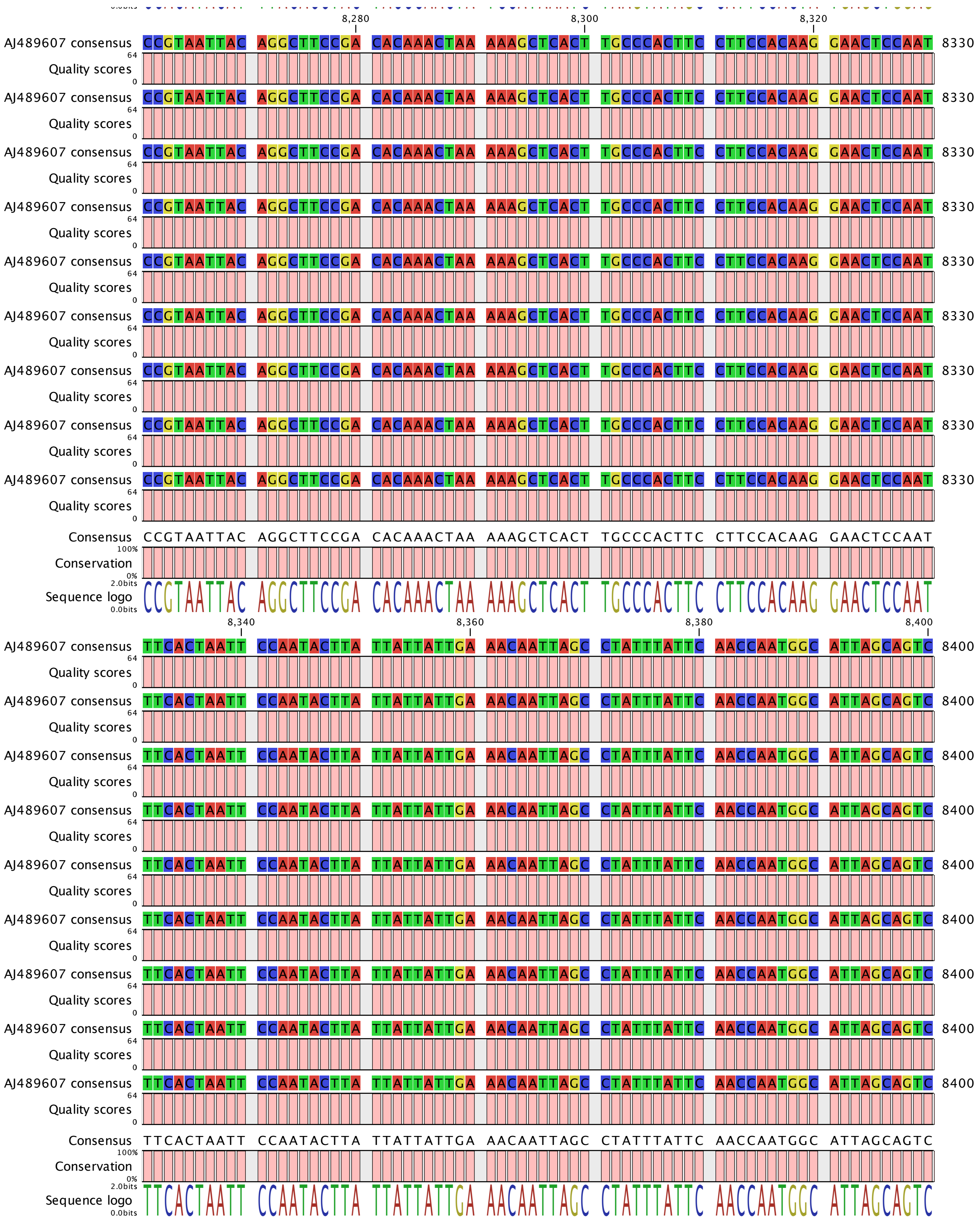
8,280
8,300
8,320
AJ489607 consensus CCGTAATTAC AGGCTTCCGA CACAAACTAA AAAGCTCACT TGCCCACTTC CTTCCACAAG GAACTCCAAT 8330
Quality scores
AJ489607 consensus CCGTAATTAC AGGCTTCCGA CACAAACTAA AAAGCTCACT TGCCCACTTC CTTCCACAAG GAACTCCAAT 8330
Quality scores
AJ489607 consensus CCGTAATTAC AGGCTTCCGA CACAAACTAA AAAGCTCACT TGCCCACTTC CTTCCACAAG GAACTCCAAT 8330
Quality scores
AJ489607 consensus CCGTAATTAC AGGCTTCCGA CACAAACTAA AAAGCTCACT TGCCCACTTC CTTCCACAAG GAACTCCAAT 8330
Quality scores
AJ489607 consensus CCGTAATTAC AGGCTTCCGA CACAAACTAA AAAGCTCACT TGCCCACTTC CTTCCACAAG GAACTCCAAT 8330
Quality scores
AJ489607 consensus CCGTAATTAC AGGCTTCCGA CACAAACTAA AAAGCTCACT TGCCCACTTC CTTCCACAAG GAACTCCAAT 8330
Quality scores
AJ489607 consensus CCGTAATTAC AGGCTTCCGA CACAAACTAA AAAGCTCACT TGCCCACTTC CTTCCACAAG GAACTCCAAT 8330
Quality scores
AJ489607 consensus CCGTAATTAC AGGCTTCCGA CACAAACTAA AAAGCTCACT TGCCCACTTC CTTCCACAAG GAACTCCAAT 8330
Quality scores
AJ489607 consensus CCGTAATTAC AGGCTTCCGA CACAAACTAA AAAGCTCACT TGCCCACTTC CTTCCACAAG GAACTCCAAT 8330
Quality scores
Consensus CCGTAATTAC AGGCTTCCGA CACAAACTAA AAAGCTCACT TGCCCACTTC CTTCCACAAG GAACTCCAAT
Conservation
Sequence logo
8,340
8,360
8,380
8,400
AJ489607 consensus TTCACTAATT CCAATACTTA TTATTATTGA AACAATTAGC CTATTTATTC AACCAATGGC ATTAGCAGTC 8400
Quality scores
AJ489607 consensus TTCACTAATT CCAATACTTA TTATTATTGA AACAATTAGC CTATTTATTC AACCAATGGC ATTAGCAGTC 8400
Quality scores
AJ489607 consensus TTCACTAATT CCAATACTTA TTATTATTGA AACAATTAGC CTATTTATTC AACCAATGGC ATTAGCAGTC 8400
Quality scores
AJ489607 consensus TTCACTAATT CCAATACTTA TTATTATTGA AACAATTAGC CTATTTATTC AACCAATGGC ATTAGCAGTC 8400
Quality scores
AJ489607 consensus TTCACTAATT CCAATACTTA TTATTATTGA AACAATTAGC CTATTTATTC AACCAATGGC ATTAGCAGTC 8400
Quality scores
AJ489607 consensus TTCACTAATT CCAATACTTA TTATTATTGA AACAATTAGC CTATTTATTC AACCAATGGC ATTAGCAGTC 8400
Quality scores
AJ489607 consensus TTCACTAATT CCAATACTTA TTATTATTGA AACAATTAGC CTATTTATTC AACCAATGGC ATTAGCAGTC 8400
Quality scores
AJ489607 consensus TTCACTAATT CCAATACTTA TTATTATTGA AACAATTAGC CTATTTATTC AACCAATGGC ATTAGCAGTC 8400
Quality scores
AJ489607 consensus TTCACTAATT CCAATACTTA TTATTATTGA AACAATTAGC CTATTTATTC AACCAATGGC ATTAGCAGTC 8400
Quality scores
Consensus TTCACTAATT CCAATACTTA TTATTATTGA AACAATTAGC CTATTTATTC AACCAATGGC ATTAGCAGTC
Conservation
Sequence logo

0.0bits

8,420 8,440 8,460

AJ489607 consensus CGGCTTACAG CTAACATTAC TGCAGGACAC TTATTAATAC ACCTAATCGG AGGAGCTACT CTAGTATTAA 8470
64 Quality scores 0
AJ489607 consensus CGGCTTACAG CTAACATTAC TGCAGGACAC TTATTAATAC ACCTAATCGG AGGAGCTACT CTAGTATTAA 8470
64 Quality scores 0
AJ489607 consensus CGGCTTACAG CTAACATTAC TGCAGGACAC TTATTAATAC ACCTAATCGG AGGAGCTACT CTAGTATTAA 8470
64 Quality scores 0
AJ489607 consensus CGGCTTACAG CTAACATTAC TGCAGGACAC TTATTAATAC ACCTAATCGG AGGAGCTACT CTAGTATTAA 8470
64 Quality scores 0
AJ489607 consensus CGGCTTACAG CTAACATTAC TGCAGGACAC TTATTAATAC ACCTAATCGG AGGAGCTACT CTAGTATTAA 8470
64 Quality scores 0
AJ489607 consensus CGGCTTACAG CTAACATTAC TGCAGGACAC TTATTAATAC ACCTAATCGG AGGAGCTACT CTAGTATTAA 8470
64 Quality scores 0
AJ489607 consensus CGGCTTACAG CTAACATTAC TGCAGGACAC TTATTAATAC ACCTAATCGG AGGAGCTACT CTAGTATTAA 8470
64 Quality scores 0
AJ489607 consensus CGGCTTACAG CTAACATTAC TGCAGGACAC TTATTAATAC ACCTAATCGG AGGAGCTACT CTAGTATTAA 8470
64 Quality scores 0
AJ489607 consensus CGGCTTACAG CTAACATTAC TGCAGGACAC TTATTAATAC ACCTAATCGG AGGAGCTACT CTAGTATTAA 8470
64 Quality scores 0
Consensus CGGCTTACAG CTAACATTAC TGCAGGACAC TTATTAATAC ACCTAATCGG AGGAGCTACT CTAGTATTAA
100% Conservation 0%
2.0bits Sequence logo 0.0bits CGGCTTACAG CTAACATTAC TGCAGGACAC TTATTAATAC ACCTAATCGG AGGAGCTACT CTAGTATTAA

8,480 8,500 8,520 8,540

AJ489607 consensus TAAATATTAG CCCACCAACA GCTACCATTA CATTTATTAT TTTACTTCTA CTCACAATTC TAGAATTTGC 8540
64 Quality scores 0
AJ489607 consensus TAAATATTAG CCCACCAACA GCTACCATTA CATTTATTAT TTTACTTCTA CTCACAATTC TAGAATTTGC 8540
64 Quality scores 0
AJ489607 consensus TAAATATTAG CCCACCAACA GCTACCATTA CATTTATTAT TTTACTTCTA CTCACAATTC TAGAATTTGC 8540
64 Quality scores 0
AJ489607 consensus TAAATATTAG CCCACCAACA GCTACCATTA CATTTATTAT TTTACTTCTA CTCACAATTC TAGAATTTGC 8540
64 Quality scores 0
AJ489607 consensus TAAATATTAG CCCACCAACA GCTACCATTA CATTTATTAT TTTACTTCTA CTCACAATTC TAGAATTTGC 8540
64 Quality scores 0
AJ489607 consensus TAAATATTAG CCCACCAACA GCTACCATTA CATTTATTAT TTTACTTCTA CTCACAATTC TAGAATTTGC 8540
64 Quality scores 0
AJ489607 consensus TAAATATTAG CCCACCAACA GCTACCATTA CATTTATTAT TTTACTTCTA CTCACAATTC TAGAATTTGC 8540
64 Quality scores 0
AJ489607 consensus TAAATATTAG CCCACCAACA GCTACCATTA CATTTATTAT TTTACTTCTA CTCACAATTC TAGAATTTGC 8540
64 Quality scores 0
AJ489607 consensus TAAATATTAG CCCACCAACA GCTACCATTA CATTTATTAT TTTACTTCTA CTCACAATTC TAGAATTTGC 8540
64 Quality scores 0
Consensus TAAATATTAG CCCACCAACA GCTACCATTA CATTTATTAT TTTACTTCTA CTCACAATTC TAGAATTTGC
100% Conservation 0%
2.0bits Sequence logo 0.0bits TAAATATTAG CCCACCAACA GCTACCATTA CATTTATTAT TTTACTTCTA CTCACAATTC TAGAATTTGC

0.0bits

8,560 8,580 8,600

AJ489607 consensus AGTAGCATTA ATTCAAGCCT ACGTATTCAC CCTCCTAGTA AGCCTATATC TACATGATAA TACATAATGA 8610
64
Quality scores
0
AJ489607 consensus AGTAGCATTA ATTCAAGCCT ACGTATTCAC CCTCCTAGTA AGCCTATATC TACATGATAA TACATAATGA 8610
64
Quality scores
0
AJ489607 consensus AGTAGCATTA ATTCAAGCCT ACGTATTCAC CCTCCTAGTA AGCCTATATC TACATGATAA TACATAATGA 8610
64
Quality scores
0
AJ489607 consensus AGTAGCATTA ATTCAAGCCT ACGTATTCAC CCTCCTAGTA AGCCTATATC TACATGATAA TACATAATGA 8610
64
Quality scores
0
AJ489607 consensus AGTAGCATTA ATTCAAGCCT ACGTATTCAC CCTCCTAGTA AGCCTATATC TACATGATAA TACATAATGA 8610
64
Quality scores
0
AJ489607 consensus AGTAGCATTA ATTCAAGCCT ACGTATTCAC CCTCCTAGTA AGCCTATATC TACATGATAA TACATAATGA 8610
64
Quality scores
0
AJ489607 consensus AGTAGCATTA ATTCAAGCCT ACGTATTCAC CCTCCTAGTA AGCCTATATC TACATGATAA TACATAATGA 8610
64
Quality scores
0
AJ489607 consensus AGTAGCATTA ATTCAAGCCT ACGTATTCAC CCTCCTAGTA AGCCTATATC TACATGATAA TACATAATGA 8610
64
Quality scores
0
AJ489607 consensus AGTAGCATTA ATTCAAGCCT ACGTATTCAC CCTCCTAGTA AGCCTATATC TACATGATAA TACATAATGA 8610
64
Quality scores
0
Consensus AGTAGCATTA ATTCAAGCCT ACGTATTCAC CCTCCTAGTA AGCCTATATC TACATGATAA TACATAATGA
100%
Conservation
0%
2.0bits
Sequence logo AGTAGCATTA ATTCAAGCCT ACGTATTCAC CCTCCTAGTA AGCCTATATC TACATGATAA TACATAATGA
0.0bits

8,620 8,640 8,660 8,680

AJ489607 consensus CCCACCAAAC TCATGCATAT CACATAGTTA ATCCAAGTCC ATGACCATTA ACTGGAGCCT TTTCAGCCCT 8680
64
Quality scores
0
AJ489607 consensus CCCACCAAAC TCATGCATAT CACATAGTTA ATCCAAGTCC ATGACCATTA ACTGGAGCCT TTTCAGCCCT 8680
64
Quality scores
0
AJ489607 consensus CCCACCAAAC TCATGCATAT CACATAGTTA ATCCAAGTCC ATGACCATTA ACTGGAGCCT TTTCAGCCCT 8680
64
Quality scores
0
AJ489607 consensus CCCACCAAAC TCATGCATAT CACATAGTTA ATCCAAGTCC ATGACCATTA ACTGGAGCCT TTTCAGCCCT 8680
64
Quality scores
0
AJ489607 consensus CCCACCAAAC TCATGCATAT CACATAGTTA ATCCAAGTCC ATGACCATTA ACTGGAGCCT TTTCAGCCCT 8680
64
Quality scores
0
AJ489607 consensus CCCACCAAAC TCATGCATAT CACATAGTTA ATCCAAGTCC ATGACCATTA ACTGGAGCCT TTTCAGCCCT 8680
64
Quality scores
0
AJ489607 consensus CCCACCAAAC TCATGCATAT CACATAGTTA ATCCAAGTCC ATGACCATTA ACTGGAGCCT TTTCAGCCCT 8680
64
Quality scores
0
AJ489607 consensus CCCACCAAAC TCATGCATAT CACATAGTTA ATCCAAGTCC ATGACCATTA ACTGGAGCCT TTTCAGCCCT 8680
64
Quality scores
0
AJ489607 consensus CCCACCAAAC TCATGCATAT CACATAGTTA ATCCAAGTCC ATGACCATTA ACTGGAGCCT TTTCAGCCCT 8680
64
Quality scores
0
Consensus CCCACCAAAC TCATGCATAT CACATAGTTA ATCCAAGTCC ATGACCATTA ACTGGAGCCT TTTCAGCCCT
100%
Conservation
0%
2.0bits
Sequence logo CCCACCAAAC TCATGCATAT CACATAGTTA ATCCAAGTCC ATGACCATTA ACTGGAGCCT TTTCAGCCCT
0.0bits

0.0bits

8,700 8,720 8,740

AJ489607 consensus CCTTCTAACA TCAGGTCTAG TAATATGATT TCACTATAAT TCAATTACAC TATTAACCCT TGGCCTACTC 8750
64
Quality scores
0
AJ489607 consensus CCTTCTAACA TCAGGTCTAG TAATATGATT TCACTATAAT TCAATTACAC TATTAACCCT TGGCCTACTC 8750
64
Quality scores
0
AJ489607 consensus CCTTCTAACA TCAGGTCTAG TAATATGATT TCACTATAAT TCAATTACAC TATTAACCCT TGGCCTACTC 8750
64
Quality scores
0
AJ489607 consensus CCTTCTAACA TCAGGTCTAG TAATATGATT TCACTATAAT TCAATTACAC TATTAACCCT TGGCCTACTC 8750
64
Quality scores
0
AJ489607 consensus CCTTCTAACA TCAGGTCTAG TAATATGATT TCACTATAAT TCAATTACAC TATTAACCCT TGGCCTACTC 8750
64
Quality scores
0
AJ489607 consensus CCTTCTAACA TCAGGTCTAG TAATATGATT TCACTATAAT TCAATTACAC TATTAACCCT TGGCCTACTC 8750
64
Quality scores
0
AJ489607 consensus CCTTCTAACA TCAGGTCTAG TAATATGATT TCACTATAAT TCAATTACAC TATTAACCCT TGGCCTACTC 8750
64
Quality scores
0
AJ489607 consensus CCTTCTAACA TCAGGTCTAG TAATATGATT TCACTATAAT TCAATTACAC TATTAACCCT TGGCCTACTC 8750
64
Quality scores
0
AJ489607 consensus CCTTCTAACA TCAGGTCTAG TAATATGATT TCACTATAAT TCAATTACAC TATTAACCCT TGGCCTACTC 8750
64
Quality scores
0
Consensus CCTTCTAACA TCAGGTCTAG TAATATGATT TCACTATAAT TCAATTACAC TATTAACCCT TGGCCTACTC
100%
Conservation
0%
2.0bits
Sequence logo CCTTCTAACA TCAGGTCTAG TAATATGATT TCACTATAAT TCAATTACAC TATTAACCCT TGGCCTACTC
0.0bits

8,760 8,780 8,800 8,820

AJ489607 consensus ACCAATATCC TCACAATATA TCAATGATGA CGAGACGTAA TTCGTGAAGG AACCTACCAA GGCCACCACA 8820
64
Quality scores
0
AJ489607 consensus ACCAATATCC TCACAATATA TCAATGATGA CGAGACGTAA TTCGTGAAGG AACCTACCAA GGCCACCACA 8820
64
Quality scores
0
AJ489607 consensus ACCAATATCC TCACAATATA TCAATGATGA CGAGACGTAA TTCGTGAAGG AACCTACCAA GGCCACCACA 8820
64
Quality scores
0
AJ489607 consensus ACCAATATCC TCACAATATA TCAATGATGA CGAGACGTAA TTCGTGAAGG AACCTACCAA GGCCACCACA 8820
64
Quality scores
0
AJ489607 consensus ACCAATATCC TCACAATATA TCAATGATGA CGAGACGTAA TTCGTGAAGG AACCTACCAA GGCCACCACA 8820
64
Quality scores
0
AJ489607 consensus ACCAATATCC TCACAATATA TCAATGATGA CGAGACGTAA TTCGTGAAGG AACCTACCAA GGCCACCACA 8820
64
Quality scores
0
AJ489607 consensus ACCAATATCC TCACAATATA TCAATGATGA CGAGACGTAA TTCGTGAAGG AACCTACCAA GGCCACCACA 8820
64
Quality scores
0
AJ489607 consensus ACCAATATCC TCACAATATA TCAATGATGA CGAGACGTAA TTCGTGAAGG AACCTACCAA GGCCACCACA 8820
64
Quality scores
0
AJ489607 consensus ACCAATATCC TCACAATATA TCAATGATGA CGAGACGTAA TTCGTGAAGG AACCTACCAA GGCCACCACA 8820
64
Quality scores
0
Consensus ACCAATATCC TCACAATATA TCAATGATGA CGAGACGTAA TTCGTGAAGG AACCTACCAA GGCCACCACA
100%
Conservation
0%
2.0bits
Sequence logo ACCAATATCC TCACAATATA TCAATGATGA CGAGACGTAA TTCGTGAAGG AACCTACCAA GGCCACCACA
0.0bits

8,840 8,860 8,880

AJ489607 consensus CTCCTATTGT ACAAAAAGGA CTACGATATG GTATAATTCT ATTCATCGTC TCGGAAGTAT TTTTCTTTGC 8890
64
Quality scores
0
AJ489607 consensus CTCCTATTGT ACAAAAAGGA CTACGATATG GTATAATTCT ATTCATCGTC TCGGAAGTAT TTTTCTTTGC 8890
64
Quality scores
0
AJ489607 consensus CTCCTATTGT ACAAAAAGGA CTACGATATG GTATAATTCT ATTCATCGTC TCGGAAGTAT TTTTCTTTGC 8890
64
Quality scores
0
AJ489607 consensus CTCCTATTGT ACAAAAAGGA CTACGATATG GTATAATTCT ATTCATCGTC TCGGAAGTAT TTTTCTTTGC 8890
64
Quality scores
0
AJ489607 consensus CTCCTATTGT ACAAAAAGGA CTACGATATG GTATAATTCT ATTCATCGTC TCGGAAGTAT TTTTCTTTGC 8890
64
Quality scores
0
AJ489607 consensus CTCCTATTGT ACAAAAAGGA CTACGATATG GTATAATTCT ATTCATCGTC TCGGAAGTAT TTTTCTTTGC 8890
64
Quality scores
0
AJ489607 consensus CTCCTATTGT ACAAAAAGGA CTACGATATG GTATAATTCT ATTCATCGTC TCGGAAGTAT TTTTCTTTGC 8890
64
Quality scores
0
AJ489607 consensus CTCCTATTGT ACAAAAAGGA CTACGATATG GTATAATTCT ATTCATCGTC TCGGAAGTAT TTTTCTTTGC 8890
64
Quality scores
0
AJ489607 consensus CTCCTATTGT ACAAAAAGGA CTACGATATG GTATAATTCT ATTCATCGTC TCGGAAGTAT TTTTCTTTGC 8890
64
Quality scores
0
Consensus CTCCTATTGT ACAAAAAGGA CTACGATATG GTATAATTCT ATTCATCGTC TCGGAAGTAT TTTTCTTTGC
100%
Conservation
0%
2.0bits
Sequence logo CTCCTATTGT ACAAAAAGGA CTACGATATG GTATAATTCT ATTCATCGTC TCGGAAGTAT TTTTCTTTGC
0.0bits

8,900 8,920 8,940 8,960

AJ489607 consensus AGGATTCTTC TGAGCGTTCT ATCATTCTAG CCTCGTACCA ACACATGATC TAGGAGGCTG CTGACCTCCA 8960
64
Quality scores
0
AJ489607 consensus AGGATTCTTC TGAGCGTTCT ATCATTCTAG CCTCGTACCA ACACATGATC TAGGAGGCTG CTGACCTCCA 8960
64
Quality scores
0
AJ489607 consensus AGGATTCTTC TGAGCGTTCT ATCATTCTAG CCTCGTACCA ACACATGATC TAGGAGGCTG CTGACCTCCA 8960
64
Quality scores
0
AJ489607 consensus AGGATTCTTC TGAGCGTTCT ATCATTCTAG CCTCGTACCA ACACATGATC TAGGAGGCTG CTGACCTCCA 8960
64
Quality scores
0
AJ489607 consensus AGGATTCTTC TGAGCGTTCT ATCATTCTAG CCTCGTACCA ACACATGATC TAGGAGGCTG CTGACCTCCA 8960
64
Quality scores
0
AJ489607 consensus AGGATTCTTC TGAGCGTTCT ATCATTCTAG CCTCGTACCA ACACATGATC TAGGAGGCTG CTGACCTCCA 8960
64
Quality scores
0
AJ489607 consensus AGGATTCTTC TGAGCGTTCT ATCATTCTAG CCTCGTACCA ACACATGATC TAGGAGGCTG CTGACCTCCA 8960
64
Quality scores
0
AJ489607 consensus AGGATTCTTC TGAGCGTTCT ATCATTCTAG CCTCGTACCA ACACATGATC TAGGAGGCTG CTGACCTCCA 8960
64
Quality scores
0
AJ489607 consensus AGGATTCTTC TGAGCGTTCT ATCATTCTAG CCTCGTACCA ACACATGATC TAGGAGGCTG CTGACCTCCA 8960
64
Quality scores
0
Consensus AGGATTCTTC TGAGCGTTCT ATCATTCTAG CCTCGTACCA ACACATGATC TAGGAGGCTG CTGACCTCCA
100%
Conservation
0%
2.0bits
Sequence logo AGGATTCTTC TGAGCGTTCT ATCATTCTAG CCTCGTACCA ACACATGATC TAGGAGGCTG CTGACCTCCA
0.0bits

8,980 9,000 9,020

AJ489607 consensus ACAGGAATTT CACCACTTAA CCCTCTAGAA GTCCCACTAC TTAATACTTC AGTACTTCTA GCATCAGGTG 9030
Quality scores

AJ489607 consensus ACAGGAATTT CACCACTTAA CCCTCTAGAA GTCCCACTAC TTAATACTTC AGTACTTCTA GCATCAGGTG 9030
Quality scores

AJ489607 consensus ACAGGAATTT CACCACTTAA CCCTCTAGAA GTCCCACTAC TTAATACTTC AGTACTTCTA GCATCAGGTG 9030
Quality scores

AJ489607 consensus ACAGGAATTT CACCACTTAA CCCTCTAGAA GTCCCACTAC TTAATACTTC AGTACTTCTA GCATCAGGTG 9030
Quality scores

AJ489607 consensus ACAGGAATTT CACCACTTAA CCCTCTAGAA GTCCCACTAC TTAATACTTC AGTACTTCTA GCATCAGGTG 9030
Quality scores

AJ489607 consensus ACAGGAATTT CACCACTTAA CCCTCTAGAA GTCCCACTAC TTAATACTTC AGTACTTCTA GCATCAGGTG 9030
Quality scores

AJ489607 consensus ACAGGAATTT CACCACTTAA CCCTCTAGAA GTCCCACTAC TTAATACTTC AGTACTTCTA GCATCAGGTG 9030
Quality scores

AJ489607 consensus ACAGGAATTT CACCACTTAA CCCTCTAGAA GTCCCACTAC TTAATACTTC AGTACTTCTA GCATCAGGTG 9030
Quality scores

AJ489607 consensus ACAGGAATTT CACCACTTAA CCCTCTAGAA GTCCCACTAC TTAATACTTC AGTACTTCTA GCATCAGGTG 9030
Quality scores

Consensus ACAGGAATTT CACCACTTAA CCCTCTAGAA GTCCCACTAC TTAATACTTC AGTACTTCTA GCATCAGGTG
Conservation
Sequence logo ACAGGAATTT CACCACTTAA CCCTCTAGAA GTCCCACTAC TTAATACTTC AGTACTTCTA GCATCAGGTG

9,040 9,060 9,080 9,100

AJ489607 consensus TTTCAATTAC ATGAGCTCAT CATAGCCTTA TAGAAGGTAA ACGAAACCAC ATAAATCAAG CCCTACTAAT 9100
Quality scores

AJ489607 consensus TTTCAATTAC ATGAGCTCAT CATAGCCTTA TAGAAGGTAA ACGAAACCAC ATAAATCAAG CCCTACTAAT 9100
Quality scores

AJ489607 consensus TTTCAATTAC ATGAGCTCAT CATAGCCTTA TAGAAGGTAA ACGAAACCAC ATAAATCAAG CCCTACTAAT 9100
Quality scores

AJ489607 consensus TTTCAATTAC ATGAGCTCAT CATAGCCTTA TAGAAGGTAA ACGAAACCAC ATAAATCAAG CCCTACTAAT 9100
Quality scores

AJ489607 consensus TTTCAATTAC ATGAGCTCAT CATAGCCTTA TAGAAGGTAA ACGAAACCAC ATAAATCAAG CCCTACTAAT 9100
Quality scores

AJ489607 consensus TTTCAATTAC ATGAGCTCAT CATAGCCTTA TAGAAGGTAA ACGAAACCAC ATAAATCAAG CCCTACTAAT 9100
Quality scores

AJ489607 consensus TTTCAATTAC ATGAGCTCAT CATAGCCTTA TAGAAGGTAA ACGAAACCAC ATAAATCAAG CCCTACTAAT 9100
Quality scores

AJ489607 consensus TTTCAATTAC ATGAGCTCAT CATAGCCTTA TAGAAGGTAA ACGAAACCAC ATAAATCAAG CCCTACTAAT 9100
Quality scores

AJ489607 consensus TTTCAATTAC ATGAGCTCAT CATAGCCTTA TAGAAGGTAA ACGAAACCAC ATAAATCAAG CCCTACTAAT 9100
Quality scores

Consensus TTTCAATTAC ATGAGCTCAT CATAGCCTTA TAGAAGGTAA ACGAAACCAC ATAAATCAAG CCCTACTAAT
Conservation
Sequence logo TTTCAATTAC ATGAGCTCAT CATAGCCTTA TAGAAGGTAA ACGAAACCAC ATAAATCAAG CCCTACTAAT

9,120 9,140 9,160

AJ489607 consensus TACCATTATA CTAGGACTTT ACTTCACCAT CCTCCAAGCT TCAGAATACT TTGAAACATC ATTCTCCATT 9170
64 Quality scores 0
AJ489607 consensus TACCATTATA CTAGGACTTT ACTTCACCAT CCTCCAAGCT TCAGAATACT TTGAAACATC ATTCTCCATT 9170
64 Quality scores 0
AJ489607 consensus TACCATTATA CTAGGACTTT ACTTCACCAT CCTCCAAGCT TCAGAATACT TTGAAACATC ATTCTCCATT 9170
64 Quality scores 0
AJ489607 consensus TACCATTATA CTAGGACTTT ACTTCACCAT CCTCCAAGCT TCAGAATACT TTGAAACATC ATTCTCCATT 9170
64 Quality scores 0
AJ489607 consensus TACCATTATA CTAGGACTTT ACTTCACCAT CCTCCAAGCT TCAGAATACT TTGAAACATC ATTCTCCATT 9170
64 Quality scores 0
AJ489607 consensus TACCATTATA CTAGGACTTT ACTTCACCAT CCTCCAAGCT TCAGAATACT TTGAAACATC ATTCTCCATT 9170
64 Quality scores 0
AJ489607 consensus TACCATTATA CTAGGACTTT ACTTCACCAT CCTCCAAGCT TCAGAATACT TTGAAACATC ATTCTCCATT 9170
64 Quality scores 0
AJ489607 consensus TACCATTATA CTAGGACTTT ACTTCACCAT CCTCCAAGCT TCAGAATACT TTGAAACATC ATTCTCCATT 9170
64 Quality scores 0
AJ489607 consensus TACCATTATA CTAGGACTTT ACTTCACCAT CCTCCAAGCT TCAGAATACT TTGAAACATC ATTCTCCATT 9170
64 Quality scores 0
Consensus TACCATTATA CTAGGACTTT ACTTCACCAT CCTCCAAGCT TCAGAATACT TTGAAACATC ATTCTCCATT
100% Conservation 0%
2.0bits Sequence logo 0.0bits TACCATTATA CTAGGACTTT ACTTCACCAT CCTCCAAGCT TCAGAATACT TTGAAACATC ATTCTCCATT

9,180 9,200 9,220 9,240

AJ489607 consensus TCAGATGGTA TCTATGGTTC TACATTCTTC ATGGCTACTG GATTCCATGG ACTCCATGTA ATTATTGGAT 9240
64 Quality scores 0
AJ489607 consensus TCAGATGGTA TCTATGGTTC TACATTCTTC ATGGCTACTG GATTCCATGG ACTCCATGTA ATTATTGGAT 9240
64 Quality scores 0
AJ489607 consensus TCAGATGGTA TCTATGGTTC TACATTCTTC ATGGCTACTG GATTCCATGG ACTCCATGTA ATTATTGGAT 9240
64 Quality scores 0
AJ489607 consensus TCAGATGGTA TCTATGGTTC TACATTCTTC ATGGCTACTG GATTCCATGG ACTCCATGTA ATTATTGGAT 9240
64 Quality scores 0
AJ489607 consensus TCAGATGGTA TCTATGGTTC TACATTCTTC ATGGCTACTG GATTCCATGG ACTCCATGTA ATTATTGGAT 9240
64 Quality scores 0
AJ489607 consensus TCAGATGGTA TCTATGGTTC TACATTCTTC ATGGCTACTG GATTCCATGG ACTCCATGTA ATTATTGGAT 9240
64 Quality scores 0
AJ489607 consensus TCAGATGGTA TCTATGGTTC TACATTCTTC ATGGCTACTG GATTCCATGG ACTCCATGTA ATTATTGGAT 9240
64 Quality scores 0
AJ489607 consensus TCAGATGGTA TCTATGGTTC TACATTCTTC ATGGCTACTG GATTCCATGG ACTCCATGTA ATTATTGGAT 9240
64 Quality scores 0
AJ489607 consensus TCAGATGGTA TCTATGGTTC TACATTCTTC ATGGCTACTG GATTCCATGG ACTCCATGTA ATTATTGGAT 9240
64 Quality scores 0
Consensus TCAGATGGTA TCTATGGTTC TACATTCTTC ATGGCTACTG GATTCCATGG ACTCCATGTA ATTATTGGAT
100% Conservation 0%
2.0bits Sequence logo 0.0bits TCAGATGGTA TCTATGGTTC TACATTCTTC ATGGCTACTG GATTCCATGG ACTCCATGTA ATTATTGGAT

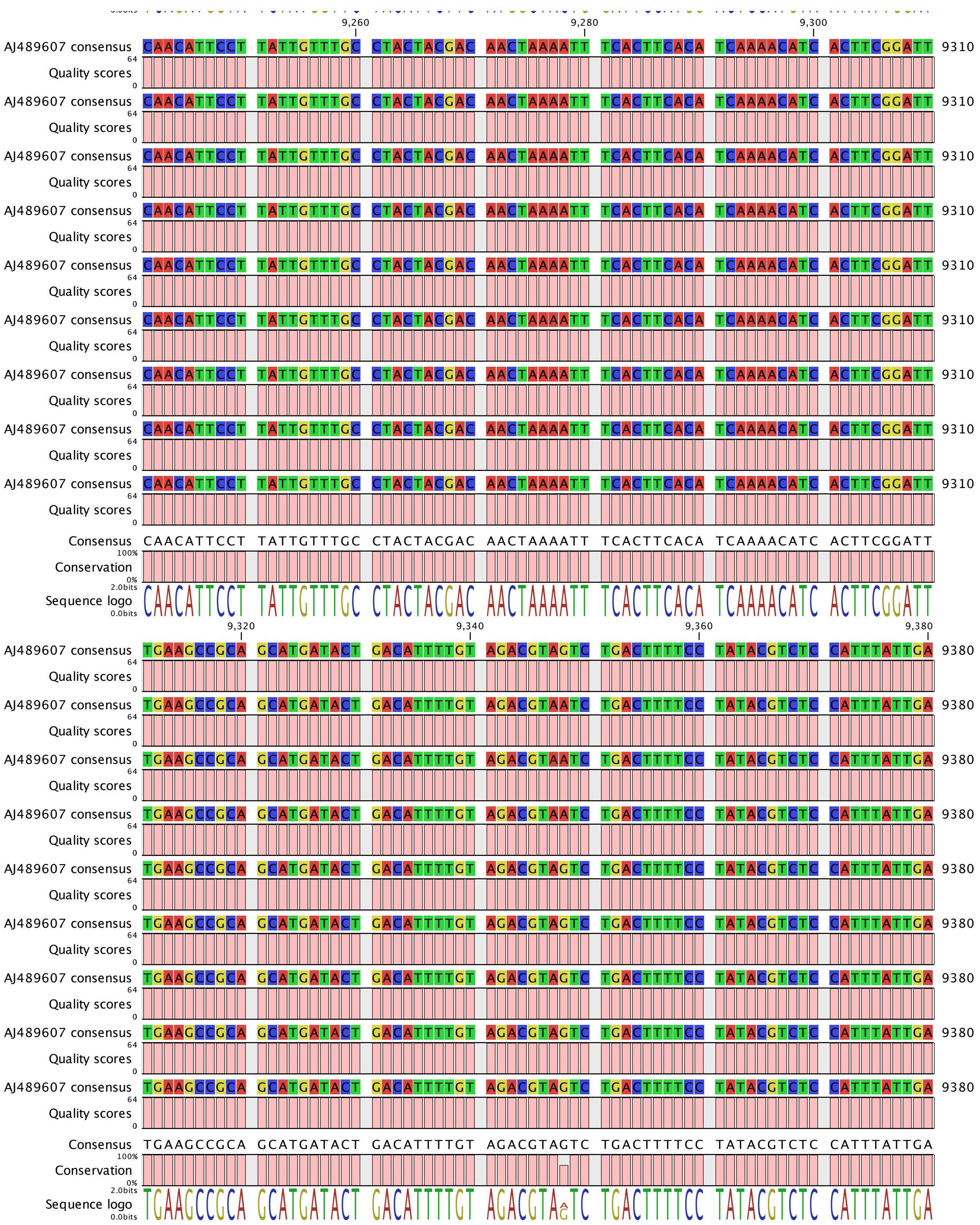
9,260
9,280
9,300
AJ489607 consensus CAACATTCCT TATTGTTTGC CTACTACGAC AACTAAAATT TCACTTCACA TCAAAACATC ACTTCGGATT 9310
Quality scores
AJ489607 consensus CAACATTCCT TATTGTTTGC CTACTACGAC AACTAAAATT TCACTTCACA TCAAAACATC ACTTCGGATT 9310
Quality scores
AJ489607 consensus CAACATTCCT TATTGTTTGC CTACTACGAC AACTAAAATT TCACTTCACA TCAAAACATC ACTTCGGATT 9310
Quality scores
AJ489607 consensus CAACATTCCT TATTGTTTGC CTACTACGAC AACTAAAATT TCACTTCACA TCAAAACATC ACTTCGGATT 9310
Quality scores
AJ489607 consensus CAACATTCCT TATTGTTTGC CTACTACGAC AACTAAAATT TCACTTCACA TCAAAACATC ACTTCGGATT 9310
Quality scores
AJ489607 consensus CAACATTCCT TATTGTTTGC CTACTACGAC AACTAAAATT TCACTTCACA TCAAAACATC ACTTCGGATT 9310
Quality scores
AJ489607 consensus CAACATTCCT TATTGTTTGC CTACTACGAC AACTAAAATT TCACTTCACA TCAAAACATC ACTTCGGATT 9310
Quality scores
AJ489607 consensus CAACATTCCT TATTGTTTGC CTACTACGAC AACTAAAATT TCACTTCACA TCAAAACATC ACTTCGGATT 9310
Quality scores
AJ489607 consensus CAACATTCCT TATTGTTTGC CTACTACGAC AACTAAAATT TCACTTCACA TCAAAACATC ACTTCGGATT 9310
Quality scores
Consensus CAACATTCCT TATTGTTTGC CTACTACGAC AACTAAAATT TCACTTCACA TCAAAACATC ACTTCGGATT
Conservation
Sequence logo
9,320
9,340
9,360
9,380
AJ489607 consensus TGAAGCCGCA GCATGATACT GACATTTTGT AGACGTAGTC TGACTTTTCC TATACGTCTC CATTTATTGA 9380
Quality scores
AJ489607 consensus TGAAGCCGCA GCATGATACT GACATTTTGT AGACGTAATC TGACTTTTCC TATACGTCTC CATTTATTGA 9380
Quality scores
AJ489607 consensus TGAAGCCGCA GCATGATACT GACATTTTGT AGACGTAATC TGACTTTTCC TATACGTCTC CATTTATTGA 9380
Quality scores
AJ489607 consensus TGAAGCCGCA GCATGATACT GACATTTTGT AGACGTAATC TGACTTTTCC TATACGTCTC CATTTATTGA 9380
Quality scores
AJ489607 consensus TGAAGCCGCA GCATGATACT GACATTTTGT AGACGTAGTC TGACTTTTCC TATACGTCTC CATTTATTGA 9380
Quality scores
AJ489607 consensus TGAAGCCGCA GCATGATACT GACATTTTGT AGACGTAGTC TGACTTTTCC TATACGTCTC CATTTATTGA 9380
Quality scores
AJ489607 consensus TGAAGCCGCA GCATGATACT GACATTTTGT AGACGTAGTC TGACTTTTCC TATACGTCTC CATTTATTGA 9380
Quality scores
AJ489607 consensus TGAAGCCGCA GCATGATACT GACATTTTGT AGACGTAGTC TGACTTTTCC TATACGTCTC CATTTATTGA 9380
Quality scores
AJ489607 consensus TGAAGCCGCA GCATGATACT GACATTTTGT AGACGTAGTC TGACTTTTCC TATACGTCTC CATTTATTGA 9380
Quality scores
Consensus TGAAGCCGCA GCATGATACT GACATTTTGT AGACGTAGTC TGACTTTTCC TATACGTCTC CATTTATTGA
Conservation
Sequence logo

9,400 9,420 9,440

AJ489607 consensus TGAGGATCTT ACTCCCTTAG TATAATTAAT ATAACTGACT TCCAATTAGT AGATTCTGAA TAAACCCAGA 9450
Quality scores
AJ489607 consensus TGAGGATCTT ACTCCCTTAG TATAATTAAT ATAACTGACT TCCAATTAGT AGATTCTGAA TAAACCCAGA 9450
Quality scores
AJ489607 consensus TGAGGATCTT ACTCCCTTAG TATAATTAAT ATAACTGACT TCCAATTAGT AGATTCTGAA TAAACCCAGA 9450
Quality scores
AJ489607 consensus TGAGGATCTT ACTCCCTTAG TATAATTAAT ATAACTGACT TCCAATTAGT AGATTCTGAA TAAACCCAGA 9450
Quality scores
AJ489607 consensus TGAGGATCTT ACTCCCTTAG TATAATTAAT ATAACTGACT TCCAATTAGT AGATTCTGAA TAAACCCAGA 9450
Quality scores
AJ489607 consensus TGAGGATCTT ACTCCCTTAG TATAATTAAT ATAACTGACT TCCAATTAGT AGATTCTGAA TAAACCCAGA 9450
Quality scores
AJ489607 consensus TGAGGATCTT ACTCCCTTAG TATAATTAAT ATAACTGACT TCCAATTAGT AGATTCTGAA TAAACCCAGA 9450
Quality scores
AJ489607 consensus TGAGGATCTT ACTCCCTTAG TATAATTAAT ATAACTGACT TCCAATTAGT AGATTCTGAA TAAACCCAGA 9450
Quality scores
AJ489607 consensus TGAGGATCTT ACTCCCTTAG TATAATTAAT ATAACTGACT TCCAATTAGT AGATTCTGAA TAAACCCAGA 9450
Quality scores
Consensus TGAGGATCTT ACTCCCTTAG TATAATTAAT ATAACTGACT TCCAATTAGT AGATTCTGAA TAAACCCAGA
Conservation
Sequence logo

9,460 9,480 9,500 9,520

AJ489607 consensus AGAGAGTAAT TAACCTGTAC ACTGTTATCT TCATTAATAT TTTATTATCC CTAACGCTAA TTCTAGTTGC 9520
Quality scores
AJ489607 consensus AGAGAGTAAT CAACCTGTAC ACTGTTATCT TCATTAATAT TTTATTATCC CTAACGCTAA TTCTAGTTGC 9520
Quality scores
AJ489607 consensus AGAGAGTAAT CAACCTGTAC ACTGTTATCT TCATTAATAT TTTATTATCC CTAACGCTAA TTCTAGTTGC 9520
Quality scores
AJ489607 consensus AGAGAGTAAT CAACCTGTAC ACTGTTATCT TCATTAATAT TTTATTATCC CTAACGCTAA TTCTAGTTGC 9520
Quality scores
AJ489607 consensus AGAGAGTAAT TAACCTGTAC ACTGTTATCT TCATTAATAT TTTATTATCC CTAACGCTAA TTCTAGTTGC 9520
Quality scores
AJ489607 consensus AGAGAGTAAT TAACCTGTAC ACTGTTATCT TCATTAATAT TTTATTATCC CTAACGCTAA TTCTAGTTGC 9520
Quality scores
AJ489607 consensus AGAGAGTAAT TAACCTGTAC ACTGTTATCT TCATTAATAT TTTATTATCC CTAACGCTAA TTCTAGTTGC 9520
Quality scores
AJ489607 consensus AGAGAGTAAT TAACCTGTAC ACTGTTATCT TCATTAATAT TTTATTATCC CTAACGCTAA TTCTAGTTGC 9520
Quality scores
AJ489607 consensus AGAGAGTAAT TAACCTGTAC ACTGTTATCT TCATTAATAT TTTATTATCC CTAACGCTAA TTCTAGTTGC 9520
Quality scores
Consensus AGAGAGTAAT TAACCTGTAC ACTGTTATCT TCATTAATAT TTTATTATCC CTAACGCTAA TTCTAGTTGC
Conservation
Sequence logo

9,540 9,560 9,580

AJ489607 consensus ATTCTGACTC CCCCAAATAA ATCTGTACTC AGAAAAAGCA AATCCATATG AATGCGGATT CGACCCTACA 9590
64 Quality scores 0
AJ489607 consensus ATTCTGACTC CCCCAAATAA ATCTGTACTC AGAAAAAGCA AATCCATATG AATGCGGATT CGACCCTACA 9590
64 Quality scores 0
AJ489607 consensus ATTCTGACTC CCCCAAATAA ATCTGTACTC AGAAAAAGCA AATCCATATG AATGCGGATT CGACCCTACA 9590
64 Quality scores 0
AJ489607 consensus ATTCTGACTC CCCCAAATAA ATCTGTACTC AGAAAAAGCA AATCCATATG AATGCGGATT CGACCCTACA 9590
64 Quality scores 0
AJ489607 consensus ATTCTGACTC CCCCAAATAA ATCTGTACTC AGAAAAAGCA AATCCATATG AATGCGGATT CGACCCTACA 9590
64 Quality scores 0
AJ489607 consensus ATTCTGACTC CCCCAAATAA ATCTGTACTC AGAAAAAGCA AATCCATATG AATGCGGATT CGACCCTACA 9590
64 Quality scores 0
AJ489607 consensus ATTCTGACTC CCCCAAATAA ATCTGTACTC AGAAAAAGCA AATCCATATG AATGCGGATT CGACCCTACA 9590
64 Quality scores 0
AJ489607 consensus ATTCTGACTC CCCCAAATAA ATCTGTACTC AGAAAAAGCA AATCCATATG AATGCGGATT CGACCCTACA 9590
64 Quality scores 0
AJ489607 consensus ATTCTGACTC CCCCAAATAA ATCTGTACTC AGAAAAAGCA AATCCATATG AATGCGGATT CGACCCTACA 9590
64 Quality scores 0
Consensus ATTCTGACTC CCCCAAATAA ATCTGTACTC AGAAAAAGCA AATCCATATG AATGCGGATT CGACCCTACA
100% Conservation 0%
2.0bits Sequence logo 0.0bits ATTCTGACTC CCCCAAATAA ATCTGTACTC AGAAAAAGCA AATCCATATG AATGCGGATT CGACCCTACA

9,600 9,620 9,640 9,660

AJ489607 consensus AGCTCTGCAC GTCTACCATT CTCAATAAAA TTTTTCTTGG TAGCAATTAC ATTTCTATTA TTTGACCTAG 9660
64 Quality scores 0
AJ489607 consensus AGCTCTGCAC GTCTACCATT CTCAATAAAA TTTTTCTTGG TAGCAATTAC ATTTCTATTA TTTGACCTAG 9660
64 Quality scores 0
AJ489607 consensus AGCTCTGCAC GTCTACCATT CTCAATAAAA TTTTTCTTGG TAGCAATTAC ATTTCTATTA TTTGACCTAG 9660
64 Quality scores 0
AJ489607 consensus AGCTCTGCAC GTCTACCATT CTCAATAAAA TTTTTCTTGG TAGCAATTAC ATTTCTATTA TTTGACCTAG 9660
64 Quality scores 0
AJ489607 consensus AGCTCTGCAC GTCTACCATT CTCAATAAAA TTTTTCTTGG TAGCAATTAC ATTTCTATTA TTTGACCTAG 9660
64 Quality scores 0
AJ489607 consensus AGCTCTGCAC GTCTACCATT CTCAATAAAA TTTTTCTTGG TAGCAATTAC ATTTCTATTA TTTGACCTAG 9660
64 Quality scores 0
AJ489607 consensus AGCTCTGCAC GTCTACCATT CTCAATAAAA TTTTTCTTGG TAGCAATTAC ATTTCTATTA TTTGACCTAG 9660
64 Quality scores 0
AJ489607 consensus AGCTCTGCAC GTCTACCATT CTCAATAAAA TTTTTCTTGG TAGCAATTAC ATTTCTATTA TTTGACCTAG 9660
64 Quality scores 0
AJ489607 consensus AGCTCTGCAC GTCTACCATT CTCAATAAAA TTTTTCTTGG TAGCAATTAC ATTTCTATTA TTTGACCTAG 9660
64 Quality scores 0
Consensus AGCTCTGCAC GTCTACCATT CTCAATAAAA TTTTTCTTGG TAGCAATTAC ATTTCTATTA TTTGACCTAG
100% Conservation 0%
2.0bits Sequence logo 0.0bits AGCTCTGCAC GTCTACCATT CTCAATAAAA TTTTTCTTGG TAGCAATTAC ATTTCTATTA TTTGACCTAG

0.0bits

9,680 9,700 9,720

AJ489607 consensus AAATTGCTCT TCTACTTCCA CTACCATGAG CAATTCAAAC AATTAAAACC TCTACTATAA TAATTATAGC 9730
64 Quality scores 0
AJ489607 consensus AAATTGCTCT TCTACTTCCA CTACCATGAG CAATTCAAAC AATTAAAACC TCTACTATAA TAATTATAGC 9730
64 Quality scores 0
AJ489607 consensus AAATTGCTCT TCTACTTCCA CTACCATGAG CAATTCAAAC AATTAAAACC TCTACTATAA TAATTATAGC 9730
64 Quality scores 0
AJ489607 consensus AAATTGCTCT TCTACTTCCA CTACCATGAG CAATTCAAAC AATTAAAACC TCTACTATAA TAATTATAGC 9730
64 Quality scores 0
AJ489607 consensus AAATTGCTCT TCTACTTCCA CTACCATGAG CAATTCAAAC AATTAAAACC TCTACTATAA TAATTATAGC 9730
64 Quality scores 0
AJ489607 consensus AAATTGCTCT TCTACTTCCA CTACCATGAG CAATTCAAAC AATTAAAACC TCTACTATAA TAATTATAGC 9730
64 Quality scores 0
AJ489607 consensus AAATTGCTCT TCTACTTCCA CTACCATGAG CAATTCAAAC AATTAAAACC TCTACTATAA TAATTATAGC 9730
64 Quality scores 0
AJ489607 consensus AAATTGCTCT TCTACTTCCA CTACCATGAG CAATTCAAAC AATTAAAACC TCTACTATAA TAATTATAGC 9730
64 Quality scores 0
AJ489607 consensus AAATTGCTCT TCTACTTCCA CTACCATGAG CAATTCAAAC AATTAAAACC TCTACTATAA TAATTATAGC 9730
64 Quality scores 0

Consensus AAATTGCTCT TCTACTTCCA CTACCATGAG CAATTCAAAC AATTAAAACC TCTACTATAA TAATTATAGC
100% Conservation 0%
2.0bits Sequence logo 0.0bits AAATTGCTCT TCTACTTCCA CTACCATGAG CAATTCAAAC AATTAAAACC TCTACTATAA TAATTATAGC

9,740 9,760 9,780 9,800

AJ489607 consensus CTTTATTCTA GTCACAATTC TATCTCTAGG CCTAGCATAT GAATGAACAC AAAAAGGATT AGAATGAACA 9800
64 Quality scores 0
AJ489607 consensus CTTTATTCTA GTCACAATTC TATCTCTAGG CCTAGCATAT GAATGAACAC AAAAAGGATT AGAATGAACA 9800
64 Quality scores 0
AJ489607 consensus CTTTATTCTA GTCACAATTC TATCTCTAGG CCTAGCATAT GAATGAACAC AAAAAGGATT AGAATGAACA 9800
64 Quality scores 0
AJ489607 consensus CTTTATTCTA GTCACAATTC TATCTCTAGG CCTAGCATAT GAATGAACAC AAAAAGGATT AGAATGAACA 9800
64 Quality scores 0
AJ489607 consensus CTTTATTCTA GTCACAATTC TATCTCTAGG CCTAGCATAT GAATGAACAC AAAAAGGATT AGAATGAACA 9800
64 Quality scores 0
AJ489607 consensus CTTTATTCTA GTCACAATTC TATCTCTAGG CCTAGCATAT GAATGAACAC AAAAAGGATT AGAATGAACA 9800
64 Quality scores 0
AJ489607 consensus CTTTATTCTA GTCACAATTC TATCTCTAGG CCTAGCATAT GAATGAACAC AAAAAGGATT AGAATGAACA 9800
64 Quality scores 0
AJ489607 consensus CTTTATTCTA GTCACAATTC TATCTCTAGG CCTAGCATAT GAATGAACAC AAAAAGGATT AGAATGAACA 9800
64 Quality scores 0
AJ489607 consensus CTTTATTCTA GTCACAATTC TATCTCTAGG CCTAGCATAT GAATGAACAC AAAAAGGATT AGAATGAACA 9800
64 Quality scores 0

Consensus CTTTATTCTA GTCACAATTC TATCTCTAGG CCTAGCATAT GAATGAACAC AAAAAGGATT AGAATGAACA
100% Conservation 0%
2.0bits Sequence logo 0.0bits CTTTATTCTA GTCACAATTC TATCTCTAGG CCTAGCATAT GAATGAACAC AAAAAGGATT AGAATGAACA

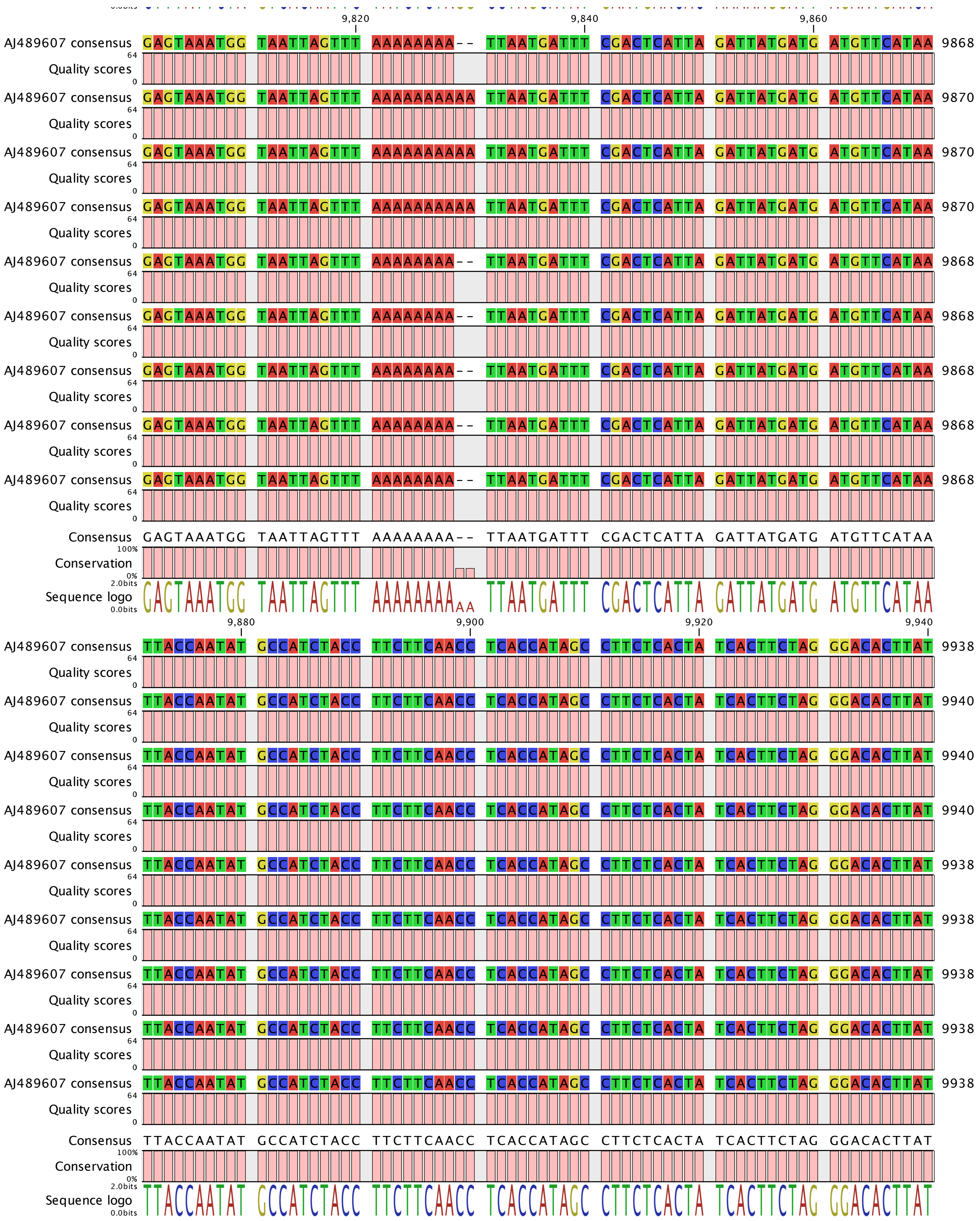
9,820
9,840
9,860
AJ489607 consensus GAGTAAATGG TAATTAGTTT AAAAAAAA-- TTAATGATTT CGACTCATTA GATTATGATG ATGTTCATAA 9868
Quality scores
AJ489607 consensus GAGTAAATGG TAATTAGTTT AAAAAAAAAA TTAATGATTT CGACTCATTA GATTATGATG ATGTTCATAA 9870
Quality scores
AJ489607 consensus GAGTAAATGG TAATTAGTTT AAAAAAAAAA TTAATGATTT CGACTCATTA GATTATGATG ATGTTCATAA 9870
Quality scores
AJ489607 consensus GAGTAAATGG TAATTAGTTT AAAAAAAAAA TTAATGATTT CGACTCATTA GATTATGATG ATGTTCATAA 9870
Quality scores
AJ489607 consensus GAGTAAATGG TAATTAGTTT AAAAAAAA-- TTAATGATTT CGACTCATTA GATTATGATG ATGTTCATAA 9868
Quality scores
AJ489607 consensus GAGTAAATGG TAATTAGTTT AAAAAAAA-- TTAATGATTT CGACTCATTA GATTATGATG ATGTTCATAA 9868
Quality scores
AJ489607 consensus GAGTAAATGG TAATTAGTTT AAAAAAAA-- TTAATGATTT CGACTCATTA GATTATGATG ATGTTCATAA 9868
Quality scores
AJ489607 consensus GAGTAAATGG TAATTAGTTT AAAAAAAA-- TTAATGATTT CGACTCATTA GATTATGATG ATGTTCATAA 9868
Quality scores
AJ489607 consensus GAGTAAATGG TAATTAGTTT AAAAAAAA-- TTAATGATTT CGACTCATTA GATTATGATG ATGTTCATAA 9868
Quality scores
Consensus GAGTAAATGG TAATTAGTTT AAAAAAAA-- TTAATGATTT CGACTCATTA GATTATGATG ATGTTCATAA
Conservation
Sequence logo
9,880
9,900
9,920
9,940
AJ489607 consensus TTACCAATAT GCCATCTACC TTCTTCAACC TCACCATAGC CTTCTCACTA TCACTTCTAG GGACACTTAT 9938
Quality scores
AJ489607 consensus TTACCAATAT GCCATCTACC TTCTTCAACC TCACCATAGC CTTCTCACTA TCACTTCTAG GGACACTTAT 9940
Quality scores
AJ489607 consensus TTACCAATAT GCCATCTACC TTCTTCAACC TCACCATAGC CTTCTCACTA TCACTTCTAG GGACACTTAT 9940
Quality scores
AJ489607 consensus TTACCAATAT GCCATCTACC TTCTTCAACC TCACCATAGC CTTCTCACTA TCACTTCTAG GGACACTTAT 9940
Quality scores
AJ489607 consensus TTACCAATAT GCCATCTACC TTCTTCAACC TCACCATAGC CTTCTCACTA TCACTTCTAG GGACACTTAT 9938
Quality scores
AJ489607 consensus TTACCAATAT GCCATCTACC TTCTTCAACC TCACCATAGC CTTCTCACTA TCACTTCTAG GGACACTTAT 9938
Quality scores
AJ489607 consensus TTACCAATAT GCCATCTACC TTCTTCAACC TCACCATAGC CTTCTCACTA TCACTTCTAG GGACACTTAT 9938
Quality scores
AJ489607 consensus TTACCAATAT GCCATCTACC TTCTTCAACC TCACCATAGC CTTCTCACTA TCACTTCTAG GGACACTTAT 9938
Quality scores
AJ489607 consensus TTACCAATAT GCCATCTACC TTCTTCAACC TCACCATAGC CTTCTCACTA TCACTTCTAG GGACACTTAT 9938
Quality scores
Consensus TTACCAATAT GCCATCTACC TTCTTCAACC TCACCATAGC CTTCTCACTA TCACTTCTAG GGACACTTAT
Conservation
Sequence logo

0.0bits

9,960 9,980 10,000

AJ489607 consensus ATTTCGCTCT CACCTAATAT CCACATTACT ATGCCTGGAA GGCATAGTAT TATCCTTATT TATTATAACT 10008
64 Quality scores 0
AJ489607 consensus ATTTCGCTCT CACCTAATAT CCACATTACT ATGCCTGGAA GGCATAGTAT TATCCTTATT TATTATAACT 10010
64 Quality scores 0
AJ489607 consensus ATTTCGCTCT CACCTAATAT CCACATTACT ATGCCTGGAA GGCATAGTAT TATCCTTATT TATTATAACT 10010
64 Quality scores 0
AJ489607 consensus ATTTCGCTCT CACCTAATAT CCACATTACT ATGCCTGGAA GGCATAGTAT TATCCTTATT TATTATAACT 10010
64 Quality scores 0
AJ489607 consensus ATTTCGCTCT CACCTAATAT CCACATTACT ATGCCTGGAA GGCATAGTAT TATCCTTATT TATTATAACT 10008
64 Quality scores 0
AJ489607 consensus ATTTCGCTCT CACCTAATAT CCACATTACT ATGCCTGGAA GGCATAGTAT TATCCTTATT TATTATAACT 10008
64 Quality scores 0
AJ489607 consensus ATTTCGCTCT CACCTAATAT CCACATTACT ATGCCTGGAA GGCATAGTAT TATCCTTATT TATTATAACT 10008
64 Quality scores 0
AJ489607 consensus ATTTCGCTCT CACCTAATAT CCACATTACT ATGCCTGGAA GGCATAGTAT TATCCTTATT TATTATAACT 10008
64 Quality scores 0
AJ489607 consensus ATTTCGCTCT CACCTAATAT CCACATTACT ATGCCTGGAA GGCATAGTAT TATCCTTATT TATTATAACT 10008
64 Quality scores 0
Consensus ATTTCGCTCT CACCTAATAT CCACATTACT ATGCCTGGAA GGCATAGTAT TATCCTTATT TATTATAACT
100% Conservation 0%
2.0bits Sequence logo 0.0bits ATTTCGCTCT CACCTAATAT CCACATTACT ATGCCTGGAA GGCATAGTAT TATCCTTATT TATTATAACT

10,020 10,040 10,060 10,080

AJ489607 consensus TCAGTAACTT CCCTAAACTC CAACTCCATA AGCTCCATAC CAATCCCCAT CACCATCTTA GTTTTCGCAG 10078
64 Quality scores 0
AJ489607 consensus TCAGTAACTT CCCTAAACTC CAACTCCATA AGCTCCATAC CAATCCCCAT CACCATCTTA GTTTTCGCAG 10080
64 Quality scores 0
AJ489607 consensus TCAGTAACTT CCCTAAACTC CAACTCCATA AGCTCCATAC CAATCCCCAT CACCATCTTA GTTTTCGCAG 10080
64 Quality scores 0
AJ489607 consensus TCAGTAACTT CCCTAAACTC CAACTCCATA AGCTCCATAC CAATCCCCAT CACCATCTTA GTTTTCGCAG 10080
64 Quality scores 0
AJ489607 consensus TCAGTAACTT CCCTAAACTC CAACTCCATA AGCTCCATAC CAATCCCCAT CACCATCTTA GTTTTCGCAG 10078
64 Quality scores 0
AJ489607 consensus TCAGTAACTT CCCTAAACTC CAACTCCATA AGCTCCATAC CAATCCCCAT CACCATCTTA GTTTTCGCAG 10078
64 Quality scores 0
AJ489607 consensus TCAGTAACTT CCCTAAACTC CAACTCCATA AGCTCCATAC CAATCCCCAT CACCATCTTA GTTTTCGCAG 10078
64 Quality scores 0
AJ489607 consensus TCAGTAACTT CCCTAAACTC CAACTCCATA AGCTCCATAC CAATCCCCAT CACCATCTTA GTTTTCGCAG 10078
64 Quality scores 0
AJ489607 consensus TCAGTAACTT CCCTAAACTC CAACTCCATA AGCTCCATAC CAATCCCCAT CACCATCTTA GTTTTCGCAG 10078
64 Quality scores 0
Consensus TCAGTAACTT CCCTAAACTC CAACTCCATA AGCTCCATAC CAATCCCCAT CACCATCTTA GTTTTCGCAG
100% Conservation 0%
2.0bits Sequence logo 0.0bits TCAGTAACTT CCCTAAACTC CAACTCCATA AGCTCCATAC CAATCCCCAT CACCATCTTA GTTTTCGCAG

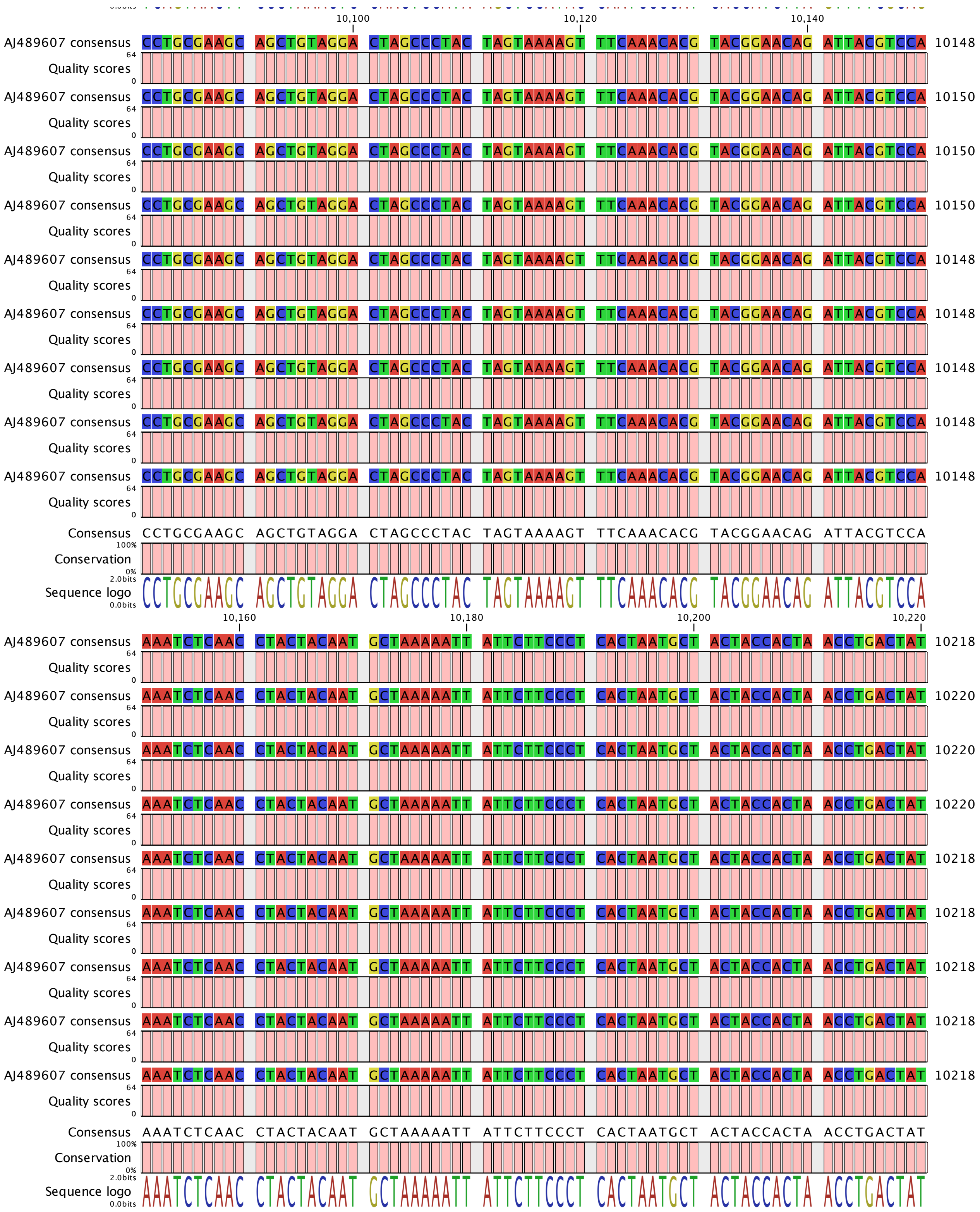
10,100
10,120
10,140
AJ489607 consensus CCTGCGAAGC AGCTGTAGGA CTAGCCCTAC TAGTAAAAGT TTCAAACACG TACGGAACAG ATTACGTCCA 10148
Quality scores
AJ489607 consensus CCTGCGAAGC AGCTGTAGGA CTAGCCCTAC TAGTAAAAGT TTCAAACACG TACGGAACAG ATTACGTCCA 10150
Quality scores
AJ489607 consensus CCTGCGAAGC AGCTGTAGGA CTAGCCCTAC TAGTAAAAGT TTCAAACACG TACGGAACAG ATTACGTCCA 10150
Quality scores
AJ489607 consensus CCTGCGAAGC AGCTGTAGGA CTAGCCCTAC TAGTAAAAGT TTCAAACACG TACGGAACAG ATTACGTCCA 10150
Quality scores
AJ489607 consensus CCTGCGAAGC AGCTGTAGGA CTAGCCCTAC TAGTAAAAGT TTCAAACACG TACGGAACAG ATTACGTCCA 10148
Quality scores
AJ489607 consensus CCTGCGAAGC AGCTGTAGGA CTAGCCCTAC TAGTAAAAGT TTCAAACACG TACGGAACAG ATTACGTCCA 10148
Quality scores
AJ489607 consensus CCTGCGAAGC AGCTGTAGGA CTAGCCCTAC TAGTAAAAGT TTCAAACACG TACGGAACAG ATTACGTCCA 10148
Quality scores
AJ489607 consensus CCTGCGAAGC AGCTGTAGGA CTAGCCCTAC TAGTAAAAGT TTCAAACACG TACGGAACAG ATTACGTCCA 10148
Quality scores
AJ489607 consensus CCTGCGAAGC AGCTGTAGGA CTAGCCCTAC TAGTAAAAGT TTCAAACACG TACGGAACAG ATTACGTCCA 10148
Quality scores
Consensus CCTGCGAAGC AGCTGTAGGA CTAGCCCTAC TAGTAAAAGT TTCAAACACG TACGGAACAG ATTACGTCCA
Conservation
Sequence logo
10,160
10,180
10,200
10,220
AJ489607 consensus AAATCTCAAC CTACTACAAT GCTAAAAATT ATTCTTCCCT CACTAATGCT ACTACCACTA ACCTGACTAT 10218
Quality scores
AJ489607 consensus AAATCTCAAC CTACTACAAT GCTAAAAATT ATTCTTCCCT CACTAATGCT ACTACCACTA ACCTGACTAT 10220
Quality scores
AJ489607 consensus AAATCTCAAC CTACTACAAT GCTAAAAATT ATTCTTCCCT CACTAATGCT ACTACCACTA ACCTGACTAT 10220
Quality scores
AJ489607 consensus AAATCTCAAC CTACTACAAT GCTAAAAATT ATTCTTCCCT CACTAATGCT ACTACCACTA ACCTGACTAT 10220
Quality scores
AJ489607 consensus AAATCTCAAC CTACTACAAT GCTAAAAATT ATTCTTCCCT CACTAATGCT ACTACCACTA ACCTGACTAT 10218
Quality scores
AJ489607 consensus AAATCTCAAC CTACTACAAT GCTAAAAATT ATTCTTCCCT CACTAATGCT ACTACCACTA ACCTGACTAT 10218
Quality scores
AJ489607 consensus AAATCTCAAC CTACTACAAT GCTAAAAATT ATTCTTCCCT CACTAATGCT ACTACCACTA ACCTGACTAT 10218
Quality scores
AJ489607 consensus AAATCTCAAC CTACTACAAT GCTAAAAATT ATTCTTCCCT CACTAATGCT ACTACCACTA ACCTGACTAT 10218
Quality scores
AJ489607 consensus AAATCTCAAC CTACTACAAT GCTAAAAATT ATTCTTCCCT CACTAATGCT ACTACCACTA ACCTGACTAT 10218
Quality scores
Consensus AAATCTCAAC CTACTACAAT GCTAAAAATT ATTCTTCCCT CACTAATGCT ACTACCACTA ACCTGACTAT
Conservation
Sequence logo

10,240 10,260 10,280

AJ489607 consensus CAAGCCCTAA AAAAACCTGA ACAAACGTAA CCTCATATAG TTTTCTAATT AGTTTAACCA GCCTAACACT 10288
Quality scores 64 0
AJ489607 consensus CAAGCCCTAA AAAAACCTGA ACAAACGTAA CCTCATATAG TTTTCTAATT AGTTTAACCA GCCTAACACT 10290
Quality scores 64 0
AJ489607 consensus CAAGCCCTAA AAAAACCTGA ACAAACGTAA CCTCATATAG TTTTCTAATT AGTTTAACCA GCCTAACACT 10290
Quality scores 64 0
AJ489607 consensus CAAGCCCTAA AAAAACCTGA ACAAACGTAA CCTCATATAG TTTTCTAATT AGTTTAACCA GCCTAACACT 10290
Quality scores 64 0
AJ489607 consensus CAAGCCCTAA AAAAACCTGA ACAAACGTAA CCTCATATAG TTTTCTAATT AGTTTAACCA GCCTAACACT 10288
Quality scores 64 0
AJ489607 consensus CAAGCCCTAA AAAAACCTGA ACAAACGTAA CCTCATATAG TTTTCTAATT AGTTTAACCA GCCTAACACT 10288
Quality scores 64 0
AJ489607 consensus CAAGCCCTAA AAAAACCTGA ACAAACGTAA CCTCATATAG TTTTCTAATT AGTTTAACCA GCCTAACACT 10288
Quality scores 64 0
AJ489607 consensus CAAGCCCTAA AAAAACCTGA ACAAACGTAA CCTCATATAG TTTTCTAATT AGTTTAACCA GCCTAACACT 10288
Quality scores 64 0
AJ489607 consensus CAAGCCCTAA AAAAACCTGA ACAAACGTAA CCTCATATAG TTTTCTAATT AGTTTAACCA GCCTAACACT 10288
Quality scores 64 0
Consensus CAAGCCCTAA AAAAACCTGA ACAAACGTAA CCTCATATAG TTTTCTAATT AGTTTAACCA GCCTAACACT
Conservation 100% 0%
Sequence logo 2.0bits 0.0bits CAAGCCCTAA AAAAACCTGA ACAAACGTAA CCTCATATAG TTTTCTAATT AGTTTAACCA GCCTAACACT

10,300 10,320 10,340 10,360

AJ489607 consensus TCTATGACAA ACCGACGAAA ATTATAAAAA CTTTTCAAAT ATATTCTCCT CAGACCCCCT ATCCACACCA 10358
Quality scores 64 0
AJ489607 consensus TCTATGACAA ACCGACGAAA ATTATAAAAA CTTTTCAAAT ATATTCTCCT CAGACCCCCT ATCCACACCA 10360
Quality scores 64 0
AJ489607 consensus TCTATGACAA ACCGACGAAA ATTATAAAAA CTTTTCAAAT ATATTCTCCT CAGACCCCCT ATCCACACCA 10360
Quality scores 64 0
AJ489607 consensus TCTATGACAA ACCGACGAAA ATTATAAAAA CTTTTCAAAT ATATTCTCCT CAGACCCCCT ATCCACACCA 10360
Quality scores 64 0
AJ489607 consensus TCTATGACAA ACCGACGAAA ATTATAAAAA CTTTTCAAAT ATATTCTCCT CAGACCCCCT ATCCACACCA 10358
Quality scores 64 0
AJ489607 consensus TCTATGACAA ACCGACGAAA ATTATAAAAA CTTTTCAAAT ATATTCTCCT CAGACCCCCT ATCCACACCA 10358
Quality scores 64 0
AJ489607 consensus TCTATGACAA ACCGACGAAA ATTATAAAAA CTTTTCAAAT ATATTCTCCT CAGACCCCCT ATCCACACCA 10358
Quality scores 64 0
AJ489607 consensus TCTATGACAA ACCGACGAAA ATTATAAAAA CTTTTCAAAT ATATTCTCCT CAGACCCCCT ATCCACACCA 10358
Quality scores 64 0
AJ489607 consensus TCTATGACAA ACCGACGAAA ATTATAAAAA CTTTTCAAAT ATATTCTCCT CAGACCCCCT ATCCACACCA 10358
Quality scores 64 0
Consensus TCTATGACAA ACCGACGAAA ATTATAAAAA CTTTTCAAAT ATATTCTCCT CAGACCCCCT ATCCACACCA
Conservation 100% 0%
Sequence logo 2.0bits 0.0bits TCTATGACAA ACCGACGAAA ATTATAAAAA CTTTTCAAAT ATATTCTCCT CAGACCCCCT ATCCACACCA

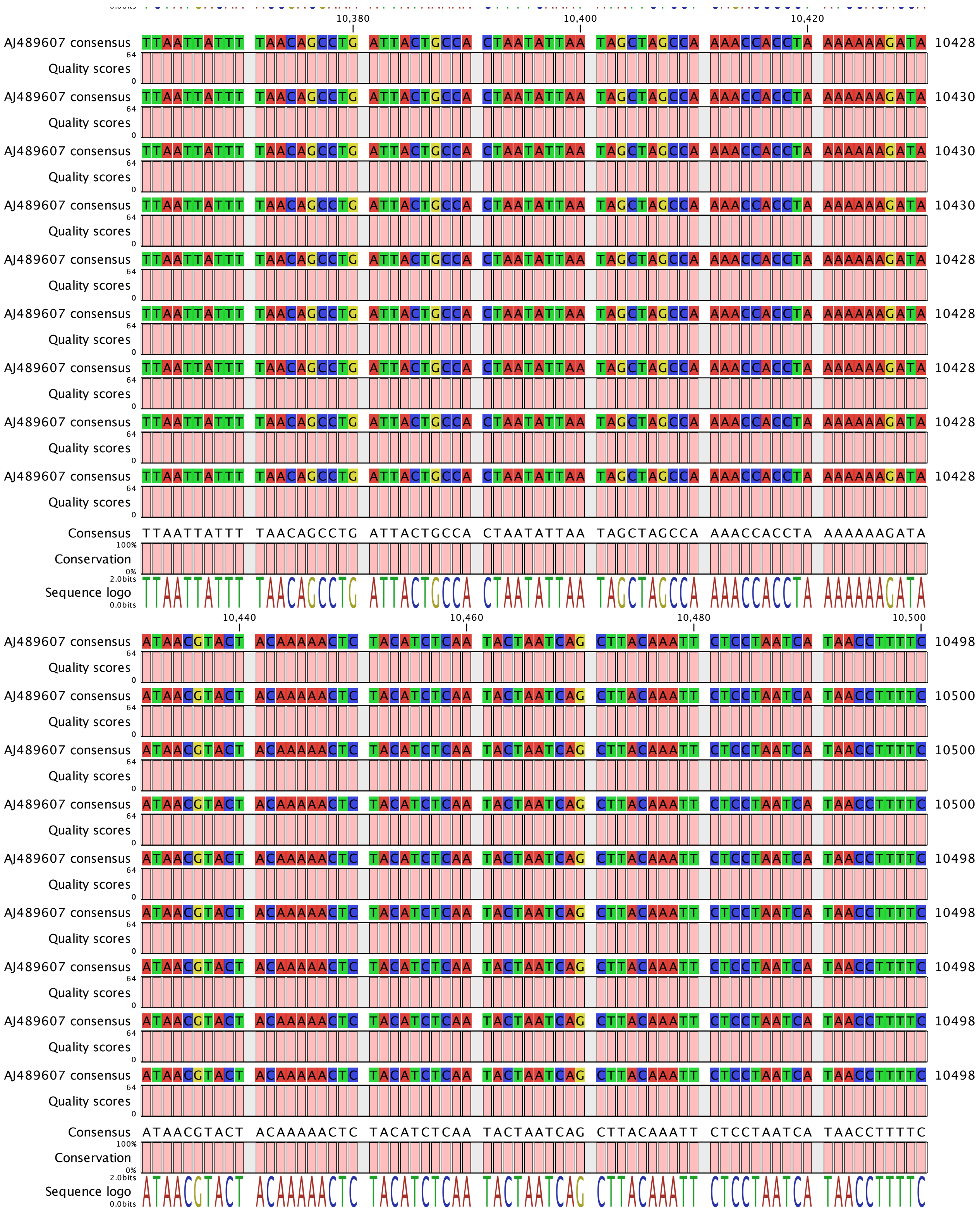
10,380 10,400 10,420
AJ489607 consensus TTAATTATTT TAACAGCCTG ATTACTGCCA CTAATATTAA TAGCTAGCCA AAACCACCTA AAAAAAGATA 10428
Quality scores
AJ489607 consensus TTAATTATTT TAACAGCCTG ATTACTGCCA CTAATATTAA TAGCTAGCCA AAACCACCTA AAAAAAGATA 10430
Quality scores
AJ489607 consensus TTAATTATTT TAACAGCCTG ATTACTGCCA CTAATATTAA TAGCTAGCCA AAACCACCTA AAAAAAGATA 10430
Quality scores
AJ489607 consensus TTAATTATTT TAACAGCCTG ATTACTGCCA CTAATATTAA TAGCTAGCCA AAACCACCTA AAAAAAGATA 10430
Quality scores
AJ489607 consensus TTAATTATTT TAACAGCCTG ATTACTGCCA CTAATATTAA TAGCTAGCCA AAACCACCTA AAAAAAGATA 10428
Quality scores
AJ489607 consensus TTAATTATTT TAACAGCCTG ATTACTGCCA CTAATATTAA TAGCTAGCCA AAACCACCTA AAAAAAGATA 10428
Quality scores
AJ489607 consensus TTAATTATTT TAACAGCCTG ATTACTGCCA CTAATATTAA TAGCTAGCCA AAACCACCTA AAAAAAGATA 10428
Quality scores
AJ489607 consensus TTAATTATTT TAACAGCCTG ATTACTGCCA CTAATATTAA TAGCTAGCCA AAACCACCTA AAAAAAGATA 10428
Quality scores
AJ489607 consensus TTAATTATTT TAACAGCCTG ATTACTGCCA CTAATATTAA TAGCTAGCCA AAACCACCTA AAAAAAGATA 10428
Quality scores
Consensus TTAATTATTT TAACAGCCTG ATTACTGCCA CTAATATTAA TAGCTAGCCA AAACCACCTA AAAAAAGATA
Conservation
Sequence logo
10,440 10,460 10,480 10,500
AJ489607 consensus ATAACGTACT ACAAAAACTC TACATCTCAA TACTAATCAG CTTACAAATT CTCCTAATCA TAACCTTTTC 10498
Quality scores
AJ489607 consensus ATAACGTACT ACAAAAACTC TACATCTCAA TACTAATCAG CTTACAAATT CTCCTAATCA TAACCTTTTC 10500
Quality scores
AJ489607 consensus ATAACGTACT ACAAAAACTC TACATCTCAA TACTAATCAG CTTACAAATT CTCCTAATCA TAACCTTTTC 10500
Quality scores
AJ489607 consensus ATAACGTACT ACAAAAACTC TACATCTCAA TACTAATCAG CTTACAAATT CTCCTAATCA TAACCTTTTC 10500
Quality scores
AJ489607 consensus ATAACGTACT ACAAAAACTC TACATCTCAA TACTAATCAG CTTACAAATT CTCCTAATCA TAACCTTTTC 10498
Quality scores
AJ489607 consensus ATAACGTACT ACAAAAACTC TACATCTCAA TACTAATCAG CTTACAAATT CTCCTAATCA TAACCTTTTC 10498
Quality scores
AJ489607 consensus ATAACGTACT ACAAAAACTC TACATCTCAA TACTAATCAG CTTACAAATT CTCCTAATCA TAACCTTTTC 10498
Quality scores
AJ489607 consensus ATAACGTACT ACAAAAACTC TACATCTCAA TACTAATCAG CTTACAAATT CTCCTAATCA TAACCTTTTC 10498
Quality scores
AJ489607 consensus ATAACGTACT ACAAAAACTC TACATCTCAA TACTAATCAG CTTACAAATT CTCCTAATCA TAACCTTTTC 10498
Quality scores
Consensus ATAACGTACT ACAAAAACTC TACATCTCAA TACTAATCAG CTTACAAATT CTCCTAATCA TAACCTTTTC
Conservation
Sequence logo

0.0bits

10,520 10,540 10,560

AJ489607 consensus AGCAACTGAA CTAATTATAT TTTATATTTT ATTTGAAGCA ACCTTAATCC CAACACTTAT TATTATTACC 10568
64 Quality scores 0
AJ489607 consensus AGCAACTGAA CTAATTATAT TTTATATTTT ATTTGAAGCA ACCTTAATCC CAACACTTAT TATTATTACC 10570
64 Quality scores 0
AJ489607 consensus AGCAACTGAA CTAATTATAT TTTATATTTT ATTTGAAGCA ACCTTAATCC CAACACTTAT TATTATTACC 10570
64 Quality scores 0
AJ489607 consensus AGCAACTGAA CTAATTATAT TTTATATTTT ATTTGAAGCA ACCTTAATCC CAACACTTAT TATTATTACC 10570
64 Quality scores 0
AJ489607 consensus AGCAACTGAA CTAATTATAT TTTATATTTT ATTTGAAGCA ACCTTAATCC CAACACTTAT TATTATTACC 10568
64 Quality scores 0
AJ489607 consensus AGCAACTGAA CTAATTATAT TTTATATTTT ATTTGAAGCA ACCTTAATCC CAACACTTAT TATTATTACC 10568
64 Quality scores 0
AJ489607 consensus AGCAACTGAA CTAATTATAT TTTATATTTT ATTTGAAGCA ACCTTAATCC CAACACTTAT TATTATTACC 10568
64 Quality scores 0
AJ489607 consensus AGCAACTGAA CTAATTATAT TTTATATTTT ATTTGAAGCA ACCTTAATCC CAACACTTAT TATTATTACC 10568
64 Quality scores 0
AJ489607 consensus AGCAACTGAA CTAATTATAT TTTATATTTT ATTTGAAGCA ACCTTAATCC CAACACTTAT TATTATTACC 10568
64 Quality scores 0
Consensus AGCAACTGAA CTAATTATAT TTTATATTTT ATTTGAAGCA ACCTTAATCC CAACACTTAT TATTATTACC
100% Conservation 0%
2.0bits Sequence logo 0.0bits AGCAACTGAA CTAATTATAT TTTATATTTT ATTTGAAGCA ACCTTAATCC CAACACTTAT TATTATTACC

10,580 10,600 10,620 10,640

AJ489607 consensus CGATGAGGGA ACCAAACTGA ACGCCTAAAC GCAGGGATTT ATTTCCTATT TTATACCCTA ATCGGTTCTA 10638
64 Quality scores 0
AJ489607 consensus CGATGAGGGA ACCAAACTGA ACGCCTAAAC GCAGGGATTT ATTTCCTATT TTATACCCTA ATCGGTTCTA 10640
64 Quality scores 0
AJ489607 consensus CGATGAGGGA ACCAAACTGA ACGCCTAAAC GCAGGGATTT ATTTCCTATT TTATACCCTA ATCGGTTCTA 10640
64 Quality scores 0
AJ489607 consensus CGATGAGGGA ACCAAACTGA ACGCCTAAAC GCAGGGATTT ATTTCCTATT TTATACCCTA ATCGGTTCTA 10640
64 Quality scores 0
AJ489607 consensus CGATGAGGGA ACCAAACTGA ACGCCTAAAC GCAGGGATTT ATTTCCTATT TTATACCCTA ATCGGTTCTA 10638
64 Quality scores 0
AJ489607 consensus CGATGAGGGA ACCAAACTGA ACGCCTAAAC GCAGGGATTT ATTTCCTATT TTATACCCTA ATCGGTTCTA 10638
64 Quality scores 0
AJ489607 consensus CGATGAGGGA ACCAAACTGA ACGCCTAAAC GCAGGGATTT ATTTCCTATT TTATACCCTA ATCGGTTCTA 10638
64 Quality scores 0
AJ489607 consensus CGATGAGGGA ACCAAACTGA ACGCCTAAAC GCAGGGATTT ATTTCCTATT TTATACCCTA ATCGGTTCTA 10638
64 Quality scores 0
AJ489607 consensus CGATGAGGGA ACCAAACTGA ACGCCTAAAC GCAGGGATTT ATTTCCTATT TTATACCCTA ATCGGTTCTA 10638
64 Quality scores 0
Consensus CGATGAGGGA ACCAAACTGA ACGCCTAAAC GCAGGGATTT ATTTCCTATT TTATACCCTA ATCGGTTCTA
100% Conservation 0%
2.0bits Sequence logo 0.0bits CGATGAGGGA ACCAAACTGA ACGCCTAAAC GCAGGGATTT ATTTCCTATT TTATACCCTA ATCGGTTCTA

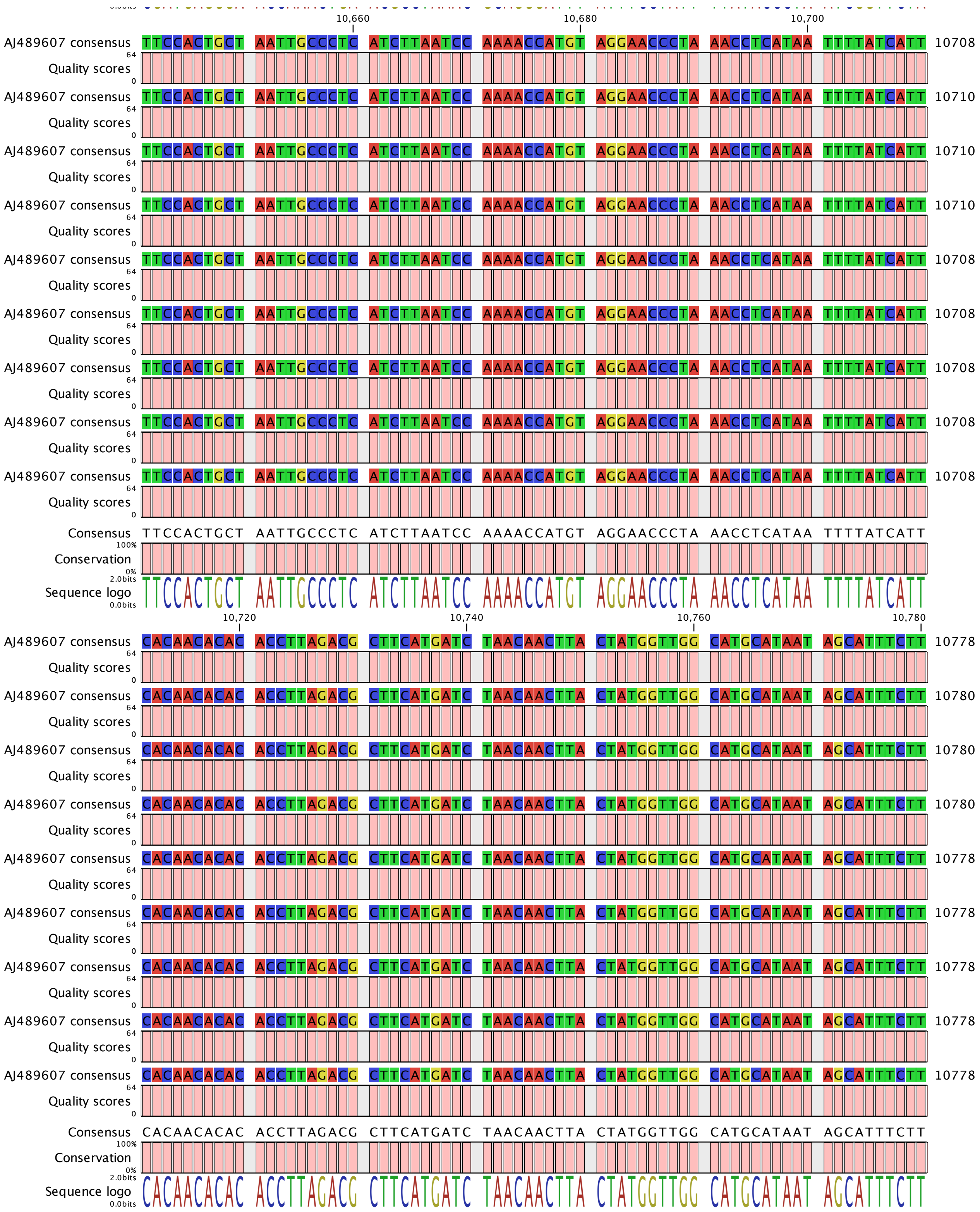
10,660
10,680
10,700
AJ489607 consensus TTCCACTGCT AATTGCCCTC ATCTTAATCC AAAACCATGT AGGAACCCTA AACCTCATAA TTTTATCATT 10708
Quality scores
AJ489607 consensus TTCCACTGCT AATTGCCCTC ATCTTAATCC AAAACCATGT AGGAACCCTA AACCTCATAA TTTTATCATT 10710
Quality scores
AJ489607 consensus TTCCACTGCT AATTGCCCTC ATCTTAATCC AAAACCATGT AGGAACCCTA AACCTCATAA TTTTATCATT 10710
Quality scores
AJ489607 consensus TTCCACTGCT AATTGCCCTC ATCTTAATCC AAAACCATGT AGGAACCCTA AACCTCATAA TTTTATCATT 10710
Quality scores
AJ489607 consensus TTCCACTGCT AATTGCCCTC ATCTTAATCC AAAACCATGT AGGAACCCTA AACCTCATAA TTTTATCATT 10708
Quality scores
AJ489607 consensus TTCCACTGCT AATTGCCCTC ATCTTAATCC AAAACCATGT AGGAACCCTA AACCTCATAA TTTTATCATT 10708
Quality scores
AJ489607 consensus TTCCACTGCT AATTGCCCTC ATCTTAATCC AAAACCATGT AGGAACCCTA AACCTCATAA TTTTATCATT 10708
Quality scores
AJ489607 consensus TTCCACTGCT AATTGCCCTC ATCTTAATCC AAAACCATGT AGGAACCCTA AACCTCATAA TTTTATCATT 10708
Quality scores
AJ489607 consensus TTCCACTGCT AATTGCCCTC ATCTTAATCC AAAACCATGT AGGAACCCTA AACCTCATAA TTTTATCATT 10708
Quality scores
Consensus TTCCACTGCT AATTGCCCTC ATCTTAATCC AAAACCATGT AGGAACCCTA AACCTCATAA TTTTATCATT
Conservation
Sequence logo
10,720
10,740
10,760
10,780
AJ489607 consensus CACAACACAC ACCTTAGACG CTTCATGATC TAACAACTTA CTATGGTTGG CATGCATAAT AGCATTTCTT 10778
Quality scores
AJ489607 consensus CACAACACAC ACCTTAGACG CTTCATGATC TAACAACTTA CTATGGTTGG CATGCATAAT AGCATTTCTT 10780
Quality scores
AJ489607 consensus CACAACACAC ACCTTAGACG CTTCATGATC TAACAACTTA CTATGGTTGG CATGCATAAT AGCATTTCTT 10780
Quality scores
AJ489607 consensus CACAACACAC ACCTTAGACG CTTCATGATC TAACAACTTA CTATGGTTGG CATGCATAAT AGCATTTCTT 10780
Quality scores
AJ489607 consensus CACAACACAC ACCTTAGACG CTTCATGATC TAACAACTTA CTATGGTTGG CATGCATAAT AGCATTTCTT 10778
Quality scores
AJ489607 consensus CACAACACAC ACCTTAGACG CTTCATGATC TAACAACTTA CTATGGTTGG CATGCATAAT AGCATTTCTT 10778
Quality scores
AJ489607 consensus CACAACACAC ACCTTAGACG CTTCATGATC TAACAACTTA CTATGGTTGG CATGCATAAT AGCATTTCTT 10778
Quality scores
AJ489607 consensus CACAACACAC ACCTTAGACG CTTCATGATC TAACAACTTA CTATGGTTGG CATGCATAAT AGCATTTCTT 10778
Quality scores
AJ489607 consensus CACAACACAC ACCTTAGACG CTTCATGATC TAACAACTTA CTATGGTTGG CATGCATAAT AGCATTTCTT 10778
Quality scores
Consensus CACAACACAC ACCTTAGACG CTTCATGATC TAACAACTTA CTATGGTTGG CATGCATAAT AGCATTTCTT
Conservation
Sequence logo

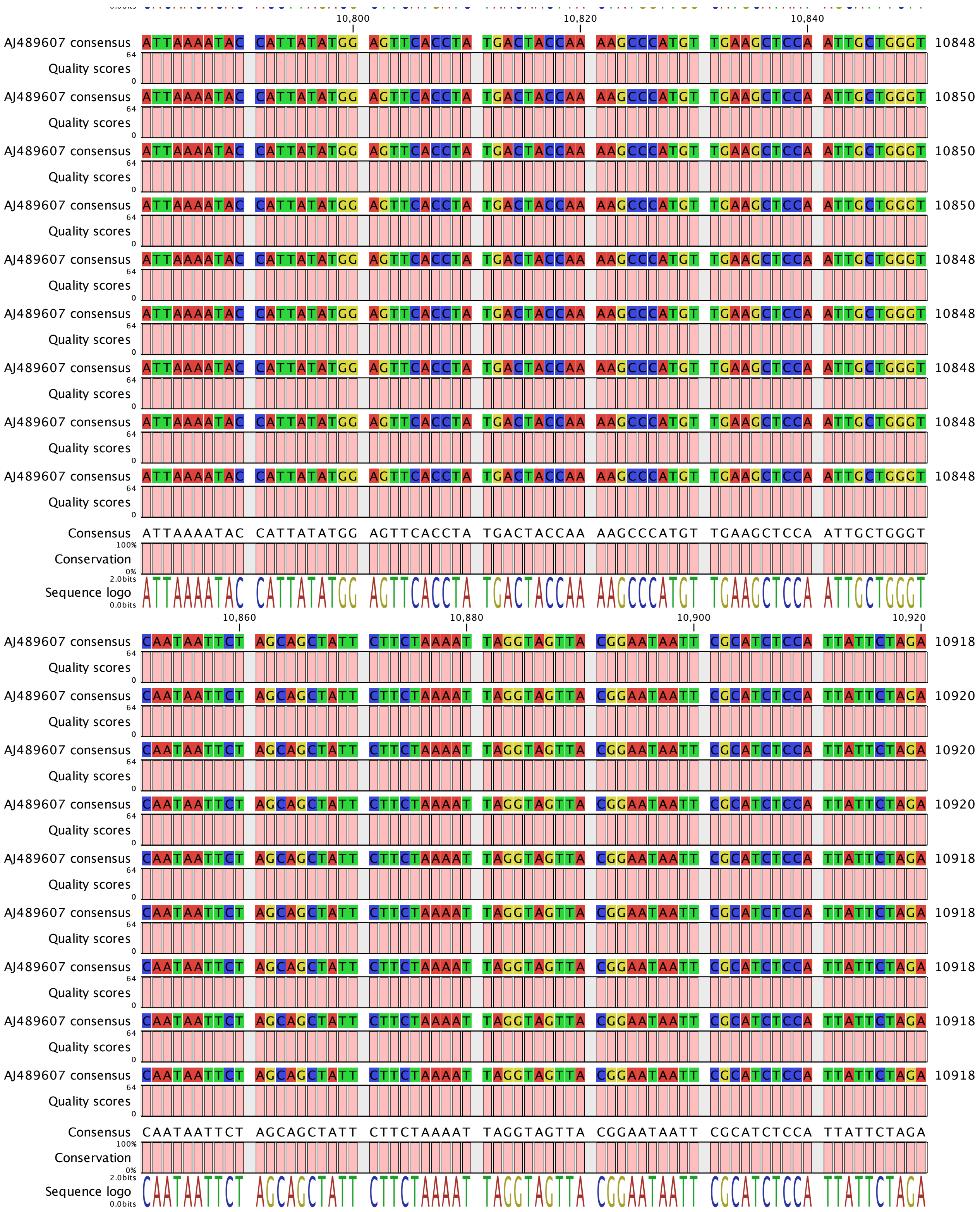

10,800 10,820 10,840
AJ489607 consensus ATTAAAATAC CATTATATGG AGTTCACCTA TGACTACCAA AAGCCCATGT TGAAGCTCCA ATTGCTGGGT 10848
Quality scores
AJ489607 consensus ATTAAAATAC CATTATATGG AGTTCACCTA TGACTACCAA AAGCCCATGT TGAAGCTCCA ATTGCTGGGT 10850
Quality scores
AJ489607 consensus ATTAAAATAC CATTATATGG AGTTCACCTA TGACTACCAA AAGCCCATGT TGAAGCTCCA ATTGCTGGGT 10850
Quality scores
AJ489607 consensus ATTAAAATAC CATTATATGG AGTTCACCTA TGACTACCAA AAGCCCATGT TGAAGCTCCA ATTGCTGGGT 10850
Quality scores
AJ489607 consensus ATTAAAATAC CATTATATGG AGTTCACCTA TGACTACCAA AAGCCCATGT TGAAGCTCCA ATTGCTGGGT 10848
Quality scores
AJ489607 consensus ATTAAAATAC CATTATATGG AGTTCACCTA TGACTACCAA AAGCCCATGT TGAAGCTCCA ATTGCTGGGT 10848
Quality scores
AJ489607 consensus ATTAAAATAC CATTATATGG AGTTCACCTA TGACTACCAA AAGCCCATGT TGAAGCTCCA ATTGCTGGGT 10848
Quality scores
AJ489607 consensus ATTAAAATAC CATTATATGG AGTTCACCTA TGACTACCAA AAGCCCATGT TGAAGCTCCA ATTGCTGGGT 10848
Quality scores
AJ489607 consensus ATTAAAATAC CATTATATGG AGTTCACCTA TGACTACCAA AAGCCCATGT TGAAGCTCCA ATTGCTGGGT 10848
Quality scores
Consensus ATTAAAATAC CATTATATGG AGTTCACCTA TGACTACCAA AAGCCCATGT TGAAGCTCCA ATTGCTGGGT
Conservation
Sequence logo
10,860 10,880 10,900 10,920
AJ489607 consensus CAATAATTCT AGCAGCTATT CTTCTAAAAT TAGGTAGTTA CGGAATAATT CGCATCTCCA TTATTCTAGA 10918
Quality scores
AJ489607 consensus CAATAATTCT AGCAGCTATT CTTCTAAAAT TAGGTAGTTA CGGAATAATT CGCATCTCCA TTATTCTAGA 10920
Quality scores
AJ489607 consensus CAATAATTCT AGCAGCTATT CTTCTAAAAT TAGGTAGTTA CGGAATAATT CGCATCTCCA TTATTCTAGA 10920
Quality scores
AJ489607 consensus CAATAATTCT AGCAGCTATT CTTCTAAAAT TAGGTAGTTA CGGAATAATT CGCATCTCCA TTATTCTAGA 10920
Quality scores
AJ489607 consensus CAATAATTCT AGCAGCTATT CTTCTAAAAT TAGGTAGTTA CGGAATAATT CGCATCTCCA TTATTCTAGA 10918
Quality scores
AJ489607 consensus CAATAATTCT AGCAGCTATT CTTCTAAAAT TAGGTAGTTA CGGAATAATT CGCATCTCCA TTATTCTAGA 10918
Quality scores
AJ489607 consensus CAATAATTCT AGCAGCTATT CTTCTAAAAT TAGGTAGTTA CGGAATAATT CGCATCTCCA TTATTCTAGA 10918
Quality scores
AJ489607 consensus CAATAATTCT AGCAGCTATT CTTCTAAAAT TAGGTAGTTA CGGAATAATT CGCATCTCCA TTATTCTAGA 10918
Quality scores
AJ489607 consensus CAATAATTCT AGCAGCTATT CTTCTAAAAT TAGGTAGTTA CGGAATAATT CGCATCTCCA TTATTCTAGA 10918
Quality scores
Consensus CAATAATTCT AGCAGCTATT CTTCTAAAAT TAGGTAGTTA CGGAATAATT CGCATCTCCA TTATTCTAGA
Conservation
Sequence logo

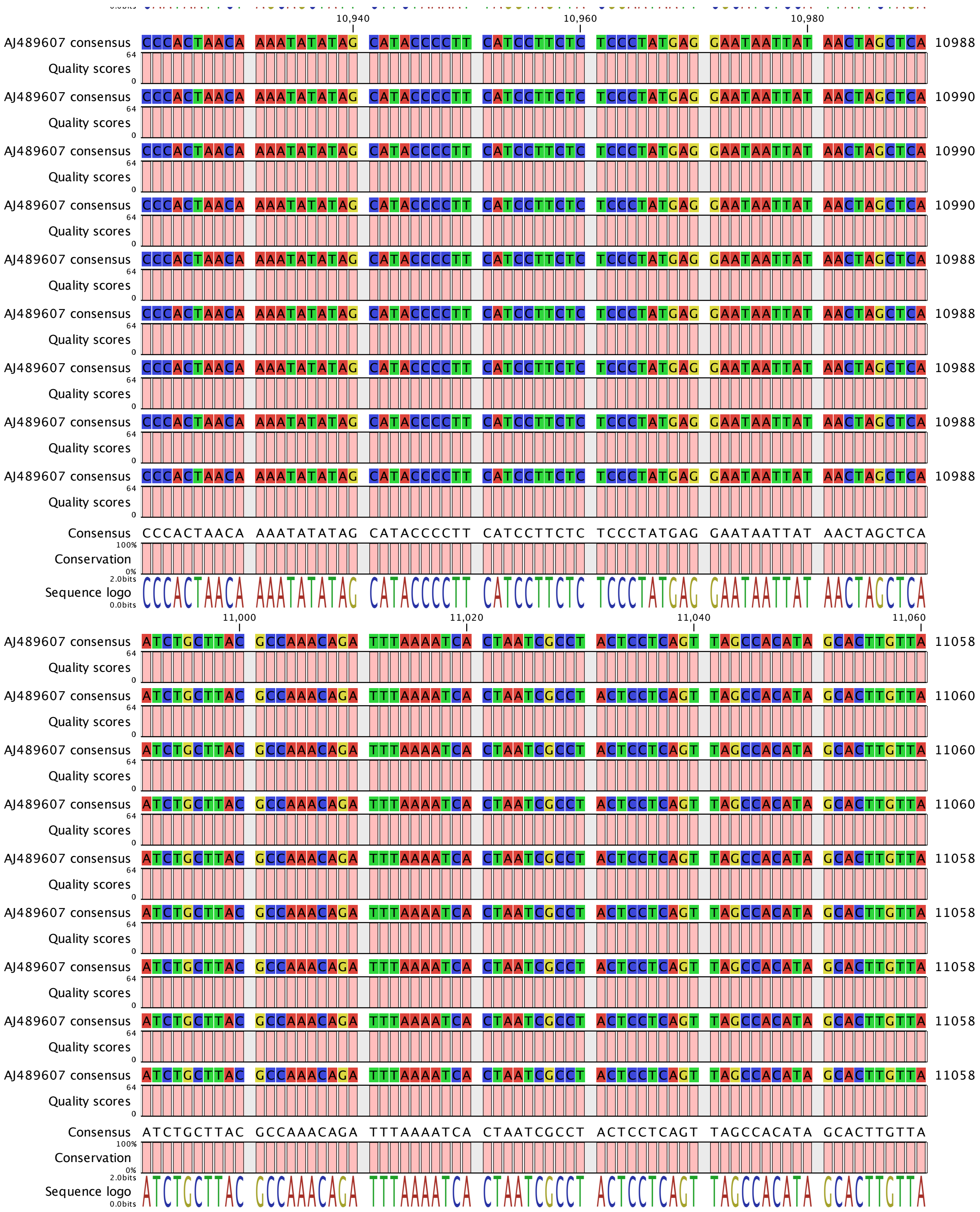
10,940
10,960
10,980
AJ489607 consensus CCCACTAACA AAATATATAG CATACCCCTT CATCCTTCTC TCCCTATGAG GAATAATTAT AACTAGCTCA 10988
Quality scores
AJ489607 consensus CCCACTAACA AAATATATAG CATACCCCTT CATCCTTCTC TCCCTATGAG GAATAATTAT AACTAGCTCA 10990
Quality scores
AJ489607 consensus CCCACTAACA AAATATATAG CATACCCCTT CATCCTTCTC TCCCTATGAG GAATAATTAT AACTAGCTCA 10990
Quality scores
AJ489607 consensus CCCACTAACA AAATATATAG CATACCCCTT CATCCTTCTC TCCCTATGAG GAATAATTAT AACTAGCTCA 10990
Quality scores
AJ489607 consensus CCCACTAACA AAATATATAG CATACCCCTT CATCCTTCTC TCCCTATGAG GAATAATTAT AACTAGCTCA 10988
Quality scores
AJ489607 consensus CCCACTAACA AAATATATAG CATACCCCTT CATCCTTCTC TCCCTATGAG GAATAATTAT AACTAGCTCA 10988
Quality scores
AJ489607 consensus CCCACTAACA AAATATATAG CATACCCCTT CATCCTTCTC TCCCTATGAG GAATAATTAT AACTAGCTCA 10988
Quality scores
AJ489607 consensus CCCACTAACA AAATATATAG CATACCCCTT CATCCTTCTC TCCCTATGAG GAATAATTAT AACTAGCTCA 10988
Quality scores
AJ489607 consensus CCCACTAACA AAATATATAG CATACCCCTT CATCCTTCTC TCCCTATGAG GAATAATTAT AACTAGCTCA 10988
Quality scores
Consensus CCCACTAACA AAATATATAG CATACCCCTT CATCCTTCTC TCCCTATGAG GAATAATTAT AACTAGCTCA
Conservation
Sequence logo CCCACTAACA AAATATATAG CATACCCCTT CATCCTTCTC TCCCTATGAG GAATAATTAT AACTAGCTCA
11,000
11,020
11,040
11,060
AJ489607 consensus ATCTGCTTAC GCCAAACAGA TTTAAAATCA CTAATCGCCT ACTCCTCAGT TAGCCACATA GCACTTGTTA 11058
Quality scores
AJ489607 consensus ATCTGCTTAC GCCAAACAGA TTTAAAATCA CTAATCGCCT ACTCCTCAGT TAGCCACATA GCACTTGTTA 11060
Quality scores
AJ489607 consensus ATCTGCTTAC GCCAAACAGA TTTAAAATCA CTAATCGCCT ACTCCTCAGT TAGCCACATA GCACTTGTTA 11060
Quality scores
AJ489607 consensus ATCTGCTTAC GCCAAACAGA TTTAAAATCA CTAATCGCCT ACTCCTCAGT TAGCCACATA GCACTTGTTA 11060
Quality scores
AJ489607 consensus ATCTGCTTAC GCCAAACAGA TTTAAAATCA CTAATCGCCT ACTCCTCAGT TAGCCACATA GCACTTGTTA 11058
Quality scores
AJ489607 consensus ATCTGCTTAC GCCAAACAGA TTTAAAATCA CTAATCGCCT ACTCCTCAGT TAGCCACATA GCACTTGTTA 11058
Quality scores
AJ489607 consensus ATCTGCTTAC GCCAAACAGA TTTAAAATCA CTAATCGCCT ACTCCTCAGT TAGCCACATA GCACTTGTTA 11058
Quality scores
AJ489607 consensus ATCTGCTTAC GCCAAACAGA TTTAAAATCA CTAATCGCCT ACTCCTCAGT TAGCCACATA GCACTTGTTA 11058
Quality scores
AJ489607 consensus ATCTGCTTAC GCCAAACAGA TTTAAAATCA CTAATCGCCT ACTCCTCAGT TAGCCACATA GCACTTGTTA 11058
Quality scores
Consensus ATCTGCTTAC GCCAAACAGA TTTAAAATCA CTAATCGCCT ACTCCTCAGT TAGCCACATA GCACTTGTTA
Conservation
Sequence logo ATCTGCTTAC GCCAAACAGA TTTAAAATCA CTAATCGCCT ACTCCTCAGT TAGCCACATA GCACTTGTTA

11,080 11,100 11,120

AJ489607 consensus TTGCATCAAT CATAATCCAA ACTCCATGAA GCTTCATAGG AGCAACAATA CTAATAATCG CACATGGCCT 11128
64 Quality scores 0
AJ489607 consensus TTGCATCAAT CATAATCCAA ACTCCATGAA GCTTCATAGG AGCAACAATA CTAATAATCG CACATGGCCT 11130
64 Quality scores 0
AJ489607 consensus TTGCATCAAT CATAATCCAA ACTCCATGAA GCTTCATAGG AGCAACAATA CTAATAATCG CACATGGCCT 11130
64 Quality scores 0
AJ489607 consensus TTGCATCAAT CATAATCCAA ACTCCATGAA GCTTCATAGG AGCAACAATA CTAATAATCG CACATGGCCT 11130
64 Quality scores 0
AJ489607 consensus TTGCATCAAT CATAATCCAA ACTCCATGAA GCTTCATAGG AGCAACAATA CTAATAATCG CACATGGCCT 11128
64 Quality scores 0
AJ489607 consensus TTGCATCAAT CATAATCCAA ACTCCATGAA GCTTCATAGG AGCAACAATA CTAATAATCG CACATGGCCT 11128
64 Quality scores 0
AJ489607 consensus TTGCATCAAT CATAATCCAA ACTCCATGAA GCTTCATAGG AGCAACAATA CTAATAATCG CACATGGCCT 11128
64 Quality scores 0
AJ489607 consensus TTGCATCAAT CATAATCCAA ACTCCATGAA GCTTCATAGG AGCAACAATA CTAATAATCG CACATGGCCT 11128
64 Quality scores 0
AJ489607 consensus TTGCATCAAT CATAATCCAA ACTCCATGAA GCTTCATAGG AGCAACAATA CTAATAATCG CACATGGCCT 11128
64 Quality scores 0
Consensus TTGCATCAAT CATAATCCAA ACTCCATGAA GCTTCATAGG AGCAACAATA CTAATAATCG CACATGGCCT
100% Conservation 0%
2.0bits Sequence logo 0.0bits TTGCATCAAT CATAATCCAA ACTCCATGAA GCTTCATAGG AGCAACAATA CTAATAATCG CACATGGCCT

11,140 11,160 11,180 11,200

AJ489607 consensus CACATCATCA CTCCTATTCT GCCTAGCAAA CTCCAACTAC GAACGGATCC ACAGCCGTAC TATAATCATG 11198
64 Quality scores 0
AJ489607 consensus CACATCATCA CTCCTATTCT GCCTAGCAAA CTCCAACTAC GAACGGATCC ACAGCCGTAC TATAATCATG 11200
64 Quality scores 0
AJ489607 consensus CACATCATCA CTCCTATTCT GCCTAGCAAA CTCCAACTAC GAACGGATCC ACAGCCGTAC TATAATCATG 11200
64 Quality scores 0
AJ489607 consensus CACATCATCA CTCCTATTCT GCCTAGCAAA CTCCAACTAC GAACGGATCC ACAGCCGTAC TATAATCATG 11200
64 Quality scores 0
AJ489607 consensus CACATCATCA CTCCTATTCT GCCTAGCAAA CTCCAACTAC GAACGGATCC ACAGCCGTAC TATAATCATG 11198
64 Quality scores 0
AJ489607 consensus CACATCATCA CTCCTATTCT GCCTAGCAAA CTCCAACTAC GAACGGATCC ACAGCCGTAC TATAATCATG 11198
64 Quality scores 0
AJ489607 consensus CACATCATCA CTCCTATTCT GCCTAGCAAA CTCCAACTAC GAACGGATCC ACAGCCGTAC TATAATCATG 11198
64 Quality scores 0
AJ489607 consensus CACATCATCA CTCCTATTCT GCCTAGCAAA CTCCAACTAC GAACGGATCC ACAGCCGTAC TATAATCATG 11198
64 Quality scores 0
AJ489607 consensus CACATCATCA CTCCTATTCT GCCTAGCAAA CTCCAACTAC GAACGGATCC ACAGCCGTAC TATAATCATG 11198
64 Quality scores 0
Consensus CACATCATCA CTCCTATTCT GCCTAGCAAA CTCCAACTAC GAACGGATCC ACAGCCGTAC TATAATCATG
100% Conservation 0%
2.0bits Sequence logo 0.0bits CACATCATCA CTCCTATTCT GCCTAGCAAA CTCCAACTAC GAACGGATCC ACAGCCGTAC TATAATCATG

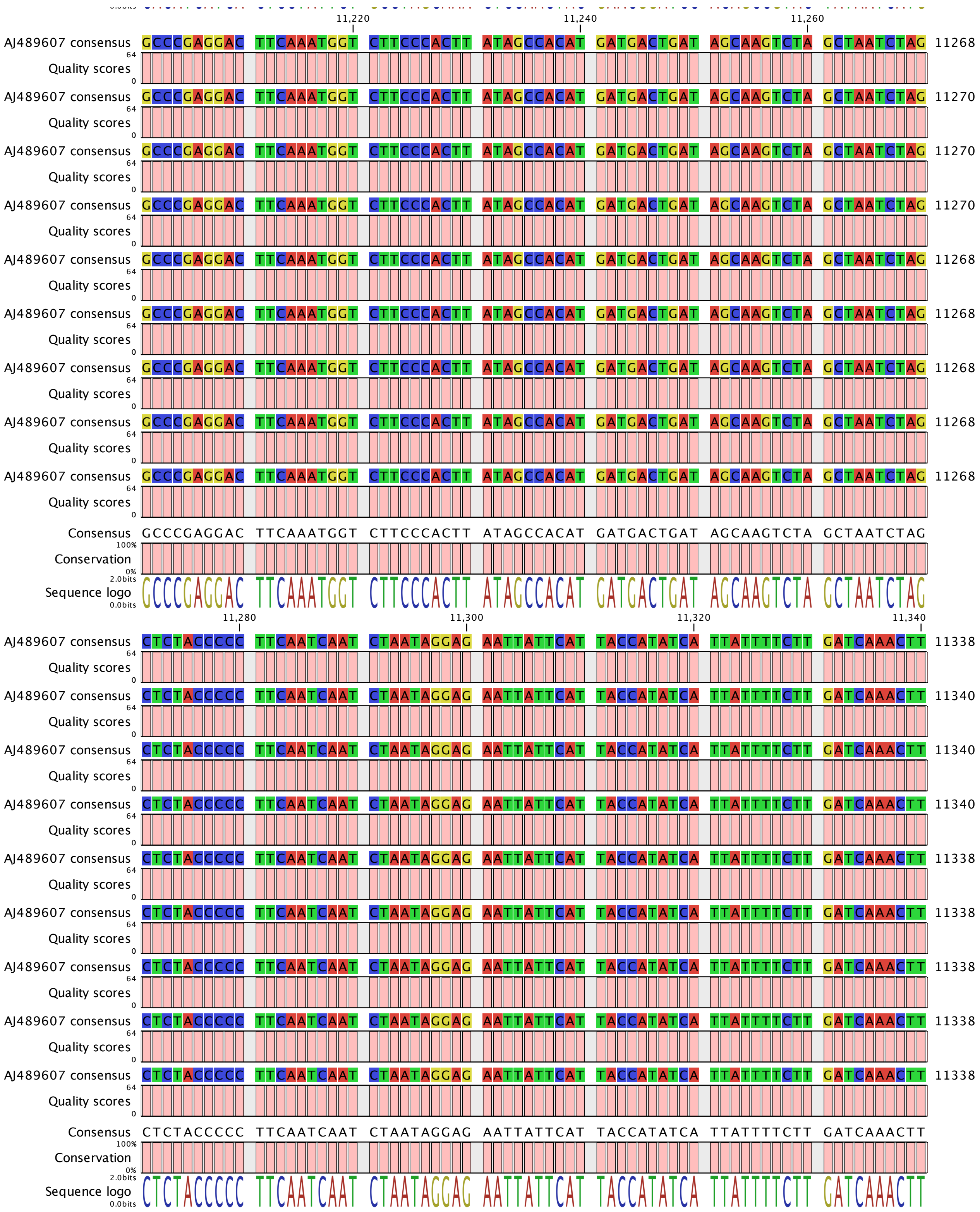
11,220
11,240
11,260
AJ489607 consensus GCCCGAGGAC TTCAAATGGT CTTCCCACTT ATAGCCACAT GATGACTGAT AGCAAGTCTA GCTAATCTAG 11268
Quality scores
AJ489607 consensus GCCCGAGGAC TTCAAATGGT CTTCCCACTT ATAGCCACAT GATGACTGAT AGCAAGTCTA GCTAATCTAG 11270
Quality scores
AJ489607 consensus GCCCGAGGAC TTCAAATGGT CTTCCCACTT ATAGCCACAT GATGACTGAT AGCAAGTCTA GCTAATCTAG 11270
Quality scores
AJ489607 consensus GCCCGAGGAC TTCAAATGGT CTTCCCACTT ATAGCCACAT GATGACTGAT AGCAAGTCTA GCTAATCTAG 11270
Quality scores
AJ489607 consensus GCCCGAGGAC TTCAAATGGT CTTCCCACTT ATAGCCACAT GATGACTGAT AGCAAGTCTA GCTAATCTAG 11268
Quality scores
AJ489607 consensus GCCCGAGGAC TTCAAATGGT CTTCCCACTT ATAGCCACAT GATGACTGAT AGCAAGTCTA GCTAATCTAG 11268
Quality scores
AJ489607 consensus GCCCGAGGAC TTCAAATGGT CTTCCCACTT ATAGCCACAT GATGACTGAT AGCAAGTCTA GCTAATCTAG 11268
Quality scores
AJ489607 consensus GCCCGAGGAC TTCAAATGGT CTTCCCACTT ATAGCCACAT GATGACTGAT AGCAAGTCTA GCTAATCTAG 11268
Quality scores
AJ489607 consensus GCCCGAGGAC TTCAAATGGT CTTCCCACTT ATAGCCACAT GATGACTGAT AGCAAGTCTA GCTAATCTAG 11268
Quality scores
Consensus GCCCGAGGAC TTCAAATGGT CTTCCCACTT ATAGCCACAT GATGACTGAT AGCAAGTCTA GCTAATCTAG
Conservation
Sequence logo
11,280
11,300
11,320
11,340
AJ489607 consensus CTCTACCCCC TTCAATCAAT CTAATAGGAG AATTATTCAT TACCATATCA TTATTTTCTT GATCAAACTT 11338
Quality scores
AJ489607 consensus CTCTACCCCC TTCAATCAAT CTAATAGGAG AATTATTCAT TACCATATCA TTATTTTCTT GATCAAACTT 11340
Quality scores
AJ489607 consensus CTCTACCCCC TTCAATCAAT CTAATAGGAG AATTATTCAT TACCATATCA TTATTTTCTT GATCAAACTT 11340
Quality scores
AJ489607 consensus CTCTACCCCC TTCAATCAAT CTAATAGGAG AATTATTCAT TACCATATCA TTATTTTCTT GATCAAACTT 11340
Quality scores
AJ489607 consensus CTCTACCCCC TTCAATCAAT CTAATAGGAG AATTATTCAT TACCATATCA TTATTTTCTT GATCAAACTT 11338
Quality scores
AJ489607 consensus CTCTACCCCC TTCAATCAAT CTAATAGGAG AATTATTCAT TACCATATCA TTATTTTCTT GATCAAACTT 11338
Quality scores
AJ489607 consensus CTCTACCCCC TTCAATCAAT CTAATAGGAG AATTATTCAT TACCATATCA TTATTTTCTT GATCAAACTT 11338
Quality scores
AJ489607 consensus CTCTACCCCC TTCAATCAAT CTAATAGGAG AATTATTCAT TACCATATCA TTATTTTCTT GATCAAACTT 11338
Quality scores
AJ489607 consensus CTCTACCCCC TTCAATCAAT CTAATAGGAG AATTATTCAT TACCATATCA TTATTTTCTT GATCAAACTT 11338
Quality scores
Consensus CTCTACCCCC TTCAATCAAT CTAATAGGAG AATTATTCAT TACCATATCA TTATTTTCTT GATCAAACTT
Conservation
Sequence logo

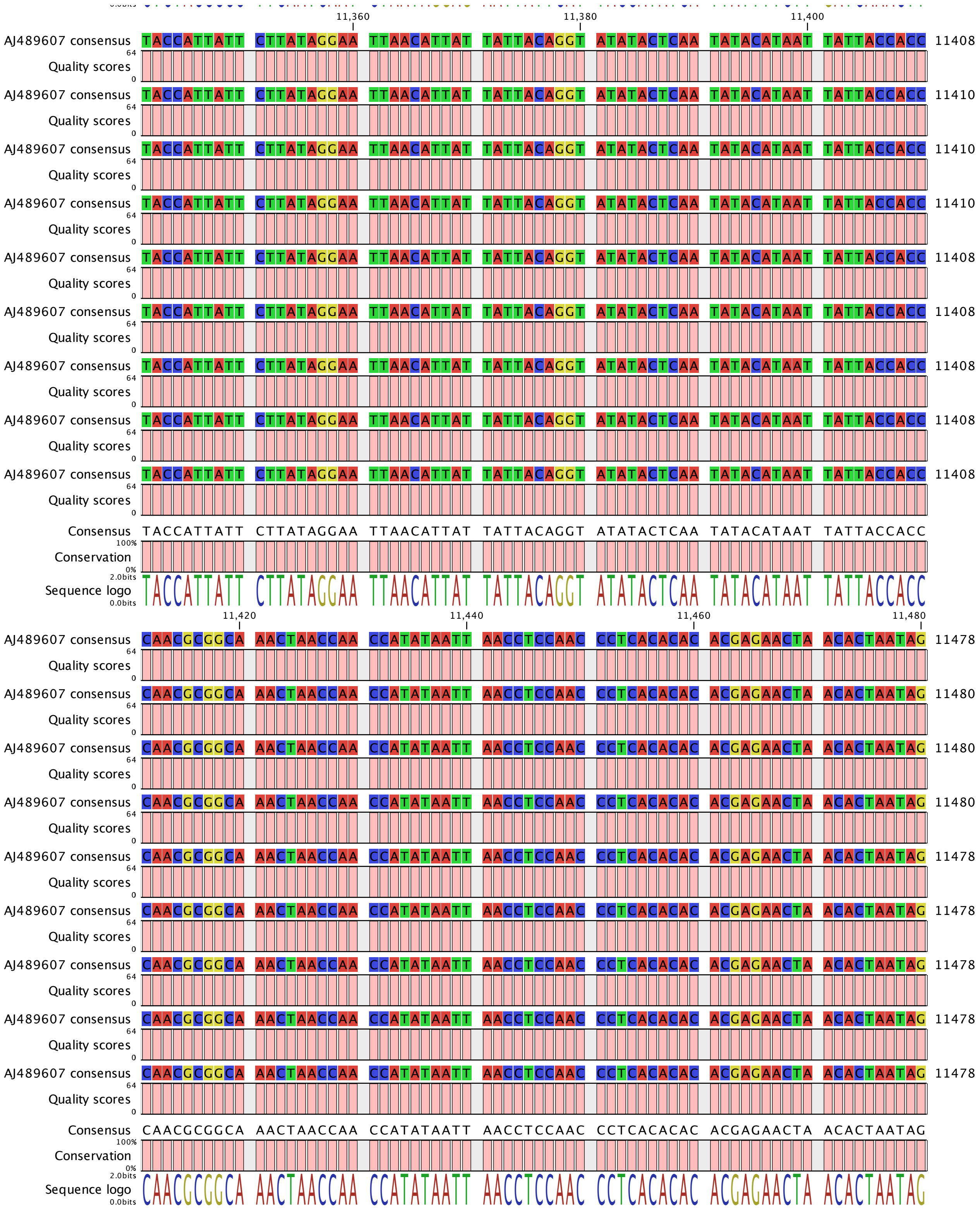

11,360
11,380
11,400
AJ489607 consensus TACCATTATT CTTATAGGAA TTAACATTAT TATTACAGGT ATATACTCAA TATACATAAT TATTACCACC 11408
Quality scores
AJ489607 consensus TACCATTATT CTTATAGGAA TTAACATTAT TATTACAGGT ATATACTCAA TATACATAAT TATTACCACC 11410
Quality scores
AJ489607 consensus TACCATTATT CTTATAGGAA TTAACATTAT TATTACAGGT ATATACTCAA TATACATAAT TATTACCACC 11410
Quality scores
AJ489607 consensus TACCATTATT CTTATAGGAA TTAACATTAT TATTACAGGT ATATACTCAA TATACATAAT TATTACCACC 11410
Quality scores
AJ489607 consensus TACCATTATT CTTATAGGAA TTAACATTAT TATTACAGGT ATATACTCAA TATACATAAT TATTACCACC 11408
Quality scores
AJ489607 consensus TACCATTATT CTTATAGGAA TTAACATTAT TATTACAGGT ATATACTCAA TATACATAAT TATTACCACC 11408
Quality scores
AJ489607 consensus TACCATTATT CTTATAGGAA TTAACATTAT TATTACAGGT ATATACTCAA TATACATAAT TATTACCACC 11408
Quality scores
AJ489607 consensus TACCATTATT CTTATAGGAA TTAACATTAT TATTACAGGT ATATACTCAA TATACATAAT TATTACCACC 11408
Quality scores
AJ489607 consensus TACCATTATT CTTATAGGAA TTAACATTAT TATTACAGGT ATATACTCAA TATACATAAT TATTACCACC 11408
Quality scores
Consensus TACCATTATT CTTATAGGAA TTAACATTAT TATTACAGGT ATATACTCAA TATACATAAT TATTACCACC
Conservation
Sequence logo
11,420
11,440
11,460
11,480
AJ489607 consensus CAACGCGGCA AACTAACCAA CCATATAATT AACCTCCAAC CCTCACACAC ACGAGAACTA ACACTAATAG 11478
Quality scores
AJ489607 consensus CAACGCGGCA AACTAACCAA CCATATAATT AACCTCCAAC CCTCACACAC ACGAGAACTA ACACTAATAG 11480
Quality scores
AJ489607 consensus CAACGCGGCA AACTAACCAA CCATATAATT AACCTCCAAC CCTCACACAC ACGAGAACTA ACACTAATAG 11480
Quality scores
AJ489607 consensus CAACGCGGCA AACTAACCAA CCATATAATT AACCTCCAAC CCTCACACAC ACGAGAACTA ACACTAATAG 11480
Quality scores
AJ489607 consensus CAACGCGGCA AACTAACCAA CCATATAATT AACCTCCAAC CCTCACACAC ACGAGAACTA ACACTAATAG 11478
Quality scores
AJ489607 consensus CAACGCGGCA AACTAACCAA CCATATAATT AACCTCCAAC CCTCACACAC ACGAGAACTA ACACTAATAG 11478
Quality scores
AJ489607 consensus CAACGCGGCA AACTAACCAA CCATATAATT AACCTCCAAC CCTCACACAC ACGAGAACTA ACACTAATAG 11478
Quality scores
AJ489607 consensus CAACGCGGCA AACTAACCAA CCATATAATT AACCTCCAAC CCTCACACAC ACGAGAACTA ACACTAATAG 11478
Quality scores
AJ489607 consensus CAACGCGGCA AACTAACCAA CCATATAATT AACCTCCAAC CCTCACACAC ACGAGAACTA ACACTAATAG 11478
Quality scores
Consensus CAACGCGGCA AACTAACCAA CCATATAATT AACCTCCAAC CCTCACACAC ACGAGAACTA ACACTAATAG
Conservation
Sequence logo

11,500 11,520 11,540

AJ489607 consensus CCCTTCACAT AATTCCACTT ATTCTTCTAA CTACCAGTCC AAAACTAATT ACAGGCCTGA CAATATGTGA 11548
Quality scores
AJ489607 consensus CCCTTCACAT AATTCCACTT ATTCTTCTAA CTACCAGTCC AAAACTAATT ACAGGCCTGA CAATATGTGA 11550
Quality scores
AJ489607 consensus CCCTTCACAT AATTCCACTT ATTCTTCTAA CTACCAGTCC AAAACTAATT ACAGGCCTGA CAATATGTGA 11550
Quality scores
AJ489607 consensus CCCTTCACAT AATTCCACTT ATTCTTCTAA CTACCAGTCC AAAACTAATT ACAGGCCTGA CAATATGTGA 11550
Quality scores
AJ489607 consensus CCCTTCACAT AATTCCACTT ATTCTTCTAA CTACCAGTCC AAAACTAATT ACAGGCCTGA CAATATGTGA 11548
Quality scores
AJ489607 consensus CCCTTCACAT AATTCCACTT ATTCTTCTAA CTACCAGTCC AAAACTAATT ACAGGCCTGA CAATATGTGA 11548
Quality scores
AJ489607 consensus CCCTTCACAT AATTCCACTT ATTCTTCTAA CTACCAGTCC AAAACTAATT ACAGGCCTGA CAATATGTGA 11548
Quality scores
AJ489607 consensus CCCTTCACAT AATTCCACTT ATTCTTCTAA CTACCAGTCC AAAACTAATT ACAGGCCTGA CAATATGTGA 11548
Quality scores
AJ489607 consensus CCCTTCACAT AATTCCACTT ATTCTTCTAA CTACCAGTCC AAAACTAATT ACAGGCCTGA CAATATGTGA 11548
Quality scores
Consensus CCCTTCACAT AATTCCACTT ATTCTTCTAA CTACCAGTCC AAAACTAATT ACAGGCCTGA CAATATGTGA
Conservation
Sequence logo CCCTTCACAT AATTCCACTT ATTCTTCTAA CTACCAGTCC AAAACTAATT ACAGGCCTGA CAATATGTGA

11,560 11,580 11,600 11,620

AJ489607 consensus ATATAGTTTA CAAAAAACAT TAGACTGTGA ATCTGACAAC AGGAAATAAA CCTCCTTATT CACCAAGAAA 11618
Quality scores
AJ489607 consensus ATATAGTTTA CAAAAAACAT TAGACTGTGA ATCTGACAAC AGGAAATAAA CCTCCTTATT CACCAAGAAA 11620
Quality scores
AJ489607 consensus ATATAGTTTA CAAAAAACAT TAGACTGTGA ATCTGACAAC AGGAAATAAA CCTCCTTATT CACCAAGAAA 11620
Quality scores
AJ489607 consensus ATATAGTTTA CAAAAAACAT TAGACTGTGA ATCTGACAAC AGGAAATAAA CCTCCTTATT CACCAAGAAA 11620
Quality scores
AJ489607 consensus ATATAGTTTA CAAAAAACAT TAGACTGTGA ATCTGACAAC AGGAAATAAA CCTCCTTATT CACCAAGAAA 11618
Quality scores
AJ489607 consensus ATATAGTTTA CAAAAAACAT TAGACTGTGA ATCTGACAAC AGGAAATAAA CCTCCTTATT CACCAAGAAA 11618
Quality scores
AJ489607 consensus ATATAGTTTA CAAAAAACAT TAGACTGTGA ATCTGACAAC AGGAAATAAA CCTCCTTATT CACCAAGAAA 11618
Quality scores
AJ489607 consensus ATATAGTTTA CAAAAAACAT TAGACTGTGA ATCTGACAAC AGGAAATAAA CCTCCTTATT CACCAAGAAA 11618
Quality scores
AJ489607 consensus ATATAGTTTA CAAAAAACAT TAGACTGTGA ATCTGACAAC AGGAAATAAA CCTCCTTATT CACCAAGAAA 11618
Quality scores
Consensus ATATAGTTTA CAAAAAACAT TAGACTGTGA ATCTGACAAC AGGAAATAAA CCTCCTTATT CACCAAGAAA
Conservation
Sequence logo ATATAGTTTA CAAAAAACAT TAGACTGTGA ATCTGACAAC AGGAAATAAA CCTCCTTATT CACCAAGAAA

11,640 11,660 11,680

AJ489607 consensus GATTGCAAGA ACTGCTAATT CATGCTTCCA TGTTTAAAAA CATGGCTTTC TTACTTTTAT AGGATAATAG 11688
64
Quality scores
0
AJ489607 consensus GATTGCAAGA ACTGCTAATT CATGCTTCCA TGTTTAAAAA CATGGCTTTC TTACTTTTAT AGGATAATAG 11690
64
Quality scores
0
AJ489607 consensus GATTGCAAGA ACTGCTAATT CATGCTTCCA TGTTTAAAAA CATGGCTTTC TTACTTTTAT AGGATAATAG 11690
64
Quality scores
0
AJ489607 consensus GATTGCAAGA ACTGCTAATT CATGCTTCCA TGTTTAAAAA CATGGCTTTC TTACTTTTAT AGGATAATAG 11690
64
Quality scores
0
AJ489607 consensus GATTGCAAGA ACTGCTAATT CATGCTTCCA TGTTTAAAAA CATGGCTTTC TTACTTTTAT AGGATAATAG 11688
64
Quality scores
0
AJ489607 consensus GATTGCAAGA ACTGCTAATT CATGCTTCCA TGTTTAAAAA CATGGCTTTC TTACTTTTAT AGGATAATAG 11688
64
Quality scores
0
AJ489607 consensus GATTGCAAGA ACTGCTAATT CATGCTTCCA TGTTTAAAAA CATGGCTTTC TTACTTTTAT AGGATAATAG 11688
64
Quality scores
0
AJ489607 consensus GATTGCAAGA ACTGCTAATT CATGCTTCCA TGTTTAAAAA CATGGCTTTC TTACTTTTAT AGGATAATAG 11688
64
Quality scores
0
AJ489607 consensus GATTGCAAGA ACTGCTAATT CATGCTTCCA TGTTTAAAAA CATGGCTTTC TTACTTTTAT AGGATAATAG 11688
64
Quality scores
0
Consensus GATTGCAAGA ACTGCTAATT CATGCTTCCA TGTTTAAAAA CATGGCTTTC TTACTTTTAT AGGATAATAG
100%
Conservation
0%
2.0bits
Sequence logo GATTGCAAGA ACTGCTAATT CATGCTTCCA TGTTTAAAAA CATGGCTTTC TTACTTTTAT AGGATAATAG
0.0bits

11,700 11,720 11,740 11,760

AJ489607 consensus TAATCCATTG GTCTTAGGAA CCAAAAACCT TGGTGCAAAT CCAAATAAAA GTAATCAATA TTTTCACAAC 11758
64
Quality scores
0
AJ489607 consensus TAATCCATTG GTCTTAGGAA CCAAAAACCT TGGTGCAAAT CCAAATAAAA GTAATCAATA TTTTCACAAC 11760
64
Quality scores
0
AJ489607 consensus TAATCCATTG GTCTTAGGAA CCAAAAACCT TGGTGCAAAT CCAAATAAAA GTAATCAATA TTTTCACAAC 11760
64
Quality scores
0
AJ489607 consensus TAATCCATTG GTCTTAGGAA CCAAAAACCT TGGTGCAAAT CCAAATAAAA GTAATCAATA TTTTCACAAC 11760
64
Quality scores
0
AJ489607 consensus TAATCCATTG GTCTTAGGAA CCAAAAACCT TGGTGCAAAT CCAAATAAAA GTAATCAATA TTTTCACAAC 11758
64
Quality scores
0
AJ489607 consensus TAATCCATTG GTCTTAGGAA CCAAAAACCT TGGTGCAAAT CCAAATAAAA GTAATCAATA TTTTCACAAC 11758
64
Quality scores
0
AJ489607 consensus TAATCCATTG GTCTTAGGAA CCAAAAACCT TGGTGCAAAT CCAAATAAAA GTAATCAATA TTTTCACAAC 11758
64
Quality scores
0
AJ489607 consensus TAATCCATTG GTCTTAGGAA CCAAAAACCT TGGTGCAAAT CCAAATAAAA GTAATCAATA TTTTCACAAC 11758
64
Quality scores
0
AJ489607 consensus TAATCCATTG GTCTTAGGAA CCAAAAACCT TGGTGCAAAT CCAAATAAAA GTAATCAATA TTTTCACAAC 11758
64
Quality scores
0
Consensus TAATCCATTG GTCTTAGGAA CCAAAAACCT TGGTGCAAAT CCAAATAAAA GTAATCAATA TTTTCACAAC
100%
Conservation
0%
2.0bits
Sequence logo TAATCCATTG GTCTTAGGAA CCAAAAACCT TGGTGCAAAT CCAAATAAAA GTAATCAATA TTTTCACAAC
0.0bits

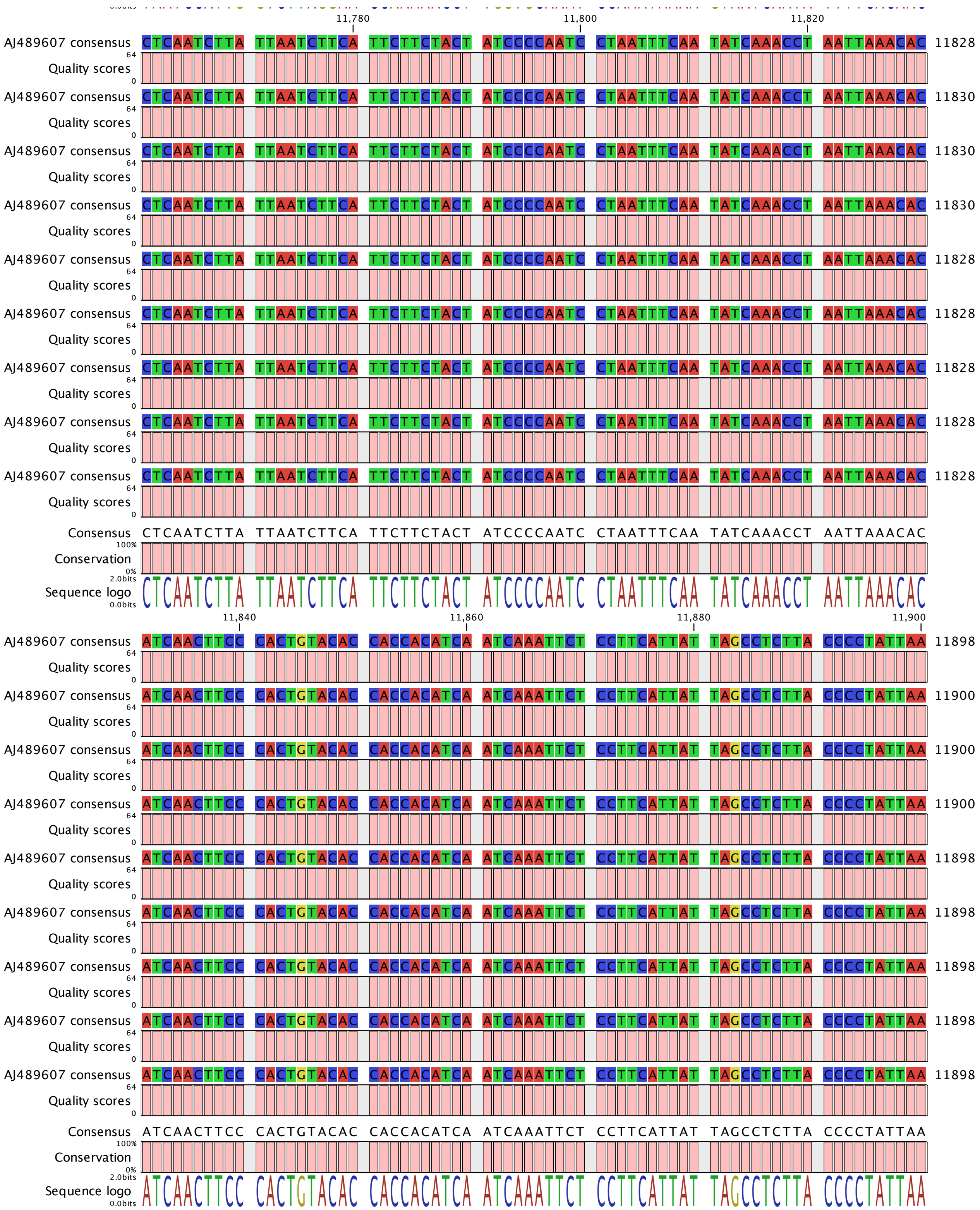
11,780
11,800
11,820
AJ489607 consensus CTCAATCTTA TTAATCTTCA TTCTTCTACT ATCCCCAATC CTAATTTCAA TATCAAACCT AATTAAACAC 11828
Quality scores
AJ489607 consensus CTCAATCTTA TTAATCTTCA TTCTTCTACT ATCCCCAATC CTAATTTCAA TATCAAACCT AATTAAACAC 11830
Quality scores
AJ489607 consensus CTCAATCTTA TTAATCTTCA TTCTTCTACT ATCCCCAATC CTAATTTCAA TATCAAACCT AATTAAACAC 11830
Quality scores
AJ489607 consensus CTCAATCTTA TTAATCTTCA TTCTTCTACT ATCCCCAATC CTAATTTCAA TATCAAACCT AATTAAACAC 11830
Quality scores
AJ489607 consensus CTCAATCTTA TTAATCTTCA TTCTTCTACT ATCCCCAATC CTAATTTCAA TATCAAACCT AATTAAACAC 11828
Quality scores
AJ489607 consensus CTCAATCTTA TTAATCTTCA TTCTTCTACT ATCCCCAATC CTAATTTCAA TATCAAACCT AATTAAACAC 11828
Quality scores
AJ489607 consensus CTCAATCTTA TTAATCTTCA TTCTTCTACT ATCCCCAATC CTAATTTCAA TATCAAACCT AATTAAACAC 11828
Quality scores
AJ489607 consensus CTCAATCTTA TTAATCTTCA TTCTTCTACT ATCCCCAATC CTAATTTCAA TATCAAACCT AATTAAACAC 11828
Quality scores
AJ489607 consensus CTCAATCTTA TTAATCTTCA TTCTTCTACT ATCCCCAATC CTAATTTCAA TATCAAACCT AATTAAACAC 11828
Quality scores
Consensus CTCAATCTTA TTAATCTTCA TTCTTCTACT ATCCCCAATC CTAATTTCAA TATCAAACCT AATTAAACAC
Conservation
Sequence logo
11,840
11,860
11,880
11,900
AJ489607 consensus ATCAACTTCC CACTGTACAC CACCACATCA ATCAAATTCT CCTTCATTAT TAGCCTCTTA CCCCTATTAA 11898
Quality scores
AJ489607 consensus ATCAACTTCC CACTGTACAC CACCACATCA ATCAAATTCT CCTTCATTAT TAGCCTCTTA CCCCTATTAA 11900
Quality scores
AJ489607 consensus ATCAACTTCC CACTGTACAC CACCACATCA ATCAAATTCT CCTTCATTAT TAGCCTCTTA CCCCTATTAA 11900
Quality scores
AJ489607 consensus ATCAACTTCC CACTGTACAC CACCACATCA ATCAAATTCT CCTTCATTAT TAGCCTCTTA CCCCTATTAA 11900
Quality scores
AJ489607 consensus ATCAACTTCC CACTGTACAC CACCACATCA ATCAAATTCT CCTTCATTAT TAGCCTCTTA CCCCTATTAA 11898
Quality scores
AJ489607 consensus ATCAACTTCC CACTGTACAC CACCACATCA ATCAAATTCT CCTTCATTAT TAGCCTCTTA CCCCTATTAA 11898
Quality scores
AJ489607 consensus ATCAACTTCC CACTGTACAC CACCACATCA ATCAAATTCT CCTTCATTAT TAGCCTCTTA CCCCTATTAA 11898
Quality scores
AJ489607 consensus ATCAACTTCC CACTGTACAC CACCACATCA ATCAAATTCT CCTTCATTAT TAGCCTCTTA CCCCTATTAA 11898
Quality scores
AJ489607 consensus ATCAACTTCC CACTGTACAC CACCACATCA ATCAAATTCT CCTTCATTAT TAGCCTCTTA CCCCTATTAA 11898
Quality scores
Consensus ATCAACTTCC CACTGTACAC CACCACATCA ATCAAATTCT CCTTCATTAT TAGCCTCTTA CCCCTATTAA
Conservation
Sequence logo

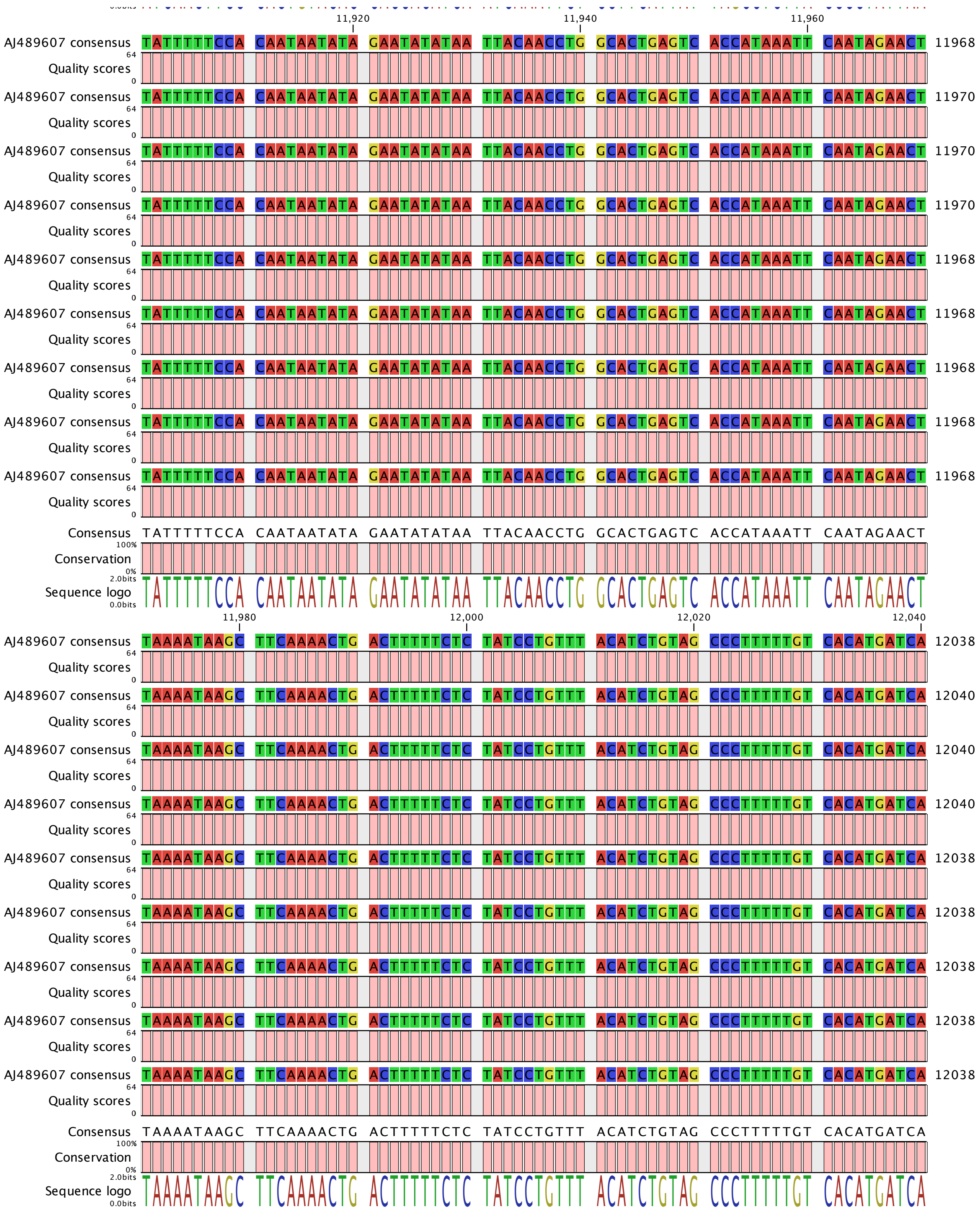
11,920
11,940
11,960
AJ489607 consensus TATTTTTCCA CAATAATATA GAATATATAA TTACAACCTG GCACTGAGTC ACCATAAATT CAATAGAACT 11968
Quality scores
AJ489607 consensus TATTTTTCCA CAATAATATA GAATATATAA TTACAACCTG GCACTGAGTC ACCATAAATT CAATAGAACT 11970
Quality scores
AJ489607 consensus TATTTTTCCA CAATAATATA GAATATATAA TTACAACCTG GCACTGAGTC ACCATAAATT CAATAGAACT 11970
Quality scores
AJ489607 consensus TATTTTTCCA CAATAATATA GAATATATAA TTACAACCTG GCACTGAGTC ACCATAAATT CAATAGAACT 11970
Quality scores
AJ489607 consensus TATTTTTCCA CAATAATATA GAATATATAA TTACAACCTG GCACTGAGTC ACCATAAATT CAATAGAACT 11968
Quality scores
AJ489607 consensus TATTTTTCCA CAATAATATA GAATATATAA TTACAACCTG GCACTGAGTC ACCATAAATT CAATAGAACT 11968
Quality scores
AJ489607 consensus TATTTTTCCA CAATAATATA GAATATATAA TTACAACCTG GCACTGAGTC ACCATAAATT CAATAGAACT 11968
Quality scores
AJ489607 consensus TATTTTTCCA CAATAATATA GAATATATAA TTACAACCTG GCACTGAGTC ACCATAAATT CAATAGAACT 11968
Quality scores
AJ489607 consensus TATTTTTCCA CAATAATATA GAATATATAA TTACAACCTG GCACTGAGTC ACCATAAATT CAATAGAACT 11968
Quality scores
Consensus TATTTTTCCA CAATAATATA GAATATATAA TTACAACCTG GCACTGAGTC ACCATAAATT CAATAGAACT
Conservation
Sequence logo
11,980
12,000
12,020
12,040
AJ489607 consensus TAAAATAAGC TTCAAAACTG ACTTTTTCTC TATCCTGTTT ACATCTGTAG CCCTTTTTGT CACATGATCA 12038
Quality scores
AJ489607 consensus TAAAATAAGC TTCAAAACTG ACTTTTTCTC TATCCTGTTT ACATCTGTAG CCCTTTTTGT CACATGATCA 12040
Quality scores
AJ489607 consensus TAAAATAAGC TTCAAAACTG ACTTTTTCTC TATCCTGTTT ACATCTGTAG CCCTTTTTGT CACATGATCA 12040
Quality scores
AJ489607 consensus TAAAATAAGC TTCAAAACTG ACTTTTTCTC TATCCTGTTT ACATCTGTAG CCCTTTTTGT CACATGATCA 12040
Quality scores
AJ489607 consensus TAAAATAAGC TTCAAAACTG ACTTTTTCTC TATCCTGTTT ACATCTGTAG CCCTTTTTGT CACATGATCA 12038
Quality scores
AJ489607 consensus TAAAATAAGC TTCAAAACTG ACTTTTTCTC TATCCTGTTT ACATCTGTAG CCCTTTTTGT CACATGATCA 12038
Quality scores
AJ489607 consensus TAAAATAAGC TTCAAAACTG ACTTTTTCTC TATCCTGTTT ACATCTGTAG CCCTTTTTGT CACATGATCA 12038
Quality scores
AJ489607 consensus TAAAATAAGC TTCAAAACTG ACTTTTTCTC TATCCTGTTT ACATCTGTAG CCCTTTTTGT CACATGATCA 12038
Quality scores
AJ489607 consensus TAAAATAAGC TTCAAAACTG ACTTTTTCTC TATCCTGTTT ACATCTGTAG CCCTTTTTGT CACATGATCA 12038
Quality scores
Consensus TAAAATAAGC TTCAAAACTG ACTTTTTCTC TATCCTGTTT ACATCTGTAG CCCTTTTTGT CACATGATCA
Conservation
Sequence logo

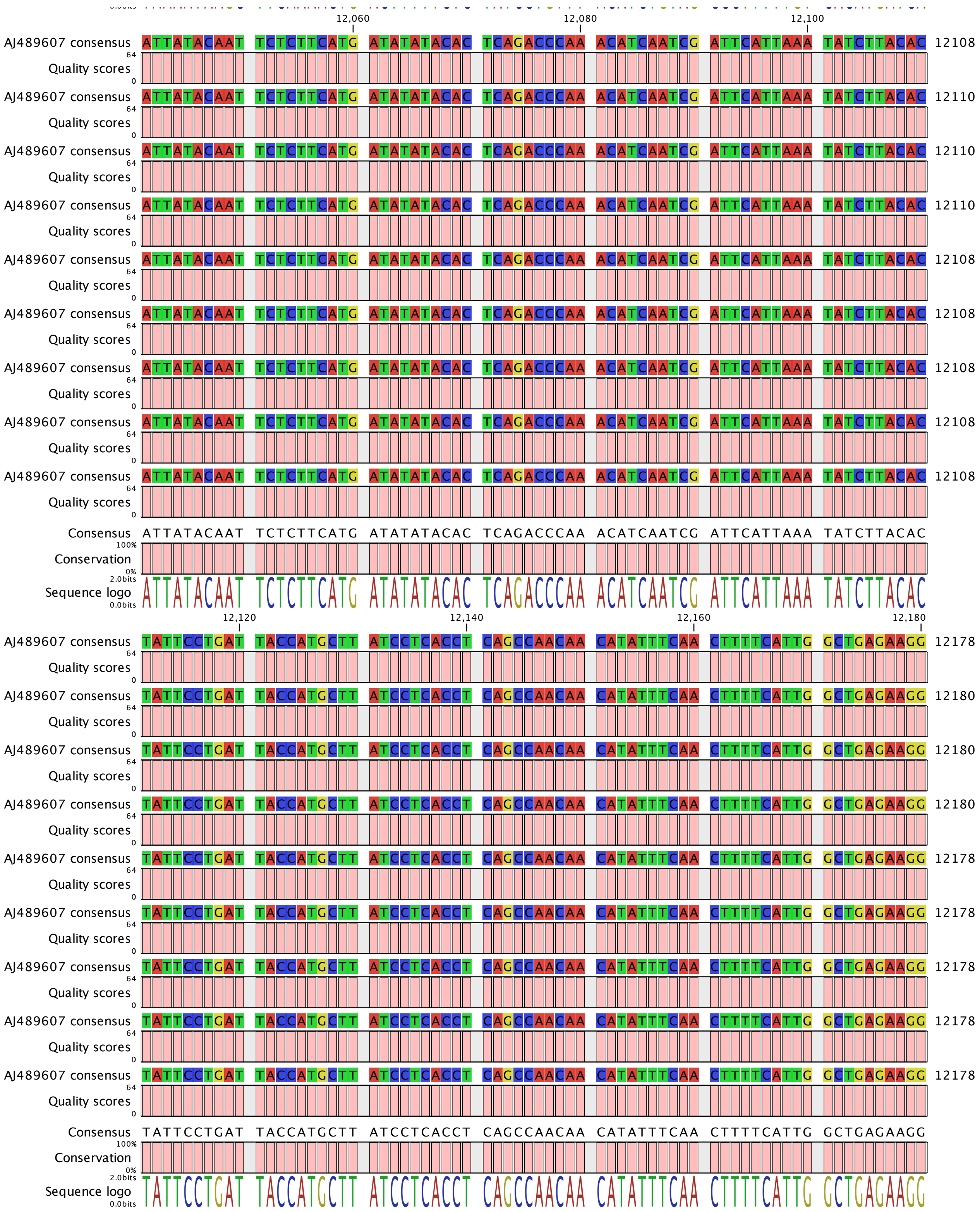
12,060
12,080
12,100
AJ489607 consensus ATTATACAAT TCTCTTCATG ATATATACAC TCAGACCCAA ACATCAATCG ATTCATTAAA TATCTTACAC 12108
Quality scores
AJ489607 consensus ATTATACAAT TCTCTTCATG ATATATACAC TCAGACCCAA ACATCAATCG ATTCATTAAA TATCTTACAC 12110
Quality scores
AJ489607 consensus ATTATACAAT TCTCTTCATG ATATATACAC TCAGACCCAA ACATCAATCG ATTCATTAAA TATCTTACAC 12110
Quality scores
AJ489607 consensus ATTATACAAT TCTCTTCATG ATATATACAC TCAGACCCAA ACATCAATCG ATTCATTAAA TATCTTACAC 12110
Quality scores
AJ489607 consensus ATTATACAAT TCTCTTCATG ATATATACAC TCAGACCCAA ACATCAATCG ATTCATTAAA TATCTTACAC 12108
Quality scores
AJ489607 consensus ATTATACAAT TCTCTTCATG ATATATACAC TCAGACCCAA ACATCAATCG ATTCATTAAA TATCTTACAC 12108
Quality scores
AJ489607 consensus ATTATACAAT TCTCTTCATG ATATATACAC TCAGACCCAA ACATCAATCG ATTCATTAAA TATCTTACAC 12108
Quality scores
AJ489607 consensus ATTATACAAT TCTCTTCATG ATATATACAC TCAGACCCAA ACATCAATCG ATTCATTAAA TATCTTACAC 12108
Quality scores
AJ489607 consensus ATTATACAAT TCTCTTCATG ATATATACAC TCAGACCCAA ACATCAATCG ATTCATTAAA TATCTTACAC 12108
Quality scores
Consensus ATTATACAAT TCTCTTCATG ATATATACAC TCAGACCCAA ACATCAATCG ATTCATTAAA TATCTTACAC
Conservation
Sequence logo
12,120
12,140
12,160
12,180
AJ489607 consensus TATTCCTGAT TACCATGCTT ATCCTCACCT CAGCCAACAA CATATTTCAA CTTTTCATTG GCTGAGAAGG 12178
Quality scores
AJ489607 consensus TATTCCTGAT TACCATGCTT ATCCTCACCT CAGCCAACAA CATATTTCAA CTTTTCATTG GCTGAGAAGG 12180
Quality scores
AJ489607 consensus TATTCCTGAT TACCATGCTT ATCCTCACCT CAGCCAACAA CATATTTCAA CTTTTCATTG GCTGAGAAGG 12180
Quality scores
AJ489607 consensus TATTCCTGAT TACCATGCTT ATCCTCACCT CAGCCAACAA CATATTTCAA CTTTTCATTG GCTGAGAAGG 12180
Quality scores
AJ489607 consensus TATTCCTGAT TACCATGCTT ATCCTCACCT CAGCCAACAA CATATTTCAA CTTTTCATTG GCTGAGAAGG 12178
Quality scores
AJ489607 consensus TATTCCTGAT TACCATGCTT ATCCTCACCT CAGCCAACAA CATATTTCAA CTTTTCATTG GCTGAGAAGG 12178
Quality scores
AJ489607 consensus TATTCCTGAT TACCATGCTT ATCCTCACCT CAGCCAACAA CATATTTCAA CTTTTCATTG GCTGAGAAGG 12178
Quality scores
AJ489607 consensus TATTCCTGAT TACCATGCTT ATCCTCACCT CAGCCAACAA CATATTTCAA CTTTTCATTG GCTGAGAAGG 12178
Quality scores
AJ489607 consensus TATTCCTGAT TACCATGCTT ATCCTCACCT CAGCCAACAA CATATTTCAA CTTTTCATTG GCTGAGAAGG 12178
Quality scores
Consensus TATTCCTGAT TACCATGCTT ATCCTCACCT CAGCCAACAA CATATTTCAA CTTTTCATTG GCTGAGAAGG
Conservation
Sequence logo

12,200 12,220 12,240

AJ489607 consensus	GGTGGGAATT ATATCTTTCC TACTAATTGG ATGATGGTAC GGACGAACAG ACGCAAATAC TGCAGCCCTA	12248
Quality scores (64 / 0)		
AJ489607 consensus	GGTGGGAATT ATATCTTTCC TACTAATTGG ATGATGGTAC GGACGAACAG ACGCAAATAC TGCAGCCCTA	12250
Quality scores (64 / 0)		
AJ489607 consensus	GGTGGGAATT ATATCTTTCC TACTAATTGG ATGATGGTAC GGACGAACAG ACGCAAATAC TGCAGCCCTA	12250
Quality scores (64 / 0)		
AJ489607 consensus	GGTGGGAATT ATATCTTTCC TACTAATTGG ATGATGGTAC GGACGAACAG ACGCAAATAC TGCAGCCCTA	12250
Quality scores (64 / 0)		
AJ489607 consensus	GGTGGGAATT ATATCTTTCC TACTAATTGG ATGATGGTAC GGACGAACAG ACGCAAATAC TGCAGCCCTA	12248
Quality scores (64 / 0)		
AJ489607 consensus	GGTGGGAATT ATATCTTTCC TACTAATTGG ATGATGGTAC GGACGAACAG ACGCAAATAC TGCAGCCCTA	12248
Quality scores (64 / 0)		
AJ489607 consensus	GGTGGGAATT ATATCTTTCC TACTAATTGG ATGATGGTAC GGACGAACAG ACGCAAATAC TGCAGCCCTA	12248
Quality scores (64 / 0)		
AJ489607 consensus	GGTGGGAATT ATATCTTTCC TACTAATTGG ATGATGGTAC GGACGAACAG ACGCAAATAC TGCAGCCCTA	12248
Quality scores (64 / 0)		
AJ489607 consensus	GGTGGGAATT ATATCTTTCC TACTAATTGG ATGATGGTAC GGACGAACAG ACGCAAATAC TGCAGCCCTA	12248
Quality scores (64 / 0)		
Consensus	GGTGGGAATT ATATCTTTCC TACTAATTGG ATGATGGTAC GGACGAACAG ACGCAAATAC TGCAGCCCTA	
Conservation (100% / 0%)		
Sequence logo (2.0bits / 0.0bits)	GGTGGGAATT ATATCTTTCC TACTAATTGG ATGATGGTAC GGACGAACAG ACGCAAATAC TGCAGCCCTA	

12,260 12,280 12,300 12,320

AJ489607 consensus	CAAGCAATCC TCTATAACCG CATCGGAGAC ATCGGATTCA TTTTAGCTAT AGTTTGATTT TCCCTAAACA	12318
Quality scores (64 / 0)		
AJ489607 consensus	CAAGCAATCC TCTATAACCG CATCGGAGAC ATCGGATTCA TTTTAGCTAT AGTTTGATTT TCCCTAAACA	12320
Quality scores (64 / 0)		
AJ489607 consensus	CAAGCAATCC TCTATAACCG CATCGGAGAC ATCGGATTCA TTTTAGCTAT AGTTTGATTT TCCCTAAACA	12320
Quality scores (64 / 0)		
AJ489607 consensus	CAAGCAATCC TCTATAACCG CATCGGAGAC ATCGGATTCA TTTTAGCTAT AGTTTGATTT TCCCTAAACA	12320
Quality scores (64 / 0)		
AJ489607 consensus	CAAGCAATCC TCTATAACCG CATCGGAGAC ATCGGATTCA TTTTAGCTAT AGTTTGATTT TCCCTAAACA	12318
Quality scores (64 / 0)		
AJ489607 consensus	CAAGCAATCC TCTATAACCG CATCGGAGAC ATCGGATTCA TTTTAGCTAT AGTTTGATTT TCCCTAAACA	12318
Quality scores (64 / 0)		
AJ489607 consensus	CAAGCAATCC TCTATAACCG CATCGGAGAC ATCGGATTCA TTTTAGCTAT AGTTTGATTT TCCCTAAACA	12318
Quality scores (64 / 0)		
AJ489607 consensus	CAAGCAATCC TCTATAACCG CATCGGAGAC ATCGGATTCA TTTTAGCTAT AGTTTGATTT TCCCTAAACA	12318
Quality scores (64 / 0)		
AJ489607 consensus	CAAGCAATCC TCTATAACCG CATCGGAGAC ATCGGATTCA TTTTAGCTAT AGTTTGATTT TCCCTAAACA	12318
Quality scores (64 / 0)		
Consensus	CAAGCAATCC TCTATAACCG CATCGGAGAC ATCGGATTCA TTTTAGCTAT AGTTTGATTT TCCCTAAACA	
Conservation (100% / 0%)		
Sequence logo (2.0bits / 0.0bits)	CAAGCAATCC TCTATAACCG CATCGGAGAC ATCGGATTCA TTTTAGCTAT AGTTTGATTT TCCCTAAACA	

12,340 12,360 12,380

AJ489607 consensus TAAACTCATG AGAACTTCAA CAGATTATAT TCTCCAACAA CAACGACAAT CTAATTCCAC TTATAGGCCT 12388
64
Quality scores
0
AJ489607 consensus TAAACTCATG AGAACTTCAA CAGATTATAT TCTCCAACAA CAACGACAAT CTAATTCCAC TTATAGGCCT 12390
64
Quality scores
0
AJ489607 consensus TAAACTCATG AGAACTTCAA CAGATTATAT TCTCCAACAA CAACGACAAT CTAATTCCAC TTATAGGCCT 12390
64
Quality scores
0
AJ489607 consensus TAAACTCATG AGAACTTCAA CAGATTATAT TCTCCAACAA CAACGACAAT CTAATTCCAC TTATAGGCCT 12390
64
Quality scores
0
AJ489607 consensus TAAACTCATG AGAACTTCAA CAGATTATAT TCTCCAACAA CAACGACAAT CTAATTCCAC TTATAGGCCT 12388
64
Quality scores
0
AJ489607 consensus TAAACTCATG AGAACTTCAA CAGATTATAT TCTCCAACAA CAACGACAAT CTAATTCCAC TTATAGGCCT 12388
64
Quality scores
0
AJ489607 consensus TAAACTCATG AGAACTTCAA CAGATTATAT TCTCCAACAA CAACGACAAT CTAATTCCAC TTATAGGCCT 12388
64
Quality scores
0
AJ489607 consensus TAAACTCATG AGAACTTCAA CAGATTATAT TCTCCAACAA CAACGACAAT CTAATTCCAC TTATAGGCCT 12388
64
Quality scores
0
AJ489607 consensus TAAACTCATG AGAACTTCAA CAGATTATAT TCTCCAACAA CAACGACAAT CTAATTCCAC TTATAGGCCT 12388
64
Quality scores
0
Consensus TAAACTCATG AGAACTTCAA CAGATTATAT TCTCCAACAA CAACGACAAT CTAATTCCAC TTATAGGCCT
100%
Conservation
0%
2.0bits
Sequence logo TAAACTCATG AGAACTTCAA CAGATTATAT TCTCCAACAA CAACGACAAT CTAATTCCAC TTATAGGCCT
0.0bits

12,400 12,420 12,440 12,460

AJ489607 consensus ATTAATCGCA GCTACAGGAA AATCAGCACA ATTTGGCCTC CACCCATGAC TACCATCAGC AATAGAAGGC 12458
64
Quality scores
0
AJ489607 consensus ATTAATCGCA GCTACAGGAA AATCAGCACA ATTTGGCCTC CACCCATGAC TACCATCAGC AATAGAAGGC 12460
64
Quality scores
0
AJ489607 consensus ATTAATCGCA GCTACAGGAA AATCAGCACA ATTTGGCCTC CACCCATGAC TACCATCAGC AATAGAAGGC 12460
64
Quality scores
0
AJ489607 consensus ATTAATCGCA GCTACAGGAA AATCAGCACA ATTTGGCCTC CACCCATGAC TACCATCAGC AATAGAAGGC 12460
64
Quality scores
0
AJ489607 consensus ATTAATCGCA GCTACAGGAA AATCAGCACA ATTTGGCCTC CACCCATGAC TACCATCAGC AATAGAAGGC 12458
64
Quality scores
0
AJ489607 consensus ATTAATCGCA GCTACAGGAA AATCAGCACA ATTTGGCCTC CACCCATGAC TACCATCAGC AATAGAAGGC 12458
64
Quality scores
0
AJ489607 consensus ATTAATCGCA GCTACAGGAA AATCAGCACA ATTTGGCCTC CACCCATGAC TACCATCAGC AATAGAAGGC 12458
64
Quality scores
0
AJ489607 consensus ATTAATCGCA GCTACAGGAA AATCAGCACA ATTTGGCCTC CACCCATGAC TACCATCAGC AATAGAAGGC 12458
64
Quality scores
0
AJ489607 consensus ATTAATCGCA GCTACAGGAA AATCAGCACA ATTTGGCCTC CACCCATGAC TACCATCAGC AATAGAAGGC 12458
64
Quality scores
0
Consensus ATTAATCGCA GCTACAGGAA AATCAGCACA ATTTGGCCTC CACCCATGAC TACCATCAGC AATAGAAGGC
100%
Conservation
0%
2.0bits
Sequence logo ATTAATCGCA GCTACAGGAA AATCAGCACA ATTTGGCCTC CACCCATGAC TACCATCAGC AATAGAAGGC
0.0bits

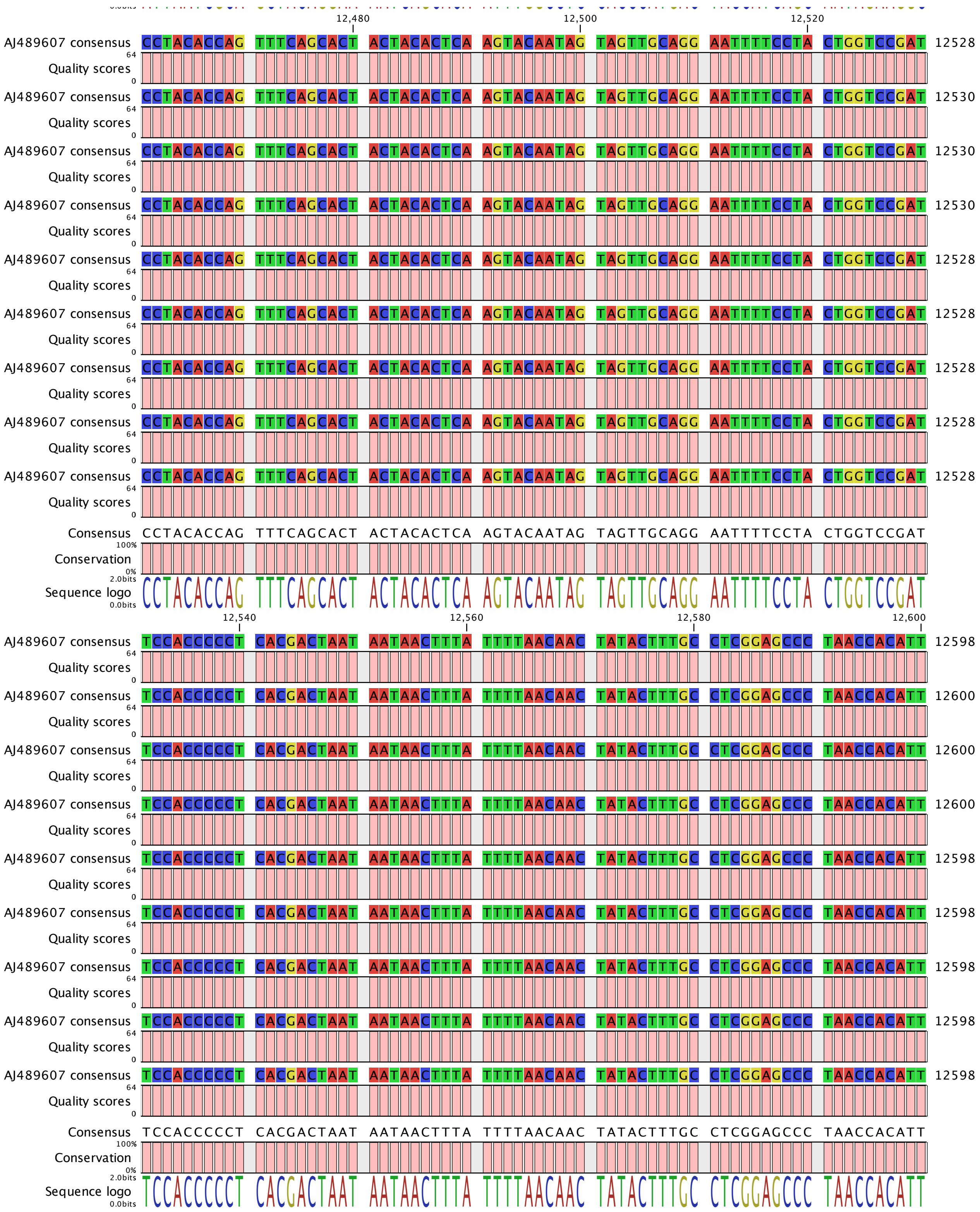
12,480 12,500 12,520
AJ489607 consensus CCTACACCAG TTTCAGCACT ACTACACTCA AGTACAATAG TAGTTGCAGG AATTTTCCTA CTGGTCCGAT 12528
Quality scores
AJ489607 consensus CCTACACCAG TTTCAGCACT ACTACACTCA AGTACAATAG TAGTTGCAGG AATTTTCCTA CTGGTCCGAT 12530
Quality scores
AJ489607 consensus CCTACACCAG TTTCAGCACT ACTACACTCA AGTACAATAG TAGTTGCAGG AATTTTCCTA CTGGTCCGAT 12530
Quality scores
AJ489607 consensus CCTACACCAG TTTCAGCACT ACTACACTCA AGTACAATAG TAGTTGCAGG AATTTTCCTA CTGGTCCGAT 12530
Quality scores
AJ489607 consensus CCTACACCAG TTTCAGCACT ACTACACTCA AGTACAATAG TAGTTGCAGG AATTTTCCTA CTGGTCCGAT 12528
Quality scores
AJ489607 consensus CCTACACCAG TTTCAGCACT ACTACACTCA AGTACAATAG TAGTTGCAGG AATTTTCCTA CTGGTCCGAT 12528
Quality scores
AJ489607 consensus CCTACACCAG TTTCAGCACT ACTACACTCA AGTACAATAG TAGTTGCAGG AATTTTCCTA CTGGTCCGAT 12528
Quality scores
AJ489607 consensus CCTACACCAG TTTCAGCACT ACTACACTCA AGTACAATAG TAGTTGCAGG AATTTTCCTA CTGGTCCGAT 12528
Quality scores
AJ489607 consensus CCTACACCAG TTTCAGCACT ACTACACTCA AGTACAATAG TAGTTGCAGG AATTTTCCTA CTGGTCCGAT 12528
Quality scores
Consensus CCTACACCAG TTTCAGCACT ACTACACTCA AGTACAATAG TAGTTGCAGG AATTTTCCTA CTGGTCCGAT
Conservation
Sequence logo
12,540 12,560 12,580 12,600
AJ489607 consensus TCCACCCCCT CACGACTAAT AATAACTTTA TTTTAACAAC TATACTTTGC CTCGGAGCCC TAACCACATT 12598
Quality scores
AJ489607 consensus TCCACCCCCT CACGACTAAT AATAACTTTA TTTTAACAAC TATACTTTGC CTCGGAGCCC TAACCACATT 12600
Quality scores
AJ489607 consensus TCCACCCCCT CACGACTAAT AATAACTTTA TTTTAACAAC TATACTTTGC CTCGGAGCCC TAACCACATT 12600
Quality scores
AJ489607 consensus TCCACCCCCT CACGACTAAT AATAACTTTA TTTTAACAAC TATACTTTGC CTCGGAGCCC TAACCACATT 12600
Quality scores
AJ489607 consensus TCCACCCCCT CACGACTAAT AATAACTTTA TTTTAACAAC TATACTTTGC CTCGGAGCCC TAACCACATT 12598
Quality scores
AJ489607 consensus TCCACCCCCT CACGACTAAT AATAACTTTA TTTTAACAAC TATACTTTGC CTCGGAGCCC TAACCACATT 12598
Quality scores
AJ489607 consensus TCCACCCCCT CACGACTAAT AATAACTTTA TTTTAACAAC TATACTTTGC CTCGGAGCCC TAACCACATT 12598
Quality scores
AJ489607 consensus TCCACCCCCT CACGACTAAT AATAACTTTA TTTTAACAAC TATACTTTGC CTCGGAGCCC TAACCACATT 12598
Quality scores
AJ489607 consensus TCCACCCCCT CACGACTAAT AATAACTTTA TTTTAACAAC TATACTTTGC CTCGGAGCCC TAACCACATT 12598
Quality scores
Consensus TCCACCCCCT CACGACTAAT AATAACTTTA TTTTAACAAC TATACTTTGC CTCGGAGCCC TAACCACATT
Conservation
Sequence logo

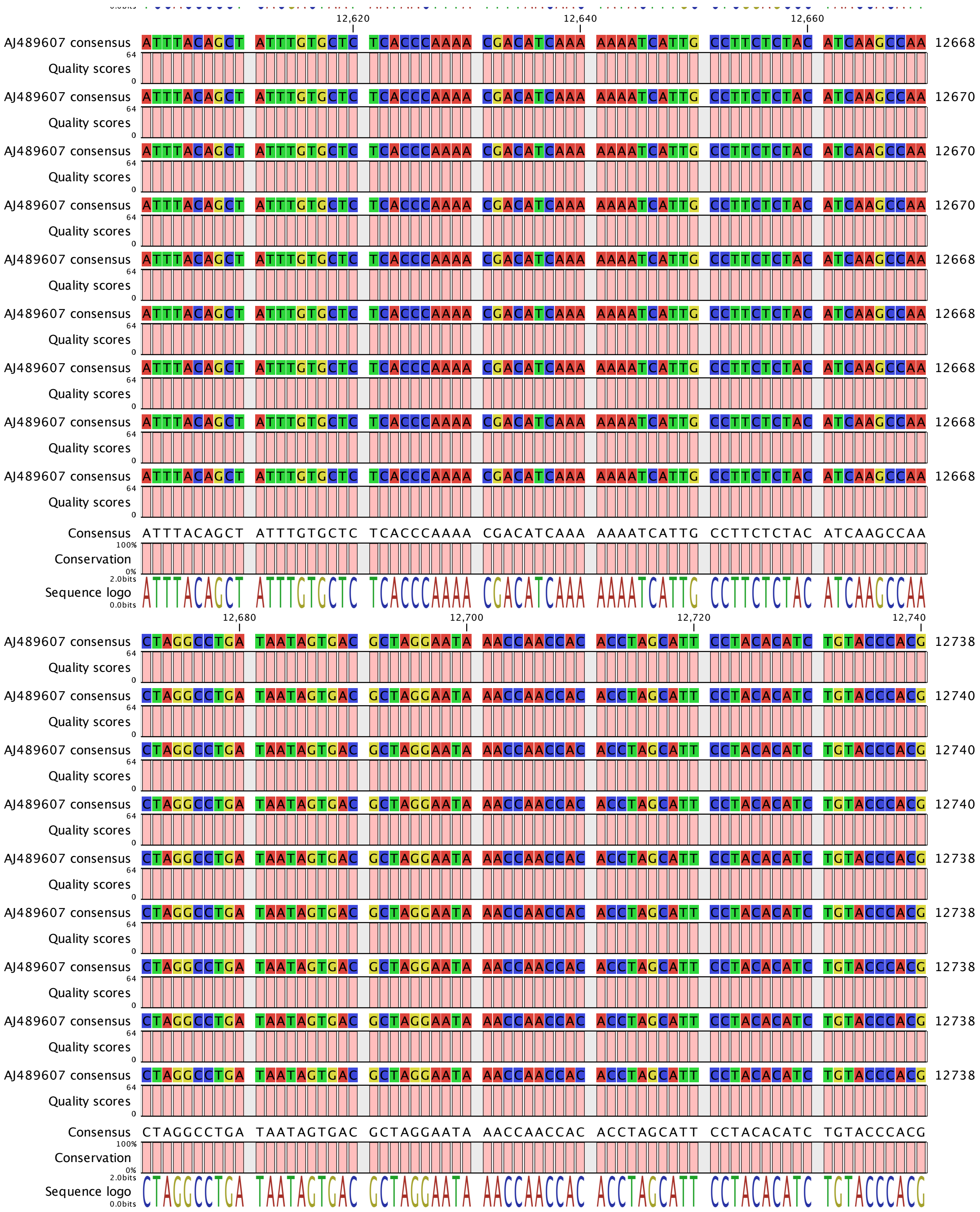
12,620
12,640
12,660
AJ489607 consensus ATTTACAGCT ATTTGTGCTC TCACCCAAAA CGACATCAAA AAAATCATTG CCTTCTCTAC ATCAAGCCAA 12668
Quality scores
AJ489607 consensus ATTTACAGCT ATTTGTGCTC TCACCCAAAA CGACATCAAA AAAATCATTG CCTTCTCTAC ATCAAGCCAA 12670
Quality scores
AJ489607 consensus ATTTACAGCT ATTTGTGCTC TCACCCAAAA CGACATCAAA AAAATCATTG CCTTCTCTAC ATCAAGCCAA 12670
Quality scores
AJ489607 consensus ATTTACAGCT ATTTGTGCTC TCACCCAAAA CGACATCAAA AAAATCATTG CCTTCTCTAC ATCAAGCCAA 12670
Quality scores
AJ489607 consensus ATTTACAGCT ATTTGTGCTC TCACCCAAAA CGACATCAAA AAAATCATTG CCTTCTCTAC ATCAAGCCAA 12668
Quality scores
AJ489607 consensus ATTTACAGCT ATTTGTGCTC TCACCCAAAA CGACATCAAA AAAATCATTG CCTTCTCTAC ATCAAGCCAA 12668
Quality scores
AJ489607 consensus ATTTACAGCT ATTTGTGCTC TCACCCAAAA CGACATCAAA AAAATCATTG CCTTCTCTAC ATCAAGCCAA 12668
Quality scores
AJ489607 consensus ATTTACAGCT ATTTGTGCTC TCACCCAAAA CGACATCAAA AAAATCATTG CCTTCTCTAC ATCAAGCCAA 12668
Quality scores
AJ489607 consensus ATTTACAGCT ATTTGTGCTC TCACCCAAAA CGACATCAAA AAAATCATTG CCTTCTCTAC ATCAAGCCAA 12668
Quality scores
Consensus ATTTACAGCT ATTTGTGCTC TCACCCAAAA CGACATCAAA AAAATCATTG CCTTCTCTAC ATCAAGCCAA
Conservation
Sequence logo
12,680
12,700
12,720
12,740
AJ489607 consensus CTAGGCCTGA TAATAGTGAC GCTAGGAATA AACCAACCAC ACCTAGCATT CCTACACATC TGTACCCACG 12738
Quality scores
AJ489607 consensus CTAGGCCTGA TAATAGTGAC GCTAGGAATA AACCAACCAC ACCTAGCATT CCTACACATC TGTACCCACG 12740
Quality scores
AJ489607 consensus CTAGGCCTGA TAATAGTGAC GCTAGGAATA AACCAACCAC ACCTAGCATT CCTACACATC TGTACCCACG 12740
Quality scores
AJ489607 consensus CTAGGCCTGA TAATAGTGAC GCTAGGAATA AACCAACCAC ACCTAGCATT CCTACACATC TGTACCCACG 12740
Quality scores
AJ489607 consensus CTAGGCCTGA TAATAGTGAC GCTAGGAATA AACCAACCAC ACCTAGCATT CCTACACATC TGTACCCACG 12738
Quality scores
AJ489607 consensus CTAGGCCTGA TAATAGTGAC GCTAGGAATA AACCAACCAC ACCTAGCATT CCTACACATC TGTACCCACG 12738
Quality scores
AJ489607 consensus CTAGGCCTGA TAATAGTGAC GCTAGGAATA AACCAACCAC ACCTAGCATT CCTACACATC TGTACCCACG 12738
Quality scores
AJ489607 consensus CTAGGCCTGA TAATAGTGAC GCTAGGAATA AACCAACCAC ACCTAGCATT CCTACACATC TGTACCCACG 12738
Quality scores
AJ489607 consensus CTAGGCCTGA TAATAGTGAC GCTAGGAATA AACCAACCAC ACCTAGCATT CCTACACATC TGTACCCACG 12738
Quality scores
Consensus CTAGGCCTGA TAATAGTGAC GCTAGGAATA AACCAACCAC ACCTAGCATT CCTACACATC TGTACCCACG
Conservation
Sequence logo

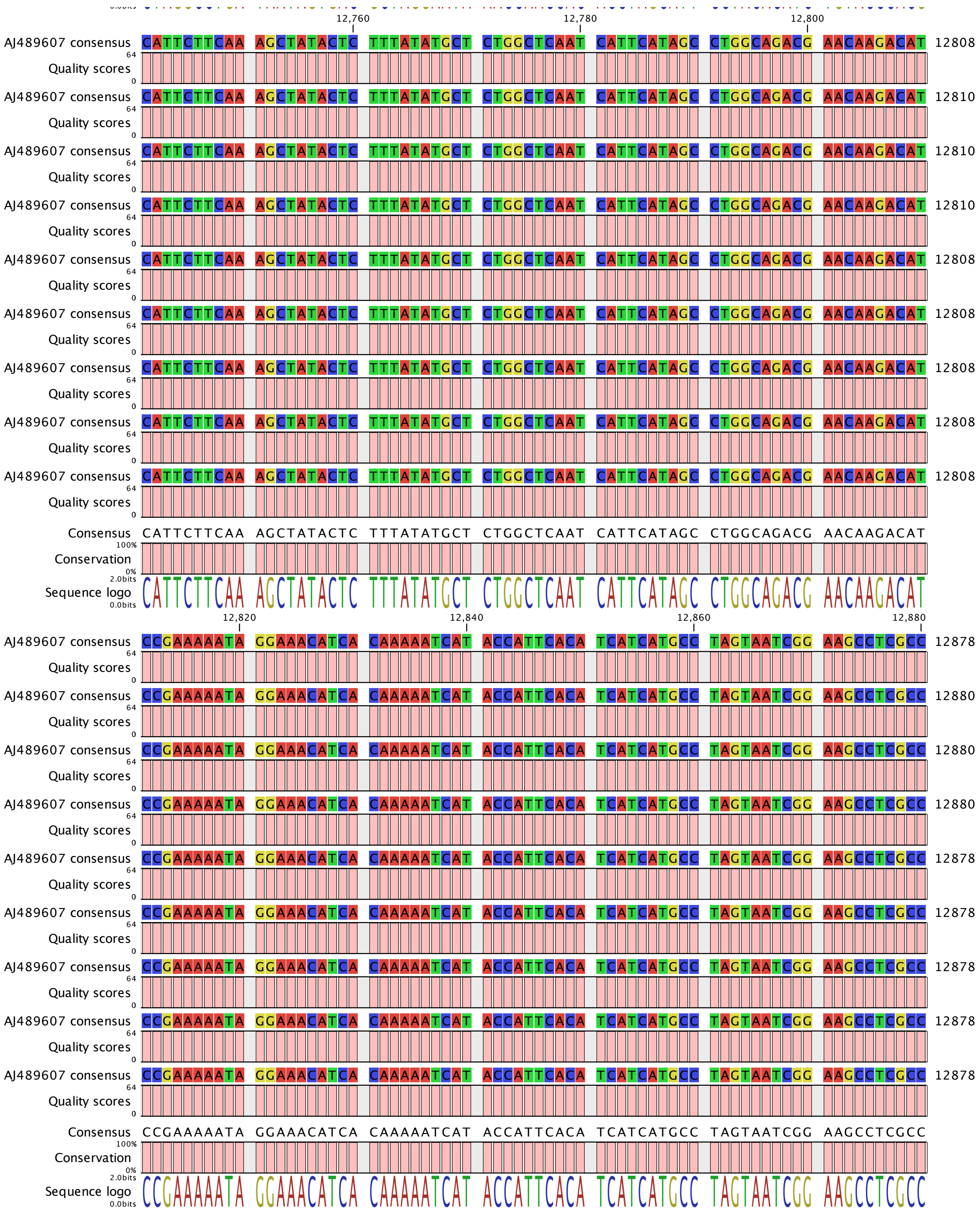
12,760
12,780
12,800
AJ489607 consensus CATTCTTCAA AGCTATACTC TTTATATGCT CTGGCTCAAT CATTCATAGC CTGGCAGACG AACAAGACAT 12808
Quality scores
AJ489607 consensus CATTCTTCAA AGCTATACTC TTTATATGCT CTGGCTCAAT CATTCATAGC CTGGCAGACG AACAAGACAT 12810
Quality scores
AJ489607 consensus CATTCTTCAA AGCTATACTC TTTATATGCT CTGGCTCAAT CATTCATAGC CTGGCAGACG AACAAGACAT 12810
Quality scores
AJ489607 consensus CATTCTTCAA AGCTATACTC TTTATATGCT CTGGCTCAAT CATTCATAGC CTGGCAGACG AACAAGACAT 12810
Quality scores
AJ489607 consensus CATTCTTCAA AGCTATACTC TTTATATGCT CTGGCTCAAT CATTCATAGC CTGGCAGACG AACAAGACAT 12808
Quality scores
AJ489607 consensus CATTCTTCAA AGCTATACTC TTTATATGCT CTGGCTCAAT CATTCATAGC CTGGCAGACG AACAAGACAT 12808
Quality scores
AJ489607 consensus CATTCTTCAA AGCTATACTC TTTATATGCT CTGGCTCAAT CATTCATAGC CTGGCAGACG AACAAGACAT 12808
Quality scores
AJ489607 consensus CATTCTTCAA AGCTATACTC TTTATATGCT CTGGCTCAAT CATTCATAGC CTGGCAGACG AACAAGACAT 12808
Quality scores
AJ489607 consensus CATTCTTCAA AGCTATACTC TTTATATGCT CTGGCTCAAT CATTCATAGC CTGGCAGACG AACAAGACAT 12808
Quality scores
Consensus CATTCTTCAA AGCTATACTC TTTATATGCT CTGGCTCAAT CATTCATAGC CTGGCAGACG AACAAGACAT
Conservation
Sequence logo
12,820
12,840
12,860
12,880
AJ489607 consensus CCGAAAAATA GGAAACATCA CAAAAATCAT ACCATTCACA TCATCATGCC TAGTAATCGG AAGCCTCGCC 12878
Quality scores
AJ489607 consensus CCGAAAAATA GGAAACATCA CAAAAATCAT ACCATTCACA TCATCATGCC TAGTAATCGG AAGCCTCGCC 12880
Quality scores
AJ489607 consensus CCGAAAAATA GGAAACATCA CAAAAATCAT ACCATTCACA TCATCATGCC TAGTAATCGG AAGCCTCGCC 12880
Quality scores
AJ489607 consensus CCGAAAAATA GGAAACATCA CAAAAATCAT ACCATTCACA TCATCATGCC TAGTAATCGG AAGCCTCGCC 12880
Quality scores
AJ489607 consensus CCGAAAAATA GGAAACATCA CAAAAATCAT ACCATTCACA TCATCATGCC TAGTAATCGG AAGCCTCGCC 12878
Quality scores
AJ489607 consensus CCGAAAAATA GGAAACATCA CAAAAATCAT ACCATTCACA TCATCATGCC TAGTAATCGG AAGCCTCGCC 12878
Quality scores
AJ489607 consensus CCGAAAAATA GGAAACATCA CAAAAATCAT ACCATTCACA TCATCATGCC TAGTAATCGG AAGCCTCGCC 12878
Quality scores
AJ489607 consensus CCGAAAAATA GGAAACATCA CAAAAATCAT ACCATTCACA TCATCATGCC TAGTAATCGG AAGCCTCGCC 12878
Quality scores
AJ489607 consensus CCGAAAAATA GGAAACATCA CAAAAATCAT ACCATTCACA TCATCATGCC TAGTAATCGG AAGCCTCGCC 12878
Quality scores
Consensus CCGAAAAATA GGAAACATCA CAAAAATCAT ACCATTCACA TCATCATGCC TAGTAATCGG AAGCCTCGCC
Conservation
Sequence logo

0.0bits

12,900 12,920 12,940

AJ489607 consensus CTCACAGGAA TACCATTCCT AACAGGGTTC TACTCAAAAG ACCTAATTAT TGAAGCAATT AATACCTGCA 12948
64
Quality scores
0

AJ489607 consensus CTCACAGGAA TACCATTCCT AACAGGGTTC TACTCAAAAG ACCTAATTAT TGAAGCAATT AATACCTGCA 12950
64
Quality scores
0

AJ489607 consensus CTCACAGGAA TACCATTCCT AACAGGGTTC TACTCAAAAG ACCTAATTAT TGAAGCAATT AATACCTGCA 12950
64
Quality scores
0

AJ489607 consensus CTCACAGGAA TACCATTCCT AACAGGGTTC TACTCAAAAG ACCTAATTAT TGAAGCAATT AATACCTGCA 12950
64
Quality scores
0

AJ489607 consensus CTCACAGGAA TACCATTCCT AACAGGGTTC TACTCAAAAG ACCTAATTAT TGAAGCAATT AATACCTGCA 12948
64
Quality scores
0

AJ489607 consensus CTCACAGGAA TACCATTCCT AACAGGGTTC TACTCAAAAG ACCTAATTAT TGAAGCAATT AATACCTGCA 12948
64
Quality scores
0

AJ489607 consensus CTCACAGGAA TACCATTCCT AACAGGGTTC TACTCAAAAG ACCTAATTAT TGAAGCAATT AATACCTGCA 12948
64
Quality scores
0

AJ489607 consensus CTCACAGGAA TACCATTCCT AACAGGGTTC TACTCAAAAG ACCTAATTAT TGAAGCAATT AATACCTGCA 12948
64
Quality scores
0

AJ489607 consensus CTCACAGGAA TACCATTCCT AACAGGGTTC TACTCAAAAG ACCTAATTAT TGAAGCAATT AATACCTGCA 12948
64
Quality scores
0

Consensus CTCACAGGAA TACCATTCCT AACAGGGTTC TACTCAAAAG ACCTAATTAT TGAAGCAATT AATACCTGCA
100%
Conservation
0%
2.0bits
Sequence logo CTCACAGGAA TACCATTCCT AACAGGGTTC TACTCAAAAG ACCTAATTAT TGAAGCAATT AATACCTGCA
0.0bits

12,960 12,980 13,000 13,020

AJ489607 consensus ACACCAACGC CTGAGCCCTA CTAATTACAC TAATCGCCAC TTCTATAACA GCTATGTACA GCATACGAAT 13018
64
Quality scores
0

AJ489607 consensus ACACCAACGC CTGAGCCCTA CTAATTACAC TAATCGCCAC TTCTATAACA GCTATGTACA GCATACGAAT 13020
64
Quality scores
0

AJ489607 consensus ACACCAACGC CTGAGCCCTA CTAATTACAC TAATCGCCAC TTCTATAACA GCTATGTACA GCATACGAAT 13020
64
Quality scores
0

AJ489607 consensus ACACCAACGC CTGAGCCCTA CTAATTACAC TAATCGCCAC TTCTATAACA GCTATGTACA GCATACGAAT 13020
64
Quality scores
0

AJ489607 consensus ACACCAACGC CTGAGCCCTA CTAATTACAC TAATCGCCAC TTCTATAACA GCTATGTACA GCATACGAAT 13018
64
Quality scores
0

AJ489607 consensus ACACCAACGC CTGAGCCCTA CTAATTACAC TAATCGCCAC TTCTATAACA GCTATGTACA GCATACGAAT 13018
64
Quality scores
0

AJ489607 consensus ACACCAACGC CTGAGCCCTA CTAATTACAC TAATCGCCAC TTCTATAACA GCTATGTACA GCATACGAAT 13018
64
Quality scores
0

AJ489607 consensus ACACCAACGC CTGAGCCCTA CTAATTACAC TAATCGCCAC TTCTATAACA GCTATGTACA GCATACGAAT 13018
64
Quality scores
0

AJ489607 consensus ACACCAACGC CTGAGCCCTA CTAATTACAC TAATCGCCAC TTCTATAACA GCTATGTACA GCATACGAAT 13018
64
Quality scores
0

Consensus ACACCAACGC CTGAGCCCTA CTAATTACAC TAATCGCCAC TTCTATAACA GCTATGTACA GCATACGAAT
100%
Conservation
0%
2.0bits
Sequence logo ACACCAACGC CTGAGCCCTA CTAATTACAC TAATCGCCAC TTCTATAACA GCTATGTACA GCATACGAAT
0.0bits

13,040 13,060 13,080

AJ489607 consensus CATTTACTTC GTAACAATAA CAAAACCGCG TTTTCCCCCC CTAATCTCCA TTAACGAAAA TGACCCAGAC 13088
64 Quality scores 0
AJ489607 consensus CATTTACTTC GTAACAATAA CAAAACCGCG TTTTCCCCCC CTAATCTCCA TTAACGAAAA TGACCCAGAC 13090
64 Quality scores 0
AJ489607 consensus CATTTACTTC GTAACAATAA CAAAACCGCG TTTTCCCCCC CTAATCTCCA TTAACGAAAA TGACCCAGAC 13090
64 Quality scores 0
AJ489607 consensus CATTTACTTC GTAACAATAA CAAAACCGCG TTTTCCCCCC CTAATCTCCA TTAACGAAAA TGACCCAGAC 13090
64 Quality scores 0
AJ489607 consensus CATTTACTTC GTAACAATAA CAAAACCGCG TTTTCCCCCC CTAATCTCCA TTAACGAAAA TGACCCAGAC 13088
64 Quality scores 0
AJ489607 consensus CATTTACTTC GTAACAATAA CAAAACCGCG TTTTCCCCCC CTAATCTCCA TTAACGAAAA TGACCCAGAC 13088
64 Quality scores 0
AJ489607 consensus CATTTACTTC GTAACAATAA CAAAACCGCG TTTTCCCCCC CTAATCTCCA TTAACGAAAA TGACCCAGAC 13088
64 Quality scores 0
AJ489607 consensus CATTTACTTC GTAACAATAA CAAAACCGCG TTTTCCCCCC CTAATCTCCA TTAACGAAAA TGACCCAGAC 13088
64 Quality scores 0
AJ489607 consensus CATTTACTTC GTAACAATAA CAAAACCGCG TTTTCCCCCC CTAATCTCCA TTAACGAAAA TGACCCAGAC 13088
64 Quality scores 0
Consensus CATTTACTTC GTAACAATAA CAAAACCGCG TTTTCCCCCC CTAATCTCCA TTAACGAAAA TGACCCAGAC
100% Conservation 0%
2.0bits Sequence logo 0.0bits

13,100 13,120 13,140 13,160

AJ489607 consensus CTCATAAACC CAATCAAACG CCTAGCATTC GGAAGCATCT TTGCAGGATT TGTCATCTCA TATAATATTC 13158
64 Quality scores 0
AJ489607 consensus CTCATAAACC CAATCAAACG CCTAGCATTC GGAAGCATCT TTGCAGGATT TGTCATCTCA TATAATATTC 13160
64 Quality scores 0
AJ489607 consensus CTCATAAACC CAATCAAACG CCTAGCATTC GGAAGCATCT TTGCAGGATT TGTCATCTCA TATAATATTC 13160
64 Quality scores 0
AJ489607 consensus CTCATAAACC CAATCAAACG CCTAGCATTC GGAAGCATCT TTGCAGGATT TGTCATCTCA TATAATATTC 13160
64 Quality scores 0
AJ489607 consensus CTCATAAACC CAATCAAACG CCTAGCATTC GGAAGCATCT TTGCAGGATT TGTCATCTCA TATAATATTC 13158
64 Quality scores 0
AJ489607 consensus CTCATAAACC CAATCAAACG CCTAGCATTC GGAAGCATCT TTGCAGGATT TGTCATCTCA TATAATATTC 13158
64 Quality scores 0
AJ489607 consensus CTCATAAACC CAATCAAACG CCTAGCATTC GGAAGCATCT TTGCAGGATT TGTCATCTCA TATAATATTC 13158
64 Quality scores 0
AJ489607 consensus CTCATAAACC CAATCAAACG CCTAGCATTC GGAAGCATCT TTGCAGGATT TGTCATCTCA TATAATATTC 13158
64 Quality scores 0
AJ489607 consensus CTCATAAACC CAATCAAACG CCTAGCATTC GGAAGCATCT TTGCAGGATT TGTCATCTCA TATAATATTC 13158
64 Quality scores 0
Consensus CTCATAAACC CAATCAAACG CCTAGCATTC GGAAGCATCT TTGCAGGATT TGTCATCTCA TATAATATTC
100% Conservation 0%
2.0bits Sequence logo 0.0bits

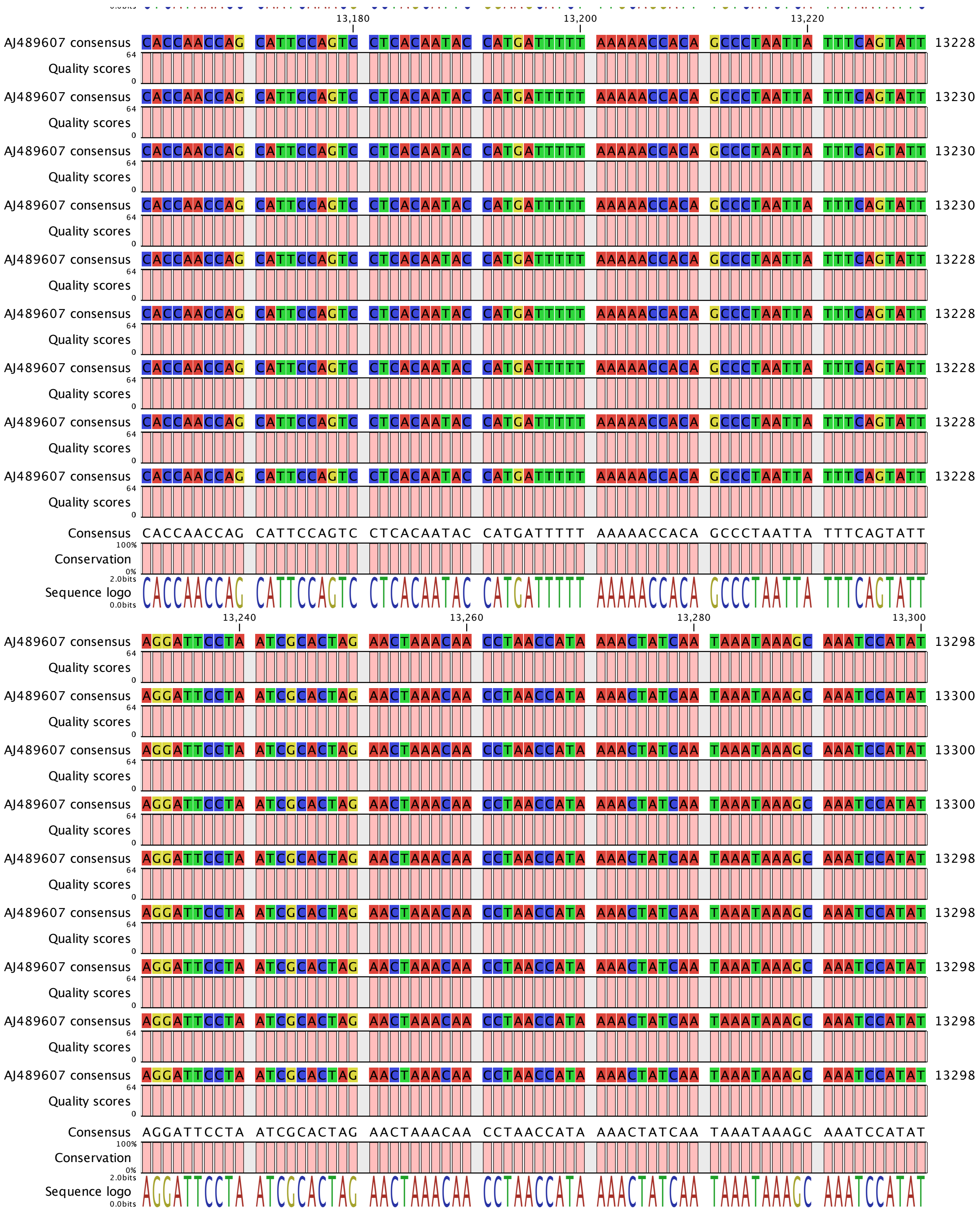
13,180 13,200 13,220
AJ489607 consensus CACCAACCAG CATTCCAGTC CTCACAATAC CATGATTTTT AAAAACCACA GCCCTAATTA TTTCAGTATT 13228
Quality scores
AJ489607 consensus CACCAACCAG CATTCCAGTC CTCACAATAC CATGATTTTT AAAAACCACA GCCCTAATTA TTTCAGTATT 13230
Quality scores
AJ489607 consensus CACCAACCAG CATTCCAGTC CTCACAATAC CATGATTTTT AAAAACCACA GCCCTAATTA TTTCAGTATT 13230
Quality scores
AJ489607 consensus CACCAACCAG CATTCCAGTC CTCACAATAC CATGATTTTT AAAAACCACA GCCCTAATTA TTTCAGTATT 13230
Quality scores
AJ489607 consensus CACCAACCAG CATTCCAGTC CTCACAATAC CATGATTTTT AAAAACCACA GCCCTAATTA TTTCAGTATT 13228
Quality scores
AJ489607 consensus CACCAACCAG CATTCCAGTC CTCACAATAC CATGATTTTT AAAAACCACA GCCCTAATTA TTTCAGTATT 13228
Quality scores
AJ489607 consensus CACCAACCAG CATTCCAGTC CTCACAATAC CATGATTTTT AAAAACCACA GCCCTAATTA TTTCAGTATT 13228
Quality scores
AJ489607 consensus CACCAACCAG CATTCCAGTC CTCACAATAC CATGATTTTT AAAAACCACA GCCCTAATTA TTTCAGTATT 13228
Quality scores
AJ489607 consensus CACCAACCAG CATTCCAGTC CTCACAATAC CATGATTTTT AAAAACCACA GCCCTAATTA TTTCAGTATT 13228
Quality scores
Consensus CACCAACCAG CATTCCAGTC CTCACAATAC CATGATTTTT AAAAACCACA GCCCTAATTA TTTCAGTATT
Conservation
Sequence logo
13,240 13,260 13,280 13,300
AJ489607 consensus AGGATTCCTA ATCGCACTAG AACTAAACAA CCTAACCATA AAACTATCAA TAAATAAAGC AAATCCATAT 13298
Quality scores
AJ489607 consensus AGGATTCCTA ATCGCACTAG AACTAAACAA CCTAACCATA AAACTATCAA TAAATAAAGC AAATCCATAT 13300
Quality scores
AJ489607 consensus AGGATTCCTA ATCGCACTAG AACTAAACAA CCTAACCATA AAACTATCAA TAAATAAAGC AAATCCATAT 13300
Quality scores
AJ489607 consensus AGGATTCCTA ATCGCACTAG AACTAAACAA CCTAACCATA AAACTATCAA TAAATAAAGC AAATCCATAT 13300
Quality scores
AJ489607 consensus AGGATTCCTA ATCGCACTAG AACTAAACAA CCTAACCATA AAACTATCAA TAAATAAAGC AAATCCATAT 13298
Quality scores
AJ489607 consensus AGGATTCCTA ATCGCACTAG AACTAAACAA CCTAACCATA AAACTATCAA TAAATAAAGC AAATCCATAT 13298
Quality scores
AJ489607 consensus AGGATTCCTA ATCGCACTAG AACTAAACAA CCTAACCATA AAACTATCAA TAAATAAAGC AAATCCATAT 13298
Quality scores
AJ489607 consensus AGGATTCCTA ATCGCACTAG AACTAAACAA CCTAACCATA AAACTATCAA TAAATAAAGC AAATCCATAT 13298
Quality scores
AJ489607 consensus AGGATTCCTA ATCGCACTAG AACTAAACAA CCTAACCATA AAACTATCAA TAAATAAAGC AAATCCATAT 13298
Quality scores
Consensus AGGATTCCTA ATCGCACTAG AACTAAACAA CCTAACCATA AAACTATCAA TAAATAAAGC AAATCCATAT
Conservation
Sequence logo

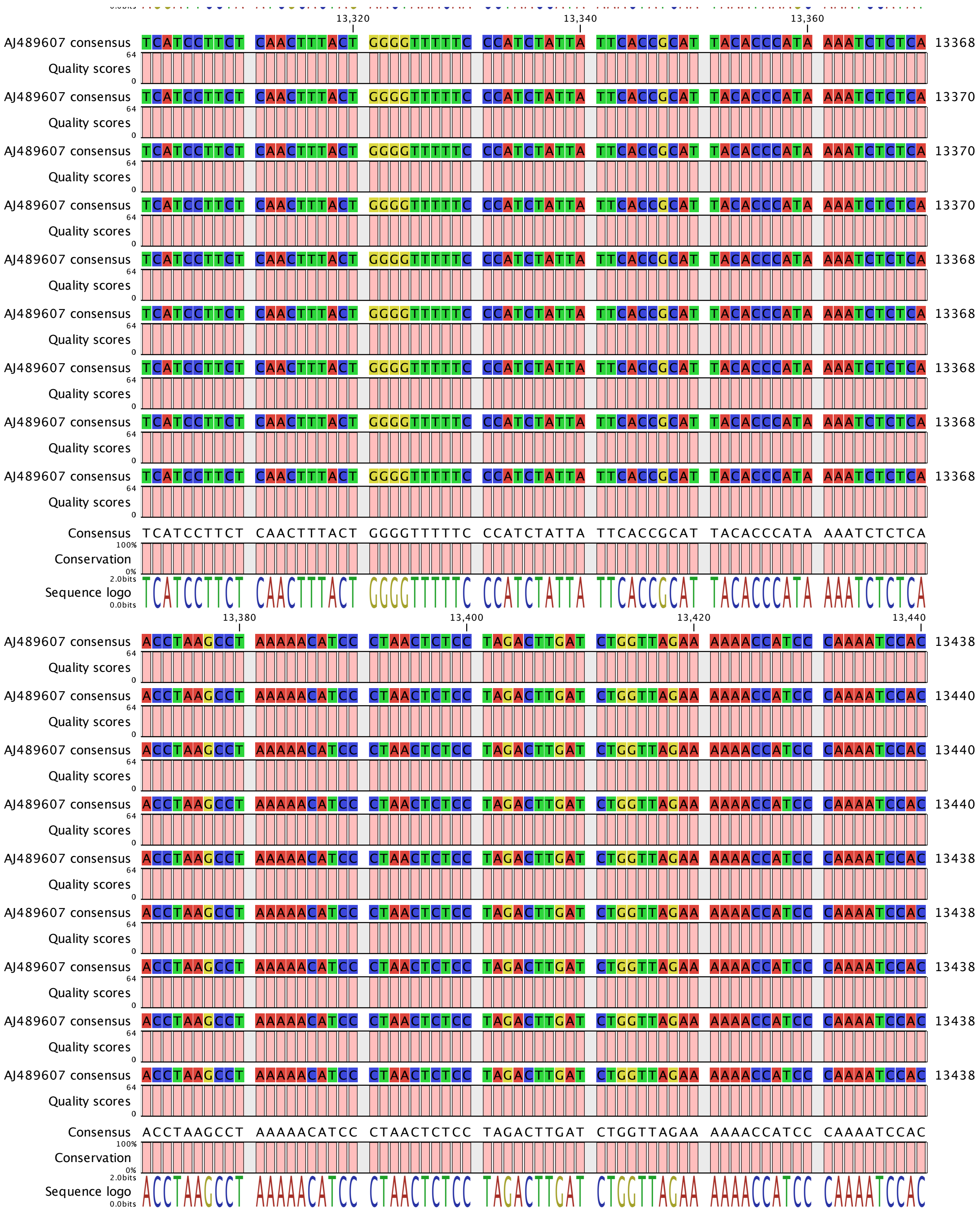
13,320 13,340 13,360
AJ489607 consensus TCATCCTTCT CAACTTTACT GGGGTTTTTC CCATCTATTA TTCACCGCAT TACACCCATA AAATCTCTCA 13368
Quality scores
AJ489607 consensus TCATCCTTCT CAACTTTACT GGGGTTTTTC CCATCTATTA TTCACCGCAT TACACCCATA AAATCTCTCA 13370
Quality scores
AJ489607 consensus TCATCCTTCT CAACTTTACT GGGGTTTTTC CCATCTATTA TTCACCGCAT TACACCCATA AAATCTCTCA 13370
Quality scores
AJ489607 consensus TCATCCTTCT CAACTTTACT GGGGTTTTTC CCATCTATTA TTCACCGCAT TACACCCATA AAATCTCTCA 13370
Quality scores
AJ489607 consensus TCATCCTTCT CAACTTTACT GGGGTTTTTC CCATCTATTA TTCACCGCAT TACACCCATA AAATCTCTCA 13368
Quality scores
AJ489607 consensus TCATCCTTCT CAACTTTACT GGGGTTTTTC CCATCTATTA TTCACCGCAT TACACCCATA AAATCTCTCA 13368
Quality scores
AJ489607 consensus TCATCCTTCT CAACTTTACT GGGGTTTTTC CCATCTATTA TTCACCGCAT TACACCCATA AAATCTCTCA 13368
Quality scores
AJ489607 consensus TCATCCTTCT CAACTTTACT GGGGTTTTTC CCATCTATTA TTCACCGCAT TACACCCATA AAATCTCTCA 13368
Quality scores
AJ489607 consensus TCATCCTTCT CAACTTTACT GGGGTTTTTC CCATCTATTA TTCACCGCAT TACACCCATA AAATCTCTCA 13368
Quality scores
Consensus TCATCCTTCT CAACTTTACT GGGGTTTTTC CCATCTATTA TTCACCGCAT TACACCCATA AAATCTCTCA
Conservation
Sequence logo
13,380 13,400 13,420 13,440
AJ489607 consensus ACCTAAGCCT AAAAACATCC CTAACTCTCC TAGACTTGAT CTGGTTAGAA AAAACCATCC CAAAATCCAC 13438
Quality scores
AJ489607 consensus ACCTAAGCCT AAAAACATCC CTAACTCTCC TAGACTTGAT CTGGTTAGAA AAAACCATCC CAAAATCCAC 13440
Quality scores
AJ489607 consensus ACCTAAGCCT AAAAACATCC CTAACTCTCC TAGACTTGAT CTGGTTAGAA AAAACCATCC CAAAATCCAC 13440
Quality scores
AJ489607 consensus ACCTAAGCCT AAAAACATCC CTAACTCTCC TAGACTTGAT CTGGTTAGAA AAAACCATCC CAAAATCCAC 13440
Quality scores
AJ489607 consensus ACCTAAGCCT AAAAACATCC CTAACTCTCC TAGACTTGAT CTGGTTAGAA AAAACCATCC CAAAATCCAC 13438
Quality scores
AJ489607 consensus ACCTAAGCCT AAAAACATCC CTAACTCTCC TAGACTTGAT CTGGTTAGAA AAAACCATCC CAAAATCCAC 13438
Quality scores
AJ489607 consensus ACCTAAGCCT AAAAACATCC CTAACTCTCC TAGACTTGAT CTGGTTAGAA AAAACCATCC CAAAATCCAC 13438
Quality scores
AJ489607 consensus ACCTAAGCCT AAAAACATCC CTAACTCTCC TAGACTTGAT CTGGTTAGAA AAAACCATCC CAAAATCCAC 13438
Quality scores
AJ489607 consensus ACCTAAGCCT AAAAACATCC CTAACTCTCC TAGACTTGAT CTGGTTAGAA AAAACCATCC CAAAATCCAC 13438
Quality scores
Consensus ACCTAAGCCT AAAAACATCC CTAACTCTCC TAGACTTGAT CTGGTTAGAA AAAACCATCC CAAAATCCAC
Conservation
Sequence logo

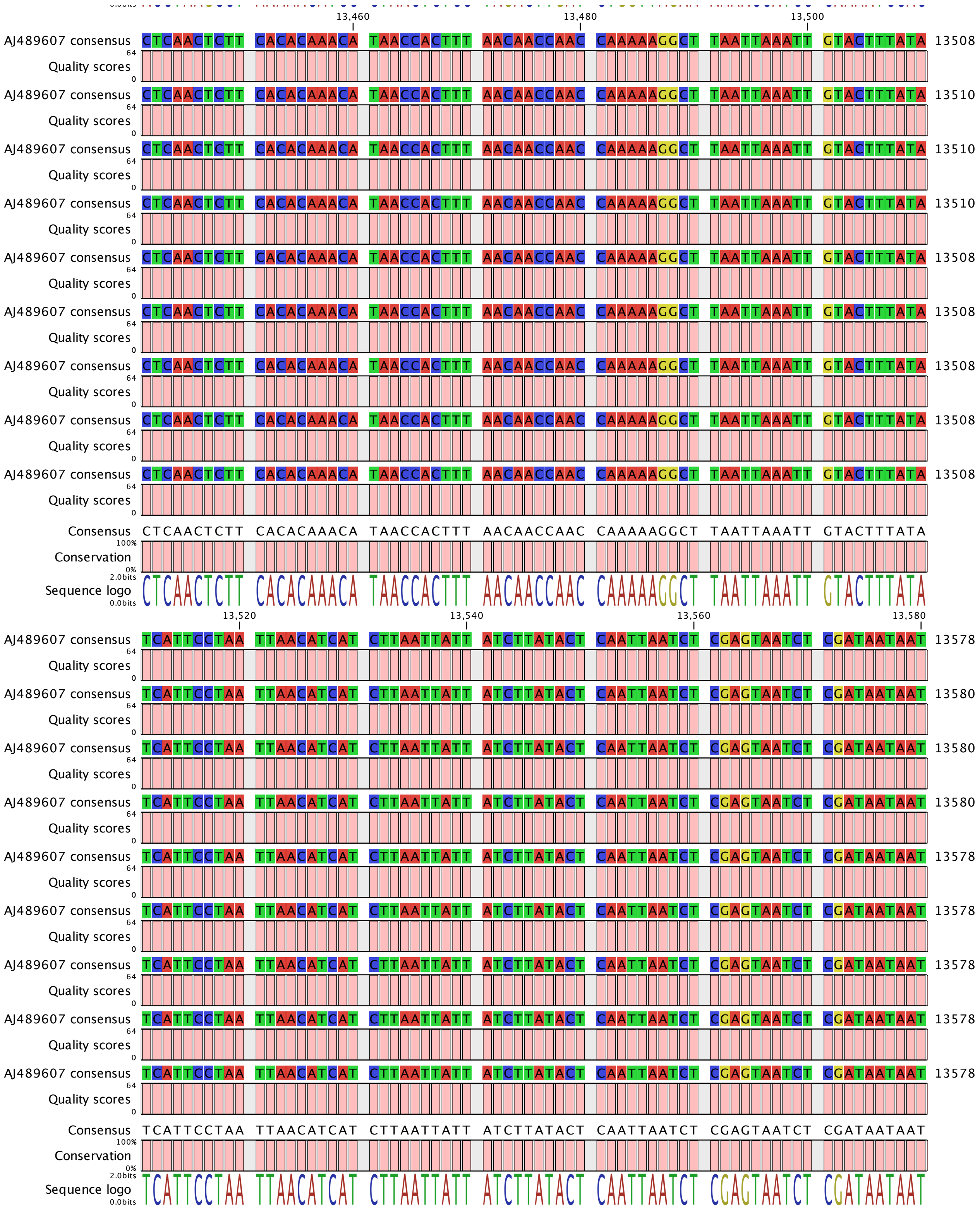
13,460
13,480
13,500
AJ489607 consensus CTCAACTCTT CACACAAACA TAACCACTTT AACAACCAAC CAAAAAGGCT TAATTAAATT GTACTTTATA 13508
Quality scores
AJ489607 consensus CTCAACTCTT CACACAAACA TAACCACTTT AACAACCAAC CAAAAAGGCT TAATTAAATT GTACTTTATA 13510
Quality scores
AJ489607 consensus CTCAACTCTT CACACAAACA TAACCACTTT AACAACCAAC CAAAAAGGCT TAATTAAATT GTACTTTATA 13510
Quality scores
AJ489607 consensus CTCAACTCTT CACACAAACA TAACCACTTT AACAACCAAC CAAAAAGGCT TAATTAAATT GTACTTTATA 13510
Quality scores
AJ489607 consensus CTCAACTCTT CACACAAACA TAACCACTTT AACAACCAAC CAAAAAGGCT TAATTAAATT GTACTTTATA 13508
Quality scores
AJ489607 consensus CTCAACTCTT CACACAAACA TAACCACTTT AACAACCAAC CAAAAAGGCT TAATTAAATT GTACTTTATA 13508
Quality scores
AJ489607 consensus CTCAACTCTT CACACAAACA TAACCACTTT AACAACCAAC CAAAAAGGCT TAATTAAATT GTACTTTATA 13508
Quality scores
AJ489607 consensus CTCAACTCTT CACACAAACA TAACCACTTT AACAACCAAC CAAAAAGGCT TAATTAAATT GTACTTTATA 13508
Quality scores
AJ489607 consensus CTCAACTCTT CACACAAACA TAACCACTTT AACAACCAAC CAAAAAGGCT TAATTAAATT GTACTTTATA 13508
Quality scores
Consensus CTCAACTCTT CACACAAACA TAACCACTTT AACAACCAAC CAAAAAGGCT TAATTAAATT GTACTTTATA
Conservation
Sequence logo
13,520
13,540
13,560
13,580
AJ489607 consensus TCATTCCTAA TTAACATCAT CTTAATTATT ATCTTATACT CAATTAATCT CGAGTAATCT CGATAATAAT 13578
Quality scores
AJ489607 consensus TCATTCCTAA TTAACATCAT CTTAATTATT ATCTTATACT CAATTAATCT CGAGTAATCT CGATAATAAT 13580
Quality scores
AJ489607 consensus TCATTCCTAA TTAACATCAT CTTAATTATT ATCTTATACT CAATTAATCT CGAGTAATCT CGATAATAAT 13580
Quality scores
AJ489607 consensus TCATTCCTAA TTAACATCAT CTTAATTATT ATCTTATACT CAATTAATCT CGAGTAATCT CGATAATAAT 13580
Quality scores
AJ489607 consensus TCATTCCTAA TTAACATCAT CTTAATTATT ATCTTATACT CAATTAATCT CGAGTAATCT CGATAATAAT 13578
Quality scores
AJ489607 consensus TCATTCCTAA TTAACATCAT CTTAATTATT ATCTTATACT CAATTAATCT CGAGTAATCT CGATAATAAT 13578
Quality scores
AJ489607 consensus TCATTCCTAA TTAACATCAT CTTAATTATT ATCTTATACT CAATTAATCT CGAGTAATCT CGATAATAAT 13578
Quality scores
AJ489607 consensus TCATTCCTAA TTAACATCAT CTTAATTATT ATCTTATACT CAATTAATCT CGAGTAATCT CGATAATAAT 13578
Quality scores
AJ489607 consensus TCATTCCTAA TTAACATCAT CTTAATTATT ATCTTATACT CAATTAATCT CGAGTAATCT CGATAATAAT 13578
Quality scores
Consensus TCATTCCTAA TTAACATCAT CTTAATTATT ATCTTATACT CAATTAATCT CGAGTAATCT CGATAATAAT
Conservation
Sequence logo

0.0bits

13,600 13,620 13,640

AJ489607 consensus AAAAATACCC GCAAACAAAG ATCACCCAGC TACTACCATC ATTCAAGTAG CACAACTATA TATTGCCGCT 13648
Quality scores 64 0
AJ489607 consensus AAAAATACCC GCAAACAAAG ATCACCCAGC TACTACCATC ATTCAAGTAG CACAACTATA TATTGCCGCT 13650
Quality scores 64 0
AJ489607 consensus AAAAATACCC GCAAACAAAG ATCACCCAGC TACTACCATC ATTCAAGTAG CACAACTATA TATTGCCGCT 13650
Quality scores 64 0
AJ489607 consensus AAAAATACCC GCAAACAAAG ATCACCCAGC TACTACCATC ATTCAAGTAG CACAACTATA TATTGCCGCT 13650
Quality scores 64 0
AJ489607 consensus AAAAATACCC GCAAACAAAG ATCACCCAGC TACTACCATC ATTCAAGTAG CACAACTATA TATTGCCGCT 13648
Quality scores 64 0
AJ489607 consensus AAAAATACCC GCAAACAAAG ATCACCCAGC TACTACCATC ATTCAAGTAG CACAACTATA TATTGCCGCT 13648
Quality scores 64 0
AJ489607 consensus AAAAATACCC GCAAACAAAG ATCACCCAGC TACTACCATC ATTCAAGTAG CACAACTATA TATTGCCGCT 13648
Quality scores 64 0
AJ489607 consensus AAAAATACCC GCAAACAAAG ATCACCCAGC TACTACCATC ATTCAAGTAG CACAACTATA TATTGCCGCT 13648
Quality scores 64 0
AJ489607 consensus AAAAATACCC GCAAACAAAG ATCACCCAGC TACTACCATC ATTCAAGTAG CACAACTATA TATTGCCGCT 13648
Quality scores 64 0
Consensus AAAAATACCC GCAAACAAAG ATCACCCAGC TACTACCATC ATTCAAGTAG CACAACTATA TATTGCCGCT
Conservation 100% 0%
Sequence logo 2.0bits 0.0bits AAAAATACCC GCAAACAAAG ATCACCCAGC TACTACCATC ATTCAAGTAG CACAACTATA TATTGCCGCT

13,660 13,680 13,700 13,720

AJ489607 consensus ACCCCAATCC CTCCTTCCAA CATAACTCCA ACATCATCAA CCTCATACAT CAACCAATCT CCCAAACCAT 13718
Quality scores 64 0
AJ489607 consensus ACCCCAATCC CTCCTTCCAA CATAACTCCA ACATCATCAA CCTCATACAT CAACCAATCT CCCAAACCAT 13720
Quality scores 64 0
AJ489607 consensus ACCCCAATCC CTCCTTCCAA CATAACTCCA ACATCATCAA CCTCATACAT CAACCAATCT CCCAAACCAT 13720
Quality scores 64 0
AJ489607 consensus ACCCCAATCC CTCCTTCCAA CATAACTCCA ACATCATCAA CCTCATACAT CAACCAATCT CCCAAACCAT 13720
Quality scores 64 0
AJ489607 consensus ACCCCAATCC CTCCTTCCAA CATAACTCCA ACATCATCAA CCTCATACAT CAACCAATCT CCCAAACCAT 13718
Quality scores 64 0
AJ489607 consensus ACCCCAATCC CTCCTTCCAA CATAACTCCA ACATCATCAA CCTCATACAT CAACCAATCT CCCAAACCAT 13718
Quality scores 64 0
AJ489607 consensus ACCCCAATCC CTCCTTCCAA CATAACTCCA ACATCATCAA CCTCATACAT CAACCAATCT CCCAAACCAT 13718
Quality scores 64 0
AJ489607 consensus ACCCCAATCC CTCCTTCCAA CATAACTCCA ACATCATCAA CCTCATACAT CAACCAATCT CCCAAACCAT 13718
Quality scores 64 0
AJ489607 consensus ACCCCAATCC CTCCTTCCAA CATAACTCCA ACATCATCAA CCTCATACAT CAACCAATCT CCCAAACCAT 13718
Quality scores 64 0
Consensus ACCCCAATCC CTCCTTCCAA CATAACTCCA ACATCATCAA CCTCATACAT CAACCAATCT CCCAAACCAT
Conservation 100% 0%
Sequence logo 2.0bits 0.0bits ACCCCAATCC CTCCTTCCAA CATAACTCCA ACATCATCAA CCTCATACAT CAACCAATCT CCCAAACCAT

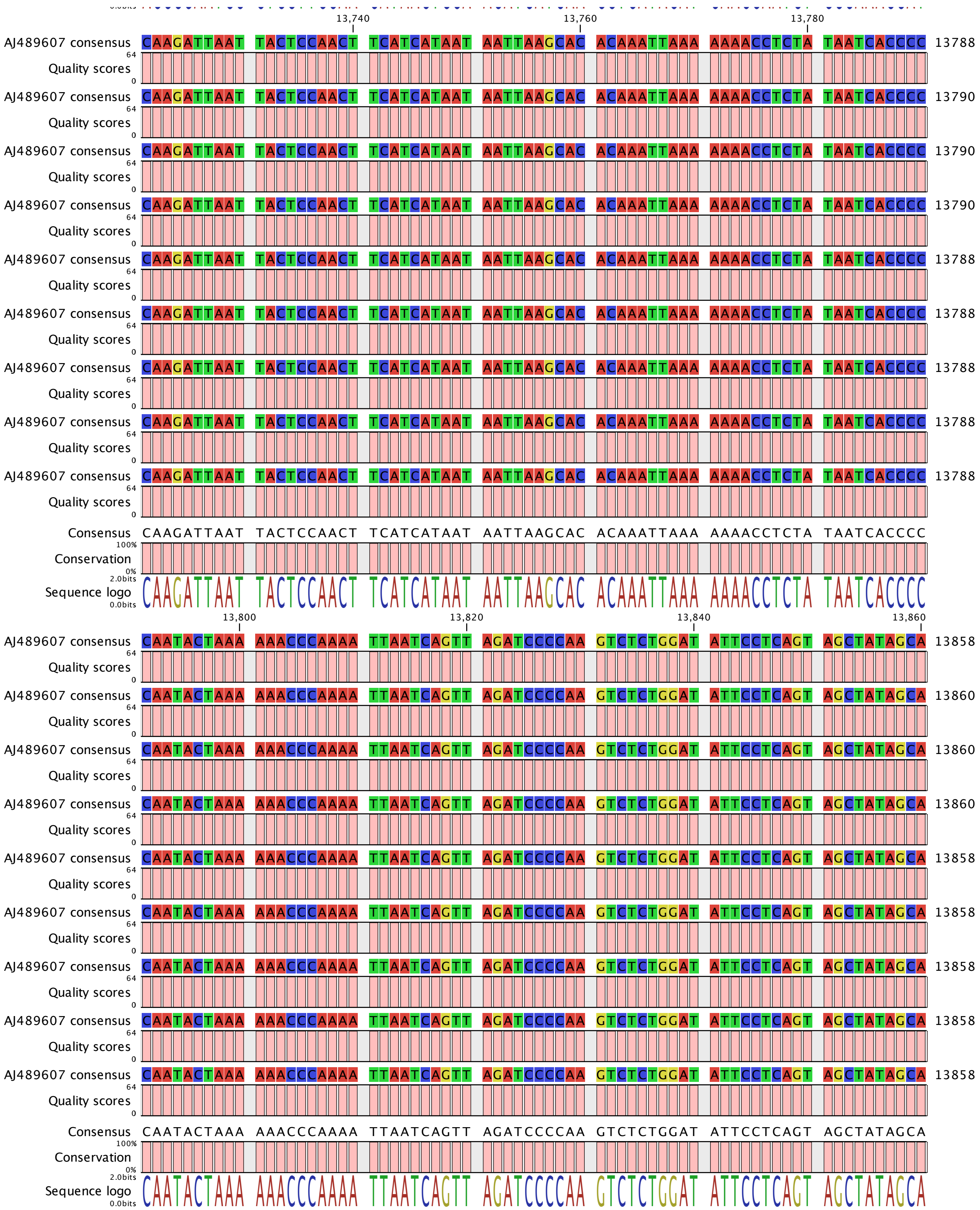

13,740
13,760
13,780
AJ489607 consensus CAAGATTAAT TACTCCAACT TCATCATAAT AATTAAGCAC ACAAATTAAA AAAACCTCTA TAATCACCCC 13788
Quality scores
AJ489607 consensus CAAGATTAAT TACTCCAACT TCATCATAAT AATTAAGCAC ACAAATTAAA AAAACCTCTA TAATCACCCC 13790
Quality scores
AJ489607 consensus CAAGATTAAT TACTCCAACT TCATCATAAT AATTAAGCAC ACAAATTAAA AAAACCTCTA TAATCACCCC 13790
Quality scores
AJ489607 consensus CAAGATTAAT TACTCCAACT TCATCATAAT AATTAAGCAC ACAAATTAAA AAAACCTCTA TAATCACCCC 13790
Quality scores
AJ489607 consensus CAAGATTAAT TACTCCAACT TCATCATAAT AATTAAGCAC ACAAATTAAA AAAACCTCTA TAATCACCCC 13788
Quality scores
AJ489607 consensus CAAGATTAAT TACTCCAACT TCATCATAAT AATTAAGCAC ACAAATTAAA AAAACCTCTA TAATCACCCC 13788
Quality scores
AJ489607 consensus CAAGATTAAT TACTCCAACT TCATCATAAT AATTAAGCAC ACAAATTAAA AAAACCTCTA TAATCACCCC 13788
Quality scores
AJ489607 consensus CAAGATTAAT TACTCCAACT TCATCATAAT AATTAAGCAC ACAAATTAAA AAAACCTCTA TAATCACCCC 13788
Quality scores
AJ489607 consensus CAAGATTAAT TACTCCAACT TCATCATAAT AATTAAGCAC ACAAATTAAA AAAACCTCTA TAATCACCCC 13788
Quality scores
Consensus CAAGATTAAT TACTCCAACT TCATCATAAT AATTAAGCAC ACAAATTAAA AAAACCTCTA TAATCACCCC
Conservation
Sequence logo
13,800
13,820
13,840
13,860
AJ489607 consensus CAATACTAAA AAACCCAAAA TTAATCAGTT AGATCCCCAA GTCTCTGGAT ATTCCTCAGT AGCTATAGCA 13858
Quality scores
AJ489607 consensus CAATACTAAA AAACCCAAAA TTAATCAGTT AGATCCCCAA GTCTCTGGAT ATTCCTCAGT AGCTATAGCA 13860
Quality scores
AJ489607 consensus CAATACTAAA AAACCCAAAA TTAATCAGTT AGATCCCCAA GTCTCTGGAT ATTCCTCAGT AGCTATAGCA 13860
Quality scores
AJ489607 consensus CAATACTAAA AAACCCAAAA TTAATCAGTT AGATCCCCAA GTCTCTGGAT ATTCCTCAGT AGCTATAGCA 13860
Quality scores
AJ489607 consensus CAATACTAAA AAACCCAAAA TTAATCAGTT AGATCCCCAA GTCTCTGGAT ATTCCTCAGT AGCTATAGCA 13858
Quality scores
AJ489607 consensus CAATACTAAA AAACCCAAAA TTAATCAGTT AGATCCCCAA GTCTCTGGAT ATTCCTCAGT AGCTATAGCA 13858
Quality scores
AJ489607 consensus CAATACTAAA AAACCCAAAA TTAATCAGTT AGATCCCCAA GTCTCTGGAT ATTCCTCAGT AGCTATAGCA 13858
Quality scores
AJ489607 consensus CAATACTAAA AAACCCAAAA TTAATCAGTT AGATCCCCAA GTCTCTGGAT ATTCCTCAGT AGCTATAGCA 13858
Quality scores
AJ489607 consensus CAATACTAAA AAACCCAAAA TTAATCAGTT AGATCCCCAA GTCTCTGGAT ATTCCTCAGT AGCTATAGCA 13858
Quality scores
Consensus CAATACTAAA AAACCCAAAA TTAATCAGTT AGATCCCCAA GTCTCTGGAT ATTCCTCAGT AGCTATAGCA
Conservation
Sequence logo

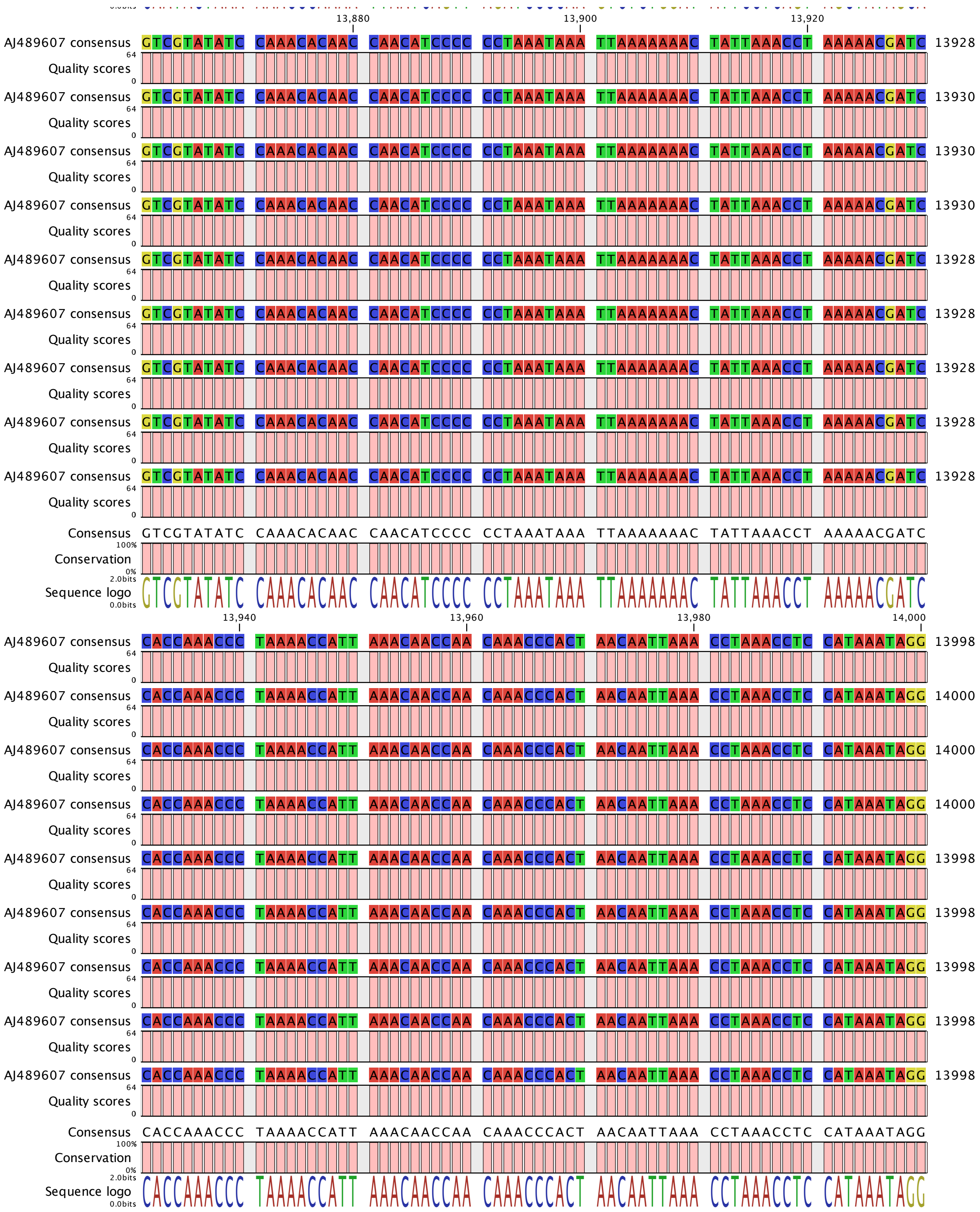
13,880
13,900
13,920
AJ489607 consensus GTCGTATATC CAAACACAAC CAACATCCCC CCTAAATAAA TTAAAAAAAC TATTAAACCT AAAAACGATC 13928
Quality scores
AJ489607 consensus GTCGTATATC CAAACACAAC CAACATCCCC CCTAAATAAA TTAAAAAAAC TATTAAACCT AAAAACGATC 13930
Quality scores
AJ489607 consensus GTCGTATATC CAAACACAAC CAACATCCCC CCTAAATAAA TTAAAAAAAC TATTAAACCT AAAAACGATC 13930
Quality scores
AJ489607 consensus GTCGTATATC CAAACACAAC CAACATCCCC CCTAAATAAA TTAAAAAAAC TATTAAACCT AAAAACGATC 13930
Quality scores
AJ489607 consensus GTCGTATATC CAAACACAAC CAACATCCCC CCTAAATAAA TTAAAAAAAC TATTAAACCT AAAAACGATC 13928
Quality scores
AJ489607 consensus GTCGTATATC CAAACACAAC CAACATCCCC CCTAAATAAA TTAAAAAAAC TATTAAACCT AAAAACGATC 13928
Quality scores
AJ489607 consensus GTCGTATATC CAAACACAAC CAACATCCCC CCTAAATAAA TTAAAAAAAC TATTAAACCT AAAAACGATC 13928
Quality scores
AJ489607 consensus GTCGTATATC CAAACACAAC CAACATCCCC CCTAAATAAA TTAAAAAAAC TATTAAACCT AAAAACGATC 13928
Quality scores
AJ489607 consensus GTCGTATATC CAAACACAAC CAACATCCCC CCTAAATAAA TTAAAAAAAC TATTAAACCT AAAAACGATC 13928
Quality scores
Consensus GTCGTATATC CAAACACAAC CAACATCCCC CCTAAATAAA TTAAAAAAAC TATTAAACCT AAAAACGATC
Conservation
Sequence logo
13,940
13,960
13,980
14,000
AJ489607 consensus CACCAAACCC TAAAACCATT AAACAACCAA CAAACCCACT AACAATTAAA CCTAAACCTC CATAAATAGG 13998
Quality scores
AJ489607 consensus CACCAAACCC TAAAACCATT AAACAACCAA CAAACCCACT AACAATTAAA CCTAAACCTC CATAAATAGG 14000
Quality scores
AJ489607 consensus CACCAAACCC TAAAACCATT AAACAACCAA CAAACCCACT AACAATTAAA CCTAAACCTC CATAAATAGG 14000
Quality scores
AJ489607 consensus CACCAAACCC TAAAACCATT AAACAACCAA CAAACCCACT AACAATTAAA CCTAAACCTC CATAAATAGG 14000
Quality scores
AJ489607 consensus CACCAAACCC TAAAACCATT AAACAACCAA CAAACCCACT AACAATTAAA CCTAAACCTC CATAAATAGG 13998
Quality scores
AJ489607 consensus CACCAAACCC TAAAACCATT AAACAACCAA CAAACCCACT AACAATTAAA CCTAAACCTC CATAAATAGG 13998
Quality scores
AJ489607 consensus CACCAAACCC TAAAACCATT AAACAACCAA CAAACCCACT AACAATTAAA CCTAAACCTC CATAAATAGG 13998
Quality scores
AJ489607 consensus CACCAAACCC TAAAACCATT AAACAACCAA CAAACCCACT AACAATTAAA CCTAAACCTC CATAAATAGG 13998
Quality scores
AJ489607 consensus CACCAAACCC TAAAACCATT AAACAACCAA CAAACCCACT AACAATTAAA CCTAAACCTC CATAAATAGG 13998
Quality scores
Consensus CACCAAACCC TAAAACCATT AAACAACCAA CAAACCCACT AACAATTAAA CCTAAACCTC CATAAATAGG
Conservation
Sequence logo

14,020 14,040 14,060

AJ489607 consensus	TGAAGGCTTT AATGCTAACC CAAGACAACC AACCAAAAAT AATGAACTTA AAACAAAAAT ATAATTATTC	14068
Quality scores (64 / 0)		
AJ489607 consensus	TGAAGGCTTT AATGCTAACC CAAGACAACC AACCAAAAAT AATGAACTTA AAACAAAAAT ATAATTATTC	14070
Quality scores (64 / 0)		
AJ489607 consensus	TGAAGGCTTT AATGCTAACC CAAGACAACC AACCAAAAAT AATGAACTTA AAACAAAAAT ATAATTATTC	14070
Quality scores (64 / 0)		
AJ489607 consensus	TGAAGGCTTT AATGCTAACC CAAGACAACC AACCAAAAAT AATGAACTTA AAACAAAAAT ATAATTATTC	14070
Quality scores (64 / 0)		
AJ489607 consensus	TGAAGGCTTT AATGCTAACC CAAGACAACC AACCAAAAAT AATGAACTTA AAACAAAAAT ATAATTATTC	14068
Quality scores (64 / 0)		
AJ489607 consensus	TGAAGGCTTT AATGCTAACC CAAGACAACC AACCAAAAAT AATGAACTTA AAACAAAAAT ATAATTATTC	14068
Quality scores (64 / 0)		
AJ489607 consensus	TGAAGGCTTT AATGCTAACC CAAGACAACC AACCAAAAAT AATGAACTTA AAACAAAAAT ATAATTATTC	14068
Quality scores (64 / 0)		
AJ489607 consensus	TGAAGGCTTT AATGCTAACC CAAGACAACC AACCAAAAAT AATGAACTTA AAACAAAAAT ATAATTATTC	14068
Quality scores (64 / 0)		
AJ489607 consensus	TGAAGGCTTT AATGCTAACC CAAGACAACC AACCAAAAAT AATGAACTTA AAACAAAAAT ATAATTATTC	14068
Quality scores (64 / 0)		
Consensus	TGAAGGCTTT AATGCTAACC CAAGACAACC AACCAAAAAT AATGAACTTA AAACAAAAAT ATAATTATTC	
Conservation (100% / 0%)		
Sequence logo (2.0bits / 0.0bits)	TGAAGGCTTT AATGCTAACC CAAGACAACC AACCAAAAAT AATGAACTTA AAACAAAAAT ATAATTATTC	

14,080 14,100 14,120 14,140

AJ489607 consensus	ATTATTTCTA CACAGCATTC AACTGCGACC AATGACATGA AAAATCATCG TTGTAATTCA ACTACAGAAA	14138
Quality scores (64 / 0)		
AJ489607 consensus	ATTATTTCTA CACAGCATTC AACTGCGACC AATGACATGA AAAATCATCG TTGTAATTCA ACTACAGAAA	14140
Quality scores (64 / 0)		
AJ489607 consensus	ATTATTTCTA CACAGCATTC AACTGCGACC AATGACATGA AAAATCATCG TTGTAATTCA ACTACAGAAA	14140
Quality scores (64 / 0)		
AJ489607 consensus	ATTATTTCTA CACAGCATTC AACTGCGACC AATGACATGA AAAATCATCG TTGTAATTCA ACTACAGAAA	14140
Quality scores (64 / 0)		
AJ489607 consensus	ATTATTTCTA CACAGCATTC AACTGCGACC AATGACATGA AAAATCATCG TTGTAATTCA ACTACAGAAA	14138
Quality scores (64 / 0)		
AJ489607 consensus	ATTATTTCTA CACAGCATTC AACTGCGACC AATGACATGA AAAATCATCG TTGTAATTCA ACTACAGAAA	14138
Quality scores (64 / 0)		
AJ489607 consensus	ATTATTTCTA CACAGCATTC AACTGCGACC AATGACATGA AAAATCATCG TTGTAATTCA ACTACAGAAA	14138
Quality scores (64 / 0)		
AJ489607 consensus	ATTATTTCTA CACAGCATTC AACTGCGACC AATGACATGA AAAATCATCG TTGTAATTCA ACTACAGAAA	14138
Quality scores (64 / 0)		
AJ489607 consensus	ATTATTTCTA CACAGCATTC AACTGCGACC AATGACATGA AAAATCATCG TTGTAATTCA ACTACAGAAA	14138
Quality scores (64 / 0)		
Consensus	ATTATTTCTA CACAGCATTC AACTGCGACC AATGACATGA AAAATCATCG TTGTAATTCA ACTACAGAAA	
Conservation (100% / 0%)		
Sequence logo (2.0bits / 0.0bits)	ATTATTTCTA CACAGCATTC AACTGCGACC AATGACATGA AAAATCATCG TTGTAATTCA ACTACAGAAA	

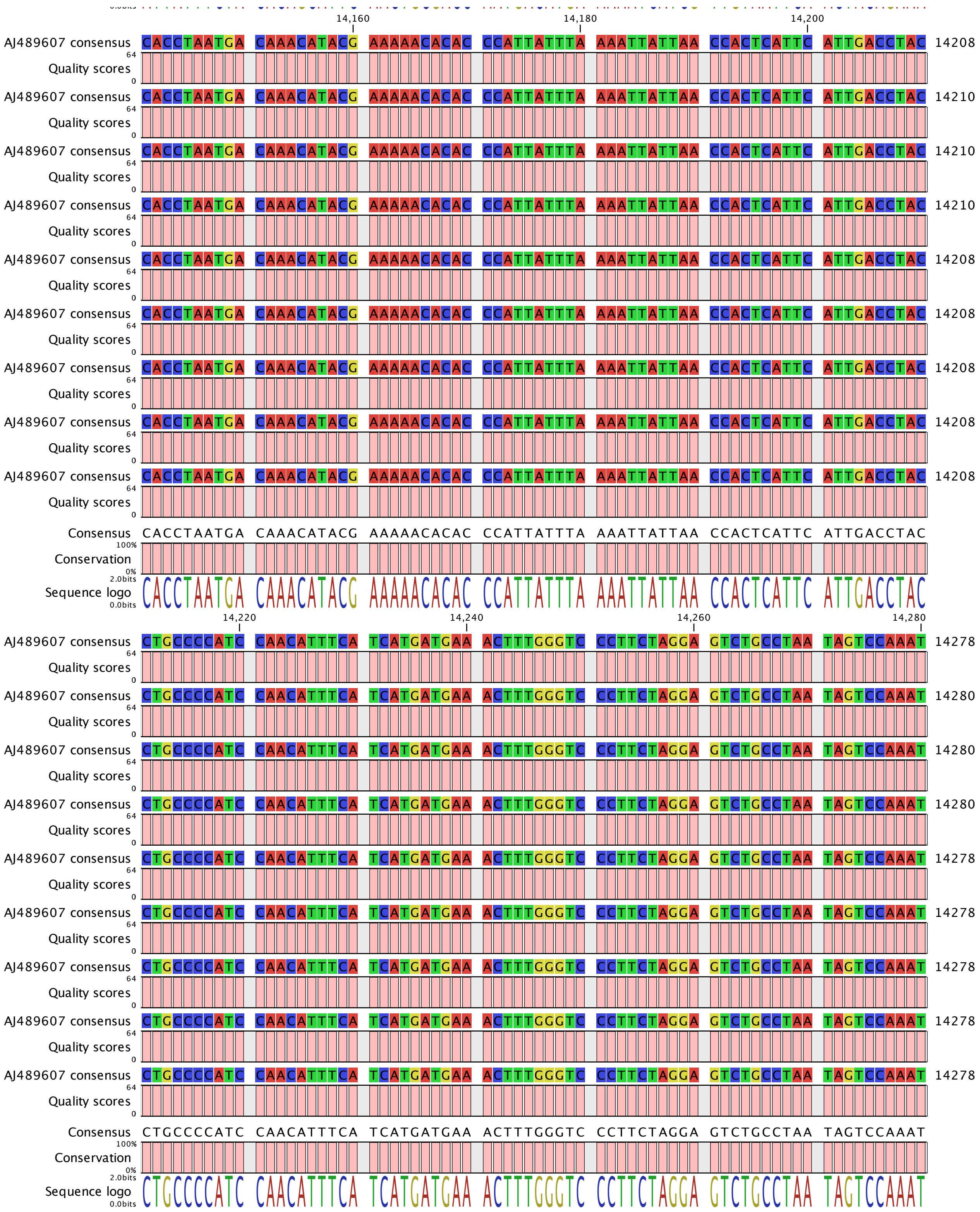
14,160
14,180
14,200
AJ489607 consensus
Quality scores
CACCTAATGA CAAACATACG AAAAACACAC CCATTATTTA AAATTATTAA CCACTCATTC ATTGACCTAC
14208
14210
14210
14210
14208
14208
14208
14208
14208
Consensus CACCTAATGA CAAACATACG AAAAACACAC CCATTATTTA AAATTATTAA CCACTCATTC ATTGACCTAC
Conservation
Sequence logo
14,220
14,240
14,260
14,280
CTGCCCCATC CAACATTTCA TCATGATGAA ACTTTGGGTC CCTTCTAGGA GTCTGCCTAA TAGTCCAAAT
14278
14280
14280
14280
14278
14278
14278
14278
14278
Consensus CTGCCCCATC CAACATTTCA TCATGATGAA ACTTTGGGTC CCTTCTAGGA GTCTGCCTAA TAGTCCAAAT
Conservation
Sequence logo

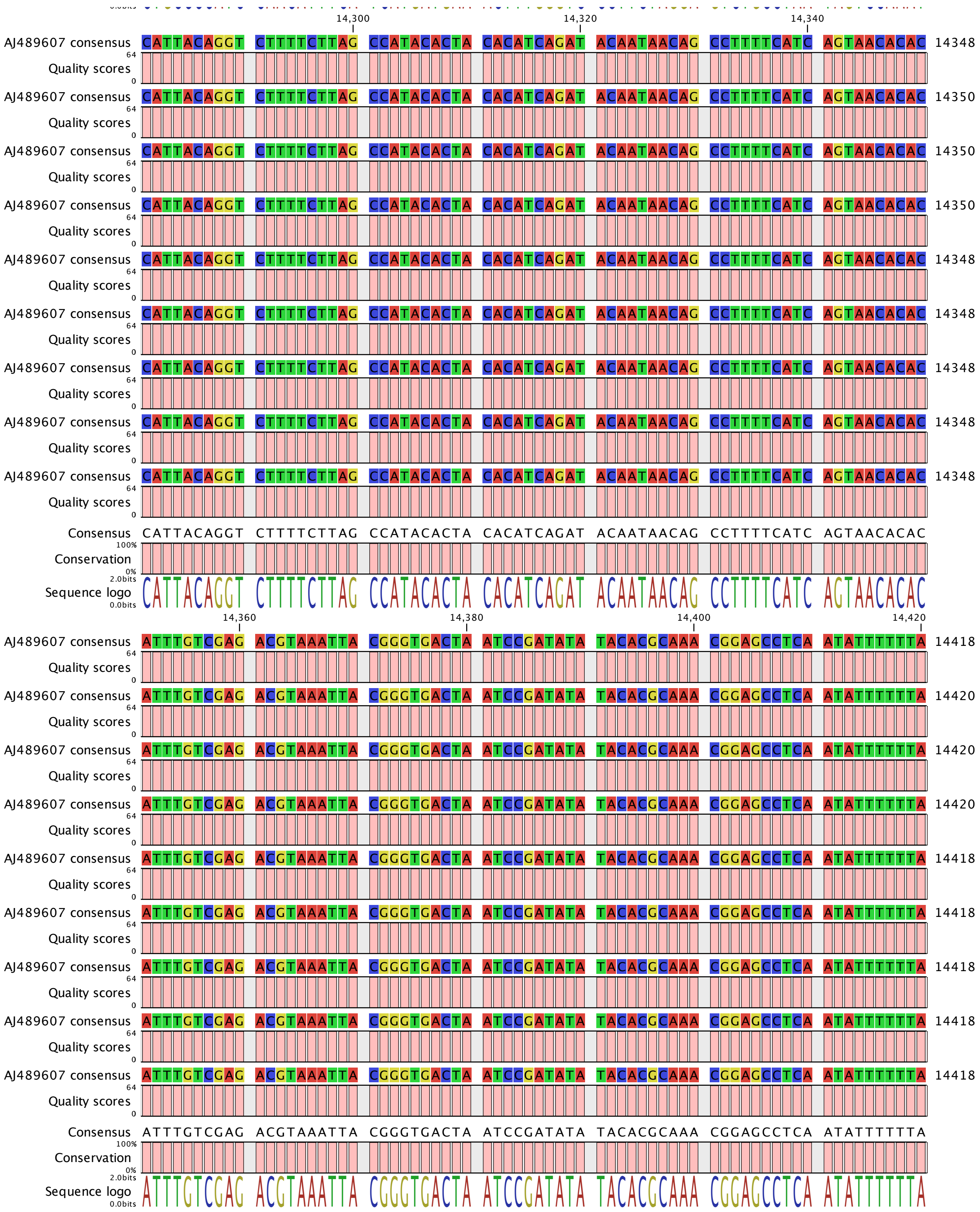

14,300
14,320
14,340
AJ489607 consensus CATTACAGGT CTTTTCTTAG CCATACACTA CACATCAGAT ACAATAACAG CCTTTTCATC AGTAACACAC 14348
64
Quality scores
0
AJ489607 consensus CATTACAGGT CTTTTCTTAG CCATACACTA CACATCAGAT ACAATAACAG CCTTTTCATC AGTAACACAC 14350
64
Quality scores
0
AJ489607 consensus CATTACAGGT CTTTTCTTAG CCATACACTA CACATCAGAT ACAATAACAG CCTTTTCATC AGTAACACAC 14350
64
Quality scores
0
AJ489607 consensus CATTACAGGT CTTTTCTTAG CCATACACTA CACATCAGAT ACAATAACAG CCTTTTCATC AGTAACACAC 14350
64
Quality scores
0
AJ489607 consensus CATTACAGGT CTTTTCTTAG CCATACACTA CACATCAGAT ACAATAACAG CCTTTTCATC AGTAACACAC 14348
64
Quality scores
0
AJ489607 consensus CATTACAGGT CTTTTCTTAG CCATACACTA CACATCAGAT ACAATAACAG CCTTTTCATC AGTAACACAC 14348
64
Quality scores
0
AJ489607 consensus CATTACAGGT CTTTTCTTAG CCATACACTA CACATCAGAT ACAATAACAG CCTTTTCATC AGTAACACAC 14348
64
Quality scores
0
AJ489607 consensus CATTACAGGT CTTTTCTTAG CCATACACTA CACATCAGAT ACAATAACAG CCTTTTCATC AGTAACACAC 14348
64
Quality scores
0
AJ489607 consensus CATTACAGGT CTTTTCTTAG CCATACACTA CACATCAGAT ACAATAACAG CCTTTTCATC AGTAACACAC 14348
64
Quality scores
0
Consensus CATTACAGGT CTTTTCTTAG CCATACACTA CACATCAGAT ACAATAACAG CCTTTTCATC AGTAACACAC
100%
Conservation
0%
2.0bits
Sequence logo
0.0bits
14,360
14,380
14,400
14,420
AJ489607 consensus ATTTGTCGAG ACGTAAATTA CGGGTGACTA ATCCGATATA TACACGCAAA CGGAGCCTCA ATATTTTTTA 14418
64
Quality scores
0
AJ489607 consensus ATTTGTCGAG ACGTAAATTA CGGGTGACTA ATCCGATATA TACACGCAAA CGGAGCCTCA ATATTTTTTA 14420
64
Quality scores
0
AJ489607 consensus ATTTGTCGAG ACGTAAATTA CGGGTGACTA ATCCGATATA TACACGCAAA CGGAGCCTCA ATATTTTTTA 14420
64
Quality scores
0
AJ489607 consensus ATTTGTCGAG ACGTAAATTA CGGGTGACTA ATCCGATATA TACACGCAAA CGGAGCCTCA ATATTTTTTA 14420
64
Quality scores
0
AJ489607 consensus ATTTGTCGAG ACGTAAATTA CGGGTGACTA ATCCGATATA TACACGCAAA CGGAGCCTCA ATATTTTTTA 14418
64
Quality scores
0
AJ489607 consensus ATTTGTCGAG ACGTAAATTA CGGGTGACTA ATCCGATATA TACACGCAAA CGGAGCCTCA ATATTTTTTA 14418
64
Quality scores
0
AJ489607 consensus ATTTGTCGAG ACGTAAATTA CGGGTGACTA ATCCGATATA TACACGCAAA CGGAGCCTCA ATATTTTTTA 14418
64
Quality scores
0
AJ489607 consensus ATTTGTCGAG ACGTAAATTA CGGGTGACTA ATCCGATATA TACACGCAAA CGGAGCCTCA ATATTTTTTA 14418
64
Quality scores
0
AJ489607 consensus ATTTGTCGAG ACGTAAATTA CGGGTGACTA ATCCGATATA TACACGCAAA CGGAGCCTCA ATATTTTTTA 14418
64
Quality scores
0
Consensus ATTTGTCGAG ACGTAAATTA CGGGTGACTA ATCCGATATA TACACGCAAA CGGAGCCTCA ATATTTTTTA
100%
Conservation
0%
2.0bits
Sequence logo
0.0bits

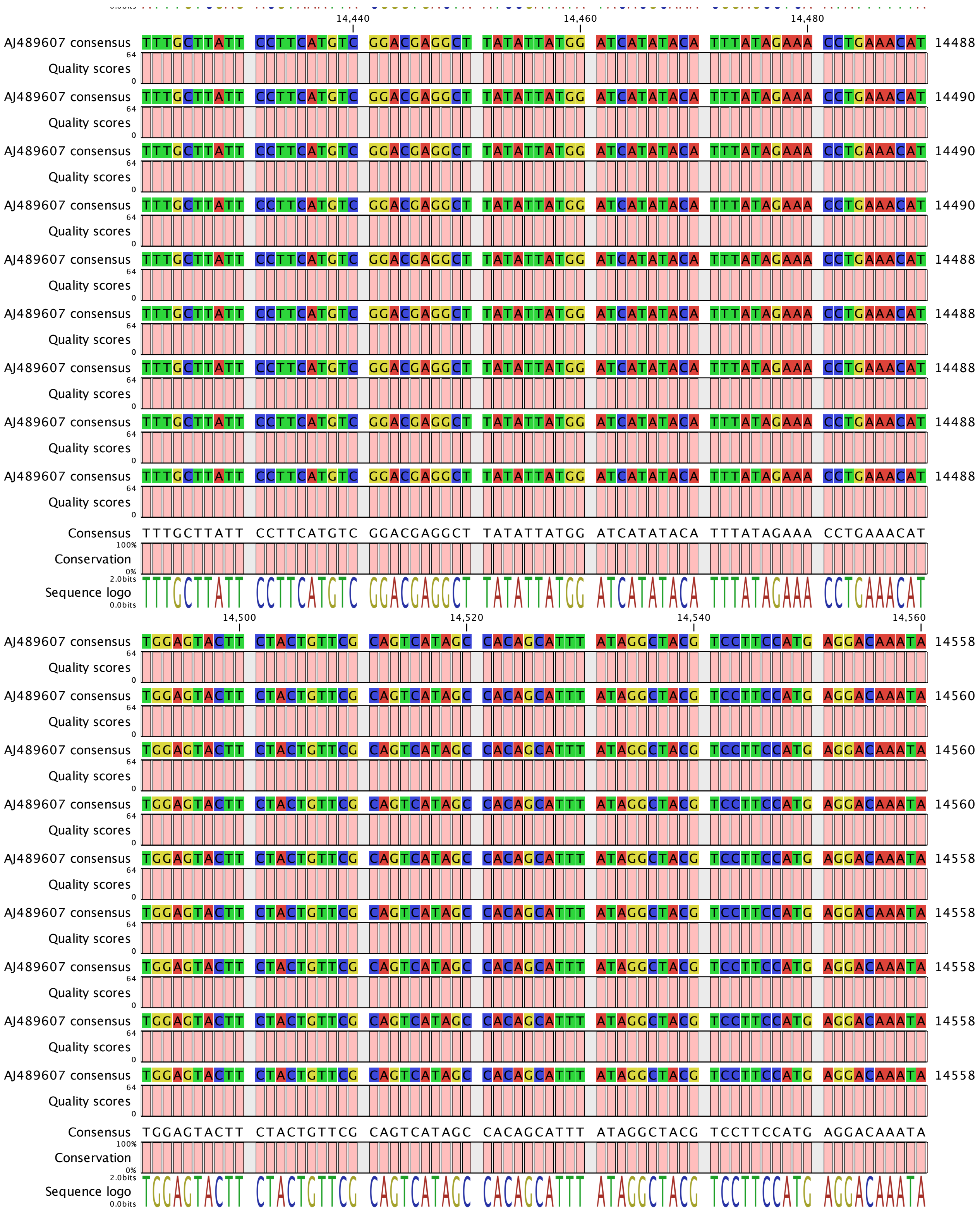
14,440
14,460
14,480
AJ489607 consensus TTTGCTTATT CCTTCATGTC GGACGAGGCT TATATTATGG ATCATATACA TTTATAGAAA CCTGAAACAT 14488
Quality scores 64 0
AJ489607 consensus TTTGCTTATT CCTTCATGTC GGACGAGGCT TATATTATGG ATCATATACA TTTATAGAAA CCTGAAACAT 14490
Quality scores 64 0
AJ489607 consensus TTTGCTTATT CCTTCATGTC GGACGAGGCT TATATTATGG ATCATATACA TTTATAGAAA CCTGAAACAT 14490
Quality scores 64 0
AJ489607 consensus TTTGCTTATT CCTTCATGTC GGACGAGGCT TATATTATGG ATCATATACA TTTATAGAAA CCTGAAACAT 14490
Quality scores 64 0
AJ489607 consensus TTTGCTTATT CCTTCATGTC GGACGAGGCT TATATTATGG ATCATATACA TTTATAGAAA CCTGAAACAT 14488
Quality scores 64 0
AJ489607 consensus TTTGCTTATT CCTTCATGTC GGACGAGGCT TATATTATGG ATCATATACA TTTATAGAAA CCTGAAACAT 14488
Quality scores 64 0
AJ489607 consensus TTTGCTTATT CCTTCATGTC GGACGAGGCT TATATTATGG ATCATATACA TTTATAGAAA CCTGAAACAT 14488
Quality scores 64 0
AJ489607 consensus TTTGCTTATT CCTTCATGTC GGACGAGGCT TATATTATGG ATCATATACA TTTATAGAAA CCTGAAACAT 14488
Quality scores 64 0
AJ489607 consensus TTTGCTTATT CCTTCATGTC GGACGAGGCT TATATTATGG ATCATATACA TTTATAGAAA CCTGAAACAT 14488
Quality scores 64 0
Consensus TTTGCTTATT CCTTCATGTC GGACGAGGCT TATATTATGG ATCATATACA TTTATAGAAA CCTGAAACAT
Conservation 100% 0%
Sequence logo 2.0bits 0.0bits
TTTGCTTATT CCTTCATGTC GGACGAGGCT TATATTATGG ATCATATACA TTTATAGAAA CCTGAAACAT
14,500
14,520
14,540
14,560
AJ489607 consensus TGGAGTACTT CTACTGTTCG CAGTCATAGC CACAGCATTT ATAGGCTACG TCCTTCCATG AGGACAAATA 14558
Quality scores 64 0
AJ489607 consensus TGGAGTACTT CTACTGTTCG CAGTCATAGC CACAGCATTT ATAGGCTACG TCCTTCCATG AGGACAAATA 14560
Quality scores 64 0
AJ489607 consensus TGGAGTACTT CTACTGTTCG CAGTCATAGC CACAGCATTT ATAGGCTACG TCCTTCCATG AGGACAAATA 14560
Quality scores 64 0
AJ489607 consensus TGGAGTACTT CTACTGTTCG CAGTCATAGC CACAGCATTT ATAGGCTACG TCCTTCCATG AGGACAAATA 14560
Quality scores 64 0
AJ489607 consensus TGGAGTACTT CTACTGTTCG CAGTCATAGC CACAGCATTT ATAGGCTACG TCCTTCCATG AGGACAAATA 14558
Quality scores 64 0
AJ489607 consensus TGGAGTACTT CTACTGTTCG CAGTCATAGC CACAGCATTT ATAGGCTACG TCCTTCCATG AGGACAAATA 14558
Quality scores 64 0
AJ489607 consensus TGGAGTACTT CTACTGTTCG CAGTCATAGC CACAGCATTT ATAGGCTACG TCCTTCCATG AGGACAAATA 14558
Quality scores 64 0
AJ489607 consensus TGGAGTACTT CTACTGTTCG CAGTCATAGC CACAGCATTT ATAGGCTACG TCCTTCCATG AGGACAAATA 14558
Quality scores 64 0
AJ489607 consensus TGGAGTACTT CTACTGTTCG CAGTCATAGC CACAGCATTT ATAGGCTACG TCCTTCCATG AGGACAAATA 14558
Quality scores 64 0
Consensus TGGAGTACTT CTACTGTTCG CAGTCATAGC CACAGCATTT ATAGGCTACG TCCTTCCATG AGGACAAATA
Conservation 100% 0%
Sequence logo 2.0bits 0.0bits
TGGAGTACTT CTACTGTTCG CAGTCATAGC CACAGCATTT ATAGGCTACG TCCTTCCATG AGGACAAATA

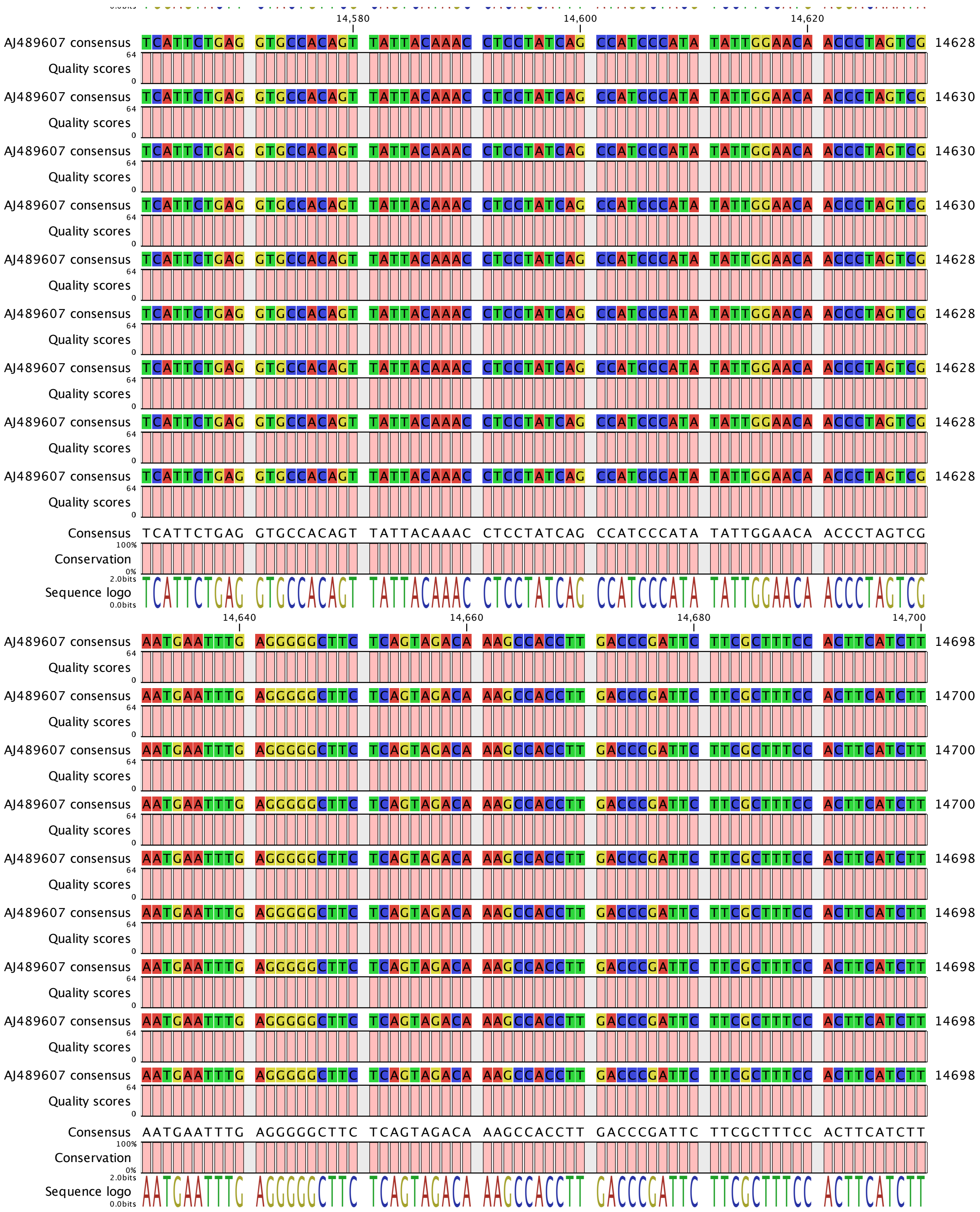

14,580
14,600
14,620
AJ489607 consensus TCATTCTGAG GTGCCACAGT TATTACAAAC CTCCTATCAG CCATCCCATA TATTGGAACA ACCCTAGTCG 14628
Quality scores
AJ489607 consensus TCATTCTGAG GTGCCACAGT TATTACAAAC CTCCTATCAG CCATCCCATA TATTGGAACA ACCCTAGTCG 14630
Quality scores
AJ489607 consensus TCATTCTGAG GTGCCACAGT TATTACAAAC CTCCTATCAG CCATCCCATA TATTGGAACA ACCCTAGTCG 14630
Quality scores
AJ489607 consensus TCATTCTGAG GTGCCACAGT TATTACAAAC CTCCTATCAG CCATCCCATA TATTGGAACA ACCCTAGTCG 14630
Quality scores
AJ489607 consensus TCATTCTGAG GTGCCACAGT TATTACAAAC CTCCTATCAG CCATCCCATA TATTGGAACA ACCCTAGTCG 14628
Quality scores
AJ489607 consensus TCATTCTGAG GTGCCACAGT TATTACAAAC CTCCTATCAG CCATCCCATA TATTGGAACA ACCCTAGTCG 14628
Quality scores
AJ489607 consensus TCATTCTGAG GTGCCACAGT TATTACAAAC CTCCTATCAG CCATCCCATA TATTGGAACA ACCCTAGTCG 14628
Quality scores
AJ489607 consensus TCATTCTGAG GTGCCACAGT TATTACAAAC CTCCTATCAG CCATCCCATA TATTGGAACA ACCCTAGTCG 14628
Quality scores
AJ489607 consensus TCATTCTGAG GTGCCACAGT TATTACAAAC CTCCTATCAG CCATCCCATA TATTGGAACA ACCCTAGTCG 14628
Quality scores
Consensus TCATTCTGAG GTGCCACAGT TATTACAAAC CTCCTATCAG CCATCCCATA TATTGGAACA ACCCTAGTCG
Conservation
Sequence logo
14,640
14,660
14,680
14,700
AJ489607 consensus AATGAATTTG AGGGGGCTTC TCAGTAGACA AAGCCACCTT GACCCGATTC TTCGCTTTCC ACTTCATCTT 14698
Quality scores
AJ489607 consensus AATGAATTTG AGGGGGCTTC TCAGTAGACA AAGCCACCTT GACCCGATTC TTCGCTTTCC ACTTCATCTT 14700
Quality scores
AJ489607 consensus AATGAATTTG AGGGGGCTTC TCAGTAGACA AAGCCACCTT GACCCGATTC TTCGCTTTCC ACTTCATCTT 14700
Quality scores
AJ489607 consensus AATGAATTTG AGGGGGCTTC TCAGTAGACA AAGCCACCTT GACCCGATTC TTCGCTTTCC ACTTCATCTT 14700
Quality scores
AJ489607 consensus AATGAATTTG AGGGGGCTTC TCAGTAGACA AAGCCACCTT GACCCGATTC TTCGCTTTCC ACTTCATCTT 14698
Quality scores
AJ489607 consensus AATGAATTTG AGGGGGCTTC TCAGTAGACA AAGCCACCTT GACCCGATTC TTCGCTTTCC ACTTCATCTT 14698
Quality scores
AJ489607 consensus AATGAATTTG AGGGGGCTTC TCAGTAGACA AAGCCACCTT GACCCGATTC TTCGCTTTCC ACTTCATCTT 14698
Quality scores
AJ489607 consensus AATGAATTTG AGGGGGCTTC TCAGTAGACA AAGCCACCTT GACCCGATTC TTCGCTTTCC ACTTCATCTT 14698
Quality scores
AJ489607 consensus AATGAATTTG AGGGGGCTTC TCAGTAGACA AAGCCACCTT GACCCGATTC TTCGCTTTCC ACTTCATCTT 14698
Quality scores
Consensus AATGAATTTG AGGGGGCTTC TCAGTAGACA AAGCCACCTT GACCCGATTC TTCGCTTTCC ACTTCATCTT
Conservation
Sequence logo

14,720 14,740 14,760

AJ489607 consensus ACCATTTATT ATCGCGGCCC TAGCAATCGT TCACCTCCTC TTCCTCCACG AAACAGGATC AAACAACCCA 14768
64
Quality scores
0
AJ489607 consensus ACCATTTATT ATCGCGGCCC TAGCAATCGT TCACCTCCTC TTCCTCCACG AAACAGGATC AAACAACCCA 14770
64
Quality scores
0
AJ489607 consensus ACCATTTATT ATCGCGGCCC TAGCAATCGT TCACCTCCTC TTCCTCCACG AAACAGGATC AAACAACCCA 14770
64
Quality scores
0
AJ489607 consensus ACCATTTATT ATCGCGGCCC TAGCAATCGT TCACCTCCTC TTCCTCCACG AAACAGGATC AAACAACCCA 14770
64
Quality scores
0
AJ489607 consensus ACCATTTATT ATCGCGGCCC TAGCAATCGT TCACCTCCTC TTCCTCCACG AAACAGGATC AAACAACCCA 14768
64
Quality scores
0
AJ489607 consensus ACCATTTATT ATCGCGGCCC TAGCAATCGT TCACCTCCTC TTCCTCCACG AAACAGGATC AAACAACCCA 14768
64
Quality scores
0
AJ489607 consensus ACCATTTATT ATCGCGGCCC TAGCAATCGT TCACCTCCTC TTCCTCCACG AAACAGGATC AAACAACCCA 14768
64
Quality scores
0
AJ489607 consensus ACCATTTATT ATCGCGGCCC TAGCAATCGT TCACCTCCTC TTCCTCCACG AAACAGGATC AAACAACCCA 14768
64
Quality scores
0
AJ489607 consensus ACCATTTATT ATCGCGGCCC TAGCAATCGT TCACCTCCTC TTCCTCCACG AAACAGGATC AAACAACCCA 14768
64
Quality scores
0
Consensus ACCATTTATT ATCGCGGCCC TAGCAATCGT TCACCTCCTC TTCCTCCACG AAACAGGATC AAACAACCCA
100%
Conservation
0%
2.0bits
Sequence logo ACCATTTATT ATCGCGGCCC TAGCAATCGT TCACCTCCTC TTCCTCCACG AAACAGGATC AAACAACCCA
0.0bits

14,780 14,800 14,820 14,840

AJ489607 consensus ACAGGATTAA ACTCAGATGC AGATAAAATT CCATTTCACC CCTACTATAC AATCAAAGAT ATCCTAGGTA 14838
64
Quality scores
0
AJ489607 consensus ACAGGATTAA ACTCAGATGC AGATAAAATT CCATTTCACC CCTACTATAC AATCAAAGAT ATCCTAGGTA 14840
64
Quality scores
0
AJ489607 consensus ACAGGATTAA ACTCAGATGC AGATAAAATT CCATTTCACC CCTACTATAC AATCAAAGAT ATCCTAGGTA 14840
64
Quality scores
0
AJ489607 consensus ACAGGATTAA ACTCAGATGC AGATAAAATT CCATTTCACC CCTACTATAC AATCAAAGAT ATCCTAGGTA 14840
64
Quality scores
0
AJ489607 consensus ACAGGATTAA ACTCAGATGC AGATAAAATT CCATTTCACC CCTACTATAC AATCAAAGAT ATCCTAGGTA 14838
64
Quality scores
0
AJ489607 consensus ACAGGATTAA ACTCAGATGC AGATAAAATT CCATTTCACC CCTACTATAC AATCAAAGAT ATCCTAGGTA 14838
64
Quality scores
0
AJ489607 consensus ACAGGATTAA ACTCAGATGC AGATAAAATT CCATTTCACC CCTACTATAC AATCAAAGAT ATCCTAGGTA 14838
64
Quality scores
0
AJ489607 consensus ACAGGATTAA ACTCAGATGC AGATAAAATT CCATTTCACC CCTACTATAC AATCAAAGAT ATCCTAGGTA 14838
64
Quality scores
0
AJ489607 consensus ACAGGATTAA ACTCAGATGC AGATAAAATT CCATTTCACC CCTACTATAC AATCAAAGAT ATCCTAGGTA 14838
64
Quality scores
0
Consensus ACAGGATTAA ACTCAGATGC AGATAAAATT CCATTTCACC CCTACTATAC AATCAAAGAT ATCCTAGGTA
100%
Conservation
0%
2.0bits
Sequence logo ACAGGATTAA ACTCAGATGC AGATAAAATT CCATTTCACC CCTACTATAC AATCAAAGAT ATCCTAGGTA
0.0bits

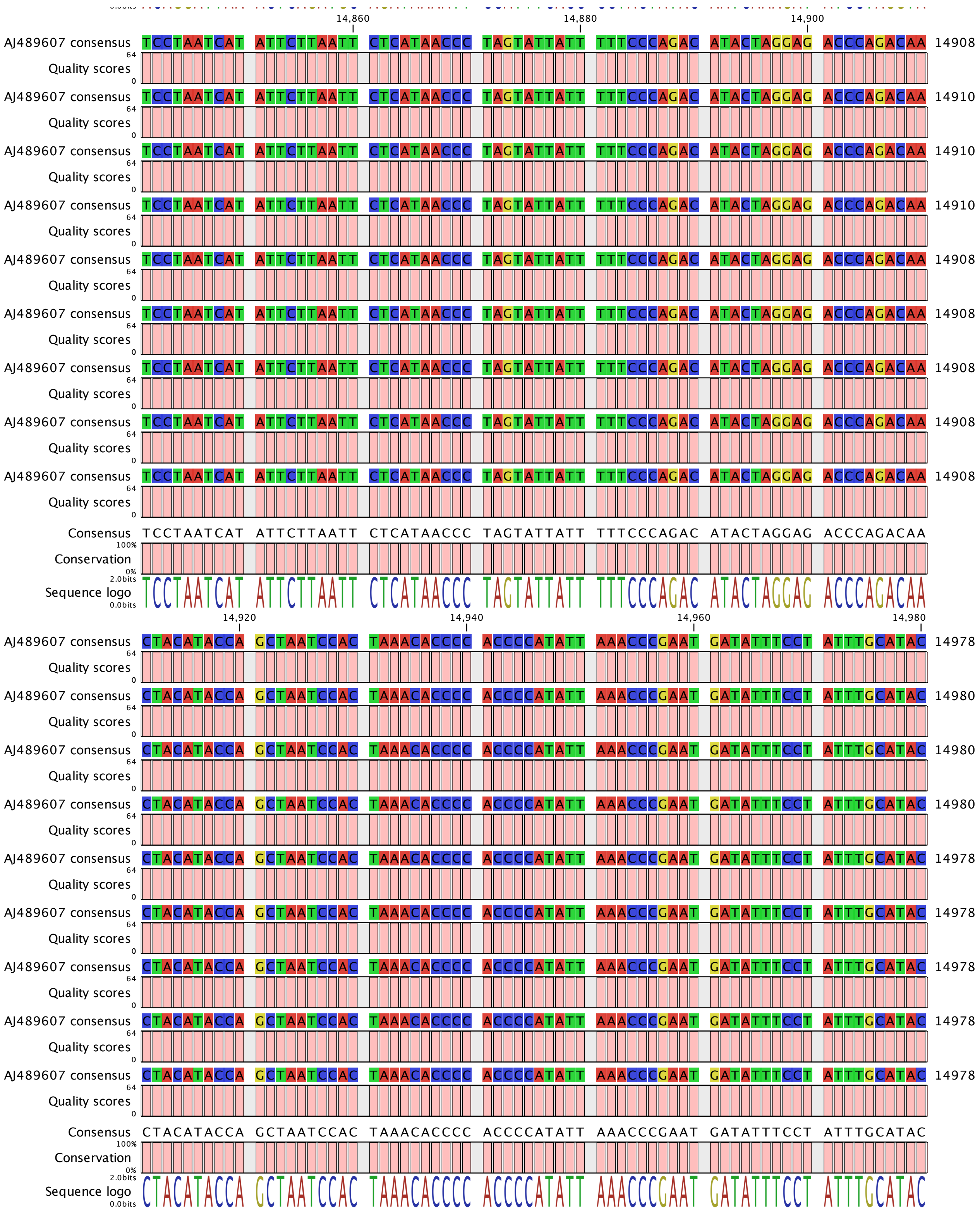

14,860
14,880
14,900
AJ489607 consensus TCCTAATCAT ATTCTTAATT CTCATAACCC TAGTATTATT TTTCCCAGAC ATACTAGGAG ACCCAGACAA 14908
Quality scores
AJ489607 consensus TCCTAATCAT ATTCTTAATT CTCATAACCC TAGTATTATT TTTCCCAGAC ATACTAGGAG ACCCAGACAA 14910
Quality scores
AJ489607 consensus TCCTAATCAT ATTCTTAATT CTCATAACCC TAGTATTATT TTTCCCAGAC ATACTAGGAG ACCCAGACAA 14910
Quality scores
AJ489607 consensus TCCTAATCAT ATTCTTAATT CTCATAACCC TAGTATTATT TTTCCCAGAC ATACTAGGAG ACCCAGACAA 14910
Quality scores
AJ489607 consensus TCCTAATCAT ATTCTTAATT CTCATAACCC TAGTATTATT TTTCCCAGAC ATACTAGGAG ACCCAGACAA 14908
Quality scores
AJ489607 consensus TCCTAATCAT ATTCTTAATT CTCATAACCC TAGTATTATT TTTCCCAGAC ATACTAGGAG ACCCAGACAA 14908
Quality scores
AJ489607 consensus TCCTAATCAT ATTCTTAATT CTCATAACCC TAGTATTATT TTTCCCAGAC ATACTAGGAG ACCCAGACAA 14908
Quality scores
AJ489607 consensus TCCTAATCAT ATTCTTAATT CTCATAACCC TAGTATTATT TTTCCCAGAC ATACTAGGAG ACCCAGACAA 14908
Quality scores
AJ489607 consensus TCCTAATCAT ATTCTTAATT CTCATAACCC TAGTATTATT TTTCCCAGAC ATACTAGGAG ACCCAGACAA 14908
Quality scores
Consensus TCCTAATCAT ATTCTTAATT CTCATAACCC TAGTATTATT TTTCCCAGAC ATACTAGGAG ACCCAGACAA
Conservation
Sequence logo
14,920
14,940
14,960
14,980
AJ489607 consensus CTACATACCA GCTAATCCAC TAAACACCCC ACCCCATATT AAACCCGAAT GATATTTCCT ATTTGCATAC 14978
Quality scores
AJ489607 consensus CTACATACCA GCTAATCCAC TAAACACCCC ACCCCATATT AAACCCGAAT GATATTTCCT ATTTGCATAC 14980
Quality scores
AJ489607 consensus CTACATACCA GCTAATCCAC TAAACACCCC ACCCCATATT AAACCCGAAT GATATTTCCT ATTTGCATAC 14980
Quality scores
AJ489607 consensus CTACATACCA GCTAATCCAC TAAACACCCC ACCCCATATT AAACCCGAAT GATATTTCCT ATTTGCATAC 14980
Quality scores
AJ489607 consensus CTACATACCA GCTAATCCAC TAAACACCCC ACCCCATATT AAACCCGAAT GATATTTCCT ATTTGCATAC 14978
Quality scores
AJ489607 consensus CTACATACCA GCTAATCCAC TAAACACCCC ACCCCATATT AAACCCGAAT GATATTTCCT ATTTGCATAC 14978
Quality scores
AJ489607 consensus CTACATACCA GCTAATCCAC TAAACACCCC ACCCCATATT AAACCCGAAT GATATTTCCT ATTTGCATAC 14978
Quality scores
AJ489607 consensus CTACATACCA GCTAATCCAC TAAACACCCC ACCCCATATT AAACCCGAAT GATATTTCCT ATTTGCATAC 14978
Quality scores
AJ489607 consensus CTACATACCA GCTAATCCAC TAAACACCCC ACCCCATATT AAACCCGAAT GATATTTCCT ATTTGCATAC 14978
Quality scores
Consensus CTACATACCA GCTAATCCAC TAAACACCCC ACCCCATATT AAACCCGAAT GATATTTCCT ATTTGCATAC
Conservation
Sequence logo

0.0bits

15,000 15,020 15,040

AJ489607 consensus GCCATTCTAC GCTCAATCCC CAATAAACTA GGAGGTGTCC TAGCCTTAAT CTTATCTATC CTAATTTTAG 15048
Quality scores 64 0
AJ489607 consensus GCCATTCTAC GCTCAATCCC CAATAAACTA GGAGGTGTCC TAGCCTTAAT CTTATCTATC CTAATTTTAG 15050
Quality scores 64 0
AJ489607 consensus GCCATTCTAC GCTCAATCCC CAATAAACTA GGAGGTGTCC TAGCCTTAAT CTTATCTATC CTAATTTTAG 15050
Quality scores 64 0
AJ489607 consensus GCCATTCTAC GCTCAATCCC CAATAAACTA GGAGGTGTCC TAGCCTTAAT CTTATCTATC CTAATTTTAG 15050
Quality scores 64 0
AJ489607 consensus GCCATTCTAC GCTCAATCCC CAATAAACTA GGAGGTGTCC TAGCCTTAAT CTTATCTATC CTAATTTTAG 15048
Quality scores 64 0
AJ489607 consensus GCCATTCTAC GCTCAATCCC CAATAAACTA GGAGGTGTCC TAGCCTTAAT CTTATCTATC CTAATTTTAG 15048
Quality scores 64 0
AJ489607 consensus GCCATTCTAC GCTCAATCCC CAATAAACTA GGAGGTGTCC TAGCCTTAAT CTTATCTATC CTAATTTTAG 15048
Quality scores 64 0
AJ489607 consensus GCCATTCTAC GCTCAATCCC CAATAAACTA GGAGGTGTCC TAGCCTTAAT CTTATCTATC CTAATTTTAG 15048
Quality scores 64 0
AJ489607 consensus GCCATTCTAC GCTCAATCCC CAATAAACTA GGAGGTGTCC TAGCCTTAAT CTTATCTATC CTAATTTTAG 15048
Quality scores 64 0
Consensus GCCATTCTAC GCTCAATCCC CAATAAACTA GGAGGTGTCC TAGCCTTAAT CTTATCTATC CTAATTTTAG
Conservation 100% 0%
Sequence logo 2.0bits 0.0bits GCCATTCTAC GCTCAATCCC CAATAAACTA GGAGGTGTCC TAGCCTTAAT CTTATCTATC CTAATTTTAG

15,060 15,080 15,100 15,120

AJ489607 consensus CCCTAATACC TTTCCTTCAT ACCTCAAAGC AACGAAGCCT AATATTCCGC CCAATCACAC AAATTTTGTA 15118
Quality scores 64 0
AJ489607 consensus CCCTAATACC TTTCCTTCAT ACCTCAAAGC AACGAAGCCT AATATTCCGC CCAATCACAC AAATTTTGTA 15120
Quality scores 64 0
AJ489607 consensus CCCTAATACC TTTCCTTCAT ACCTCAAAGC AACGAAGCCT AATATTCCGC CCAATCACAC AAATTTTGTA 15120
Quality scores 64 0
AJ489607 consensus CCCTAATACC TTTCCTTCAT ACCTCAAAGC AACGAAGCCT AATATTCCGC CCAATCACAC AAATTTTGTA 15120
Quality scores 64 0
AJ489607 consensus CCCTAATACC TTTCCTTCAT ACCTCAAAGC AACGAAGCCT AATATTCCGC CCAATCACAC AAATTTTGTA 15118
Quality scores 64 0
AJ489607 consensus CCCTAATACC TTTCCTTCAT ACCTCAAAGC AACGAAGCCT AATATTCCGC CCAATCACAC AAATTTTGTA 15118
Quality scores 64 0
AJ489607 consensus CCCTAATACC TTTCCTTCAT ACCTCAAAGC AACGAAGCCT AATATTCCGC CCAATCACAC AAATTTTGTA 15118
Quality scores 64 0
AJ489607 consensus CCCTAATACC TTTCCTTCAT ACCTCAAAGC AACGAAGCCT AATATTCCGC CCAATCACAC AAATTTTGTA 15118
Quality scores 64 0
AJ489607 consensus CCCTAATACC TTTCCTTCAT ACCTCAAAGC AACGAAGCCT AATATTCCGC CCAATCACAC AAATTTTGTA 15118
Quality scores 64 0
Consensus CCCTAATACC TTTCCTTCAT ACCTCAAAGC AACGAAGCCT AATATTCCGC CCAATCACAC AAATTTTGTA
Conservation 100% 0%
Sequence logo 2.0bits 0.0bits CCCTAATACC TTTCCTTCAT ACCTCAAAGC AACGAAGCCT AATATTCCGC CCAATCACAC AAATTTTGTA

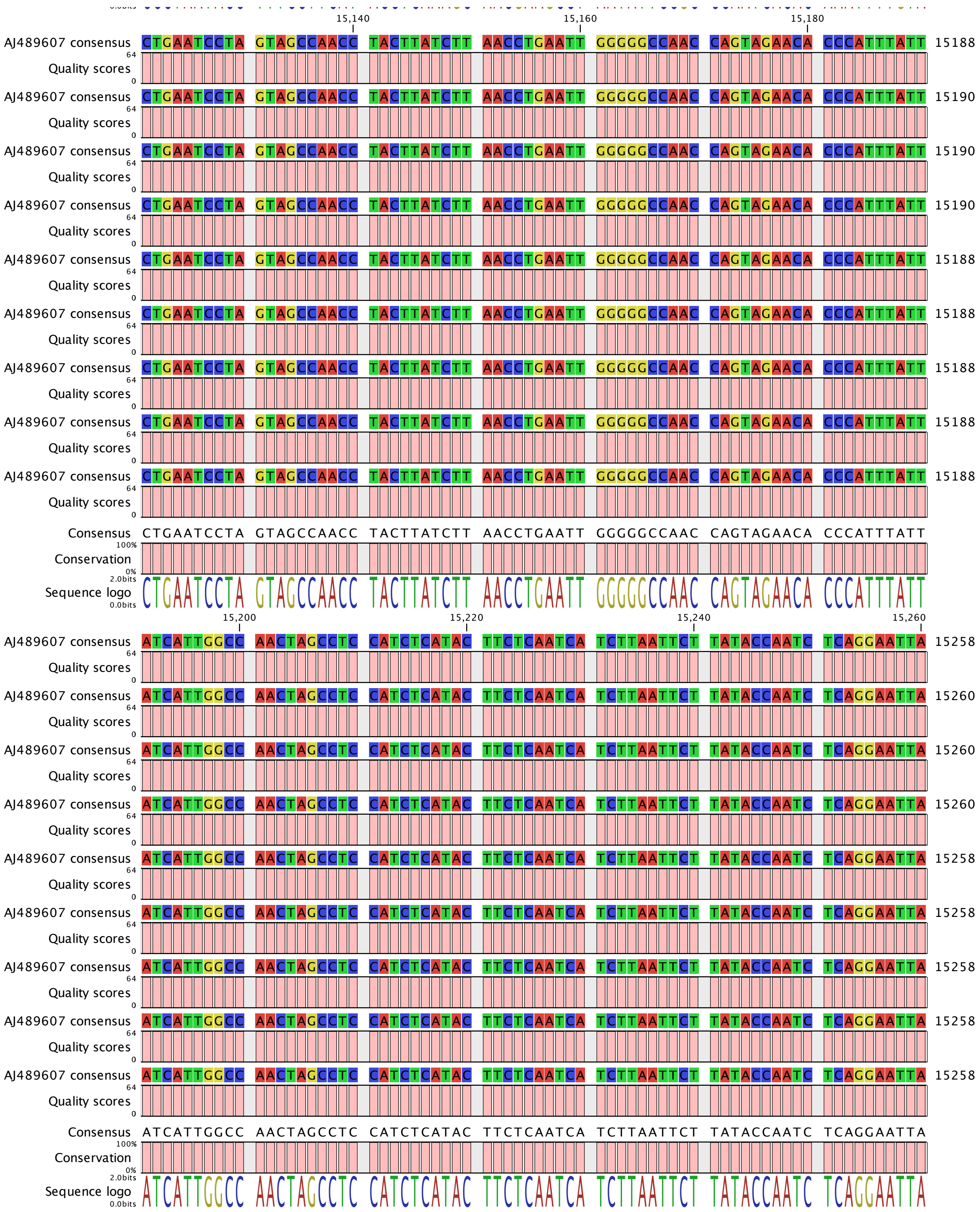

15,140
15,160
15,180
AJ489607 consensus CTGAATCCTA GTAGCCAACC TACTTATCTT AACCTGAATT GGGGGCCAAC CAGTAGAACA CCCATTTATT 15188
Quality scores
AJ489607 consensus CTGAATCCTA GTAGCCAACC TACTTATCTT AACCTGAATT GGGGGCCAAC CAGTAGAACA CCCATTTATT 15190
Quality scores
AJ489607 consensus CTGAATCCTA GTAGCCAACC TACTTATCTT AACCTGAATT GGGGGCCAAC CAGTAGAACA CCCATTTATT 15190
Quality scores
AJ489607 consensus CTGAATCCTA GTAGCCAACC TACTTATCTT AACCTGAATT GGGGGCCAAC CAGTAGAACA CCCATTTATT 15190
Quality scores
AJ489607 consensus CTGAATCCTA GTAGCCAACC TACTTATCTT AACCTGAATT GGGGGCCAAC CAGTAGAACA CCCATTTATT 15188
Quality scores
AJ489607 consensus CTGAATCCTA GTAGCCAACC TACTTATCTT AACCTGAATT GGGGGCCAAC CAGTAGAACA CCCATTTATT 15188
Quality scores
AJ489607 consensus CTGAATCCTA GTAGCCAACC TACTTATCTT AACCTGAATT GGGGGCCAAC CAGTAGAACA CCCATTTATT 15188
Quality scores
AJ489607 consensus CTGAATCCTA GTAGCCAACC TACTTATCTT AACCTGAATT GGGGGCCAAC CAGTAGAACA CCCATTTATT 15188
Quality scores
AJ489607 consensus CTGAATCCTA GTAGCCAACC TACTTATCTT AACCTGAATT GGGGGCCAAC CAGTAGAACA CCCATTTATT 15188
Quality scores
Consensus CTGAATCCTA GTAGCCAACC TACTTATCTT AACCTGAATT GGGGGCCAAC CAGTAGAACA CCCATTTATT
Conservation
Sequence logo CTGAATCCTA GTAGCCAACC TACTTATCTT AACCTGAATT GGGGGCCAAC CAGTAGAACA CCCATTTATT
15,200
15,220
15,240
15,260
AJ489607 consensus ATCATTGGCC AACTAGCCTC CATCTCATAC TTCTCAATCA TCTTAATTCT TATACCAATC TCAGGAATTA 15258
Quality scores
AJ489607 consensus ATCATTGGCC AACTAGCCTC CATCTCATAC TTCTCAATCA TCTTAATTCT TATACCAATC TCAGGAATTA 15260
Quality scores
AJ489607 consensus ATCATTGGCC AACTAGCCTC CATCTCATAC TTCTCAATCA TCTTAATTCT TATACCAATC TCAGGAATTA 15260
Quality scores
AJ489607 consensus ATCATTGGCC AACTAGCCTC CATCTCATAC TTCTCAATCA TCTTAATTCT TATACCAATC TCAGGAATTA 15260
Quality scores
AJ489607 consensus ATCATTGGCC AACTAGCCTC CATCTCATAC TTCTCAATCA TCTTAATTCT TATACCAATC TCAGGAATTA 15258
Quality scores
AJ489607 consensus ATCATTGGCC AACTAGCCTC CATCTCATAC TTCTCAATCA TCTTAATTCT TATACCAATC TCAGGAATTA 15258
Quality scores
AJ489607 consensus ATCATTGGCC AACTAGCCTC CATCTCATAC TTCTCAATCA TCTTAATTCT TATACCAATC TCAGGAATTA 15258
Quality scores
AJ489607 consensus ATCATTGGCC AACTAGCCTC CATCTCATAC TTCTCAATCA TCTTAATTCT TATACCAATC TCAGGAATTA 15258
Quality scores
AJ489607 consensus ATCATTGGCC AACTAGCCTC CATCTCATAC TTCTCAATCA TCTTAATTCT TATACCAATC TCAGGAATTA 15258
Quality scores
Consensus ATCATTGGCC AACTAGCCTC CATCTCATAC TTCTCAATCA TCTTAATTCT TATACCAATC TCAGGAATTA
Conservation
Sequence logo ATCATTGGCC AACTAGCCTC CATCTCATAC TTCTCAATCA TCTTAATTCT TATACCAATC TCAGGAATTA

15,280 15,300 15,320

AJ489607 consensus TCGAAGACAA AATACTAAAA TTATATCCAT GTCTTGATAG TATAAACATT ACTCTGGTCT TGTAAACCTG 15328
64
Quality scores
0
AJ489607 consensus TCGAAGACAA AATACTAAAA TTATATCCAT GTCTTGATAG TATAAACATT ACTCTGGTCT TGTAAACCTG 15330
64
Quality scores
0
AJ489607 consensus TCGAAGACAA AATACTAAAA TTATATCCAT GTCTTGATAG TATAAACATT ACTCTGGTCT TGTAAACCTG 15330
64
Quality scores
0
AJ489607 consensus TCGAAGACAA AATACTAAAA TTATATCCAT GTCTTGATAG TATAAACATT ACTCTGGTCT TGTAAACCTG 15330
64
Quality scores
0
AJ489607 consensus TCGAAGACAA AATACTAAAA TTATATCCAT GTCTTGATAG TATAAACATT ACTCTGGTCT TGTAAACCTG 15328
64
Quality scores
0
AJ489607 consensus TCGAAGACAA AATACTAAAA TTATATCCAT GTCTTGATAG TATAAACATT ACTCTGGTCT TGTAAACCTG 15328
64
Quality scores
0
AJ489607 consensus TCGAAGACAA AATACTAAAA TTATATCCAT GTCTTGATAG TATAAACATT ACTCTGGTCT TGTAAACCTG 15328
64
Quality scores
0
AJ489607 consensus TCGAAGACAA AATACTAAAA TTATATCCAT GTCTTGATAG TATAAACATT ACTCTGGTCT TGTAAACCTG 15328
64
Quality scores
0
AJ489607 consensus TCGAAGACAA AATACTAAAA TTATATCCAT GTCTTGATAG TATAAACATT ACTCTGGTCT TGTAAACCTG 15328
64
Quality scores
0
Consensus TCGAAGACAA AATACTAAAA TTATATCCAT GTCTTGATAG TATAAACATT ACTCTGGTCT TGTAAACCTG
100%
Conservation
0%
2.0bits
Sequence logo TCGAAGACAA AATACTAAAA TTATATCCAT GTCTTGATAG TATAAACATT ACTCTGGTCT TGTAAACCTG
0.0bits

15,340 15,360 15,380 15,400

AJ489607 consensus AAATGAAGAT CTTCTCTTCT CAAGACATCA AGAAGAAGGA GCTACTCCCC ACCACCAGCA CCCAAAGCTG 15398
64
Quality scores
0
AJ489607 consensus AAATGAAGAT CTTCTCTTCT CAAGACATCA AGAAGAAGGA GCTACTCCCC ACCACCAGCA CCCAAAGCTG 15400
64
Quality scores
0
AJ489607 consensus AAATGAAGAT CTTCTCTTCT CAAGACATCA AGAAGAAGGA GCTACTCCCC ACCACCAGCA CCCAAAGCTG 15400
64
Quality scores
0
AJ489607 consensus AAATGAAGAT CTTCTCTTCT CAAGACATCA AGAAGAAGGA GCTACTCCCC ACCACCAGCA CCCAAAGCTG 15400
64
Quality scores
0
AJ489607 consensus AAATGAAGAT CTTCTCTTCT CAAGACATCA AGAAGAAGGA GCTACTCCCC ACCACCAGCA CCCAAAGCTG 15398
64
Quality scores
0
AJ489607 consensus AAATGAAGAT CTTCTCTTCT CAAGACATCA AGAAGAAGGA GCTACTCCCC ACCACCAGCA CCCAAAGCTG 15398
64
Quality scores
0
AJ489607 consensus AAATGAAGAT CTTCTCTTCT CAAGACATCA AGAAGAAGGA GCTACTCCCC ACCACCAGCA CCCAAAGCTG 15398
64
Quality scores
0
AJ489607 consensus AAATGAAGAT CTTCTCTTCT CAAGACATCA AGAAGAAGGA GCTACTCCCC ACCACCAGCA CCCAAAGCTG 15398
64
Quality scores
0
AJ489607 consensus AAATGAAGAT CTTCTCTTCT CAAGACATCA AGAAGAAGGA GCTACTCCCC ACCACCAGCA CCCAAAGCTG 15398
64
Quality scores
0
Consensus AAATGAAGAT CTTCTCTTCT CAAGACATCA AGAAGAAGGA GCTACTCCCC ACCACCAGCA CCCAAAGCTG
100%
Conservation
0%
2.0bits
Sequence logo AAATGAAGAT CTTCTCTTCT CAAGACATCA AGAAGAAGGA GCTACTCCCC ACCACCAGCA CCCAAAGCTG
0.0bits

15,420 15,440 15,460

AJ489607 consensus GTATTCTAAT TAAACTACTT CTTGAGTACA TAAATTTACA TAGTACAACA GTACATTTAT GTATATCGTA 15468
64 Quality scores 0
AJ489607 consensus GTATTCTAAT TAAACTACTT CTTGAGTACA TAAATTTACA TAGTACAACA GTACATTTAT GTATATCGTA 15470
64 Quality scores 0
AJ489607 consensus GTATTCTAAT TAAACTACTT CTTGAGTACA TAAATTTACA TAGTACAACA GTACATTTAT GTATATCGTA 15470
64 Quality scores 0
AJ489607 consensus GTATTCTAAT TAAACTACTT CTTGAGTACA TAAATTTACA TAGTACAACA GTACATTTAT GTATATCGTA 15470
64 Quality scores 0
AJ489607 consensus GTATTCTAAT TAAACTACTT CTTGAGTACA TAAATTTACA TAGTACAACA GTACATTTAT GTATATCGTA 15468
64 Quality scores 0
AJ489607 consensus GTATTCTAAT TAAACTACTT CTTGAGTACA TAAATTTACA TAGTACAACA GTACATTTAT GTATATCGTA 15468
64 Quality scores 0
AJ489607 consensus GTATTCTAAT TAAACTACTT CTTGAGTACA TAAATTTACA TAGTACAACA GTACATTTAT GTATATCGTA 15468
64 Quality scores 0
AJ489607 consensus GTATTCTAAT TAAACTACTT CTTGAGTACA TAAATTTACA TAGTACAACA GTACATTTAT GTATATCGTA 15468
64 Quality scores 0
AJ489607 consensus GTATTCTAAT TAAACTACTT CTTGAGTACA TAAATTTACA TAGTACAACA GTACATTTAT GTATATCGTA 15468
64 Quality scores 0
Consensus GTATTCTAAT TAAACTACTT CTTGAGTACA TAAATTTACA TAGTACAACA GTACATTTAT GTATATCGTA
100% Conservation 0%
2.0bits Sequence logo 0.0bits GTATTCTAAT TAAACTACTT CTTGAGTACA TAAATTTACA TAGTACAACA GTACATTTAT GTATATCGTA

15,480 15,500 15,520 15,540

AJ489607 consensus CATTAAACTA TTTTCCCCAA GCATATAAGC TAGTACATTA AATCAATGGT TCAGGTCATA AAATAATCAT 15538
64 Quality scores 0
AJ489607 consensus CATTAAACTA TTTTCCCCAA GCATATAAGC TAGTACATTA AATCAATGGT TCAGGTCATA AAATAATCAT 15540
64 Quality scores 0
AJ489607 consensus CATTAAACTA TTTTCCCCAA GCATATAAGC TAGTACATTA AATCAATGGT TCAGGTCATA AAATAATCAT 15540
64 Quality scores 0
AJ489607 consensus CATTAAACTA TTTTCCCCAA GCATATAAGC TAGTACATTA AATCAATGGT TCAGGTCATA AAATAATCAT 15540
64 Quality scores 0
AJ489607 consensus CATTAAACTA TTTTCCCCAA GCATATAAGC TAGTACATTA AATCAATGGT TCAGGTCATA AAATAATCAT 15538
64 Quality scores 0
AJ489607 consensus CATTAAACTA TTTTCCCCAA GCATATAAGC TAGTACATTA AATCAATGGT TCAGGTCATA AAATAATCAT 15538
64 Quality scores 0
AJ489607 consensus CATTAAACTA TTTTCCCCAA GCATATAAGC TAGTACATTA AATCAATGGT TCAGGTCATA AAATAATCAT 15538
64 Quality scores 0
AJ489607 consensus CATTAAACTA TTTTCCCCAA GCATATAAGC TAGTACATTA AATCAATGGT TCAGGTCATA AAATAATCAT 15538
64 Quality scores 0
AJ489607 consensus CATTAAACTA TTTTCCCCAA GCATATAAGC TAGTACATTA AATCAATGGT TCAGGTCATA AAATAATCAT 15538
64 Quality scores 0
Consensus CATTAAACTA TTTTCCCCAA GCATATAAGC TAGTACATTA AATCAATGGT TCAGGTCATA AAATAATCAT
100% Conservation 0%
2.0bits Sequence logo 0.0bits CATTAAACTA TTTTCCCCAA GCATATAAGC TAGTACATTA AATCAATGGT TCAGGTCATA AAATAATCAT

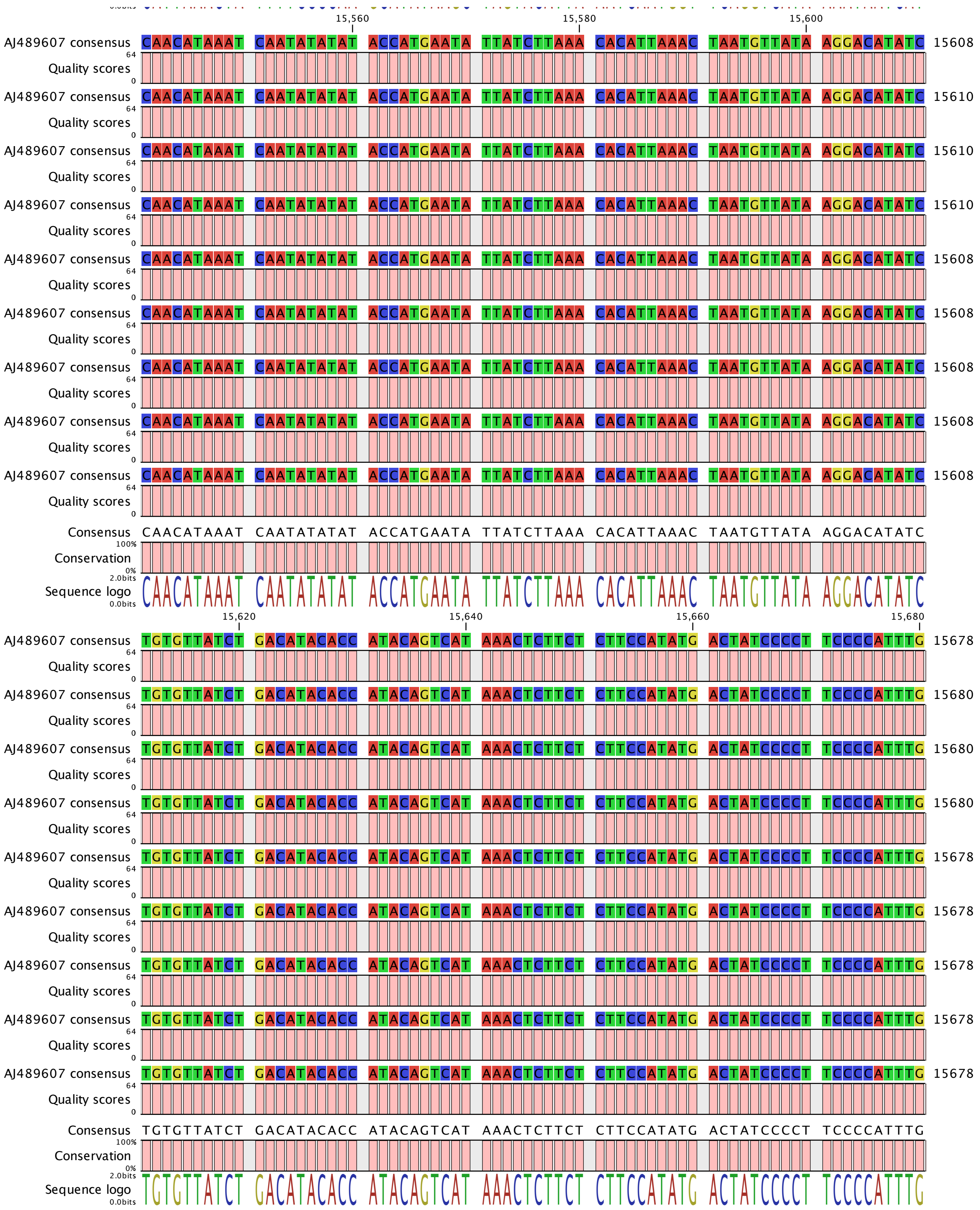
15,560
15,580
15,600
AJ489607 consensus CAACATAAAT CAATATATAT ACCATGAATA TTATCTTAAA CACATTAAAC TAATGTTATA AGGACATATC 15608
Quality scores
AJ489607 consensus CAACATAAAT CAATATATAT ACCATGAATA TTATCTTAAA CACATTAAAC TAATGTTATA AGGACATATC 15610
Quality scores
AJ489607 consensus CAACATAAAT CAATATATAT ACCATGAATA TTATCTTAAA CACATTAAAC TAATGTTATA AGGACATATC 15610
Quality scores
AJ489607 consensus CAACATAAAT CAATATATAT ACCATGAATA TTATCTTAAA CACATTAAAC TAATGTTATA AGGACATATC 15610
Quality scores
AJ489607 consensus CAACATAAAT CAATATATAT ACCATGAATA TTATCTTAAA CACATTAAAC TAATGTTATA AGGACATATC 15608
Quality scores
AJ489607 consensus CAACATAAAT CAATATATAT ACCATGAATA TTATCTTAAA CACATTAAAC TAATGTTATA AGGACATATC 15608
Quality scores
AJ489607 consensus CAACATAAAT CAATATATAT ACCATGAATA TTATCTTAAA CACATTAAAC TAATGTTATA AGGACATATC 15608
Quality scores
AJ489607 consensus CAACATAAAT CAATATATAT ACCATGAATA TTATCTTAAA CACATTAAAC TAATGTTATA AGGACATATC 15608
Quality scores
AJ489607 consensus CAACATAAAT CAATATATAT ACCATGAATA TTATCTTAAA CACATTAAAC TAATGTTATA AGGACATATC 15608
Quality scores
Consensus CAACATAAAT CAATATATAT ACCATGAATA TTATCTTAAA CACATTAAAC TAATGTTATA AGGACATATC
Conservation
Sequence logo
15,620
15,640
15,660
15,680
AJ489607 consensus TGTGTTATCT GACATACACC ATACAGTCAT AAACTCTTCT CTTCCATATG ACTATCCCCT TCCCCATTTG 15678
Quality scores
AJ489607 consensus TGTGTTATCT GACATACACC ATACAGTCAT AAACTCTTCT CTTCCATATG ACTATCCCCT TCCCCATTTG 15680
Quality scores
AJ489607 consensus TGTGTTATCT GACATACACC ATACAGTCAT AAACTCTTCT CTTCCATATG ACTATCCCCT TCCCCATTTG 15680
Quality scores
AJ489607 consensus TGTGTTATCT GACATACACC ATACAGTCAT AAACTCTTCT CTTCCATATG ACTATCCCCT TCCCCATTTG 15680
Quality scores
AJ489607 consensus TGTGTTATCT GACATACACC ATACAGTCAT AAACTCTTCT CTTCCATATG ACTATCCCCT TCCCCATTTG 15678
Quality scores
AJ489607 consensus TGTGTTATCT GACATACACC ATACAGTCAT AAACTCTTCT CTTCCATATG ACTATCCCCT TCCCCATTTG 15678
Quality scores
AJ489607 consensus TGTGTTATCT GACATACACC ATACAGTCAT AAACTCTTCT CTTCCATATG ACTATCCCCT TCCCCATTTG 15678
Quality scores
AJ489607 consensus TGTGTTATCT GACATACACC ATACAGTCAT AAACTCTTCT CTTCCATATG ACTATCCCCT TCCCCATTTG 15678
Quality scores
AJ489607 consensus TGTGTTATCT GACATACACC ATACAGTCAT AAACTCTTCT CTTCCATATG ACTATCCCCT TCCCCATTTG 15678
Quality scores
Consensus TGTGTTATCT GACATACACC ATACAGTCAT AAACTCTTCT CTTCCATATG ACTATCCCCT TCCCCATTTG
Conservation
Sequence logo

0.0bits

15,700 15,720 15,740

AJ489607 consensus GTCTATTAAT CTACCATCCT CCGTGAAACC AACAACCCGC CCACCAATGC CCCTCTTCTC GCTCCGGGCC 15748
64
Quality scores
0
AJ489607 consensus GTCTATTAAT CTACCATCCT CCGTGAAACC AACAACCCGC CCACCAATGC CCCTCTTCTC GCTCCGGGCC 15750
64
Quality scores
0
AJ489607 consensus GTCTATTAAT CTACCATCCT CCGTGAAACC AACAACCCGC CCACCAATGC CCCTCTTCTC GCTCCGGGCC 15750
64
Quality scores
0
AJ489607 consensus GTCTATTAAT CTACCATCCT CCGTGAAACC AACAACCCGC CCACCAATGC CCCTCTTCTC GCTCCGGGCC 15750
64
Quality scores
0
AJ489607 consensus GTCTATTAAT CTACCATCCT CCGTGAAACC AACAACCCGC CCACCAATGC CCCTCTTCTC GCTCCGGGCC 15748
64
Quality scores
0
AJ489607 consensus GTCTATTAAT CTACCATCCT CCGTGAAACC AACAACCCGC CCACCAATGC CCCTCTTCTC GCTCCGGGCC 15748
64
Quality scores
0
AJ489607 consensus GTCTATTAAT CTACCATCCT CCGTGAAACC AACAACCCGC CCACCAATGC CCCTCTTCTC GCTCCGGGCC 15748
64
Quality scores
0
AJ489607 consensus GTCTATTAAT CTACCATCCT CCGTGAAACC AACAACCCGC CCACCAATGC CCCTCTTCTC GCTCCGGGCC 15748
64
Quality scores
0
AJ489607 consensus GTCTATTAAT CTACCATCCT CCGTGAAACC AACAACCCGC CCACCAATGC CCCTCTTCTC GCTCCGGGCC 15748
64
Quality scores
0
Consensus GTCTATTAAT CTACCATCCT CCGTGAAACC AACAACCCGC CCACCAATGC CCCTCTTCTC GCTCCGGGCC
100%
Conservation
0%
2.0bits
Sequence logo GTCTATTAAT CTACCATCCT CCGTGAAACC AACAACCCGC CCACCAATGC CCCTCTTCTC GCTCCGGGCC
0.0bits

15,760 15,780 15,800 15,820

AJ489607 consensus CATTAAACTT GGGGGTAGCT AAACTGAAAC TTTATCAGAC ATCTGGTTCT TACTTCAGGG CCATCAAATG 15818
64
Quality scores
0
AJ489607 consensus CATTAAACTT GGGGGTAGCT AAACTGAAAC TTTATCAGAC ATCTGGTTCT TACTTCAGGG CCATCAAATG 15820
64
Quality scores
0
AJ489607 consensus CATTAAACTT GGGGGTAGCT AAACTGAAAC TTTATCAGAC ATCTGGTTCT TACTTCAGGG CCATCAAATG 15820
64
Quality scores
0
AJ489607 consensus CATTAAACTT GGGGGTAGCT AAACTGAAAC TTTATCAGAC ATCTGGTTCT TACTTCAGGG CCATCAAATG 15820
64
Quality scores
0
AJ489607 consensus CATTAAACTT GGGGGTAGCT AAACTGAAAC TTTATCAGAC ATCTGGTTCT TACTTCAGGG CCATCAAATG 15818
64
Quality scores
0
AJ489607 consensus CATTAAACTT GGGGGTAGCT AAACTGAAAC TTTATCAGAC ATCTGGTTCT TACTTCAGGG CCATCAAATG 15818
64
Quality scores
0
AJ489607 consensus CATTAAACTT GGGGGTAGCT AAACTGAAAC TTTATCAGAC ATCTGGTTCT TACTTCAGGG CCATCAAATG 15818
64
Quality scores
0
AJ489607 consensus CATTAAACTT GGGGGTAGCT AAACTGAAAC TTTATCAGAC ATCTGGTTCT TACTTCAGGG CCATCAAATG 15818
64
Quality scores
0
AJ489607 consensus CATTAAACTT GGGGGTAGCT AAACTGAAAC TTTATCAGAC ATCTGGTTCT TACTTCAGGG CCATCAAATG 15818
64
Quality scores
0
Consensus CATTAAACTT GGGGGTAGCT AAACTGAAAC TTTATCAGAC ATCTGGTTCT TACTTCAGGG CCATCAAATG
100%
Conservation
0%
2.0bits
Sequence logo CATTAAACTT GGGGGTAGCT AAACTGAAAC TTTATCAGAC ATCTGGTTCT TACTTCAGGG CCATCAAATG
0.0bits

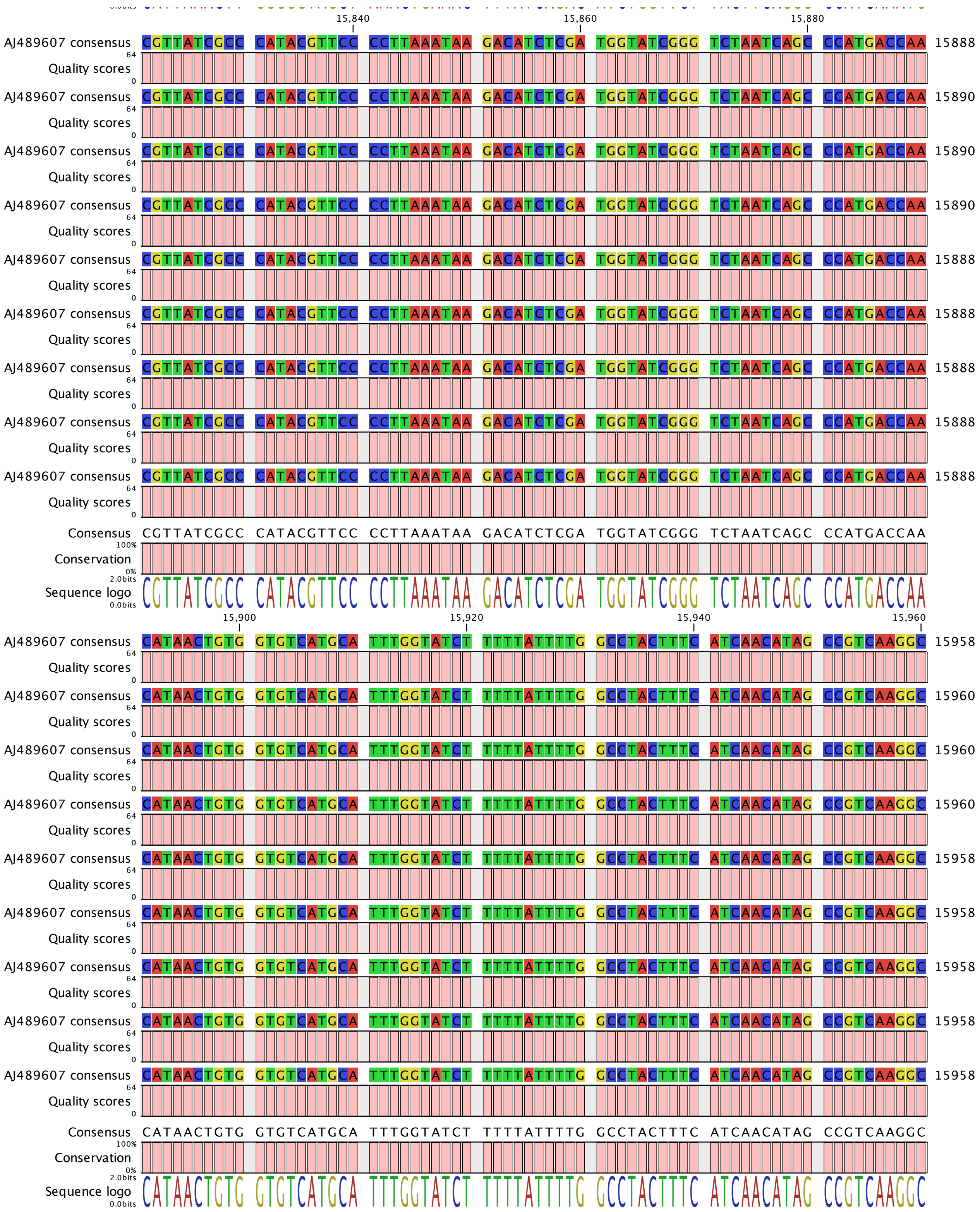
15,840
15,860
15,880
AJ489607 consensus CGTTATCGCC CATACGTTCC CCTTAAATAA GACATCTCGA TGGTATCGGG TCTAATCAGC CCATGACCAA 15888
Quality scores
AJ489607 consensus CGTTATCGCC CATACGTTCC CCTTAAATAA GACATCTCGA TGGTATCGGG TCTAATCAGC CCATGACCAA 15890
Quality scores
AJ489607 consensus CGTTATCGCC CATACGTTCC CCTTAAATAA GACATCTCGA TGGTATCGGG TCTAATCAGC CCATGACCAA 15890
Quality scores
AJ489607 consensus CGTTATCGCC CATACGTTCC CCTTAAATAA GACATCTCGA TGGTATCGGG TCTAATCAGC CCATGACCAA 15890
Quality scores
AJ489607 consensus CGTTATCGCC CATACGTTCC CCTTAAATAA GACATCTCGA TGGTATCGGG TCTAATCAGC CCATGACCAA 15888
Quality scores
AJ489607 consensus CGTTATCGCC CATACGTTCC CCTTAAATAA GACATCTCGA TGGTATCGGG TCTAATCAGC CCATGACCAA 15888
Quality scores
AJ489607 consensus CGTTATCGCC CATACGTTCC CCTTAAATAA GACATCTCGA TGGTATCGGG TCTAATCAGC CCATGACCAA 15888
Quality scores
AJ489607 consensus CGTTATCGCC CATACGTTCC CCTTAAATAA GACATCTCGA TGGTATCGGG TCTAATCAGC CCATGACCAA 15888
Quality scores
AJ489607 consensus CGTTATCGCC CATACGTTCC CCTTAAATAA GACATCTCGA TGGTATCGGG TCTAATCAGC CCATGACCAA 15888
Quality scores
Consensus CGTTATCGCC CATACGTTCC CCTTAAATAA GACATCTCGA TGGTATCGGG TCTAATCAGC CCATGACCAA
Conservation
Sequence logo
15,900
15,920
15,940
15,960
AJ489607 consensus CATAACTGTG GTGTCATGCA TTTGGTATCT TTTTATTTTG GCCTACTTTC ATCAACATAG CCGTCAAGGC 15958
Quality scores
AJ489607 consensus CATAACTGTG GTGTCATGCA TTTGGTATCT TTTTATTTTG GCCTACTTTC ATCAACATAG CCGTCAAGGC 15960
Quality scores
AJ489607 consensus CATAACTGTG GTGTCATGCA TTTGGTATCT TTTTATTTTG GCCTACTTTC ATCAACATAG CCGTCAAGGC 15960
Quality scores
AJ489607 consensus CATAACTGTG GTGTCATGCA TTTGGTATCT TTTTATTTTG GCCTACTTTC ATCAACATAG CCGTCAAGGC 15960
Quality scores
AJ489607 consensus CATAACTGTG GTGTCATGCA TTTGGTATCT TTTTATTTTG GCCTACTTTC ATCAACATAG CCGTCAAGGC 15958
Quality scores
AJ489607 consensus CATAACTGTG GTGTCATGCA TTTGGTATCT TTTTATTTTG GCCTACTTTC ATCAACATAG CCGTCAAGGC 15958
Quality scores
AJ489607 consensus CATAACTGTG GTGTCATGCA TTTGGTATCT TTTTATTTTG GCCTACTTTC ATCAACATAG CCGTCAAGGC 15958
Quality scores
AJ489607 consensus CATAACTGTG GTGTCATGCA TTTGGTATCT TTTTATTTTG GCCTACTTTC ATCAACATAG CCGTCAAGGC 15958
Quality scores
AJ489607 consensus CATAACTGTG GTGTCATGCA TTTGGTATCT TTTTATTTTG GCCTACTTTC ATCAACATAG CCGTCAAGGC 15958
Quality scores
Consensus CATAACTGTG GTGTCATGCA TTTGGTATCT TTTTATTTTG GCCTACTTTC ATCAACATAG CCGTCAAGGC
Conservation
Sequence logo

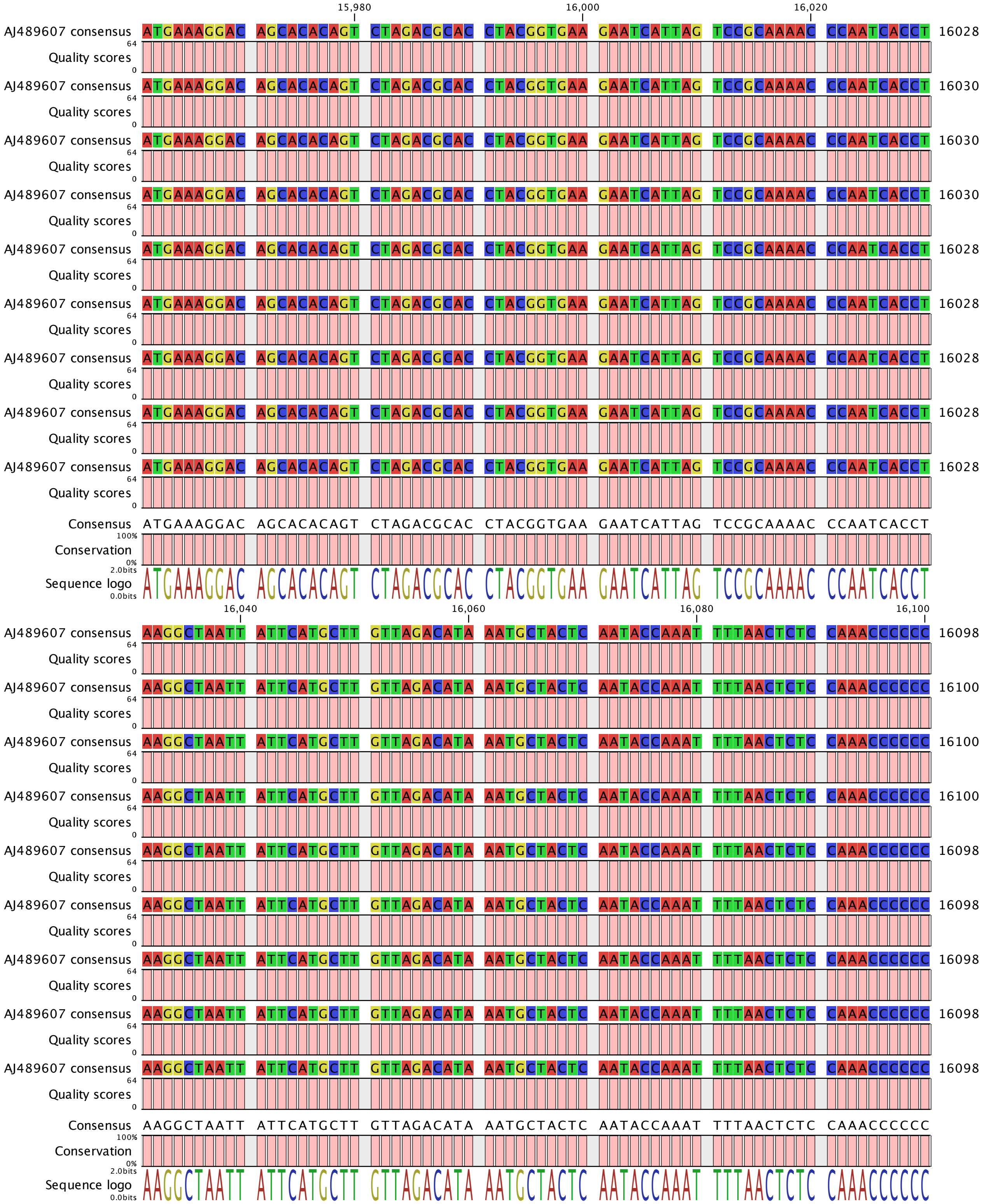
15,980
16,000
16,020
AJ489607 consensus ATGAAAGGAC AGCACACAGT CTAGACGCAC CTACGGTGAA GAATCATTAG TCCGCAAAAC CCAATCACCT 16028
Quality scores
AJ489607 consensus ATGAAAGGAC AGCACACAGT CTAGACGCAC CTACGGTGAA GAATCATTAG TCCGCAAAAC CCAATCACCT 16030
Quality scores
AJ489607 consensus ATGAAAGGAC AGCACACAGT CTAGACGCAC CTACGGTGAA GAATCATTAG TCCGCAAAAC CCAATCACCT 16030
Quality scores
AJ489607 consensus ATGAAAGGAC AGCACACAGT CTAGACGCAC CTACGGTGAA GAATCATTAG TCCGCAAAAC CCAATCACCT 16030
Quality scores
AJ489607 consensus ATGAAAGGAC AGCACACAGT CTAGACGCAC CTACGGTGAA GAATCATTAG TCCGCAAAAC CCAATCACCT 16028
Quality scores
AJ489607 consensus ATGAAAGGAC AGCACACAGT CTAGACGCAC CTACGGTGAA GAATCATTAG TCCGCAAAAC CCAATCACCT 16028
Quality scores
AJ489607 consensus ATGAAAGGAC AGCACACAGT CTAGACGCAC CTACGGTGAA GAATCATTAG TCCGCAAAAC CCAATCACCT 16028
Quality scores
AJ489607 consensus ATGAAAGGAC AGCACACAGT CTAGACGCAC CTACGGTGAA GAATCATTAG TCCGCAAAAC CCAATCACCT 16028
Quality scores
AJ489607 consensus ATGAAAGGAC AGCACACAGT CTAGACGCAC CTACGGTGAA GAATCATTAG TCCGCAAAAC CCAATCACCT 16028
Quality scores
Consensus ATGAAAGGAC AGCACACAGT CTAGACGCAC CTACGGTGAA GAATCATTAG TCCGCAAAAC CCAATCACCT
Conservation
Sequence logo
16,040
16,060
16,080
16,100
AJ489607 consensus AAGGCTAATT ATTCATGCTT GTTAGACATA AATGCTACTC AATACCAAAT TTTAACTCTC CAAACCCCCC 16098
Quality scores
AJ489607 consensus AAGGCTAATT ATTCATGCTT GTTAGACATA AATGCTACTC AATACCAAAT TTTAACTCTC CAAACCCCCC 16100
Quality scores
AJ489607 consensus AAGGCTAATT ATTCATGCTT GTTAGACATA AATGCTACTC AATACCAAAT TTTAACTCTC CAAACCCCCC 16100
Quality scores
AJ489607 consensus AAGGCTAATT ATTCATGCTT GTTAGACATA AATGCTACTC AATACCAAAT TTTAACTCTC CAAACCCCCC 16100
Quality scores
AJ489607 consensus AAGGCTAATT ATTCATGCTT GTTAGACATA AATGCTACTC AATACCAAAT TTTAACTCTC CAAACCCCCC 16098
Quality scores
AJ489607 consensus AAGGCTAATT ATTCATGCTT GTTAGACATA AATGCTACTC AATACCAAAT TTTAACTCTC CAAACCCCCC 16098
Quality scores
AJ489607 consensus AAGGCTAATT ATTCATGCTT GTTAGACATA AATGCTACTC AATACCAAAT TTTAACTCTC CAAACCCCCC 16098
Quality scores
AJ489607 consensus AAGGCTAATT ATTCATGCTT GTTAGACATA AATGCTACTC AATACCAAAT TTTAACTCTC CAAACCCCCC 16098
Quality scores
AJ489607 consensus AAGGCTAATT ATTCATGCTT GTTAGACATA AATGCTACTC AATACCAAAT TTTAACTCTC CAAACCCCCC 16098
Quality scores
Consensus AAGGCTAATT ATTCATGCTT GTTAGACATA AATGCTACTC AATACCAAAT TTTAACTCTC CAAACCCCCC
Conservation
Sequence logo

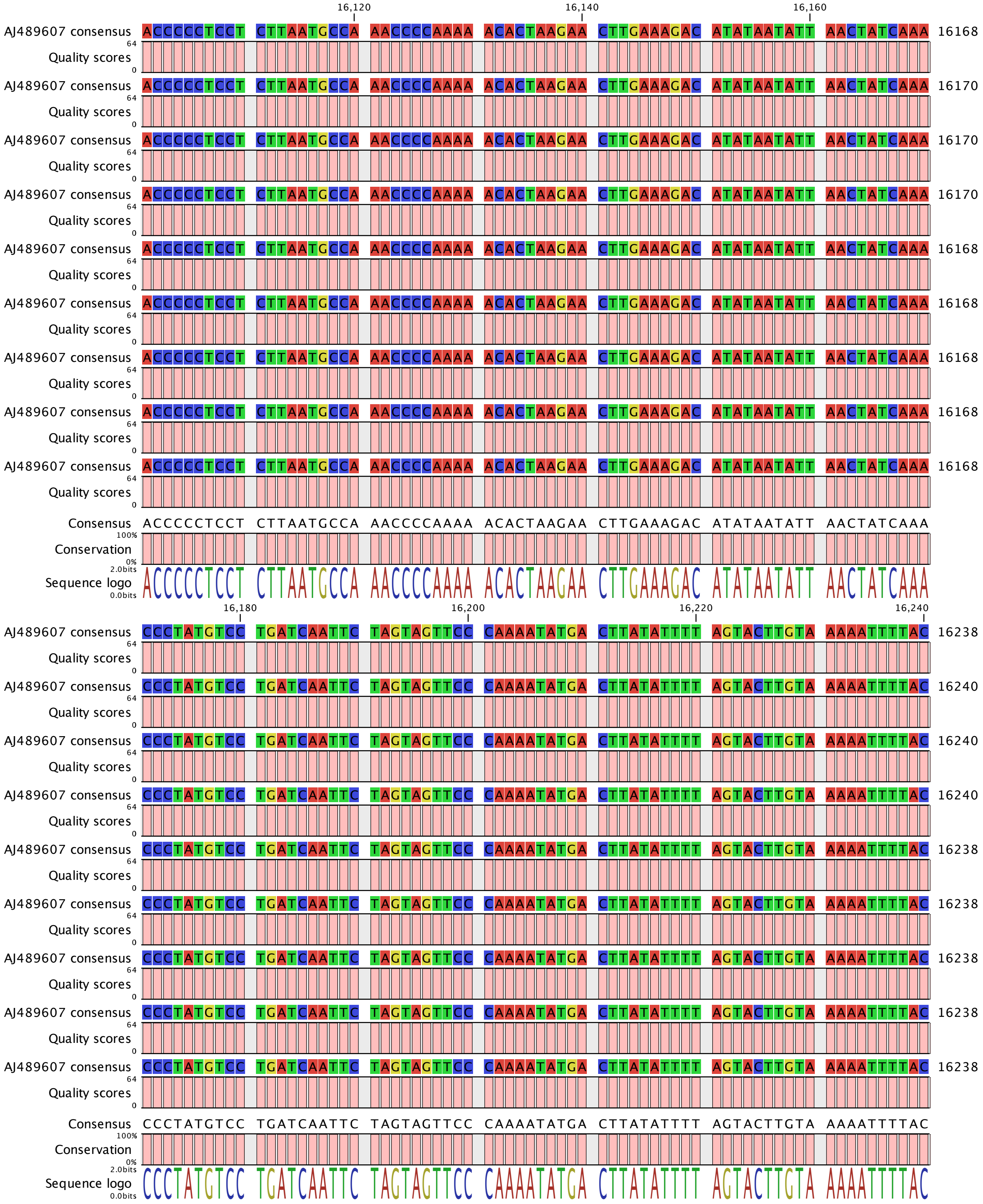
16,120
16,140
16,160
AJ489607 consensus ACCCCCTCCT CTTAATGCCA AACCCCAAAA ACACTAAGAA CTTGAAAGAC ATATAATATT AACTATCAAA 16168
Quality scores
AJ489607 consensus ACCCCCTCCT CTTAATGCCA AACCCCAAAA ACACTAAGAA CTTGAAAGAC ATATAATATT AACTATCAAA 16170
Quality scores
AJ489607 consensus ACCCCCTCCT CTTAATGCCA AACCCCAAAA ACACTAAGAA CTTGAAAGAC ATATAATATT AACTATCAAA 16170
Quality scores
AJ489607 consensus ACCCCCTCCT CTTAATGCCA AACCCCAAAA ACACTAAGAA CTTGAAAGAC ATATAATATT AACTATCAAA 16170
Quality scores
AJ489607 consensus ACCCCCTCCT CTTAATGCCA AACCCCAAAA ACACTAAGAA CTTGAAAGAC ATATAATATT AACTATCAAA 16168
Quality scores
AJ489607 consensus ACCCCCTCCT CTTAATGCCA AACCCCAAAA ACACTAAGAA CTTGAAAGAC ATATAATATT AACTATCAAA 16168
Quality scores
AJ489607 consensus ACCCCCTCCT CTTAATGCCA AACCCCAAAA ACACTAAGAA CTTGAAAGAC ATATAATATT AACTATCAAA 16168
Quality scores
AJ489607 consensus ACCCCCTCCT CTTAATGCCA AACCCCAAAA ACACTAAGAA CTTGAAAGAC ATATAATATT AACTATCAAA 16168
Quality scores
AJ489607 consensus ACCCCCTCCT CTTAATGCCA AACCCCAAAA ACACTAAGAA CTTGAAAGAC ATATAATATT AACTATCAAA 16168
Quality scores
Consensus ACCCCCTCCT CTTAATGCCA AACCCCAAAA ACACTAAGAA CTTGAAAGAC ATATAATATT AACTATCAAA
Conservation
Sequence logo
16,180
16,200
16,220
16,240
AJ489607 consensus CCCTATGTCC TGATCAATTC TAGTAGTTCC CAAAATATGA CTTATATTTT AGTACTTGTA AAAATTTTAC 16238
Quality scores
AJ489607 consensus CCCTATGTCC TGATCAATTC TAGTAGTTCC CAAAATATGA CTTATATTTT AGTACTTGTA AAAATTTTAC 16240
Quality scores
AJ489607 consensus CCCTATGTCC TGATCAATTC TAGTAGTTCC CAAAATATGA CTTATATTTT AGTACTTGTA AAAATTTTAC 16240
Quality scores
AJ489607 consensus CCCTATGTCC TGATCAATTC TAGTAGTTCC CAAAATATGA CTTATATTTT AGTACTTGTA AAAATTTTAC 16240
Quality scores
AJ489607 consensus CCCTATGTCC TGATCAATTC TAGTAGTTCC CAAAATATGA CTTATATTTT AGTACTTGTA AAAATTTTAC 16238
Quality scores
AJ489607 consensus CCCTATGTCC TGATCAATTC TAGTAGTTCC CAAAATATGA CTTATATTTT AGTACTTGTA AAAATTTTAC 16238
Quality scores
AJ489607 consensus CCCTATGTCC TGATCAATTC TAGTAGTTCC CAAAATATGA CTTATATTTT AGTACTTGTA AAAATTTTAC 16238
Quality scores
AJ489607 consensus CCCTATGTCC TGATCAATTC TAGTAGTTCC CAAAATATGA CTTATATTTT AGTACTTGTA AAAATTTTAC 16238
Quality scores
AJ489607 consensus CCCTATGTCC TGATCAATTC TAGTAGTTCC CAAAATATGA CTTATATTTT AGTACTTGTA AAAATTTTAC 16238
Quality scores
Consensus CCCTATGTCC TGATCAATTC TAGTAGTTCC CAAAATATGA CTTATATTTT AGTACTTGTA AAAATTTTAC
Conservation
Sequence logo

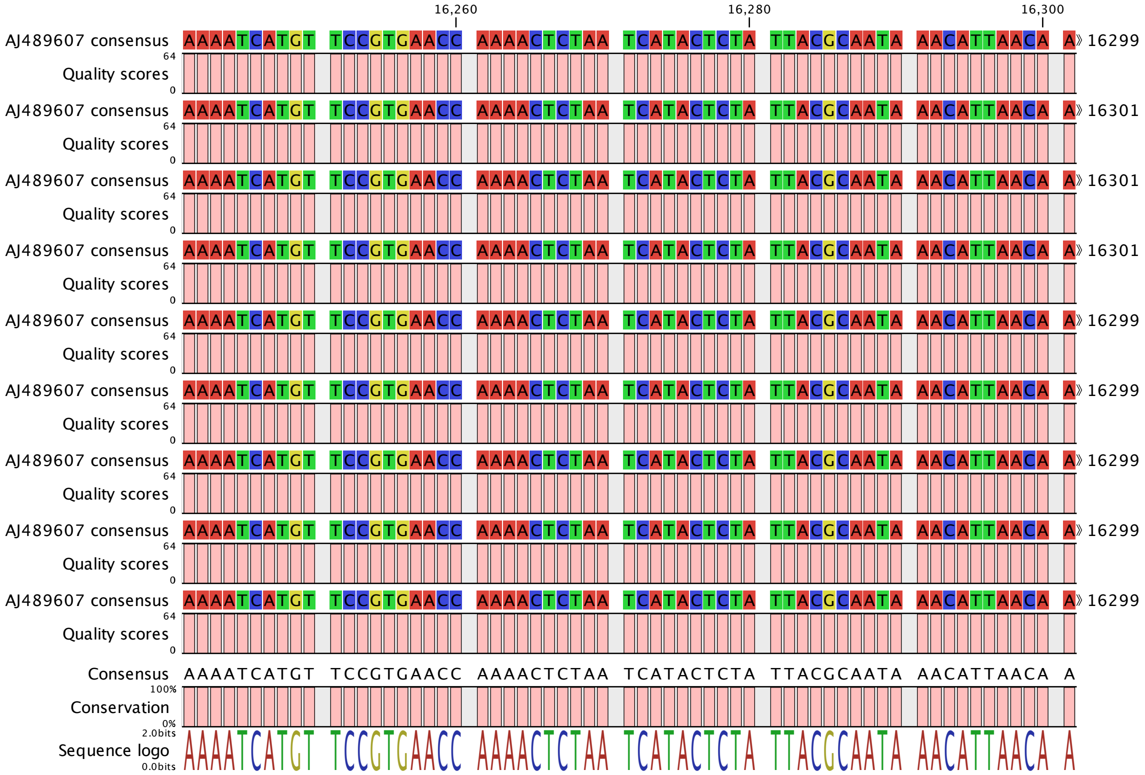
16,260
16,280
16,300
AJ489607 consensus AAAATCATGT TCCGTGAACC AAAACTCTAA TCATACTCTA TTACGCAATA AACATTAACA A 16299
Quality scores
AJ489607 consensus AAAATCATGT TCCGTGAACC AAAACTCTAA TCATACTCTA TTACGCAATA AACATTAACA A 16301
Quality scores
AJ489607 consensus AAAATCATGT TCCGTGAACC AAAACTCTAA TCATACTCTA TTACGCAATA AACATTAACA A 16301
Quality scores
AJ489607 consensus AAAATCATGT TCCGTGAACC AAAACTCTAA TCATACTCTA TTACGCAATA AACATTAACA A 16301
Quality scores
AJ489607 consensus AAAATCATGT TCCGTGAACC AAAACTCTAA TCATACTCTA TTACGCAATA AACATTAACA A 16299
Quality scores
AJ489607 consensus AAAATCATGT TCCGTGAACC AAAACTCTAA TCATACTCTA TTACGCAATA AACATTAACA A 16299
Quality scores
AJ489607 consensus AAAATCATGT TCCGTGAACC AAAACTCTAA TCATACTCTA TTACGCAATA AACATTAACA A 16299
Quality scores
AJ489607 consensus AAAATCATGT TCCGTGAACC AAAACTCTAA TCATACTCTA TTACGCAATA AACATTAACA A 16299
Quality scores
AJ489607 consensus AAAATCATGT TCCGTGAACC AAAACTCTAA TCATACTCTA TTACGCAATA AACATTAACA A 16299
Quality scores
Consensus AAAATCATGT TCCGTGAACC AAAACTCTAA TCATACTCTA TTACGCAATA AACATTAACA A
Conservation
Sequence logo
